# Development of a Screening Workflow for the Halogenation of Nitrogen Heterocycles with Vanadium-Dependent Haloperoxidases

**DOI:** 10.1021/acsbiomedchemau.6c00157

**Published:** 2026-10-12

**Authors:** Carter U. Brzezinski, Sergio A. Solis, Hannah S. Slocumb, John Bacsa, Kyle F. Biegasiewicz

**Affiliations:** † Department of Chemistry, 1371 Emory University, Atlanta, Georgia 30322, United States; ‡ Department of Chemistry, University of Utah, Salt Lake City, Utah 84112, United States

**Keywords:** halogenation, heterocycles, vanadium, haloperoxidase, biocatalysis

## Abstract

The halogenation of nitrogen heterocycles is a privileged transformation for accessing important synthetic intermediates and biologically active targets in medicinal chemistry. Despite the importance of N-heterocycle halogenation, current methods rely on reagents or catalysts that are largely unselective and produce a significant number of waste products. With an emerging interest in using halogenating enzymes to address these challenges, the vanadium-dependent haloperoxidase class of enzymes has emerged as a powerful platform for halogenation reactions under a uniquely broad set of reaction conditions. Herein, we report the development of a screening workflow for the halogenation of heterocycles with chloroperoxidase from *Curvularia inaequalis* (*Ci*VCPO). Using a high-throughput screening strategy, representative model heterocycles are screened for direct chlorination and bromination reactions, and the optimized conditions are translated to an extended substrate scope in moderate to high yields. The volume of high-throughput data obtained allowed for the development of a predictive model for the substrate halogenation conditions. This screening platform is applied to established industrial intermediates and to the discovery of previously undiscovered enzymatic nitrogen-chlorine bond formation and decarboxylative halogenation reactions.

## Graphical abstract

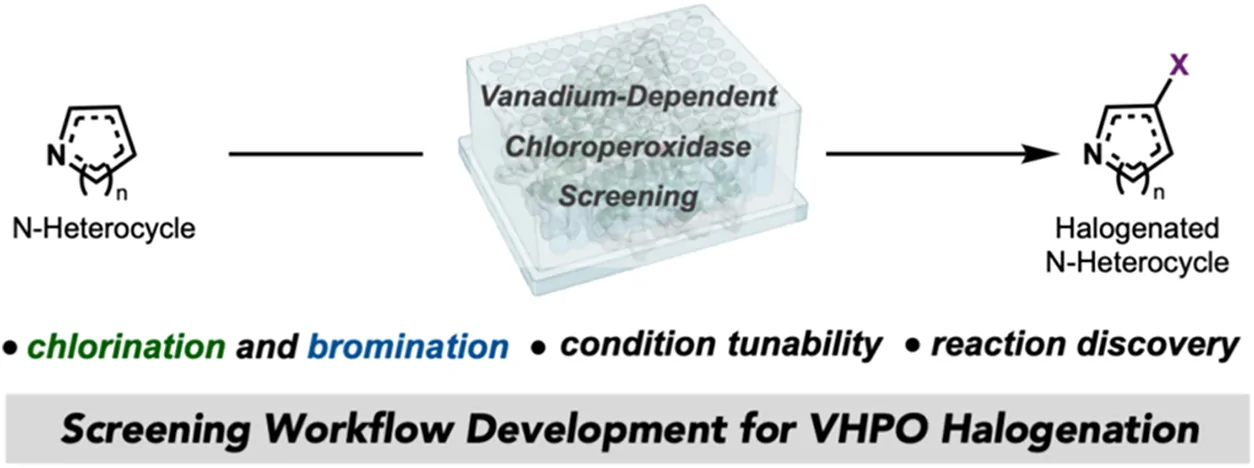

## Introduction

Halogenated nitrogen heterocycles (N-heterocycles) are important targets and synthetic intermediates in chemical synthesis. Halogen atoms are often incorporated into biologically active molecules as an optimization handle for improving their metabolic stability, target binding, and pharmacokinetic profile (Figure 1A­(i)).^1–6^ They are also highly versatile synthetic precursors in chemical transformations that are critical for the synthesis of biologically active molecules.^7–10^ Despite the importance of halogenated N-heterocycles, methods for direct halogenation of N-heterocycles rely on reagent-based methods^11,12^ or catalyst systems using transition metals,^13,14^ sulfur-mediation,^15^ photoredox catalysts,^16^ or organocatalysts^17^ that are unselective, have limited tunability across structurally diverse substrate scopes, or produce large quantities of waste (Figure 1A­(ii)). As a result, the development of selective, tunable, and sustainable methods for halogenation of N-heterocycles remains of great interest to the scientific community.

**Figure 1:**
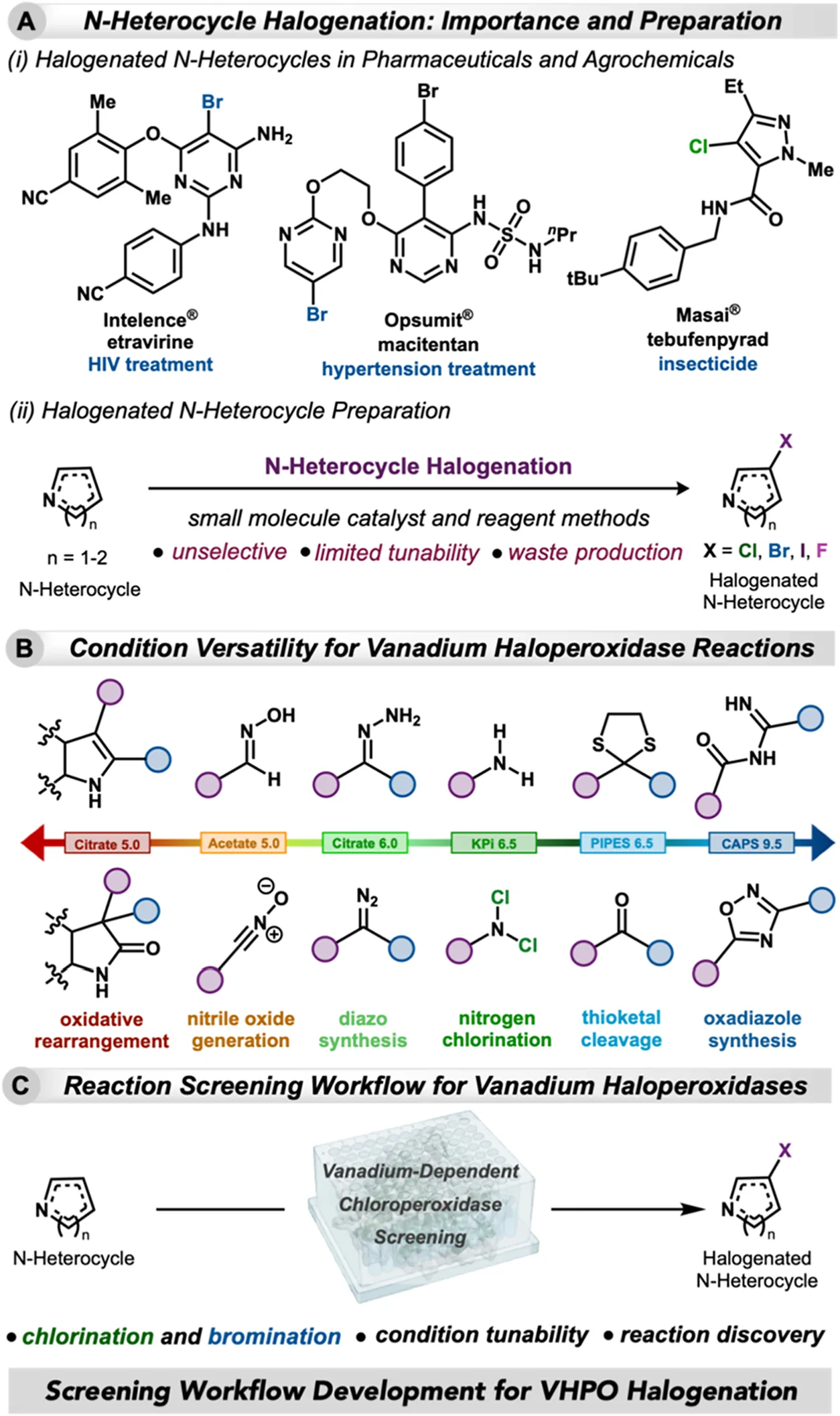
N-heterocycle halogenation overview. (A) Importance and preparation of halogenated N-heterocycles. (B) Condition versatility of vanadium haloperoxidases. (C) Proposed screening workflow for vanadium haloperoxidase halogenation.

Enzymes are an attractive option for N-heterocycle halogenation because of their inherent selectivity and sustainability parameters.^18–21^ Among the wide range of halogenating enzymes in nature,^22–25^ the vanadium-dependent haloperoxidase (VHPO) class of enzymes has emerged as a promising biocatalytic platform for chemical synthesis because of their lack of requirement for an exogenous turnover system and their ability to act on a wide range of organic substrates in reactions outside of their natural substrate scope.^26–35^ Our laboratory has discovered that one of the most attractive features of VHPOs is their ability to operate under a broad range of pH conditions and organic cosolvents in a plethora of reaction types (Figure 1B).^36–42^ With these unique tunability parameters available in VHPO-catalyzed transformations, we were drawn to the development of an efficient screening workflow for halogenation of heterocycles with VHPOs. As a proof of principle, we chose to develop the screening workflow around the vanadium-dependent chloroperoxidase from *Curvularia inaequalis* (*Ci*VCPO)^43^ because of its documented ability to catalyze selective chlorination and bromination in a wide range of chemical reactions.^30–32,36,39,41,42,44^ Herein, we report the establishment of a screening workflow for the development of chlorination and bromination reactions on a broad selection of N-heterocycles with *Ci*VCPO (Figure 1C).

## Results and Discussion

Our study began by designing a 64-well screening plate for the chlorination and bromination of 2-methyl-2*H*-indazole (**1**). This model substrate was chosen on the basis of its structural similarity to indoles that serve as highly reactive compounds in VHPO chemistry.^34,36,42,44,45^ Based on our own previous experiences in reaction development,^36–42^ our ‘haloplate’ design prioritized testing combinations of buffer, pH, and cosolvent, as we have demonstrated that these are often the most critical parameters for halogenation optimization. Using a standard set of reaction parameters for loadings of enzyme (*Ci*VCPO, 0.00005 mol %), sodium orthovanadate (Na_3_VO_4_, 1 mM equiv), potassium halide salt (KX, 1.1 equiv), hydrogen peroxide (H_2_O_2_, 2.0 equiv), buffer concentration (50 mM), cosolvent (20% v/v), and substrate loading (10 mM, 0.004 mmol), the haloplate features a set of 32 reaction conditions that vary in buffer content (50 mM, pH 4–10) and cosolvent type (DMSO, DMF, MeCN, THF) for both chlorination and bromination reactions (Figure 2, Step 1). For initial yield determination in the haloplate, liquid chromatography percent (LCAP) yield was used to determine optimal conditions for preparative-scale reactions. Applying the designed haloplate conditions to indazole **1** demonstrated that the optimal conditions for chlorination (50 mM MES pH 6.0, 20% MeCN) varied significantly from the optimal bromination conditions (50 mM Acetate pH 4.0, 20% THF), providing chloroindazole **2** in 53% LCAP yield and bromoindazole **3** in 87% LCAP yield, validating the relevance of these two parameters on reaction performance (Figure 2, Step 2). Conveniently, the entire screening protocol is set up completely open to air and does not require any special handling of the biocatalyst or reagents (Figures S1–S5). To ensure consistency across the plate, it was discovered that a 15 min preincubation stirring of all reaction components prior to H_2_O_2_ addition gave optimal results and consistency across the 64 wells with <5% LCAP yield variation (Figures S6 and S7). Interestingly, we discovered that the haloplate was largely unaffected by changes in enzyme, Na_3_VO_4_, H_2_O_2_, halide, cosolvent, and substrate loadings for both chlorination (Figures S8–S14) and bromination (Figures S15–S21). Notably, the screen accommodates a wide range of solvents outside of the designed plate format for further versatility (Figure S12 for chlorination and Figure S19 for bromination). Moreover, the apparent solubility of the biocatalyst was maintained across the plate. The highest-performing conditions from this screen are then validated in vial format, and control conditions are run excluding *Ci*VCPO, Na_3_VO_4_, halide, and peroxide, in turn, to ensure all reaction components are necessary for reactivity and to ensure enzyme-mediated catalysis at higher pH (Figures S22 and S23). These reaction conditions are then applied to preparative-scale reactions (Figure 2, Step 3). In the case of halogenation of **1**, chloroindazole **2** was obtained in 53% isolated yield via flash chromatography, and bromoindazole **3** was obtained in 92% yield and required no further purification after workup (Figure 2, Step 4).

**Figure 2:**
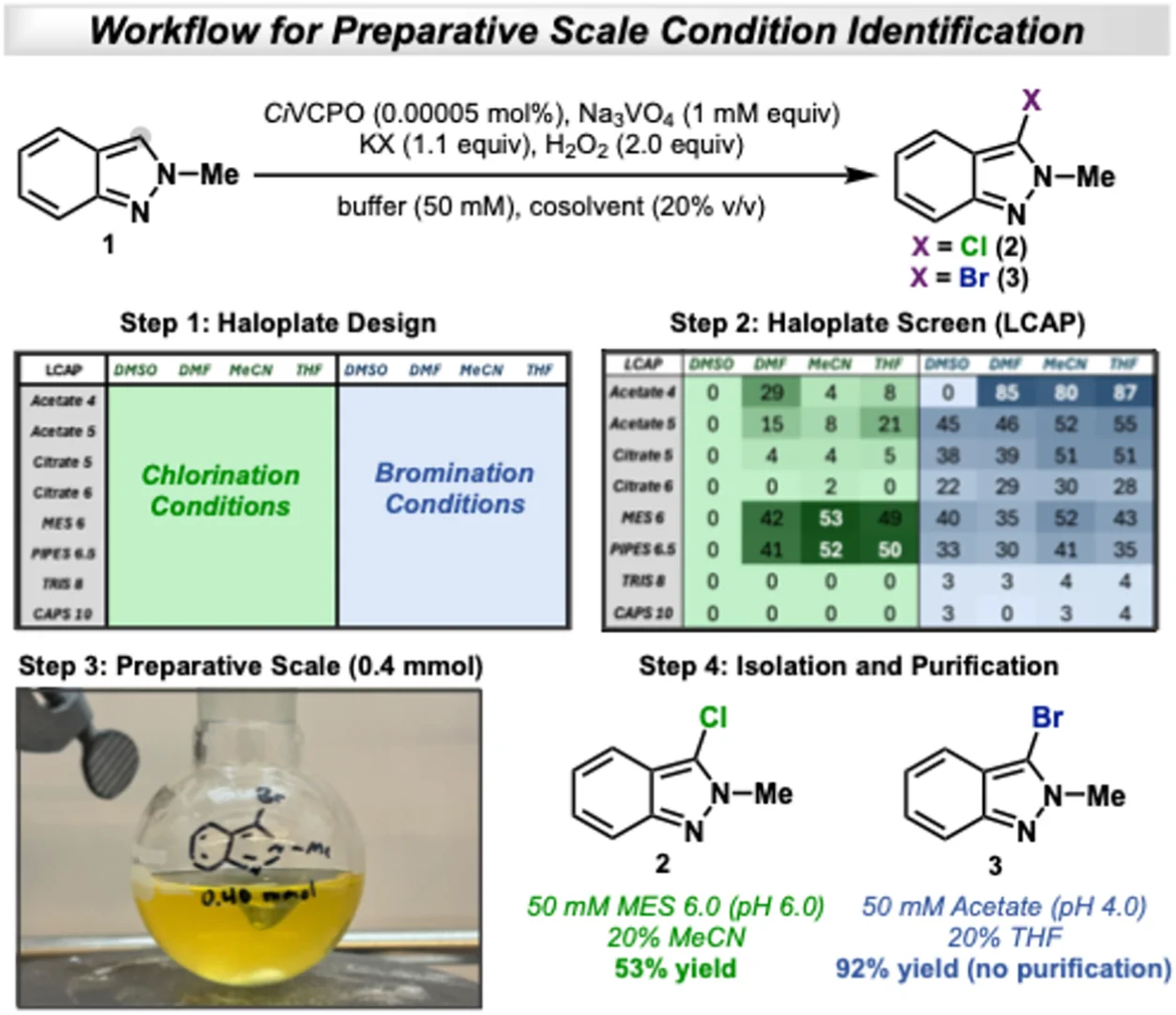
Workflow for preparative-scale condition identification.

With optimized conditions for chlorination and bromination of model indazole **1** in hand, application of these conditions to other substituted indazoles was explored. The developed conditions translated to the chlorination and bromination of four different indazoles to give halogenated indazoles **4–11** in trace to 97% yield (Figure 3A). Importantly, reanalyzing each substrate individually for optimal buffer conditions confirmed that the initially discovered chlorination and bromination conditions are still the highest-performing option (Figure S24). A noticeable feature of these experiments is that bromination conditions translated effectively to a broader range of substrates, while chlorination translated only to compounds that were more structurally related to the model compound, suggesting enzyme binding differences for bromination and chlorination reactions in *Ci*VCPO. Performing a similar analysis on structurally distinct N-heterocycle 1-phenyl-1*H*-pyrazole (**12**) gave similar results, where MES and Acetate buffer systems at pH = 6 were the highest-performing conditions for chlorinations and bromination, respectively. While MeCN remained the top-performing cosolvent for chlorination, it also prevailed as the best solvent for bromination for this substrate class. Another interesting feature is the comparatively low chlorination activity for the model pyrazole **12**. Despite this observation, the top-performing chlorination and bromination conditions translated cleanly to four different structurally diverse substituted pyrazoles in 73–97% isolated yield for chlorination and 85–95% isolated yield for bromination (Figure 3, **15–22**), suggesting that even low reactivity on a model system substrate serves as a useful reactivity readout for application across new substituted substrates (Figure 3B). Finally, the azaindole class of N-heterocycles was explored using model substrate ethyl 1H-pyrrolo­[3,2-*b*]­pyridine-2-carboxylate (**23**). In this case, MES buffer systems were identified as optimal for both bromination and chlorination, but THF was identified as the best cosolvent for chlorination, and MeCN was best suited for bromination of **23**, providing **24** and **25** in 82% and 92% yield, respectively. Intriguingly, the conditions were readily applied to the halogenation of arene nitrogen isomers to give halogenated azaindoles **26–31** in moderate to high yield. Notably, for the corresponding methyl analogues, simply changing the buffer type to citrate at the same pH provided the corresponding halogenated azaindoles (**32–33**) in high yield (Figure 3C). This would suggest that while pH will serve as a primary readout for reactivity, another tunability parameter is available in the buffer type applied in VHPO halogenation reactions. A similar observation has been observed in previously developed VHPO-mediated transformations.^36–42^

**Figure 3:**
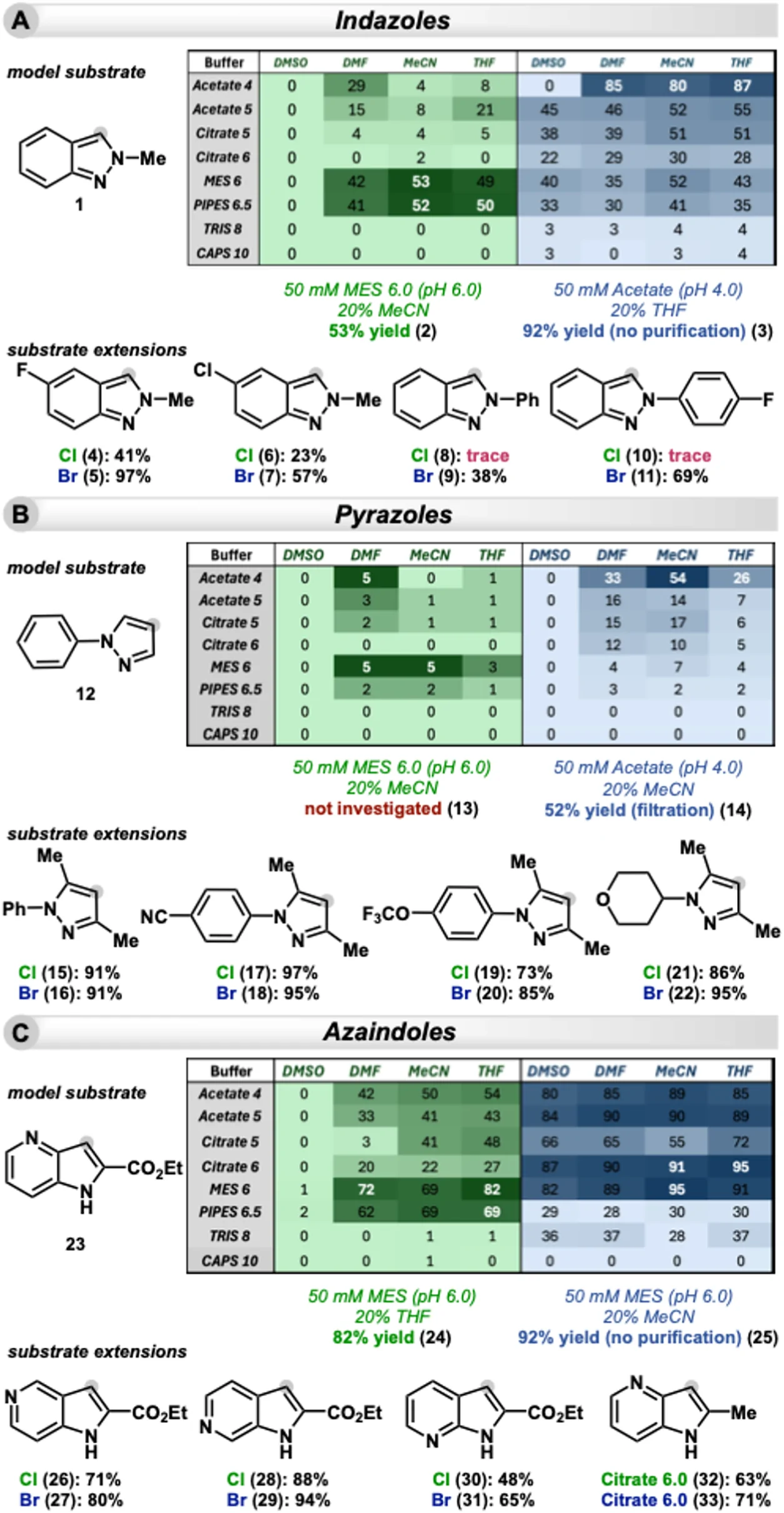
Model substrate and substrate extensions. See Supporting Information for experimental details.

We next investigated the halogenation of imidazo­[1,2-*a*]­pyridine (Figure 4, **34**) using our described protocol and successfully identified high-yielding chlorination (50 mM citrate, pH 5.0, 20% THF) and bromination (50 mM TRIS, pH 6.0, 20% THF) conditions. While these conditions did translate to some structurally related imidazopyridines (Figure 4, **37–40**) in high yield, they were less successful for imidazopyridine **41** or other related fused nitrogen heterocycles. We chose to individually analyze each of these fused heterocyclic substrates to better understand the similarities and differences in optimal halogenation conditions based on the different substitution patterns. This abbreviated optimization enabled successful halogenation of a much wider variety of imidazo­[1,2-*a*]­pyridines, pyrazolo­[1,5-*a*]­pyridines, pyrazolo­[1,5-*a*]­pyrimidines, and imidazo­[1,5-*a*]­pyridines (Figure 4, **41–57)** in excellent yield and further demonstrated the wide variety of conditions tolerated by *Ci*VCPO. This also revealed the impact of small changes in substitution under optimal reaction conditions, for example, in products **48–51**. Bromination of imidazo­[1,5-*a*]­pyridine **58** readily produced bromide **59** in an excellent yield without purification. However, chlorination conditions instead yielded picolinamide **60** via a cryptic chlorination pathway.^46^ Control experiments and a proposed mechanism for this transformation are found in Figure S25.

**Figure 4:**
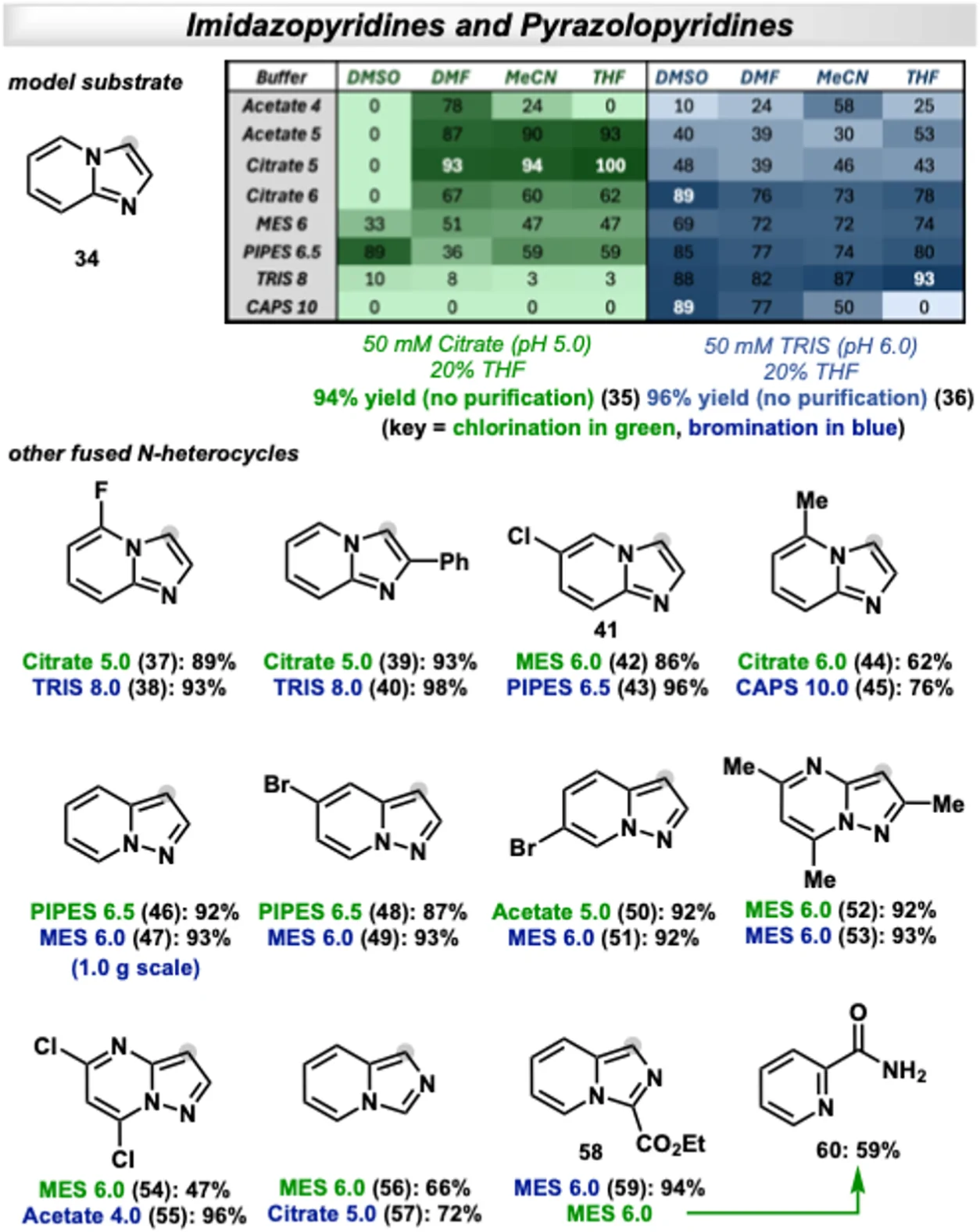
Buffer tuning for N-heterocycle halogenation. See Supporting Information for additional experimental details.

To test our screening workflow on industrially relevant substrates, we examined the chlorination of pyrazole **61** by *Ci*VCPO. Chloropyrazole **62** is a key precursor to the insecticide tebufenpyrad.^4^ Like the previously analyzed pyrazole **12**, MES and PIPES buffer systems were especially effective for the desired chlorination. With these identified conditions, the chlorination of **61** was performed on a 5.0 mmol scale with C*i*VCPO-clarified lysate, yielding **62** in 87% yield via a simple filtration from hot water (Figure 5A). As an additional case study for VHPO-mediated halogenation reactions, we investigated the bromination of pyrrole **63** to give bromopyrrole **64**, a valuable intermediate toward 6-bromo-2,4,5-trichloropyrrolo-[2,1-*f*]­1,2,4-triazine, an anticancer agent precursor by BMS.^47^ Typical reagent-based bromination conditions give poor selectivity for the desired bromopyrrole **64** over dibrominated pyrrole **65**, which could only be controlled with HFIP as cosolvent (88:9 – **64**:**65**).^47^ Using our described screening protocol, we found optimal conditions with purified *Ci*VCPO with improved bromination selectivity (96:3 – **64**:**65**). These conditions were successfully translated to a 5.0 mmol scale with *Ci*VCPO-clarified lysate without any further modification of conditions, and pyrrole **64** was easily isolated via filtration from heptane (Figure 5B).

**Figure 5:**
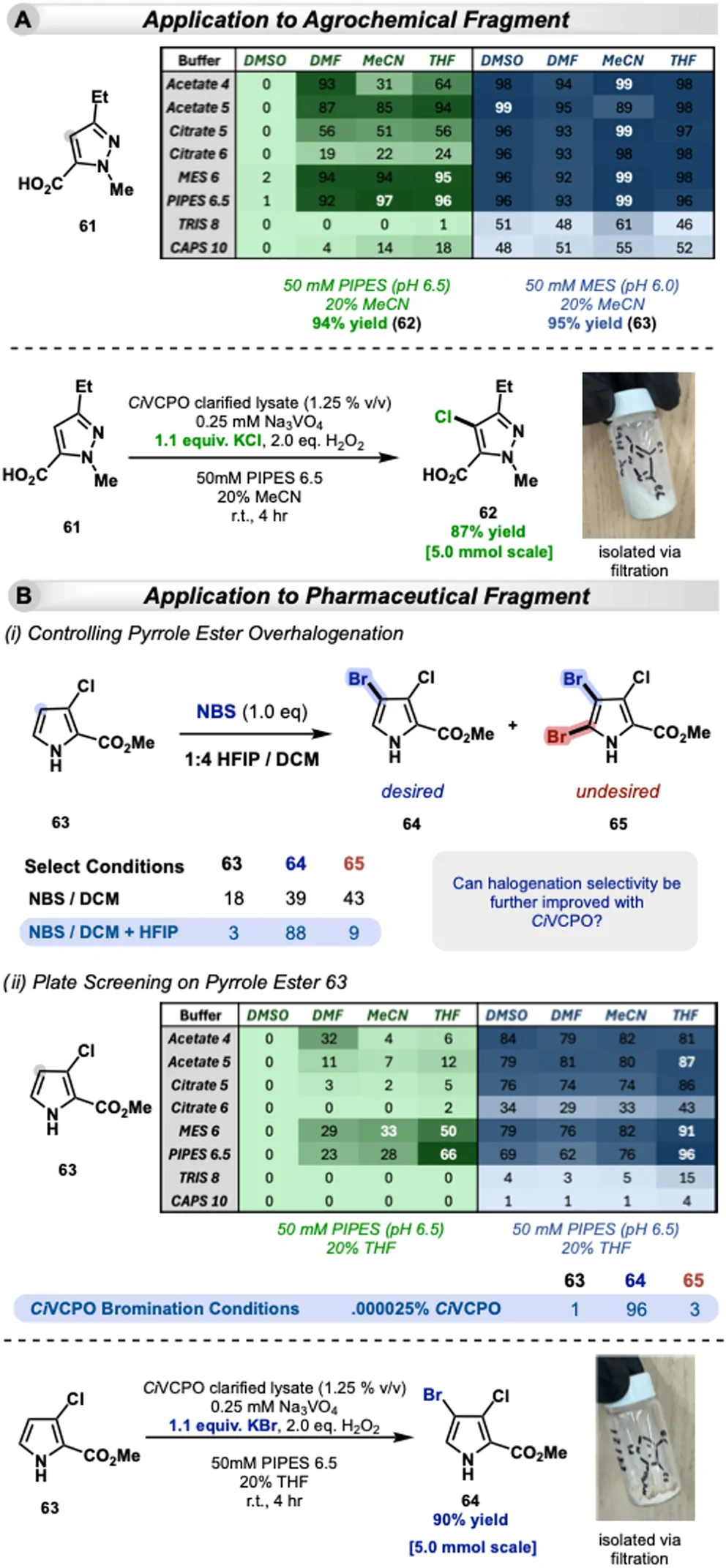
Application on agrochemical and pharmaceutical intermediates. See Supporting Information for additional experimental details.

We noticed that the buffer conditions for *Ci*VCPO halogenations could vary drastically between substrates. There was no discernible pattern for these variations; however, ideally, we would avoid screening all buffer conditions across each new substrate in future applications of this methodology. Therefore, we investigated whether we could develop predictive models of optimal reaction pH using computational data (Figure 6). Predicting reaction conditions can be challenging; however, there has been some success in predicting optimal pH for enzymatic catalysis reactions.^48,49^ In this case, we hoped that predicting optimal pH for each reaction could reveal mechanistic similarities or differences between bromination and chlorination.

**Figure 6:**
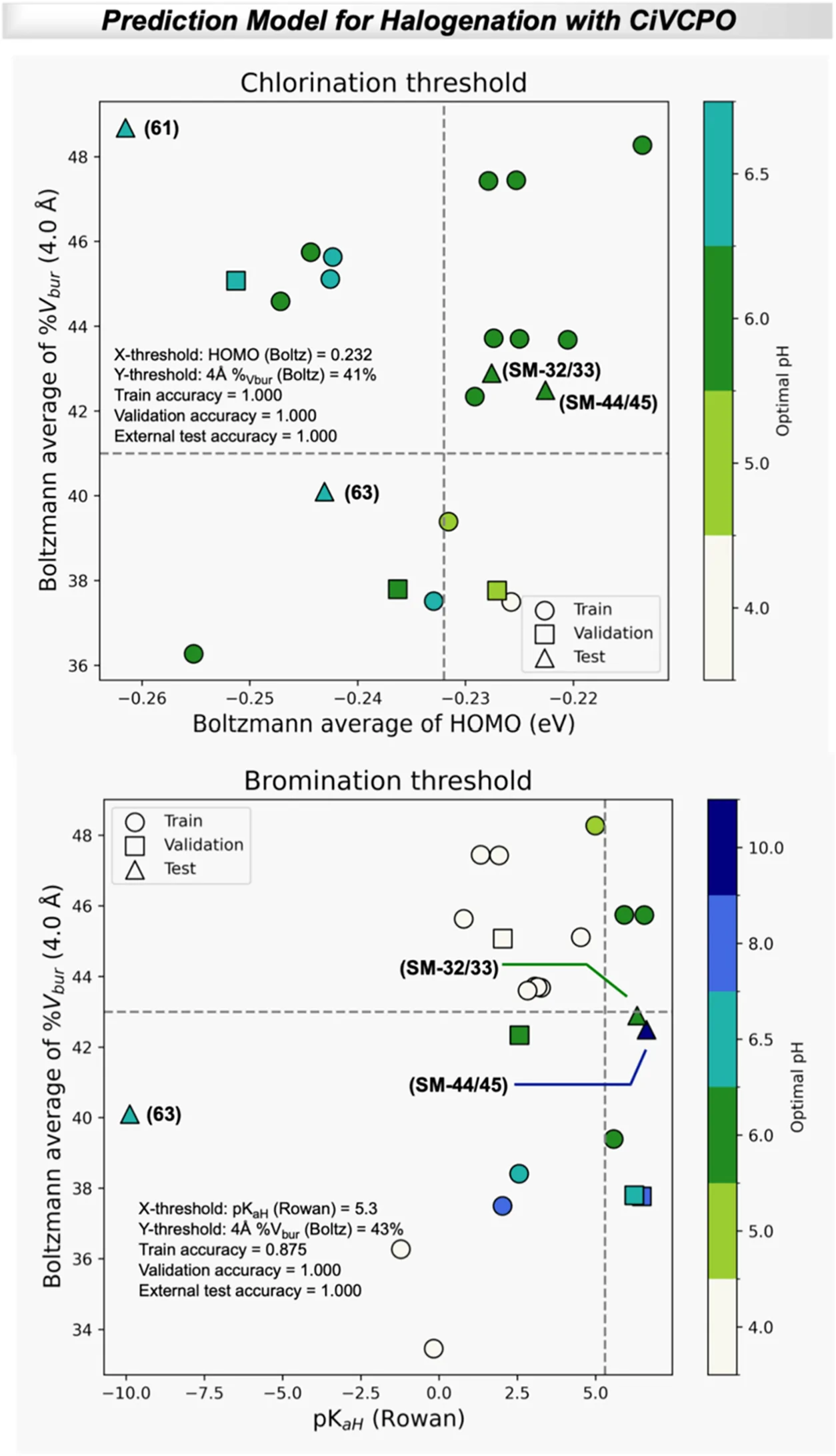
Prediction model for halogenation with *Ci*VCPO

For each substrate, we calculated xTB-level conformer ensembles using CREST (GFN2) to identify energetically relevant structures. Then, we optimized these structures using DFT (PBE0-D3­(BJ)/def2-SVP) and calculated single-point energies (PBE0-D3­(BJ)/def2-TZVP). From these calculations, we extracted electronic and steric molecular features, focusing on global parameters (e.g., HOMO, solvent-accessible surface area, etc.) and atom-specific parameters for the halogenated carbon (e.g., NBO charge, Buried Volume, etc.). For a full overview of all parameters, see Figure S9. Features were then condensed across the ensembles to the Boltzmann average; other representative features (e.g., ensemble maximum, standard deviation, etc.) were not used, as many substrates only had one energetically relevant conformer. In addition to DFT-calculated features, we also considered predicted p*K*_a_’s for the protonated substrates in water using the Rowan^50^ and DeepSynthesis^51^ ML p*K*_a_ predictors.

Generally, we were interested in identifying threshold models that bin substrates into quadrants based on the optimal pH. We recognize that this approach is a simplification of the reaction and disregards several important reaction factors. However, our goals for this were 2-fold: that this would allow us to identify some of the most impactful factors in each reaction and generally allow for the pH prediction. Further, we point out that these models will be specific for bromination or chlorination with this specific *Ci*VCPO catalyst, though we hope that through careful model evaluation and extrapolation to external test sets, we will be able to predict most potential substrates. We chose to focus on threshold-based modeling due to the discrete nature of our data (i.e., buffers tested were only at discrete values such as 5.0, 6.0, and 6.5). From the number of data points, examining two parameters seemed appropriate. We envisioned looking for 2D plots with one computational feature on each axis, with threshold values that allow for differentiation of substrates based upon optimal pH. This would allow us to identify stereoelectronic trends leading to high and low optimal pH, as well as allowing for fewer required testing conditions for the application of these reactions to new substrates. To create these models, we separate the data into three sections: training data, which the model uses to identify thresholds; validation data, which tests the accuracy of the model; and external test data, which tests the ability of the model to predict outside known substrate structures. The best models are chosen based on the statistical performance of each of these data categories, as well as the interpretability of the features.

We investigated two-parameter threshold models based on a cutoff value of pH < 5.5, then manually identified models that further binned pH values by visualization. Most substrates were included in modeling efforts; however, if substrates had yields within ±10% for both high and low pHs, then these were excluded. Additionally, if the maximum yield was <10%, the substrate was excluded. Data was split into training and validation sets in an 80:20 ratio using a Kennard-Stone algorithm.^52^ An external test set was chosen after preliminary scope evaluation based on structures that were most dissimilar to structures in the training and validation sets. These consisted of methyl-3-chloro-1-methyl-1*H*-pyrrole-2-carboxylate (**63**), as it is the only pyrrole; 3-ethyl-1-methyl-1*H*-pyrazole-5-carboxylic acid (**61**), as it is the only substrate containing a free acid; and both 5-methylimidazo­[1,2-α]­pyridine (**SM-44/45**) and 2-methyl-1*H*-pyrrolo­[3,2-β]­pyridine (**SM-32/33**) due to their inductive methyl substitutions, differentiating them from other substrates in their respective classes. Models were generated using code that used scikit-learn’s DecisionTreeClassifier function to identify double threshold models.^53^ Then, these models were evaluated based on training, validation, and external test statistics. Numerous threshold models showed similar performances, so preferred models were identified based on their abilities to further separate optimal pH past the cutoff threshold and based on the interpretability of the models. We chose the maximum number of thresholds to be two based on the data set size.

For chlorination, thresholds of HOMO = 0.232 eV and percent buried volume (%V_bur_) at 4.0 Å = 41% were identified. The HOMO of the molecule is generally reflective of nucleophilicity, while the 4Å %V_bur_ is indicative of the steric environment relevant to the halogen during nucleophilic addition, with a higher %V_bur_ correlating to great steric encumbrance. This dual threshold gives 100% accuracy for the training, validation, and external test sets. These thresholds identify a low optimal pH region (pH = 4–5) for substrates, featuring high HOMO and low 4Å %V_bur_. These substrates will be the most nucleophilic due to the lack of steric hindrance and high HOMO, so a lower pH may be required to temper reactivity by favoring the protonated species. For sterically encumbered substrates with a high HOMO, an optimal pH = 6 is observed, where substrates of varying steric profiles but low HOMO see optimal pH = 6–6.5. These less reactive substrates may require more moderate conditions for chlorination, leading to the more moderate optimal pH’s.

For bromination, thresholds of p*K*_aH_ (Rowan model) = 5.3 and 4Å %V_bur_ = 43% were identified. This dual threshold gives 87.5% accuracy for the training set and 100% accuracy for the validation and external test sets. The outliers include 1-phenyl-1H-pyrazole (**12**), which has a low yield, which may contribute to low accuracy, and 5,7-dichloropyrazolo-[1,5-*a*]-pyrimidine (**41**), which has a high yield across the 4–8 pH range, which may obfuscate accuracy. For substrates with a low p*K*_aH_ and high steric encumbrance, a lower optimal pH (4–5) is observed. Outside of this region, generally lower 4Å %V_bur_ leads to higher optimal pH. For bromination, it is harder to differentiate between higher optimal pH (>6) substrates, as it is more common to see high yields of halogenation across multiple pHs for bromination than chlorination.

In both chlorination and bromination threshold models, 4Å %V_bur_ was identified as one threshold. Interestingly, for chlorination, a lower 4Å %V_bur_ was observed for substrates with low optimal pH, whereas for bromination, a higher 4Å %V_bur_ was observed for substrates with a high optimal pH. These opposing trends may indicate a deviation in mechanism, leading to differing preferences for protonated or unprotonated species.

While investigating the halogenation of indazoles with indazole **66**, we discovered a novel N-chlorination reaction catalyzed by *Ci*VCPO. This reaction was optimized using our developed screening protocol and was successful on a variety of other unsubstituted indazoles (**68–71**), albeit in moderate yield due to the instability of the N–Cl bond. A single X-ray crystal structure of **69** was obtained to unambiguously confirm the presence of the N–Cl bond, which, to the best of our knowledge, has never been observed in indazole systems (Figure 7(i)). Additional heterocycles including pyrazole, benzotriazole, and 2-phenylbenzimidazole were screened, but no conversion was observed. Bromination of indazole **66** or any N1-alkylated indazole was never observed under any reaction conditions. To address this limitation, we developed a decarboxylative process for decarboxylative halogenation on a variety of heterocycles (**35**, **36**, **46**, **47**, and **72**) in moderate to high yield (Figure 7(ii)).

**Figure 7:**
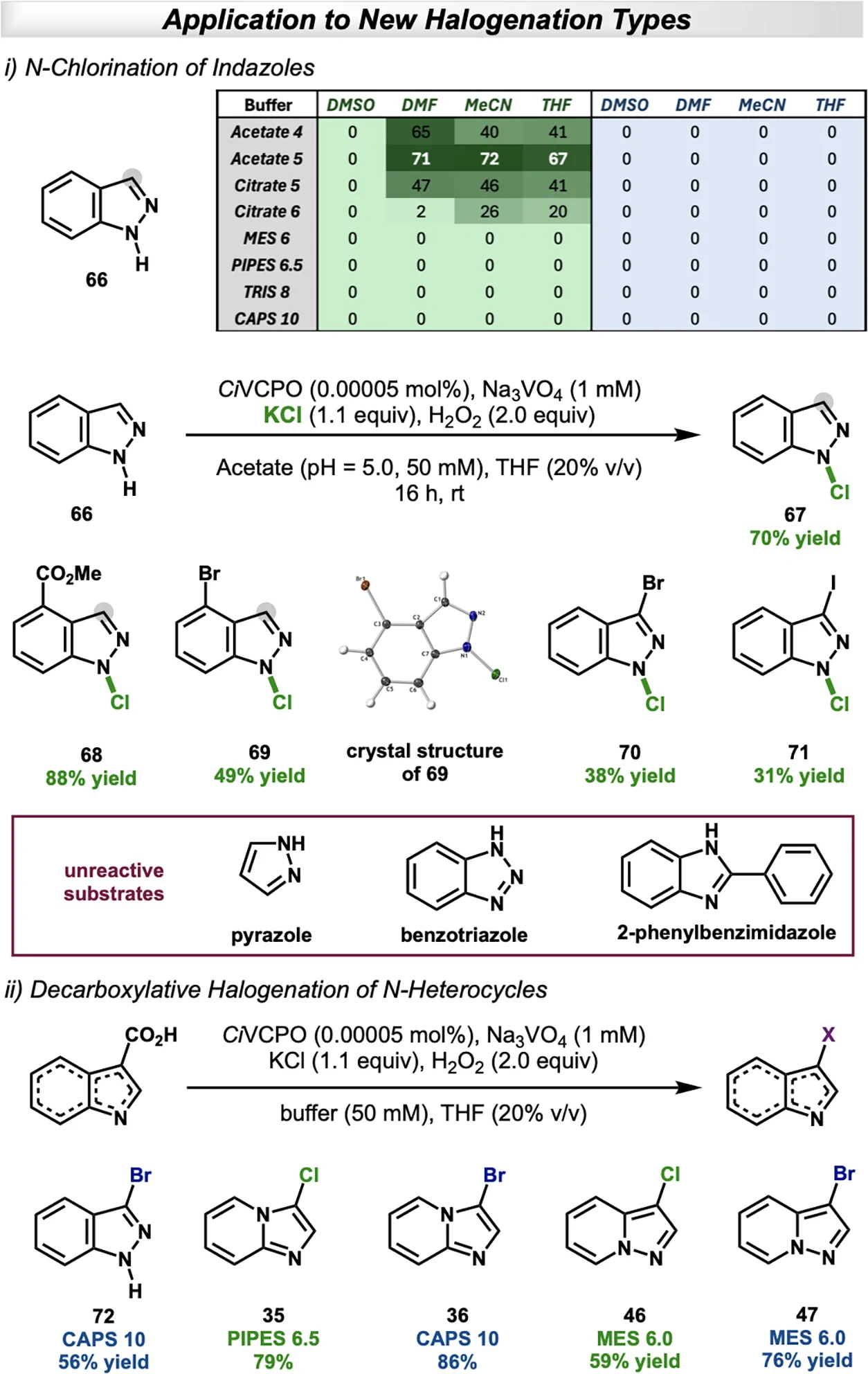
Extension to new halogenation types.

## Conclusion

In conclusion, an effective screening protocol for VHPO halogenation was developed. This study demonstrates the unique screening parameters available to VHPOs in reaction development and has been applied to the halogenation of a wide range of N-heterocycles and halogenation types. We anticipate that this technology will dramatically enhance the ability to optimize and discover reactions with VHPOs and allow for their further establishment as powerful catalyst platforms in chemoenzymatic synthesis.

## Methods and Experimental

### General Procedure for Halogenation in Well-Plates

For a detailed procedure including figures outlining stock preparation, reaction setup, preincubation, and analysis, see the Supporting Information.

### General Procedure for Halogenation in 1 Dram Vials

An enzyme aliquot of purified VHPO from *Curvularia inaequalis* (*Ci*VCPO, 10 μM, 0.5 mL) was removed from a −80 °C freezer and allowed to warm to room temperature over 5 min. The thawed aliquot was then combined with an aqueous solution of 250 mM Na_3_VO_4_ (40 μL) in a microcentrifuge tube and stored at room temperature. To ensure mixing, the resulting solution was spun in a mini centrifuge for 10 s and placed on the benchtop until further use. To a 1 dram vial, purified H_2_O (212 μL), cosolvent (40 μL, see individual entries for details), 500 mM buffer (40 μL, see individual entries for details), and 100 mM KCl or KBr (44 μL, 1.1 equiv) were added. A 100 mM stock of the substrate (0.004 mmol, 1 equiv, 40 μL) was added to the reaction mixture. Each vial was inverted 3x to ensure proper mixing, then 20 μL of the *Ci*VCPO stock was added, followed by a 10% stock of H_2_O_2_ (2.5 μL, 2 equiv) to initiate the reaction. The reactions were shaken either on a Heidolph Unimax shaker or a Heidolph Multi Reax (depending on the total number of reactions) for 16h. Upon completion, the reaction mixture was diluted with 650 μL of MeCN, transferred to a 1.5 mL Eppendorf tube, and centrifuged at 13,000 rpm for 5 min. 600 μL of the reaction mixture was removed for LCMS analysis (see Supporting Information for more details).

### General Procedure for Preparative-Scale Halogenation

Two enzyme aliquots of purified VHPO from *Curvularia inaequalis* (*Ci*VCPO, 10 μM, 1.0 mL) were removed from a −80 °C freezer and allowed to warm to room temperature over 5 min. The thawed aliquot was then combined with an aqueous solution of 250 mM Na_3_VO_4_ (160 μL, 0.1 equiv) in a microcentrifuge tube and stored at room temperature. To ensure mixing, the resulting solution was spun in a mini centrifuge for 10 s and placed on the benchtop until further use. To a 100 mL round-bottom flask containing a magnetic stir bar, purified H_2_O (21 mL), cosolvent (4.0 mL), 500 mM buffer (4.0 mL), and 100 mM KCl or KBr (4.4 mL, 1.1 equiv) were added. The starting material (0.40 mmol, 1 equiv) was dissolved in cosolvent (4.0 mL) and added to the reaction mixture. The microcentrifuge tubes containing the CiVCPO (0.00005 mol %, 2.0 mL total volume) and Na_3_VO_4_ (0.1 equiv) were then added to the reaction mixture. A 10% stock of H_2_O_2_ (264 μL, 2 equiv) was then added to the reaction mixture, and the resulting solution was allowed to stir at room temperature for 16h. The reaction mixture was then diluted with EtOAc (40 mL) and transferred to a 125 mL separatory funnel. The organic layer was collected, and the aqueous layer was re-extracted 3 times with additional EtOAc (20 mL). The combined organic layer was then washed with saturated brine (50 mL), dried on Na_2_SO_4_ for 10 min, concentrated under reduced pressure, and further purified via flash chromatography or recrystallization if needed (see Supporting Information for more details).

For detailed information including chromatography, analytical analysis, protein expression and purification, and stock solution preparation, see the Supporting Information.

## Supplementary Material









## Notes

Detailed experimental, supplemental figures, and characterization data. (PDF)
Computational features used for the modeling efforts. (XLSX)
Data used for the modeling efforts. (XLSX)
DFT-optimized structures. (ZIP)
