# Supplementary material for: Development of a Screening Workflow for the Halogenation of Nitrogen Heterocycles with Vanadium-Dependent Haloperoxidases
Source: ACS Bio Med Chem Au. 2026 Oct 12;6(5):733–43. doi: 10.1021/acsbiomedchemau.6c00157 (PMC13692743; doi:10.1021/acsbiomedchemau.6c00157)
Supplement: Supplementary file 1 [file bg6c00157_si_001.pdf]

Supplementary Materials for

Development of a Screening Workflow for the Halogenation of Nitrogen Heterocycles with Vanadium-Dependent Haloperoxidases

Carter U. Brzezinski[1], Sergio A. Solis[1], Hannah S. Slocumb[2], John Bacsa[1], Kyle F. Biegasiewicz[1]

[1] Department of Chemistry, Emory University, Atlanta, Georgia 30322, USA

[2]Department of Chemistry, University of Utah, Salt Lake City, Utah 84112, USA

This PDF file includes:

Materials and Methods
Product Characterizations
Supplementary Text
Figs. S1-S27
Spectral Data
References

Table of Contents

General Experimental Information 4
Synthesis and Characterization of Starting Materials: 6
General Procedure for N_2-selective methylation with Meerwein's salt: 6
General Procedure for Indazole Synthesis: 9
General Procedure for Pyrazole Synthesis: 12
General Procedure For Well-Plate Screening of Heterocycle Halogenation with *Ci*VCPO: 17
Figure S1: Well Plate Layout 17
Figure S2: Reaction Design 17
Figure S3: Reagent preparation 18
Figure S4: Reaction Setup 19
Figure S5: Pre-Incubation of *Ci*VCPO Halogenation Prior to H_2O_2 Addition 19
Figure S6: Well-plate Consistency Study: 20
Figure S7: Well Plate Reproducibility Study 21
General Procedure For Analytical Scale Heterocycle Halogenation with *Ci*VCPO in Vials: 23
Figure S8: *Ci*VCPO Loading Effect on Indazole Chlorination 24
Figure S9: Na_3VO_4 Loading Effect on Indazole Chlorination 24
Figure S10: H_2O_2 Loading Effect on Indazole Chlorination 24
Figure S11: KCl Loading Effect on Indazole Chlorination 25
Figure S12: Other Cosolvents for Indazole Chlorination 25
Figure S13: Cosolvent Loading Effect on Indazole Chlorination 25
Figure S14: SM Concentration Effect on Indazole Chlorination 26
Figure S15: *Ci*VCPO Loading Effect on Indazole Bromination 26
Figure S16: Na_3VO_4 Loading Effect on Indazole Bromination 26
Figure S17: H_2O_2 Loading Effect on Indazole Bromination 27
Figure S18: KBr Loading Effect on Indazole Bromination 27
Figure S19: Additional Cosolvents for Indazole Bromination 27
Figure S20: Cosolvent Loading Effect on Indazole Bromination 28
Figure S21: SM Concentration Effect on Indazole Bromination 28
Figure S22: Validation + Control Reactions for Indazole Halogenation 29
Figure S23: Validation + Control Reactions for High pH Brominations 30

Figure S24: Re-analysis of Indazole Substrate Scope.. 31
Figure S25: Investigation of Chlorination-Mediated Decomposition of Imidazopyridine 58 .. 32
Figure S26: PMI Comparison of *Ci*VCPO Indazole Halogenation Compared to NBS........... 33
Figure S27: Comparison of *Ci*VCPO Yields (LCAP) Compared to NCS / NBS..................... 34
General Procedure For Preparative Scale Heterocycle Halogenation with *Ci*VCPO.................. 35
Characterization Data of Halogenated Products. 36
General Procedure for Preparative Scale Indazole N-Chlorination:... 95
Characterization Data for N-Chlorinated Indazoles 96
General Procedure for Decarboxylative Halogenation. 102
Computational Details. 105
References.. 108
Spectral Data 111

General Experimental Information

General: Unless specified, all reagents and solvents used in this study were purchased from commercial suppliers and used as received (Combi-Blocks, Sigma-Aldrich, Oakwood Chemicals, Fischer Scientific, VWR). All nonaqueous reactions were performed using glassware that was oven-dried and capped with a rubber septum under nitrogen atmosphere using an inlet and outlet needle connected to a mineral oil bubbler. All aqueous reactions were conducted using glassware without flame-drying prior to experimental set up and without nitrogen atmosphere. For experiments requiring dried or degassed solvent, it was obtained from a solvent purification system from Pure Process Technology. Unless otherwise indicated, deionized water (H_2O) was used in any experiments where H_2O is included in the procedure. Ultrapure Milli-Q Water (Milli-Q H_2O) was accessed through a Milli-Q® EQ 7000 Ultrapure Water Purification System. Reagents including BugBuster® (Sigma-Aldrich, 70584-3), 4X Laemmli Buffer (Bio-Rad, 1610747), 2-mercaptoethanol (Sigma-Aldrich, M6250), Precision Plus Protein Dual Color Standards (Bio-Rad, 1610374), IPTG (Sigma-Aldrich, 16758), Kanamycin Sulfate (VWR, 0408-EU), Deoxyribonuclease from bovine pancreas (Sigma Aldrich, DN25) were purchased from indicated vendors. Cole-Parmer® 96 Deep-Well Microplates (76533-132) and 12-channel reservoir plates (76509-436) were purchased from VWR.

Chromatography: Flash chromatography was performed on SiliaFlash® P60 (230-400 mesh, particle size 0.040-0.063 mm) using the listed solvent systems in each procedure. Thin-layer chromatography (TLC) was performed using Uniplate HLF 250 micron F254 precoated glass plates and preparative TLC was performed on Uniplate GF 1000 micron F254 precoated glass plates. For TLC analysis, a short-wave UV lamp was used.

Spectroscopy and HRMS Analysis: ^{1}H- and ^{13}C-NMR were obtained on a Bruker AVIII or Bruker NEO (400 and 101 MHz, respectively). Chemical shifts are reported in ppm (δ) downfield from tetramethylsilane and are internally referenced to the internal deuterated solvent indicated. ^{1}H-NMR data is reported as follows: chemical shift [multiplicity, coupling constant (Hz), number of hydrogens]. Multiplicities are reported as follows: s (singlet), b (broad signal), d (doublet), dd (doublet of doublets), ddd (doublet of doublet of doublets), t (triplet), dt (doublet of triplets), tt (triplet of triplets), q (quartet), dq (doublet of quartets), p (pentet), m (multiplet). Infrared (IR) were acquired on a Thermo-Fisher Nicolet iS50 spectrometer taken neat, and peaks are reported in frequency of absorption (cm-1). High-resolution mass spectra were obtained on a Thermo Finnigan LTQ-FTMS spectrometer using APCI with an orbitrap mass analyzer.

Analytical: Analytical high-performance liquid chromatography (HPLC) was carried out using a Shimadzu LCMS-2020 System with a Kromasil EternityXT-2.5-C18 column (Dimensions: 4.6 x 50mm, Batch/Serial: 0000016627/A, Part No. XH2CLA05). Liquid chromatography area percentage (LCAP) yields were calculated by integration of all observed non solvent-front peaks in the 254nM chromatogram plot.

Protein Expression and Purification: All protein expression and purification of *Ci*VCPO were performed using previously reported methods[1].

Procedure for the Preparation of Wet Lysate Expressing *Ci*VCPO: E. coli cells expressing *Ci*VCPO were pelleted using centrifugation at 3.5 krpm for 20 minutes at 10 °C in a Sorvall ST Plus centrifuge, and resuspended in storage buffer (25 mM pH 6.5 PIPES/H2SO4 buffer, 2.5 mM Ca(NO3)2, 25 mM Na2SO4) until adjusted to an of OD600 = 18.5. Cells were lysed using a Qsonica Q500 sonicator with a ½ inch probe on ice in 15 s pulses at 32% amplitude, 30 W in 10 bursts with 55 s between bursts. The lysed cell solution was clarified using centrifugation at 13.0 krpm for 20 minutes at 10 °C to remove cell debris and subsequently transferred into a separate 50 mL conical tube. The clarified cell lysate solution was then aliquoted into 0.5 or 1.0 mL microcentrifuge tubes, which were then flash frozen using liquid nitrogen and stored at -80 °C until further use.

Synthesis and Characterization of Starting Materials:

General Procedure for N_2-selective methylation with Meerwein's salt:

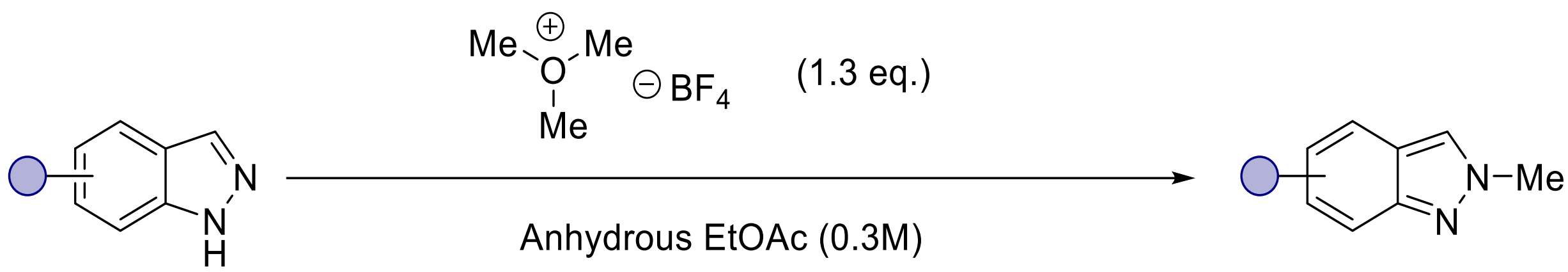

Solvent prep: An oven-dried 50 mL round bottom flask was charged with 10g of oven-dried 4ÅMS, and ~30 mL EtOAc was added. The solvent was left to dry for at least 24 hours under an N_2 atmosphere via balloon.

Warning: OMe_3-BF_4 is highly reactive to water, and thus should be stored in a freezer and only opened inside a fume hood.

Procedure: An oven-dried 20 mL scintillation vial was charged with the corresponding 1H-indazole (2.00 mmol) and dissolved in dry EtOAc (6.67 mL). This reaction mixture was tared on a balance inside a fume hood, then trimethyloxonium tetrafluoroborate (385 mg, 1.3 Eq, 2.60 mmol) was quickly added to the mixture of indazole, and left to stir for 5 hours at room temp. Upon completion, the reaction was diluted with additional EtOAc (5 mL) and $NaHCO_3$ (~5 mL). The organic layer was separated, washed with brine, dried on sodium sulfates, and concentrated in vacuo. The crude material was purified via flash column chromatography (20% EtOAc / Hex).

Note: the methylated indazole's Rf is typically lower than the corresponding SM.

5-fluoro-2-methyl-2H-indazole (SM-4/5)

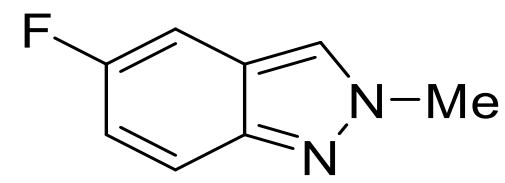

Purification Method: 0 --> 40% EtOAc / Hexanes

^{1}H NMR (400 MHz, $CDCl_3$) δ 7.83 (d, *J* = 1.0 Hz, 1H), 7.65 (ddt, *J* = 9.3, 4.6, 0.9 Hz, 1H), 7.20 (ddd, *J* = 9.1, 2.5, 0.7 Hz, 1H), 7.07 (td, *J* = 9.3, 2.5 Hz, 1H), 4.19 (s, 3H).

^{13}C NMR (101 MHz, $CDCl_3$) δ 159.62, 157.24, 146.43, 123.79, 123.71, 121.47, 121.35, 119.31, 119.21, 117.46, 117.17, 102.72, 102.48, 40.56.

HRMS: calculated for $C_8H_8N_2F$ [M+H]+: 151.0666. Found [M+H]+: 151.0663

5-chloro-2-methyl-2H-indazole (SM-6/7)

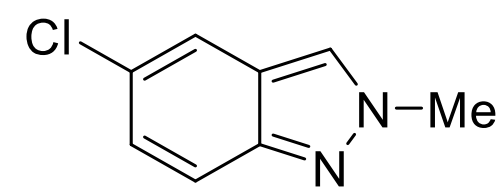

Purification Method: 0 --> 30% EtOAc / Hexanes

^{1}H NMR (400 MHz, $CDCl_3$) δ 7.83 (d, *J* = 0.9 Hz, 1H), 7.65 – 7.54 (m, 2H), 7.20 (dd, *J* = 9.1, 2.0 Hz, 1H), 4.20 (s, 3H).

^{13}C NMR (101 MHz, $CDCl_3$) δ 147.49, 127.43, 127.32, 123.30, 122.61, 118.91, 118.83, 40.60.

NMR data was consistent with literature reported values[2]

General Procedure for Indazole Synthesis:

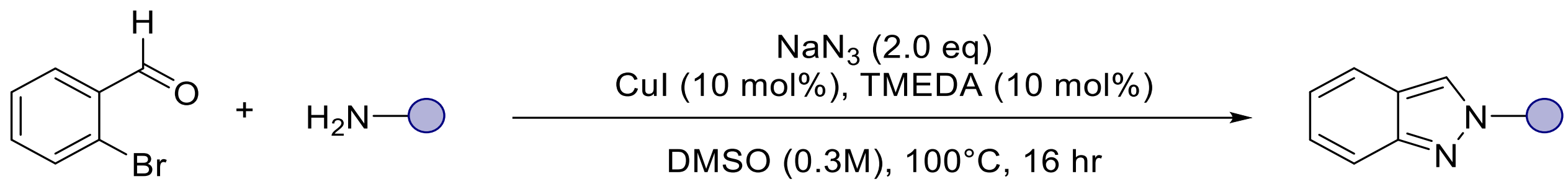

Warning: NaN_3 is shock sensitive, acid sensitive, and this reaction produces N_2 gas. It is recommended to keep this reaction behind a blast shield before starting the reaction and while heating and should never be quenched with acid. It is also highly recommended to extract with EtOAc and completely avoid the use of any halogenated solvents (DCM, $CHCl_3$, etc.)[3].

Procedure: An oven dried 100 mL round bottom flask was charged with 2-bromobenzaldehyde (1.110 g, 700.4 µL, 1.00 Eq, 6.000 mmol) and the corresponding amine (1.20 eq), and dissolved in DMSO (20.00 mL). The flask was sparged via an Ar baloon for 20 minutes, then sodium azide (780.1 mg, 2.00 Eq, 12.00 mmol) was added, followed by copper(I) iodide (114.3 mg, 0.10 Eq, 600.0 µmol) and tetramethylenediamine (69.73 mg, 90.0 µL, 0.10 Eq, 600.0 µmol). The reaction was sealed with a rubber septum and kept under a positive pressure of Ar via balloon, and heated to 100°C for 18 hours. Upon completion, the reaction was diluted with 50 mL of EtOAc and quenched with 50 mL of saturated brine and transferred to a 250 mL separatory funnel. The organic layer was washed with 50 mL of brine 3 times, and dried over sodium sulfate. The crude material was concentrated in vacuo, and purified via flash column chromatography (0-->8% EtOAc, 1% increments). Note: complete removal of DMSO is necessary for clean separation of the product from the imine intermediate, two chromatography purifications were typically necessary for high purity.

2-phenyl-2H-indazole (SM-9)

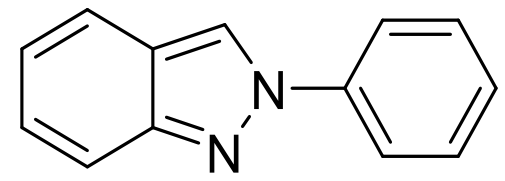

Yield: 43%

Purification Method: 0 --> 10% EtOAc / Hexanes

^{1}H NMR (400 MHz, $CDCl_3$) δ 8.41 (d, *J* = 1.0 Hz, 1H), 7.95 – 7.87 (m, 2H), 7.81 (dq, *J* = 8.8, 1.0 Hz, 1H), 7.72 (dt, *J* = 8.5, 1.1 Hz, 1H), 7.58 – 7.48 (m, 2H), 7.45 – 7.37 (m, 1H), 7.33 (ddd, *J* = 8.8, 6.6, 1.1 Hz, 1H), 7.12 (ddd, *J* = 8.5, 6.6, 0.9 Hz, 1H).

^{13}C NMR (101 MHz, $CDCl_3$) δ 149.94, 140.69, 129.70, 128.04, 126.96, 122.92, 122.60, 121.14, 120.54, 120.52, 118.10.

NMR data was consistent with literature reported values[4].

2-(4-fluorophenyl)-2H-indazole (SM-11)

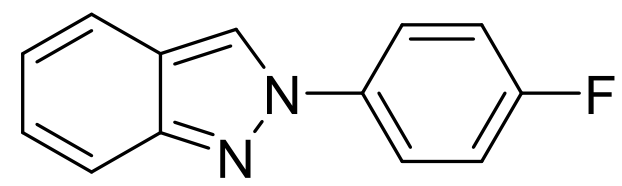

Yield: 51%

Purification Method: 0 --> 10% EtOAc / Hexanes

^{1}H NMR (400 MHz, $CDCl_3$) δ 8.34 (d, J = 1.0 Hz, 1H), 7.92 – 7.74 (m, 3H), 7.70 (dt, J = 8.5, 1.1 Hz, 1H), 7.33 (ddd, J = 8.8, 6.6, 1.1 Hz, 1H), 7.25 – 7.18 (m, 2H), 7.16 – 7.08 (m, 1H).

^{13}C NMR (101 MHz, $CDCl_3$) δ 163.41, 160.95, 149.96, 137.02, 136.99, 127.08, 122.97, 122.94, 122.86, 122.72, 120.62, 120.47, 118.02, 116.70, 116.47.

NMR Data was consistent with literature reported values[5].

General Procedure for Pyrazole Synthesis:

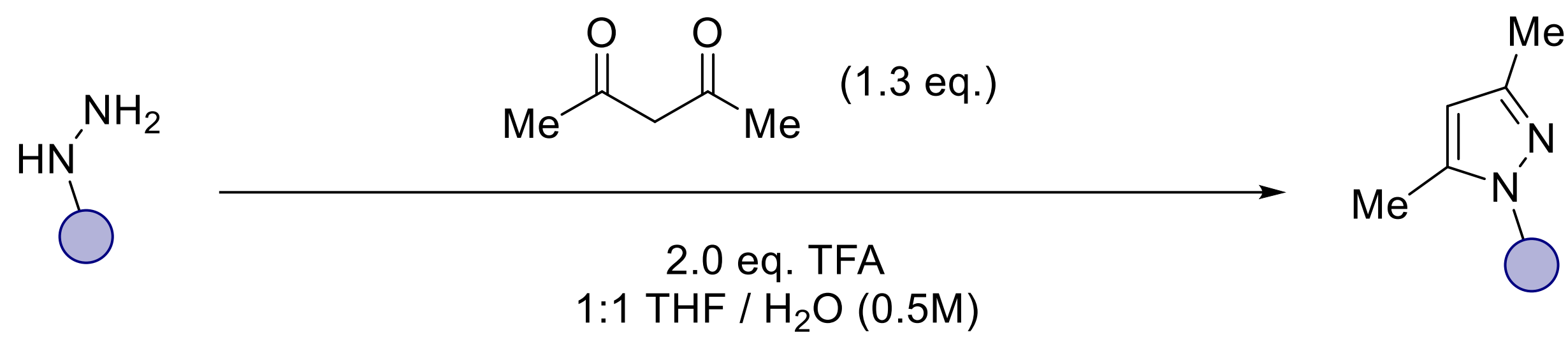

An 20 mL scintillation vial was charged with the corresponding hydrazine (5.0 mmol) and dissolved in THF (5 mL) and water (5 mL). Pentane-2,4-dione (650.8 mg, 667 μL, 1.3 eq, 6.5 mmol) was added followed by trifluoroacetic acid (1.140 g, 770.4 μL, 2.0 Eq, 10.0 mmol), and the reaction was placed in a pre-heated 60°C aluminum heating block. The biphasic reaction was stirred vigorously for 4 hours and monitored by TLC (20% EtOAc / Hexanes). Upon completion, the reaction was quenched with 10 mL EtOAc, and transferred to a 100 mL separatory funnel. The organic layer was separated, and the aqueous layer was reextracted 2 additional times with 10 mL EtOAc. The combined organic layers were dried on sodium sulfate, concentrated in vacuo, and purified via flash column chromatography.

4-(3,5-dimethyl-1H-pyrazol-1-yl)benzonitrile (SM-17/18)

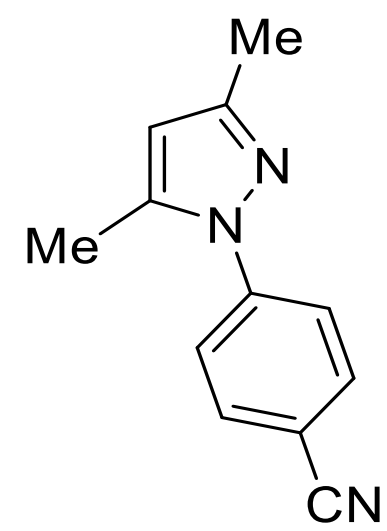

Yield: 85%

Purification Method: 0 --> 30% EtOAc / Hexanes

^{1}H NMR (400 MHz, $CDCl_3$) δ 7.83 – 7.75 (m, 2H), 7.65 – 7.56 (m, 2H), 6.12 (s, 1H), 2.39 (d, *J* = 0.8 Hz, 3H), 2.34 (s, 3H).

^{13}C NMR (101 MHz, $CDCl_3$) δ 150.49, 142.23, 140.57, 133.29, 124.75, 118.14, 111.15, 108.82, 13.04, 12.67.

HRMS: calculated for $C_{12}H_{12}N_3$ [M+H]+: 198.1025. Found [M+H]+: 198.1020

3,5-dimethyl-1-(4-(trifluoromethoxy)phenyl)-1H-pyrazole (SM-19/20)

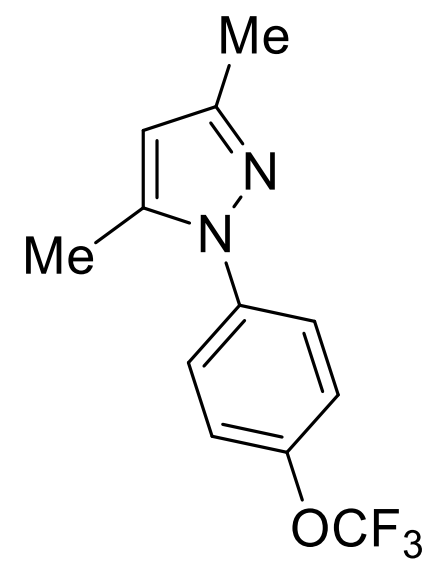

Yield: 80%

Purification Method: 0-->20% EtOAc / Hexanes

^{1}H NMR (400 MHz, $CDCl_3$) δ 7.50 – 7.43 (m, 2H), 7.33 – 7.27 (m, 2H), 6.01 (s, 1H), 2.34 – 2.26 (m, 6H).

^{13}C NMR (101 MHz, $CDCl_3$) δ 149.59, 147.99, 147.97, 139.58, 138.64, 126.04, 121.73, 121.71, 107.53, 13.59, 12.52.

HRMS: calculated for $C_{12}H_{12}ON_2F_3$ [M+H]+: 257.0896. Found [M+H]+: 257.0903

3,5-dimethyl-1-(tetrahydro-2H-pyran-4-yl)-1H-pyrazole (SM-21/22)

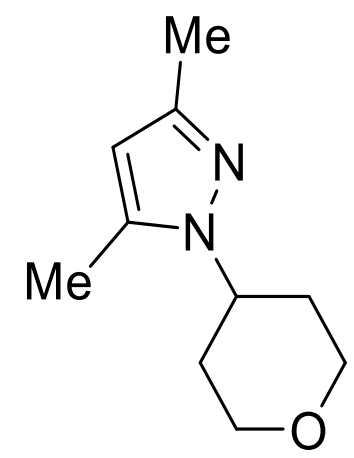

Yield: 71%

Purification Method: 0 --> 75% EtOAc / Hexanes

^{1}H NMR: (400 MHz, $CDCl_3$) δ 5.80 – 5.75 (m, 1H), 4.16 – 4.03 (m, 3H), 3.50 (td, *J* = 12.2, 2.0 Hz, 2H), 2.36 – 2.24 (m, 2H), 2.23 (dd, *J* = 9.7, 0.6 Hz, 6H), 1.83 – 1.73 (m, 2H).

^{13}C NMR: (101 MHz, $CDCl_3$) δ 147.21, 137.77, 105.05, 67.29, 54.40, 32.83, 13.59, 11.04.

HRMS: calculated for $C_{10}H_{17}ON_2$ [M+H]+: 181.1335. Found [M+H]+: 181.1340.

2,5,7-trimethylpyrazolo[1,5-a]pyrimidine (SM-45/46)

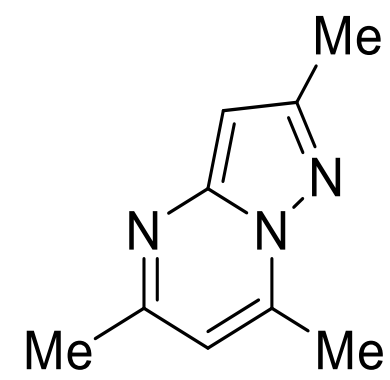

A vial was charged with 3-methyl-1H-pyrazol-5-amine (486 mg, 1 Eq, 5.00 mmol) and dissolved in HCl (5.00 mL). pentane-2,4-dione (551 mg, 565 µL, 1.10 Eq, 5.50 mmol) was added via syringe, and the reaction was capped, sealed with electrical tape, and placed in a pre-heated 100 °C aluminum heating block. The reaction was vigorously stirred for 16 hours, and upon completion quenched with 10 mL of EtOAc and ~50 mL of saturated sodium bicarbonate (**Caution**: effervescence. Add dropwise). The biphasic mixture was transferred to a separatory funnel, and the organic layer was separated, and the aqueous layer was re-extracted 2 additional times with 10 mL EtOAc. The combined organic layers were dried on sodium sulfate, concentrated in vacuo, and purified via flash column chromatography.

Yield: 76%

Purification Method: 0 --> 30% EtOAc / Hexanes

^{1}H NMR (400 MHz, $CDCl_3$) δ 6.45 (q, J = 1.0 Hz, 1H), 6.33 (d, J = 0.6 Hz, 1H), 2.69 (d, J = 0.9 Hz, 3H), 2.55 – 2.47 (m, 6H).

^{13}C NMR (101 MHz, $CDCl_3$) δ 158.00, 154.49, 149.31, 144.63, 107.56, 95.09, 24.54, 17.15, 14.67.

NMR Data was consistent with literature reported values[6]

General Procedure For Well-Plate Screening of Heterocycle Halogenation with *Ci*VCPO:

Figure S1: Well Plate Layout

	Chlorination (1.1 eq KCl)				Bromination (1.1 eq KBr)			
**LC Yield**	DMSO	DMF	MeCN	THF	DMSO	DMF	MeCN	THF
**Acetate 4**								
**Acetate 5**								
**Citrate 5**								
**Citrate 6**								
**MES 6**								
**PIPES 6.5**								
**TRIS 8**								
**CAPS 10**								

Figure S2: Reaction Design

Reaction ID Num	(uM)	(mM)		(mM)	(mM)		(mM)		Total Vol (uL)
Stock Concentration:	10	250		500	100		100	1% stock	400
Reagent:	*Ci*VCPO	Na_3VO_4	H_2O	Buffer	Halide	Co-solvent	Substrate	H_2O_2	
Volume (µL):	20	1.6	189	40	44	40	40	25	
Final concentration (mM)	0.5 µM	1		50	11		10	2.0 eq	

Figure S3: Reagent preparation

Two enzyme aliquots of purified VHPO from *Curvularia inaequalis* (*Ci*VCPO, 10 µM, 1.0 mL) were removed from a -80°C freezer and allowed to warm to room temperature over 5 min. Each thawed aliquot was then charged with an aqueous solution of 250 mM Na_3VO_4 (80 µL per 1.0 mL enzyme aliquot) in a microcentrifuge tube and stored at room temperature for at least 15 minutes.

A 12-column reservoir plate was labeled and filled as follows (from left to right): H_2O, KCl (100mM), KBr (100mM), [empty column], DMSO, DMF, MeCN, THF, [empty column], *Ci*VCPO (10 µM), [empty column], and 1% H_2O_2 (Figure S3-A). **Note: The 1% H_2O_2 solution should only be prepared immediately prior to starting the reactions to prevent light-mediated degradation.** In separate 1-dram vials, prepare ~1.0 mL of 100mM SM in each cosolvent to be tested. Another 12-column reservoir plate was labeled and filled with each 500mM buffer stock solutions (Figure S3-B). **Note: all buffers should be prepared and pH adjusted with H_2SO_4 and NaOH to avoid inclusion of additional halide salts.**

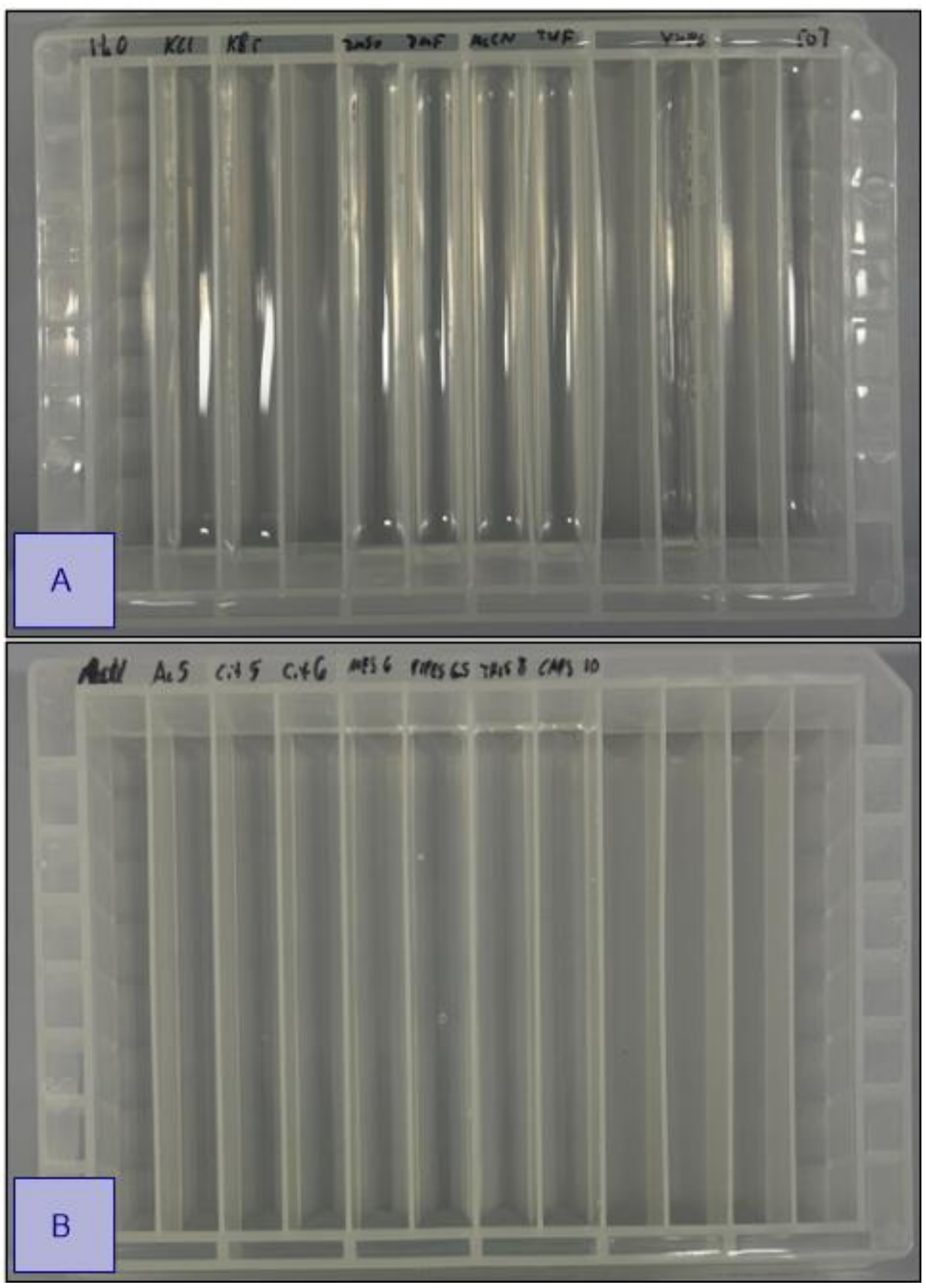

Figure S1: (A) 12-column well plate containing water, 500 mM KCl / KBr, co-solvents, *Ci*VCPO, and H_2O_2. (B) 12-column well plate containing each buffer to be tested.

Figure S4: Reaction Setup

Using a 300µl multichannel pipette, 189 µL of Milli-Q purified H_2O was added to every well. Then 40 µL of each buffer (500mM stock, 50mM final concentration) was added to the corresponding wells, moving from the top to bottom rows (make sure to change pipette tips between buffers). Using a 50µl multichannel pipette, 44 µL of either KCl or KBr was then added to the appropriate wells via multichannel. Next, 40 µL of the appropriate cosolvent was added to each column via multichannel pipette, followed by 40 µL of the appropriate SM stock to each column via micropipette. Finally, 20 µL of the *Ci*VCPO stock was added to every well via multichannel.

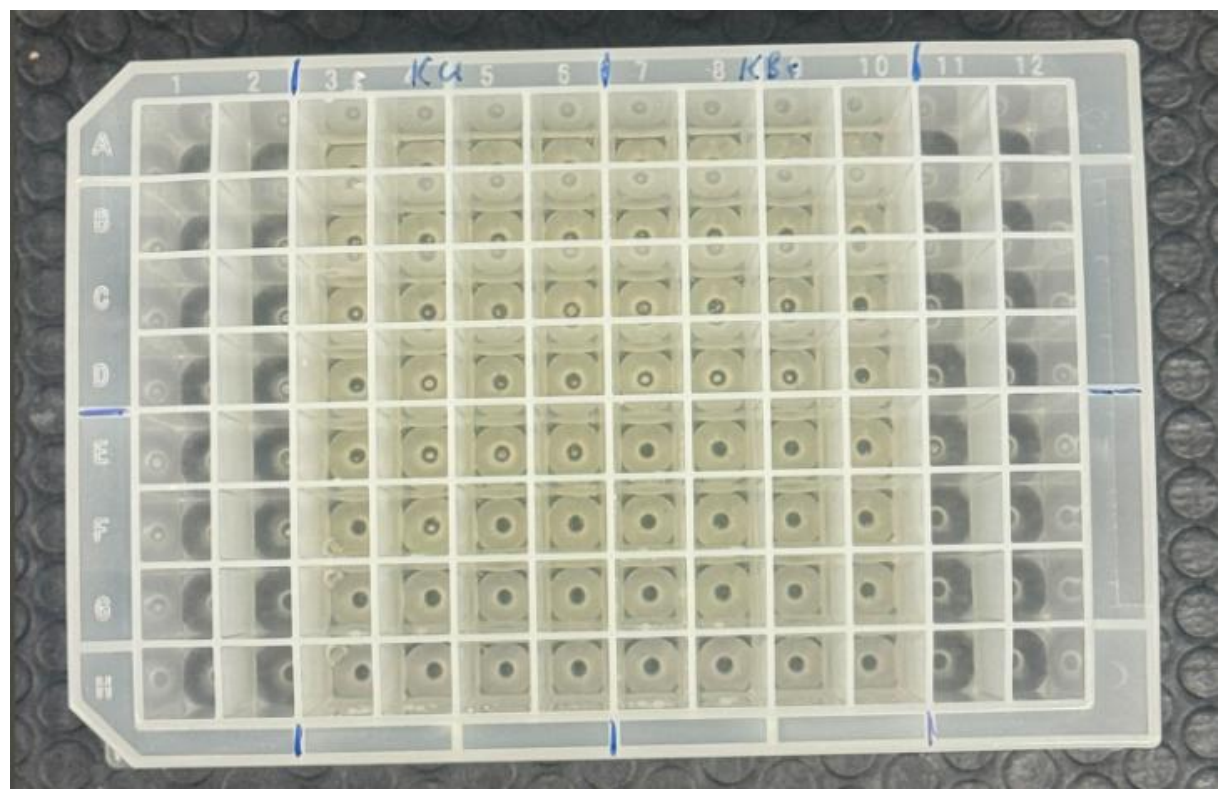

Figure S2: Example reaction setup prior to pre-incubation

Figure S5: Pre-Incubation of *Ci*VCPO Halogenation Prior to H_2O_2 Addition

The reaction plate was then covered with aluminum foil, and shaken at 200RPM on a Heidolph Unimax shaker for 15 min to ensure even distribution of the reaction mixture. The foil was then removed, and 25 µL of 1% H_2O_2 was added (this typically led to a yellow color change). The plate was re-covered with foil and shaken at 200RPM for 16 hours.

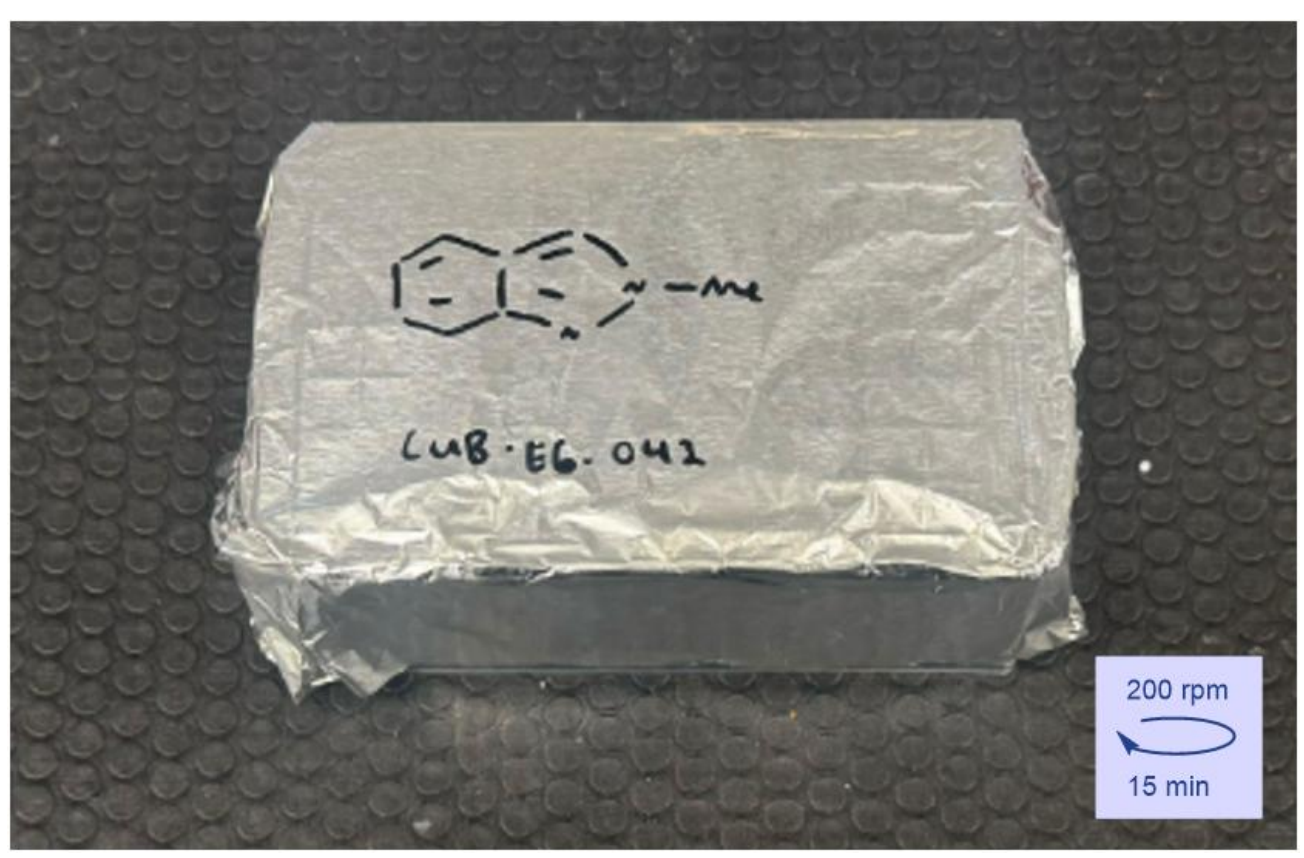

Figure S3: Example HTS plate during pre-incubation before adding H_2O_2

Note: It is recommended to fully submerge the pipette tip and fully eject the solution when adding halide, SM, enzyme, and H_2O_2 *(changing pipette tips between columns) to ensure these components are fully incorporated.*

Workup: Upon completion of the reaction, the aluminum foil was removed and 600 µL of MeCN was added to each well via a 300µl multichannel pipette. The reaction plate was then centrifuged at 1,300 RPM (balanced by another 96 well plate with 1.0 mL of water in each well) in a benchtop centrifuge for 10 minutes. After centrifugation, 600 µL of each reaction was removed for LCMS analysis. Yields were estimated based on liquid-chromatography area percentage (LCAP).

Figure S6: Well-plate Consistency Study:

The following indazole chlorination reaction was used to investigate the consistency of the reaction on a per-well basis, demonstrating the slight improvement with an added 15 min preincubation of the reactions prior to adding H_2O_2.

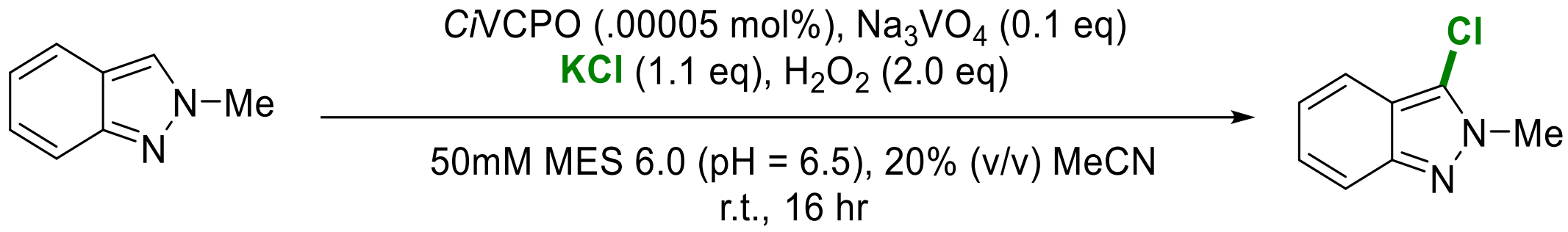

LC Yield	Chlorination MeCN							
MES 6.0	44	49	47	47	49	51	46	49
	43	49	49	0	0	0	46	46
	47	47	50	49	47	49	49	48
	47	49	49	49	49	50	49	49
	47	48	49	49	49	47	48	52
	45	49	48	48	48	49	49	49
	46	50	49	47	49	49	47	48
	45	53	47	47	46	48	47	49

LCMS Yields without 15-min pre-incubation

LC Yield	Chlorination MeCN							
MES 6.0	48	49	49	48	49	47	49	47
	47	48	49	47	48	47	48	47
	47	48	48	48	47	48	47	48
	46	49	47	47	47	44	45	47
	44	46	47	47	46	46	47	47
	45	45	46	45	45	46	47	47
	45	45	45	45	46	49	47	48
	44	46	47	47	48	50	49	49

LCMS Yields with 15-min pre-incubation

	no pre-incubation	15 min pre-incubation
Std. Dev.	1.7	1.1
Average	48.0	47.0
Min	42.7	44.4
Max	52.8	49.5

Figure S6: Reactions to quantify per-well variations in yield and effect of pre-incubation

Figure S7: Well Plate Reproducibility Study

To assess the reproducibility of the described high-throughput screen, we performed three replicates of the model indazole chlorination and bromination screen.

Experiment 1:

	Chlorination				Bromination			
CUB-E6-152A	DMSO	DMF	MeCN	THF	DMSO	DMF	MeCN	THF
Acetate 4	0	31	3	5	0	81	75	87
Acetate 5	0	22	5	7	35	56	54	56
Citrate 5	0	3	4	5	27	34	23	34
Citrate 6	0	0	0	1	17	24	33	21
MES 6	0	43	51	51	35	30	38	26
PIPES 6.5	0	44	50	49	28	28	40	22
TRIS 8	0	0	0	0	2	3	5	3
CAPS 10	0	0	0	0	1	1	1	2

Experiment 2:

	Chlorination				Bromination			
CUB-E6-152B	DMSO	DMF	MeCN	THF	DMSO	DMF	MeCN	THF
Acetate 4	0	34	3	4	0	82	76	87
Acetate 5	0	15	5	5	36	56	54	53
Citrate 5	0	3	2	3	27	32	31	29
Citrate 6	0	0	0	1	19	24	23	18
MES 6	0	42	48	41	35	30	33	24
PIPES 6.5	0	45	49	42	30	28	38	19
TRIS 8	0	0	0	0	2	2	2	3
CAPS 10	0	0	0	0	1	1	1	2

Experiment 3:

	Chlorination				Bromination			
CUB-E6-152C	DMSO	DMF	MeCN	THF	DMSO	DMF	MeCN	THF
Acetate 4	0	33	4	5	0	79	78	84
Acetate 5	0	18	7	6	33	58	58	61
Citrate 5	0	3	2	2	26.4	32	28	39
Citrate 6	0	0	0	0	19	24	29	28
MES 6	0	39	53	45	35	31	41	27
PIPES 6.5	0	43	50	45	30	29	43	23
TRIS 8	0	0	0	0	2	0	2	2
CAPS 10	0	0	0	0	0	0	1	1

Average Across 3 Runs:

	Chlorination				Bromination			
AVERAGE	DMSO	DMF	MeCN	THF	DMSO	DMF	MeCN	THF
Acetate 4	0.0	32.7	3.3	4.7	0.0	80.7	76.3	86.0
Acetate 5	0.0	18.3	5.7	6.0	34.7	56.7	55.3	56.7
Citrate 5	0.0	3.0	2.7	3.3	26.8	32.7	27.3	34.0
Citrate 6	0.0	0.0	0.0	0.7	18.3	24.0	28.3	22.3
MES 6	0.0	41.3	50.7	45.7	35.0	30.3	37.3	25.7
PIPES 6.5	0.0	44.0	49.7	45.3	29.3	28.3	40.3	21.3
TRIS 8	0.0	0.0	0.0	0.0	2.0	1.7	3.0	2.7
CAPS 10	0.0	0.0	0.0	0.0	0.7	0.7	1.0	1.7

Standard Deviation:

	Chlorination				Bromination			
STD DEVIATION	DMSO	DMF	MeCN	THF	DMSO	DMF	MeCN	THF
Acetate 4	0.0	1.5	0.6	0.6	0.0	1.5	1.5	1.7
Acetate 5	0.0	3.5	1.2	1.0	1.5	1.2	2.3	4.0
Citrate 5	0.0	0.0	1.2	1.5	0.3	1.2	4.0	5.0
Citrate 6	0.0	0.0	0.0	0.6	1.2	0.0	5.0	5.1
MES 6	0.0	2.1	2.5	5.0	0.0	0.6	4.0	1.5
PIPES 6.5	0.0	1.0	0.6	3.5	1.2	0.6	2.5	2.1
TRIS 8	0.0	0.0	0.0	0.0	0.0	1.5	1.7	0.6
CAPS 10	0.0	0.0	0.0	0.0	0.6	0.6	0.0	0.6

General Procedure For Analytical Scale Heterocycle Halogenation with *Ci*VCPO in Vials:

An enzyme aliquot of purified VHPO from *Curvularia inaequalis* (*Ci*VCPO, 10 µM, 0.5 mL) was removed from a -80°C freezer and allowed to warm to room temperature over 5 min. The thawed aliquot was then combined with an aqueous solution of 250 mM Na_3VO_4 (40 µL) in a microcentrifuge tube and stored at room temperature. To ensure mixing, the resulting solution was spun in a mini centrifuge for 10 seconds and placed on the benchtop until further use. To a 1-dram vial was added purified H_2O (212 µL), **Cosolvent** (40 µL, see individual entries for details), **500 mM Buffer** (40 µL, see individual entries for details) and 100mM KCl or KBr (44 µL, 1.1 equiv) was added. A 100mM stock of the starting material (0.004 mmol, 1 equiv, 40 µL) was added to the reaction mixture. Each vial was inverted 3x to ensure proper mixing, then 20 µL of the *Ci*VCPO stock was added followed by a 10% stock of H_2O_2 (2.5 µL, 2 equiv) to initiate the reaction. The reactions were shaken either on a Heidolph Unimax shaker or a Heidolph Multi Reax (depending on total number of reactions) for 16h. Upon completion, the reaction mixture was diluted with 650 µL of MeCN, transferred to a 1.5 mL Eppendorf tube, and centrifuged at 13,000 RPM for 5 minutes. 600 µL of the reaction mixture was removed for LCMS analysis.

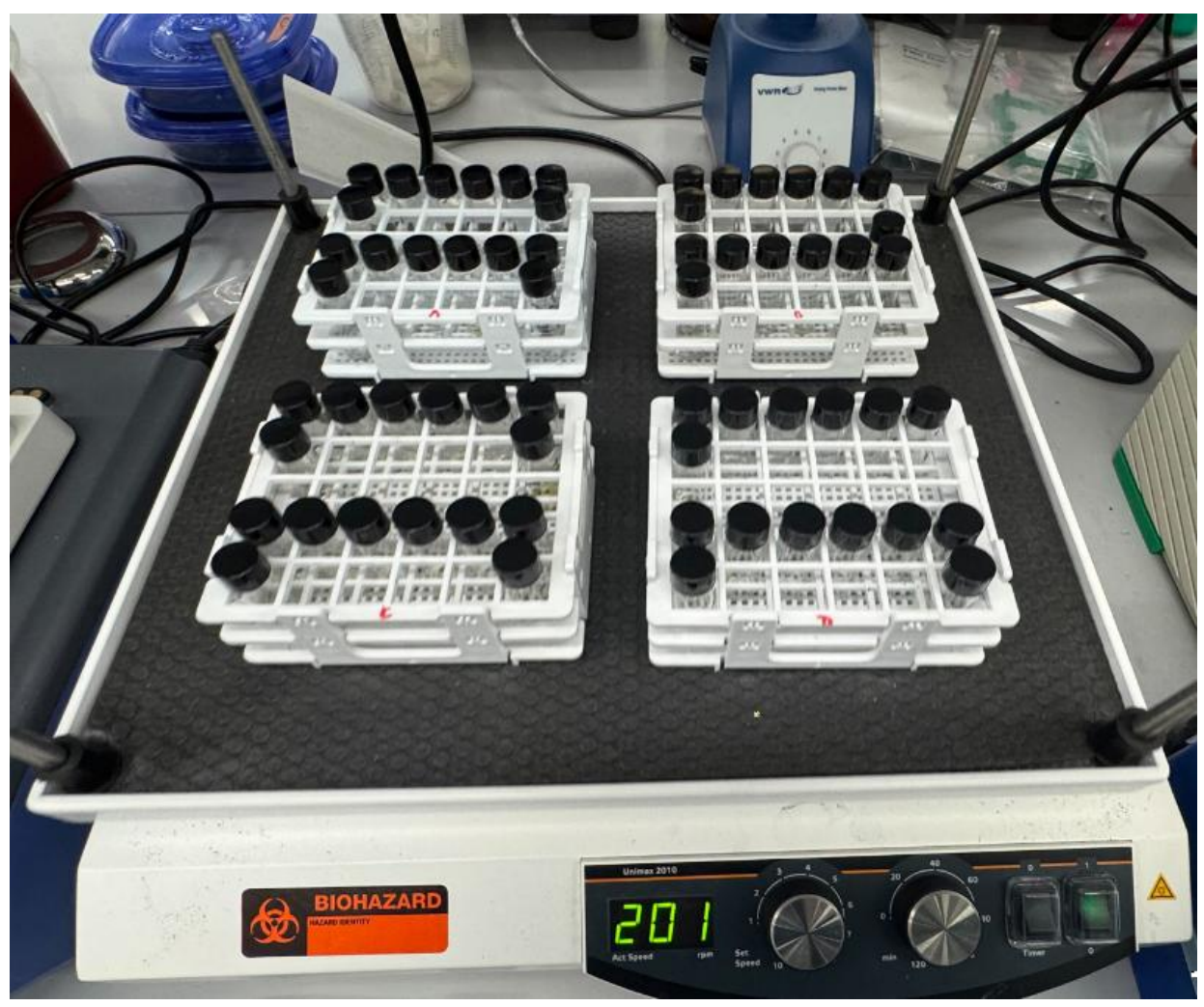

Heidolph Unimax Reaction Setup

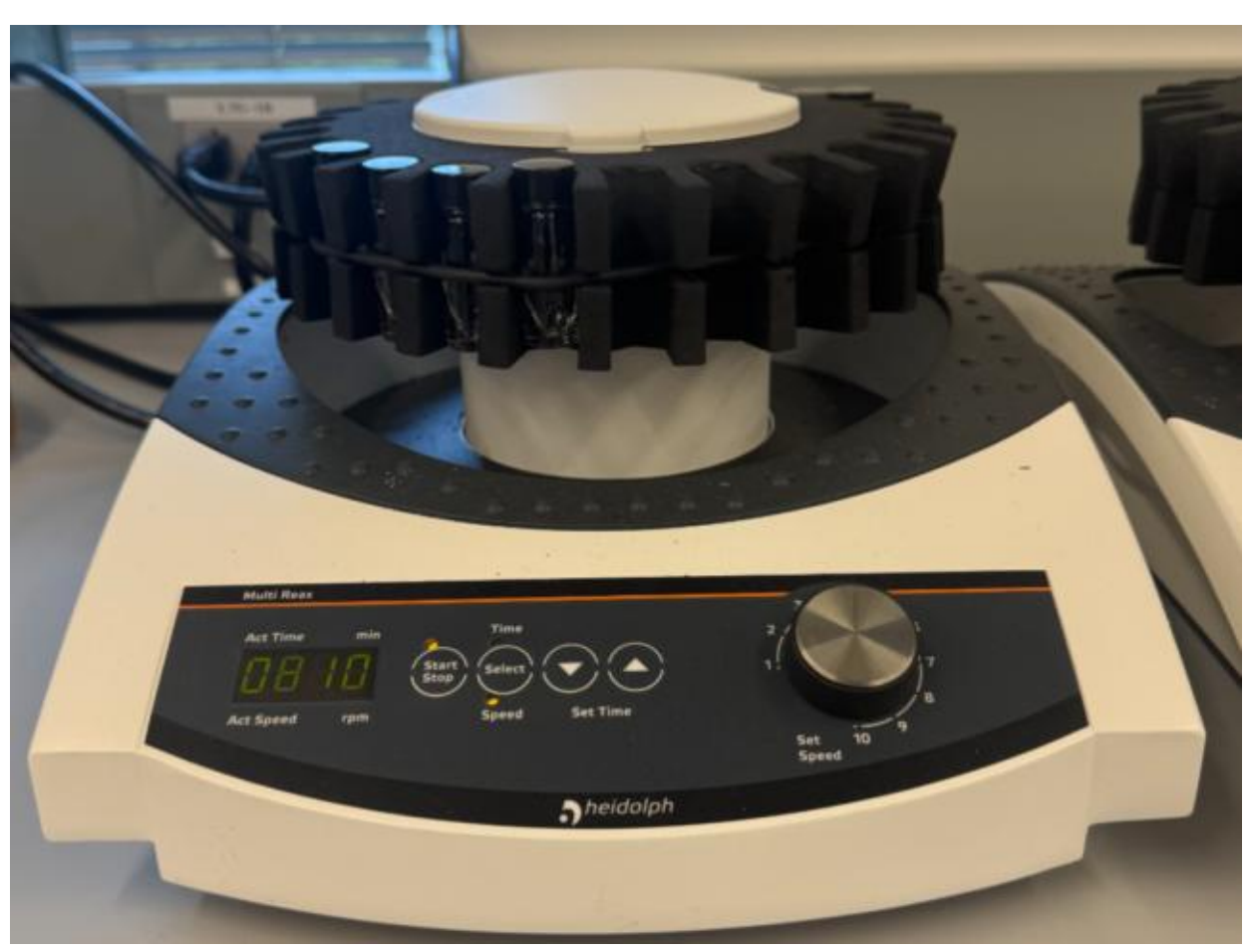

Heidolph Multi Reax Reaction Setup:

Figure S8: *Ci*VCPO Loading Effect on Indazole Chlorination

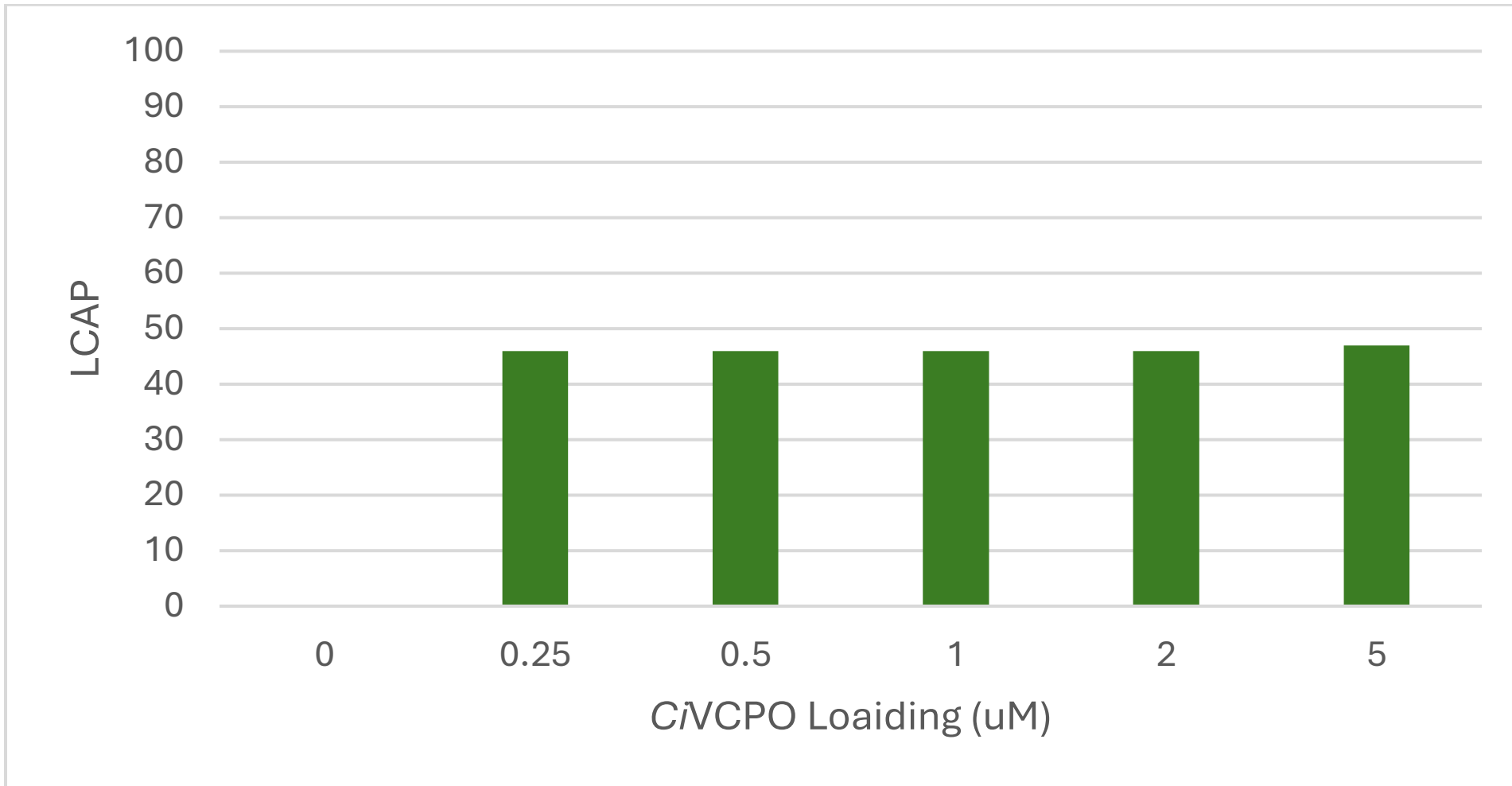

Figure S9: Na_3VO_4 Loading Effect on Indazole Chlorination

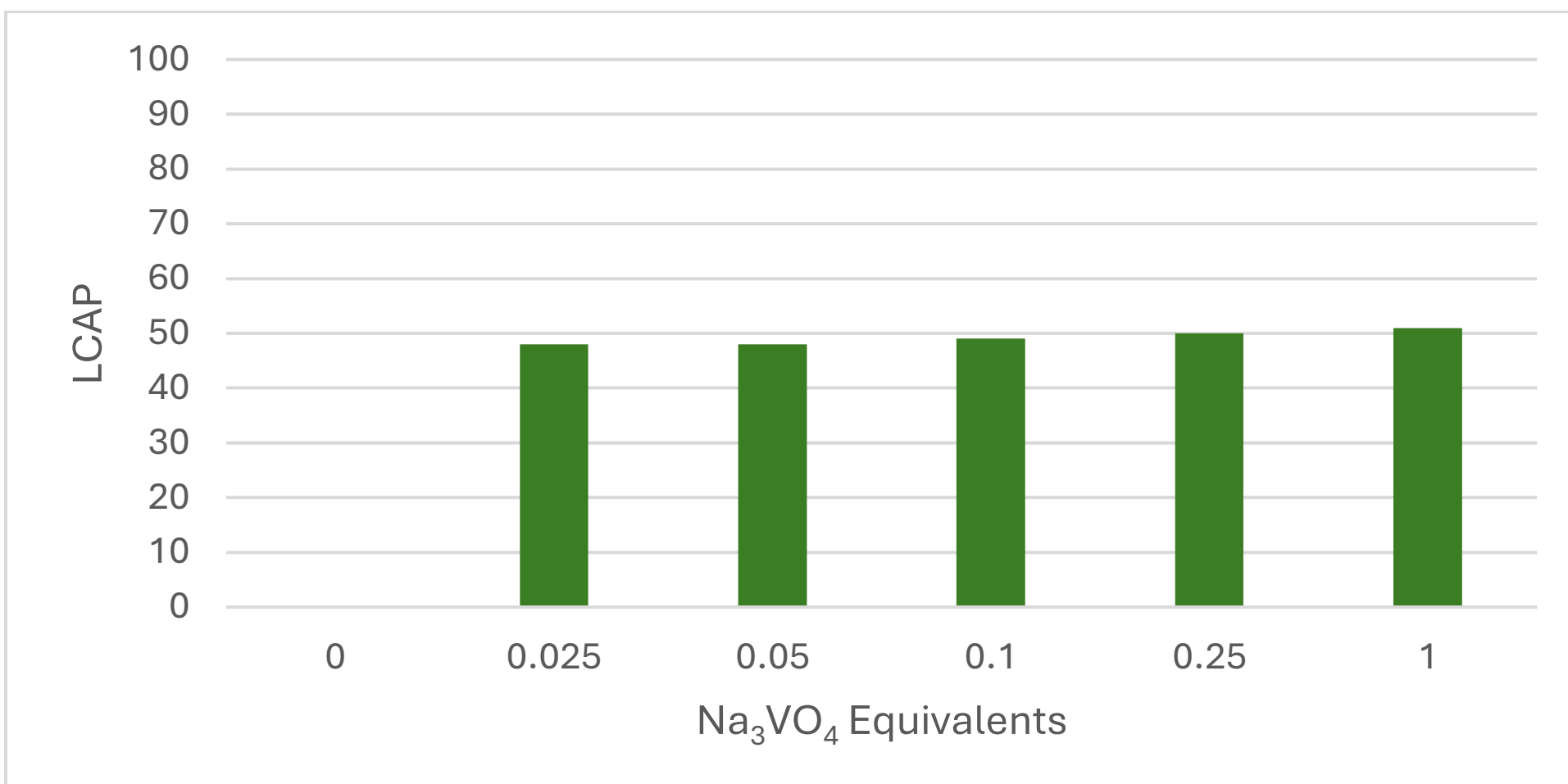

Figure S10: H_2O_2 Loading Effect on Indazole Chlorination

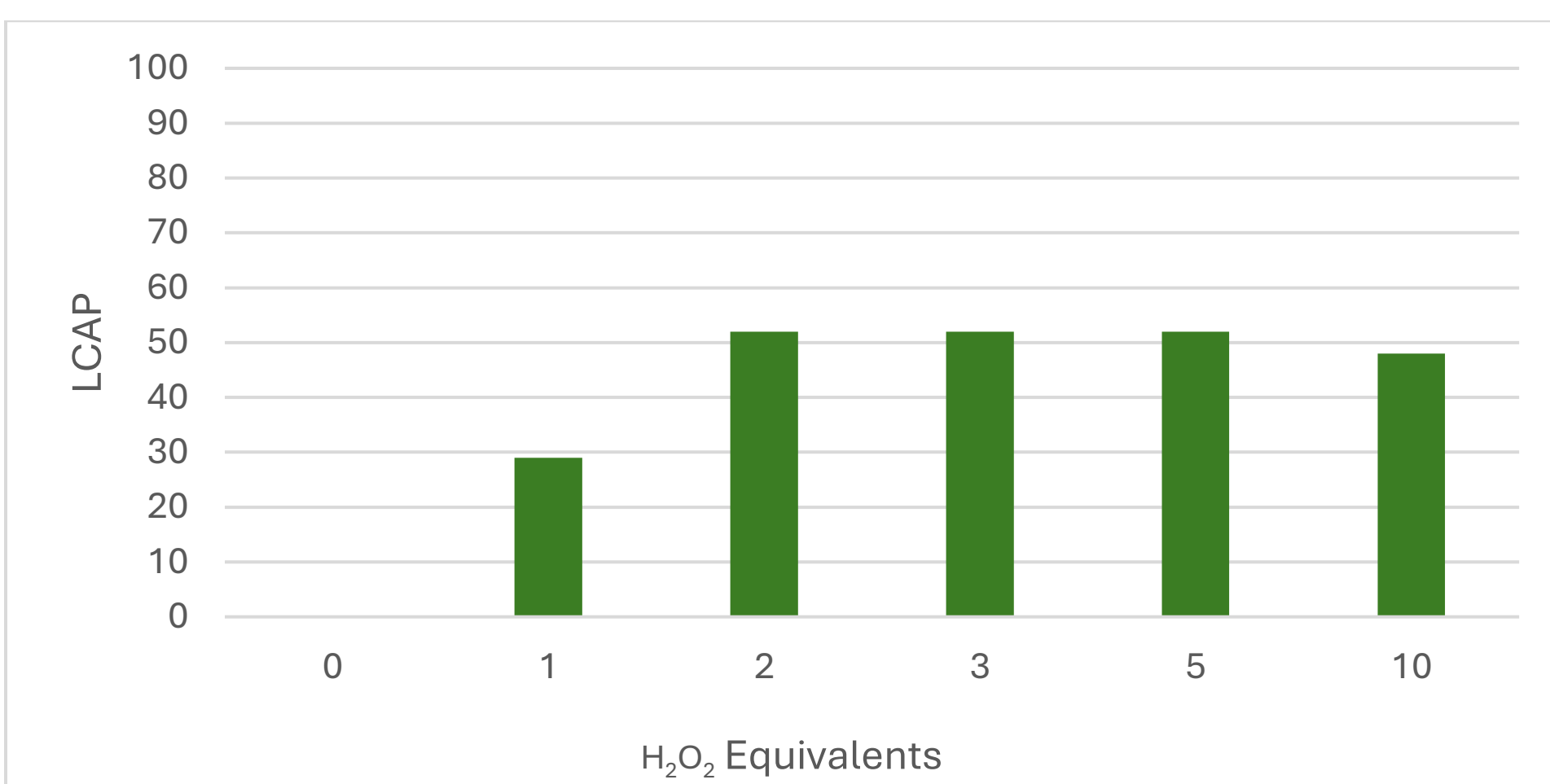

Figure S11: KCl Loading Effect on Indazole Chlorination

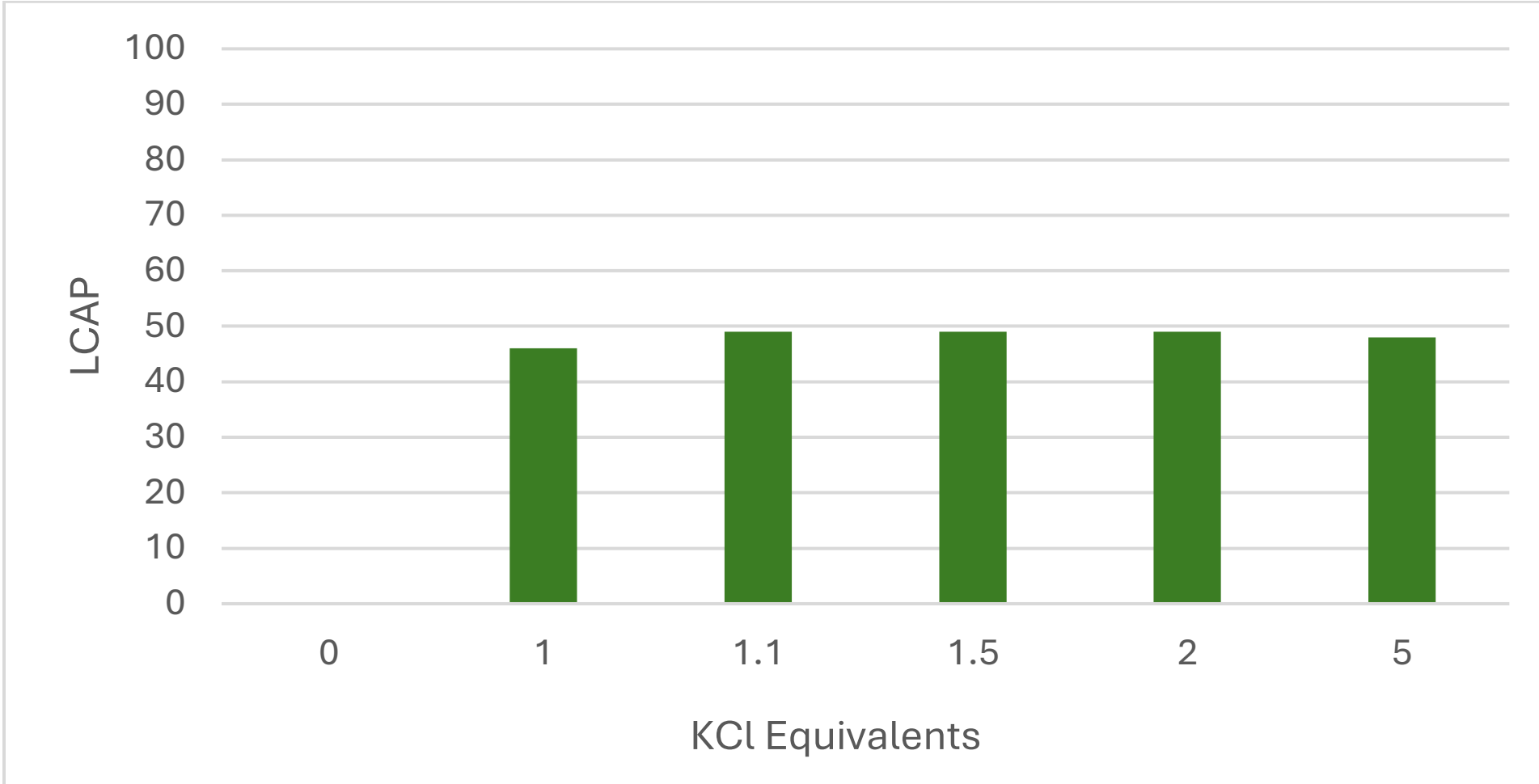

Figure S12: Other Cosolvents for Indazole Chlorination

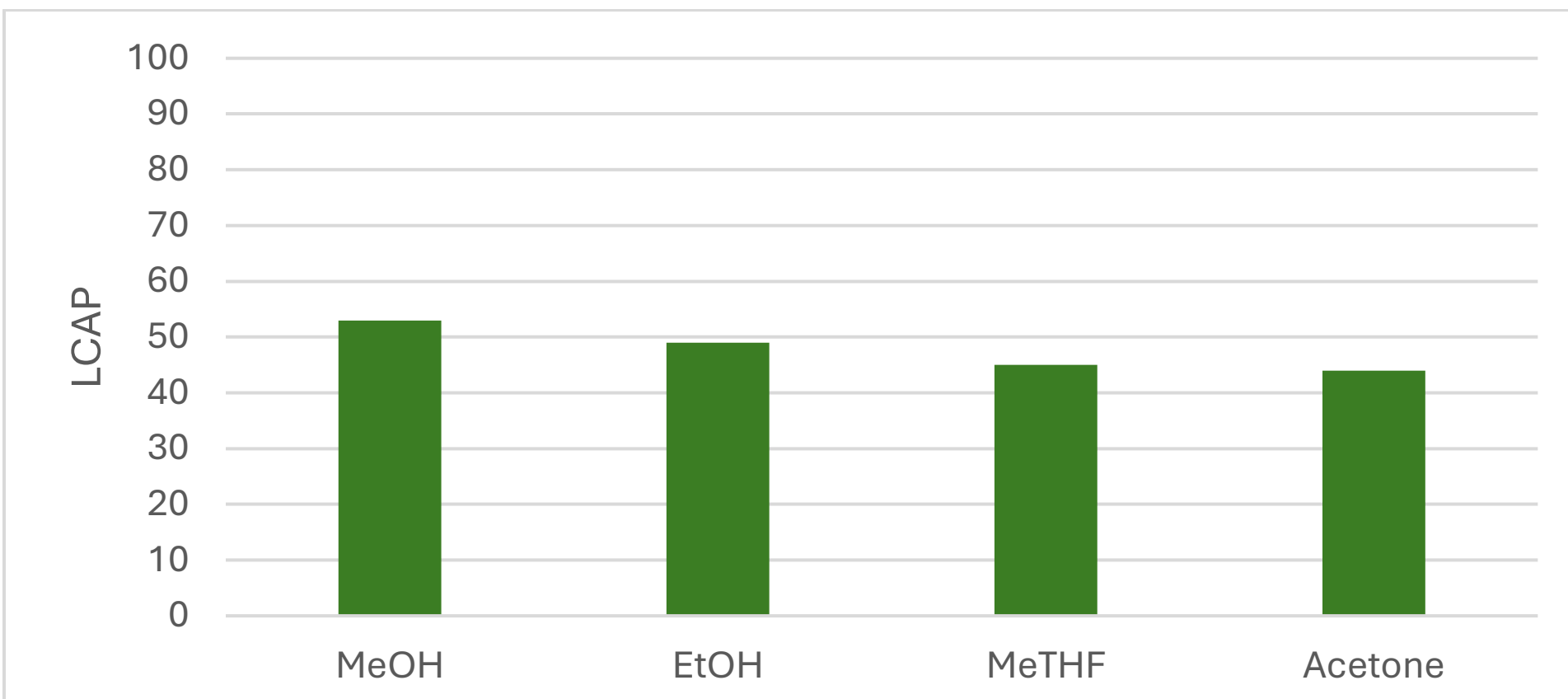

Figure S13: Cosolvent Loading Effect on Indazole Chlorination

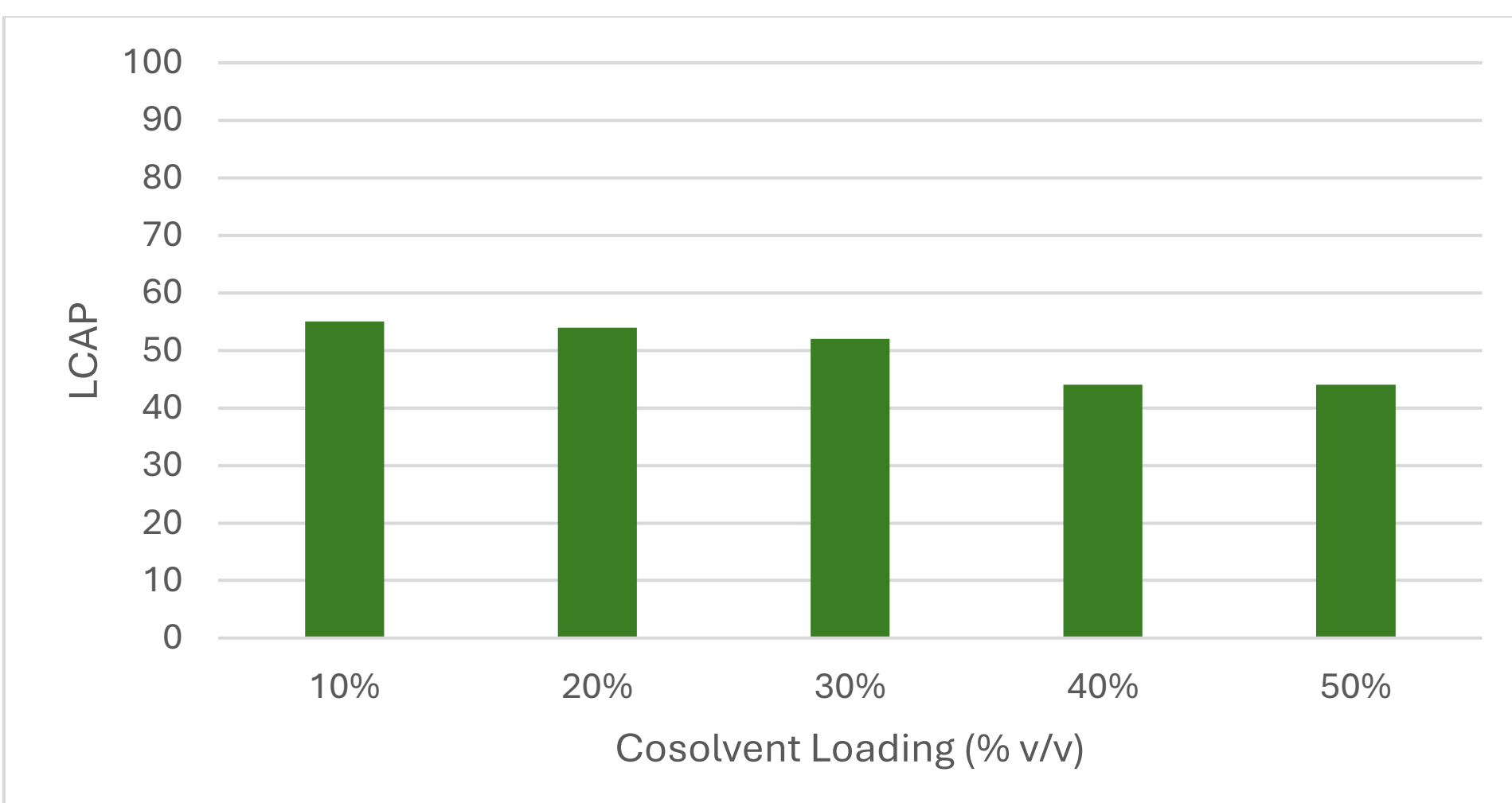

Figure S14: SM Concentration Effect on Indazole Chlorination

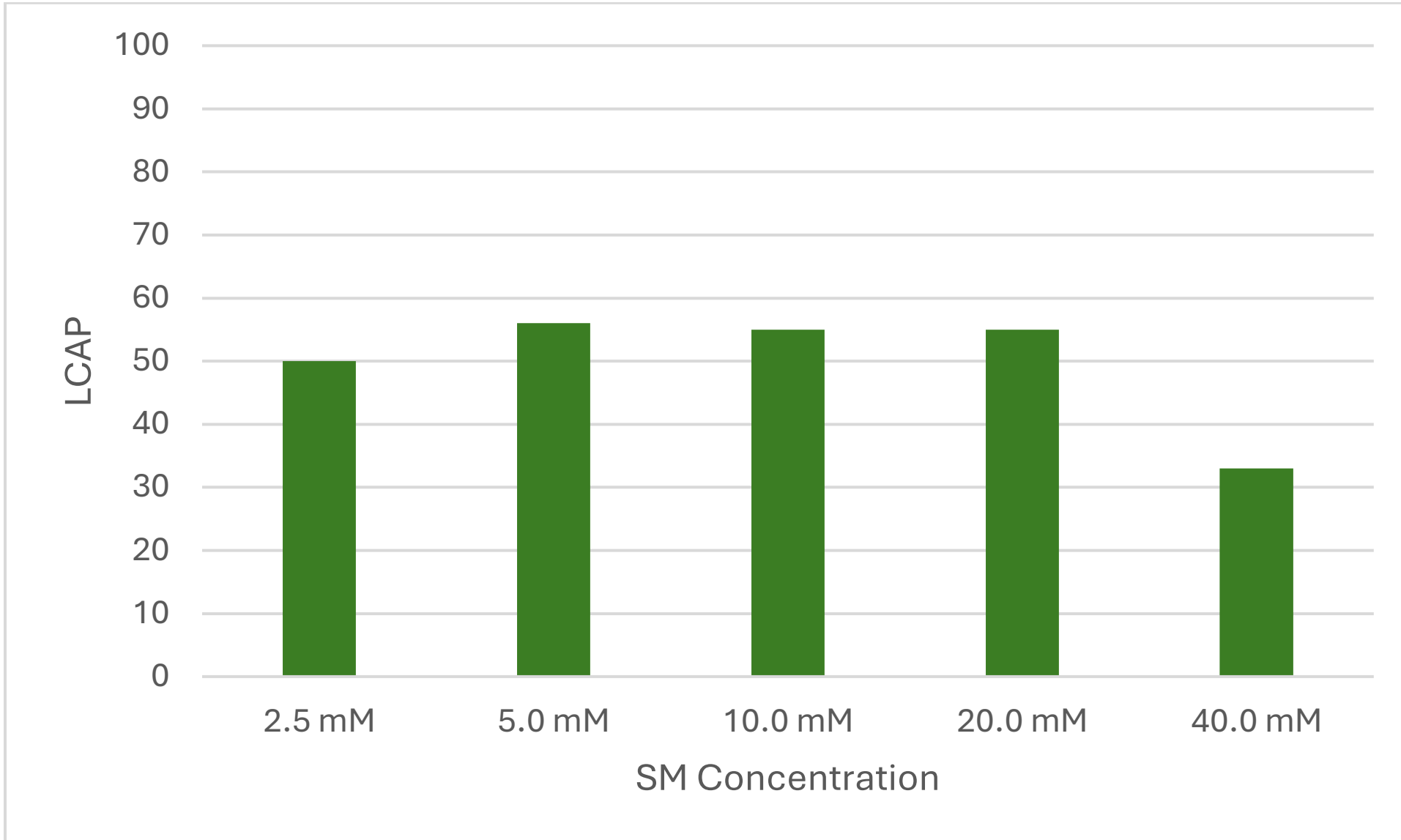

Figure S15: *Ci*VCPO Loading Effect on Indazole Bromination

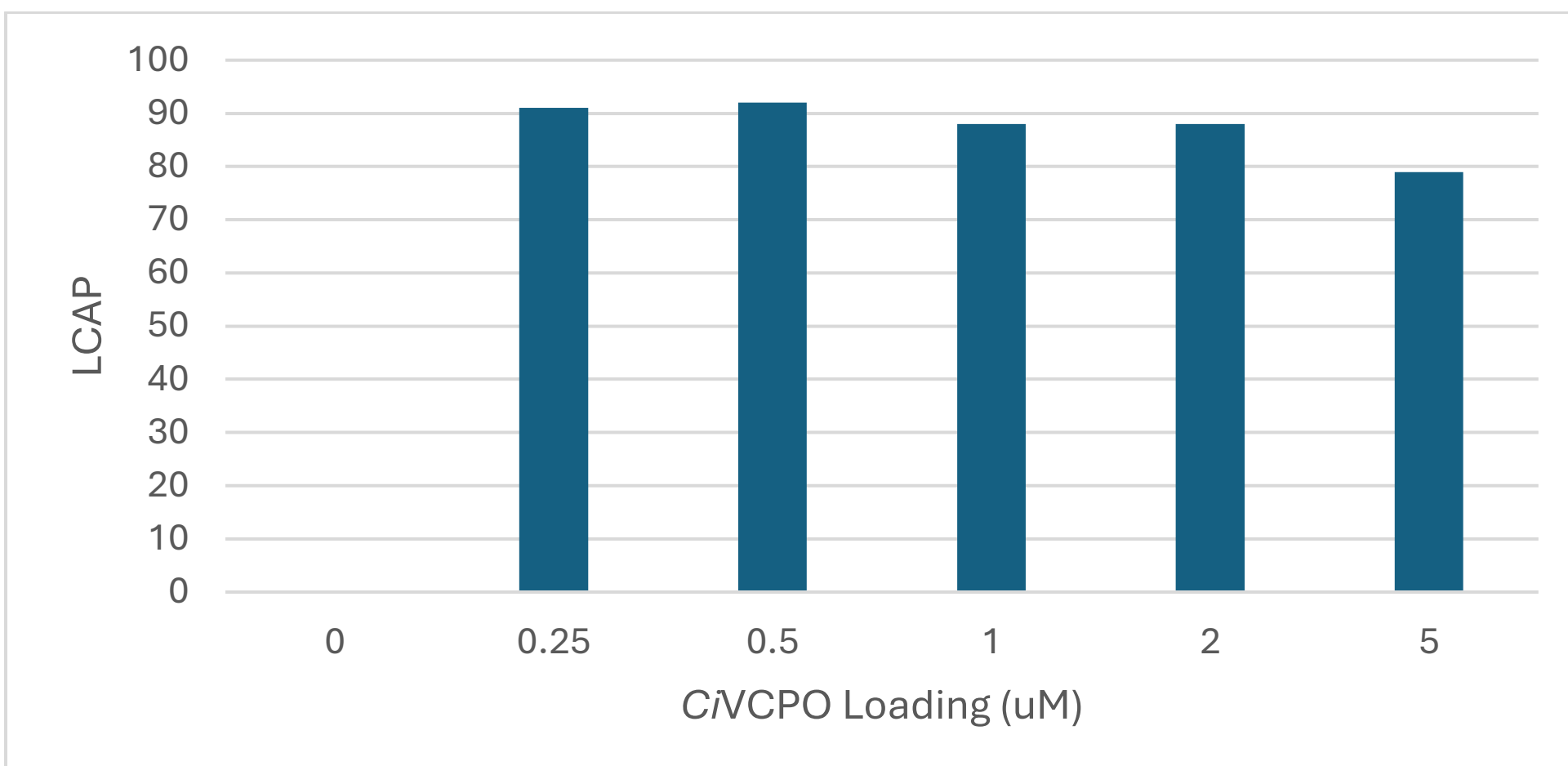

Figure S16: Na_3VO_4 Loading Effect on Indazole Bromination

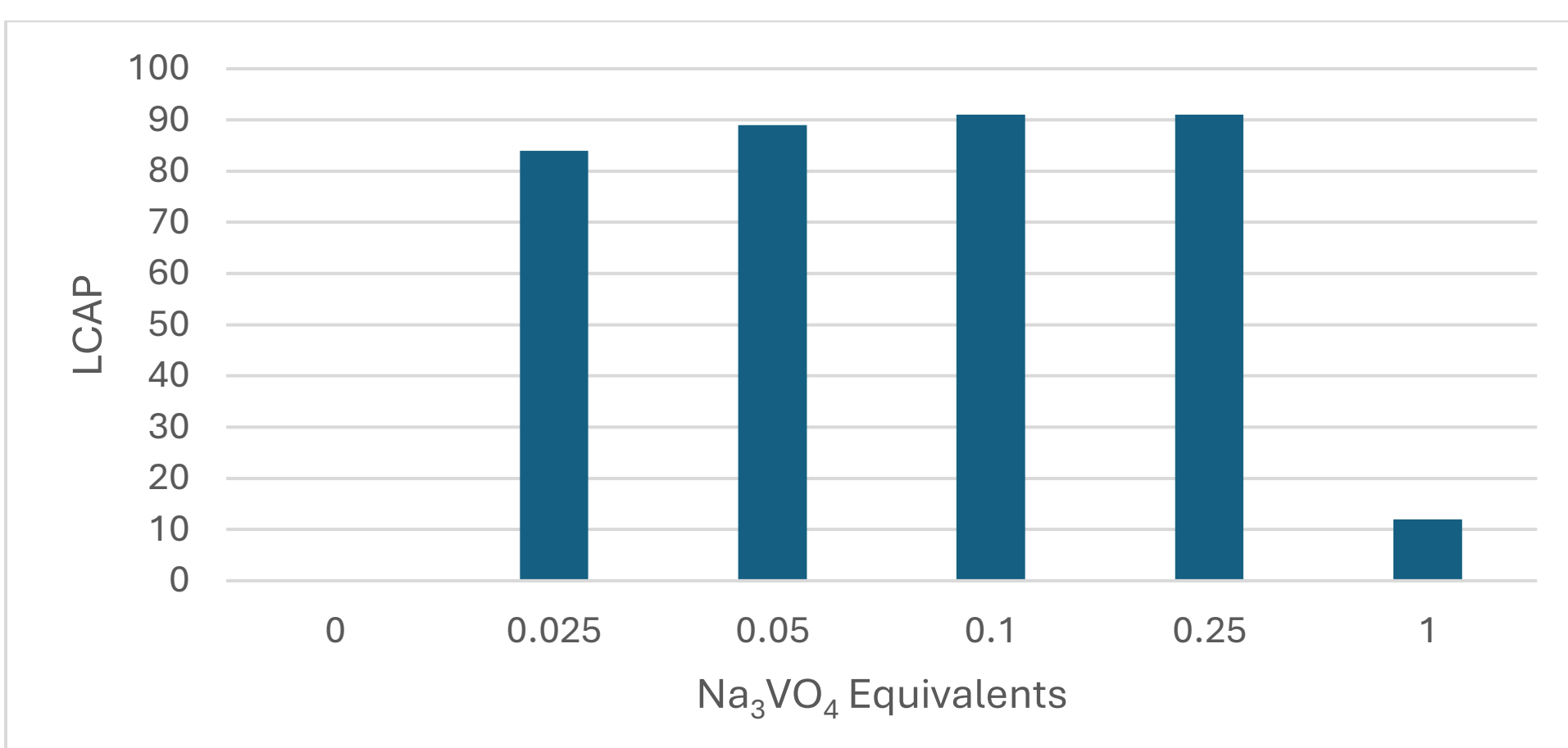

Figure S17: H_2O_2 Loading Effect on Indazole Bromination

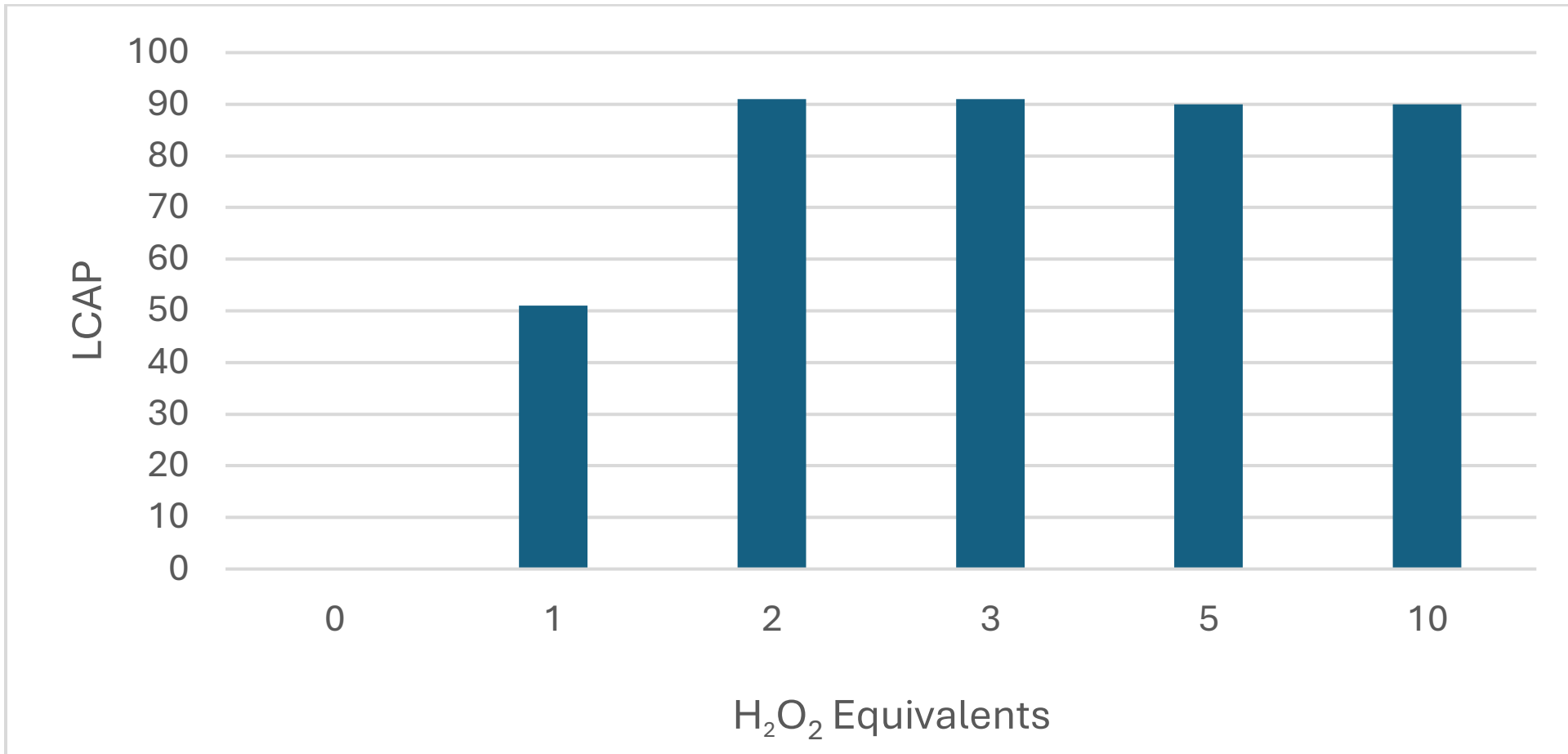

Figure S18: KBr Loading Effect on Indazole Bromination

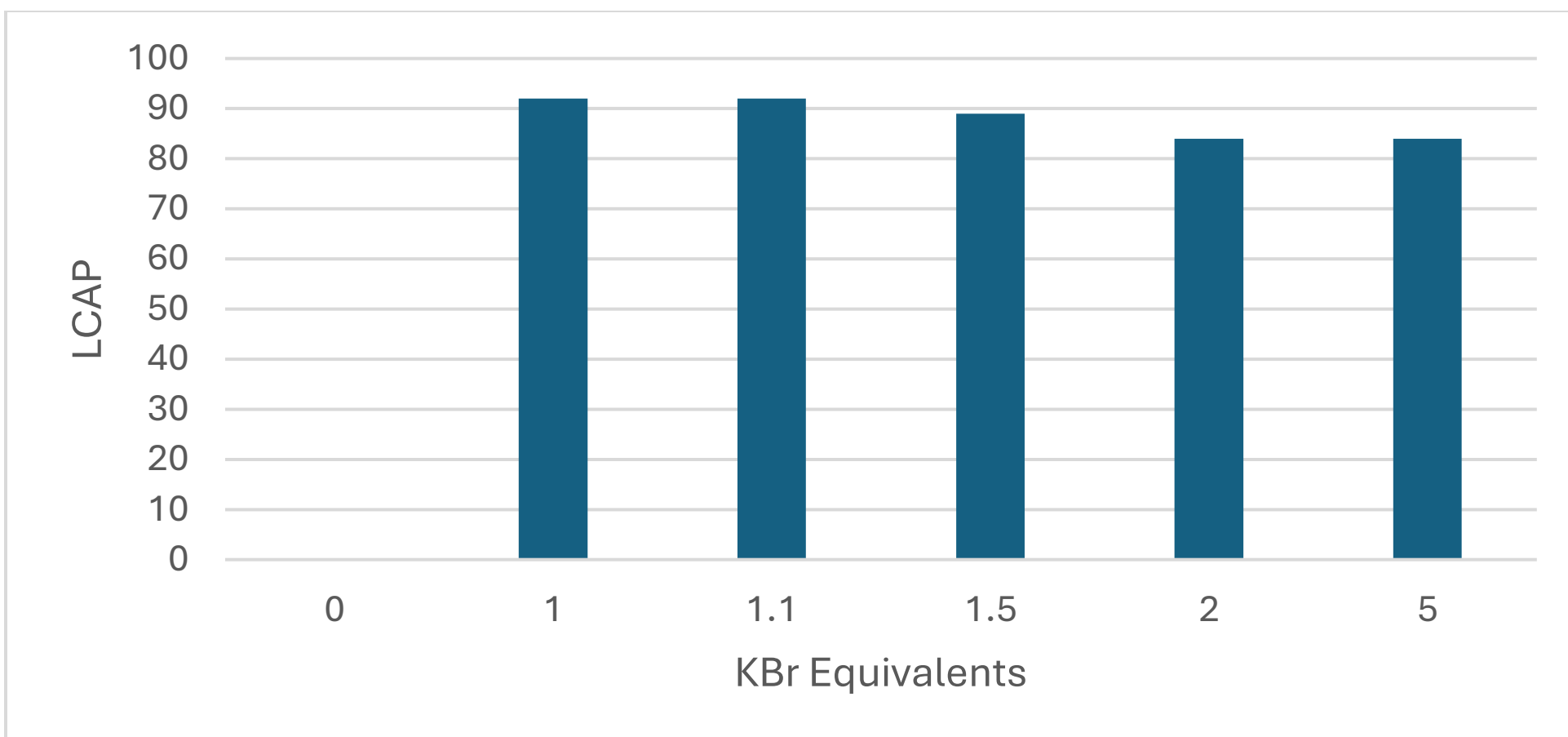

Figure S19: Additional Cosolvents for Indazole Bromination

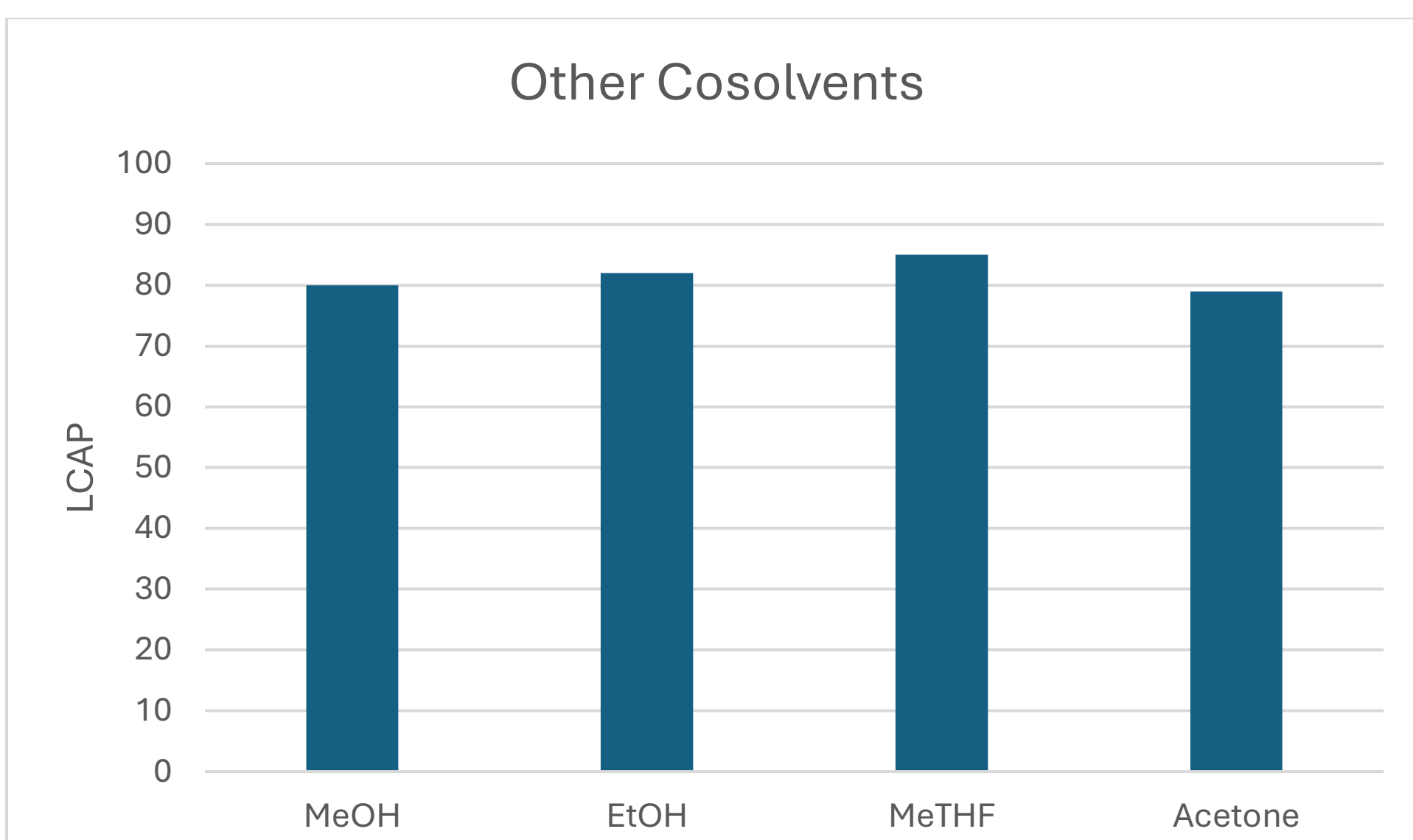

Figure S20: Cosolvent Loading Effect on Indazole Bromination

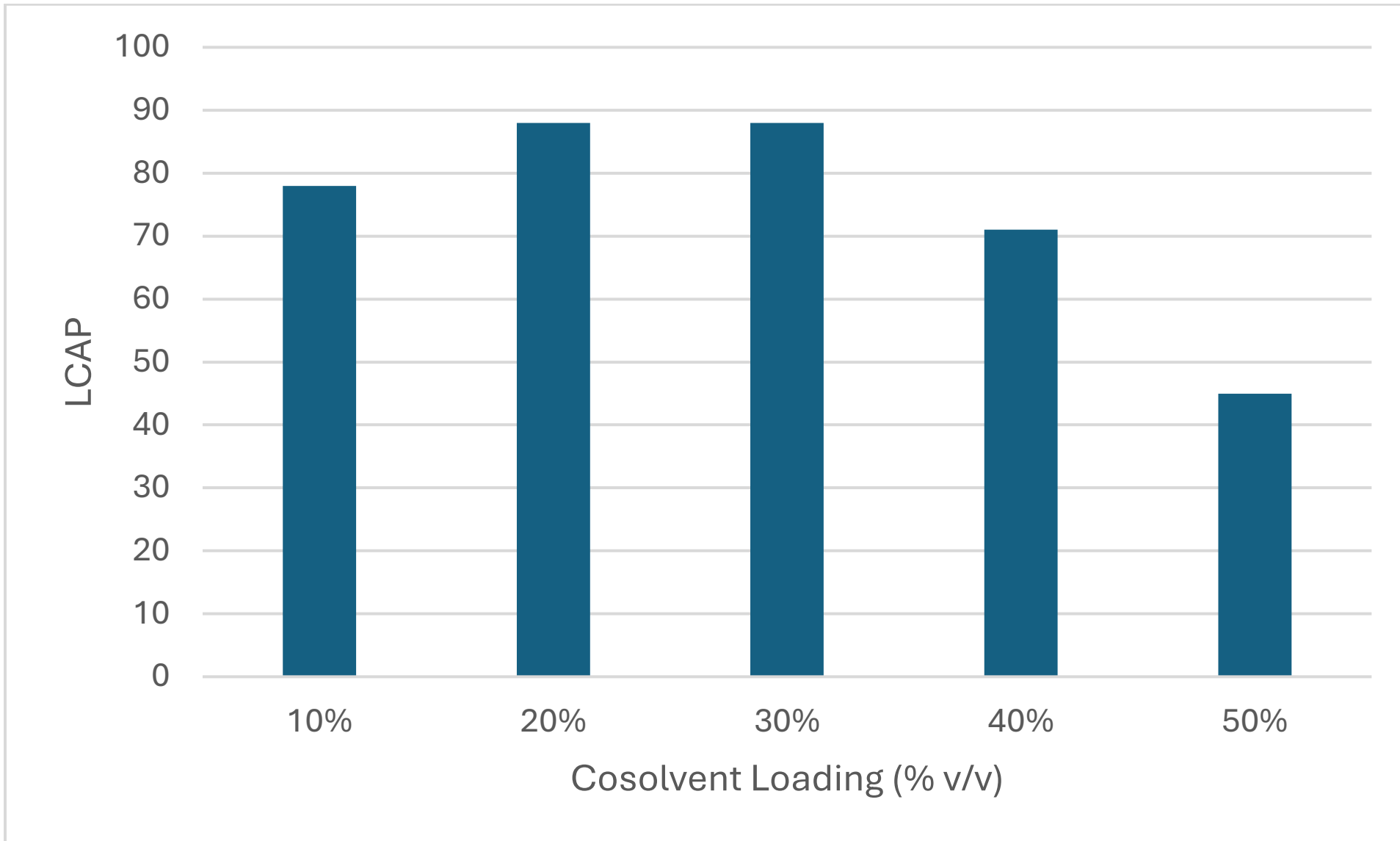

Figure S21: SM Concentration Effect on Indazole Bromination

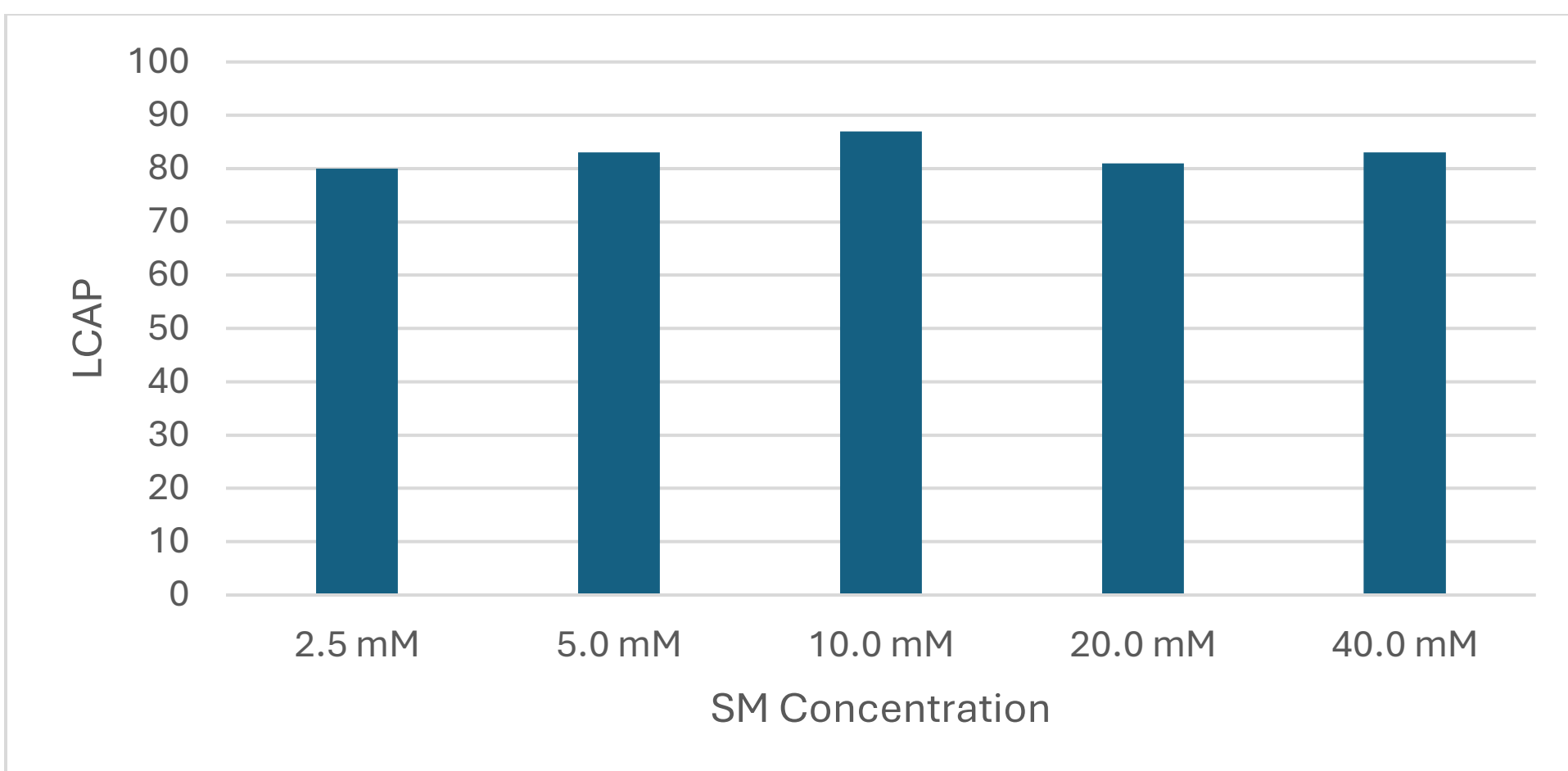

Figure S22: Validation + Control Reactions for Indazole Halogenation

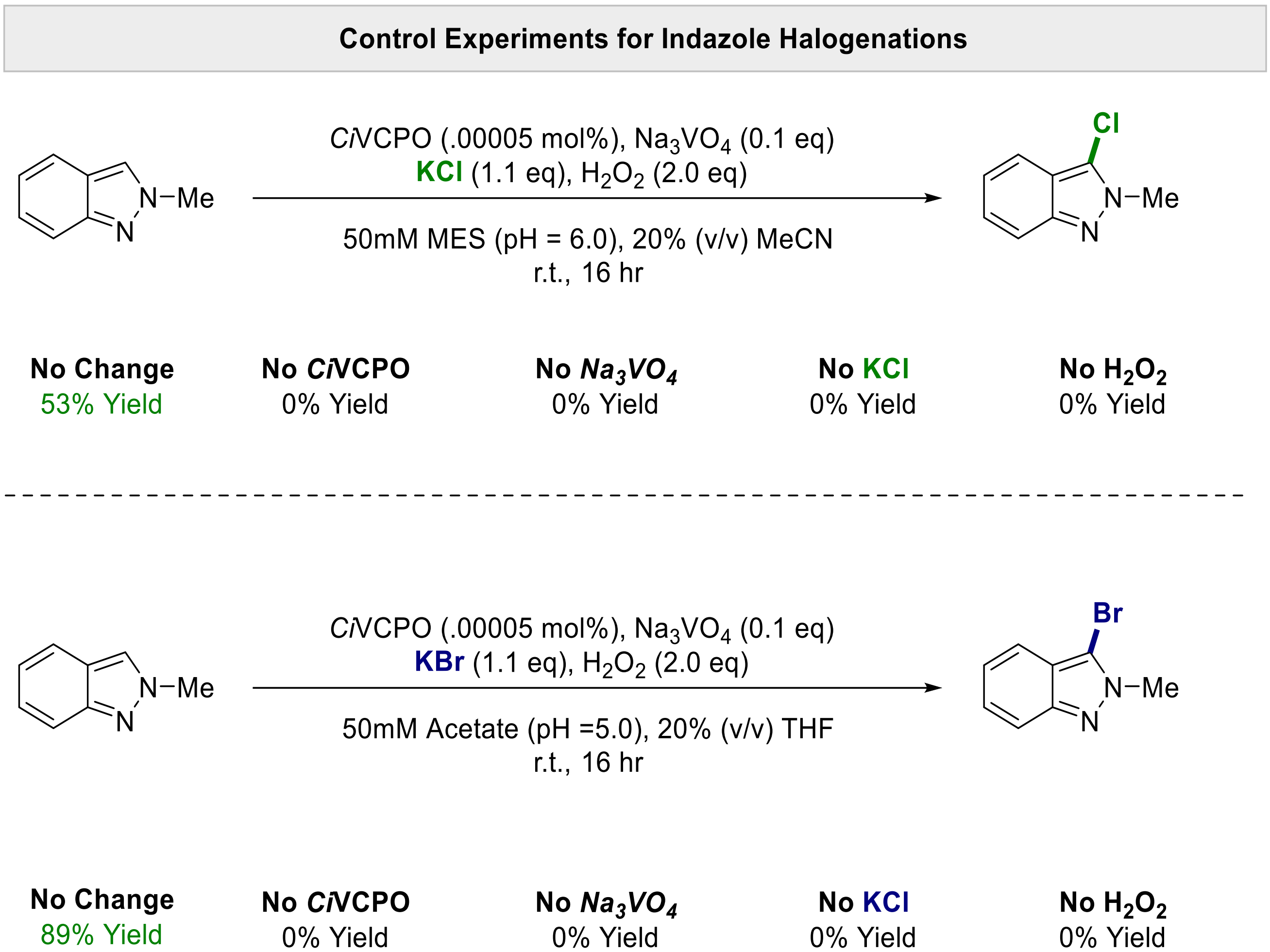

Figure S23: Validation + Control Reactions for High pH Brominations

Control Experiments for High pH Brominations

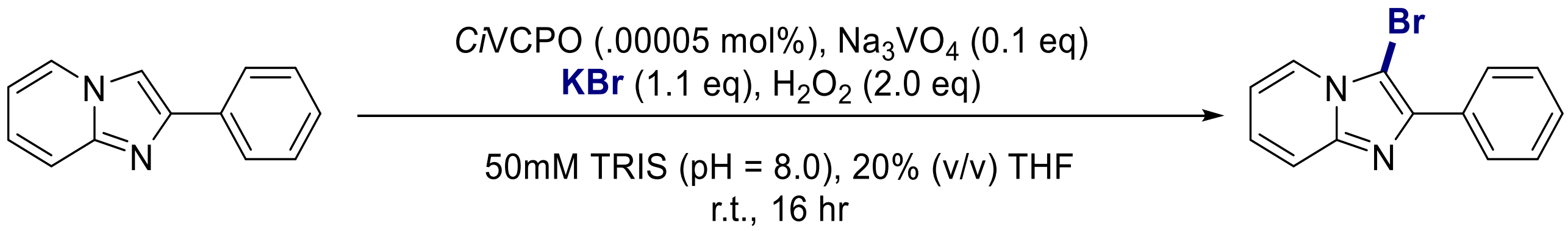

No Change	No *Ci*VCPO	No Na_3VO_4	No KBr	No H_2O_2
98% Yield	0% Yield	0% Yield	0% Yield	0% Yield

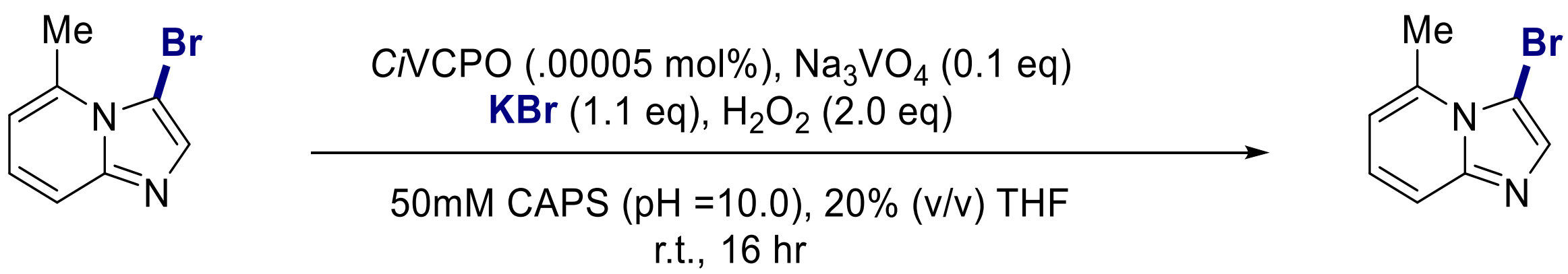

No Change	No *Ci*VCPO	No Na_3VO_4	No KCl	No H_2O_2
86% Yield	0% Yield	0% Yield	0% Yield	0% Yield

No Significant Changes in Reaction pH After Reaction Completion

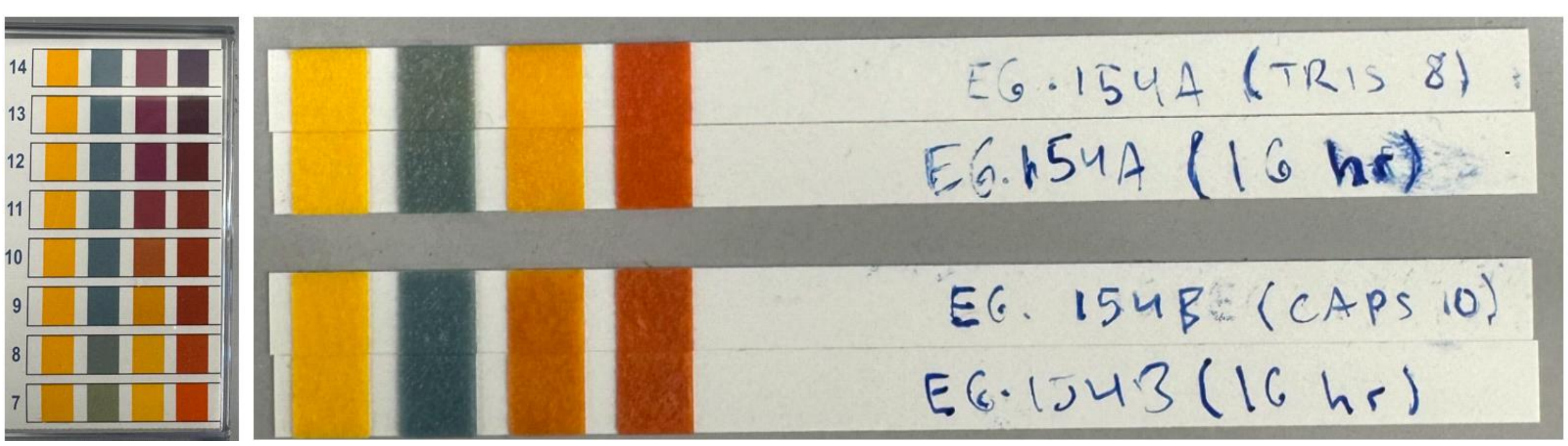

Figure S24: Re-analysis of Indazole Substrate Scope:

To assess the generality of the conditions identified from the high-throughput screen of model indazole **1**, we re-analyzed each member of the indazole substrate scope for optimal buffer conditions for chlorination or bromination. All chlorination reactions were performed in 20% v/v MeCN, and brominations were performed in 20% v/v THF. In each case, 50mM MES (pH = 6.0) and 50mM Acetate (pH = 4.0) were optimal for chlorination and bromination respectively.

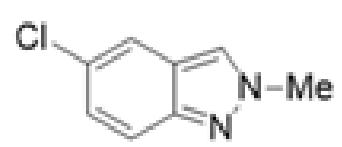 5-chloro-2-methyl-2*H*-indazole Exact Mass: 166.03	Acetate 4	Acetate 5	Citrate 5	Citrate 6	MES 6	PIPES 6.5	TRIS 8	CAPS 10
Chlorination	2	5	4	0	23	17	0	0
Bromination	68	28	29	23	20	14	1	1

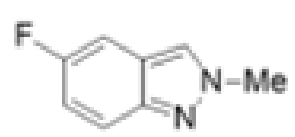 5-fluoro-2-methyl-2*H*-indazole Exact Mass: 150.06	Acetate 4	Acetate 5	Citrate 5	Citrate 6	MES 6	PIPES 6.5	TRIS 8	CAPS 10
Chlorination	5	13	8	0	41	37	0	0
Bromination	99	51	53	32	31	24	6	7

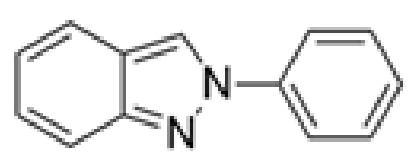 2-phenyl-2*H*-indazole Exact Mass: 194.08	Acetate 4	Acetate 5	Citrate 5	Citrate 6	MES 6	PIPES 6.5	TRIS 8	CAPS 10
Chlorination	6	5	4	0	39	36	0	0
Bromination	84	42	47	32	30	18	0	0

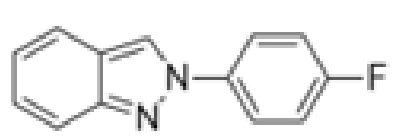 2-(4-fluorophenyl)-2*H*-indazole Exact Mass: 212.07	Acetate 4	Acetate 5	Citrate 5	Citrate 6	MES 6	PIPES 6.5	TRIS 8	CAPS 10
Chlorination	0	0	0	0	10	5	0	0
Bromination	28	13	11	10	9	1	0	0

Figure S25: Investigation of Chlorination-Mediated Decomposition of Imidazopyridine 58

Probing Conversion of Imidazopyridine 58 to Pyridine 60

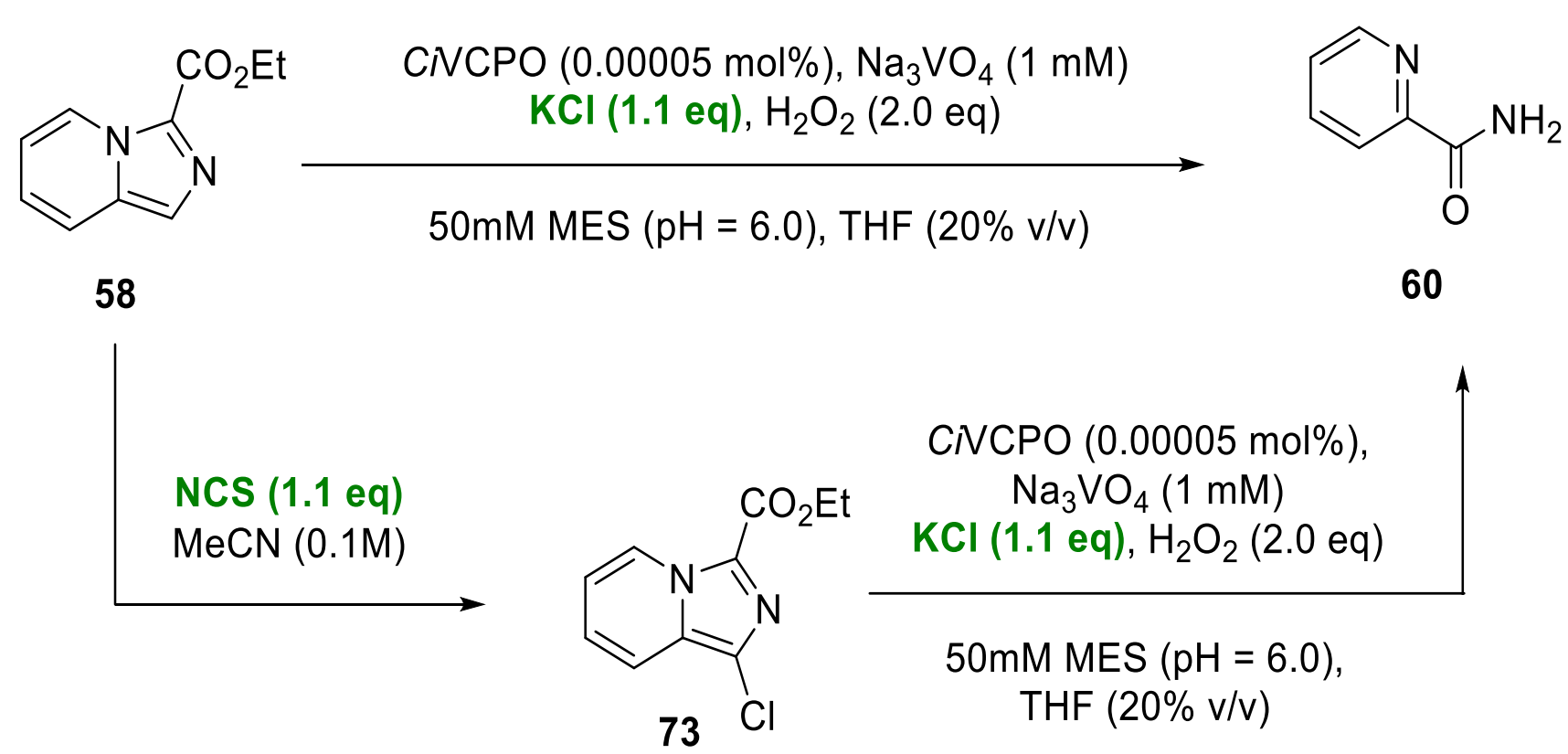

Control Reactions of Chlorination Induced Decomposition of 58

		(Std cond.)	
**0.1 eq KCl**	**0.3 eq KCl**	**1.1 eq KCl**	**2.0 eq KCl**
25% Yield	43% Yield	59% Yield	55% Yield
+ 10% **73**	+ 20% **73**	+ 8% **73**	+ 8% **73**

**No *Ci*VCPO**	**No *Na_3VO_4***	**No KCl**	**No H_2O_2**
0% Yield	0% Yield	*trace* (<3%)	0% Yield

Takeaway: conversion of 58 to 60 is dependent on HOCl, and not just oxidant

Control Reactions of Oxidation Induced Decomposition of 73

**STD Conditions**	**No *Ci*VCPO or Na_3VO_4**	**No *Na_3VO_4***	**No *Ci*VCPO**
54% Yield	0% Yield	0% Yield	0% Yield

**No KCl**	**No H_2O_2**	**Only Buffer, THF, and H_2O**
57% Yield	0% Yield	0% Yield

Takeaway: oxidation of 73 is enzyme catalyzed, and not reliant on HOCl

Potential Mechanism

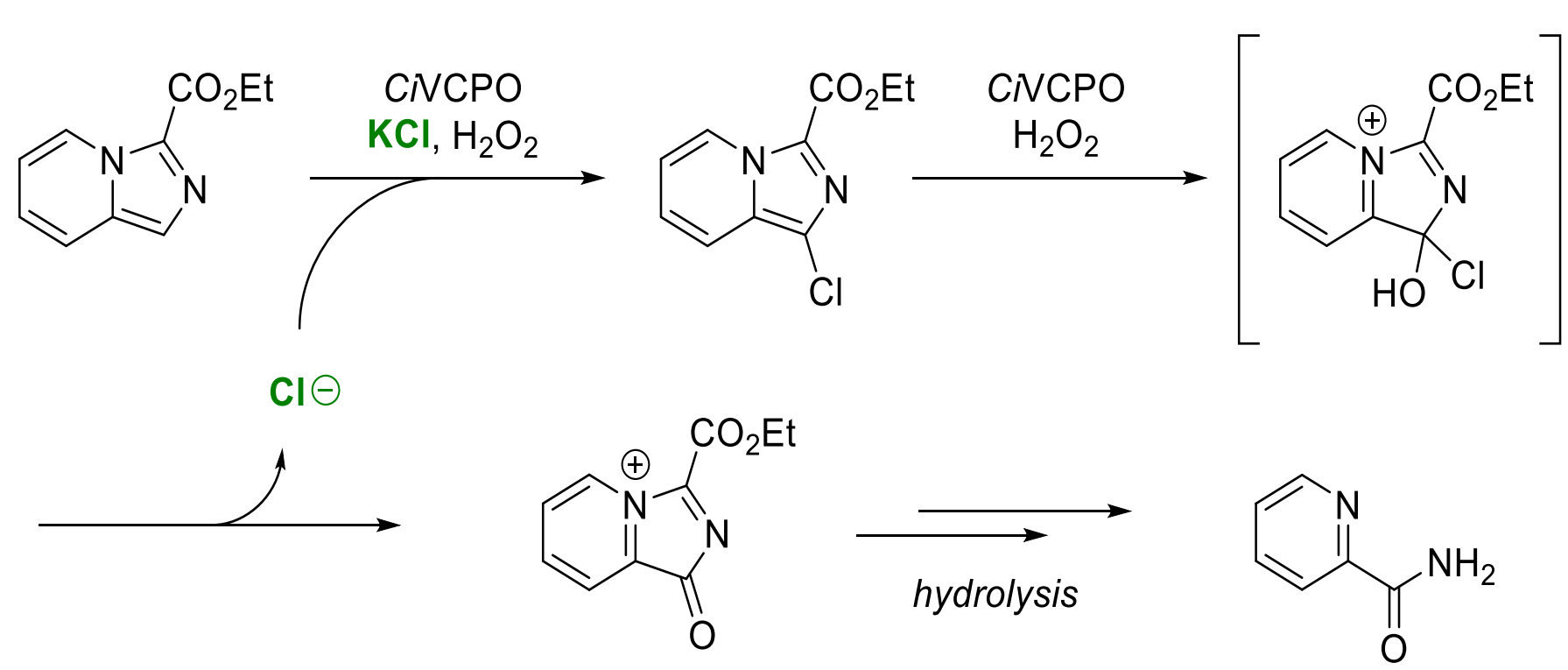

Figure S26: PMI Comparison of *Ci*VCPO Indazole Halogenation Compared to NBS

Calculated using the ACS Green Chemistry Institute's Process Mass Intensity Calculator: https://www.acs.org/green-chemistry-sustainability/green-chemistry-nexus/articles/process-mass-intensity-calculation-tool.htmL.

NBS Mediated Bromination		
Assay Batch Size (input pure)	1.0	g
Assay Kg product (output pure)	1.5	g
Raw Materials	Physical Charge	Units
Main Substrate (Enter only 1 substrate)		
2Me-Indazole	1.00	g
Reagents		
NBS	1.41	g
Solvents		
MeCN (Rxn solvent)	60.0	g
EtOAc (Organic extraction)	100.0	g
Aqueous		
water (Aqeuous wash)	100.0	g
Mass Substrate (g)		1
Mass Reagents (g)		1.4
Mass Solvents (g)		128
Mass Aqueous (g)		100
Step PMI		153.6
Step PMI Substrate, Reagents, Solvents		86.9
Step PMI Substrates and Reagents		1.6
Step PMI Solvents		85.3
Step PMI Water		66.7

Ci VCPO Catalyzed Bromination		
Assay Batch Size (input pure)	1.0	g
Assay Kg product (output pure)	1.5	g
Raw Materials	Physical Charge	Units
Main Substrate (Enter only 1 substrate)		
2Me-Indazole	1.00	g
Reagents		
Na3VO4	0.14	g
H2O2	0.50	g
KBr	0.99	g
Solvents		
THF (Organic Cosolvent)	62.0	g
EtOAc (Organic extraction)	200.0	g
Aqueous		
Water (Solvent)	400.0	g
NaOAc buffer	3.0	g
Mass Substrate (g)		1
Mass Reagents (g)		1.6
Mass Solvents (g)		242
Mass Aqueous (g)		403
Step PMI		440.6
Step PMI Substrate, Reagents, Solvents		166.4
Step PMI Substrates and Reagents		1.8
Step PMI Solvents		164.6
Step PMI Water		274.1

Figure S27: Comparison of *Ci*VCPO Yields (LCAP) Compared to NCS / NBS

To benchmark the performance of our highest-performing *Ci*VCPO catalyzed conditions, we subjected a selection of our substrate scope to representative halogenation conditions with NCS or NBS (1.05 eq NCS / NBS, 0.1M SM concentration in anhydrous MeCN, 16 hrs, r.t., 0.1 mmol scale) and compared the relative LCAP yields.

SM	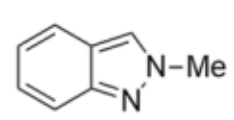 2-methyl-2*H*-indazole Exact Mass: 132.07	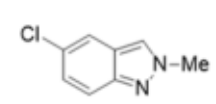 5-chloro-2-methyl-2*H*-indazole Exact Mass: 166.03	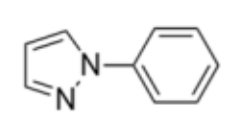 1-phenyl-1*H*-pyrazole Exact Mass: 144.07	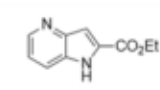 ethyl 1*H*-pyrrolo[3,2-*b*]pyridine-2-carboxylate Exact Mass: 190.07	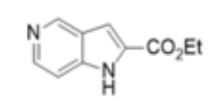 ethyl 1*H*-pyrrolo[3,2-*c*]pyridine-2-carboxylate Exact Mass: 190.07	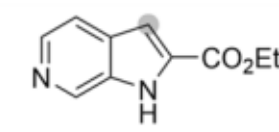 ethyl 1*H*-pyrrolo[2,3-*c*]pyridine-2-carboxylate	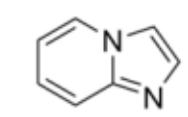 imidazo[1,2-*a*]pyridine Exact Mass: 118.05
NCS	1	0	0	0	20	20	2
Ci VCPO Cl	68	23	5	82	96	96	99
NBS	95	99	99	24	35	80	10
Ci VCPO - Br	87	68	54	95	98	98	93

SM	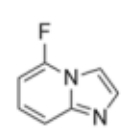 5-fluoroimidazo[1,2-*a*]pyridine Exact Mass: 136.04	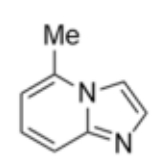 5-methylimidazo[1,2-*a*]pyridine Exact Mass: 132.07	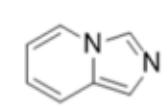 imidazo[1,5-*a*]pyridine Exact Mass: 118.05	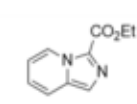 ethyl imidazo[1,5-*a*]pyridine-3-carboxylate Exact Mass: 190.07	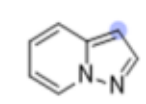 pyrazolo[1,5-*a*]pyridine Exact Mass: 118.05	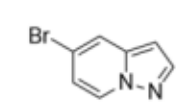 5-bromopyrazolo[1,5-*a*]pyridine Exact Mass: 195.96	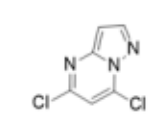 5,7-dichloropyrazolo[1,5-*a*]pyrimidine Exact Mass: 186.97
NCS	21	9	64	80	70	35	4
Ci VCPO Cl	98	74	66	0	84	97	62
NBS	90	11	61	99	95	100	97
Ci VCPO - Br	96	68	63	94	99	93	97

General Procedure For Preparative Scale Heterocycle Halogenation with *Ci*VCPO:

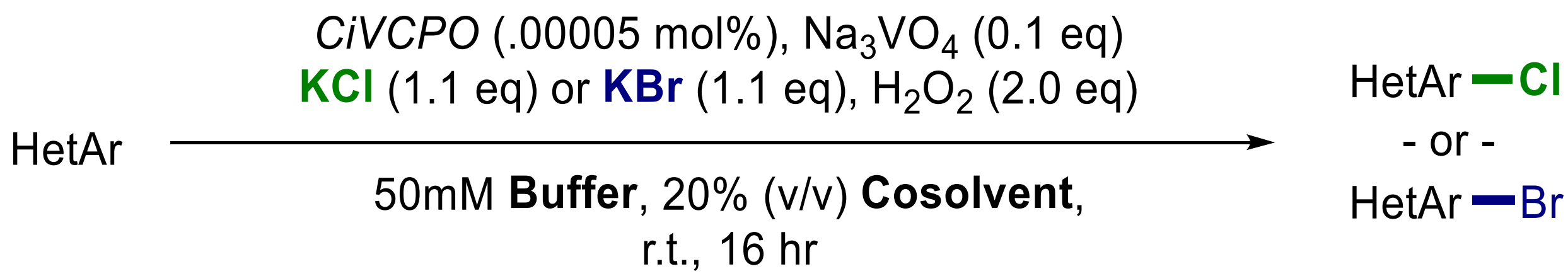

Two enzyme aliquots of purified VHPO from *Curvularia inaequalis* (*Ci*VCPO, 10 μM, 1.0 mL) was removed from a -80°C freezer and allowed to warm to room temperature over 5 min. The thawed aliquot was then combined with an aqueous solution of 250 mM Na_3VO_4 (160 μL, 0.1 equiv) in a microcentrifuge tube and stored at room temperature. To ensure mixing, the resulting solution was spun in a mini centrifuge for 10 seconds and placed on the benchtop until further use. To a 100 mL round bottom flask containing a magnetic stir bar, purified H_2O (21 mL), **Cosolvent** (4.0 mL), **500mM Buffer** (4.0 mL) and 100mM KCl or KBr (4.4 mL, 1.1 equiv) was added. The starting material (0.40 mmol, 1 equiv) was dissolved in **Cosolvent** (4.0 mL) and added to the reaction mixture. The microcentrifuge tubes containing the CiVCPO (0.00005 mol%, 2.0 mL total volume) and Na_3VO_4 (0.1 equiv) was then added to the reaction mixture. A 10% stock of H_2O_2 (264 μL, 2 equiv) was then added to the reaction mixture and the resulting solution was allowed to stir at room temperature for 16h. The reaction mixture was then diluted with EtOAc (40 mL) and transferred to a 125 mL separatory funnel. The organic layer was collected, and the aqueous layer was re-extracted 3X with additional EtOAc (20 mL). The combined organic layer was then washed with saturated brine (50 mL), dried on sodium sulfate for 10 minutes, concentrated under reduced pressure, and further purified via flash chromatography or recrystallization if needed (see individual entries for details).

Note: It is generally recommended to add the starting material as a solution in cosolvent, as adding the SM directly as a solid occasionally led to precipitation or formation of oily phases at the bottom of the reaction flask.

__**Characterization Data of Halogenated Products:**__

3-chloro-2-methyl-2H-indazole (2)

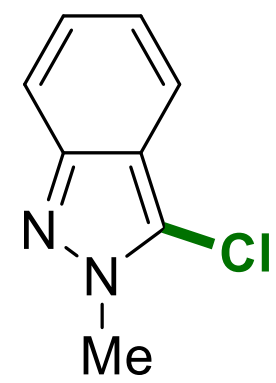

	Chlorination			
**LCAP**	**DMSO**	**DMF**	**MeCN**	**THF**
**Acetate 4**	0	29	4	8
**Acetate 5**	0	15	8	21
**Citrate 5**	0	4	4	5
**Citrate 6**	0	0	2	0
**MES 6**	0	42	53	49
**PIPES 6.5**	0	41	52	50
**TRIS 8**	0	0	0	0
**CAPS 10**	0	0	0	0

Yield: 53%

Purification Method: 0 --> 30% EtOAc / Hexanes

^{1}H NMR (400 MHz, $CDCl_3$) δ 7.62 (dt, *J* = 8.9, 0.9 Hz, 1H), 7.54 (dt, *J* = 8.5, 1.0 Hz, 1H), 7.29 (ddd, *J* = 8.8, 6.6, 1.1 Hz, 1H), 7.10 (ddd, *J* = 8.5, 6.6, 0.8 Hz, 1H), 4.15 (d, *J* = 0.8 Hz, 3H).

^{13}C NMR (101 MHz, $CDCl_3$) δ 147.89, 126.92, 122.25, 119.76, 119.03, 118.66, 117.74, 37.59.

HRMS: calculated for $C_8H_8N_2{}^{35}Cl$ [M+H]+: 167.0370. Found [M+H]+: 167.0364

3-bromo-2-methyl-2H-indazole (3)

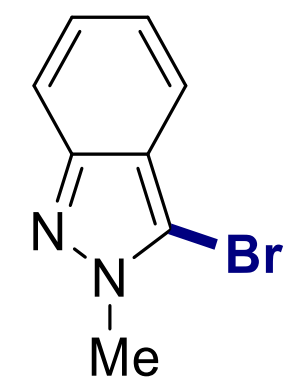

	Bromination			
LCAP	DMSO	DMF	MeCN	THF
Acetate 4	0	85	80	87
Acetate 5	45	46	52	55
Citrate 5	38	39	51	51
Citrate 6	22	29	30	28
MES 6	40	35	52	43
PIPES 6.5	33	30	41	35
TRIS 8	3	3	4	4
CAPS 10	3	0	3	4

Yield: 92%

Purification Method: 0 --> 30% EtOAc / Hexanes

^{1}H NMR (400 MHz, $CDCl_3$) δ 7.65 (dq, J = 8.9, 0.9 Hz, 1H), 7.50 (dt, J = 8.4, 1.0 Hz, 1H), 7.30 (ddt, J = 8.8, 6.6, 1.1 Hz, 1H), 7.12 (ddt, J = 8.5, 6.7, 0.9 Hz, 1H), 4.20 (d, J = 1.0 Hz, 3H).

^{13}C NMR (101 MHz, $CDCl_3$) δ 148.58, 127.00, 122.48, 122.14, 119.32, 117.76, 106.50, 38.98.

HRMS: calculated for $C_8H_8N_2{}^{79}Br$ [M+H]+: 210.9865. Found [M+H]+: 210.9862

3-chloro-5-fluoro-2-methyl-2H-indazole (4)

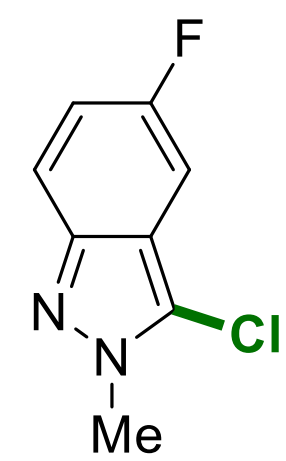

Yield: 41%

Purification Method: 0 --> 30% EtOAc / Hexanes

^{1}H NMR (400 MHz, $CDCl_3$) δ 7.59 (ddd, *J* = 9.3, 4.5, 0.9 Hz, 1H), 7.15 – 7.04 (m, 2H), 4.14 (s, 3H).

^{13}C NMR (101 MHz, $CDCl_3$) δ 159.87, 157.47, 145.19, 120.05, 119.95, 118.67, 118.38, 101.53, 101.28, 37.81.

HRMS: calculated $C_8H_7N_2{}^{35}ClF$ [M+H]+: 185.0276. Found [M+H]+: 185.0275

3-bromo-5-fluoro-2-methyl-2H-indazole (5)

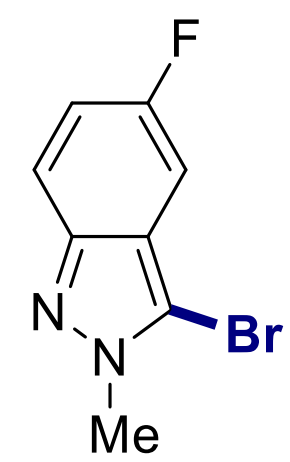

Yield: 97%

Purification Method: Aqueous wash only, no further purification

^{1}H NMR (400 MHz, $CDCl_3$) δ 7.62 (ddq, *J* = 9.0, 4.6, 1.0 Hz, 1H), 7.14 – 7.03 (m, 2H), 4.18 (d, *J* = 1.7 Hz, 3H).

^{13}C NMR (101 MHz, $CDCl_3$) δ 160.01, 157.60, 145.80, 143.46, 121.59, 121.47, 119.99, 119.89, 118.69, 118.40, 106.27, 106.18, 102.18, 101.93, 39.17.

HRMS: calculated $C_8H_7N_2{}^{79}BrF$ [M+H]+: 228.9771. Found [M+H]+: 228.9774

3,5-dichloro-2-methyl-2H-indazole (6)

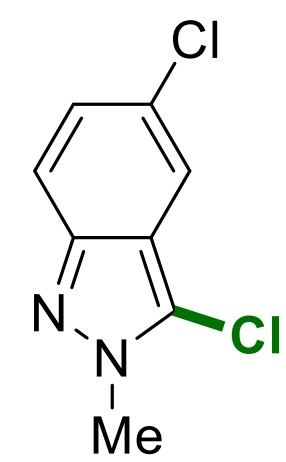

Yield: 23%

Purification Method: 0 --> 20% EtOAc / Hexanes

^{1}H NMR (400 MHz, $CDCl_3$) δ 7.59 – 7.49 (m, 2H), 7.21 (dd, *J* = 9.2, 2.0 Hz, 1H), 4.14 (s, 3H).

^{13}C NMR (101 MHz, $CDCl_3$) δ 146.23, 128.39, 128.10, 119.46, 119.41, 117.49, 37.79.

HRMS: calculated $C_8H_7N_2{}^{35}Cl_2$ [M+H]+: 200.9980. Found [M+H]+: 200.9974

3-bromo-5-chloro-2-methyl-2H-indazole (7)

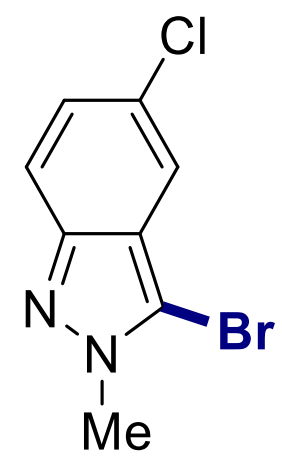

Yield: 57%

Purification Method: 0 --> 30% EtOAc / Hexanes

^{1}H NMR (400 MHz, $CDCl_3$) δ 7.57 (dq, *J* = 9.1, 0.9 Hz, 1H), 7.47 (dt, *J* = 2.2, 1.0 Hz, 1H), 7.21 (dt, *J* = 9.1, 2.0 Hz, 1H), 4.17 (d, *J* = 2.1 Hz, 3H).

^{13}C NMR (101 MHz, $CDCl_3$) δ 146.84, 128.38, 128.22, 122.53, 119.35, 118.10, 106.04, 39.14.

HRMS: calculated $C_8H_7N_2{}^{79}Br{}^{35}Cl$ [M+H]+: 244.9475. Found [M+H]+: 244.947

3-bromo-2-phenyl-2H-indazole (9)

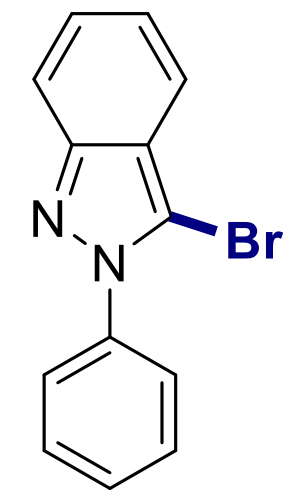

Yield: 38%

Purification Method: 0 --> 5% EtOAc / Hexanes, 1% increments

^{1}H NMR: (400 MHz, $CDCl_3$) δ 7.74 (dt, *J* = 8.8, 0.9 Hz, 1H), 7.71 – 7.66 (m, 2H), 7.62 – 7.51 (m, 4H), 7.37 (ddd, *J* = 8.8, 6.6, 1.1 Hz, 1H), 7.19 (ddd, *J* = 8.6, 6.6, 0.9 Hz, 1H).

^{13}C NMR: (101 MHz, $CDCl_3$) δ 149.33, 139.37, 129.39, 129.20, 127.74, 126.31, 123.12, 123.02, 119.84, 118.32, 106.31.

NMR data was consistent with literature reported values[7]

3-bromo-2-(4-fluorophenyl)-2H-indazole (11)

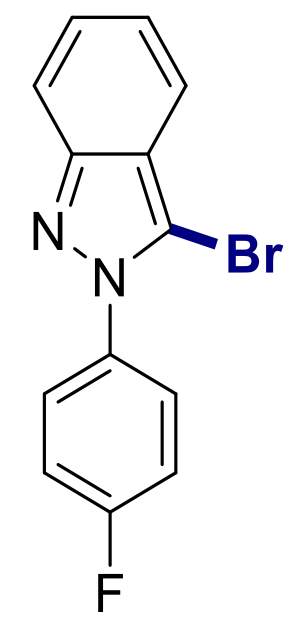

Yield: 69%

Purification Method: 0 -->5% EtOAc / Hexanes,. 1% increments

^{1}H NMR (400 MHz, $CDCl_3$) δ 7.76 (dt, J = 8.8, 0.9 Hz, 1H), 7.73 – 7.66 (m, 2H), 7.61 (dt, J = 8.5, 1.1 Hz, 1H), 7.41 (ddd, J = 8.8, 6.6, 1.1 Hz, 1H), 7.32 – 7.25 (m, 2H), 7.22 (ddd, J = 8.5, 6.6, 0.8 Hz, 1H).

^{13}C NMR (101 MHz, $CDCl_3$) δ 164.15, 161.67, 149.38, 135.51, 135.48, 128.27, 128.18, 127.90, 123.28, 123.00, 119.81, 118.29, 116.36, 116.13, 106.48.

NMR data was consistent with literature reported values[7]:

4-chloro-1-phenyl-1H-pyrazole (13)

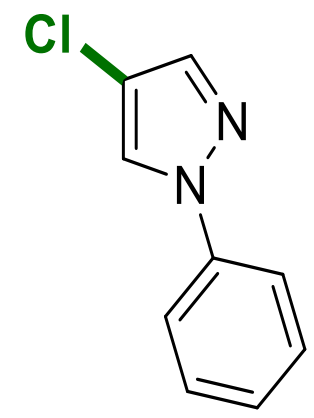

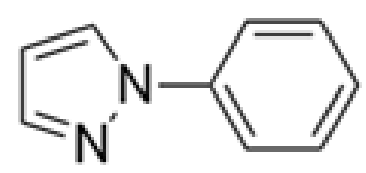 1-phenyl-1*H*-pyrazole<br>Exact Mass: 144.07	***Chlorination***			
**LCAP**	***DMSO***	***DMF***	***MeCN***	***THF***
Acetate 4	0	5	0	1
Acetate 5	0	3	1	1
Citrate 5	0	2	1	1
Citrate 6	0	0	0	0
MES 6	0	5	5	3
PIPES 6.5	0	2	2	1
TRIS 8	0	0	0	0
CAPS 10	0	0	0	0

*This chloro-pyrazole was prepared chemically to confirm the identity of the product observed in the Ci*VCPO *mediated chlorination on analytical scale.*

Prepared according to the method reported by Kalyani and Sanford[8]: A 20 mL scintillation vial was charged with 1-phenyl-1H-pyrazole (150 mg, 1 Eq, 1.04 mmol), NCS (146 mg, 1.05 Eq, 1.09 mmol), and dissolved in Acetic Acid (8.7 mL). The vial was placed in a pre-heated 100°C aluminum heating block and was stirred vigorously overnight (~16hr). Upon completion, the reaction was diluted with 10 mL of DCM and transferred to a 100 mL round bottom flask. The acetic acid was quenched with ~10 mL of $NaHCO_3$ (***caution, significant effervescence)*** and the organic layer was separated and dried on sodium sulfates. The crude material was concentrated in vacuo, and purified via flash column chromatography (0--> 20% EtOAc) to yield -chloro-1-phenyl-1H-pyrazole (165 mg) as a white solid.

Yield: 88%

^{1}H NMR (400 MHz, $CDCl_3$) δ 7.94 (s, 1H), 7.70 – 7.61 (m, 3H), 7.46 (t, J = 7.7 Hz, 2H), 7.32 (t, J = 7.4 Hz, 1H).

NMR data was consistent with literature reported values[8]

4-bromo-1-phenyl-1H-pyrazole (14)

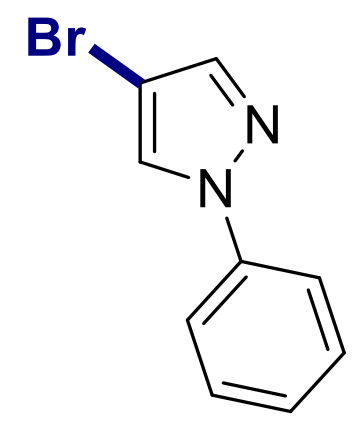

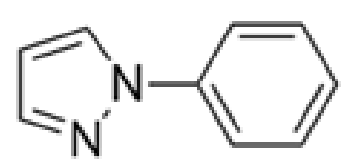 1-phenyl-1*H*-pyrazole Exact Mass: 144.07	***Bromination***			
**LCAP**	***DMSO***	***DMF***	***MeCN***	***THF***
Acetate 4	0	**33**	**54**	**26**
Acetate 5	0	16	14	7
Citrate 5	0	15	17	6
Citrate 6	0	12	10	5
MES 6	0	4	7	4
PIPES 6.5	0	3	2	2
TRIS 8	0	0	0	0
CAPS 10	0	0	0	0

Yield: 52%

Purification Method: Direct filtration of aqueous reaction mixture over glass-fritted funnel

^{1}H NMR (400 MHz, $CDCl_3$) δ 7.94 (s, 1H), 7.70 – 7.61 (m, 3H), 7.46 (t, *J* = 7.6 Hz, 2H), 7.32 (s, 1H).

^{13}C NMR (101 MHz, $CDCl_3$) δ 141.66, 139.76, 129.69, 127.19, 127.15, 119.16, 95.76.

NMR data was consistent with literature reported values[9].

4-chloro-3,5-dimethyl-1-phenyl-1H-pyrazole (15)

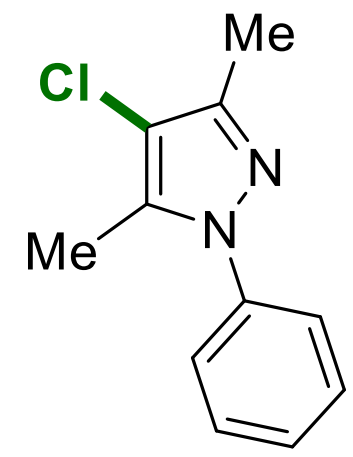

Yield: 91%

Purification Method: Aqueous wash only, no chromatography

^{1}H NMR (400 MHz, $CDCl_3$) δ 7.61 – 7.48 (m, 5H), 2.42 (s, 6H).

^{13}C NMR (101 MHz, $CDCl_3$) δ 146.17, 139.86, 135.82, 129.30, 127.84, 124.66, 109.93, 11.54, 10.96.

NMR data was consistent with literature reported values[10]

4-chloro-3,5-dimethyl-1-phenyl-1H-pyrazole (16)

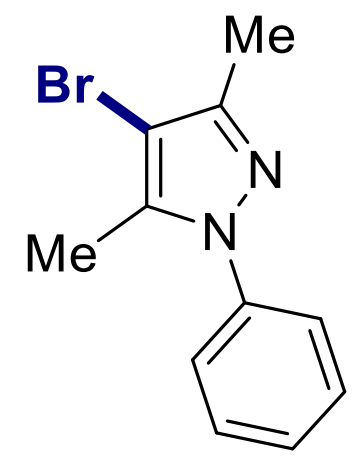

Yield: 91%

Purification Method: Aqueous wash only, no chromatography

^{1}H NMR (400 MHz, $CDCl_3$) δ 7.62 – 7.47 (m, 5H), 2.43 (d, *J* = 1.9 Hz, 6H).

^{13}C NMR (101 MHz, $CDCl_3$) δ 147.68, 139.92, 137.61, 129.29, 127.93, 124.78, 96.49, 12.50, 11.89.

NMR data was consistent with literature reported values[10]

4-(4-chloro-3,5-dimethyl-1H-pyrazol-1-yl)benzonitrile (17)

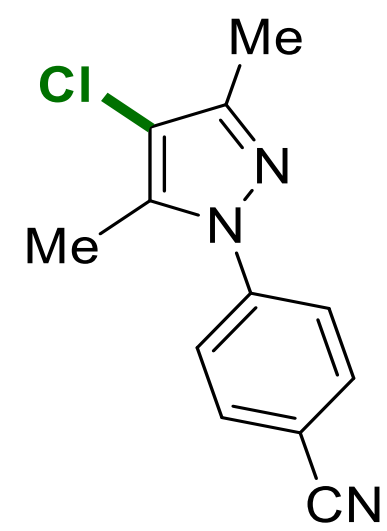

Yield: 97%

Purification Method: Aqueous wash only, no chromatography

^{1}H NMR (400 MHz, $CDCl_3$) δ 7.80 – 7.71 (m, 2H), 7.65 – 7.56 (m, 2H), 2.39 (s, 3H), 2.30 (s, 3H).

^{13}C NMR (101 MHz, $CDCl_3$) δ 147.87, 143.26, 135.94, 133.41, 123.95, 118.32, 111.96, 110.81, 11.59, 11.51.

NMR data was consistent with literature reported values[11]

4-(4-chloro-3,5-dimethyl-1H-pyrazol-1-yl)benzonitrile (18)

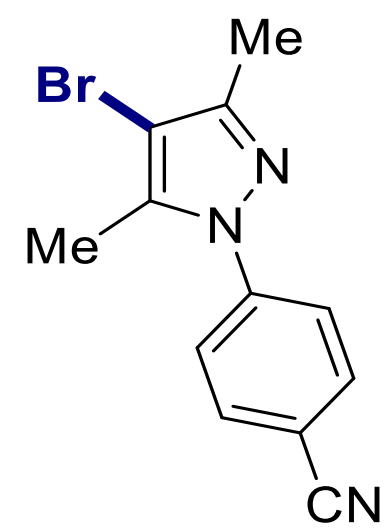

Yield: 95%

Purification Method: Aqueous wash only, no chromatography

1H-NMR: (400 MHz, $CDCl_3$) δ 7.81 – 7.73 (m, 2H), 7.62 – 7.55 (m, 2H), 2.40 (s, 3H), 2.30 (s, 3H).

^{13}C NMR (101 MHz, $CDCl_3$) δ 149.35, 143.28, 137.74, 133.40, 124.14, 118.31, 110.94, 98.68, 12.56, 12.45.

NMR data was consistent with literature reported values[12]

4-chloro-3,5-dimethyl-1-(4-(trifluoromethoxy)phenyl)-1H-pyrazole (19)

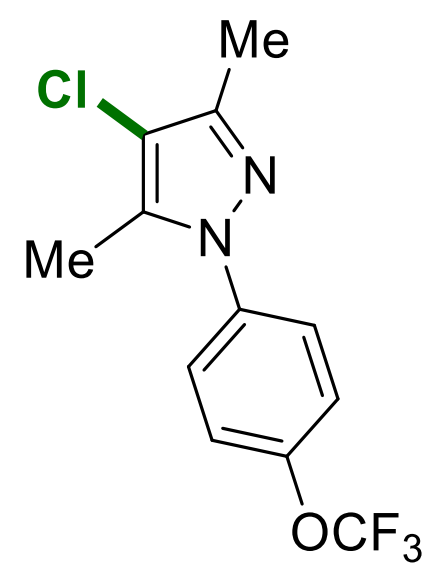

Yield: 73%

Purification Method: Flash column chromatography (0 --> 20% EtOAc / Hex)

^{1}H NMR (400 MHz, $CDCl_3$) δ 7.49 – 7.40 (m, 2H), 7.36 – 7.27 (m, 2H), 2.31 (s, 3H), 2.29 (s, 3H).

^{13}C NMR (101 MHz, $CDCl_3$) δ 148.32, 148.30, 146.74, 138.35, 135.91, 125.89, 121.91, 119.25, 110.49, 11.53, 11.00.

HRMS: calculated $C_{12}H_{11}ON_2{}^{35}ClF_3$ [M+H]+: 291.0506. Found [M+H]+: 291.0508

4-chloro-3,5-dimethyl-1-(4-(trifluoromethoxy)phenyl)-1H-pyrazole (20)

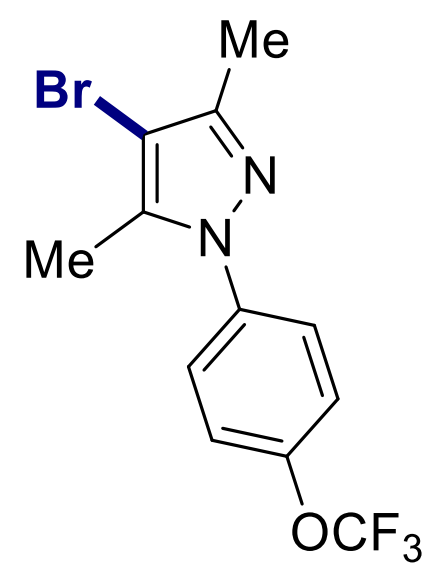

Yield: 85%

Purification Method: Flash column chromatography (0 --> 20% EtOAc / Hex)

^{1}H NMR (400 MHz, $CDCl_3$) δ 7.48 – 7.41 (m, 2H), 7.32 (d, *J* = 8.5 Hz, 2H), 2.32 (s, 3H), 2.29 (s, 3H).

^{13}C NMR (101 MHz, $CDCl_3$) δ 148.39, 148.24, 138.40, 137.70, 126.03, 121.90, 119.24, 97.06, 12.50, 11.93.

NMR data was consistent with literature reported values[12]

4-chloro-3,5-dimethyl-1-(tetrahydro-2H-pyran-4-yl)-1H-pyrazole (21)

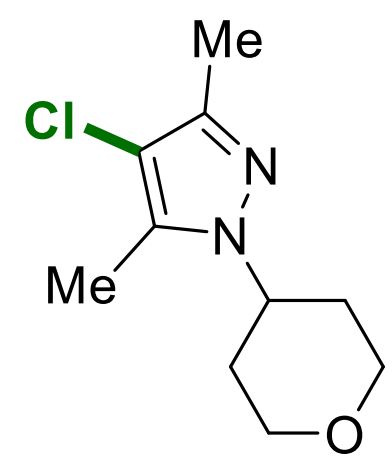

Yield: 86%

Purification Method: Flash column chromatography (0 --> 50% EtOAc / Hex)

^{1}H NMR (400 MHz, $CDCl_3$) δ 4.20 – 4.08 (m, 3H), 3.52 (td, *J* = 12.2, 2.0 Hz, 2H), 2.37 – 2.19 (m, 8H), 1.85 – 1.74 (m, 2H).

^{13}C NMR (101 MHz, $CDCl_3$) δ 144.22, 134.22, 107.61, 67.16, 55.45, 32.53, 11.42, 9.34.

HRMS: calculated $C_{12}H_{11}ON_2{}^{35}ClF_3$ [M+H]+: 215.0945. Found [M+H]+: 215.0947

4-chloro-3,5-dimethyl-1-(tetrahydro-2H-pyran-4-yl)-1H-pyrazole (22)

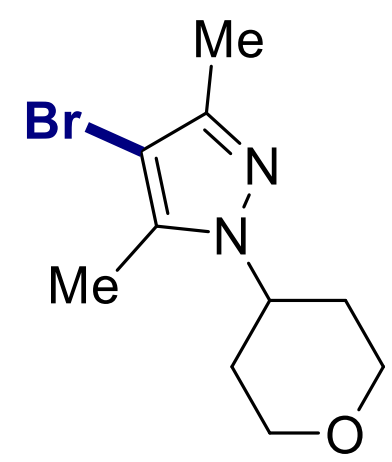

Yield: 95%

Purification Method: Aqueous wash only, no chromatography.

^{1}H NMR (400 MHz, $CDCl_3$) δ 4.20 – 4.06 (m, 3H), 3.50 (td, *J* = 12.2, 2.0 Hz, 2H), 2.34 – 2.14 (m, 8H), 1.83 – 1.72 (m, 2H).

^{13}C NMR (101 MHz, $CDCl_3$) δ 145.82, 136.00, 94.18, 67.16, 55.62, 32.56, 12.37, 10.30.

HRMS: calculated $C_{10}H_{16}ON_2{}^{79}Br$ [M+H]+: 259.0440. Found [M+H]+: 259.0441

ethyl 3-chloro-1H-pyrrolo[3,2-b]pyridine-2-carboxylate (24)

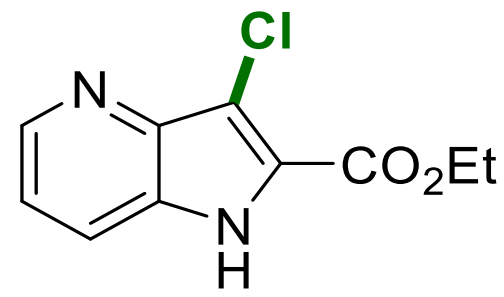

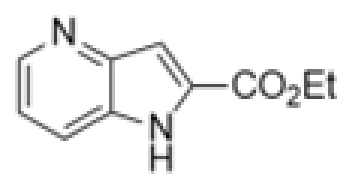 ethyl 1*H*-pyrrolo[3,2-*b*]pyridine-2-carboxylate<br>Exact Mass: 190.07	***Chlorination***			
**LCAP**	***DMSO***	***DMF***	***MeCN***	***THF***
Acetate 4	0	42	50	54
Acetate 5	0	33	41	43
Citrate 5	0	3	41	48
Citrate 6	0	20	22	27
MES 6	1	72	69	82
PIPES 6.5	2	62	69	69
TRIS 8	0	0	1	1
CAPS 10	0	0	1	0

Yield: 82%

Purification Method: Flash column chromatography (0 --> 75% EtOAc / Hex)

^{1}H NMR (400 MHz, DMSO) δ 12.39 (s, 1H), 8.53 (dd, *J* = 4.4, 1.4 Hz, 1H), 7.88 (dd, *J* = 8.4, 1.4 Hz, 1H), 7.38 (dd, *J* = 8.4, 4.4 Hz, 1H), 4.40 (q, *J* = 7.1 Hz, 2H), 1.37 (t, *J* = 7.1 Hz, 3H).

^{13}C NMR (101 MHz, DMSO) δ 159.93, 145.38, 140.56, 128.71, 124.74, 120.99, 109.82, 61.19, 14.21.

HRMS: calculated $C_{10}H_{10}O_2N_2{}^{35}Cl$ [M+H]+: 225.0425. Found [M+H]+: 225.0425

ethyl 3-bromo-1H-pyrrolo[3,2-b]pyridine-2-carboxylate (25)

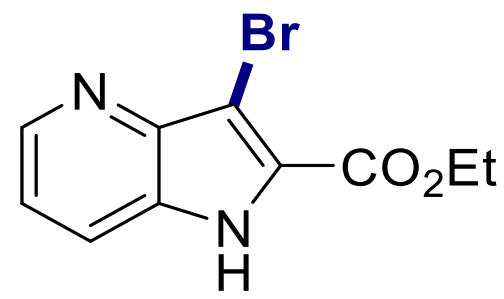

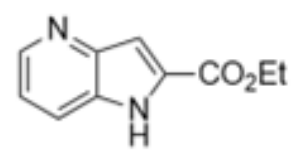 ethyl 1*H*-pyrrolo[3,2-*b*]pyridine-2-carboxylate Exact Mass: 190.07	***Bromination***			
**LCAP**	***DMSO***	***DMF***	***MeCN***	***THF***
Acetate 4	80	85	89	85
Acetate 5	84	90	90	89
Citrate 5	66	65	55	72
Citrate 6	87	90	**91**	**95**
MES 6	82	89	**95**	91
PIPES 6.5	29	28	30	30
TRIS 8	36	37	28	37
CAPS 10	0	0	0	0

Yield: 92%

Purification Method: Aqueous wash only, no chromatography

^{1}H NMR (400 MHz, DMSO) δ 12.48 (s, 1H), 8.53 (dd, *J* = 4.4, 1.4 Hz, 1H), 7.88 (dd, *J* = 8.4, 1.4 Hz, 1H), 7.37 (dd, *J* = 8.4, 4.4 Hz, 1H), 4.40 (q, *J* = 7.1 Hz, 2H), 1.38 (t, *J* = 7.1 Hz, 3H).

^{13}C NMR (101 MHz, DMSO) δ 160.04, 145.46, 142.17, 129.25, 126.33, 120.96, 120.93, 96.76, 61.22, 14.21.

HRMS: calculated $C_{10}H_{10}O_2N_2{}^{79}Br$ [M+H]+: 268.9920. Found [M+H]+: 225.0425

ethyl 3-chloro-1H-pyrrolo[3,2-c]pyridine-2-carboxylate (26)

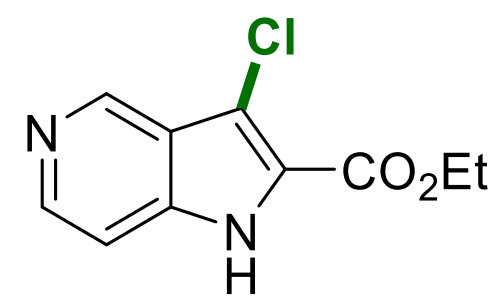

Yield: 71%

Purification Method: Flash column chromatography (0 à 75% EtOAc / Hex)

^{1}H NMR (400 MHz, DMSO) δ 12.55 (s, 1H), 8.94 (s, 1H), 8.37 (d, *J* = 5.9 Hz, 1H), 7.43 (dd, *J* = 5.9, 1.1 Hz, 1H), 4.39 (q, *J* = 7.1 Hz, 2H), 1.36 (t, *J* = 7.1 Hz, 3H).

^{13}C NMR (101 MHz, DMSO) δ 159.64, 143.77, 143.22, 138.24, 123.53, 122.21, 109.41, 107.73, 61.24, 14.20.

HRMS: calculated $C_{10}H_{10}O_2N_2{}^{35}Cl$ [M+H]+: 225.0425. Found [M+H]+: 225.0423

ethyl 3-chloro-1H-pyrrolo[3,2-c]pyridine-2-carboxylate (27)

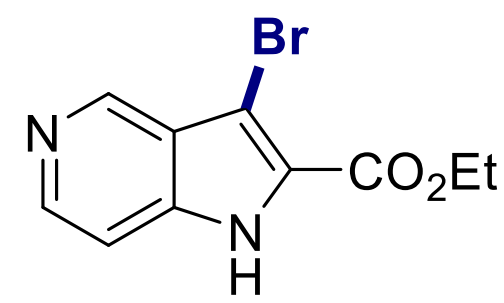

Yield: 80%

Purification Method: Flash column chromatography (0 à 75% EtOAc / Hex)

^{1}H NMR (400 MHz, DMSO) δ 12.63 (s, 1H), 8.85 (s, 1H), 8.37 (d, *J* = 5.9 Hz, 1H), 7.43 (dd, *J* = 5.9, 1.1 Hz, 1H), 4.39 (q, *J* = 7.1 Hz, 2H), 1.37 (t, *J* = 7.1 Hz, 3H).

^{13}C NMR (101 MHz, DMSO) δ 159.78, 144.14, 143.77, 139.13, 125.22, 124.00, 107.67, 95.29, 61.26, 14.19.

HRMS: calculated $C_{10}H_{10}O_2N_2{}^{79}Br$ [M+H]+: 268.9920. Found [M+H]+: 268.9924

ethyl 3-chloro-1H-pyrrolo[2,3-c]pyridine-2-carboxylate (28)

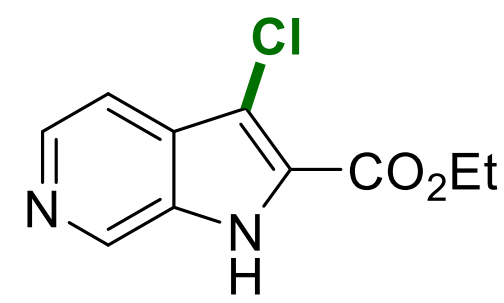

Yield: 88%

Purification Method: Flash column chromatography (0 à 100% EtOAc / Hex)

^{1}H NMR (400 MHz, DMSO) δ 12.69 (s, 1H), 8.89 (d, *J* = 1.2 Hz, 1H), 8.28 (d, *J* = 5.6 Hz, 1H), 7.60 (dd, *J* = 5.6, 1.2 Hz, 1H), 4.42 (q, *J* = 7.1 Hz, 2H), 1.38 (t, *J* = 7.1 Hz, 3H).

^{13}C NMR (101 MHz, DMSO) δ 160.10, 139.49, 137.44, 131.97, 129.40, 125.80, 113.48, 108.86, 61.85, 14.61.

HRMS: calculated $C_{10}H_{10}O_2N_2{}^{35}Cl$ [M+H]+: 225.0425. Found [M+H]+: 225.0424

ethyl 3-bromo-1H-pyrrolo[2,3-c]pyridine-2-carboxylate (29)

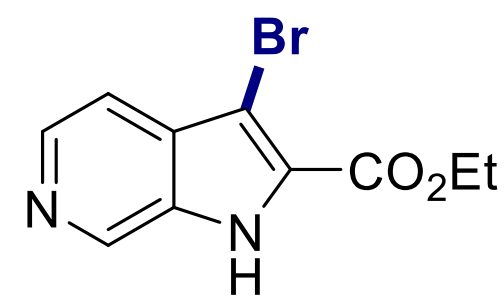

Yield: 94%

Purification Method: Flash column chromatography (0 --> 100% EtOAc / Hex)

^{1}H NMR (400 MHz, DMSO) δ 12.78 (s, 1H), 8.88 (d, *J* = 1.2 Hz, 1H), 8.29 (d, *J* = 5.6 Hz, 1H), 7.53 (dd, *J* = 5.6, 1.2 Hz, 1H), 4.42 (q, *J* = 7.1 Hz, 2H), 1.39 (t, *J* = 7.1 Hz, 3H).

^{13}C NMR (101 MHz, DMSO) δ 160.24, 139.64, 137.41, 132.86, 131.40, 127.40, 114.46, 94.86, 61.88, 14.61.

HRMS: calculated $C_{10}H_{10}O_2N_2{}^{79}Br$ [M+H]+: 268.9920. Found [M+H]+: 268.9922

ethyl 3-chloro-1H-pyrrolo[2,3-b]pyridine-2-carboxylate (30)

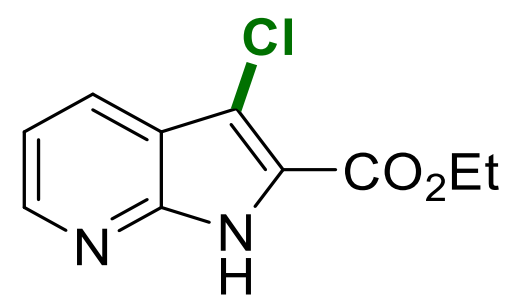

This chlorinated product was inseparable from the aza-indole starting-material by column chromatography, and was characterized as a mixture and referenced to trimethoxy-benzene as an internal standard for an NMR yield. No other products were observed. Only the signals of the chlorinated product are reported.

NMR Yield: 48%

^{1}H NMR (400 MHz, DMSO) δ 12.82 (s, 1H), 8.52 (dd, *J* = 4.6, 1.6 Hz, 1H), 8.11 (ddd, *J* = 8.1, 4.6, 1.6 Hz, 1H), 7.28 (dd, *J* = 8.1, 4.6 Hz, 1H), 4.37 (dt, *J* = 14.2, 7.1 Hz, 2H), 1.36 (t, *J* = 7.1 Hz, 2H).

HRMS: calculated $C_{10}H_{10}O_2N_2{}^{35}Cl$ [M+H]+: 225.0425. Found [M+H]+: 225.0422

ethyl 3-chloro-1H-pyrrolo[2,3-b]pyridine-2-carboxylate (31)

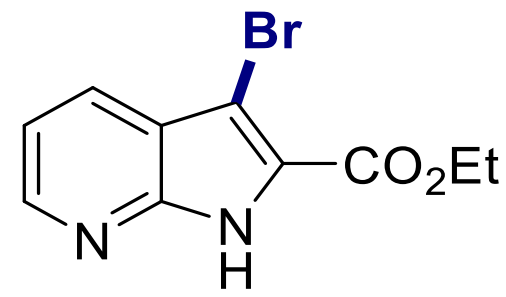

This brominated product was inseparable from the aza-indole starting-material by column chromatography, and was characterized as a mixture and referenced to trimethoxy-benzene as an internal standard for an NMR yield. No other products were observed. Only the signals of the brominated product are reported.

NMR Yield: 65%

^{1}H NMR (400 MHz, DMSO) δ 12.90 (s, 1H), 8.56 – 8.47 (m, 1H), 8.04 – 7.97 (m, 1H), 7.28 (dd, *J* = 8.0, 4.6 Hz, 1H), 4.38 (q, *J* = 7.0 Hz, 2H), 1.37 (t, *J* = 7.1 Hz, 3H).

HRMS: calculated $C_{10}H_{10}O_2N_2{}^{79}Br$ [M+H]+: 268.9920. Found [M+H]+: 268.9921

3-chloro-2-methyl-1H-pyrrolo[3,2-b]pyridine (32)

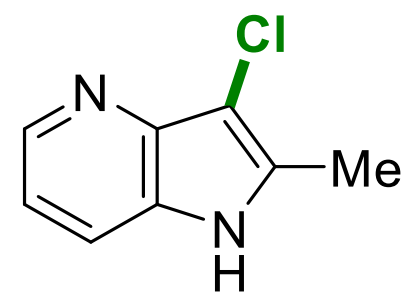

Yield: 63%

Purification Method: Flash column chromatography (0 à 5% MeOH / DCM, 1% increments)

^{1}H NMR (400 MHz, DMSO) δ 11.57 (s, 1H), 8.30 (dd, *J* = 4.6, 1.4 Hz, 1H), 7.70 (dd, *J* = 8.1, 1.4 Hz, 1H), 7.10 (dd, *J* = 8.1, 4.6 Hz, 1H), 2.42 (s, 3H).

^{13}C NMR (101 MHz, DMSO) δ 142.63, 141.88, 135.64, 127.13, 118.25, 116.67, 101.07, 11.35.

HRMS: calculated $C_8H_8N_2{}^{35}Cl$ [M+H]+: 167.0370. Found [M+H]+: 167.0370

3-bromo-2-methyl-1H-pyrrolo[3,2-b]pyridine (33)

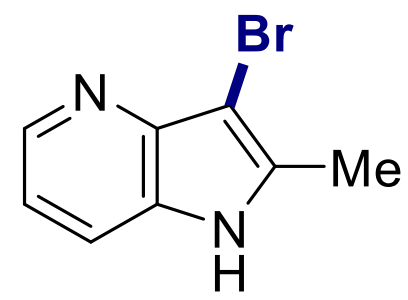

Yield: 71%

Purification Method: Flash column chromatography (0 à 5% MeOH / DCM, 1% increments)

^{1}H NMR (400 MHz, DMSO) δ 11.69 (s, 1H), 8.30 (dd, *J* = 4.6, 1.4 Hz, 1H), 7.69 (dd, *J* = 8.1, 1.4 Hz, 1H), 7.10 (dd, *J* = 8.1, 4.6 Hz, 1H), 2.43 (s, 3H).

^{13}C NMR (101 MHz, DMSO) δ 143.32, 142.72, 137.45, 127.84, 118.19, 116.73, 88.68, 12.46.

HRMS: calculated $C_8H_8N_2{}^{79}Br$ [M+H]+: 210.9865. Found [M+H]+: 210.9865

3-chloroimidazo[1,2-a]pyridine (35)

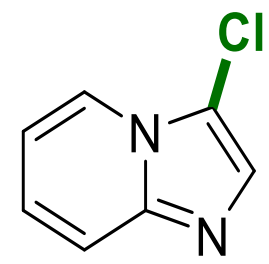

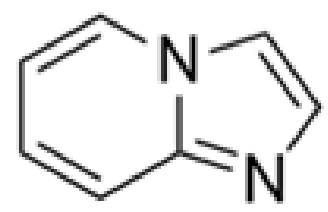 imidazo[1,2-*a*]pyridine<br>Exact Mass: 118.05	*Chlorination*			
LCAP	***DMSO***	***DMF***	***MeCN***	***THF***
Acetate 4	0	78	24	0
Acetate 5	0	87	90	93
Citrate 5	0	**93**	**94**	**100**
Citrate 6	0	67	60	62
MES 6	33	51	47	47
PIPES 6.5	89	36	59	59
TRIS 8	10	8	3	3
CAPS 10	0	0	0	0

Yield: 94%

Purification Method: no purification, aqueous wash only

^{1}H NMR (400 MHz, $CDCl_3$) δ 8.05 (dd, *J* = 6.9, 1.2 Hz, 1H), 7.59 (dd, *J* = 9.1, 1.1 Hz, 1H), 7.54 (s, 1H), 7.20 (ddd, *J* = 9.2, 6.7, 1.3 Hz, 1H), 6.91 (td, *J* = 6.8, 1.1 Hz, 1H).

^{13}C NMR (101 MHz, $CDCl_3$) δ 144.58, 130.22, 124.42, 122.68, 118.16, 113.10, 109.73.

NMR data was consistent with literature reported values[13]

3-chloroimidazo[1,2-a]pyridine (36)

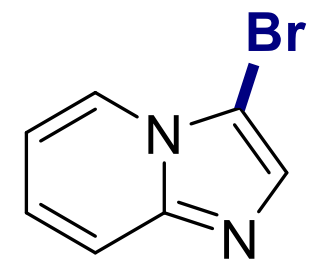

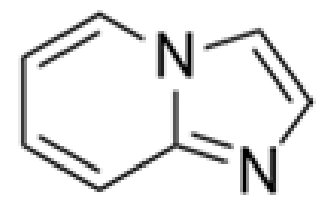 imidazo[1,2-*a*]pyridine Exact Mass: 118.05	*Bromination*			
LCAP	***DMSO***	***DMF***	***MeCN***	***THF***
Acetate 4	10	24	58	25
Acetate 5	40	39	30	53
Citrate 5	48	39	46	43
Citrate 6	**89**	76	73	78
MES 6	69	72	72	74
PIPES 6.5	85	77	74	80
TRIS 8	88	82	87	**93**
CAPS 10	**89**	77	50	0

Yield: 96%

Purification Method: no purification, aqueous wash only

^{1}H NMR (400 MHz, $CDCl_3$) δ 8.10 (dt, *J* = 6.9, 1.2 Hz, 1H), 7.64 – 7.57 (m, 2H), 7.21 (ddd, *J* = 9.3, 6.8, 1.3 Hz, 1H), 6.91 (td, *J* = 6.8, 1.1 Hz, 1H).

^{13}C NMR (101 MHz, $CDCl_3$) δ 145.97, 133.73, 124.58, 123.83, 118.12, 113.23, 94.86.

NMR data was consistent with literature reported values[14]

3-chloro-5-fluoroimidazo[1,2-a]pyridine (37)

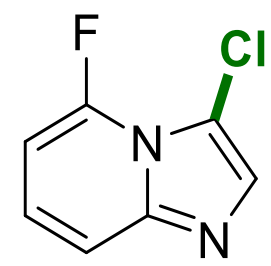

Yield: 89%

Purification Method: Flash column chromatography (0 à 40% EtOAc)

^{1}H NMR (400 MHz, $CDCl_3$) δ 7.89 (dd, *J* = 6.8, 1.1 Hz, 1H), 7.56 (s, 1H), 6.91 (ddd, *J* = 10.3, 7.6, 1.1 Hz, 1H), 6.84 (td, *J* = 7.2, 4.5 Hz, 1H).

^{13}C NMR (101 MHz, $CDCl_3$) δ 152.76, 150.22, 137.21, 136.91, 130.51, 125.51, 119.09, 119.04, 112.26, 112.19, 111.49, 107.16, 107.00.

HRMS: calculated $C_7H_5N_2{}^{35}ClF$ [M+H]+: 171.0119. Found [M+H]+: 171.0119

3-bromo-5-fluoroimidazo[1,2-a]pyridine (38)

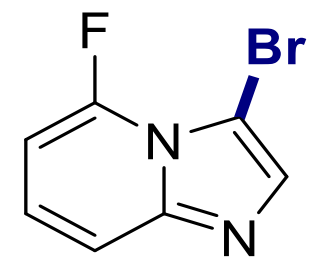

Yield: 93%

Purification Method: Aqueous wash only, no chromatography.

^{1}H NMR (400 MHz, $CDCl_3$) δ 7.93 (dd, J = 6.8, 1.0 Hz, 1H), 7.61 (s, 1H), 6.93 (ddt, J = 9.7, 7.5, 1.0 Hz, 1H), 6.90 – 6.78 (m, 1H).

^{13}C NMR (101 MHz, $CDCl_3$) δ 152.67, 150.13, 144.85, 143.99, 138.55, 138.26, 133.91, 120.23, 120.18, 112.30, 112.23, 107.34, 107.17, 96.60.

HRMS: calculated $C_7H_5N_2{}^{79}BrF$ [M+H]+: 214.9614. Found [M+H]+: 214.9614

3-chloro-2-phenylimidazo[1,2-a]pyridine (39)

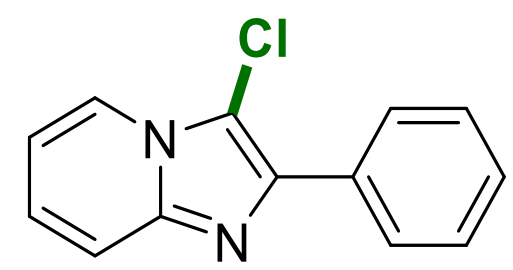

Yield: 93%

Purification Method: Flash column chromatography (0 à 30% EtOAc)

^{1}H NMR (400 MHz, $CDCl_3$) δ 8.27 – 8.03 (m, 3H), 7.64 (dt, *J* = 9.1, 1.1 Hz, 1H), 7.54 – 7.44 (m, 2H), 7.44 – 7.35 (m, 1H), 7.27 (dd, *J* = 6.9, 1.4 Hz, 1H), 6.94 (td, *J* = 6.9, 1.2 Hz, 1H).

^{13}C NMR (101 MHz, $CDCl_3$) δ 145.63, 142.88, 133.05, 128.61, 128.44, 128.04, 125.20, 124.12, 117.82, 113.18, 91.84.

NMR data was consistent with literature reports[15]

3-bromo-2-phenylimidazo[1,2-a]pyridine (40)

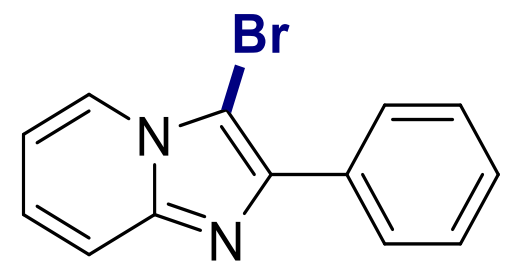

Yield: 98%

Purification Method: 0 --> 30% EtOAc / Hexanes

^{1}H NMR (400 MHz, $CDCl_3$) δ 8.13 (ddt, J = 11.6, 6.9, 1.3 Hz, 3H), 7.64 (dt, J = 9.1, 1.1 Hz, 1H), 7.53 – 7.44 (m, 2H), 7.43 – 7.34 (m, 1H), 7.26 – 7.22 (m, 1H), 6.93 (td, J = 6.8, 1.2 Hz, 1H).

^{13}C NMR (101 MHz, $CDCl_3$) δ 143.88, 140.00, 132.72, 128.69, 128.38, 127.64, 124.94, 122.84, 117.85, 113.03, 105.82.

NMR Data was consistent with literature reports[16]

3,6-dichloroimidazo[1,2-a]pyridine (42)

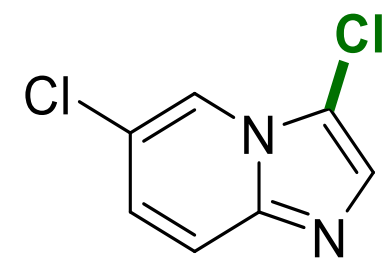

Yield: 86%

Purification Method: Flash column chromatography (0 à 20% EtOAc)

^{1}H NMR (400 MHz, $CDCl_3$) δ 8.13 (dd, *J* = 2.0, 0.9 Hz, 1H), 7.62 – 7.51 (m, 2H), 7.19 (dd, *J* = 9.6, 2.0 Hz, 1H).

^{13}C NMR (101 MHz, $CDCl_3$) δ 143.02, 131.36, 126.00, 121.83, 120.75, 118.69, 110.35.

HRMS: calculated $C_7H_5N_2{}^{35}Cl_2$ [M+H]+: 186.9824. Found [M+H]+: 186.9825

3-bromo-6-chloroimidazo[1,2-a]pyridine (43)

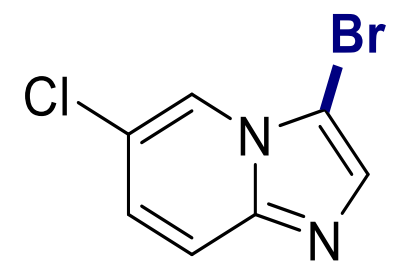

Yield: 96%

Purification Method: Aqueous wash only, no chromatography.

^{1}H NMR (400 MHz, $CDCl_3$) δ 8.17 (dd, *J* = 2.0, 0.9 Hz, 1H), 7.64 (s, 1H), 7.57 (dd, *J* = 9.6, 0.9 Hz, 1H), 7.21 (dd, *J* = 9.6, 2.0 Hz, 1H).

^{13}C NMR (101 MHz, $CDCl_3$) δ 144.32, 134.66, 126.07, 121.85, 121.80, 118.47, 95.37.

HRMS: calculated $C_7H_5N_2{}^{79}Br^{35}Cl$ [M+H]+: 230.9319. Found [M+H]+: 230.9319

3-chloro-5-methylimidazo[1,2-a]pyridine (44)

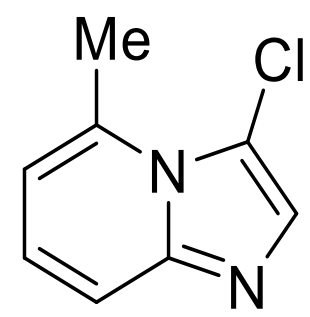

Yield: 62%

Purification Method: Flash column chromatography (0 à 50% EtOAc)

^{1}H NMR (400 MHz, DMSO) δ 11.57 (s, 1H), 8.30 (dd, *J* = 4.6, 1.4 Hz, 1H), 7.70 (dd, *J* = 8.1, 1.4 Hz, 1H), 7.10 (dd, *J* = 8.1, 4.6 Hz, 1H), 2.42 (s, 3H).

^{13}C NMR (101 MHz, $CDCl_3$) δ 146.52, 136.25, 132.25, 124.88, 116.81, 114.24, 110.44, 20.50.

HRMS: calculated $C_8H_8N_2{}^{35}Cl$ [M+H]+: 167.0370. Found [M+H]+: 167.0370

3-bromo-5-methylimidazo[1,2-a]pyridine (45)

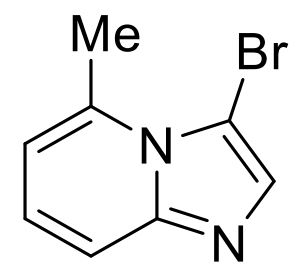

Yield: 76%

Purification Method: Flash column chromatography (0 à 50% EtOAc)

^{1}H NMR (400 MHz, $CDCl_3$) δ 7.52 (s, 1H), 7.50 – 7.43 (m, 1H), 7.05 (dd, *J* = 9.1, 6.8 Hz, 1H), 6.50 (dt, *J* = 6.8, 1.1 Hz, 1H), 3.03 (s, 3H).

^{13}C NMR (101 MHz, $CDCl_3$) δ 147.81, 136.59, 136.15, 124.95, 116.86, 114.36, 92.96, 21.22.

HRMS: calculated $C_8H_8N_2{}^{79}Br$ [M+H]+: 210.9865. Found [M+H]+: 210.98633

3-chloropyrazolo[1,5-a]pyridine (46)

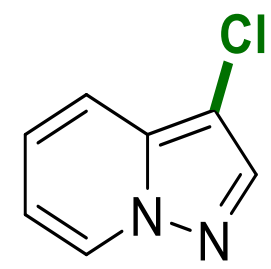

Yield: 92%

Purification Method: 0 --> 30% EtOAc / Hexanes

^{1}H NMR (400 MHz, $CDCl_3$) δ 8.38 (dt, *J* = 6.9, 1.1 Hz, 1H), 7.88 (s, 1H), 7.51 (dt, *J* = 8.9, 1.2 Hz, 1H), 7.15 (ddd, *J* = 8.9, 6.7, 1.0 Hz, 1H), 6.77 (td, *J* = 6.9, 1.4 Hz, 1H).

^{13}C NMR (101 MHz, $CDCl_3$) δ 139.64, 136.80, 129.04, 123.90, 116.16, 112.41, 99.97.

HRMS: calculated $C_7H_6N_2{}^{35}Cl$ [M+H]+: 153.0135. Found [M+H]+: 153.0146

3-bromopyrazolo[1,5-a]pyridine (47)

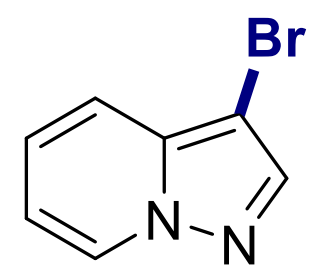

Yield: 93%

Purification Method: Aqueous wash only, no chromatography.

^{1}H NMR (400 MHz, $CDCl_3$) δ 8.41 (dt, *J* = 7.0, 1.1 Hz, 1H), 7.91 (s, 1H), 7.49 (dt, *J* = 8.9, 1.2 Hz, 1H), 7.17 (ddd, *J* = 9.0, 6.7, 1.1 Hz, 1H), 6.78 (td, *J* = 6.9, 1.4 Hz, 1H).

^{13}C NMR (101 MHz, $CDCl_3$) δ 141.79, 138.25, 129.11, 124.28, 116.76, 112.48, 83.79.

HRMS: calculated $C_7H_6N_2{}^{35}Cl$ [M+H]+: 196.9652. Found [M+H]+: 196.9667

Procedure for 6.0 mmol scale bromination with purified *Ci*VCPO:

Six 5 mL enzyme aliquots of purified VHPO from *Curvularia inaequalis* (*Ci*VCPO, 10 µM, 30.0 mL total) were removed from a -80°C freezer and allowed to warm to room temperature over 5 min in a room temperature water bath. The combined thawed aliquots were then combined with an aqueous solution of 250 mM Na_3VO_4 (2.4 mL total, 0.1 equiv) and stored at room temperature. To a 1L Erlenmeyer, purified H_2O (440 mL), THF (60.0 mL), 500mM TRIS (pH = 8.0, 60 mL) and KBr (0.79g, 1.1 equiv) was added. The starting material (6.0 mmol, 0.71g, 1 equiv) was dissolved in THF (60 mL) and added to the reaction mixture. The *Ci*VCPO (0.00005 mol%, 30 mL total volume) and Na_3VO_4 (0.1 equiv) solution was then added to the reaction mixture and briefly mixed. A 10% stock of H_2O_2 (1.4 mL, 2 equiv) was then added to the reaction mixture and the resulting solution was stirred at 200 RPM in a 25°C incubator for 16 hours. Upon completion, the reaction mixture was then diluted with EtOAc (300 mL) and transferred to a 1L separatory funnel. The organic layer was collected, and the aqueous layer was re-extracted 3X with additional EtOAc (100 mL). The combined organic layer was then washed with saturated brine (250 mL), dried on sodium sulfate for 10 minutes, concentrated under reduced pressure, yielding 3-bromopyrazolo[1,5-a]pyridine (1.095g, 93% yield).

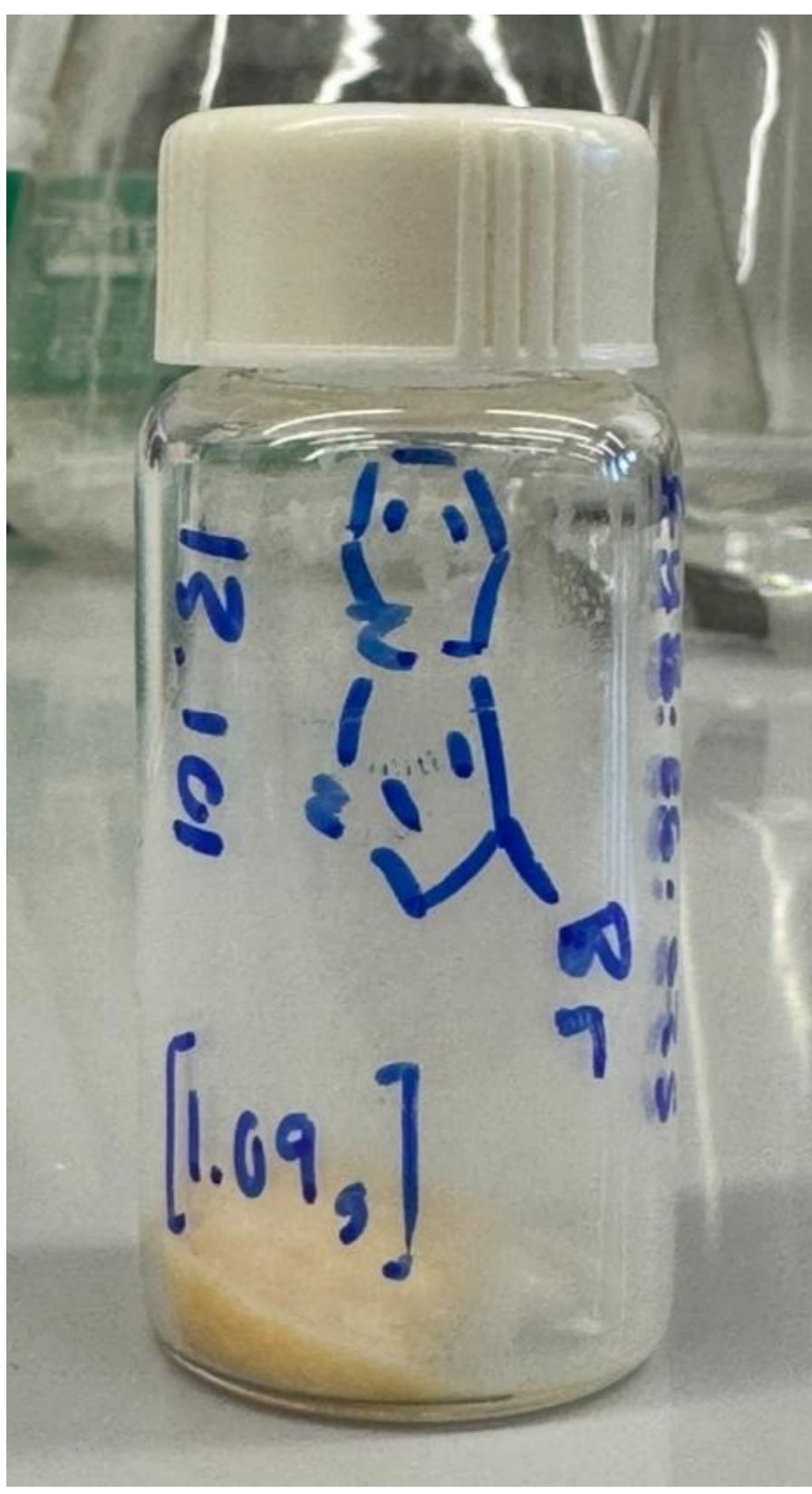

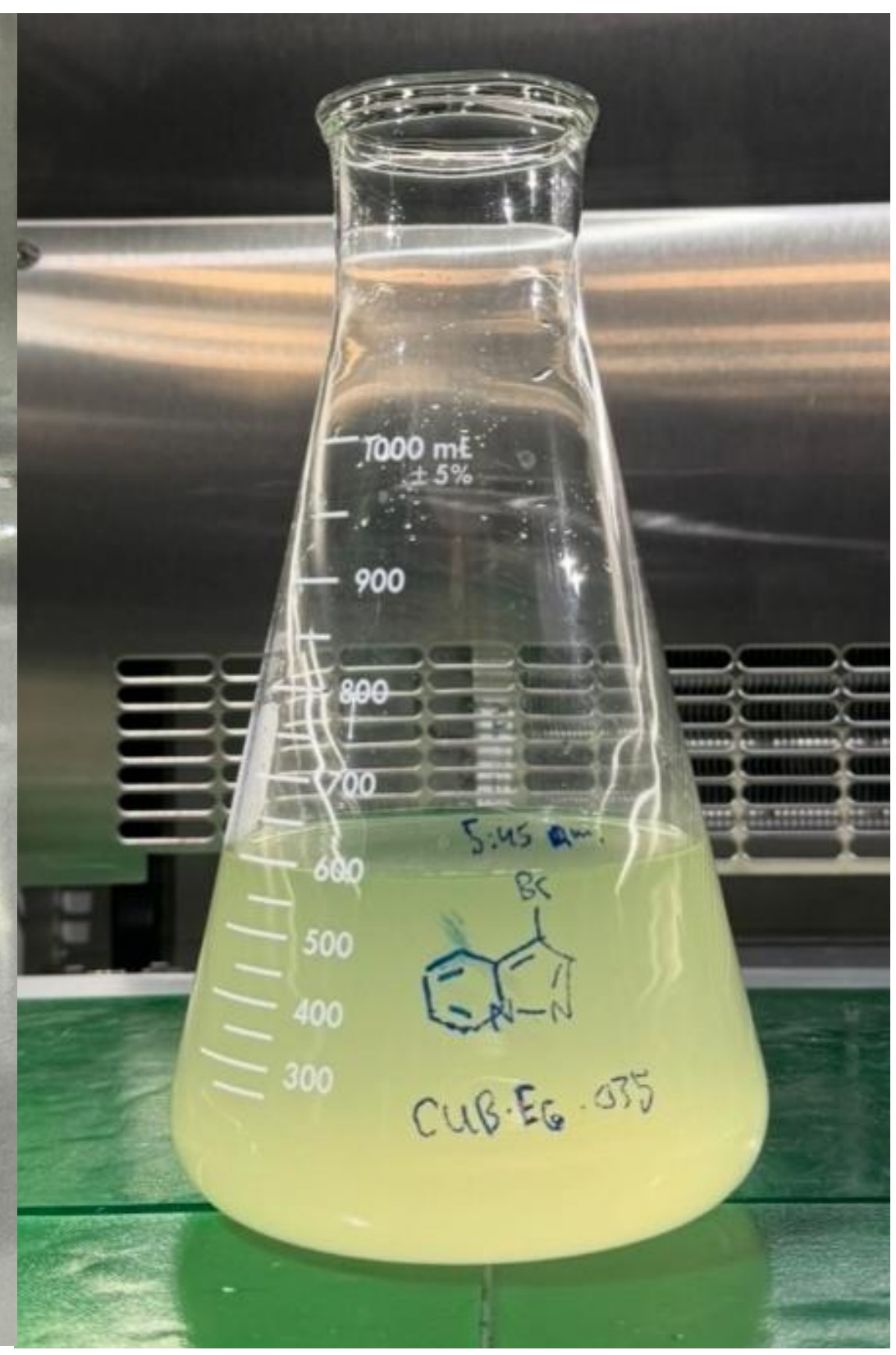

5-bromo-3-chloropyrazolo[1,5-a]pyridine (48)

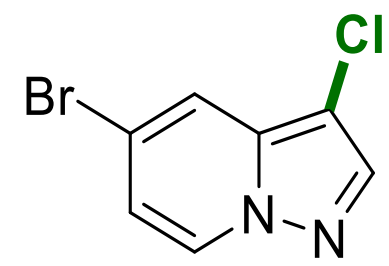

Yield: 87%

Purification Method: Flash column chromatography (0 à 30% EtOAc)

^{1}H NMR (400 MHz, $CDCl_3$) δ 8.24 (dd, *J* = 7.4, 0.8 Hz, 1H), 7.88 (s, 1H), 7.71 (dd, *J* = 2.1, 0.8 Hz, 1H), 6.86 (dd, *J* = 7.4, 2.2 Hz, 1H).

^{13}C NMR (101 MHz, $CDCl_3$) δ 140.80, 137.31, 129.62, 118.49, 118.18, 116.35, 100.04.

HRMS: calculated $C_7H_5N_2{}^{79}Br^{35}Cl$ [M+H]+: 230.9319. Found [M+H]+: 230.93184

3,5-dibromopyrazolo[1,5-a]pyridine (49)

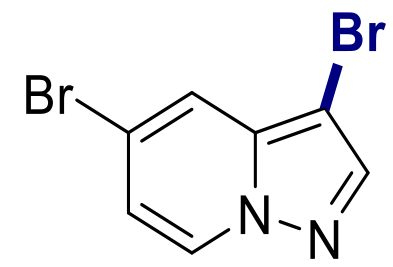

Yield: 93%

Purification Method: Aqueous wash only, no chromatography.

^{1}H NMR (400 MHz, $CDCl_3$) δ 8.27 (dd, J = 7.4, 0.8 Hz, 1H), 7.91 (s, 1H), 7.69 (dd, J = 2.1, 0.8 Hz, 1H), 6.87 (dd, J = 7.4, 2.1 Hz, 1H).

^{13}C NMR (101 MHz, $CDCl_3$) δ 142.92, 138.85, 129.68, 119.12, 118.60, 116.45, 83.80.

HRMS: calculated $C_7H_5N_2{}^{79}Br_2$ [M+H]+: 274.8814. Found [M+H]+: 274.8814

6-bromo-3-chloropyrazolo[1,5-a]pyridine (50)

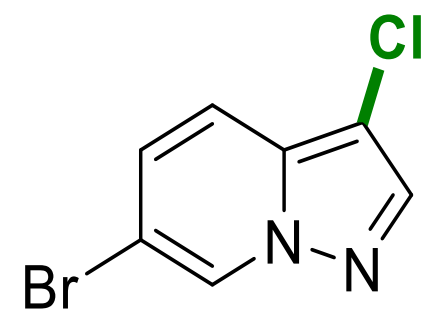

Yield: 92%

Purification Method: Flash column chromatography (0 à 30% EtOAc)

^{1}H NMR (400 MHz, $CDCl_3$) δ 8.58 (dd, *J* = 1.6, 0.8 Hz, 1H), 7.89 (s, 1H), 7.40 (dd, *J* = 9.3, 0.9 Hz, 1H), 7.25 (dd, *J* = 9.3, 1.5 Hz, 1H).

^{13}C NMR (101 MHz, $CDCl_3$) δ 142.29, 136.86, 129.34, 127.92, 117.29, 107.25, 85.02.

HRMS: calculated $C_7H_5N_2{}^{79}Br^{35}Cl$ [M+H]+: 230.9319. Found [M+H]+: 230.9319

3,6-dibromopyrazolo[1,5-a]pyridine (51)

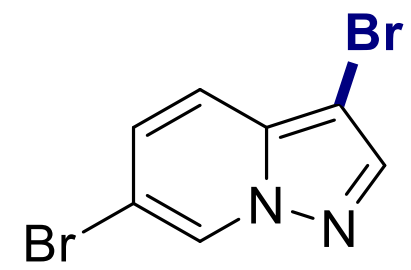

Yield: 92%

Purification Method: Flash column chromatography (0 à 30% EtOAc)

^{1}H NMR (400 MHz, $CDCl_3$) δ 8.55 (dd, *J* = 1.6, 0.9 Hz, 1H), 7.86 (s, 1H), 7.43 (dd, *J* = 9.3, 0.9 Hz, 1H), 7.28 – 7.20 (m, 1H).

^{13}C NMR (101 MHz, $CDCl_3$) δ 140.15, 135.39, 129.30, 127.54, 116.71, 107.18, 101.24.

HRMS: calculated $C_7H_5N_2{}^{79}Br_2$ [M+H]+: 274.8814. Found [M+H]+: 274.8813

3-chloro-2,5,7-trimethylpyrazolo[1,5-a]pyrimidine (52)

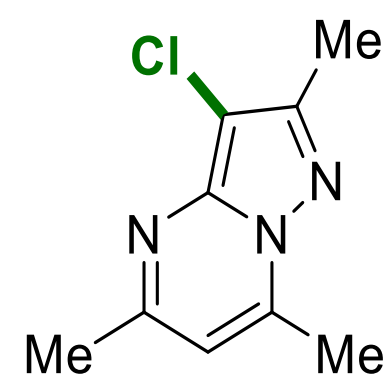

Yield: 92%

Purification Method: Aqueous wash only, no purification

^{1}H NMR (400 MHz, $CDCl_3$) δ 6.52 (q, J = 1.0 Hz, 1H), 2.68 (d, J = 1.0 Hz, 3H), 2.58 (s, 3H), 2.49 (s, 3H).

^{13}C NMR (101 MHz, $CDCl_3$) δ 159.32, 151.29, 145.25, 144.33, 108.61, 97.67, 24.83, 16.68, 12.42.

NMR data was consistent with literature reports[17]

3-bromo-2,5,7-trimethylpyrazolo[1,5-a]pyrimidine (53)

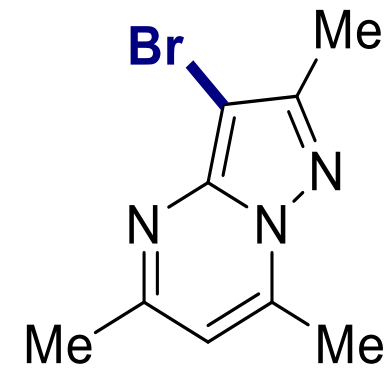

Yield: 93%

Purification Method: aqueous wash only, no purification

^{1}H NMR (400 MHz, $CDCl_3$) δ 6.53 (q, J = 0.9 Hz, 1H), 2.69 (d, J = 1.0 Hz, 3H), 2.59 (s, 3H), 2.49 (s, 3H).

^{13}C NMR (101 MHz, $CDCl_3$) δ 159.67, 152.81, 145.80, 145.32, 108.75, 83.60, 24.88, 16.71, 13.42.

NMR Data was consistent with literature reports[17]

3,5,7-trichloropyrazolo[1,5-a]pyrimidine (54)

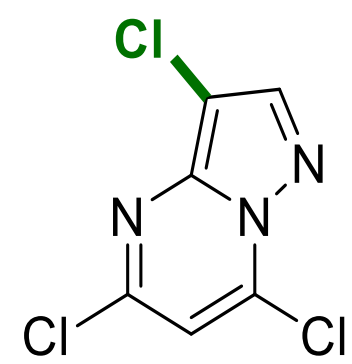

Yield: 47%

Purification Method: Flash column chromatography (0 à 30% EtOAc)

^{1}H NMR (400 MHz, $CDCl_3$) δ 8.19 (s, 1H), 7.03 (s, 1H).

^{13}C NMR (101 MHz, $CDCl_3$) δ 150.43, 144.48, 143.76, 140.68, 109.56, 102.13.

HRMS: calculated $C_6H_3N_3{}^{35}Cl_3$ [M+H]+: 221.9387. Found [M+H]+: 221.9386

3-bromo-5,7-dichloropyrazolo[1,5-a]pyrimidine (55)

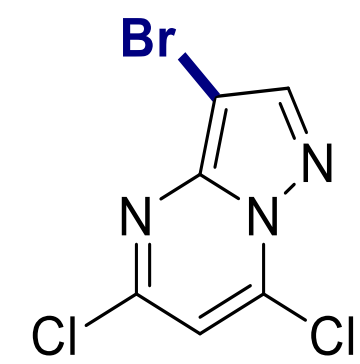

Yield: 96%

Purification Method: Aqueous wash only, no purification

^{1}H NMR (400 MHz, $CDCl_3$) δ 8.22 (s, 1H), 7.04 (s, 1H).

^{13}C NMR (101 MHz, $CDCl_3$) δ 150.75, 146.47, 145.16, 140.60, 109.62, 86.35.

HRMS: calculated $C_6H_3N_3{}^{79}Br^{35}Cl_2$ [M+H]+: 265.8881. Found [M+H]+: 265.8881

1-chloroimidazo[1,5-a]pyridine (56)

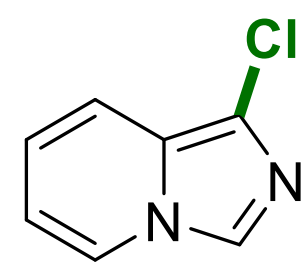

Yield: 66%

Purification Method: 0 --> 30% EtOAc / Hexanes

^{1}H NMR (400 MHz, $CDCl_3$) δ 7.95 (s, 1H), 7.84 (dt, J = 7.1, 1.1 Hz, 1H), 7.38 (dq, J = 9.1, 1.0 Hz, 1H), 6.73 (ddd, J = 9.3, 6.4, 1.0 Hz, 1H), 6.57 (ddd, J = 7.4, 6.4, 1.2 Hz, 1H).

^{13}C NMR (101 MHz, $CDCl_3$) δ 125.48, 124.99, 122.36, 119.50, 118.80, 117.22, 113.59.

HRMS: calculated $C_7H_6N_2{}^{35}Cl$ [M+H]+: 153.0214. Found [M+H]+: 153.0214

1-bromoimidazo[1,5-a]pyridine (57)

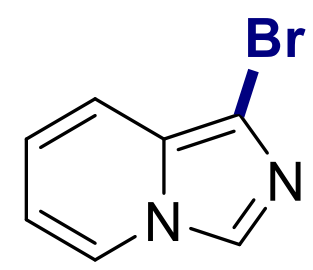

Yield: 72%

Purification Method: 0 --> 30% EtOAc / Hexanes

^{1}H NMR (400 MHz, $CDCl_3$) δ 8.06 (s, 1H), 7.90 (dt, J = 7.1, 1.1 Hz, 1H), 7.37 (dq, J = 9.2, 1.1 Hz, 1H), 6.78 (ddd, J = 9.3, 6.4, 0.9 Hz, 1H), 6.61 (ddd, J = 7.3, 6.5, 1.1 Hz, 1H).

^{13}C NMR (101 MHz, $CDCl_3$) δ 128.07, 126.66, 122.54, 120.06, 117.74, 113.78, 105.00.

HRMS: calculated $C_7H_6N_2{}^{79}Br$ [M+H]+: 196.9708. Found [M+H]+: 196.9704

ethyl 1-bromoimidazo[1,5-a]pyridine-3-carboxylate (59)

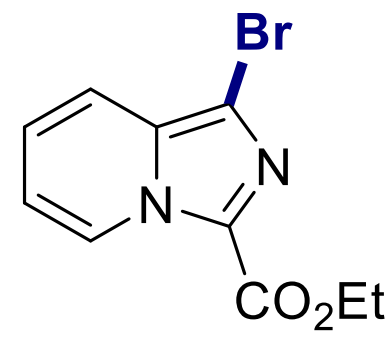

Yield: 94%

Purification Method: Aqueous wash only, no chromatography.

^{1}H NMR (400 MHz, $CDCl_3$) δ 9.34 (dt, *J* = 7.2, 1.1 Hz, 1H), 7.61 (dt, *J* = 9.1, 1.2 Hz, 1H), 7.15 (ddd, *J* = 9.1, 6.7, 1.0 Hz, 1H), 6.98 (ddd, *J* = 7.2, 6.6, 1.3 Hz, 1H), 4.50 (q, *J* = 7.1 Hz, 2H), 1.46 (t, *J* = 7.1 Hz, 3H).

^{13}C NMR (101 MHz, $CDCl_3$) δ 159.06, 132.37, 126.52, 125.88, 123.58, 117.86, 116.51, 108.85, 61.66, 14.61.

HRMS: calculated $C_{10}H_{10}O_2N_2{}^{79}Br$ [M+H]+: 268.9920. Found [M+H]+: 268.9920

Picolinamide (60)

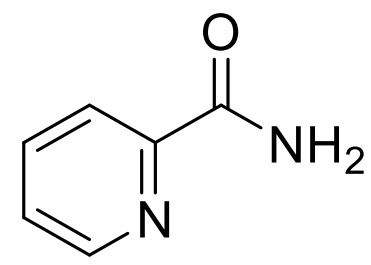

Yield: 59%

Purification Method: 0 --> 5% MeOH / DCM.

^{1}H NMR (400 MHz, $CDCl_3$) δ 8.58 (ddd, *J* = 4.8, 1.8, 1.0 Hz, 1H), 8.21 (dt, *J* = 7.8, 1.1 Hz, 1H), 7.86 (td, *J* = 7.7, 1.7 Hz, 2H), 7.45 (ddd, *J* = 7.6, 4.8, 1.2 Hz, 1H), 5.74 (s, 1H).

^{13}C NMR (151 MHz, $CDCl_3$) δ 166.74, 149.55, 148.33, 137.32, 126.49, 122.48.

NMR data was consistent with literature reports[18]

4-chloro-3-ethyl-1-methyl-1H-pyrazole-5-carboxylic acid (62)

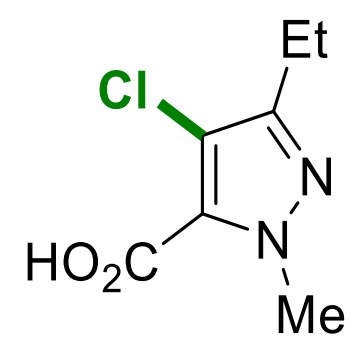

Yield: 90%

Purification Method: Recrystallization from boiling water, then filtration over glass fritted funnel

^{1}H NMR (400 MHz, DMSO) δ 13.80 (s, 1H), 4.00 (s, 3H), 2.54 (q, *J* = 7.6 Hz, 2H), 1.15 (t, *J* = 7.6 Hz, 3H).

^{113}C NMR (101 MHz, DMSO) δ 159.71, 148.91, 129.63, 111.30, 40.44, 18.64, 12.63.

HRMS: calculated $C_7H_{10}O_2N_2{}^{35}Cl$ [M+H]+: 189.0425. Found [M+H]+: 189.0425

4-bromo-3-ethyl-1-methyl-1H-pyrazole-5-carboxylic acid (63)

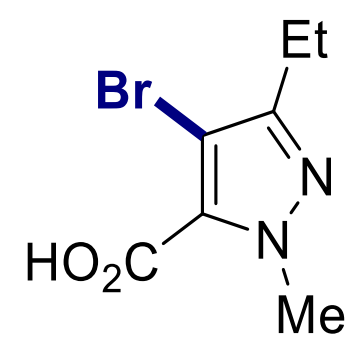

Yield: 91%

Purification Method: Recrystallization from boiling water, then filtration over glass fritted funnel

^{1}H NMR (400 MHz, DMSO) δ 13.80 (s, 1H), 4.01 (s, 3H), 2.53 (q, *J* = 7.6 Hz, 2H), 1.15 (t, *J* = 7.5 Hz, 3H).

^{113}C NMR (101 MHz, DMSO) δ 159.89, 150.58, 131.28, 97.19, 40.56, 19.59, 12.82.

HRMS: calculated $C_7H_{10}O_2N_2{}^{79}Br$ [M+H]+: 232.9920. Found [M+H]+: 232.9919

Procedure for 5.0 mmol scale chlorination with *Ci*VCPO clarified lysate:

A total of 6.25 mL of clarified *Ci*VCPO lysate was removed from a -80°C freezer and allowed to warm to room temperature over 5 min in a room temperature water bath. The combined thawed aliquots were then combined with an aqueous solution of 250 mM Na_3VO_4 (500µl total, 0.025 equiv) and stored at room temperature. To a 1L round bottom flask equipped with a magnetic stir-bar, purified H_2O (285 mL), MeCN (50.0 mL), 500mM PIPES (pH = 6.5, 50 mL) and KCl (55.0 mL, 100mmolar, 1.1 equiv) was added. The starting material (5.0 mmol, 771 mg, 1 equiv) was dissolved in MeCN (50 mL) and added to the reaction mixture. The *Ci*VCPO lysate (1.25% v/v) and Na_3VO_4 (0.025 equiv) solution was then added to the reaction mixture and stirred. A 10% stock of H_2O_2 (3.06 mL, 2.0 equiv) was then added to the reaction mixture and the resulting solution was stirred at 670 RPM at room temperature for 2 hours. An additional charge of H_2O_2 (3.06 mL, 2.0 equiv) was added after 2 hours, and stirred for an additional 2 hours. Upon completion the crude reaction mixture was filtered through a 2-inch thick pad of celite to remove cellular debris, and the filter pad was re- washed with additional 1:1 H_2O / MeCN (100 mL). The filtered reaction mixture was then acidified with 1M HCl to a pH of 3.0, then diluted with 300 mL of EtOAc. This biphasic solution was transferred to a 1L separatory funnel, the organic layer was collected, and the aqueous layer was re-extracted 3X with additional EtOAc (100 mL). The combined organic layer was then washed with saturated brine (250 mL), dried on sodium sulfate for 10 minutes, and concentrated under reduced pressure. The crude white solid was resuspended in 100 mL of H_2O and heated to 100 °C, then filtered over a glass-fritted funnel to yield 4-chloro-3-ethyl-1-methyl-1H-pyrazole-5-carboxylic acid (820 mg, 4.35 mmol, 87.0 %) as a white solid.

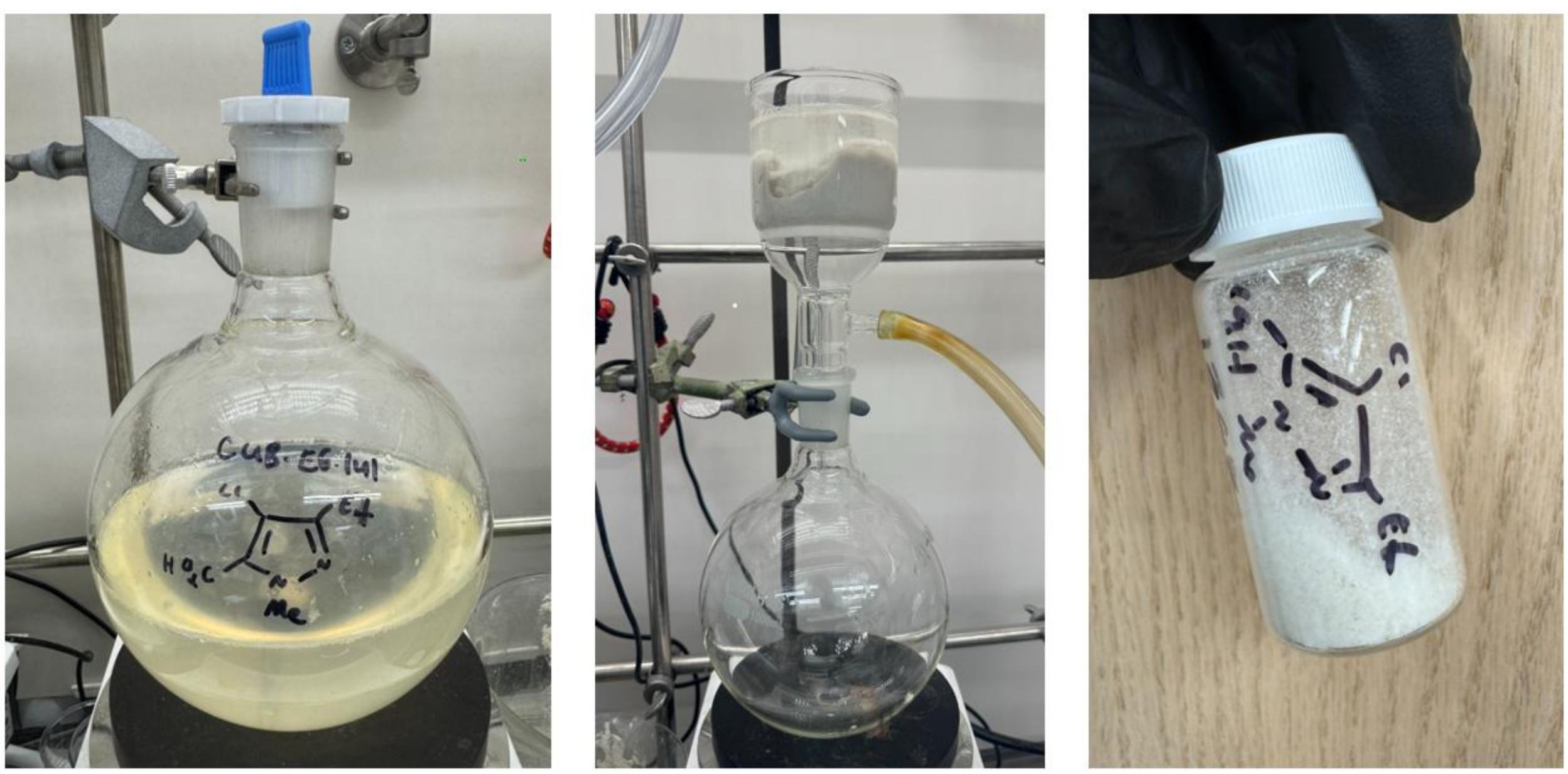

Images of crude reaction mixture, celite filtration to remove cellular debris, and final isolated product.

methyl 4-bromo-3-chloro-1H-pyrrole-2-carboxylate (64)

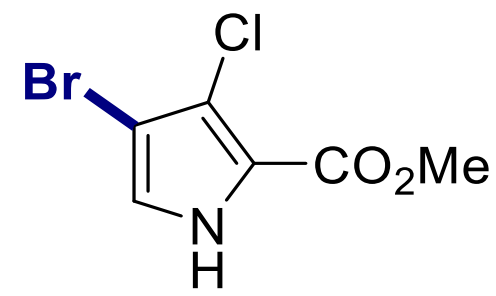

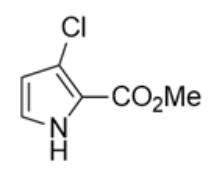 methyl 3-chloro-1*H*-pyrrole-2-carboxylate Exact Mass: 159.01	*Chlorination*				*Bromination*			
**LCAP**	***DMSO***	***DMF***	***MeCN***	***THF***	***DMSO***	***DMF***	***MeCN***	***THF***
Acetate 4	0	32	4	6	84	79	82	81
Acetate 5	0	11	7	12	79	81	80	**87**
Citrate 5	0	3	2	5	76	74	74	86
Citrate 6	0	0	0	2	34	29	33	43
MES 6	0	29	**33**	**50**	79	76	82	**91**
PIPES 6.5	0	23	28	**66**	69	62	76	**96**
TRIS 8	0	0	0	0	4	3	5	15
CAPS 10	0	0	0	0	1	1	1	4

Yield: 90%

Purification Method: Brine wash, then organic layer was concentrated in vacuo to dryness then resuspended in heptane (50 mL) and filtered over a fine glass-fritted funnel.

^{1}H NMR (400 MHz, $CDCl_3$) δ 9.42 (s, 1H), 6.97 (d, *J* = 3.5 Hz, 1H), 3.92 (s, 3H).

^{113}C NMR (101 MHz, $CDCl_3$) δ 160.20, 121.93, 119.64, 118.87, 100.21, 52.19.

NMR data was consistent with literature reports[19]

SM LCMS trace:

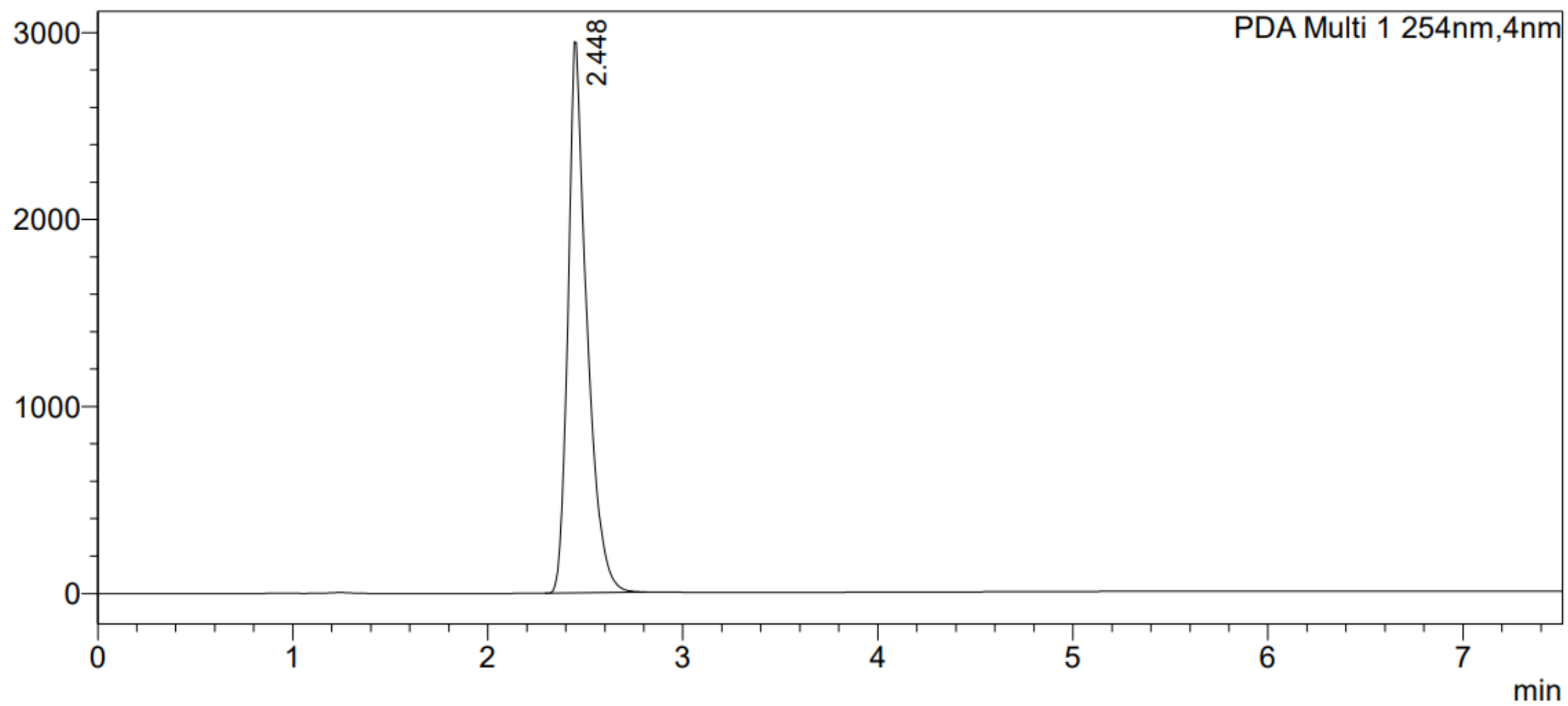

PIPES 6.5 / THF LCMS trace:

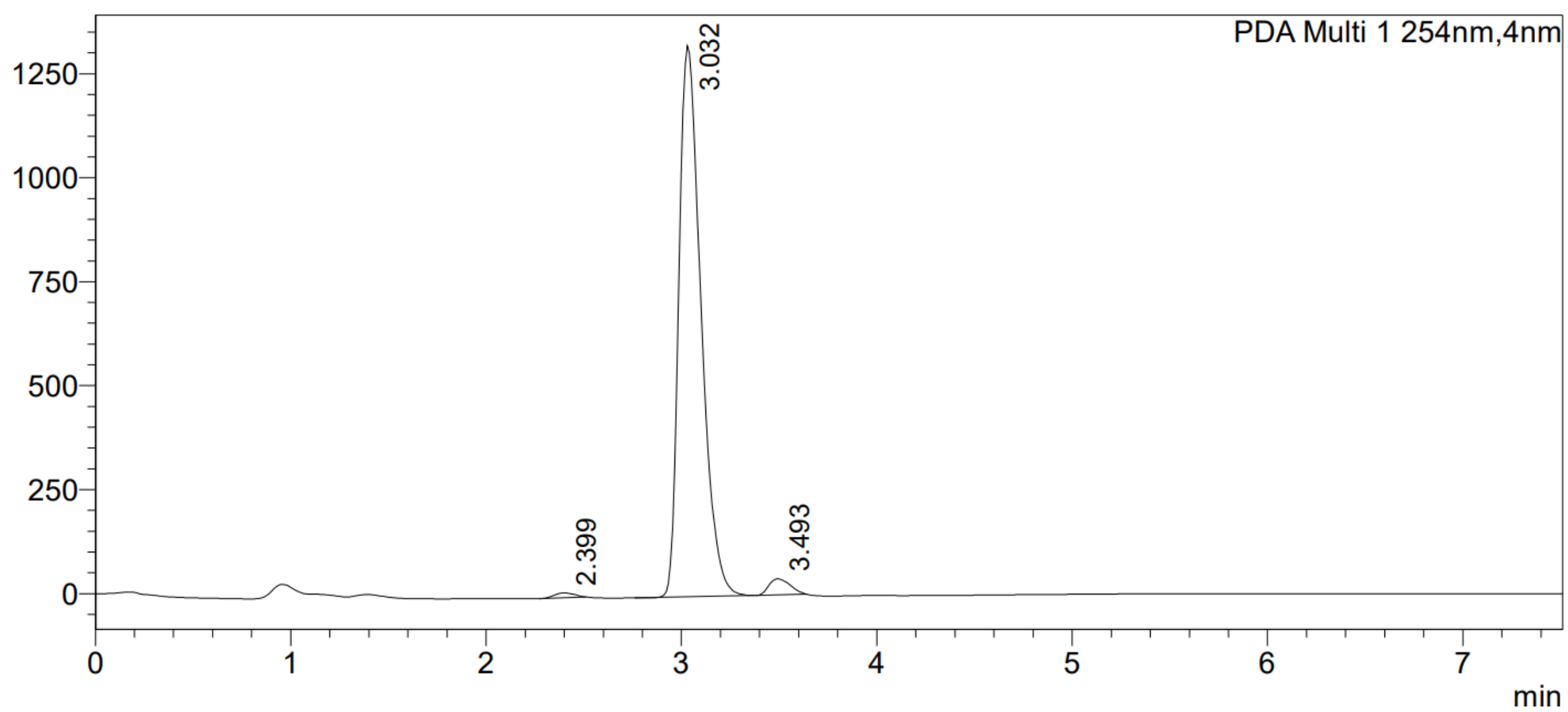

Note: the SM, product, and over-brominated product are not separable via TLC / column chromatography

Procedure for 5.0 mmol scale bromination with *Ci*VCPO clarified lysate:

A total of 6.25 mL of clarified *Ci*VCPO lysate was removed from a -80°C freezer and allowed to warm to room temperature over 5 min in a room temperature water bath. The combined thawed aliquots were then combined with an aqueous solution of 250 mM Na_3VO_4 (500µl total, 0.025 equiv) and stored at room temperature. To a 1L round bottom flask equipped with a magnetic stir-bar, purified H_2O (285 mL), THF (50.0 mL), 500mM PIPES (pH = 6.5, 50 mL) and KBr (55.0 mL, 100mmolar, 1.1 equiv) was added. The starting material (5.0 mmol, 798 mg, 1 equiv) was dissolved in THF (50 mL) and added to the reaction mixture. The *Ci*VCPO lysate (1.25% v/v) and Na_3VO_4 (0.025 equiv) solution was then added to the reaction mixture and stirred. A 10% stock of H_2O_2 (3.06 mL, 2.0 equiv) was then added to the reaction mixture and the resulting solution was stirred at 670 RPM at room temperature for 2 hours. An additional charge of H_2O_2 (3.06 mL, 2.0 equiv) was added after 2 hours, and stirred for an additional 2 hours. Upon completion the crude reaction mixture was filtered through a 2-inch thick pad of celite to remove cellular debris, and the filter pad was re- washed with additional 1:1 H_2O / THF (100 mL). The filtered reaction mixture was then then diluted with 300 mL of EtOAc. This biphasic solution was transferred to a 1L separatory funnel, the organic layer was collected, and the aqueous layer was re-extracted 3X with additional EtOAc (100 mL). The combined organic layer was then washed with saturated brine (250 mL), dried on sodium sulfate for 10 minutes, and concentrated under reduced pressure. The crude solid was resuspended in 200 mL of heptane and 5 mL of DCM and then filtered over a glass-fritted funnel. This process was repeated 3 times, yielding methyl 4-bromo-3-chloro-1H-pyrrole-2-carboxylate (1.08 g, 4.53 mmol, 90.6 %) as a tan solid.

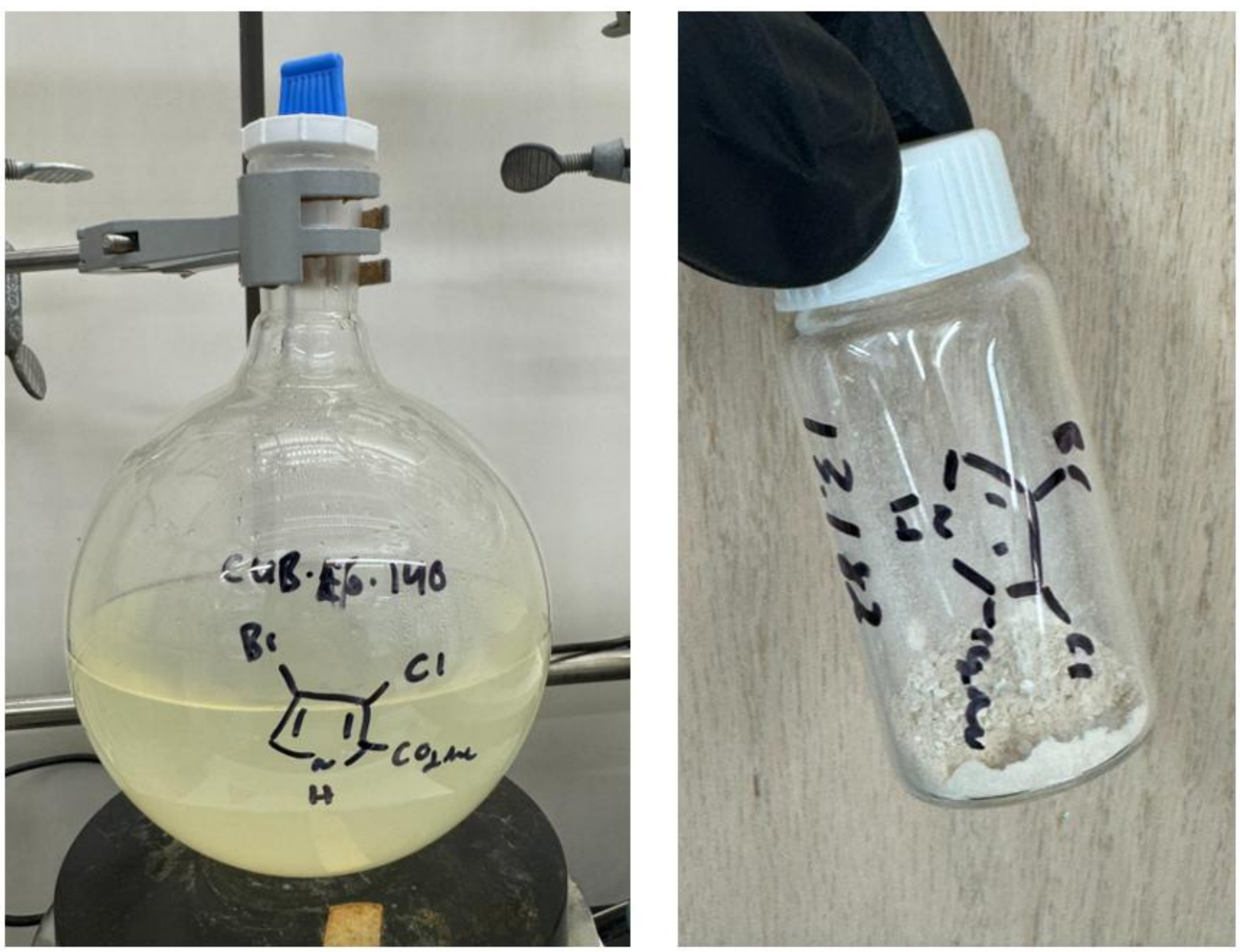

Images of crude reaction mixture and final isolated product.

General Procedure for Preparative Scale Indazole N-Chlorination:

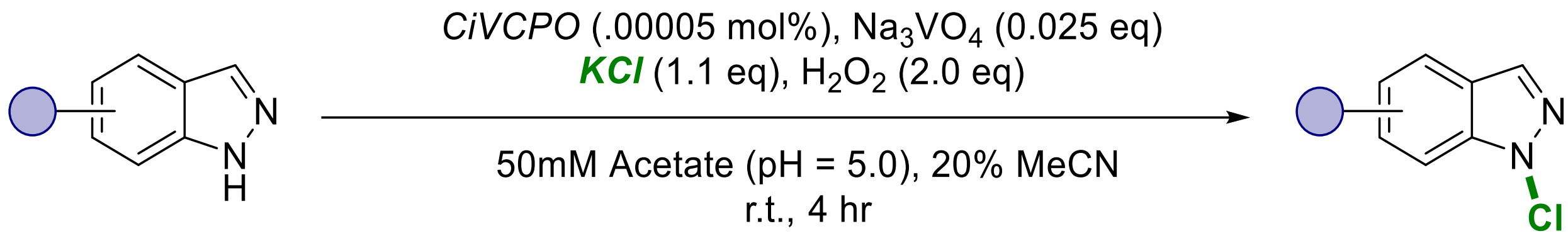

Two enzyme aliquots of purified VHPO from *Curvularia inaequalis* (*Ci*VCPO, 10 µM, 1.0 mL) were removed from a -80°C freezer and allowed to warm to room temperature over 5 min. The thawed aliquot was then combined with an aqueous solution of 250 mM Na_3VO_4 (160 µL, 0.1 equiv) in a microcentrifuge tube and stored at room temperature. To ensure mixing, the resulting solution was spun in the mini centrifuge for 10 seconds and placed on the benchtop until further use. To a 100 mL round bottom flask containing a magnetic stir bar, purified H_2O (21 mL), MeCN (4.0 mL), 4.0 mL 500mM Acetate (pH = 5.0) and 100mM KCl (4.4 mL, 1.1 equiv) was added. The starting material (0.40 mmol, 1 equiv) was dissolved in MeCN (4.0 mL) and then added to the reaction mixture. The microcentrifuge tubes containing the *Ci*VCPO (0.00005 mol%, 2.0 mL total volume) and Na_3VO_4 (0.1 equiv) were then added to the reaction mixture. A 10% stock of H_2O_2 (264 µL, 2 equiv) was then added to the reaction mixture and the resulting solution was allowed to stir at room temperature for 16h. The reaction mixture was then diluted with EtOAc (40 mL) and transferred to a 125 mL separatory funnel. The organic layer was collected, and the aqueous layer was re-extracted 3X with additional EtOAc (20 mL). The combined organic layer was then washed with saturated brine (50mL), dried on sodium sulfate for 10 minutes, concentrated under reduced pressure, and further purified via flash chromatography.

Characterization Data for N-Chlorinated Indazoles

1-chloro-1H-indazole (67)

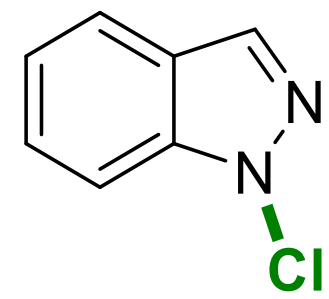

Purification Method**:** Aqueous wash only. Isolation of this compound was unsuccessful as it is unstable on silica gel and rapidly degrades to the corresponding indazole and C3-chlorinated indazole. The following characterization data is solely to support the proposal of this as the major product of the enzymatic reaction.

^{1}H NMR (400 MHz, DMSO) δ 8.06 (d, *J* = 1.0 Hz, 1H), 7.75 (dt, *J* = 8.1, 1.0 Hz, 1H), 7.54 (dq, *J* = 8.4, 1.0 Hz, 1H), 7.33 (ddd, *J* = 8.4, 6.8, 1.1 Hz, 1H), 7.09 (ddd, *J* = 7.8, 6.8, 0.9 Hz, 1H).

^{13}C NMR (101 MHz, DMSO) δ 139.92, 133.34, 125.92, 122.80, 120.51, 120.21, 110.15.

96-well plate optimization data:

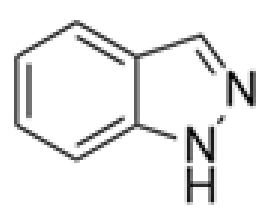 1*H*-indazole<br>Exact Mass: 118.05	***Chlorination***				***Bromination***			
**LCAP**	***DMSO***	***DMF***	***MeCN***	***THF***	***DMSO***	***DMF***	***MeCN***	***THF***
Acetate 4	0	65	40	41	0	0	0	0
Acetate 5	0	**71**	**72**	**67**	0	0	0	0
Citrate 5	0	47	46	41	0	0	0	0
Citrate 6	0	2	26	20	0	0	0	0
MES 6	0	0	0	0	0	0	0	0
PIPES 6.5	0	0	0	0	0	0	0	0
TRIS 8	0	0	0	0	0	0	0	0
CAPS 10	0	0	0	0	0	0	0	0

LCMS Comparison of SM, N-Cl, and C3-Cl:

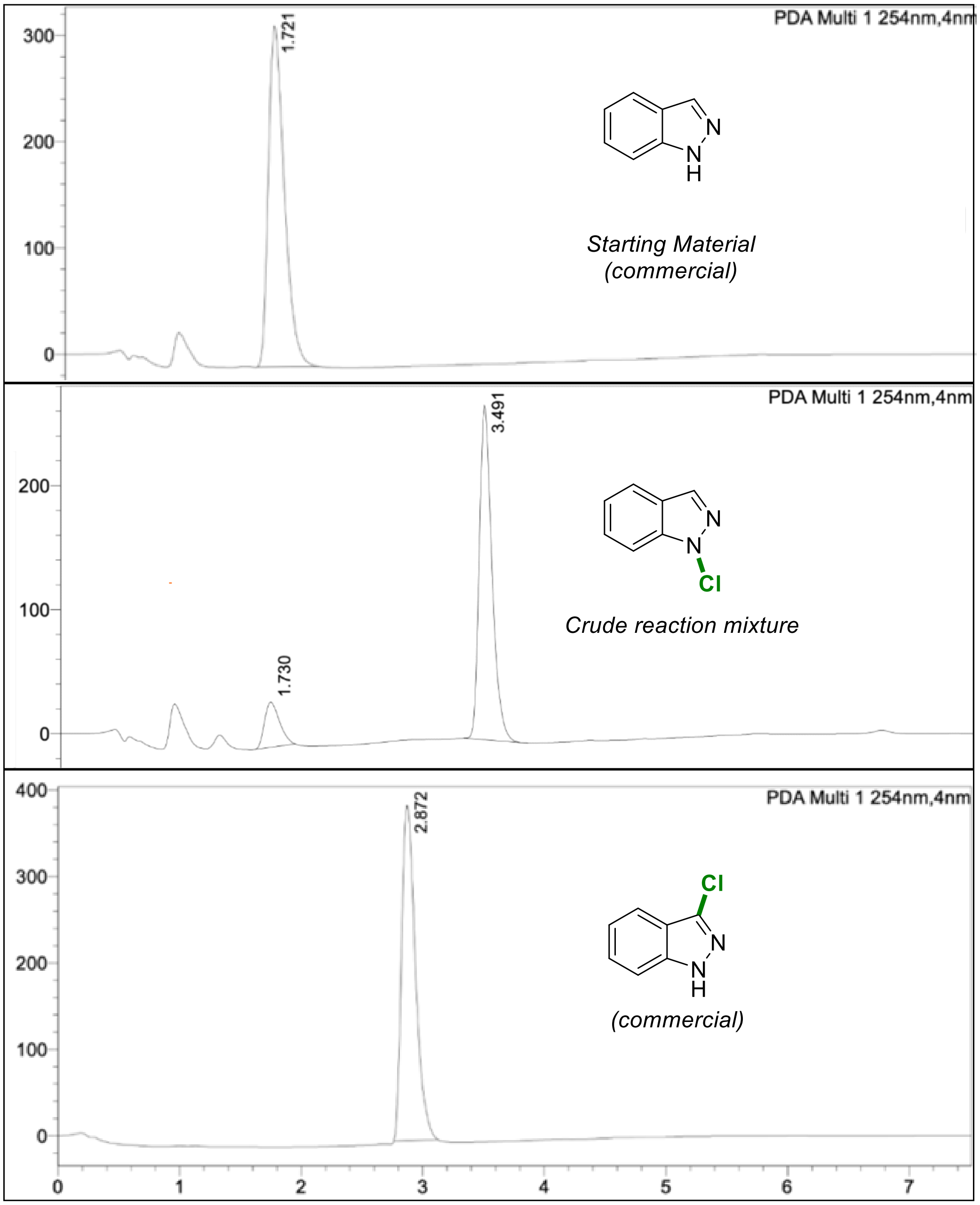

Methyl 1-chloro-1H-indazole-6-carboxylate (68)

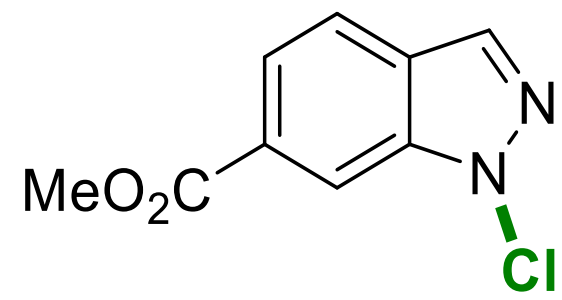

Yield: 88%

Purification Method: Flash column chromatography (10% EtOAc / Hexanes)

^{1}H NMR (400 MHz, $CDCl_3$) δ 8.23 (q, *J* = 1.0 Hz, 1H), 8.12 (d, *J* = 1.2 Hz, 1H), 7.90 (dd, *J* = 8.4, 1.3 Hz, 1H), 7.77 (dd, *J* = 8.5, 0.8 Hz, 1H), 3.99 (s, 3H).

^{13}C NMR (151 MHz, $CDCl_3$) δ 166.74, 142.28, 135.69, 129.98, 126.49, 122.97, 121.43, 112.07, 52.68.

HRMS: calculated $C_9H_8O_2N_2{}^{35}Cl$ [M+H]+: 211.0268. Found [M+H]+: 211.0268

4-bromo-1-chloro-1H-indazole (69)

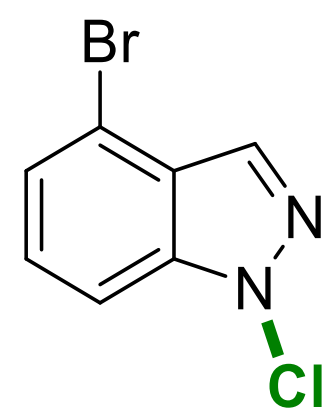

Yield: 49%

Purification Method: Flash column chromatography (10% EtOAc / Hexanes)

^{1}H NMR (400 MHz, $CDCl_3$) δ 8.07 (d, *J* = 1.1 Hz, 1H), 7.45 (dt, *J* = 8.1, 1.1 Hz, 1H), 7.41 – 7.32 (m, 2H).

^{13}C NMR (151 MHz, $CDCl_3$) δ 143.23, 135.69, 128.95, 125.31, 124.95, 115.02, 108.99.

HRMS: calculated $C_7H_5N_2{}^{79}Br^{35}Cl$ [M+H]+: 230.9319. Found [M+H]+: 230.9321

3-bromo-1-chloro-1H-indazole (70)

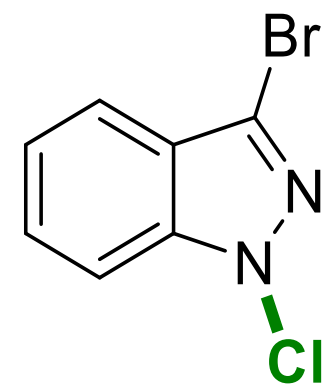

Yield: 38%

Purification Method: Flash column chromatography (10% EtOAc / Hexanes)

^{1}H NMR (400 MHz, $CDCl_3$) δ 7.63 (dt, J = 8.2, 0.9 Hz, 1H), 7.56 (ddd, J = 8.1, 6.9, 1.1 Hz, 1H), 7.49 (dt, J = 8.5, 0.9 Hz, 1H), 7.31 (ddd, J = 8.0, 6.9, 1.0 Hz, 1H).

^{13}C NMR (101 MHz, $CDCl_3$) δ 144.78, 129.45, 124.69, 123.50, 123.27, 120.86, 110.36.

HRMS: calculated $C_7H_5N_2{}^{79}Br^{35}Cl$ [M+H]+: 230.9243. Found [M+H]+: 230.9248

1-chloro-3-iodo-1H-indazole (71)

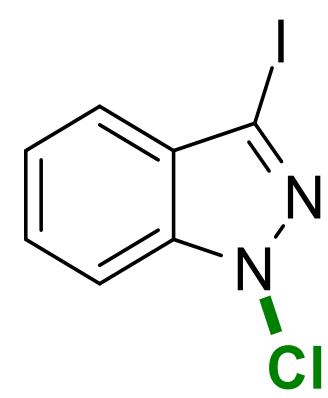

Yield: 31%

Purification Method: Flash column chromatography (10% EtOAc / Hexanes)

^{1}H NMR (400 MHz, $CDCl_3$) δ 7.49 (ddd, *J* = 8.2, 6.9, 1.1 Hz, 1H), 7.41 (ddd, *J* = 7.7, 2.1, 1.2 Hz, 2H), 7.23 (ddd, *J* = 8.0, 6.9, 0.9 Hz, 1H).

^{13}C NMR (151 MHz, $CDCl_3$) δ 143.80, 129.38, 129.02, 123.21, 122.22, 110.15, 94.12.

HRMS: calculated $C_7H_4N_2{}^{127}I^{35}Cl$ [M+H]+: 278.9167. Found [M+H]+: 278.9176

General Procedure for Decarboxylative Halogenation:

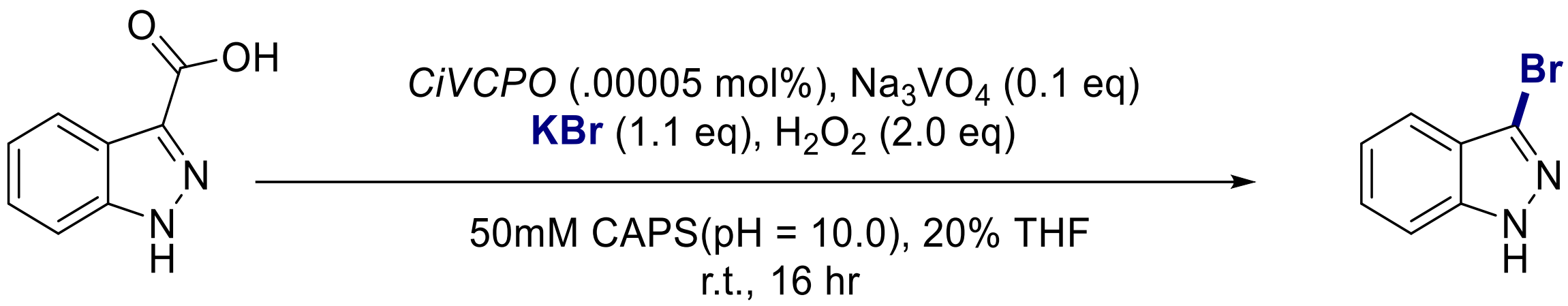

A 500 µl aliquot of purified VHPO from *Curvularia inaequalis* (*Ci*VCPO, 10 µM) was removed from a -80°C freezer and allowed to warm to room temperature over 5 min. The thawed aliquot was then combined with an aqueous solution of 250 mM Na_3VO_4 (40 µL, 0.1 equiv) in a microcentrifuge tube and stored at room temperature. To ensure mixing, the resulting solution was spun in the mini centrifuge for 10 seconds and placed on the benchtop until further use. To a 20mL scintillation vial containing a magnetic stir bar, purified H_2O (5.3 mL), THF (1.0 mL), 500mM Citrate (pH = 5.0, 1.0 mL) and 100mM KCl (1.1 mL, 1.1 equiv) was added. The starting material (0.10 mmol, 1 equiv) was dissolved in THF (1.0 mL) and added to the reaction mixture. The microcentrifuge tubes containing the *Ci*VCPO (0.00005 mol%, 2.0 mL total volume) and Na_3VO_4 (0.1 equiv) were then added to the reaction mixture. A 10% stock of H_2O_2 (62 µL, 2 equiv) was then added to the reaction mixture and the resulting solution was allowed to stir at room temperature for 16h. The reaction mixture was then diluted with EtOAc (5 mL) and transferred to a 125 mL separatory funnel. The organic layer was collected, and the aqueous layer was re-extracted 3X with additional EtOAc (20 mL). The combined organic layer was then washed with saturated brine (50 mL), dried on sodium sulfate for 10 minutes, and concentrated under reduced pressure yielding 3-bromo-1H-indazole (11 mg, 56% yield).

3-bromo-1H-indazole (72)

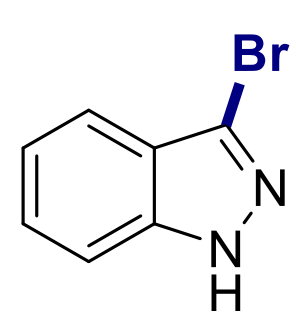

Yield: 56%

Purification Method: Aqueous wash only, no chromatography

^{1}H NMR (400 MHz, DMSO) δ 13.44 (s, 1H), 7.61 – 7.54 (m, 2H), 7.45 (ddd, J = 8.1, 6.8, 1.1 Hz, 1H), 7.26 – 7.18 (m, 1H).

^{13}C NMR (101 MHz, DMSO) δ 140.97, 127.49, 122.08, 121.48, 120.39, 119.18, 110.85.

NMR data was consistent with literature reports[20]

NMR data for other decarboxylative halogenations were consistent with data from direct C-H halogenation

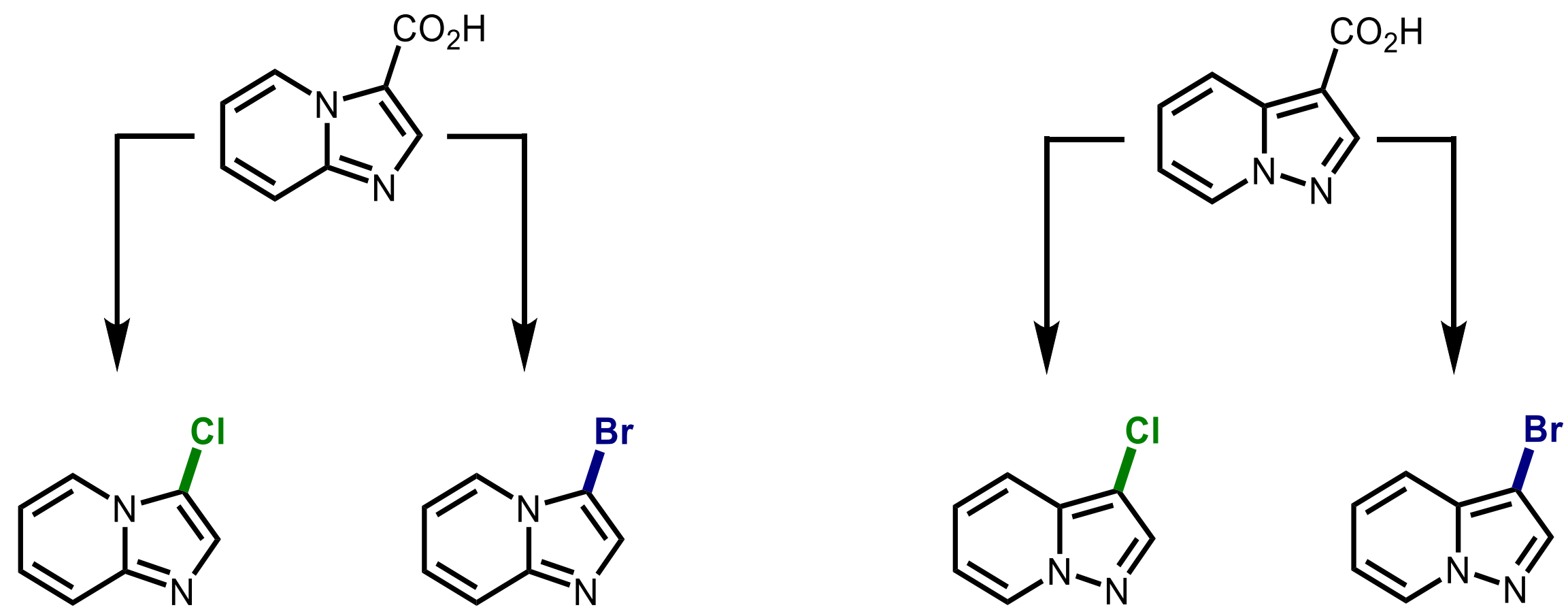

33
79% yield
(PIPES 6.5, 20% THF)

34
86% yield
(CAPS 10, 20% THF)

41
59% yield
(MES 6.0, 20% THF)

42
76% yield
(MES 6.0, 20% THF)

Ethyl 1-chloroimidazo[1,5-a]pyridine-3-carboxylate (73)

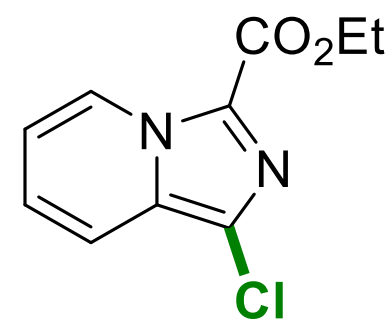

*This chloro-imidazopyridine was prepared chemically to probe the mechanism of the Ci*VCPO *catalyzed conversion of* ***58*** *to* ***60****.*

An oven dried 20   scintillation vial was charged with ethyl-imidazo[1,5-a]pyridine-3-carboxylate (380 mg, 20.0 mL, 100 mmolar, 1 Eq, 2.00 mmol) and dissolved in MeCN (10.0 mL). NCS (280 mg, 1.05 Eq, 2.10 mmol) was then added in one portion at r.t. causing the solution to quickly form a dark green color. The reaction was vigorously stirred for 24 hours, then quenched with water (5 mL). This mixture was transferred to a separatory funnel and further diluted with 10 mL EtOAc, and the resulting organic layer was further washed with water (10 mL) and brine (10 mL), then dried on sodium sulfates. The crude material was concentrated in vacuo, and purified via flash column chromatography (0--> 50% EtOAc) to yield ethyl 1-chloroimidazo[1,5-a]pyridine-3-carboxylate (315 mg, 1.40 mmol, 70.1 %) as a fine, pale green solid.

Yield: 70%

Purification Method: Flash column chromatography, 0 → 50% EtOAc

^{1}H NMR (600 MHz, $CDCl_3$) δ 9.30 (dt, *J* = 7.2, 1.1 Hz, 1H), 7.61 (dt, *J* = 9.1, 1.2 Hz, 1H), 7.12 (ddd, *J* = 9.1, 6.6, 1.0 Hz, 1H), 6.96 (ddd, *J* = 7.2, 6.6, 1.3 Hz, 1H), 4.48 (q, *J* = 7.1 Hz, 2H), 1.45 (t, *J* = 7.1 Hz, 3H).

^{13}C NMR (151 MHz, $CDCl_3$) δ 159.08, 129.94, 125.72, 124.56, 123.22, 121.90, 117.26, 116.47, 61.59, 14.57.

Computational Details:

CREST 3.0.1[21]/XTB 6.7.0[22] was used to generate conformers for each molecule at the GFN2 level[23] and filtered using the MD screen process at a 5.0 kcal/mol energy window. Each conformer was then DFT-optimized using Gaussian16 at the PBE0[24]-D3(BJ) [25]/def2-SVP[26] level of theory, then single point energy calculations were conducted at the PBE0-D3(BJ)/def2-TZVP level of theory. Properties were extracted,[27] including:

- Global parameters
  - HOMO energy
  - LUMO energy
  - μ (chemical potential, HOMO+LUMO)
  - η (hardness, HOMO–LUMO)
  - ω (electrophilicity index, $\mu^2/2*\eta$)
  - Isotropic and anisotropic polarizability
  - Dipole
  - Molar volume
  - Solvent accessible surface area (sphericity, area, volume)
- Atomic properties for halogenated carbon
  - NBO charge[28]
  - Isotropic NMR shielding
  - $\%V_{bur}$ (2–5 Å in 0.5 Å intervals)[29]
  - Pyramidalization
  - ChelpG charge[30]
  - Hirshfeld charge[31]

Parameters were also obtained from Rowan[32] ($pKaH^+$) or DeepSynthesis (H_2O, $pKaH^+$, HBD, HBA)[33].

Two-parameter thresholds were identified based on accuracy and precision. A number of thresholds with similar performances were identified, in which case we focused on models with increased differentiation of points and further division beyond low and high pH. The code used to generate these models can be found on the Sigman group GitHub at https://github.com/SigmanGroup/python-modeling. The initial substrate scope was divided into training and validation in an 80:20 ratio using a Kennard-Stone algorithm. Further, we chose an external test set based on chemical interest, identifying five-membered azoles that were significantly different in stereoelectronics from the initial substrate scope and were commercially available.

For chlorination, a threshold of 41% $\%V_{bur}$ (4Å) and 0.232 eV HOMO (Boltzmann averages) was identified, and for bromination a threshold of 43% $\%V_{bur}$ (4Å) and 5.3 pK_{aH}

(Boltzmann averages). Since the $\%V_{bur}$ was colinear across all radii, we selected 4.0 Å as this gave the best separation between data points.

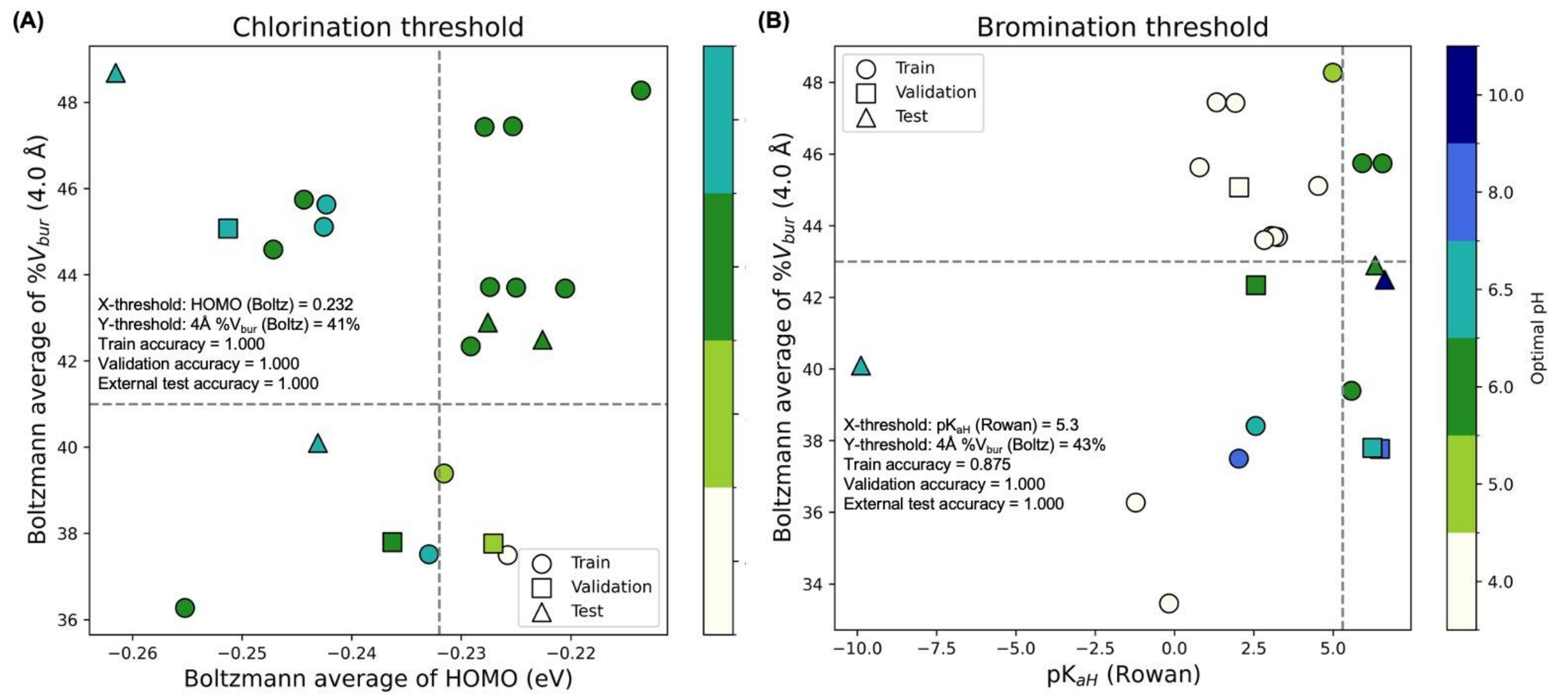

Figure S9. Prediction Model for Halogenation with *Ci*VCPO. (A) Threshold model of optimal pH for chlorination; (B) threshold model of optimal pH for bromination.

We also identified several alternative models for both chlorination and bromination. While these are not identified as best models, they are interesting to consider.

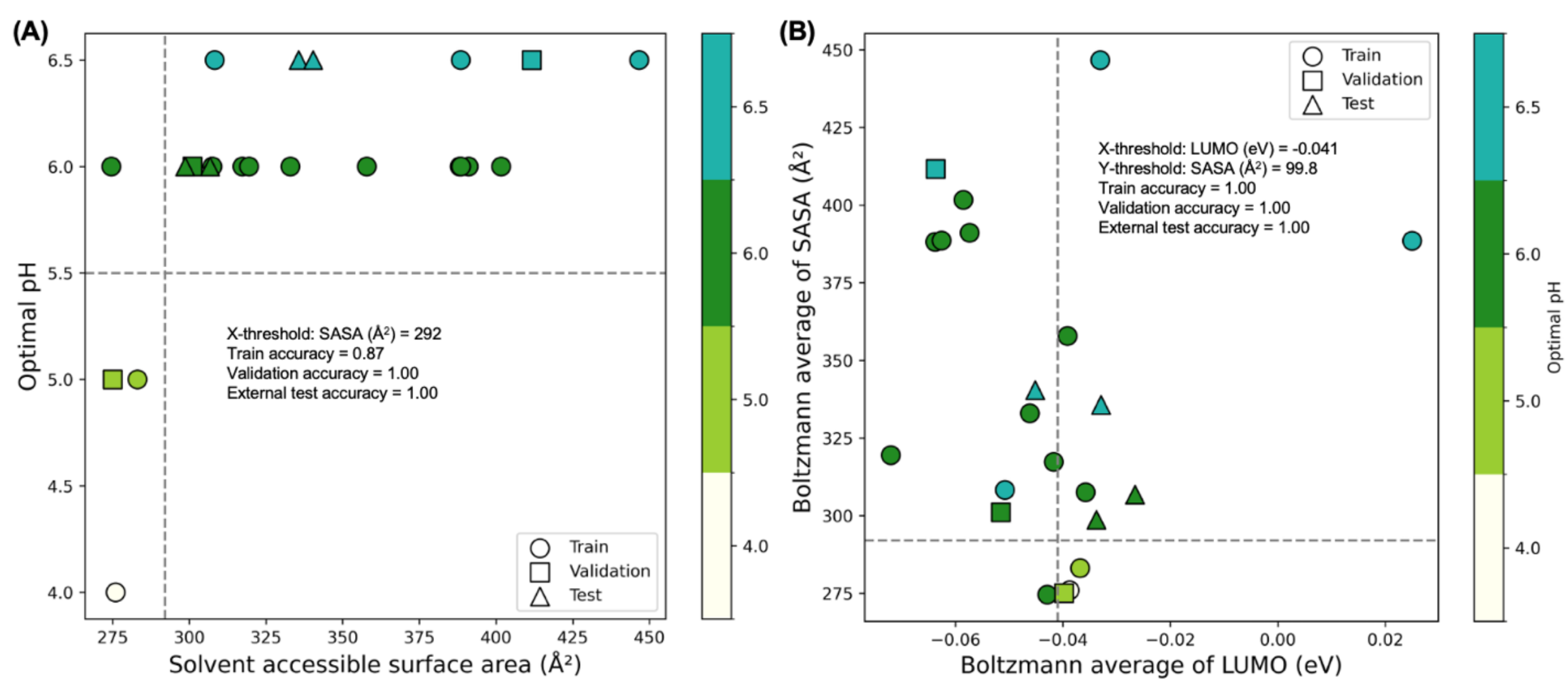

Figure S10. Alternate chlorination models. (A) Single-node threshold for optimal pH with Boltzmann average of SASA ($Å^2$). (B) Double-node threshold for optimal pH with Boltzmann averages of SASA ($Å^2$) and LUMO (eV).

A fairly accurate single-node model can be obtained using the Boltzmann average of SASA ($Å^2$). This feature may be suggestive of interactions between the enzyme active site and substrate.

However, the single-node threshold fails to divide substrates past low and high optimal pH, so we focused our efforts on double thresholds. An alternative double threshold featured the same Boltzmann average of SASA (Å^2), in addition to the Boltzmann average of the LUMO (eV). While this model also has perfect accuracy, the spread of data points is less optimal than the chosen best model, and differentiation between the optimal pH's of 6.0 and 6.5 is more challenging.

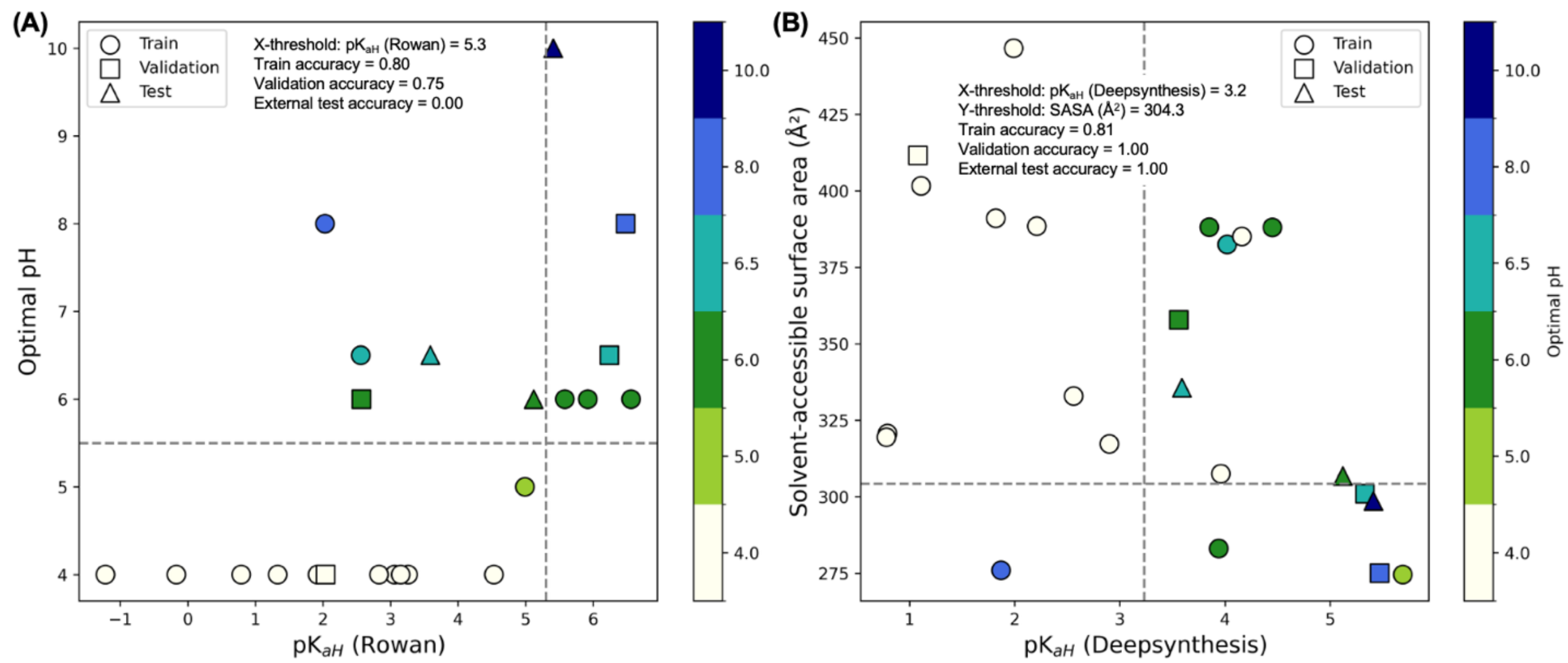

Figure S11. Alternate bromination models. (A) Single-node threshold with pK$_{aH}$ (Rowan); (B) Double-node threshold with pK$_{aH}$ (Deepsynthesis) and Boltzmann average of SASA (Å^2).

We were again able to identify a reasonably performing single-node threshold, though accuracy is much lower in this case. Here, pK$_{aH}$ (Rowan) predicts that substrates with lower pK$_{aH}$'s perform better under lower pH conditions. However, the performance of this models was not satisfactory, leading us to believe that while pK$_{aH}$ is important for optimal reaction pH, it is not the only factor. In expanding to examine other double thresholds, we again found that the Boltzmann average of SASA (Å^2) was important, as was pK$_{aH}$ (though Deepsynthesis this time). This may suggest an interplay of pK$_{aH}$ and enzyme active site-substrate interactions leading to optimal pH. However, this model did not differentiate between high optimal pH substrates as well, and thus was not chosen as the best model.

References

(1) Gross, E. J.; Barthel, S. G.; Brzezinski, C. U.; Hessefort, L. Z.; Bacsa, J.; Biegasiewicz, K. F. Chlorination of Amines by a Vanadium-Dependent Chloroperoxidase. ACS Catal. 2026, 16 (7), 6241–6246. https://doi.org/10.1021/acscatal.6c00816.

(2) Cheung, M.; Boloor, A.; Stafford, J. A. Efficient and Regioselective Synthesis of 2-Alkyl-2H-Indazoles. J. Org. Chem. 2003, 68 (10), 4093–4095. https://doi.org/10.1021/jo0265434.

(3) Treitler, D. S.; Leung, S. How Dangerous Is Too Dangerous? A Perspective on Azide Chemistry. J. Org. Chem. 2022, 87 (17), 11293–11295. https://doi.org/10.1021/acs.joc.2c01402.

(4) Nykaza, T. V.; Harrison, T. S.; Ghosh, A.; Putnik, R. A.; Radosevich, A. T. A Biphilic Phosphetane Catalyzes N–N Bond-Forming Cadogan Heterocyclization via PIII/PV═O Redox Cycling. J. Am. Chem. Soc. 2017, 139 (20), 6839–6842. https://doi.org/10.1021/jacs.7b03260.

(5) Singsardar, M.; Laru, S.; Mondal, S.; Hajra, A. Visible-Light-Induced Regioselective Cross-Dehydrogenative Coupling of 2H-Indazoles with Ethers. J. Org. Chem. 2019, 84 (7), 4543–4550. https://doi.org/10.1021/acs.joc.9b00318.

(6) Aranzazu, S.-L.; Tigreros, A.; Arias-Gómez, A.; Zapata-Rivera, J.; Portilla, J. BF3-Mediated Acetylation of Pyrazolo[1,5-a]Pyrimidines and Other π-Excedent (N-Hetero)Arenes. J. Org. Chem. 2022, 87 (15), 9839–9850. https://doi.org/10.1021/acs.joc.2c00881.

(7) Liu, X.; Wu, Z.; Feng, C.; Liu, W.; Li, M.; Shen, Z. Catalyst- and Oxidant-Free Electrochemical Halogenation Reactions of 2H-Indazoles with NaX (X=Cl, Br). European Journal of Organic Chemistry 2022, 2022 (17), e202200262. https://doi.org/10.1002/ejoc.202200262.

(8) Kalyani, D.; Dick, A. R.; Anani, W. Q.; Sanford, M. S. Scope and Selectivity in Palladium-Catalyzed Directed C–H Bond Halogenation Reactions. Tetrahedron 2006, 62 (49), 11483–11498. https://doi.org/10.1016/j.tet.2006.06.075.

(9) Targos, K.; Williams, O. P.; Wickens, Z. K. Unveiling Potent Photooxidation Behavior of Catalytic Photoreductants. J. Am. Chem. Soc. 2021, 143 (11), 4125–4132. https://doi.org/10.1021/jacs.1c00399.

(10) Xie, Y.; Lin, L.; Xu, B. A Continuous Flow Electrochemical Reactor Using Readily Available Metal Wires and Carbon Fibers as Electrodes: Environmentally Benign Halogenations of Alkenes, Alkynes, and Aromatics. Green Chem. 2024, 26 (9), 5173–5177. https://doi.org/10.1039/D4GC01503G.

(11) Beveridge, R. E.; Fernando, D.; Gerstenberger, B. S. One-Pot Copper-Catalyzed Synthesis of N-Functionalized Pyrazoles from Boronic Acids. Tetrahedron Letters 2010, 51 (38), 5005–5008. https://doi.org/10.1016/j.tetlet.2010.07.077.

(12) Kour, J.; Khajuria, P.; Sharma, A.; Sawant, S. D. TBAX/Oxone-Mediated Halogenation of Pyrazoles and Other Heterocycles: An Entry to Important Cross-Coupling Reactions. Chemistry – An Asian Journal 2022, 17 (22), e202200778. https://doi.org/10.1002/asia.202200778.

(13) Li, J.; Tang, J.; Wu, Y.; He, Q.; Yu, Y. Transition-Metal-Free Regioselective C–H Halogenation of Imidazo[1,2-a]Pyridines: Sodium Chlorite/Bromite as the Halogen Source. RSC Adv. 2018, 8 (9), 5058–5062. https://doi.org/10.1039/C7RA12100H.

(14) Song, S.; Sun, X.; Li, X.; Yuan, Y.; Jiao, N. Efficient and Practical Oxidative Bromination and Iodination of Arenes and Heteroarenes with DMSO and Hydrogen Halide: A Mild Protocol for Late-Stage Functionalization. Org. Lett. 2015, 17 (12), 2886–2889. https://doi.org/10.1021/acs.orglett.5b00932.

(15) Duan, Y.; Luo, S. Phase-Transfer Catalysis for Electrochemical Chlorination and Nitration of Arenes. Angewandte Chemie International Edition 2024, 63 (17), e202319206. https://doi.org/10.1002/anie.202319206.

(16) Rostrup, F.; Falk-Petersen, C. B.; Harpsøe, K.; Buchleithner, S.; Conforti, I.; Jung, S.; Gloriam, D. E.; Schirmeister, T.; Wellendorph, P.; Frølund, B. Structural Determinants for the Mode of Action of Imidazopyridine DS2 at δ-Containing γ-Aminobutyric Acid Type A Receptors. J. Med. Chem. 2021, 64 (8), 4730–4743. https://doi.org/10.1021/acs.jmedchem.0c02163.

(17) Chillal, A. S.; Bhawale, R. T.; Kshirsagar, U. A. Regioselective C(Sp2)–H Halogenation of Pyrazolo[1,5-a]Pyrimidines Facilitated by Hypervalent Iodine(III) under Aqueous and Ambient Conditions. RSC Adv. 2024, 14 (19), 13095–13099. https://doi.org/10.1039/D4RA02090A.

(18) Nageswara Rao, S.; Reddy, N. N. K.; Samanta, S.; Adimurthy, S. I2 -Catalyzed Oxidative Amidation of Benzylamines and Benzyl Cyanides under Mild Conditions. J. Org. Chem. 2017, 82 (24), 13632–13642. https://doi.org/10.1021/acs.joc.7b02211.

(19) Bhattasali, D.; Neithnadka, P. R.; Dhineshkumar, J.; Kuppusamy, P.; Dhanukoti, S.; Reddy, T.; Rao, P.; Lakshminarasimhan, T.; Qin, L.-Y.; Fink, B.; Mathur, A.; Gupta, A. A Practical and Scalable Route to 6-Bromo-2,4,5-Trichloropyrrolo[2,1-f][1,2,4]Triazine: Process Optimization Guided by Thermal Safety Insights. Org. Process Res. Dev. 2026, 30 (4), 880–891. https://doi.org/10.1021/acs.oprd.5c00446.

(20) Qasid, M.; Pfeiffer, M. A.; Jeffries, C. J.; Hornbrook, L. F.; Purser, G. H.; Lamar, A. A. Organocatalytic Halogenation of Indazoles and Pyrazoles with N-Halosuccinimides Enhanced by Gallocyanine as Halogen-Transfer Agent. J. Org. Chem. 2025, 90 (37), 13103–13115. https://doi.org/10.1021/acs.joc.5c01711.

(21) Pracht, P; Grimme, S.; Bannwarth, C.; Bohle, F.; Ehlert, S; *et. al.* CREST—A program for the exploration of low-energy molecular chemical space. *The Journal of Chemical Physics* **2024**, *160*, 114110. DOI: 10.1063/5.0197592.

(22) Bannwarth, C.; Caldehweyher, E.; Ehlert, S.; Hansen, A.; Pracht, P.; Seibert, J.; Spicher, S.; Grimme, S. Extended tight-binding quantum chemistry methods. *WIREs Computational Molecular Science* **2020**, *11* (2), e1493. DOI: 10.1002/wcms.1493

(23) Bannwarth, C.; Ehlert, S.; Grimme, S. GFN2-xTB—An Accurate and Broadly Parametrized Self-Consistent Tight-Binding Quantum Chemical Method with Multipole Electrostatics and Density-Dependent Dispersion Contributions. *Journal of Chemical Theory and Computation* **2019**, *15* (3), 1652–1671. DOI: 10.1021/acs.jctc.8b01176.

(24) Adamo, C.; Barone, V. Toward reliable density functional methods without adjustable parameters: The PBE0 model. *The Journal of Chemical Physics* **1999**, *110* (13), 6158–6170. DOI: 10.1063/1.478522.

(25) Grimme, S.; Ehrlich, S.; Goerigk, L. Effect of the damping function in dispersion corrected density functional theory. *Journal of Computational Chemistry* **2011**, *32* (7), 1456–1465. DOI: 10.1002/jcc.21759.

(26) Jensen, F. Atomic orbital basis sets. *WIREs Computational Molecular Science* **2013**, *3* (3), 273–295. DOI: 10.1002/wcms.1123.

(27) Howard, J.; Haas, B.; Hardy, M. *GetProperties v1.0.1.* Zenodo, 2026. DOI: 10.5281/zenodo.10651726

(28) Lu, T.; Chen, F. Multiwfn: A multifunctional wavefunction analyzer. *Journal of Computational Chemistry* **2012**, *33* (5), 580–592. DOI: 10.1002/jcc.22885.

(29) MORFEUS. https://digital-chemistry-laboratory.github.io/morfeus/index.htmL (accessed September 19, 2024).

(30) Breneman, C. M.; Wiberg, K. B. Determining atom-centered monopoles from molecular electrostatic potentials. The need for high sampling density in formamide conformational analysis. *Journal of Computational Chemistry* **1990**, *11* (3), 361–373. DOI: 10.1002/jcc.540110311.

(31) Marenich, A. V.; Jerome, S. V.; Cramer, C. J.; Truhlar, D. G. Charge Model 5: An Extension of Hirshfeld Population Analysis for the Accurate Description of Molecular Interactions in Gaseous and Condensed Phases. *Journal of Chemical Theory and Computation* **2012**, *8* (2), 527–541. DOI: 10.1021/ct200866d.

(32) *Rowan Scientific*. https://www.rowansci.com (accessed 20206-04-09)

(33) Liu, S., Yang, Q., Zhang, L., Luo, S. Highly Precise Prediction of Micro- and Supra-pKa Based on 3D Descriptors Integrating Non-Covalent Interactions. *Angewandte Chemie International Edition* **2025**, *64* (16), e202424069. DOI: 10.1002/anie.202424069

Spectral Data

Starting Material Spectra:

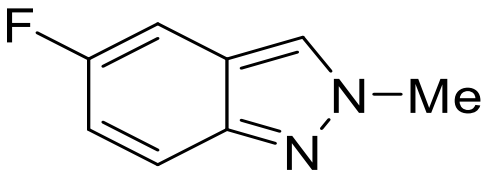

5-fluoro-2-methyl-2H-indazole (SM-4/5)

^{1}H-NMR: $CDCl_3$

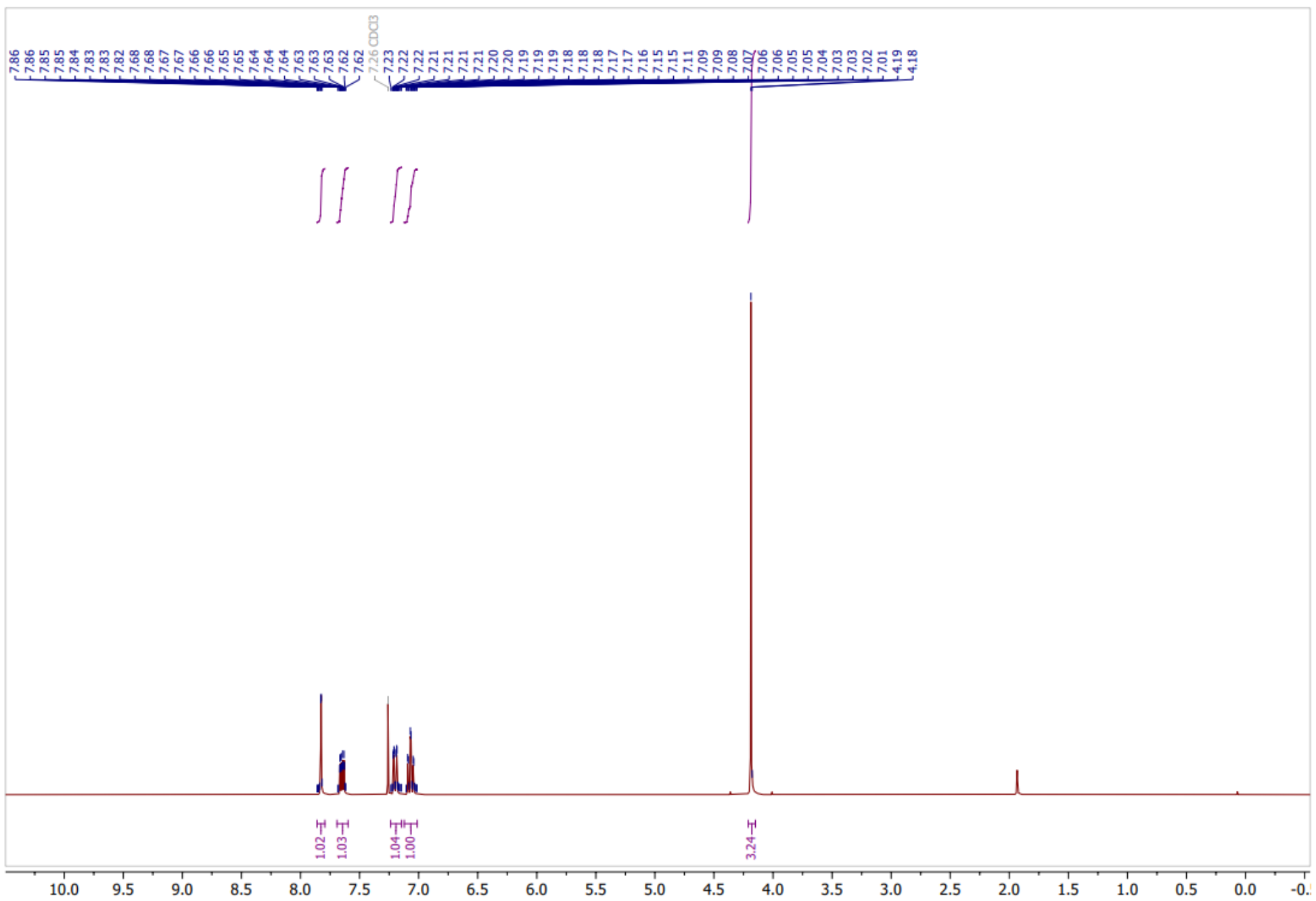

^{13}C-NMR: $CDCl_3$

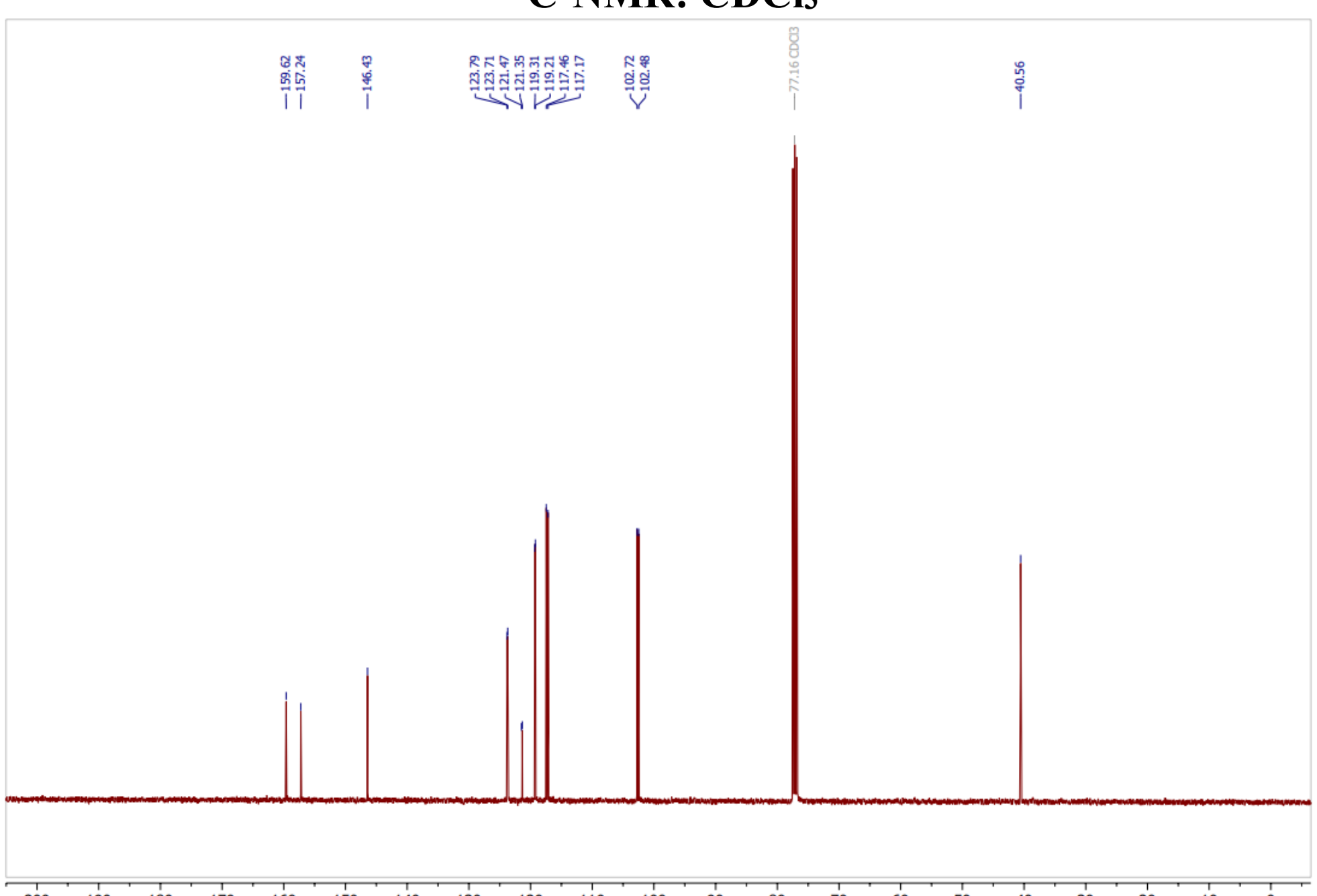

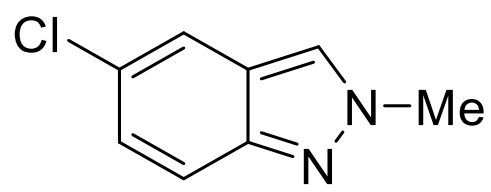

5-chloro-2-methyl-2H-indazole (SM-6/7)

^{1}H-NMR: $CDCl_3$

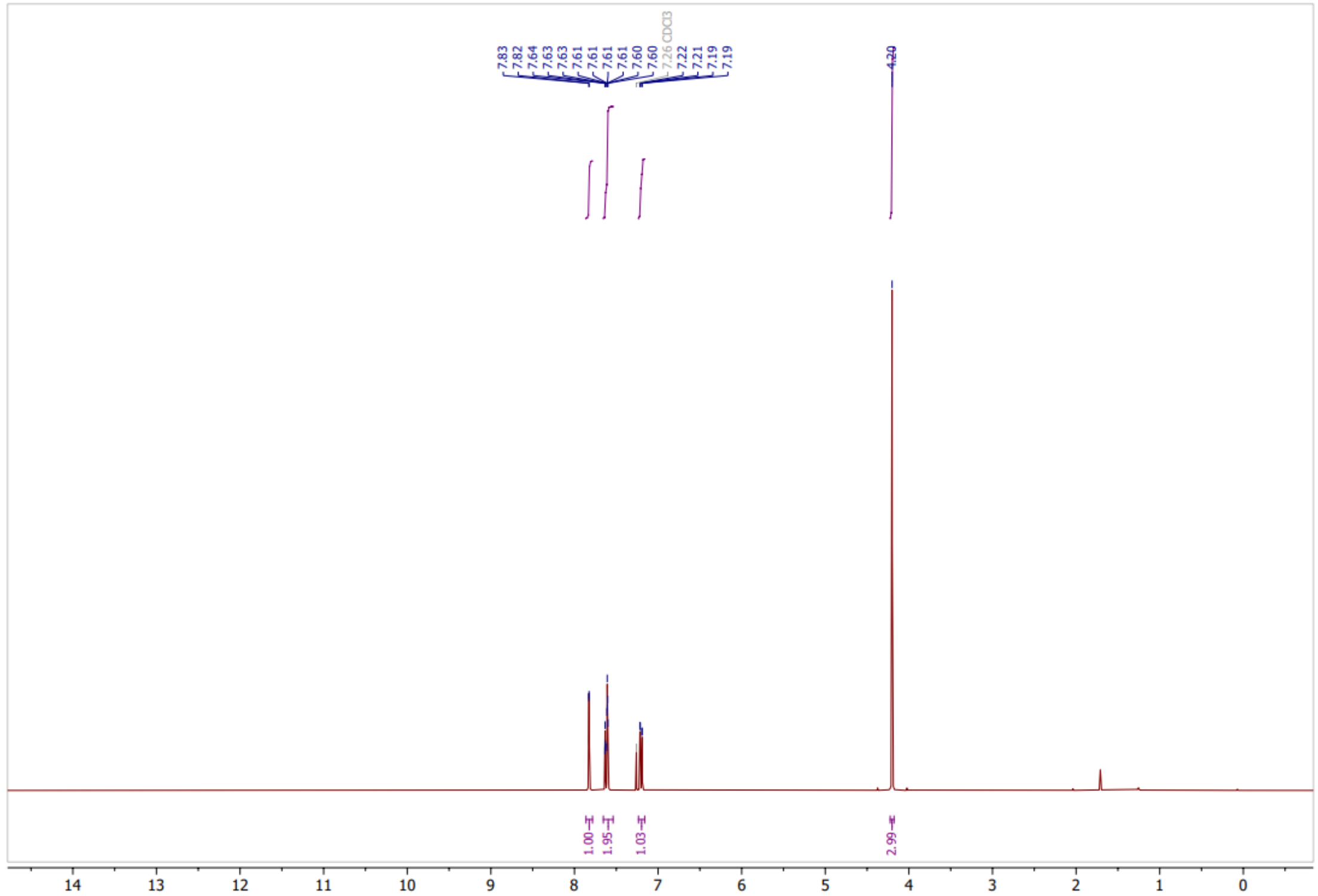

^{13}C-NMR: $CDCl_3$

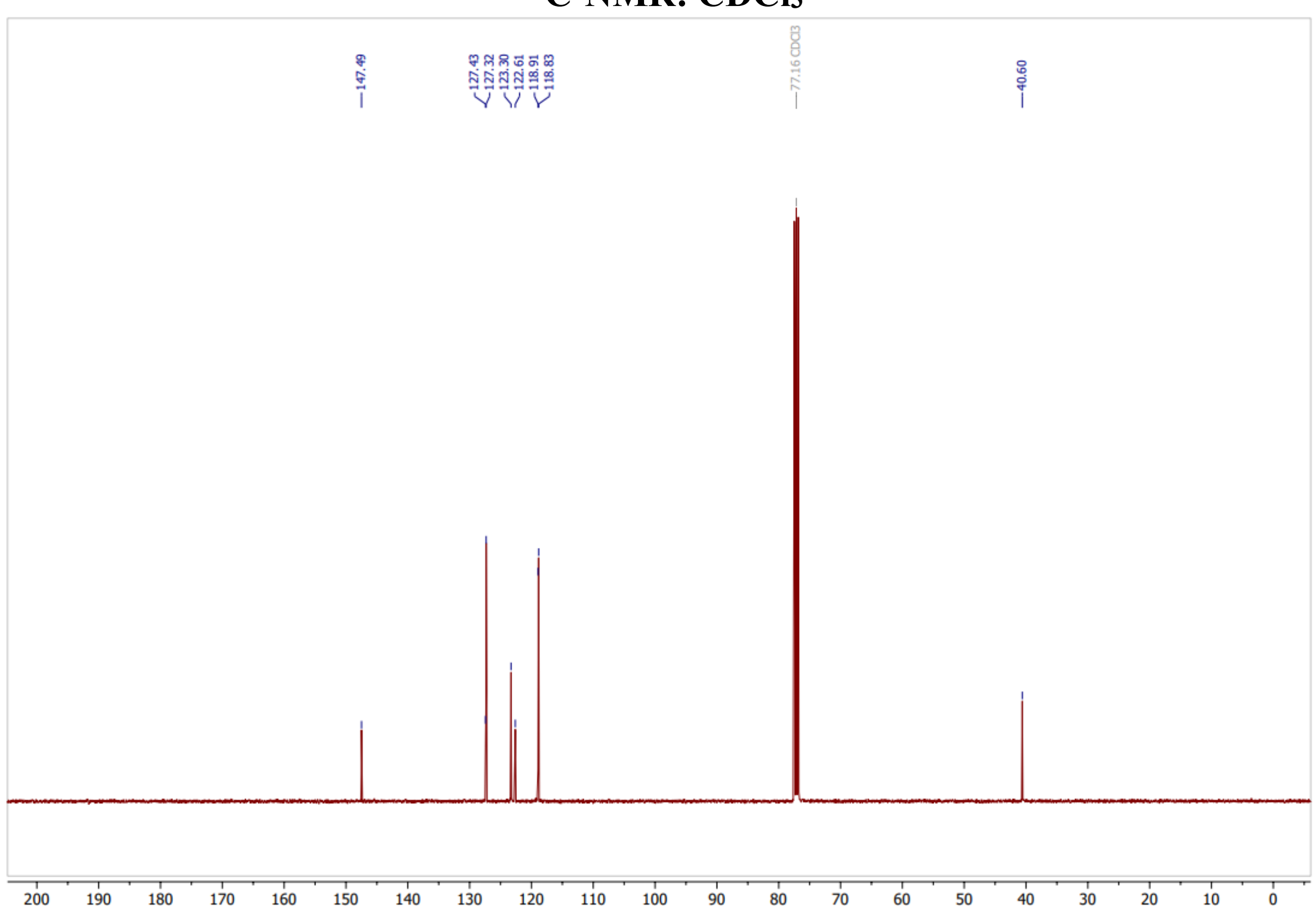

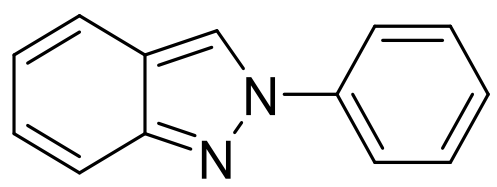

2-phenyl-2H-indazole (SM-9)

^{1}H-NMR: CDCl$_3$

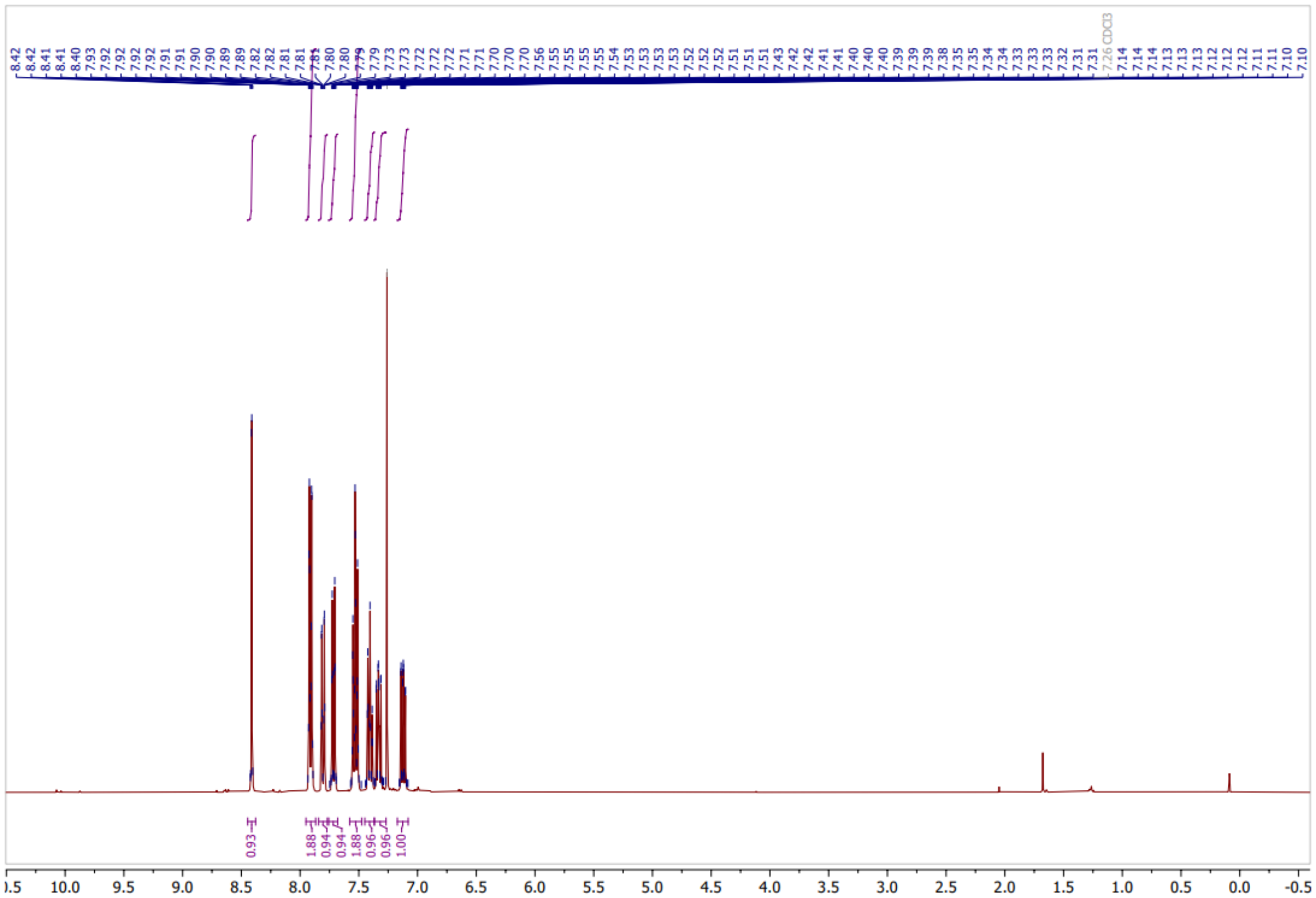

^{13}C-NMR: CDCl$_3$

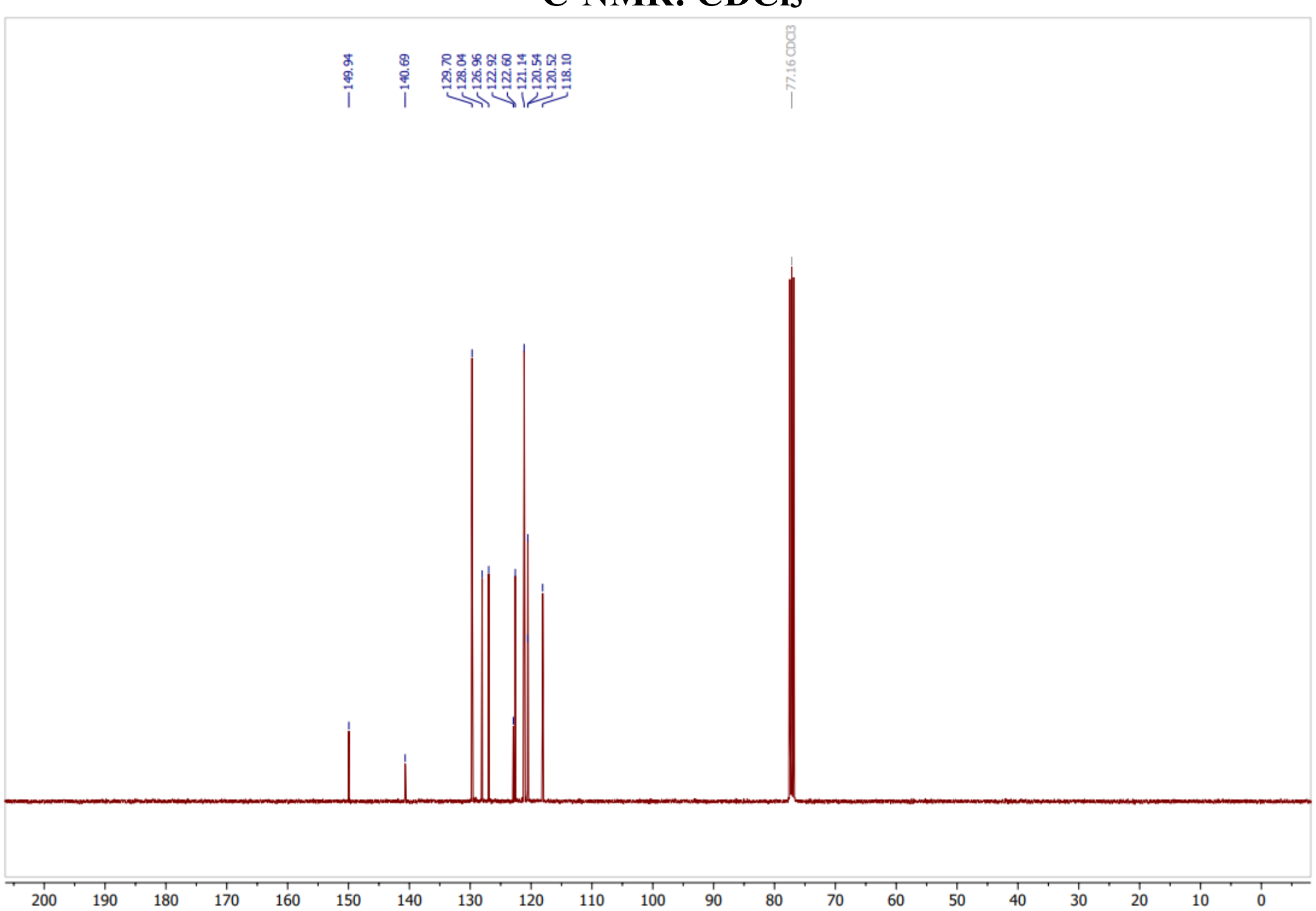

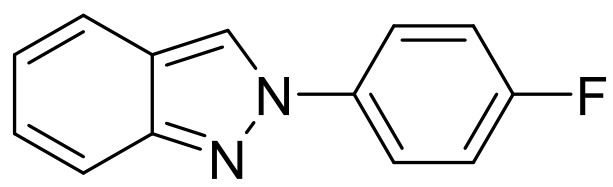

2-(4-fluorophenyl)-2H-indazole (SM-11)

^{1}H-NMR: $CDCl_3$

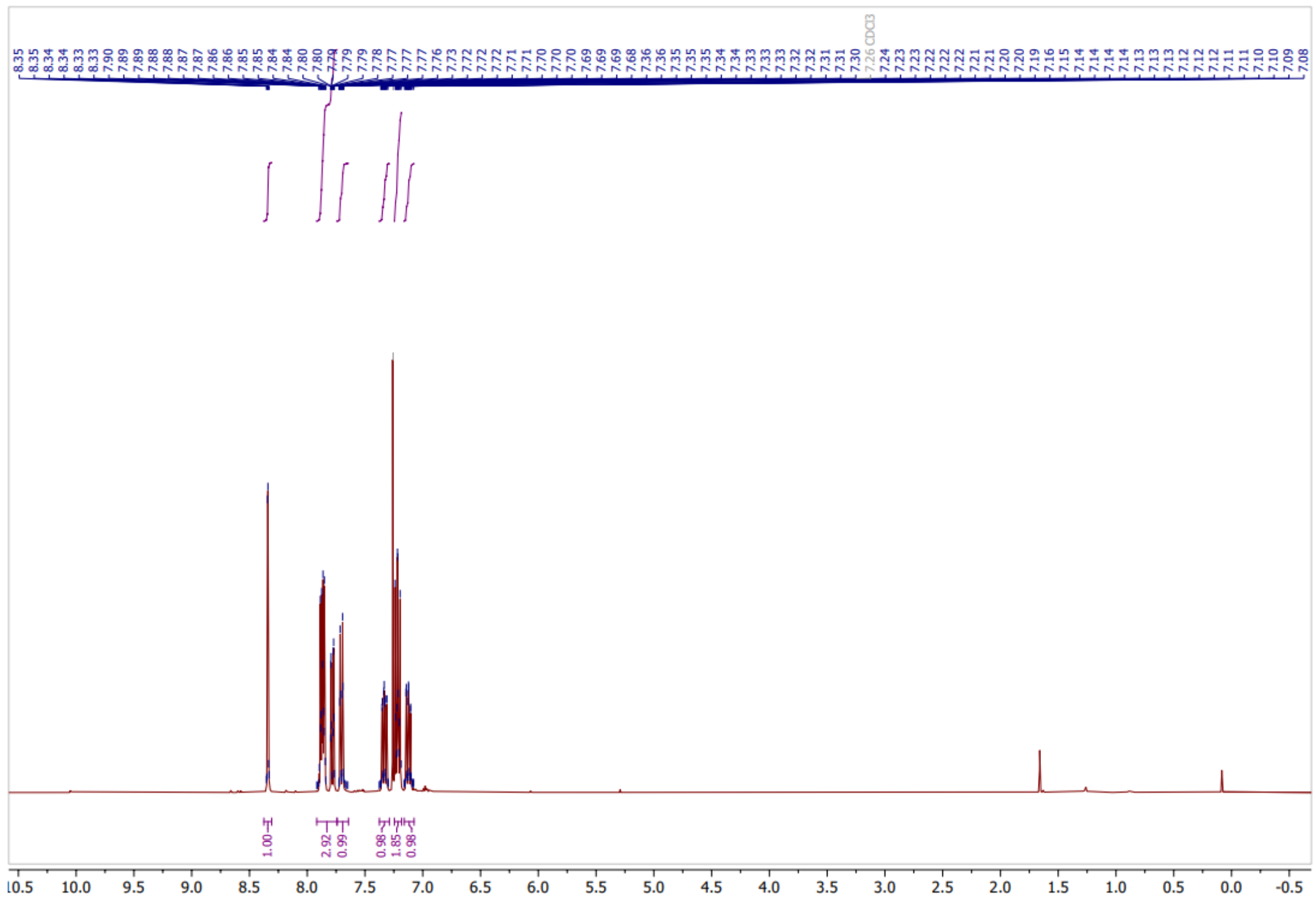

^{13}C-NMR: $CDCl_3$

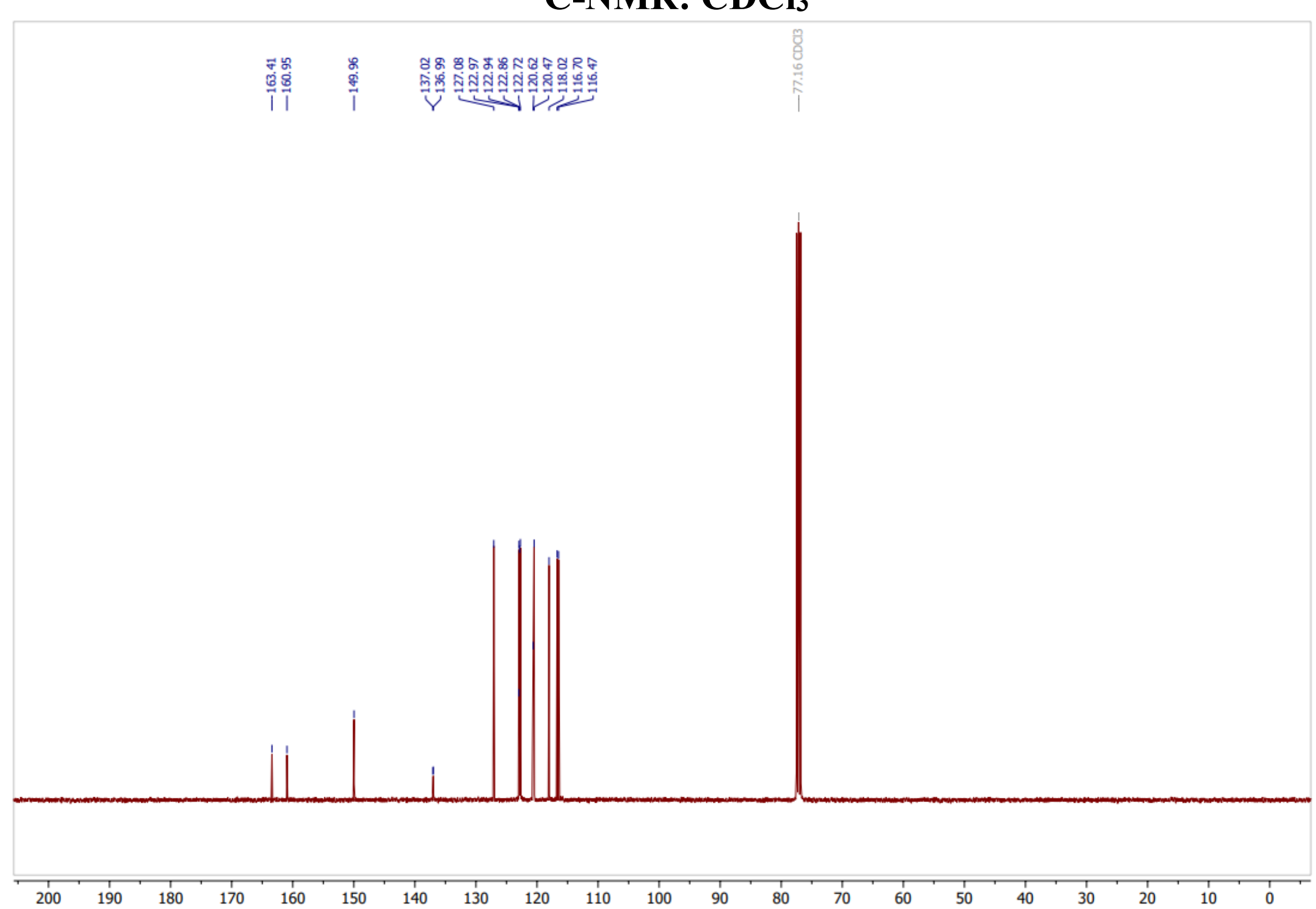

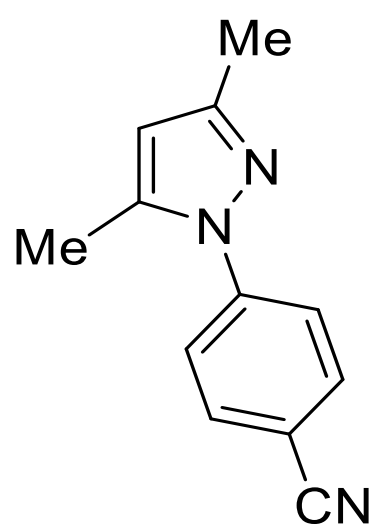

4-(3,5-dimethyl-1H-pyrazol-1-yl)benzonitrile (SM-17/18)

^{1}H-NMR: $CDCl_3$

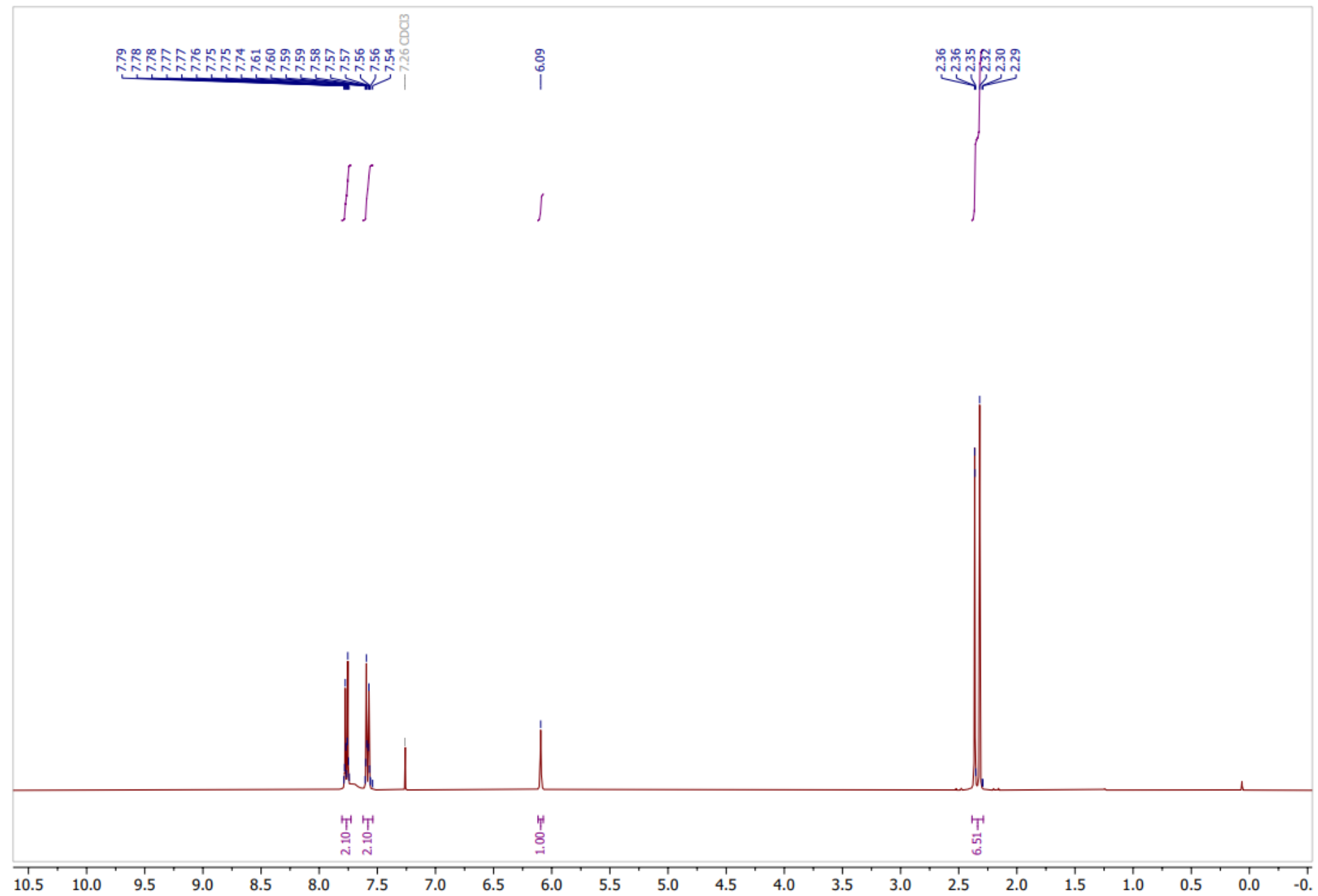

^{13}C-NMR: $CDCl_3$

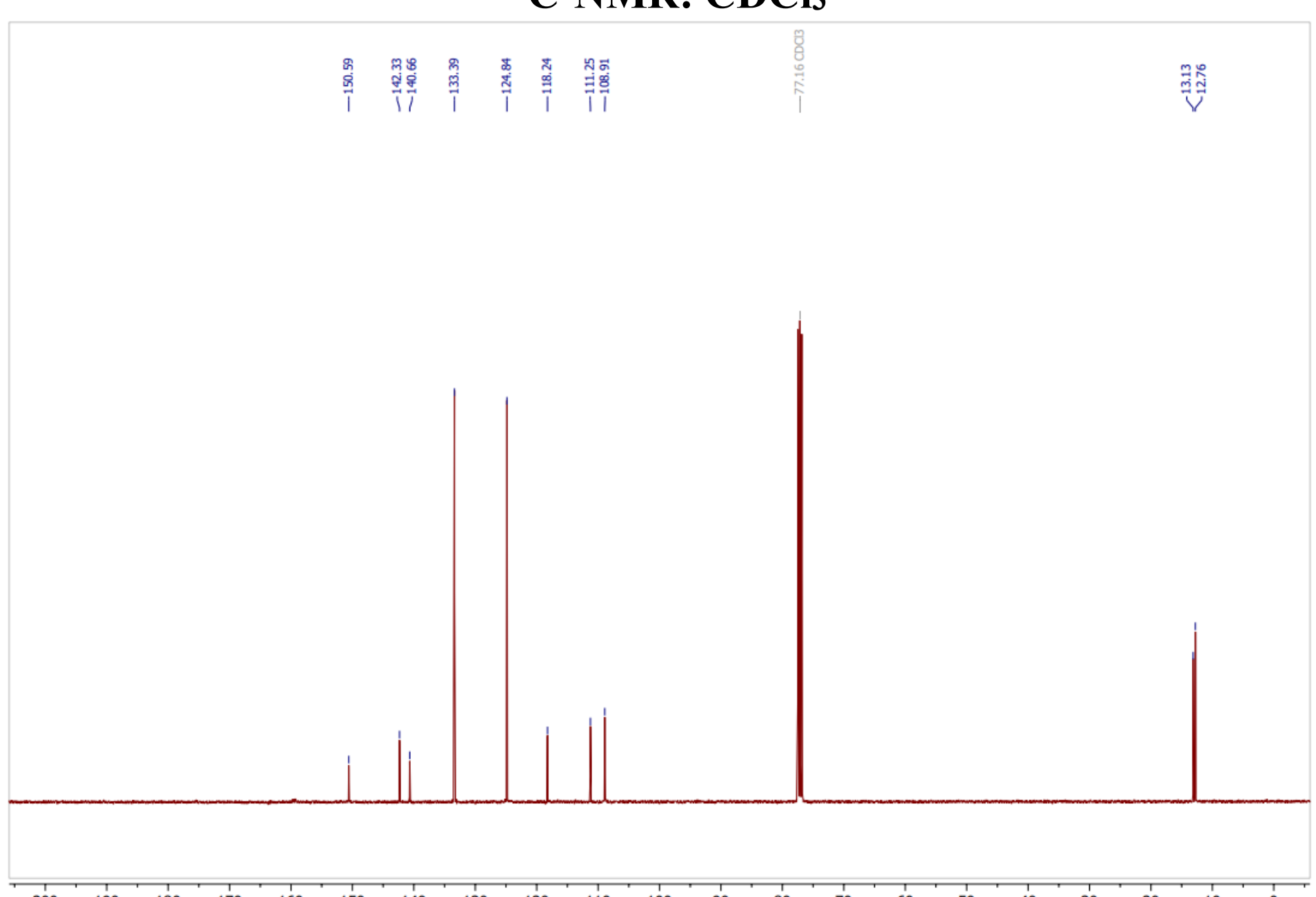

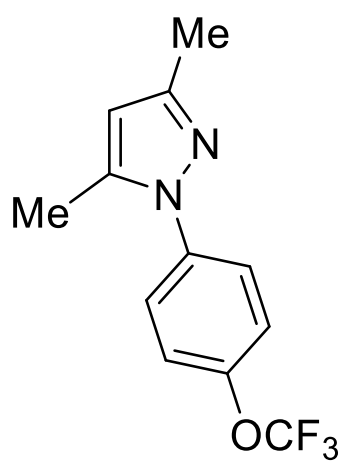

3,5-dimethyl-1-(4-(trifluoromethoxy)phenyl)-1H-pyrazole (SM-19/20)

^{1}H-NMR: CDCl$_3$

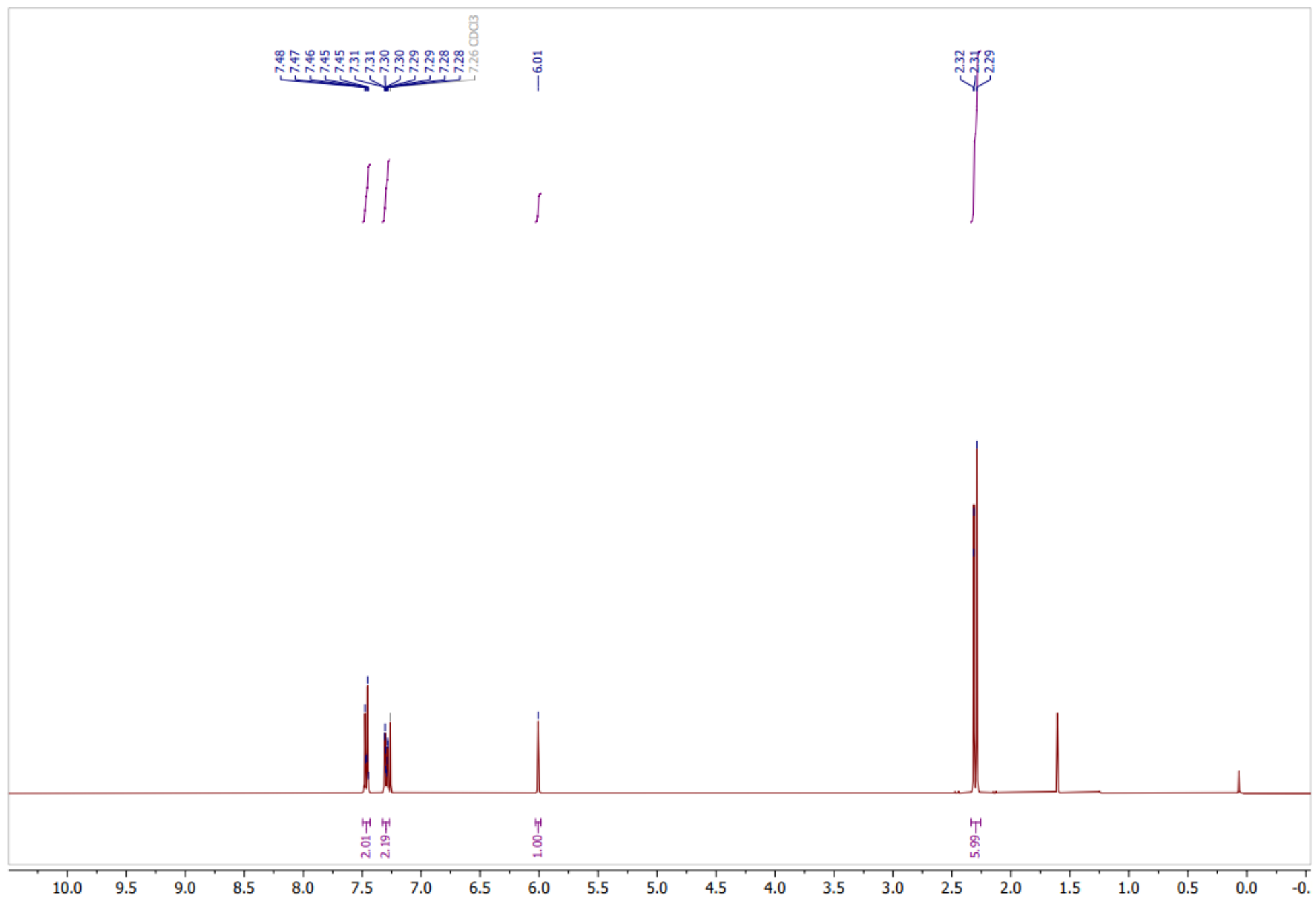

^{13}C-NMR: CDCl$_3$

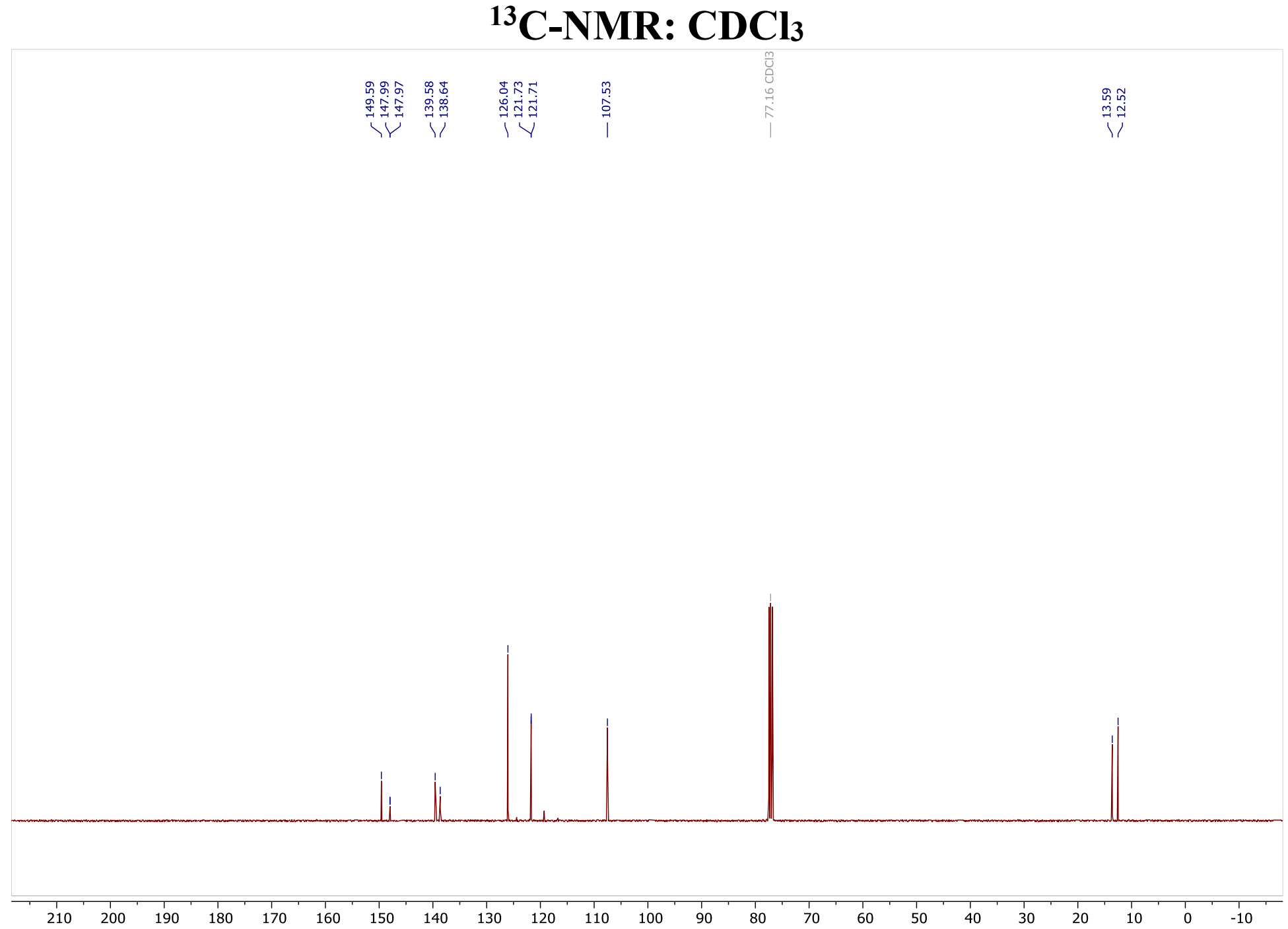

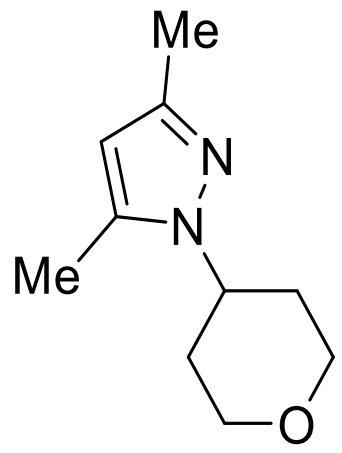

3,5-dimethyl-1-(tetrahydro-2H-pyran-4-yl)-1H-pyrazole (SM-21/22)

^{1}H-NMR: CDCl3

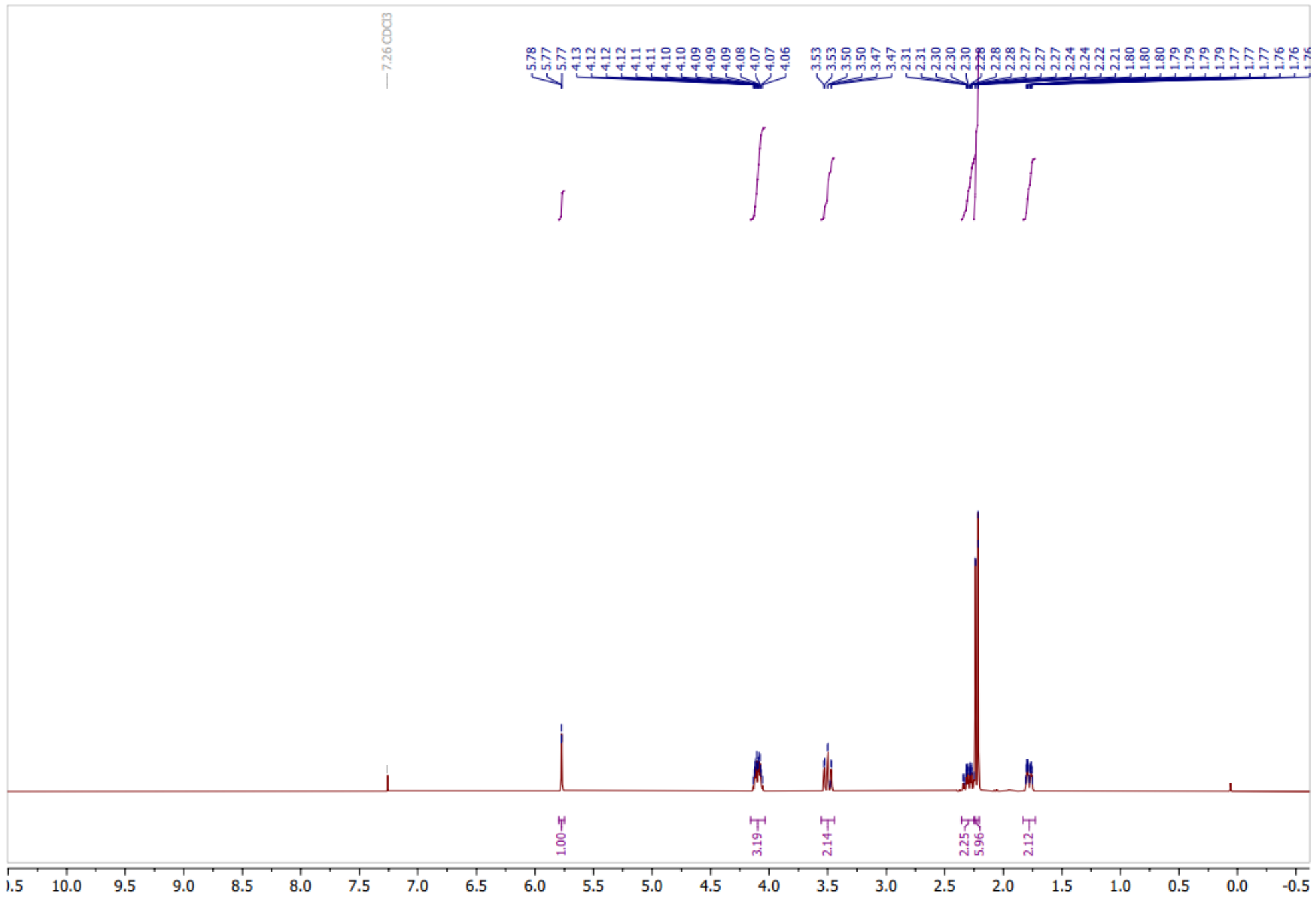

^{13}C-NMR: CDCl3

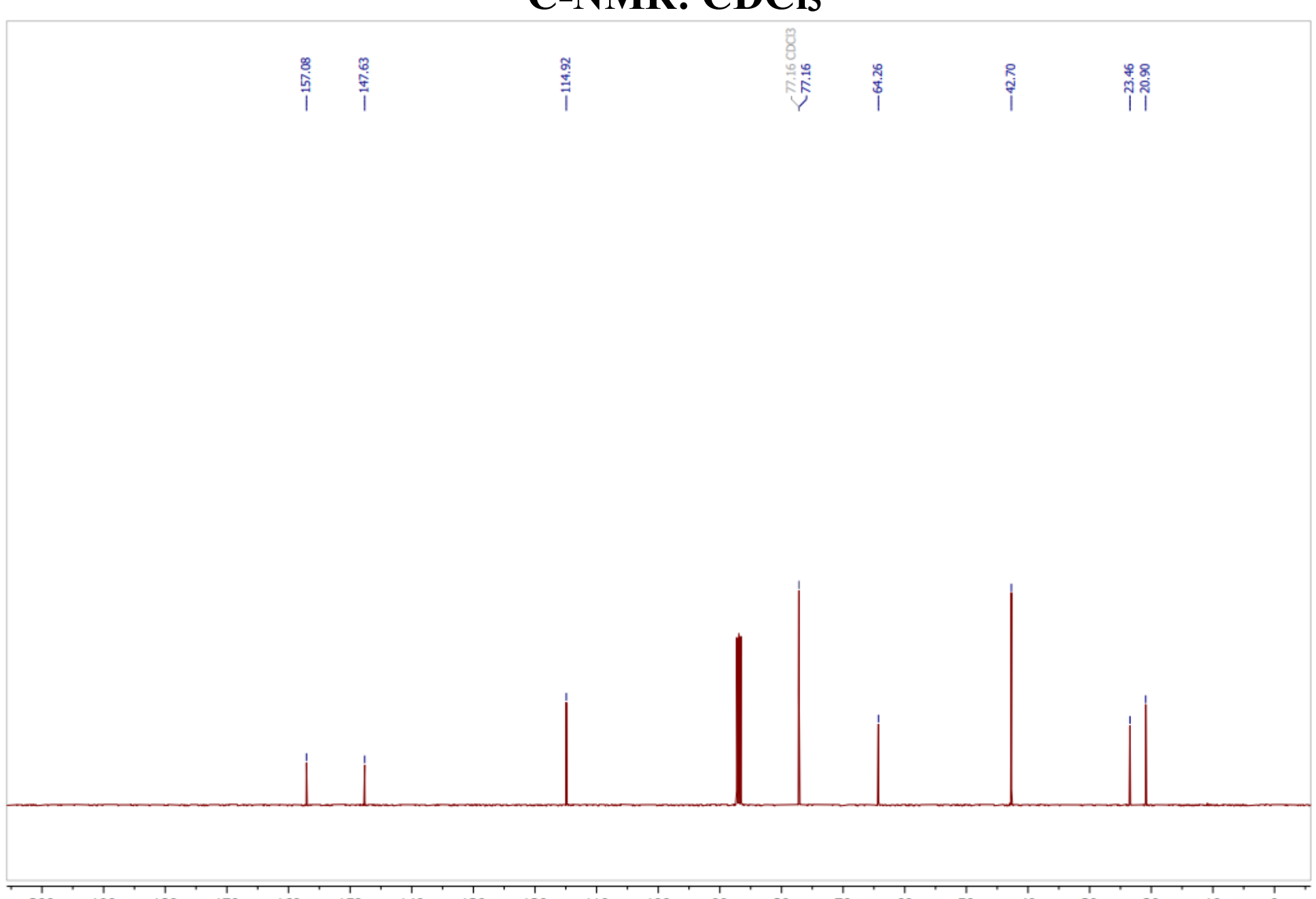

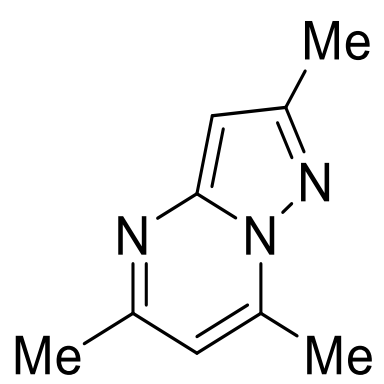

2,5,7-trimethylpyrazolo[1,5-a]pyrimidine (SM-45/46)

^{1}H-NMR: $CDCl_3$

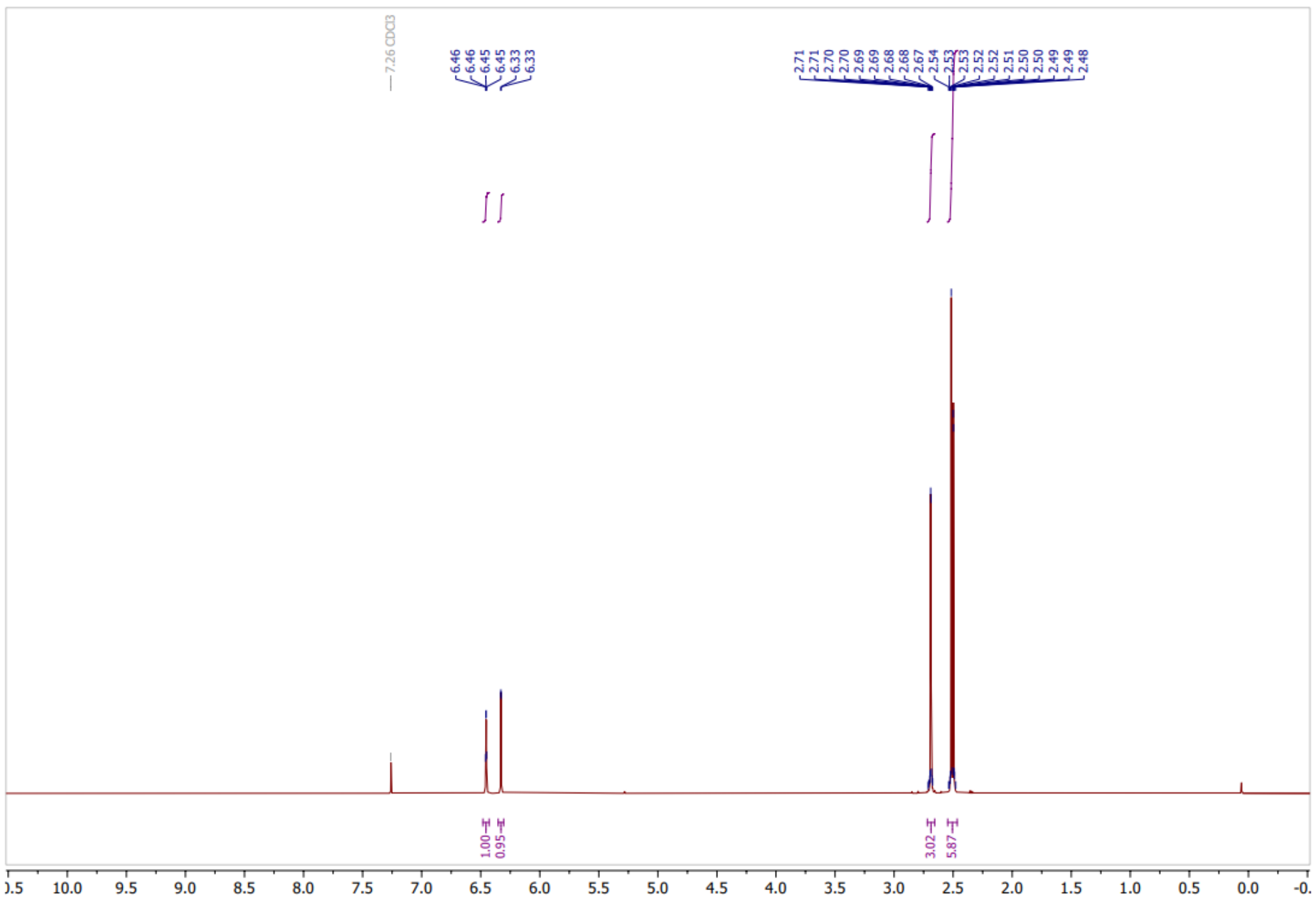

^{13}C-NMR: $CDCl_3$

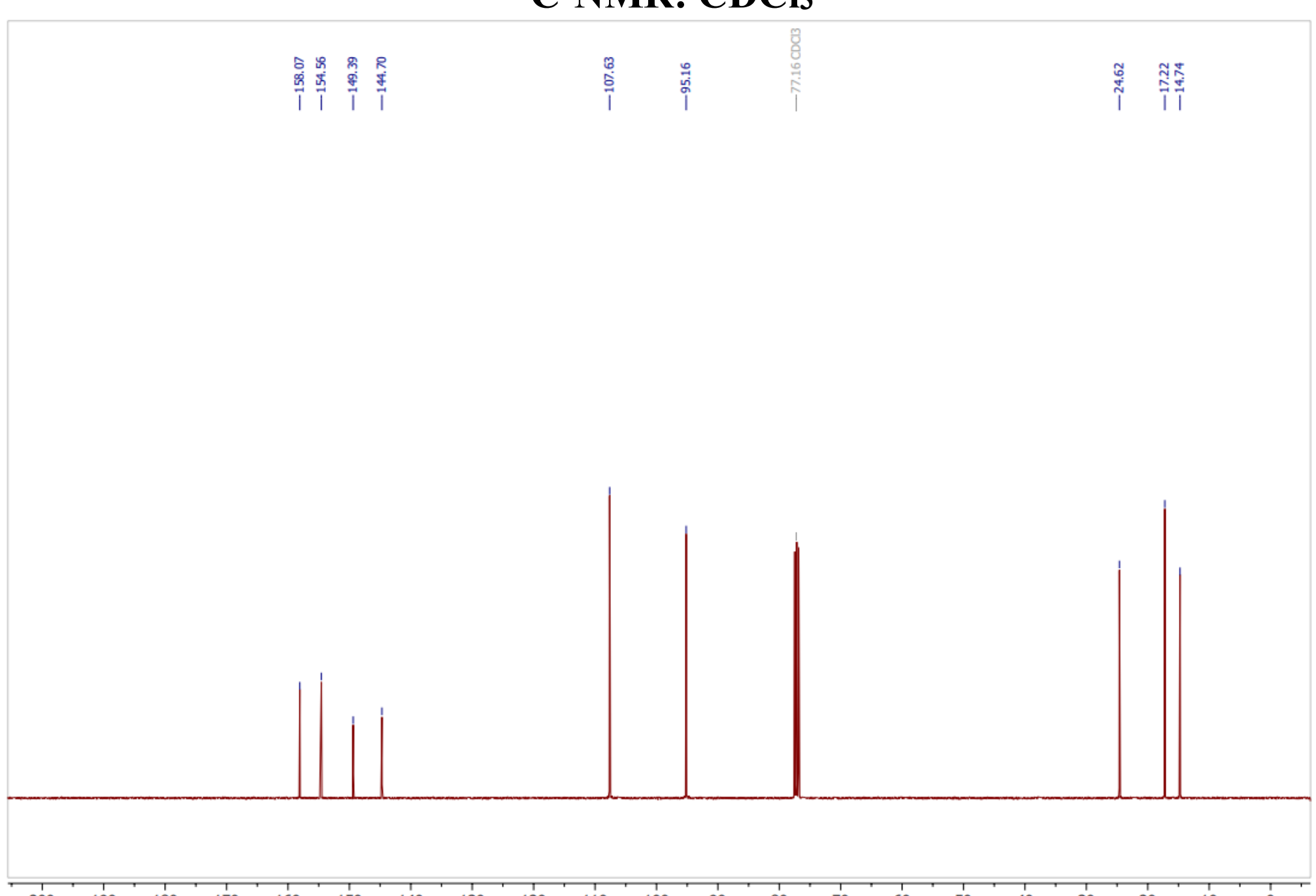

Halogenated Products:

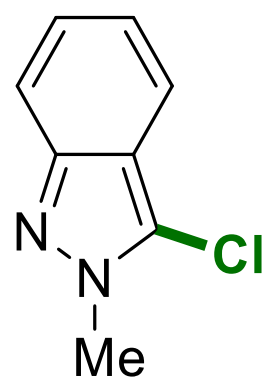

3-chloro-2-methyl-2H-indazole (2)

^{1}H-NMR: $CDCl_3$

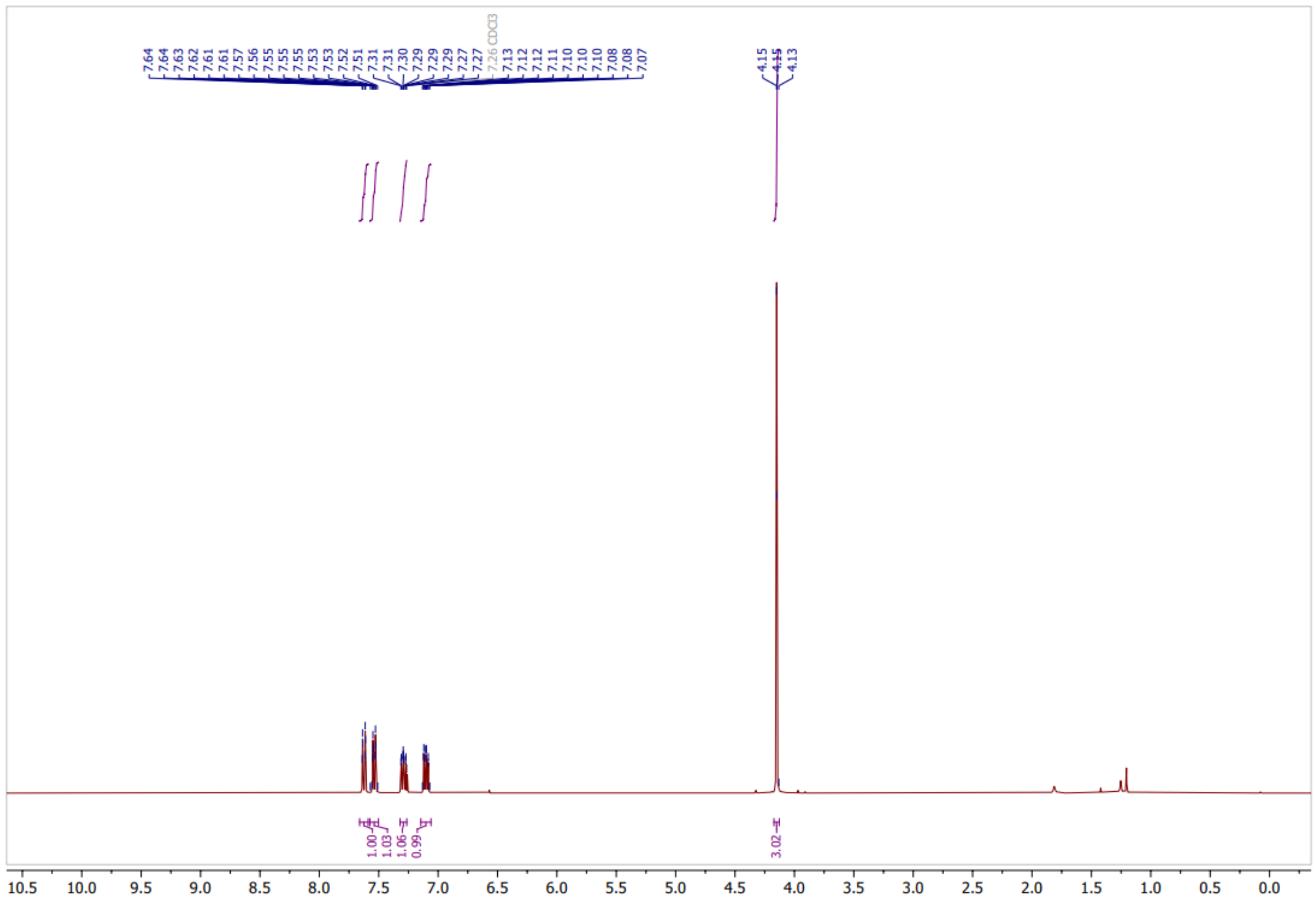

^{13}C-NMR: $CDCl_3$

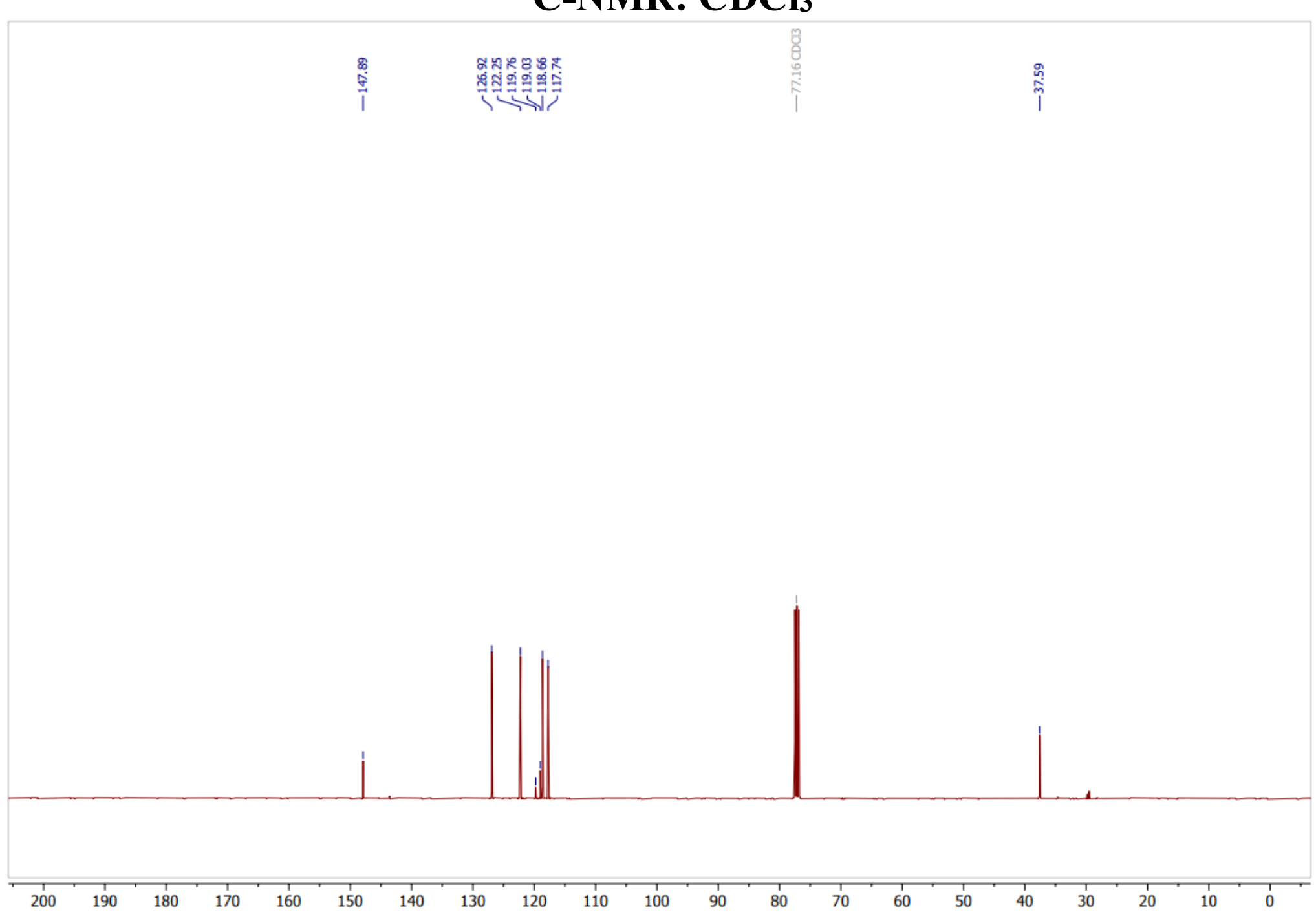

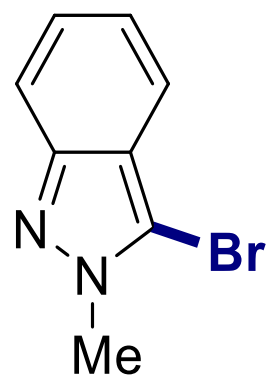

3-bromo-2-methyl-2H-indazole (3)

^{1}H-NMR: $CDCl_3$

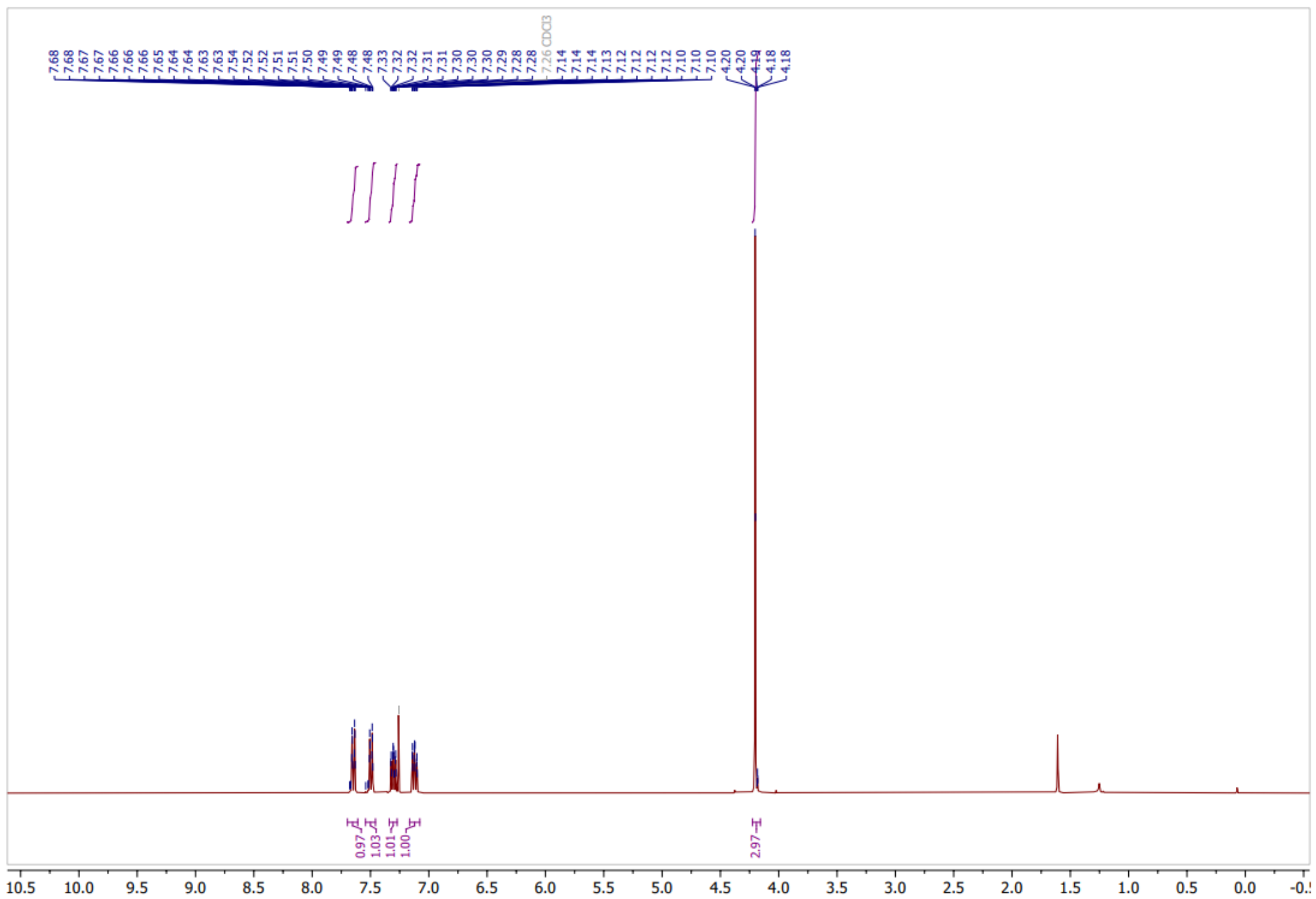

^{13}C-NMR: $CDCl_3$

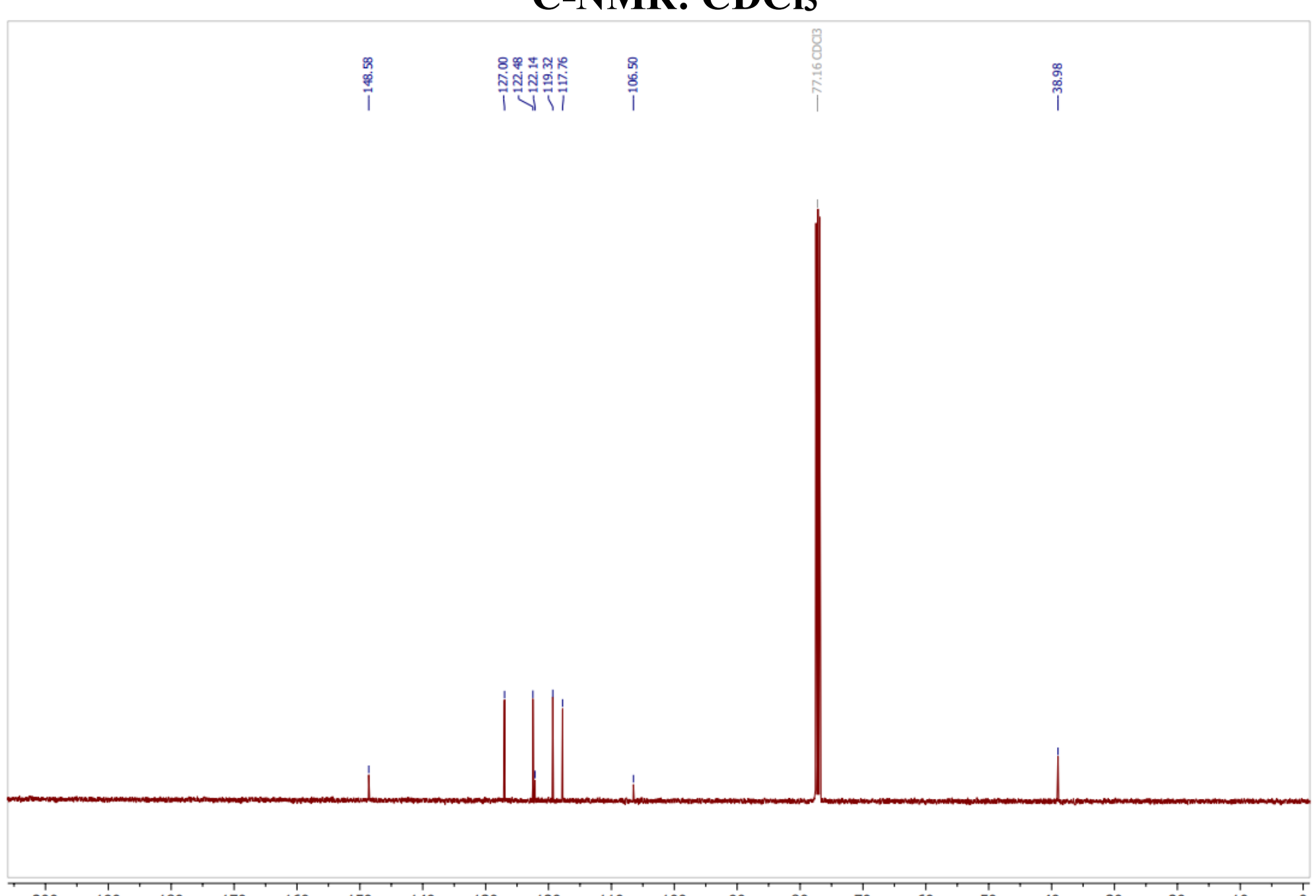

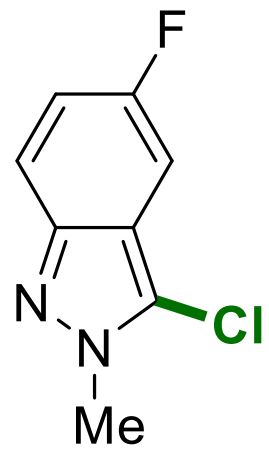

3-chloro-5-fluoro-2-methyl-2H-indazole (4)

^{1}H-NMR: CDCl$_3$

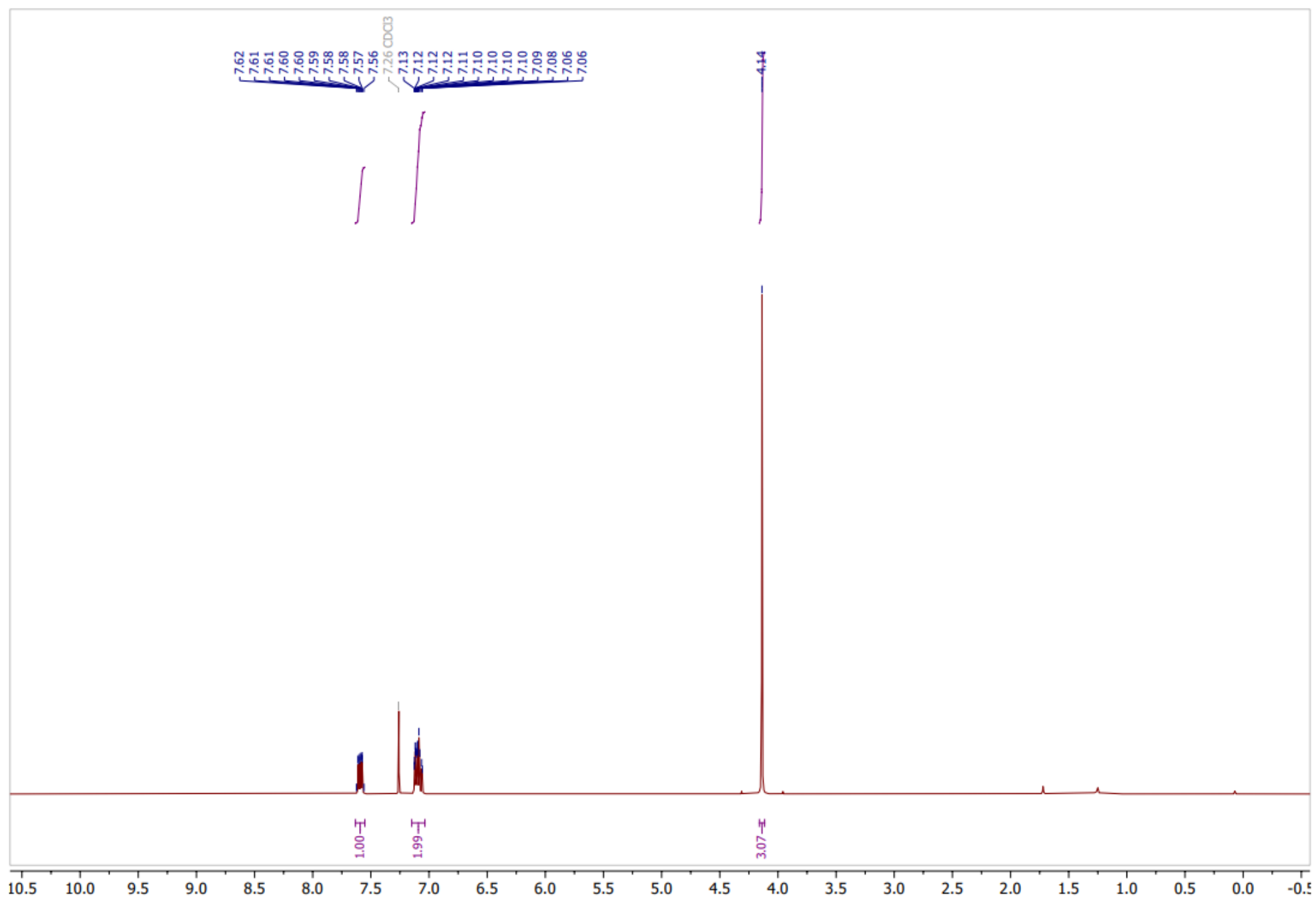

^{13}C-NMR: CDCl$_3$

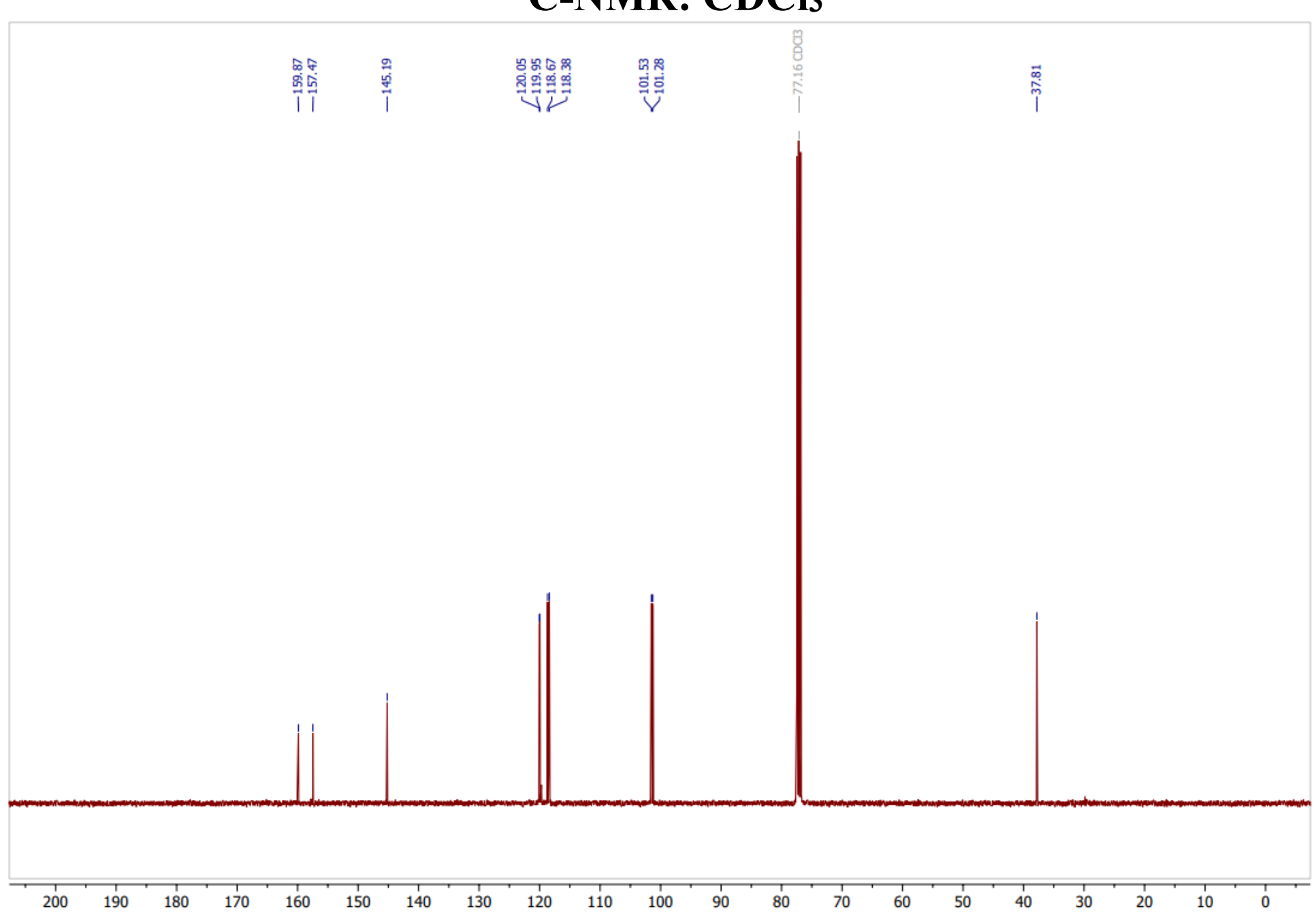

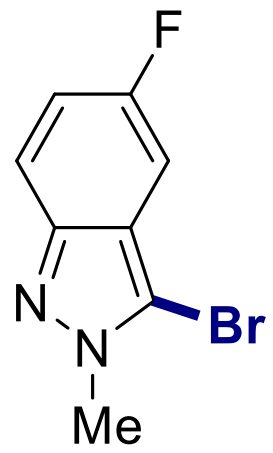

3-bromo-5-fluoro-2-methyl-2H-indazole (5)

^{1}H-NMR: CDCl$_3$

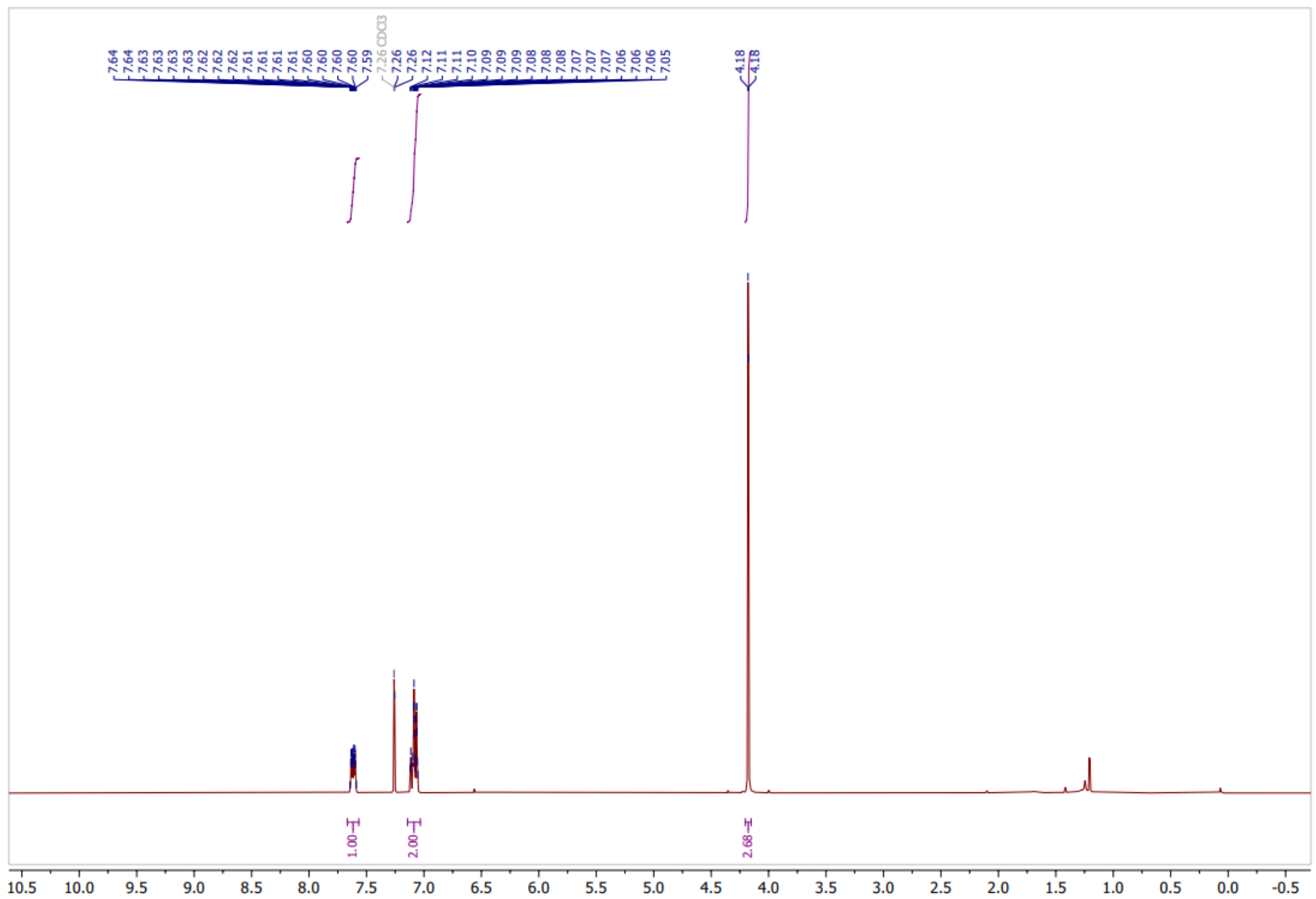

^{13}C-NMR: CDCl$_3$

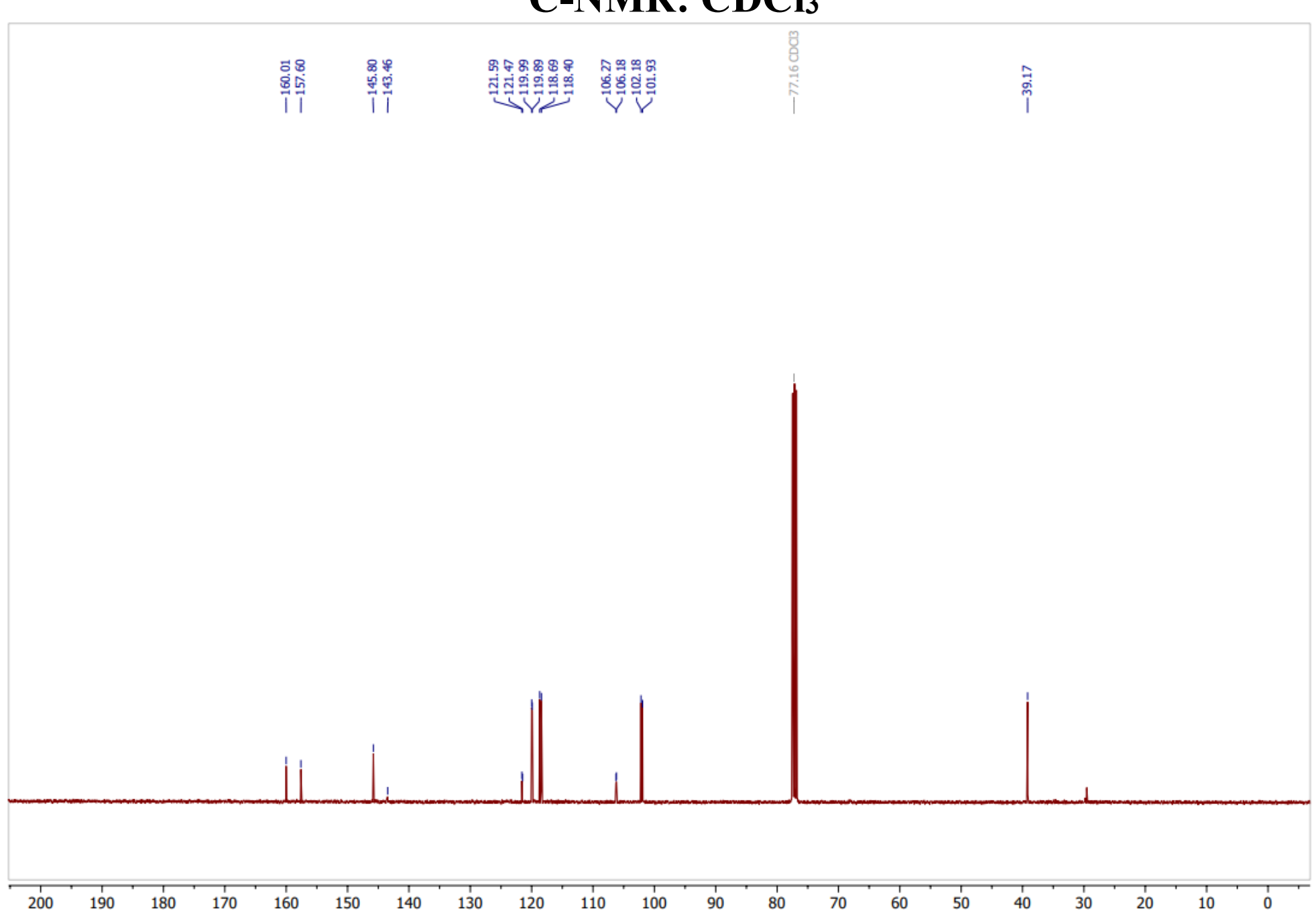

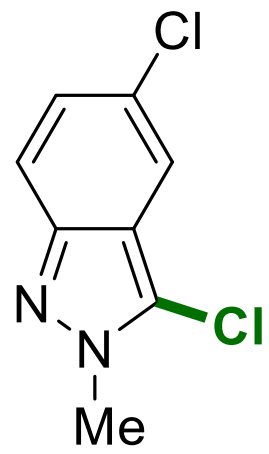

3,5-dichloro-2-methyl-2H-indazole (6)

^{1}H-NMR: CDCl$_3$

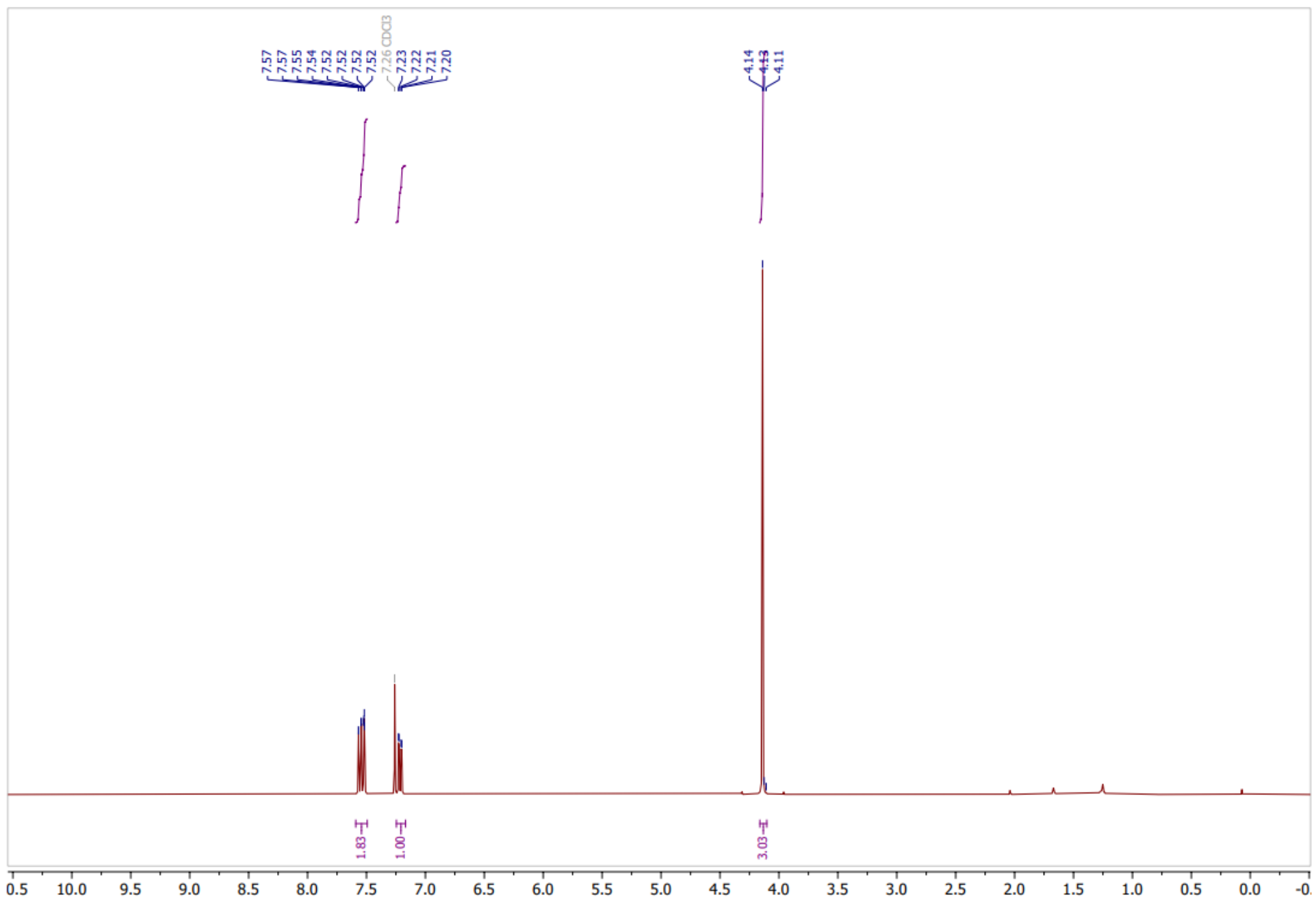

^{1}H-NMR: CDCl$_3$

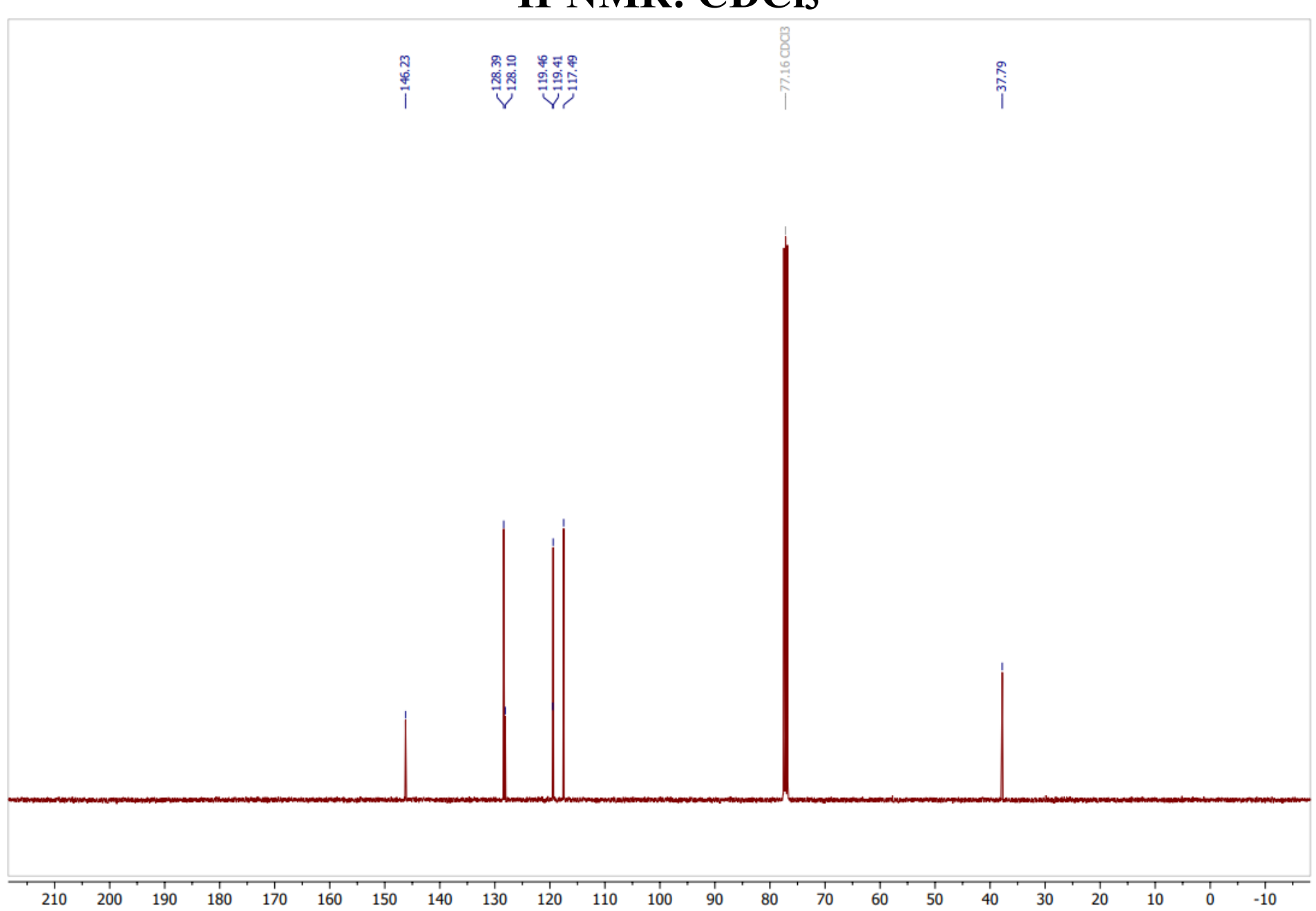

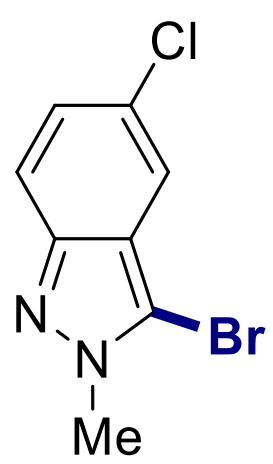

3-bromo-5-chloro-2-methyl-2H-indazole (7)

^{1}H-NMR: CDCl$_3$

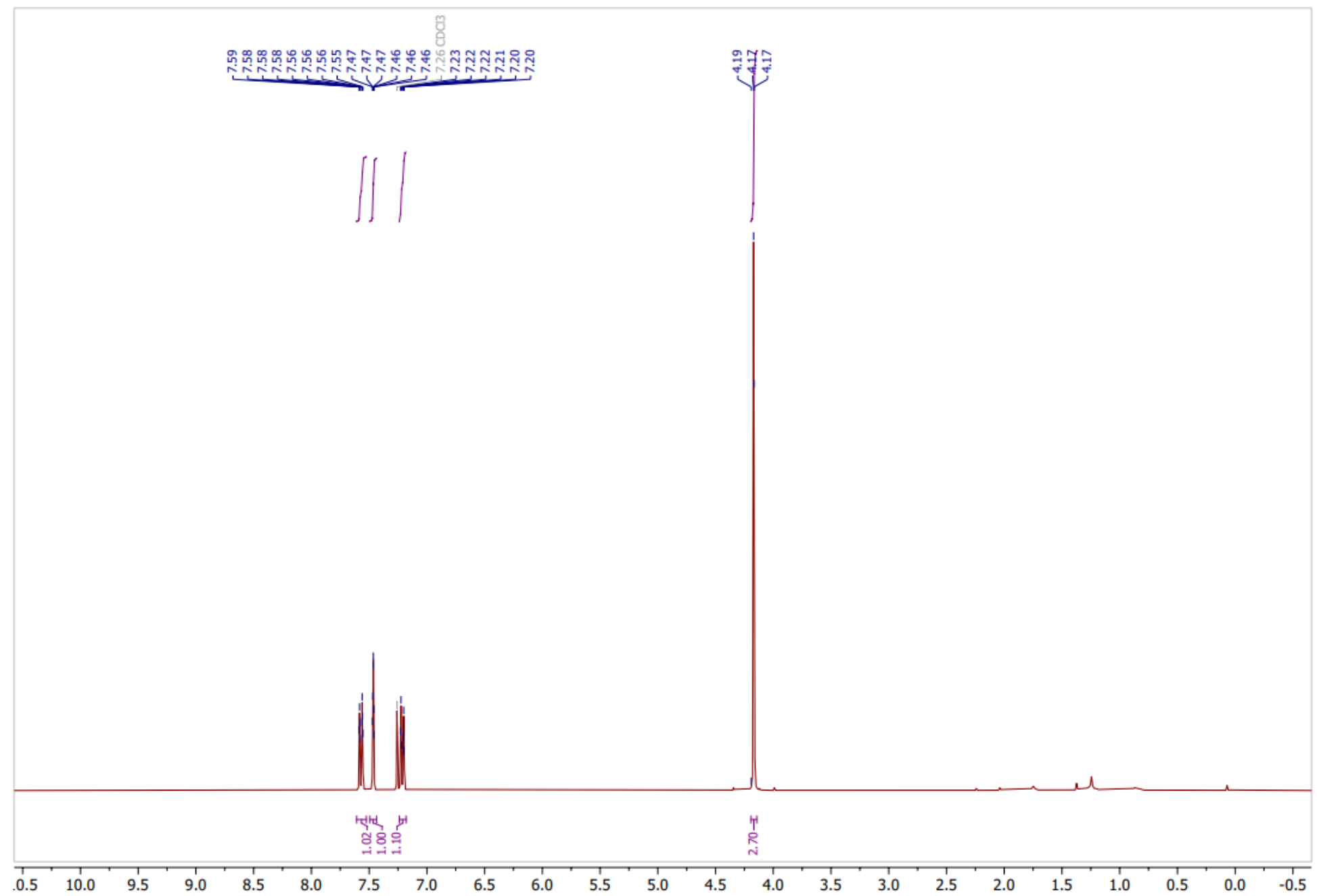

^{13}C-NMR: CDCl$_3$

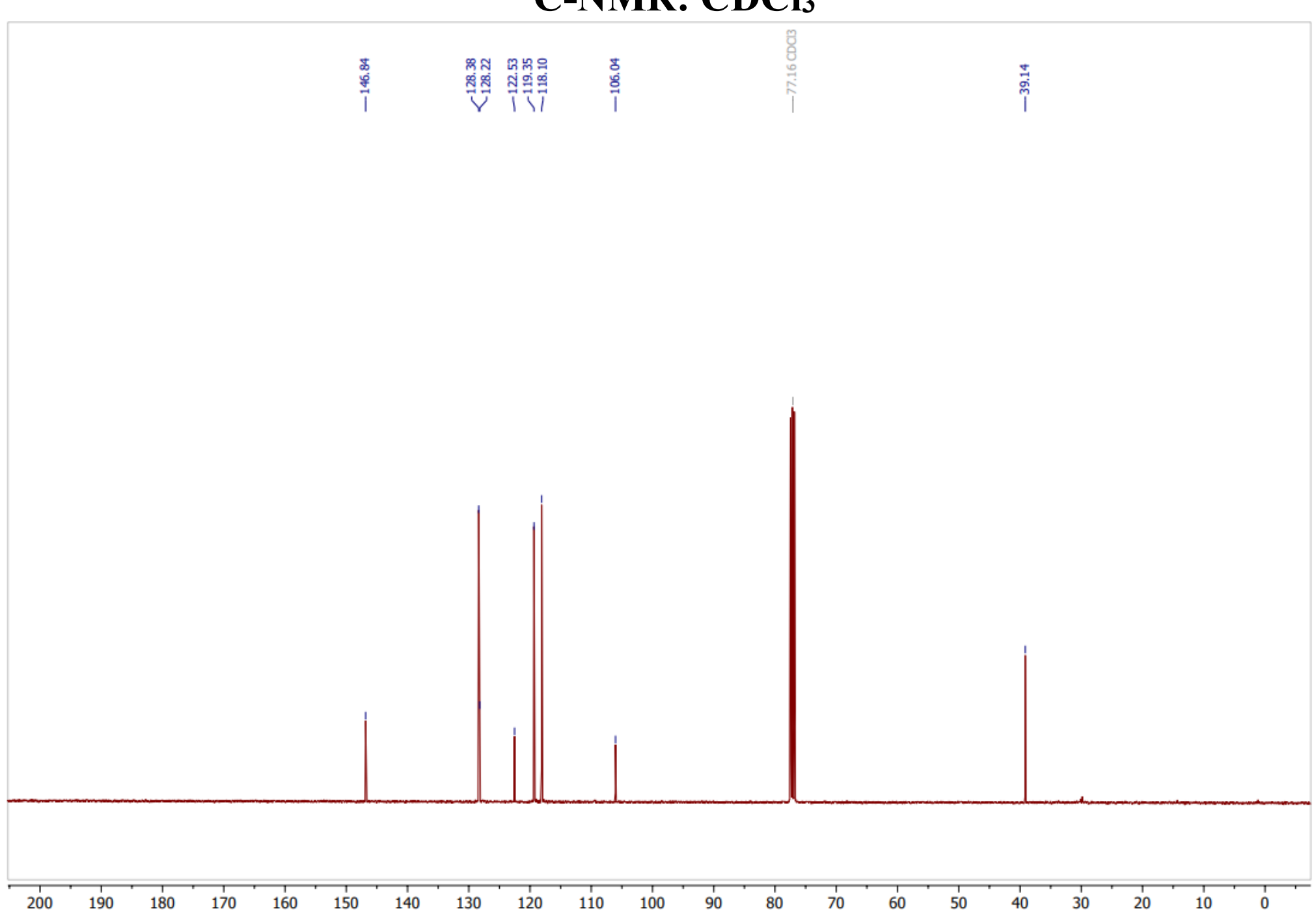

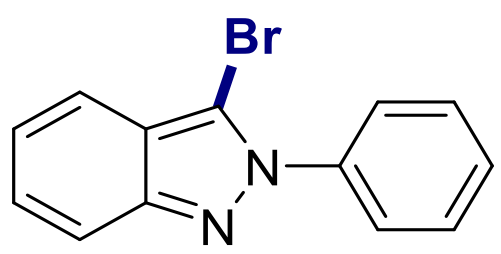

3-bromo-2-phenyl-2H-indazole (9)

^{1}H-NMR: $CDCl_3$

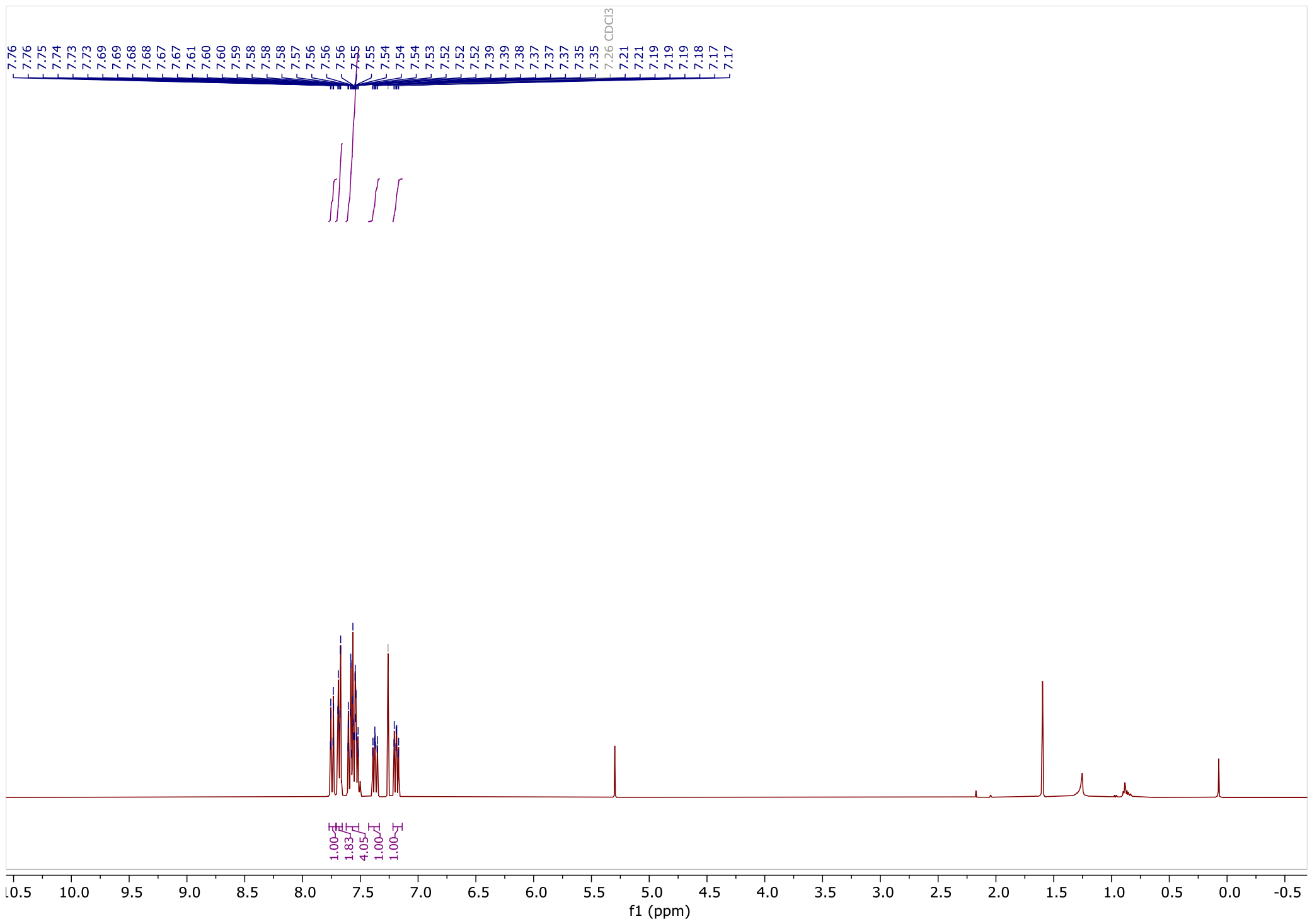

^{13}C-NMR: $CDCl_3$

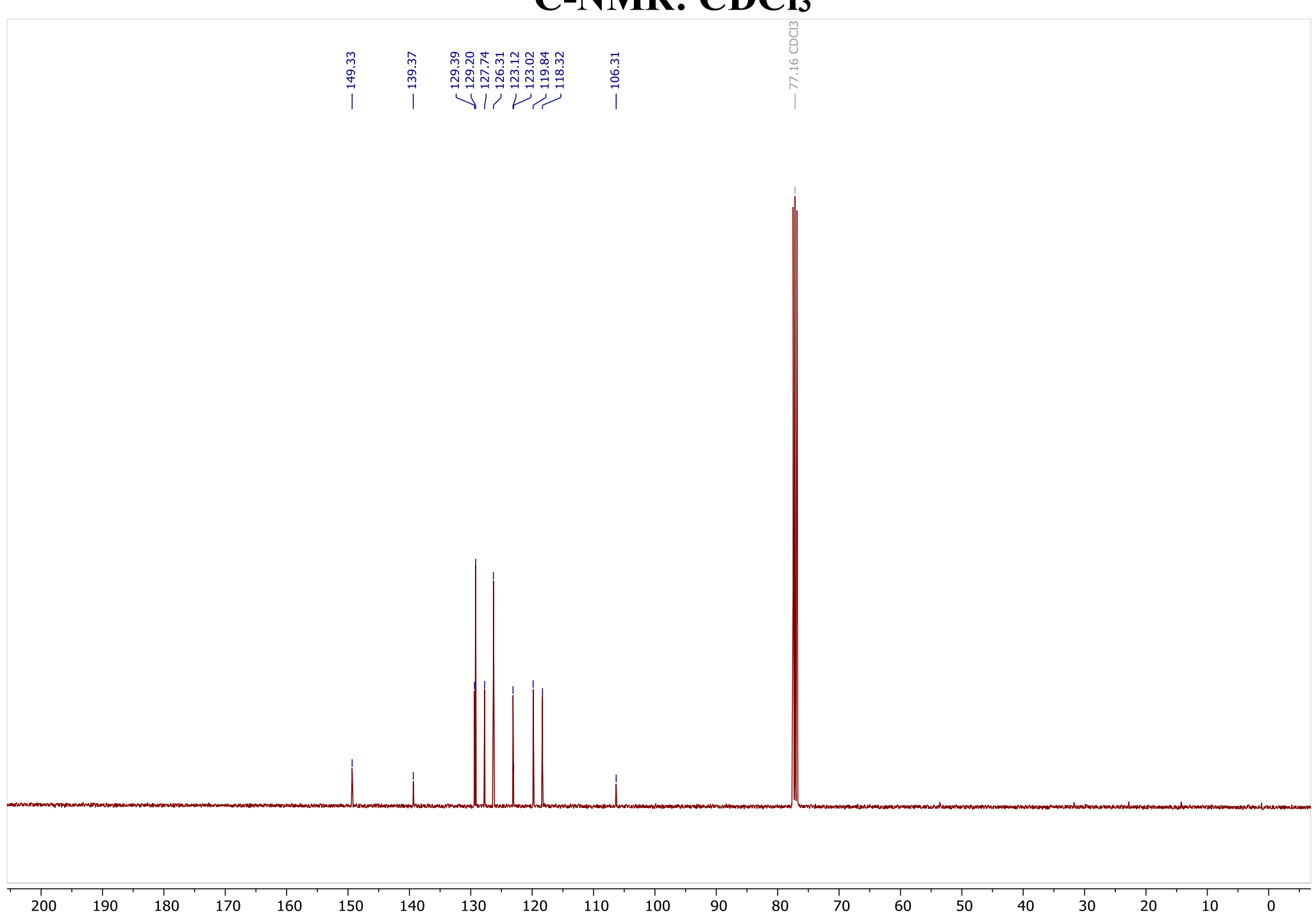

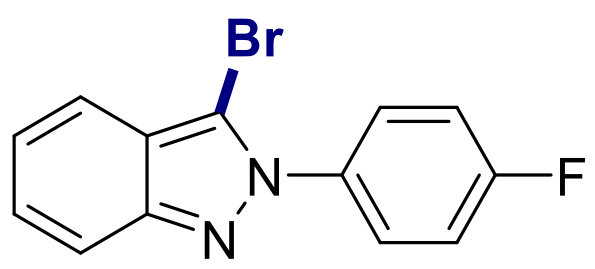

3-bromo-2-(4-fluorophenyl)-2H-indazole (11)

^{1}H-NMR: $CDCl_3$

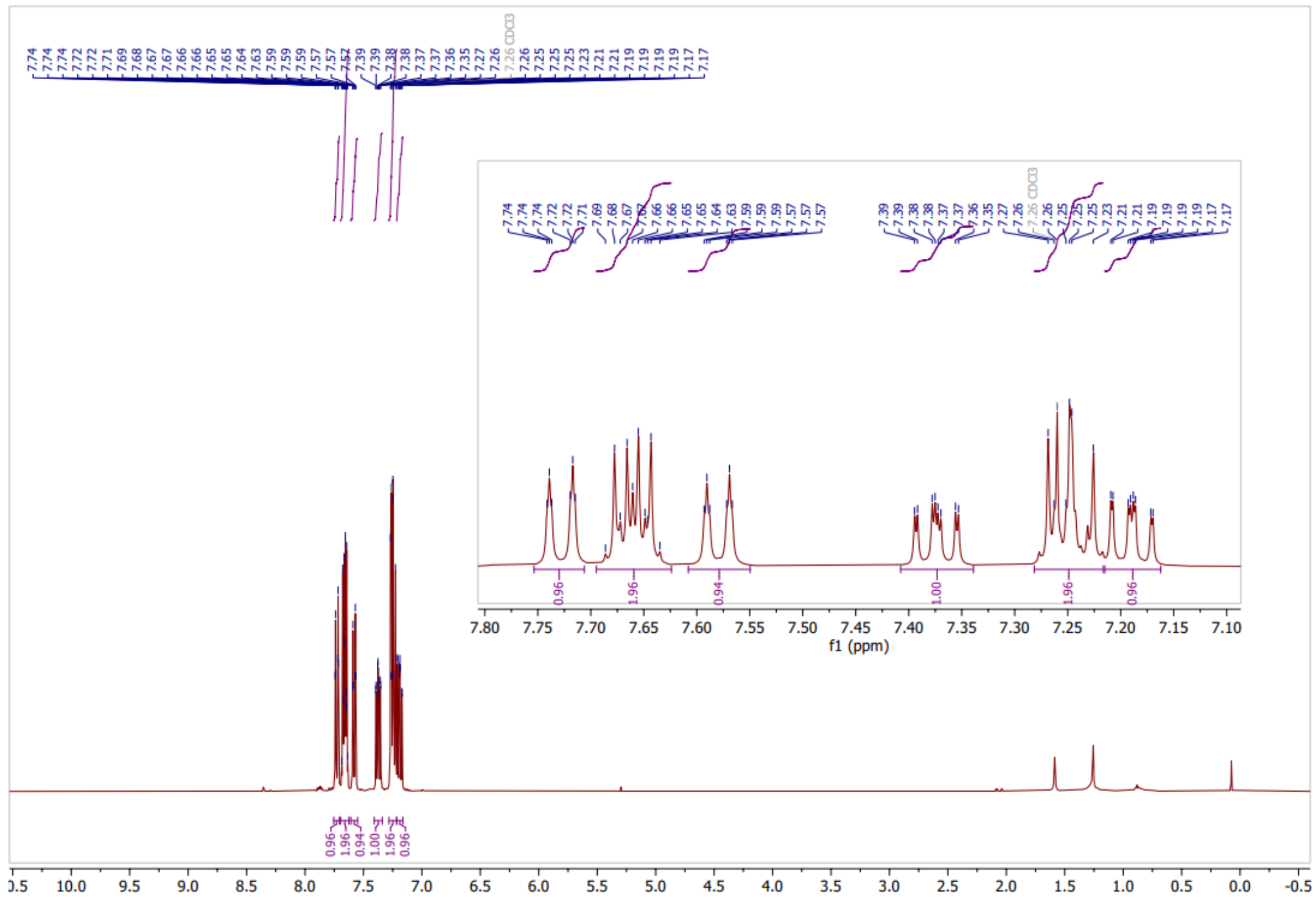

^{13}C-NMR: $CDCl_3$

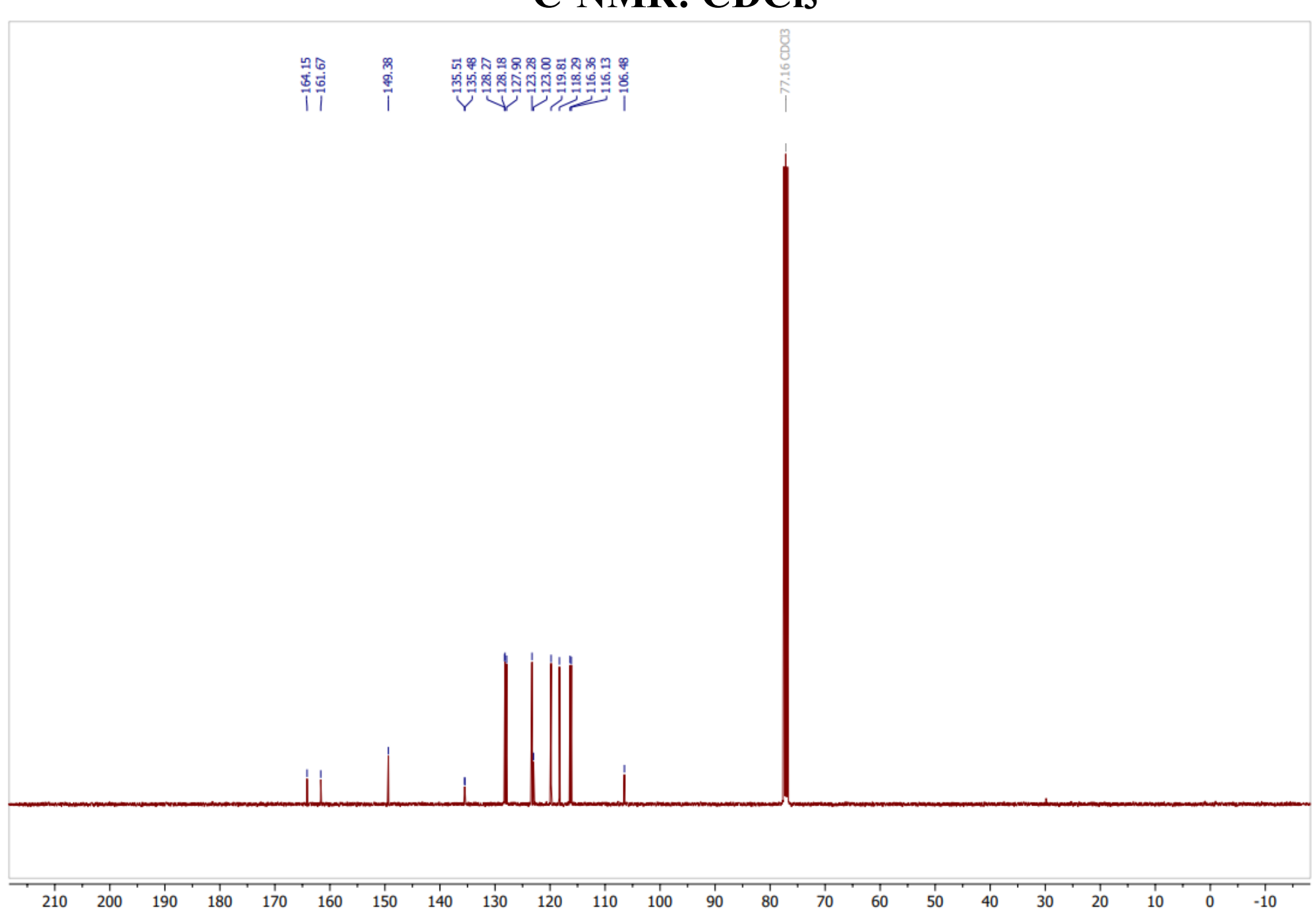

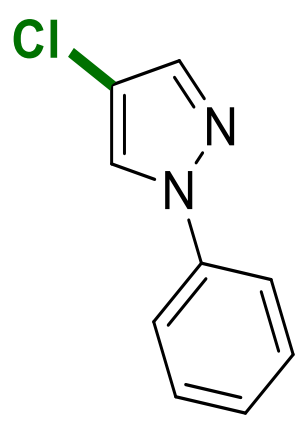

4-chloro-1-phenyl-1H-pyrazole (13)

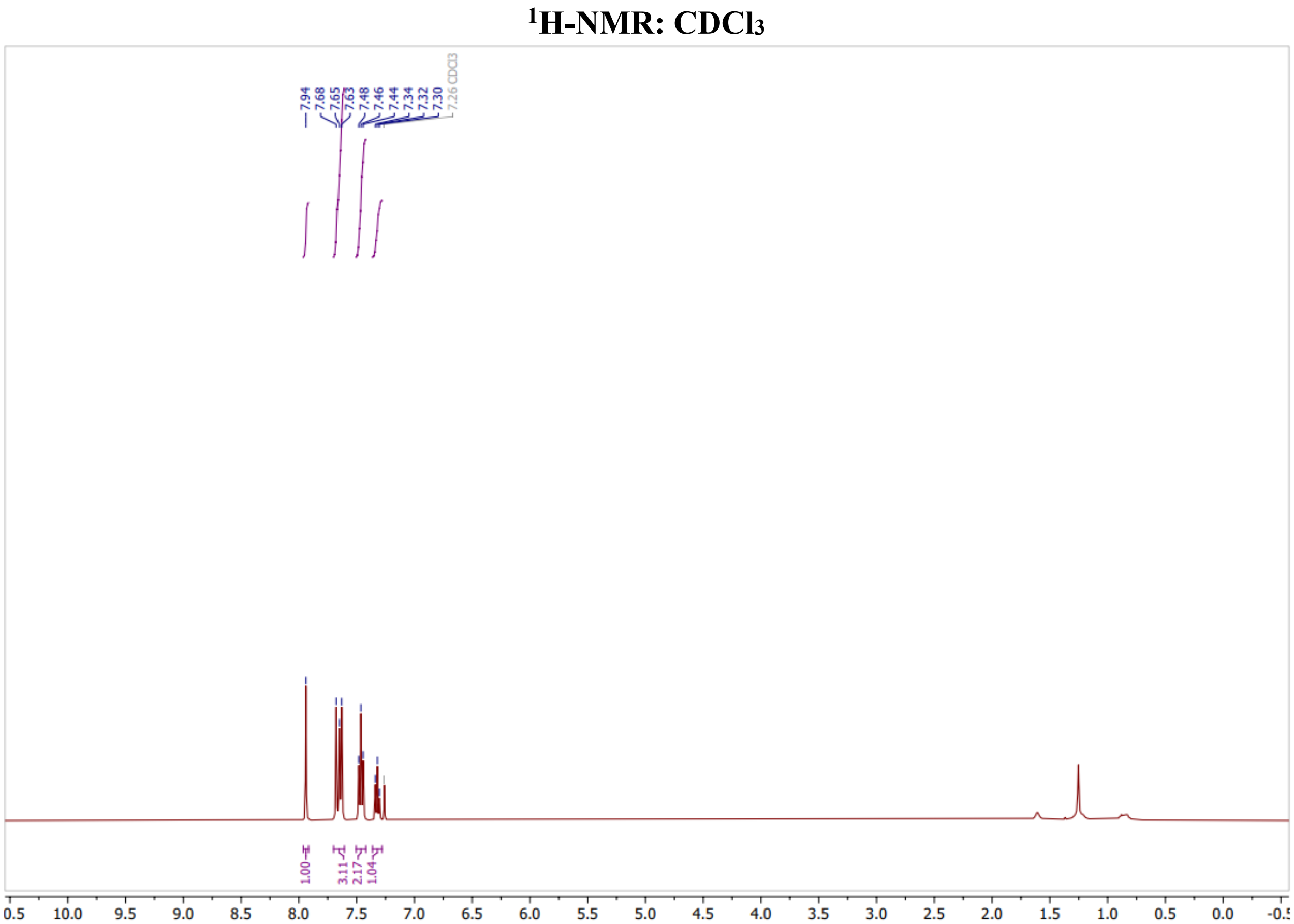

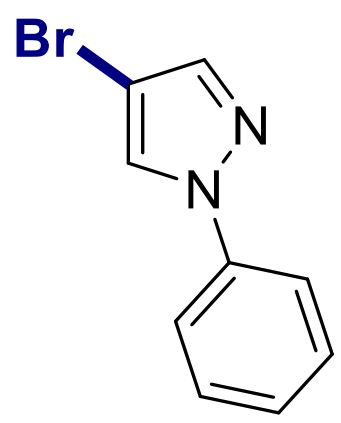

4-bromo-1-phenyl-1H-pyrazole (14)

'^{1}H-NMR: CDCl3

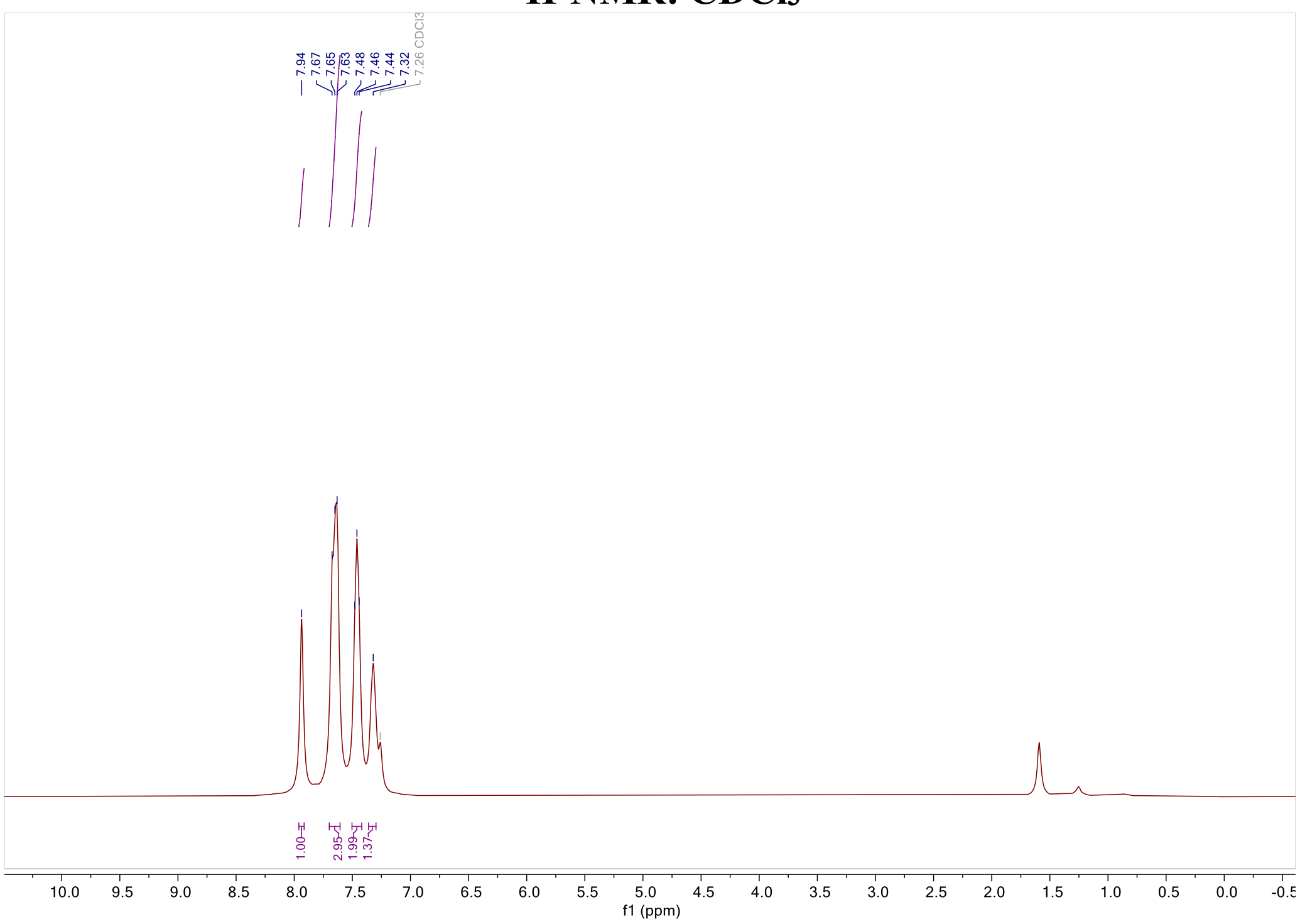

^{13}C-NMR: CDCl3

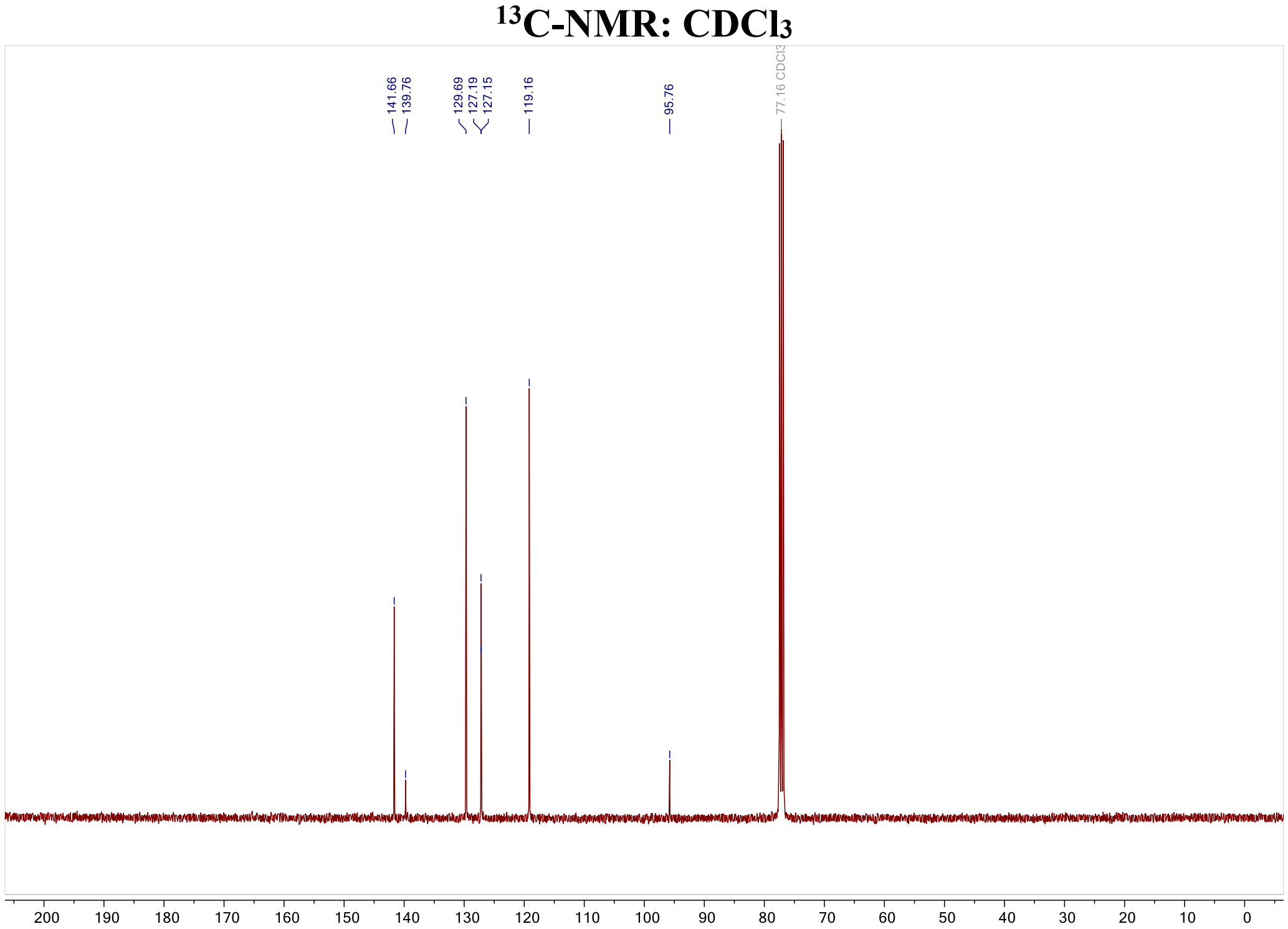

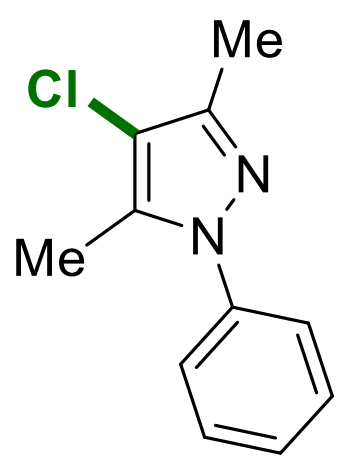

4-chloro-3,5-dimethyl-1-phenyl-1H-pyrazole (15)

^{1}H-NMR: CDCl$_3$

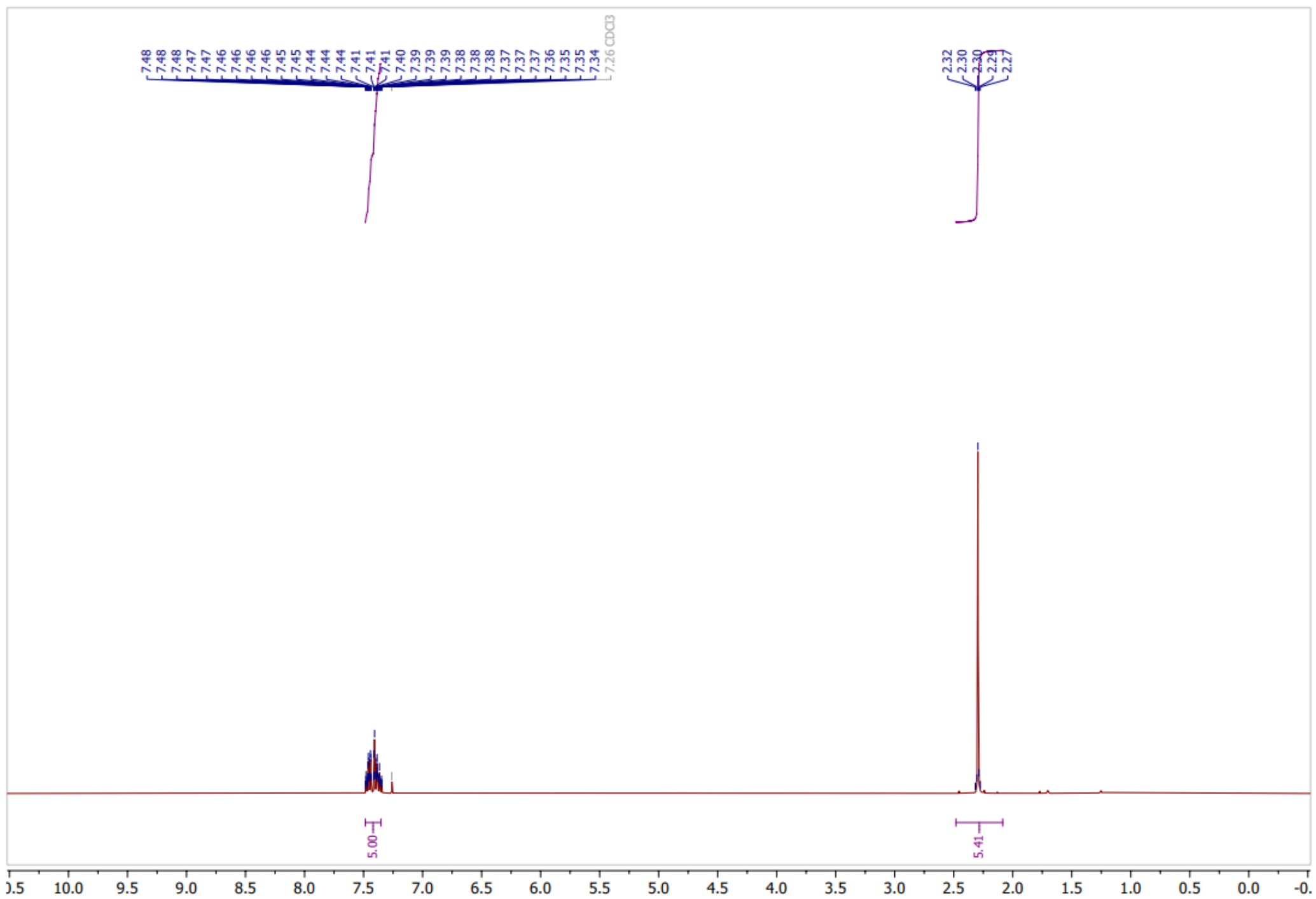

^{13}C-NMR: CDCl$_3$

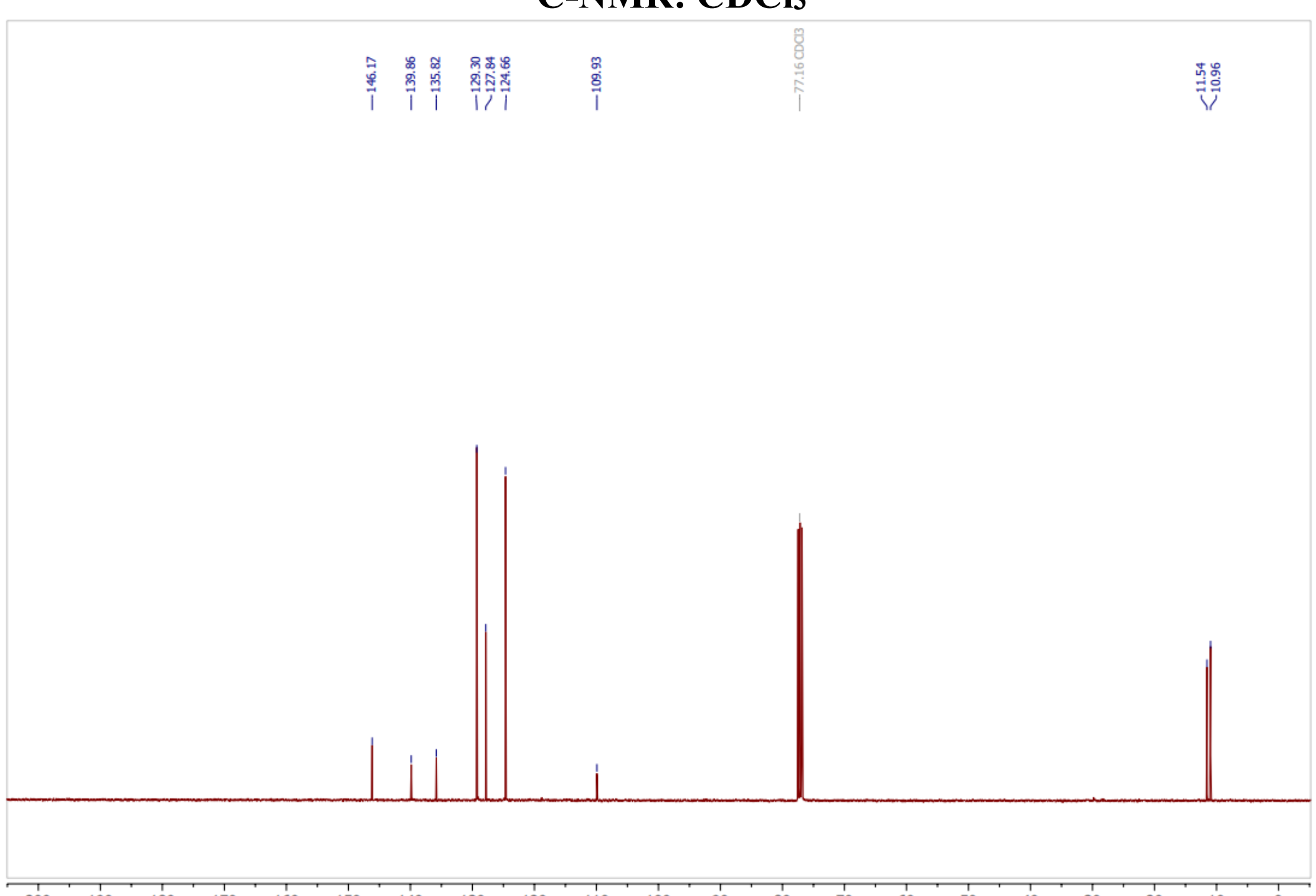

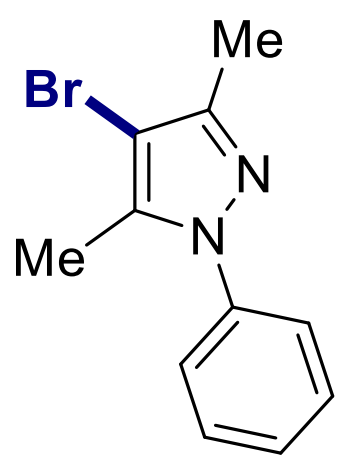

4-chloro-3,5-dimethyl-1-phenyl-1H-pyrazole (16)

^{1}H-NMR: CDCl$_3$

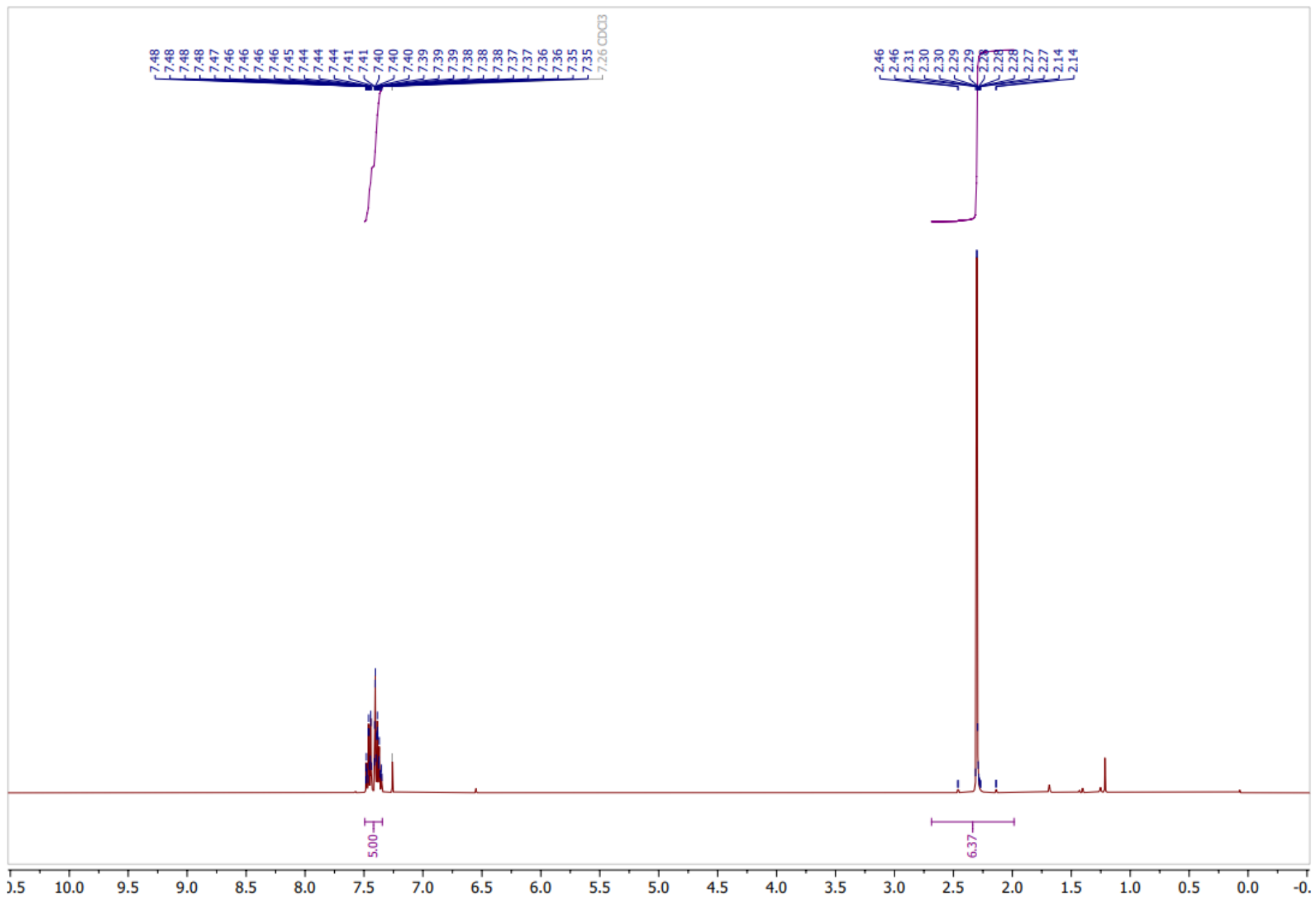

^{13}C-NMR: CDCl$_3$

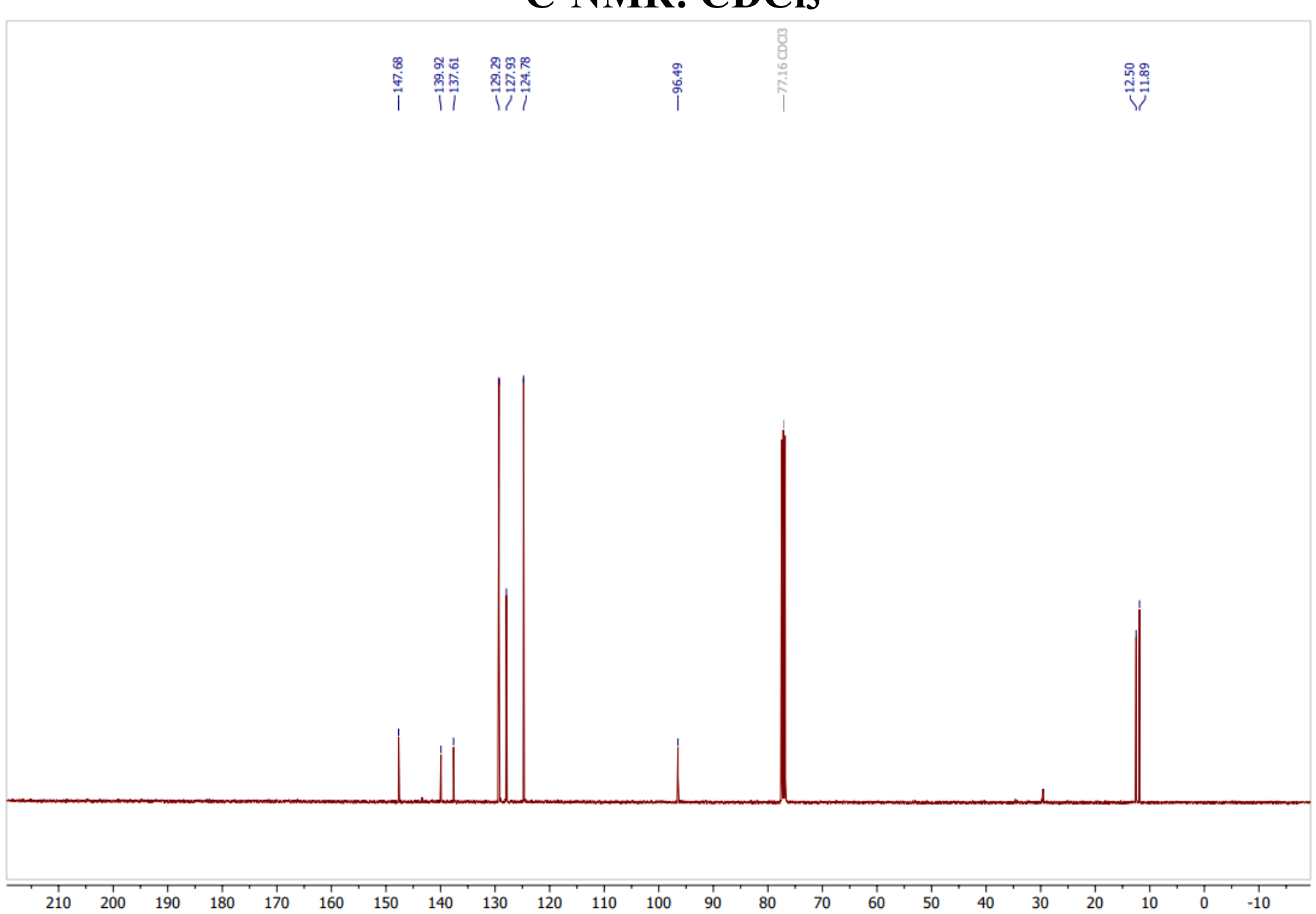

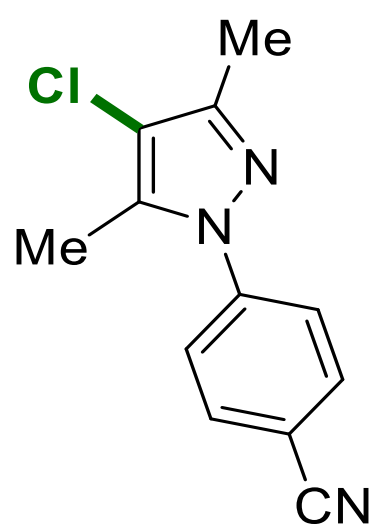

4-(4-chloro-3,5-dimethyl-1H-pyrazol-1-yl)benzonitrile (17)

^{1}H-NMR: CDCl3

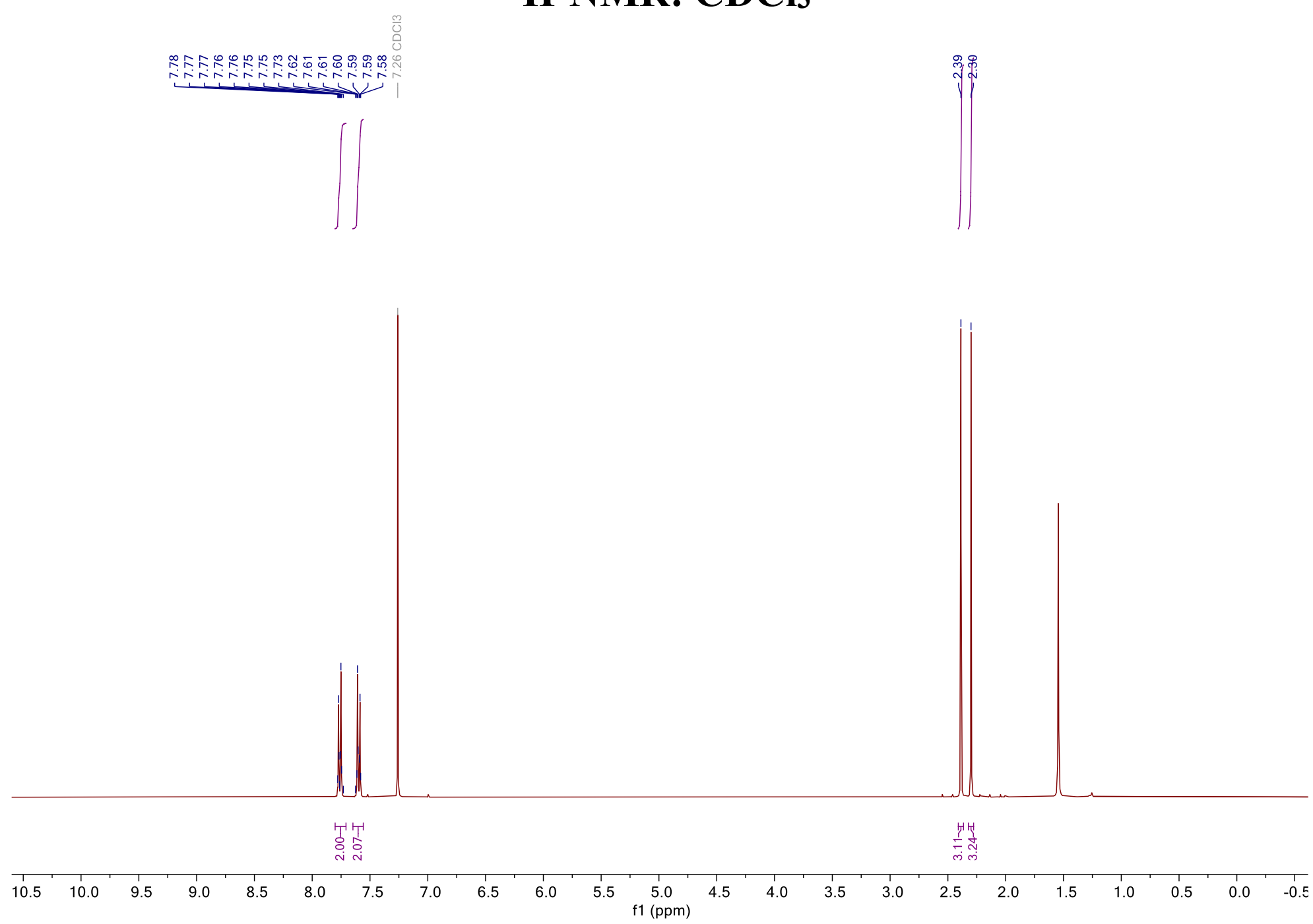

^{13}C-NMR: CDCl3

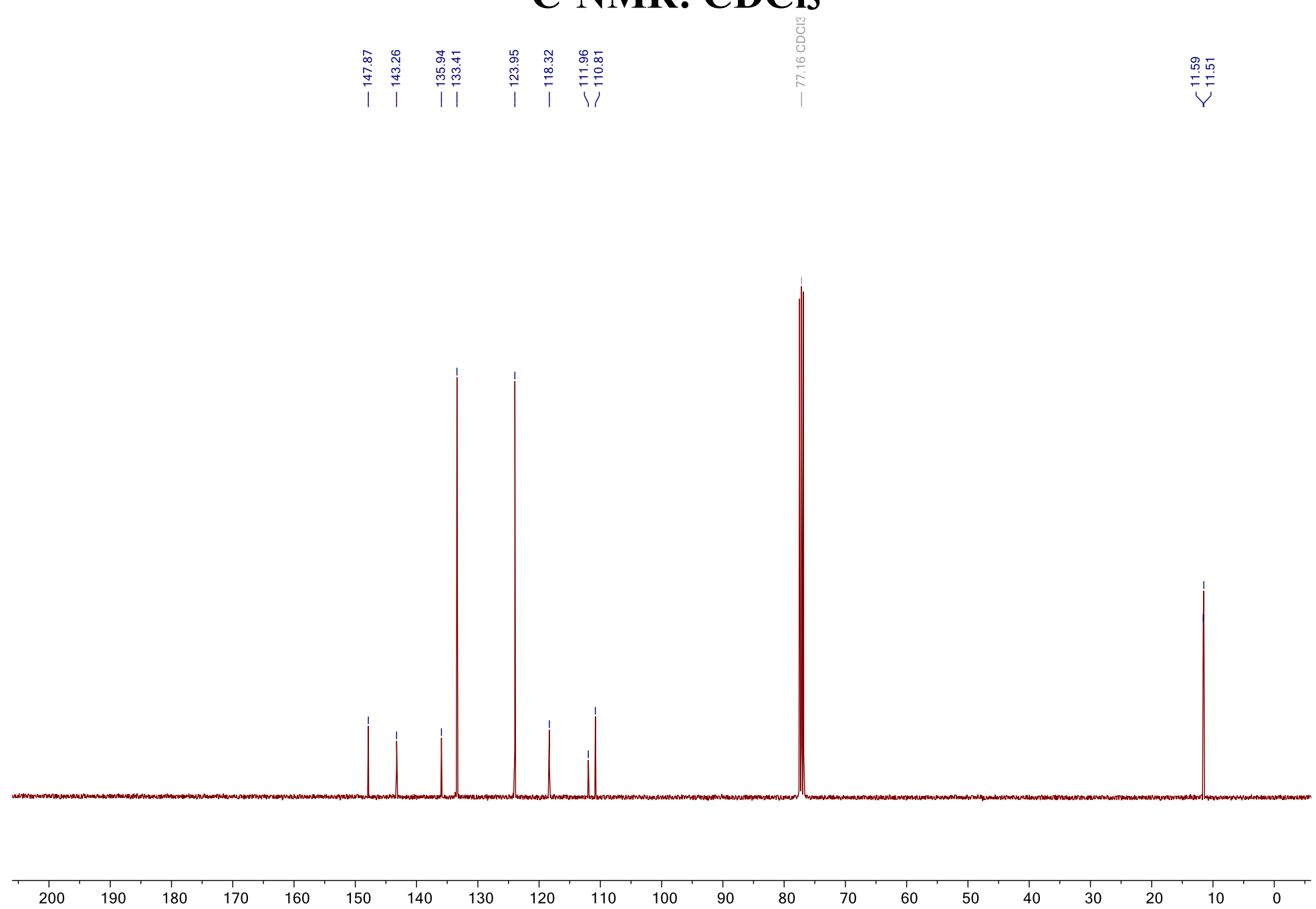

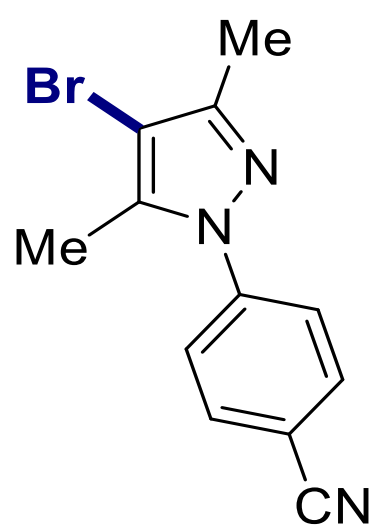

4-(4-chloro-3,5-dimethyl-1H-pyrazol-1-yl)benzonitrile (18)

^{1}H-NMR: $CDCl_3$

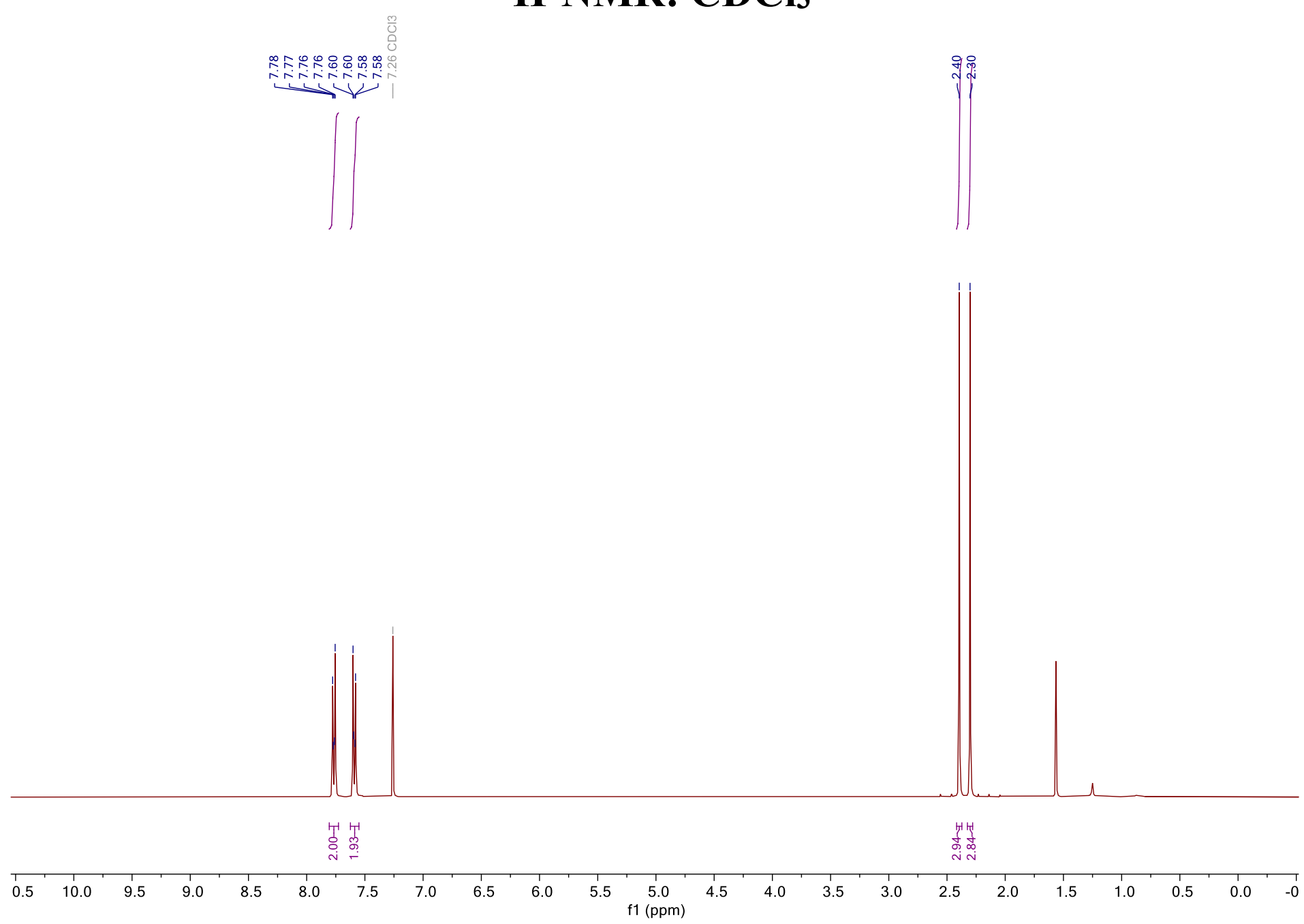

^{13}C-NMR: $CDCl_3$

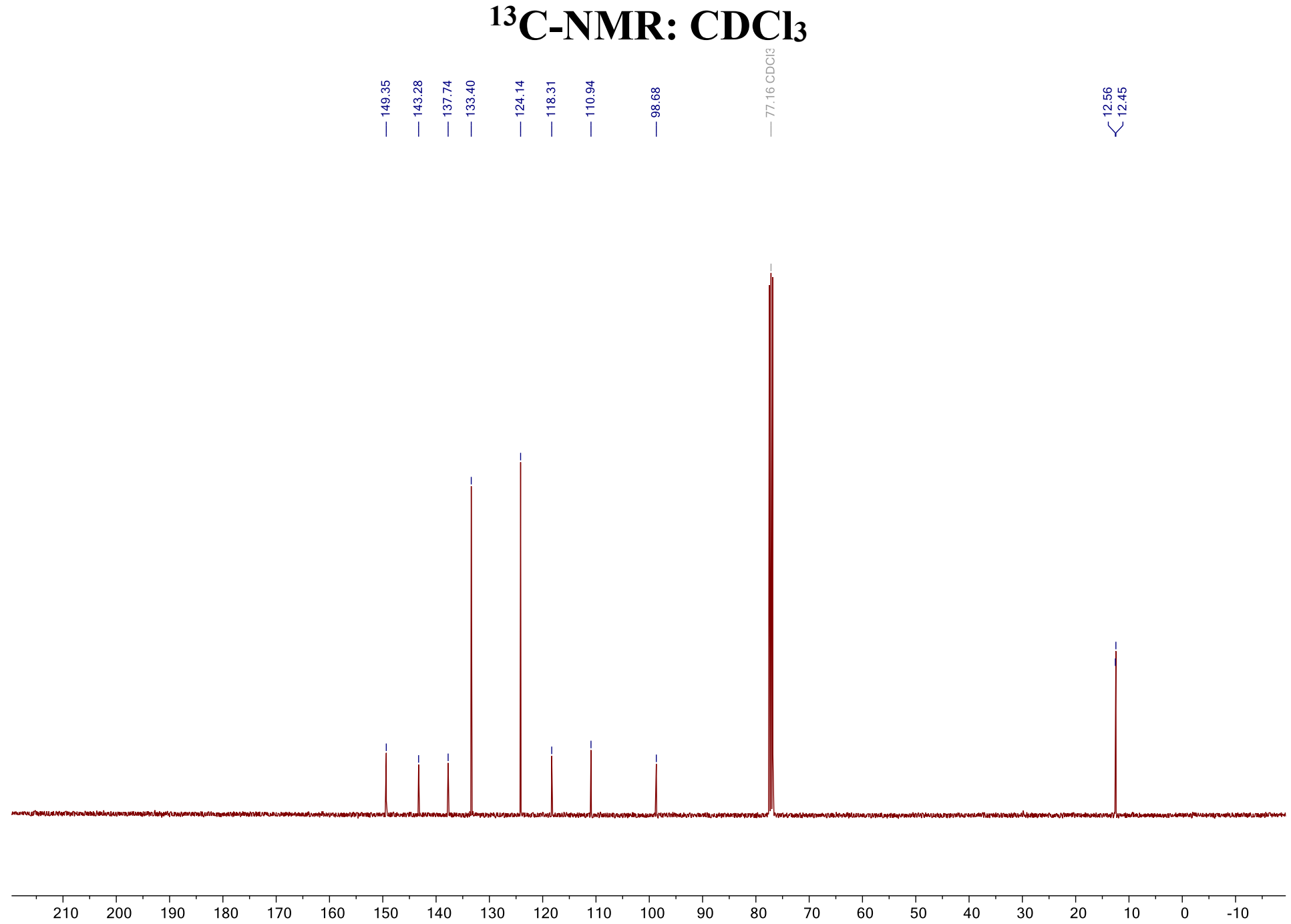

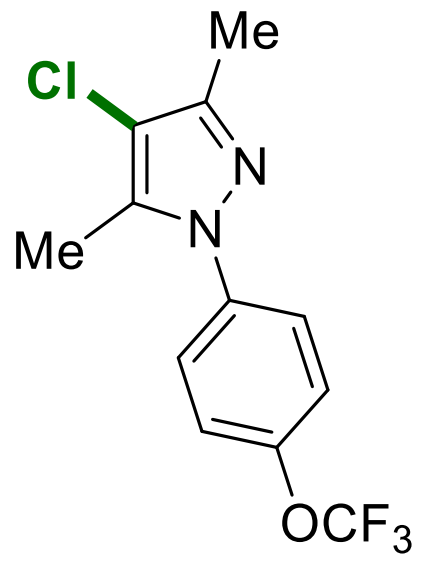

4-chloro-3,5-dimethyl-1-(4-(trifluoromethoxy)phenyl)-1H-pyrazole (19)

^{1}H-NMR: $CDCl_3$

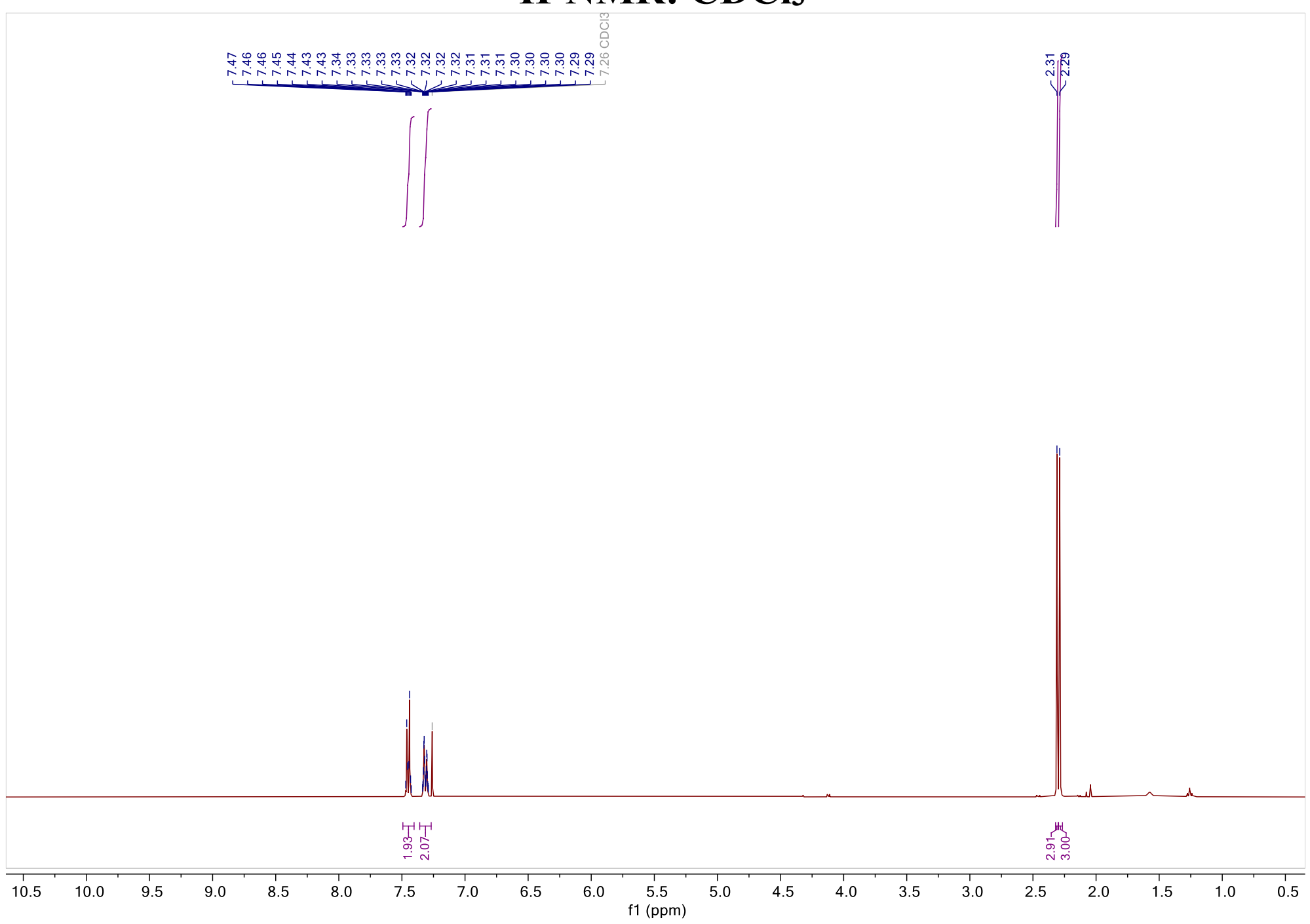

^{13}C-NMR: $CDCl_3$

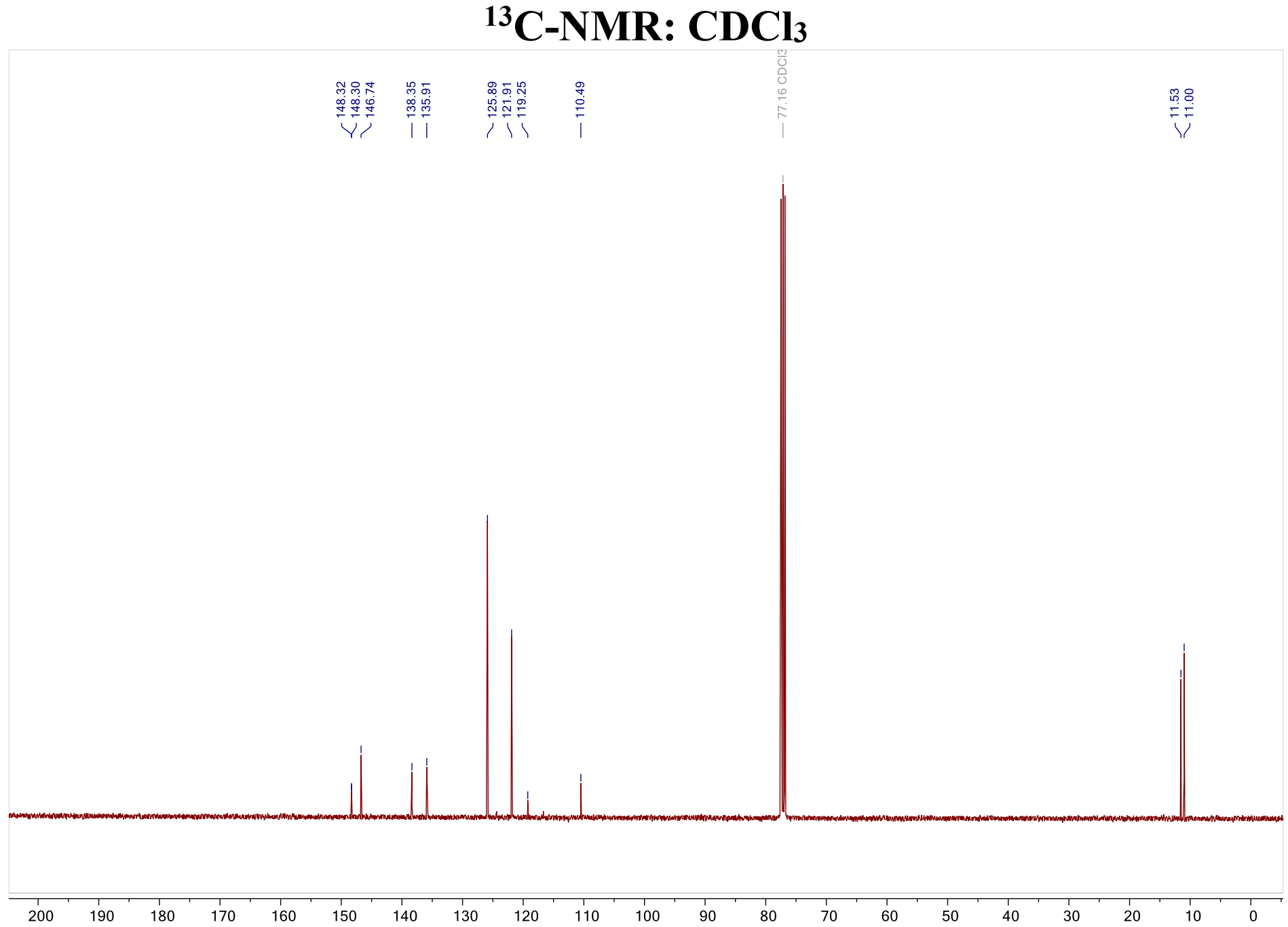

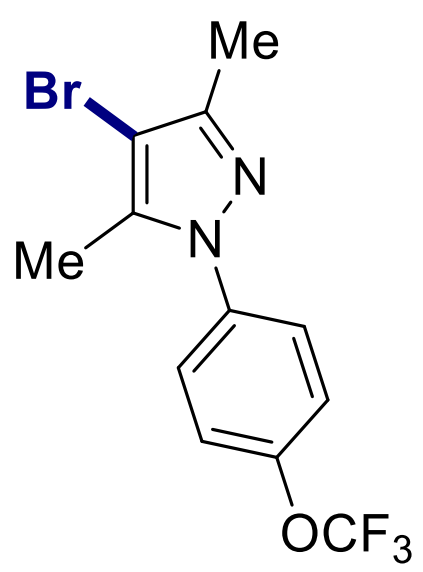

4-chloro-3,5-dimethyl-1-(4-(trifluoromethoxy)phenyl)-1H-pyrazole (20)

^{1}H-NMR: CDCl$_3$

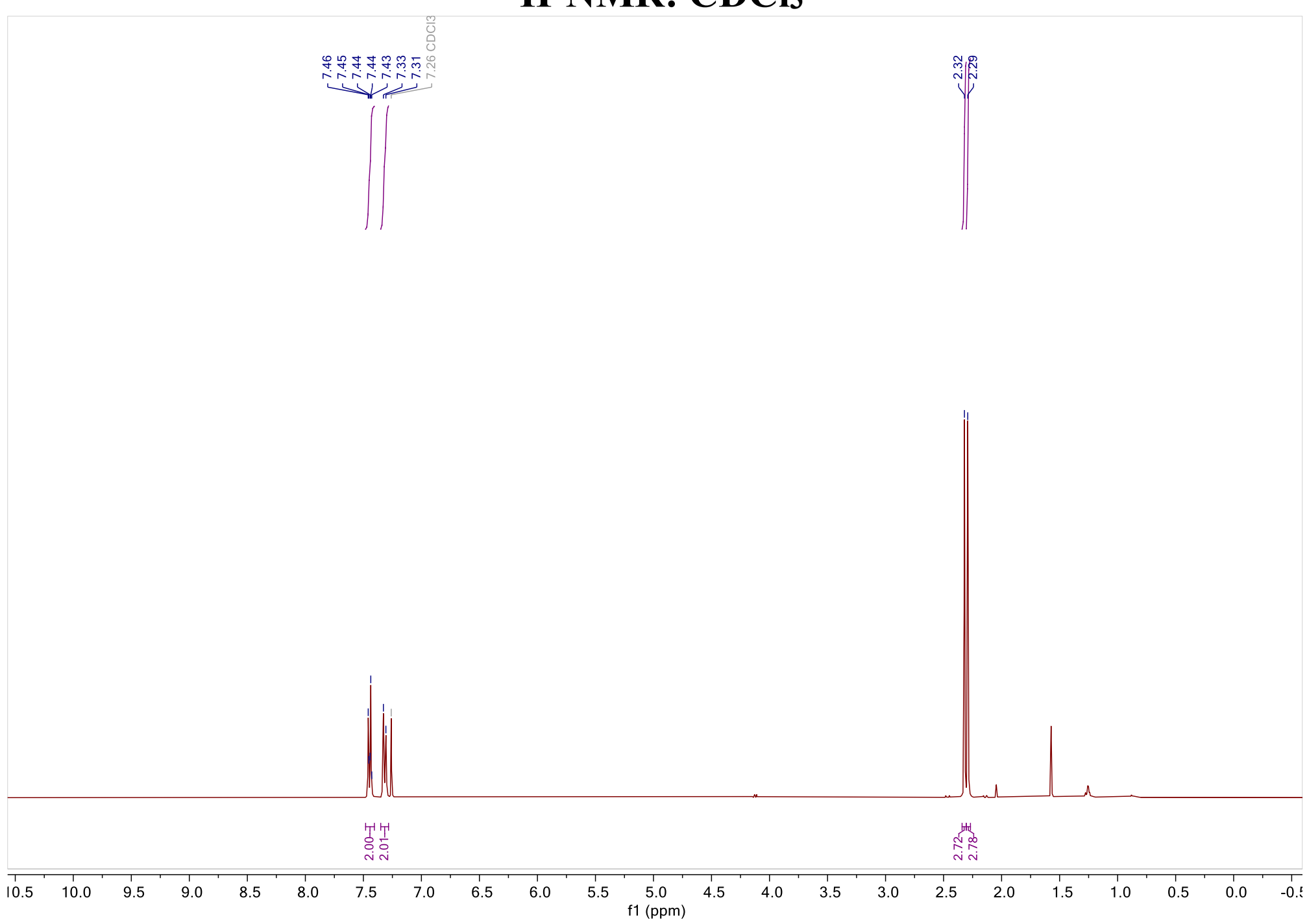

^{13}C-NMR: CDCl$_3$

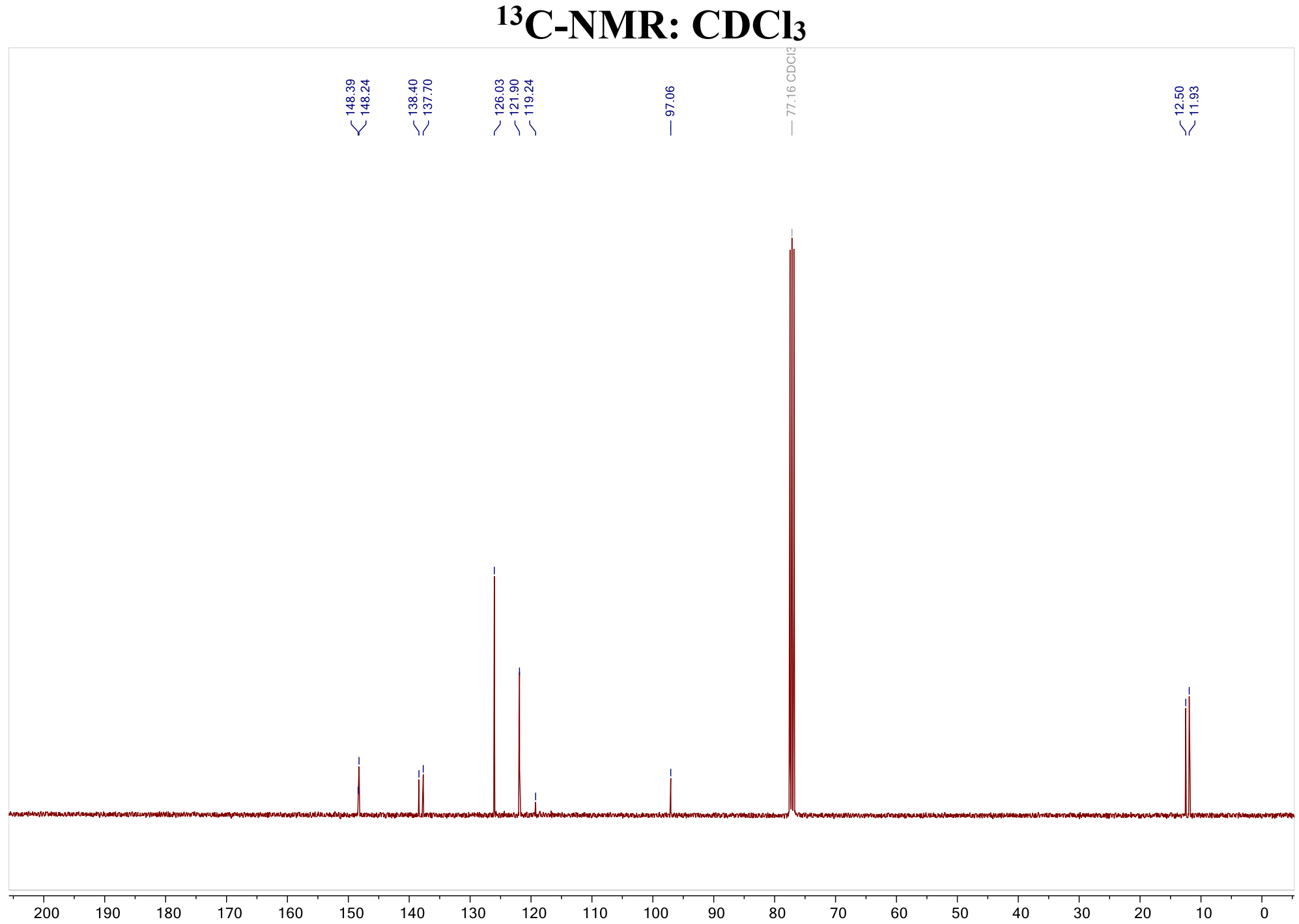

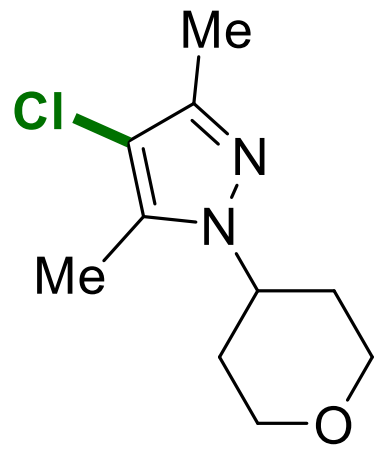

4-chloro-3,5-dimethyl-1-(tetrahydro-2H-pyran-4-yl)-1H-pyrazole (21)

^{1}H-NMR: $CDCl_3$

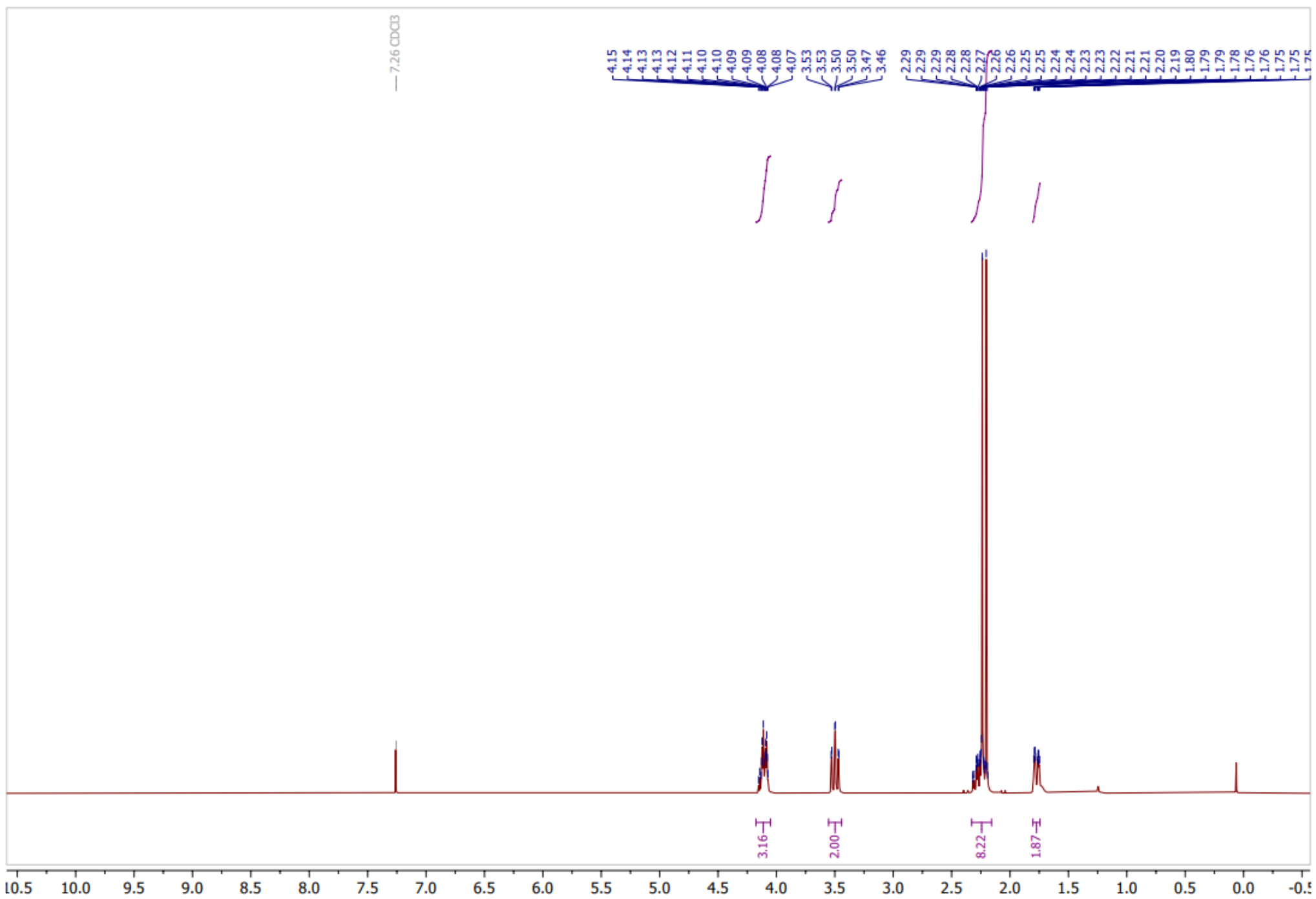

^{13}C-NMR: $CDCl_3$

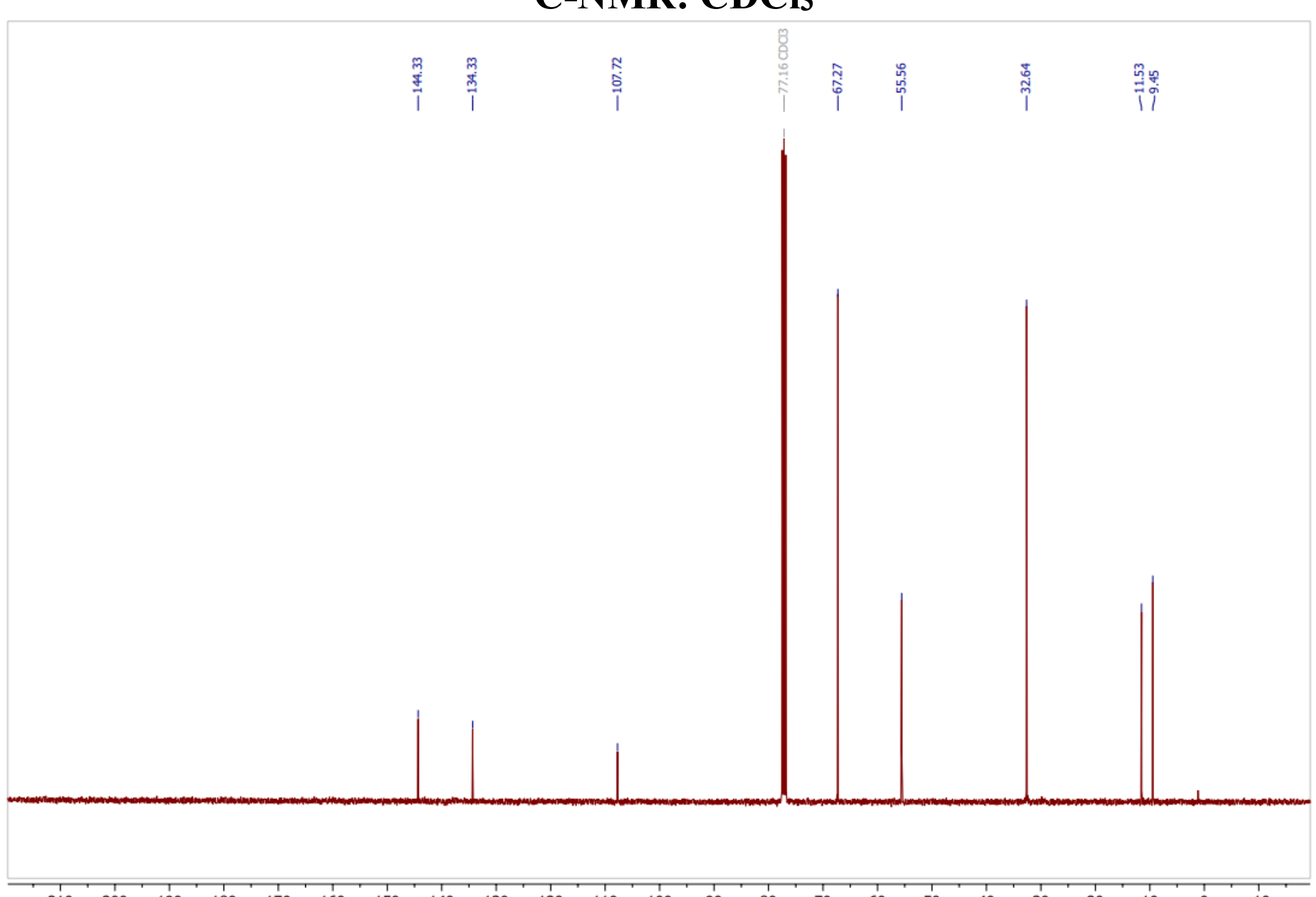

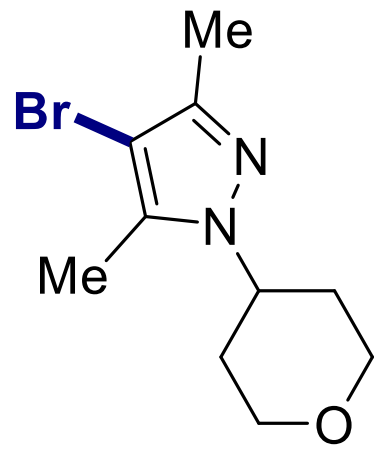

4-chloro-3,5-dimethyl-1-(tetrahydro-2H-pyran-4-yl)-1H-pyrazole (22)

^{1}H-NMR: $CDCl_3$

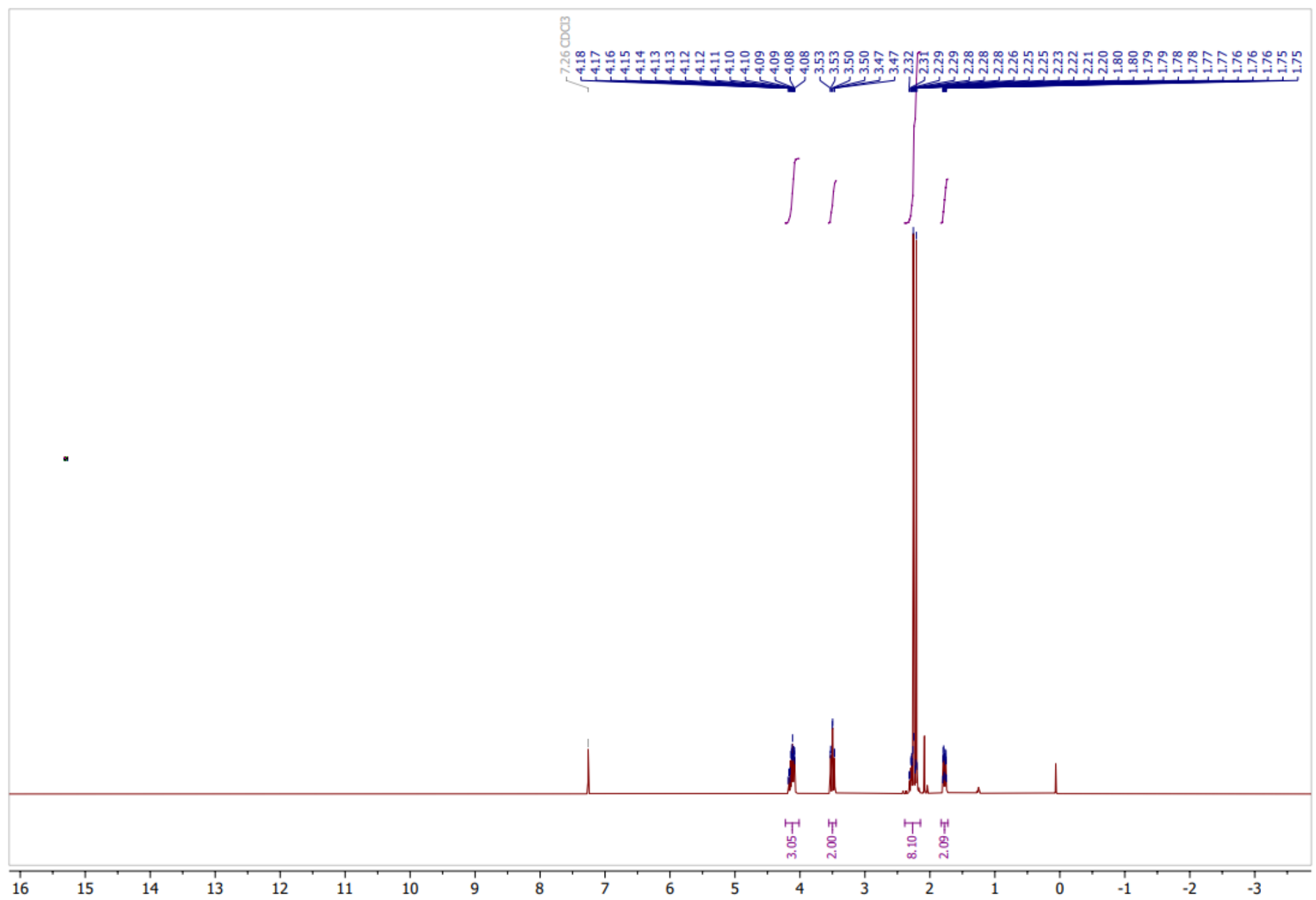

^{13}C-NMR: $CDCl_3$

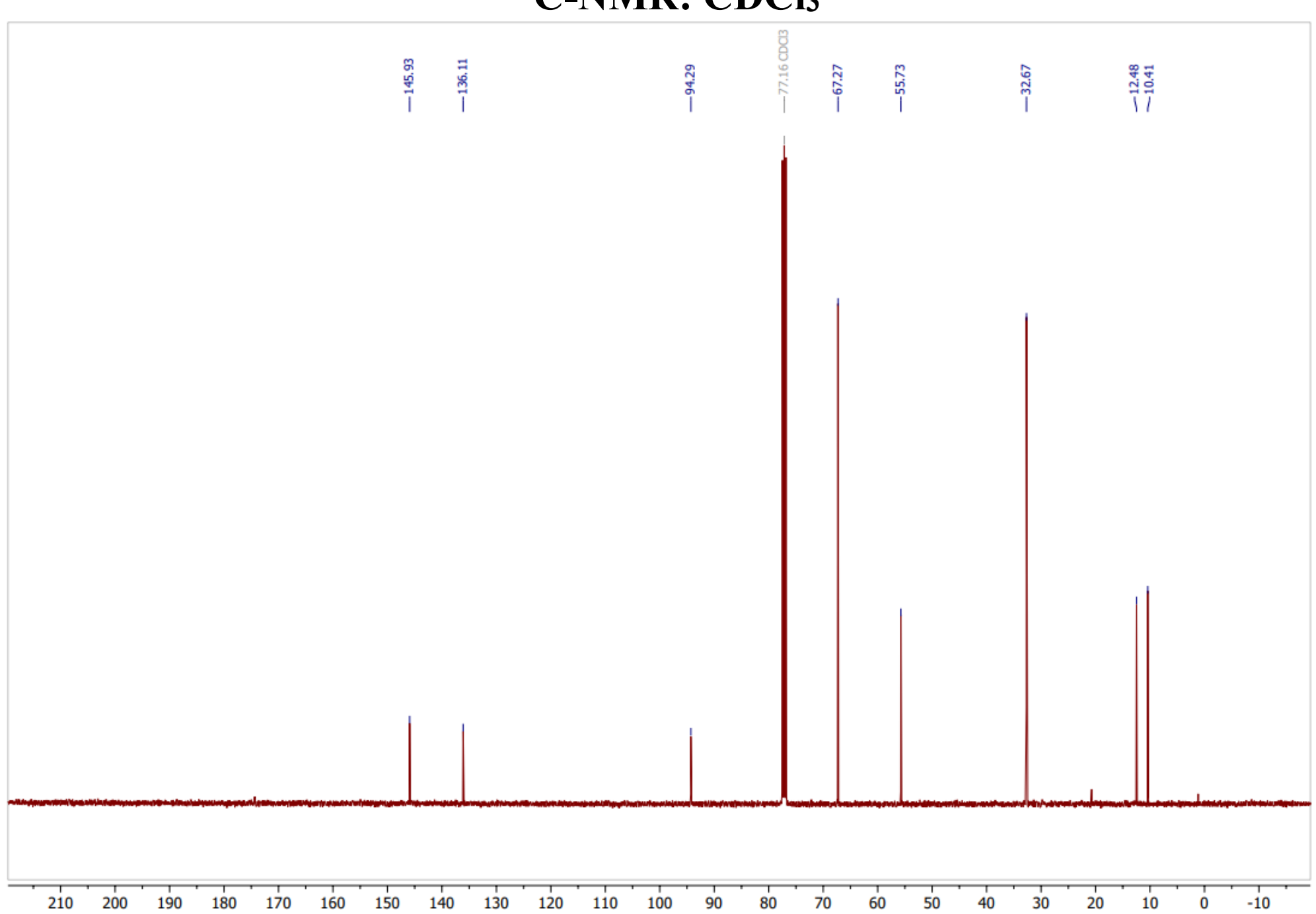

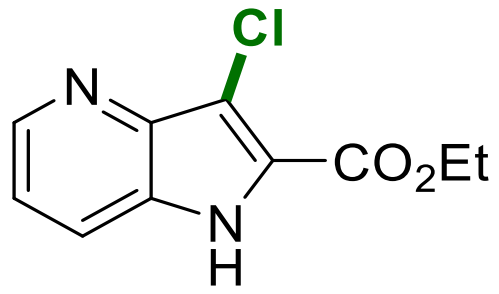

ethyl 3-chloro-1H-pyrrolo[3,2-b]pyridine-2-carboxylate (24)

^{1}H-NMR: DMSO-D$_6$

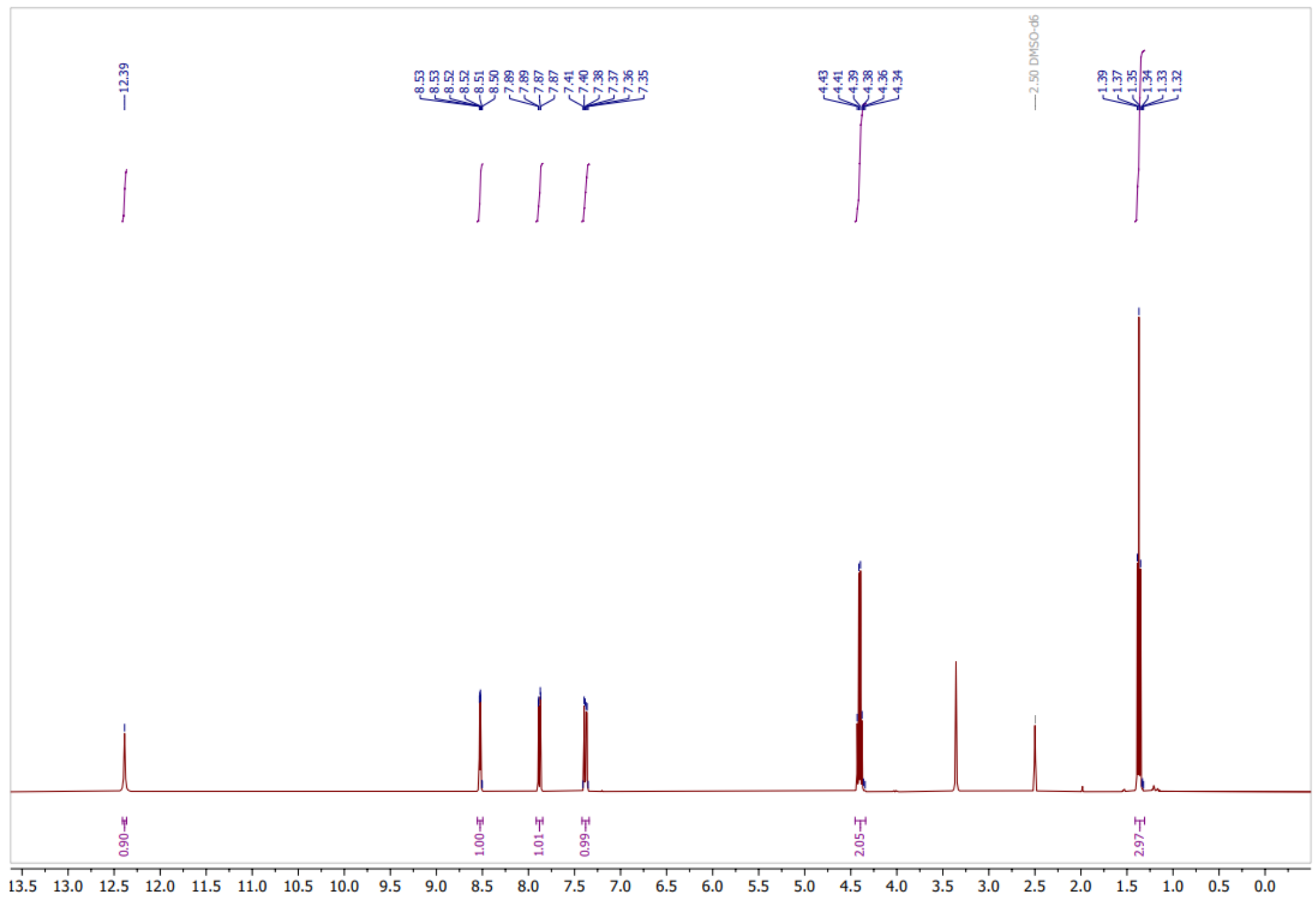

^{13}C-NMR: DMSO-D$_6$

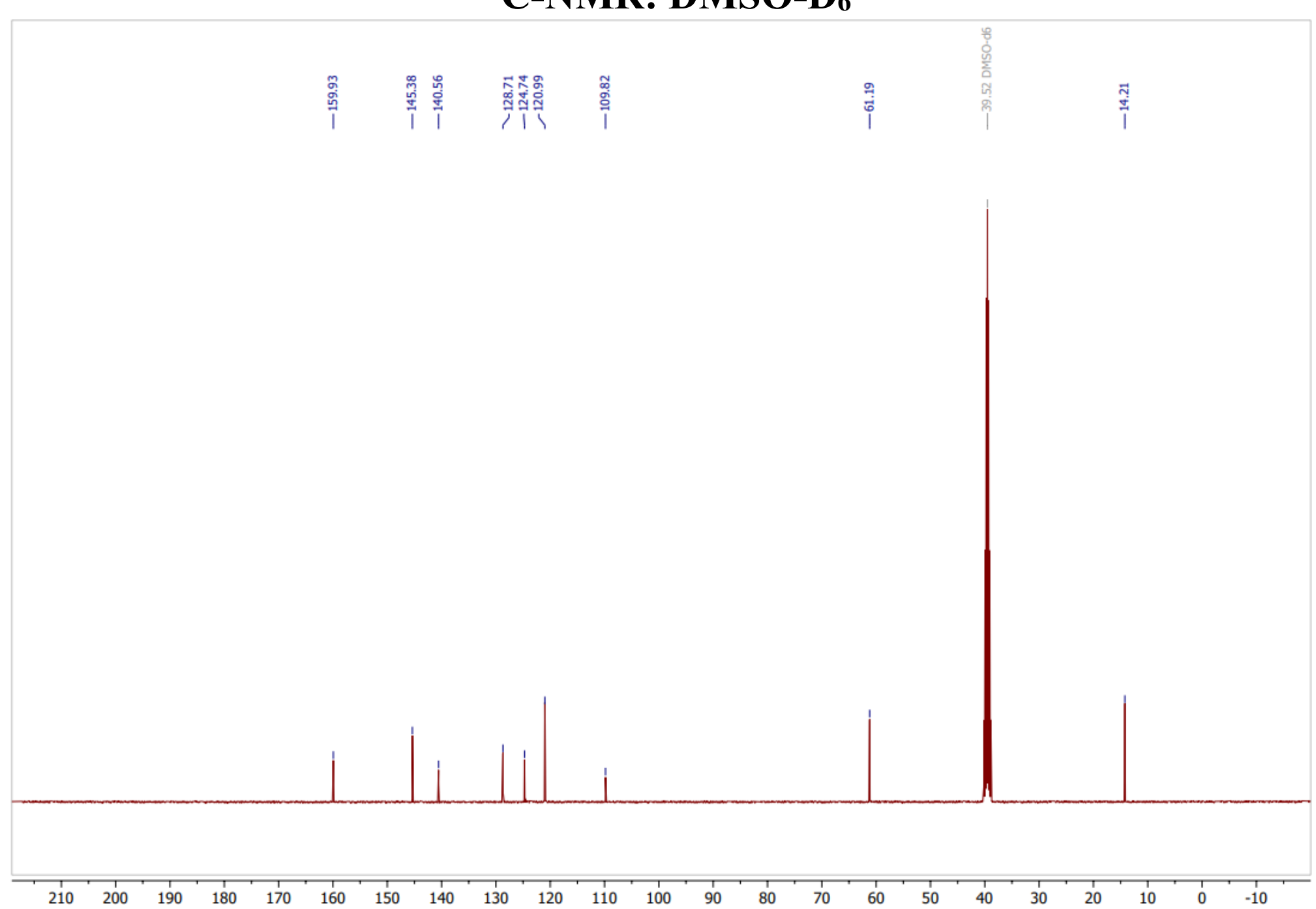

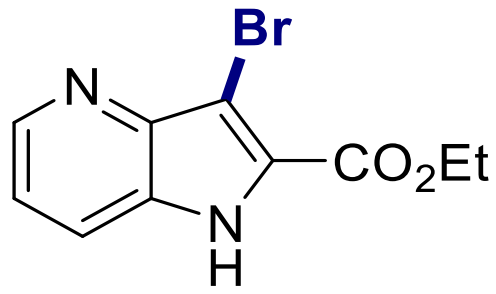

ethyl 3-bromo-1H-pyrrolo[3,2-b]pyridine-2-carboxylate (25)

^{1}H-NMR: DMSO-D$_6$

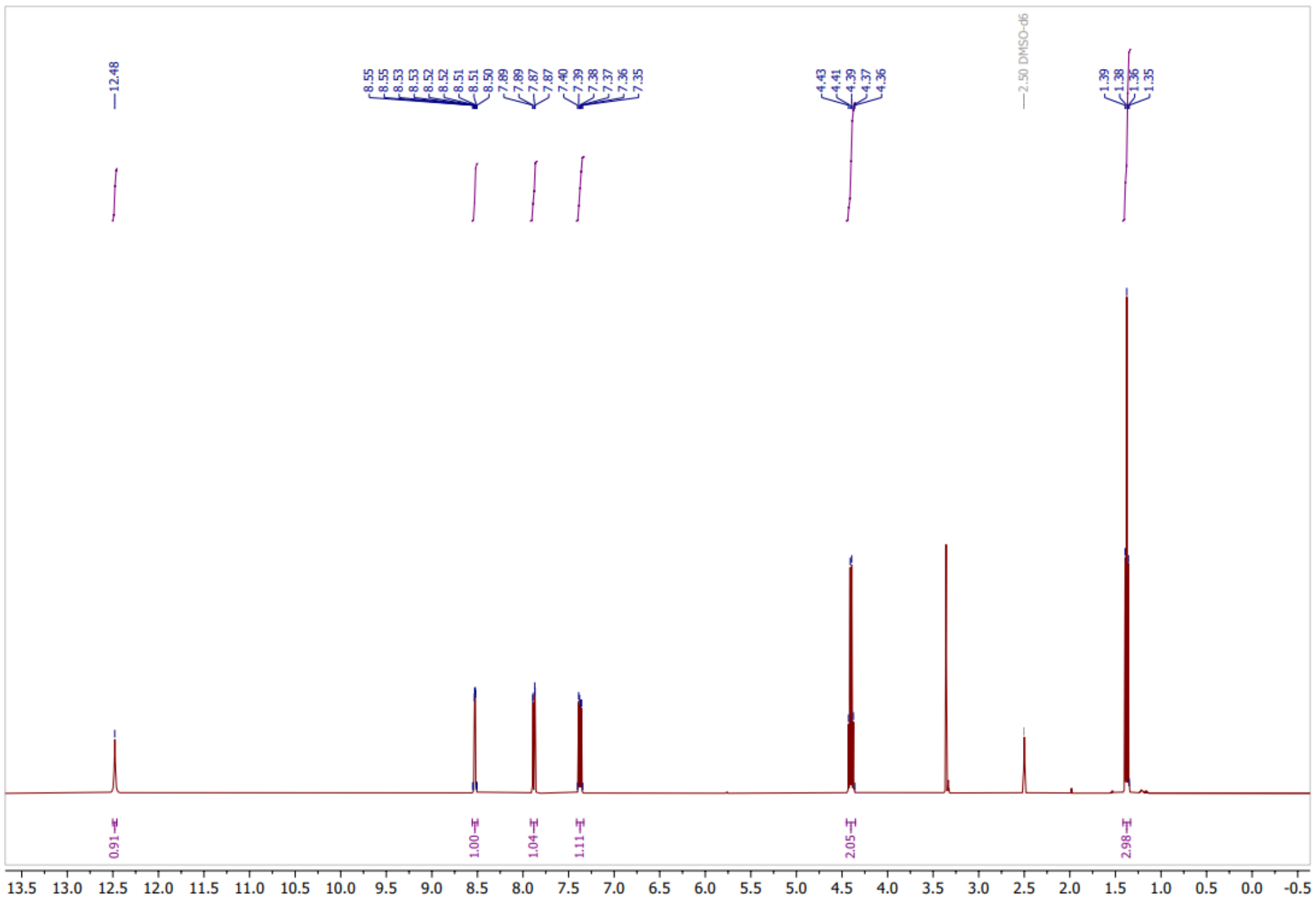

^{13}C-NMR: DMSO-D$_6$

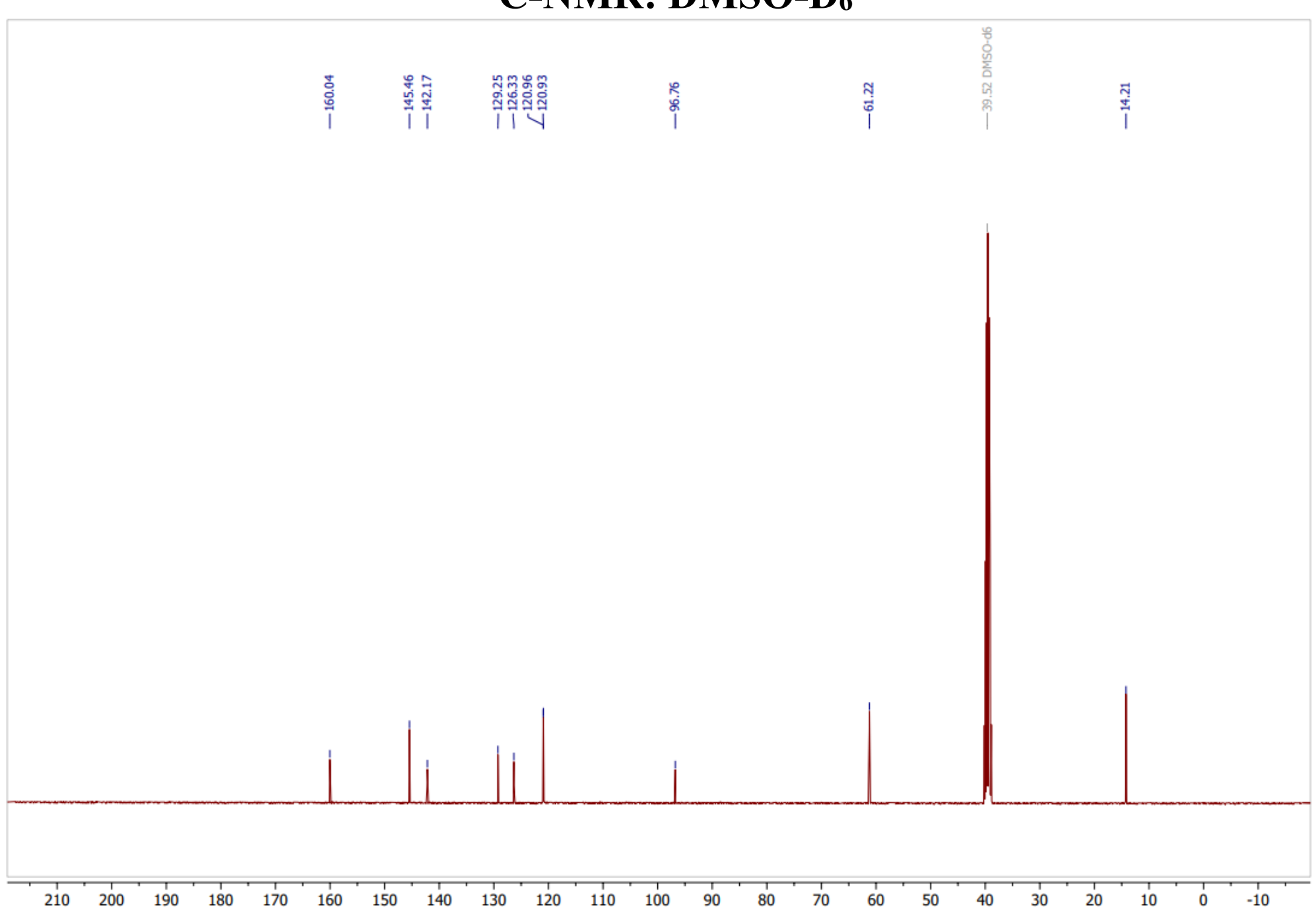

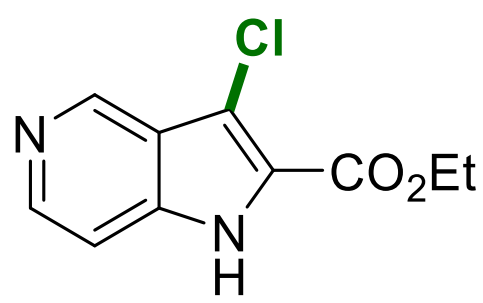

ethyl 3-chloro-1H-pyrrolo[3,2-c]pyridine-2-carboxylate (26)

^{1}H-NMR: DMSO-D$_6$

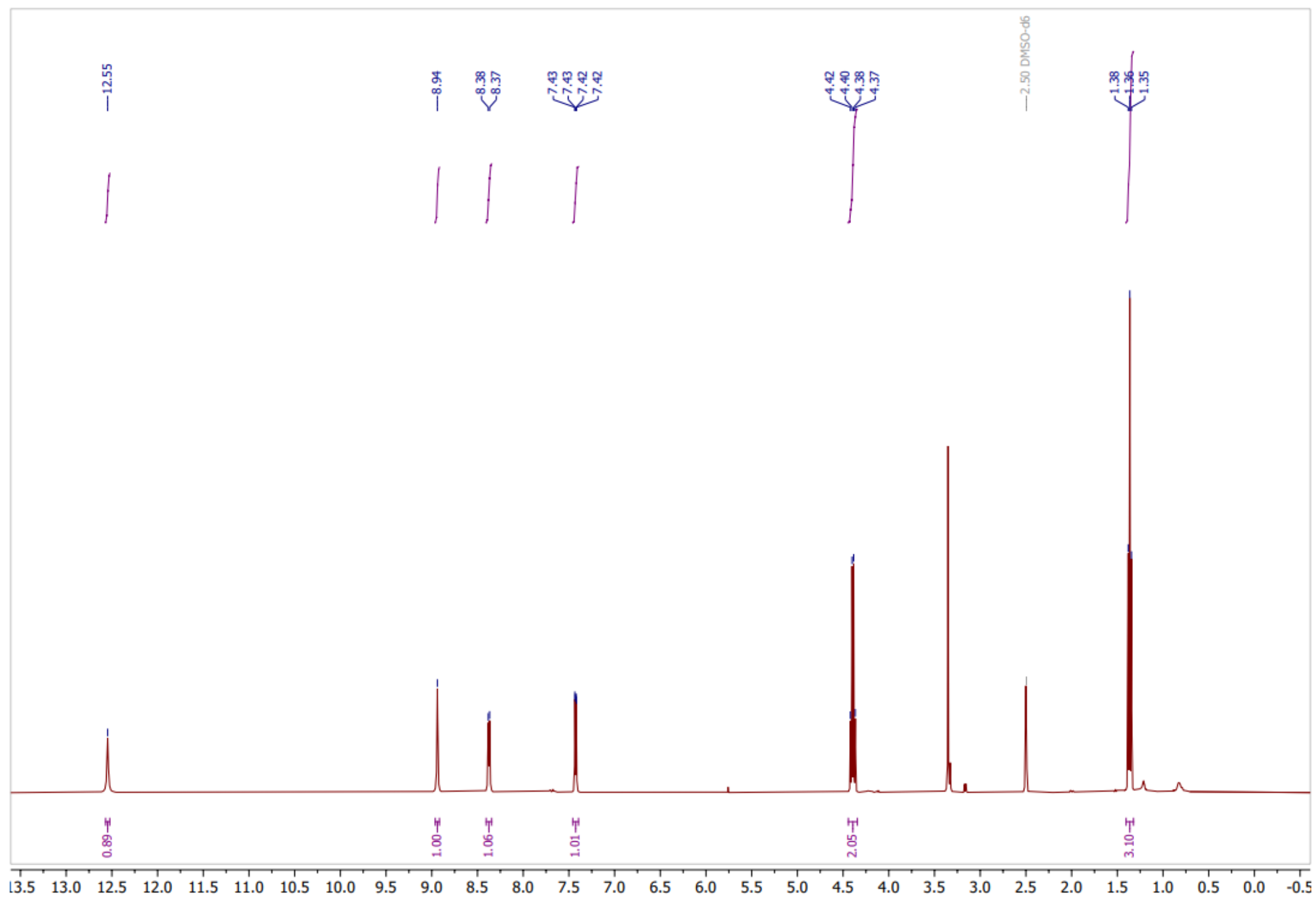

^{13}C-NMR: DMSO-D$_6$

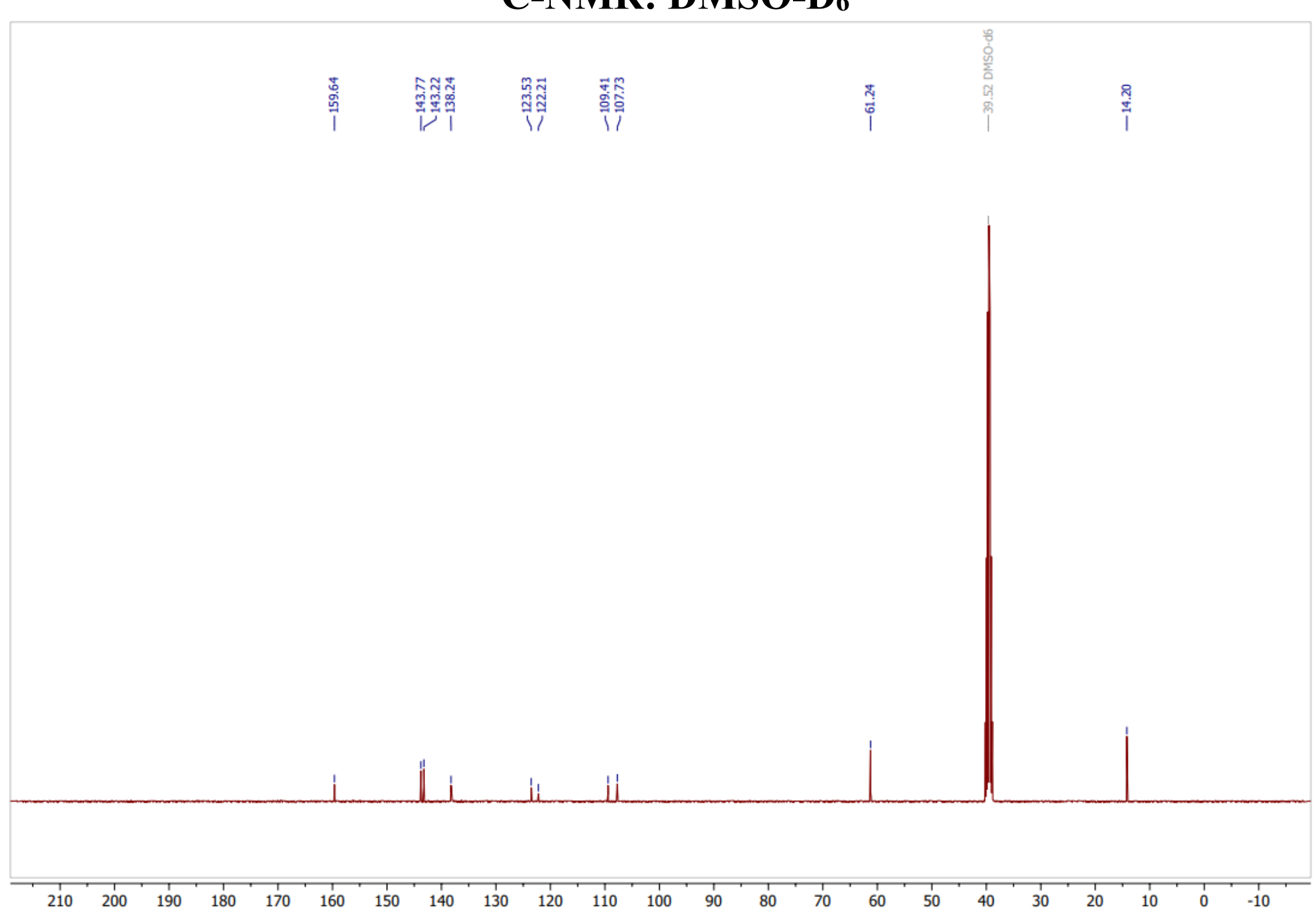

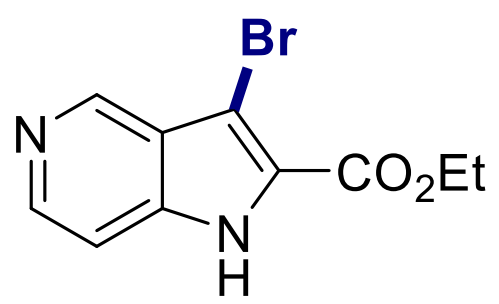

ethyl 3-chloro-1H-pyrrolo[3,2-c]pyridine-2-carboxylate (27)

^{1}H-NMR: DMSO-D$_6$

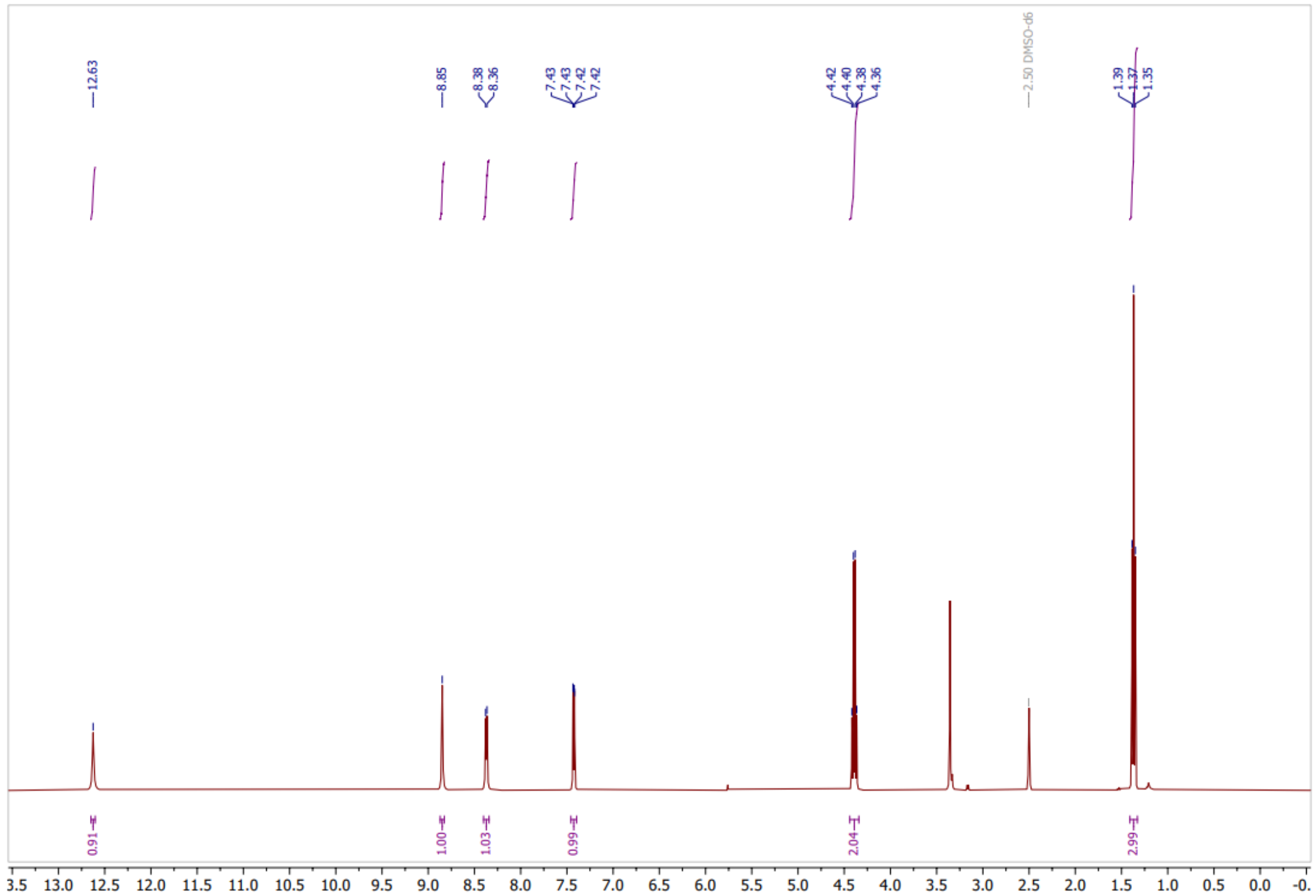

^{13}C-NMR: DMSO-D$_6$

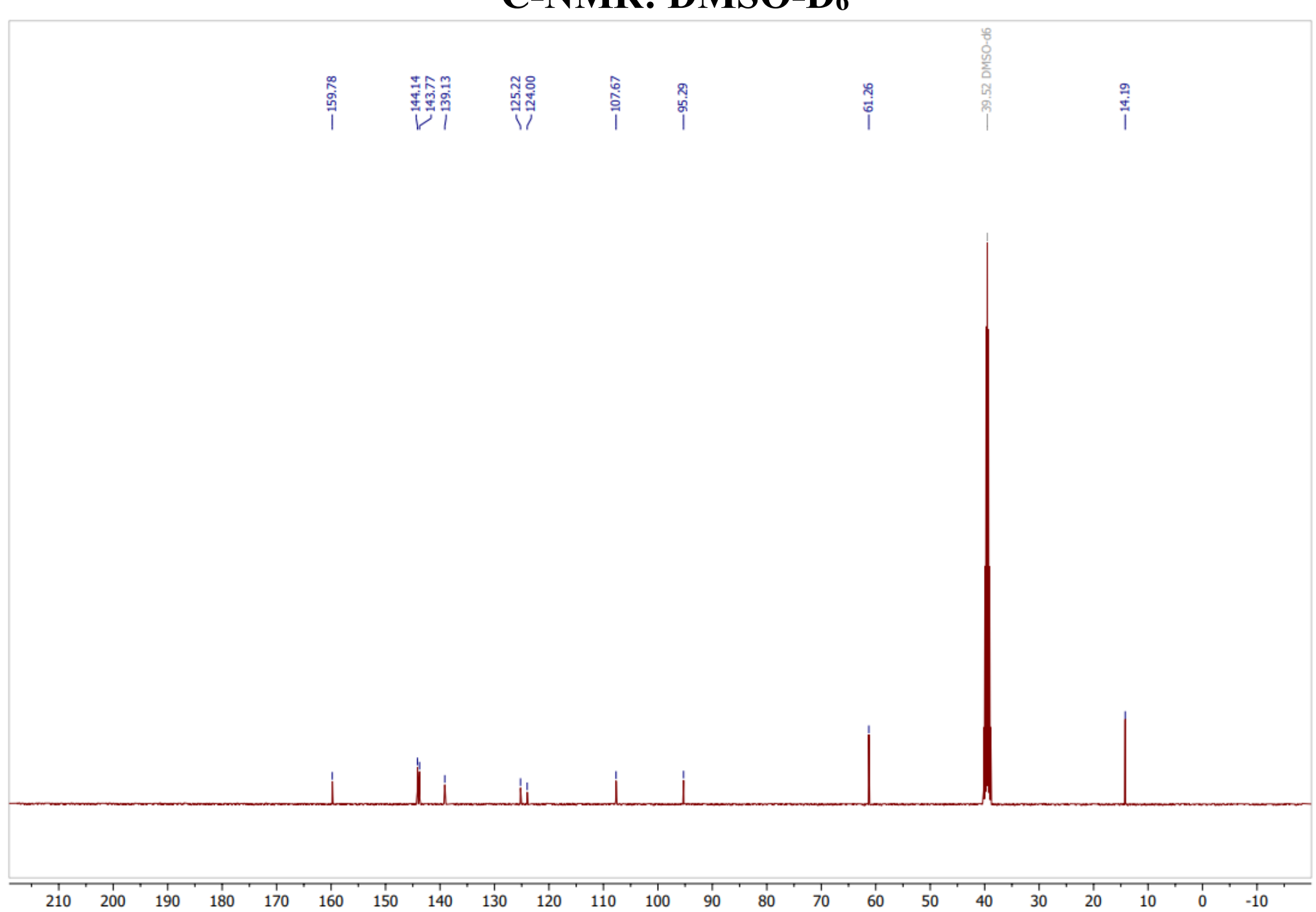

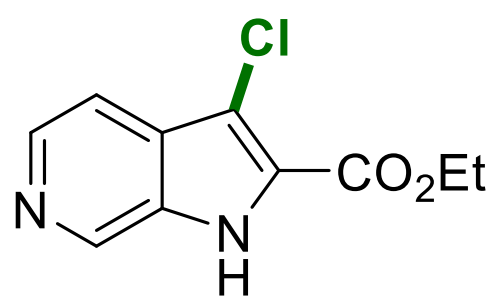

ethyl 3-chloro-1H-pyrrolo[2,3-c]pyridine-2-carboxylate (28)

^{1}H-NMR: DMSO-D$_6$

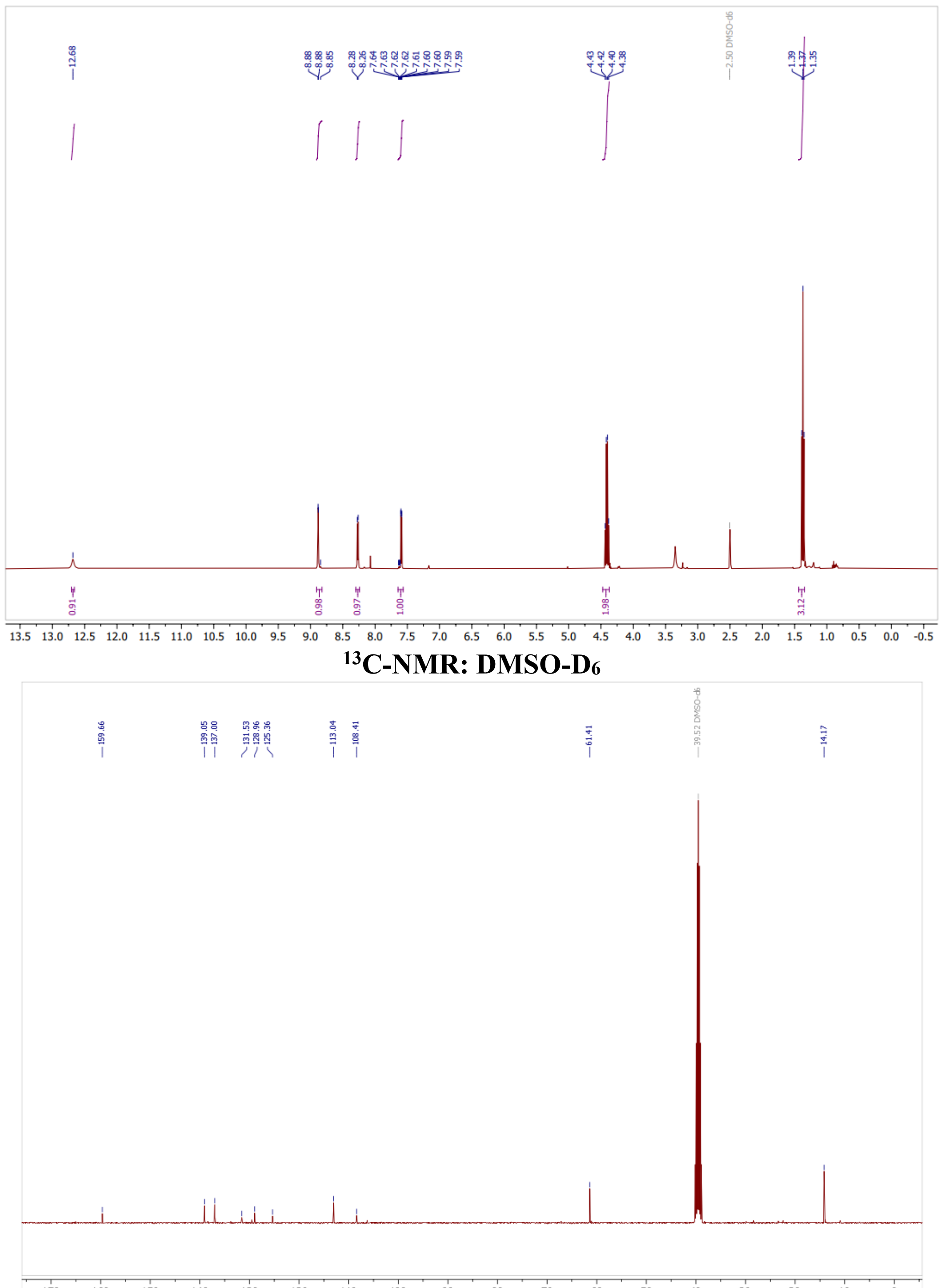

^{13}C-NMR: DMSO-D$_6$

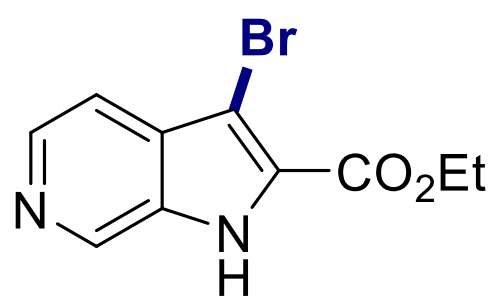

ethyl 3-bromo-1H-pyrrolo[2,3-c]pyridine-2-carboxylate (29)

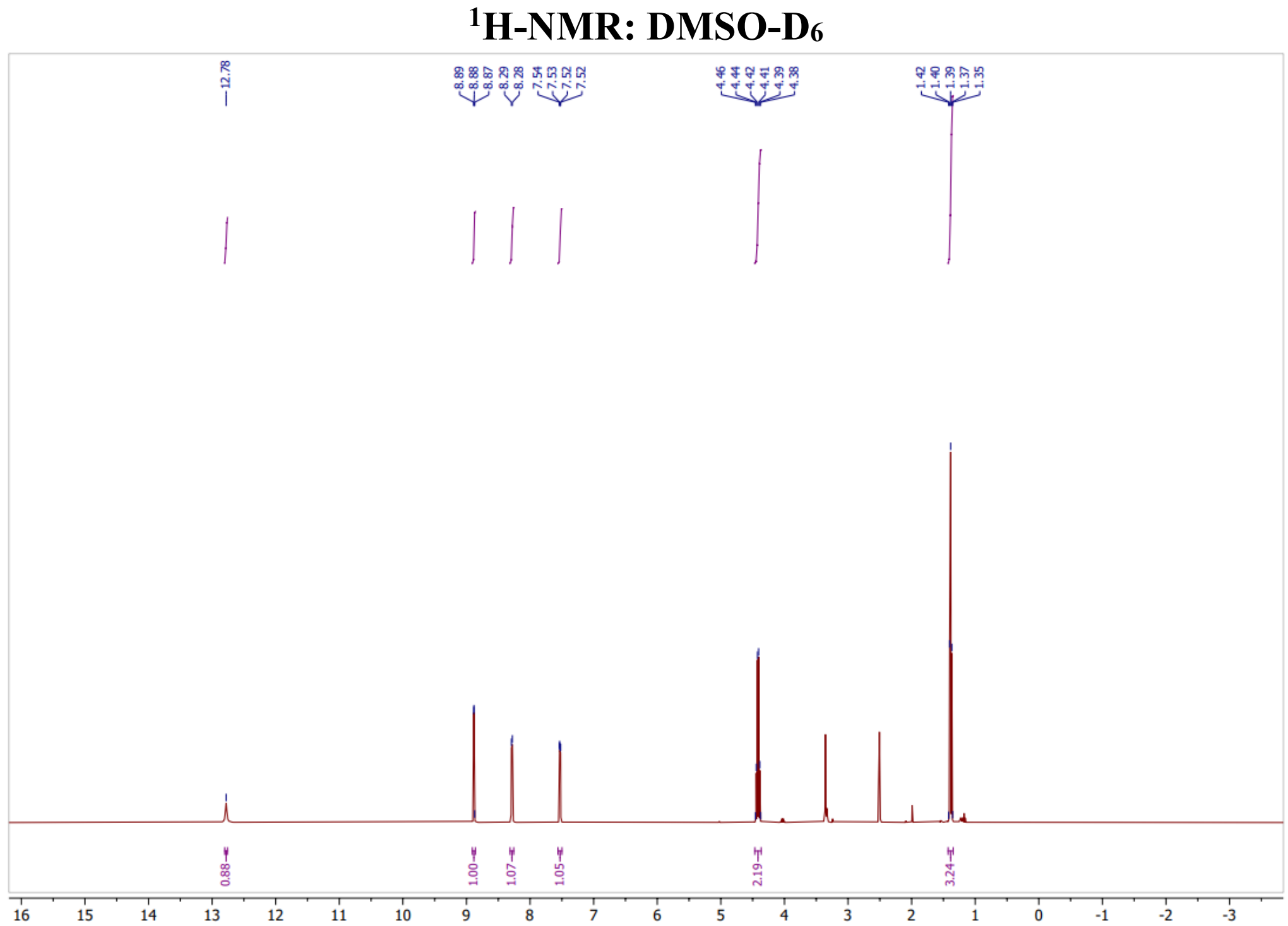

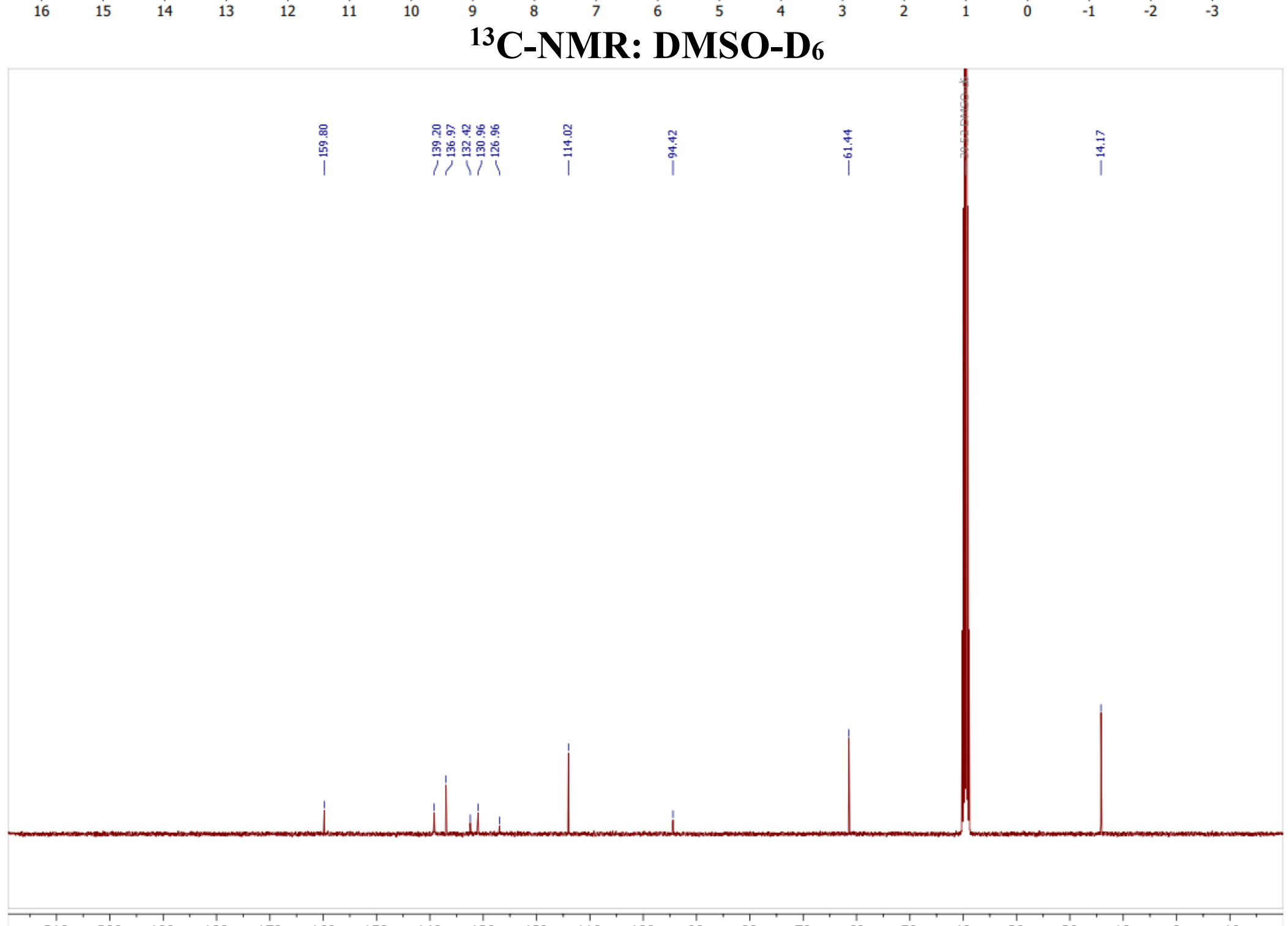

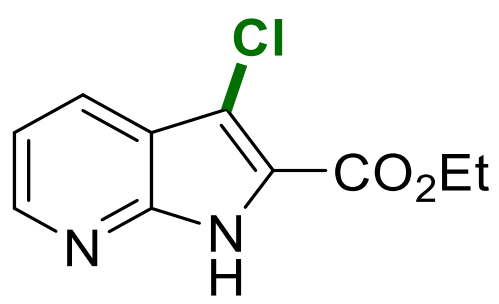

ethyl 3-chloro-1H-pyrrolo[2,3-b]pyridine-2-carboxylate (30)

48% Chlorinated Product + 48% SM

NMR yield referenced to 1,3,5-trimethoxybenzene

^{1}H-NMR: DMSO-d$_6$

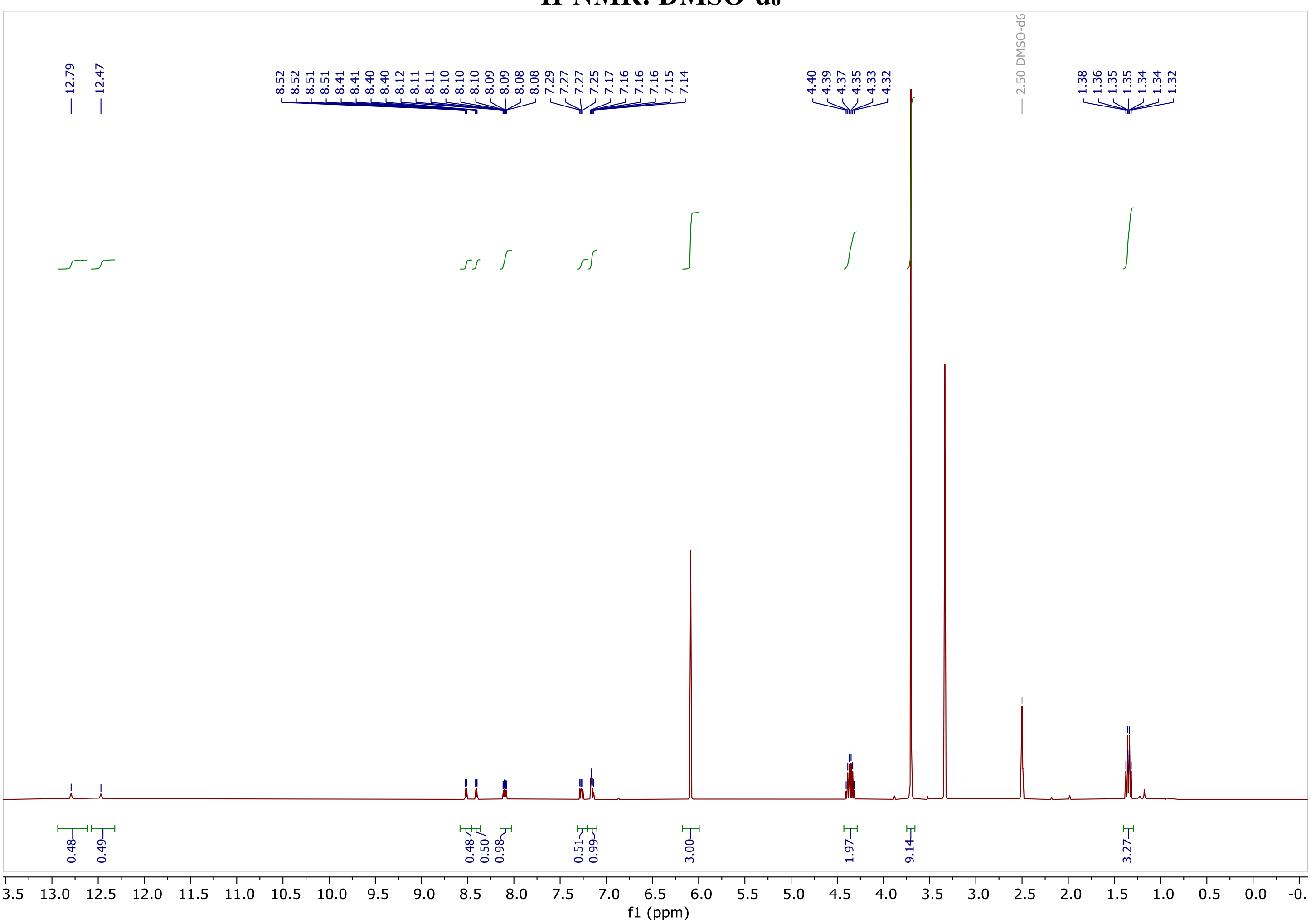

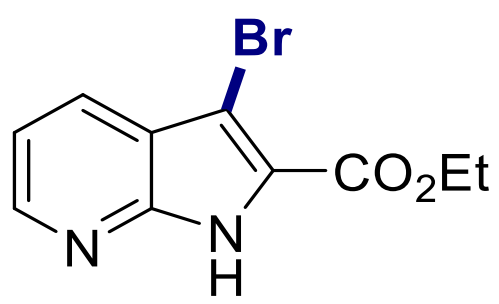

ethyl 3-chloro-1H-pyrrolo[2,3-b]pyridine-2-carboxylate (31)

65% Brominated Product + 30% SM

NMR yield referenced to 1,3,5-trimethoxybenzene

^{1}H-NMR: DMSO-d$_6$

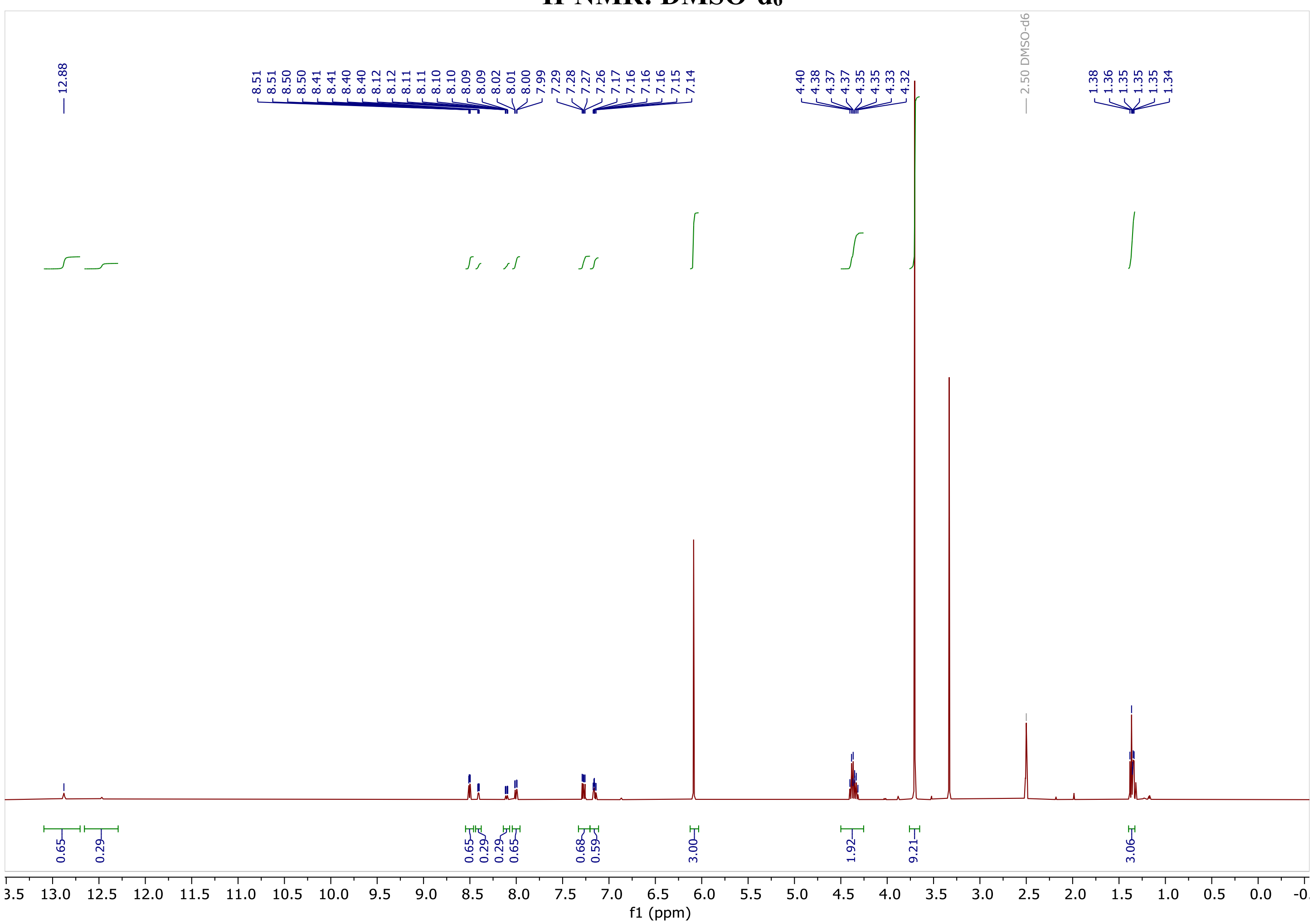

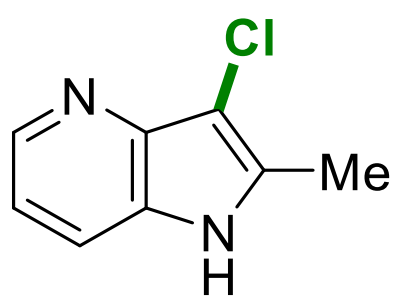

3-chloro-2-methyl-1H-pyrrolo[3,2-b]pyridine (32)

^{1}H-NMR: DMSO-D6

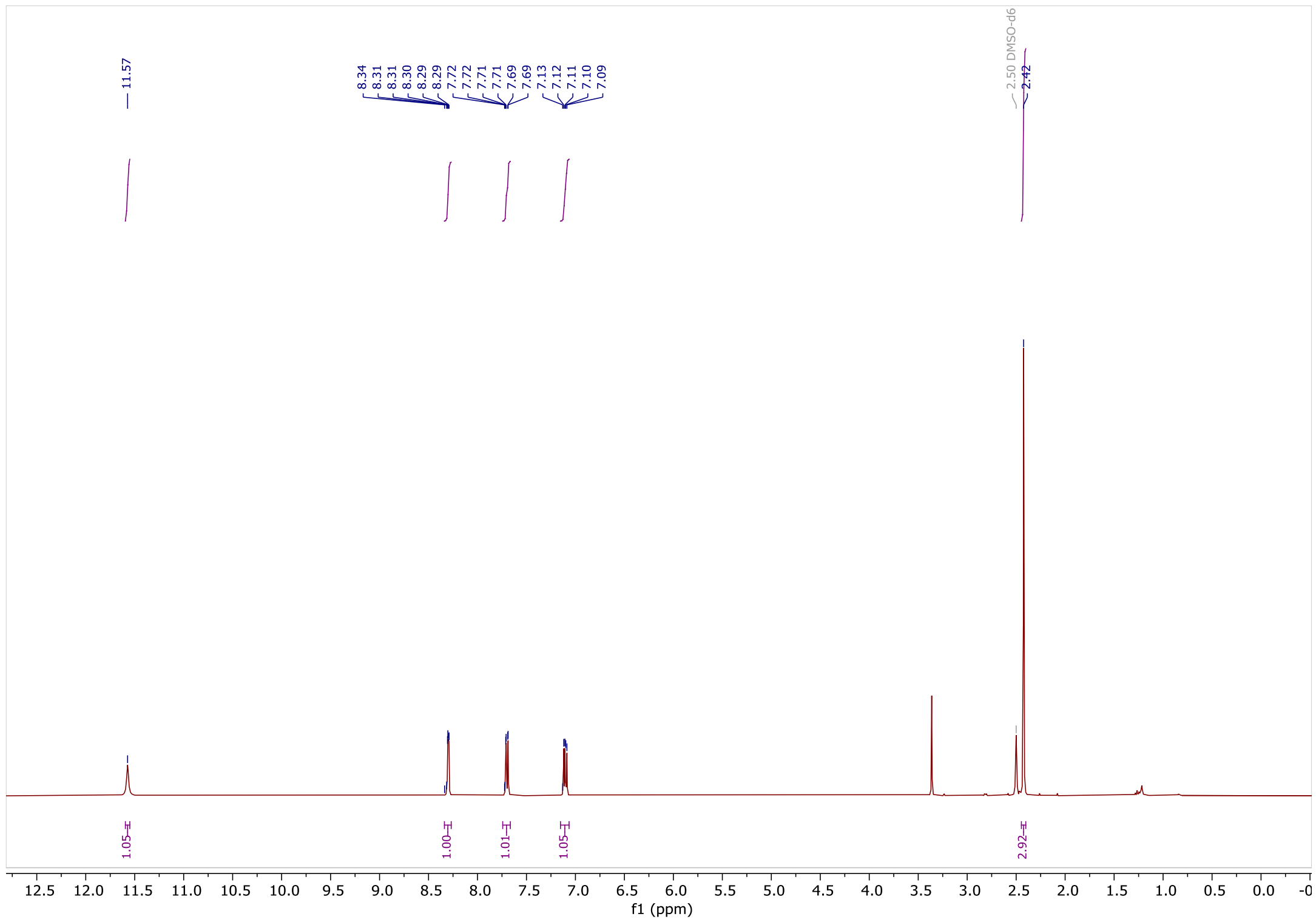

^{13}C-NMR: DMSO-D6

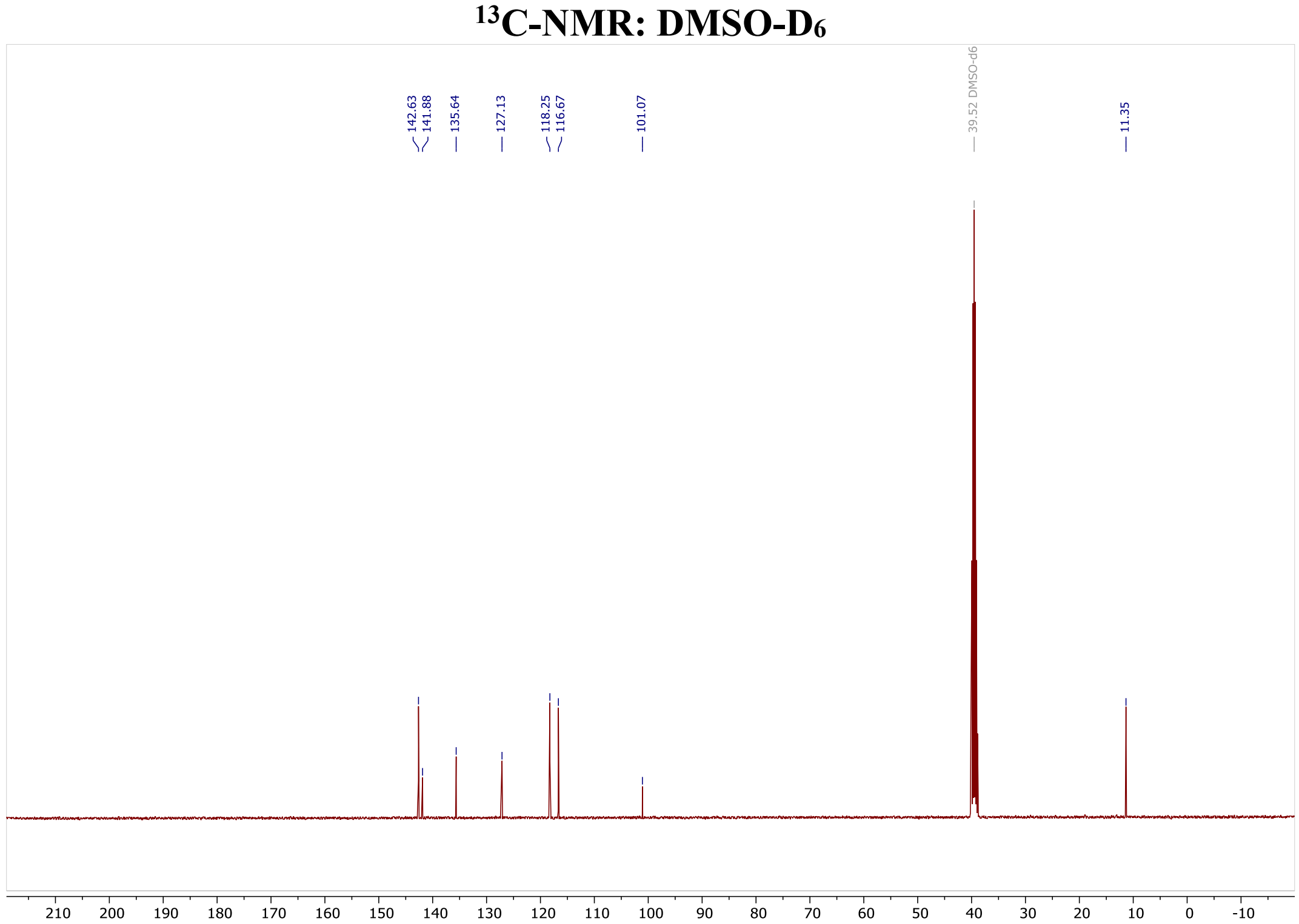

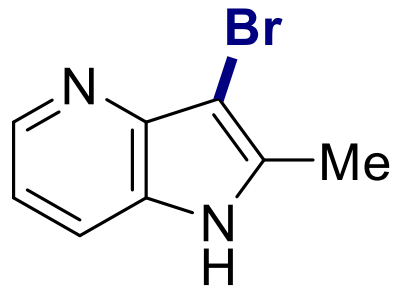

3-bromo-2-methyl-1H-pyrrolo[3,2-b]pyridine (33)

^{1}H-NMR: DMSO-D_6

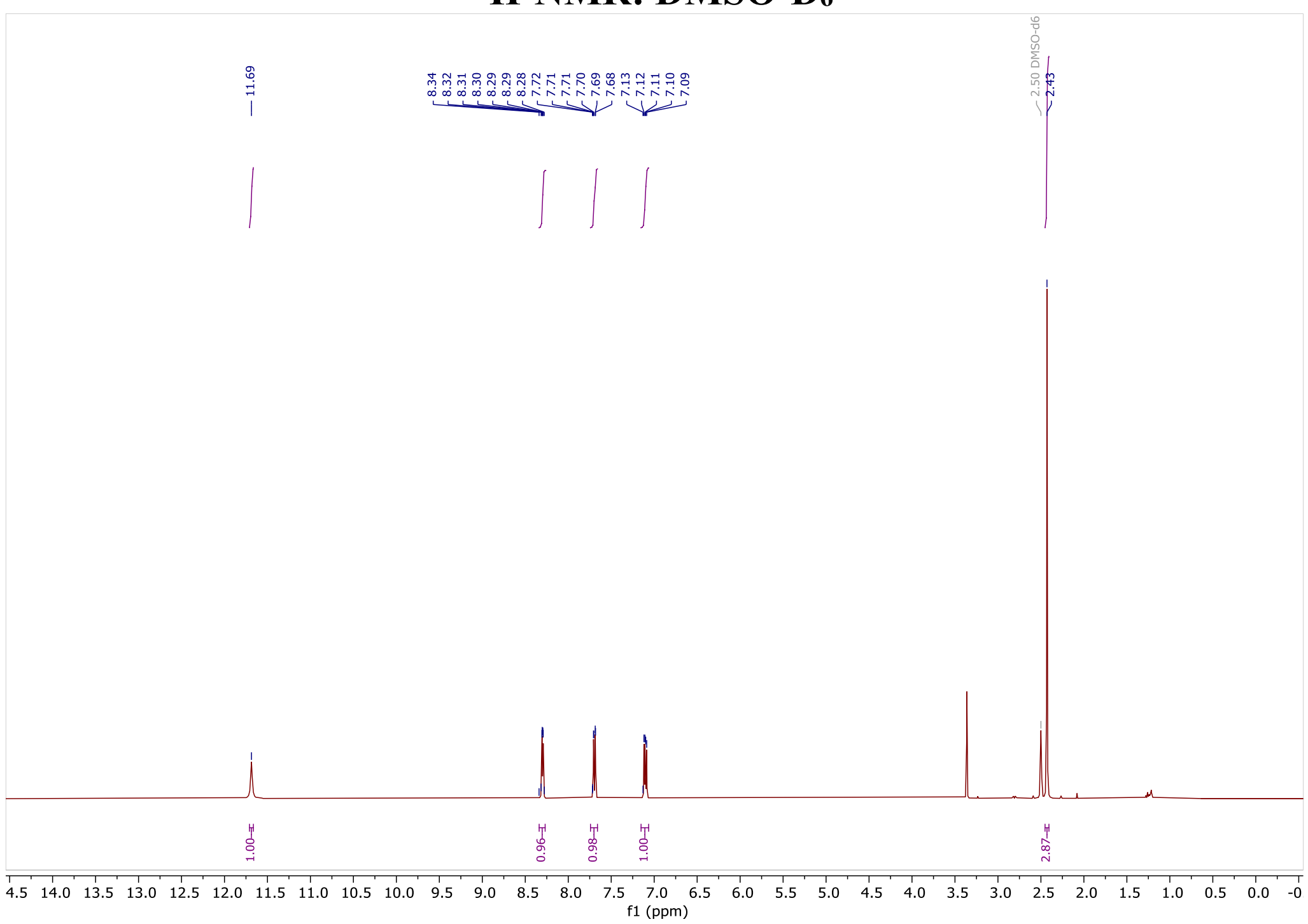

^{13}C-NMR: DMSO-D_6

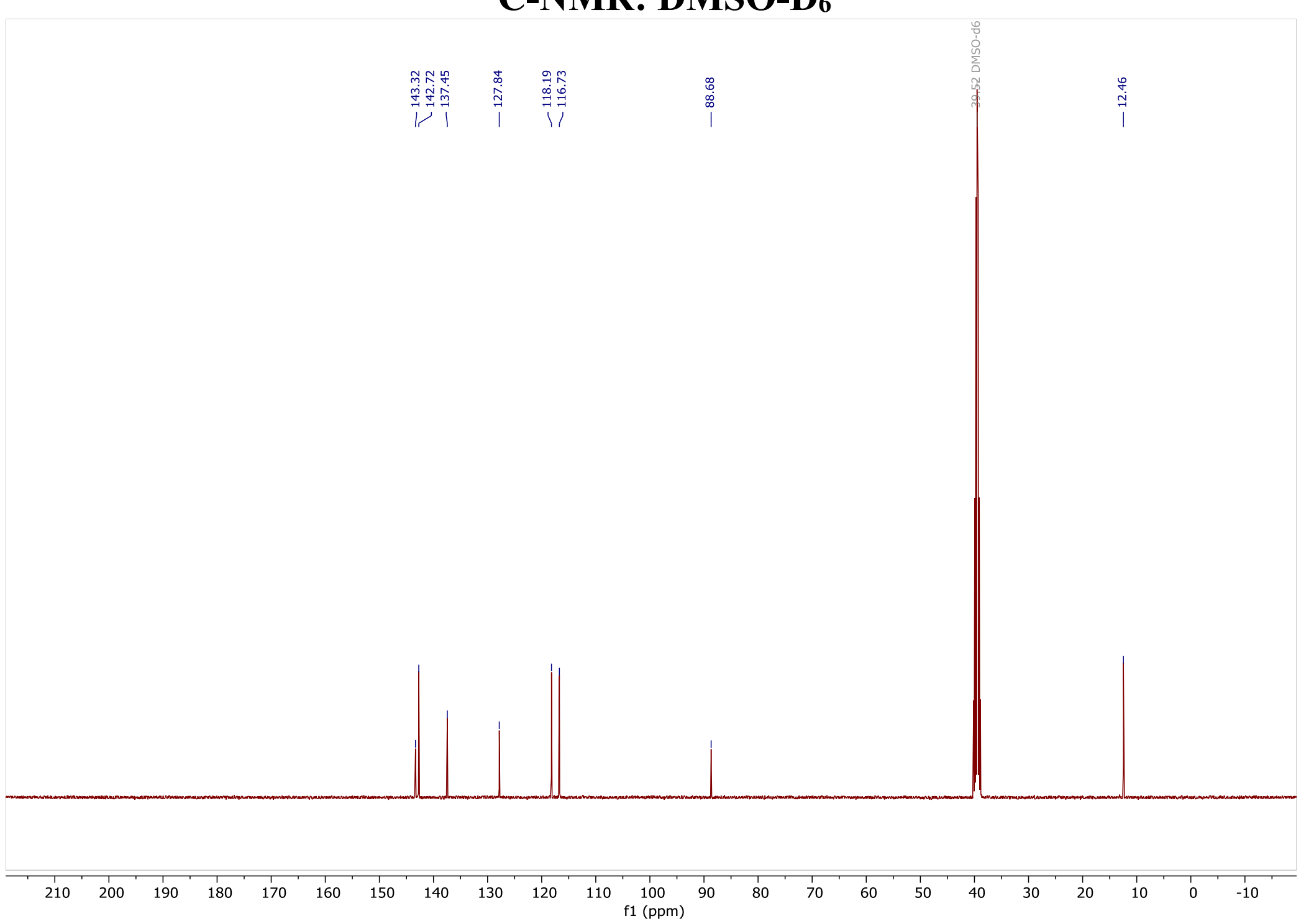

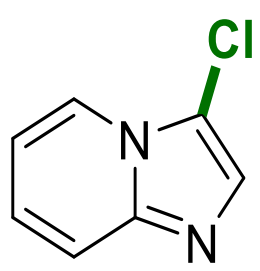

3-chloroimidazo[1,2-a]pyridine (35)

^{1}H-NMR: $CDCl_3$

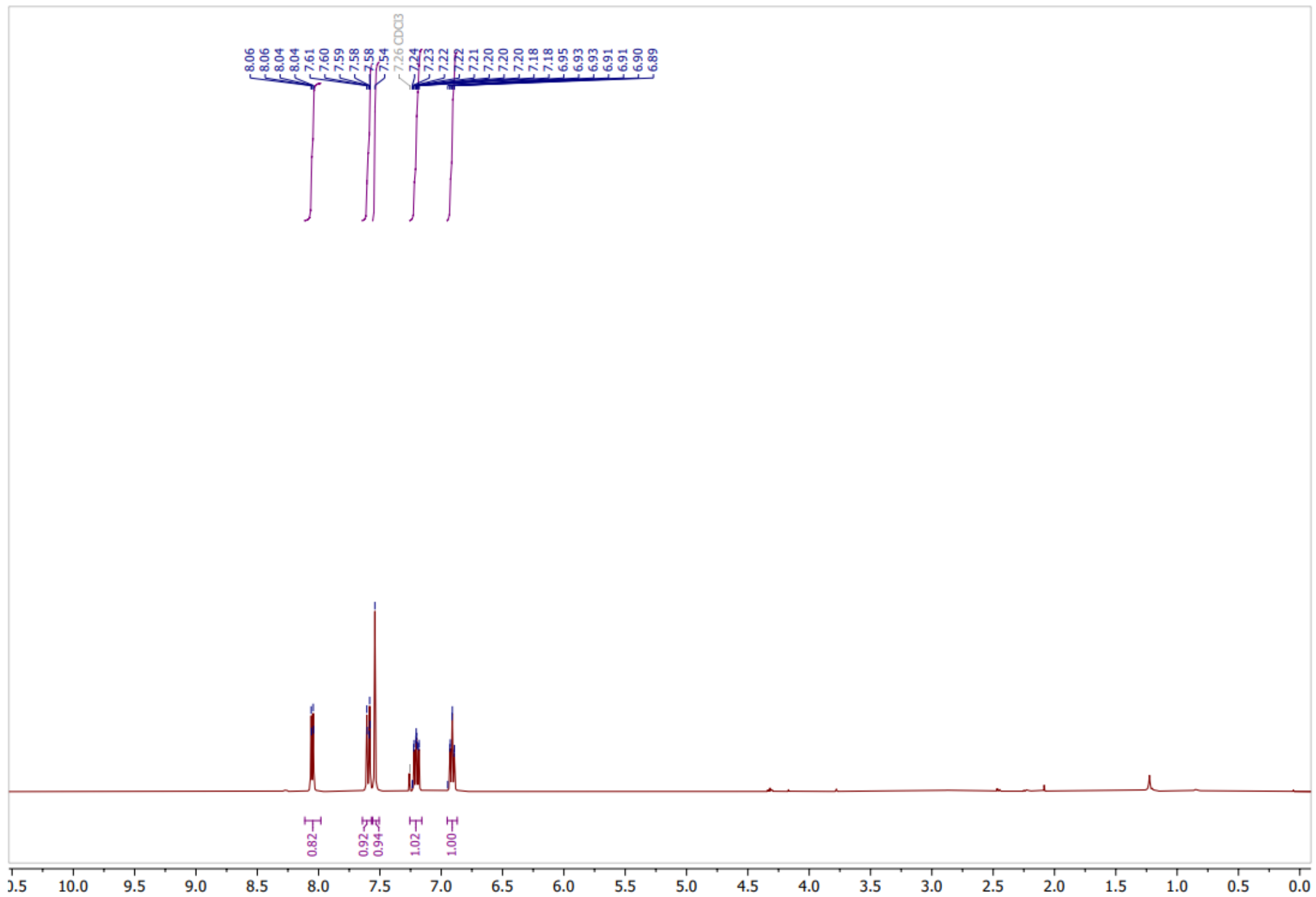

^{13}C-NMR: $CDCl_3$

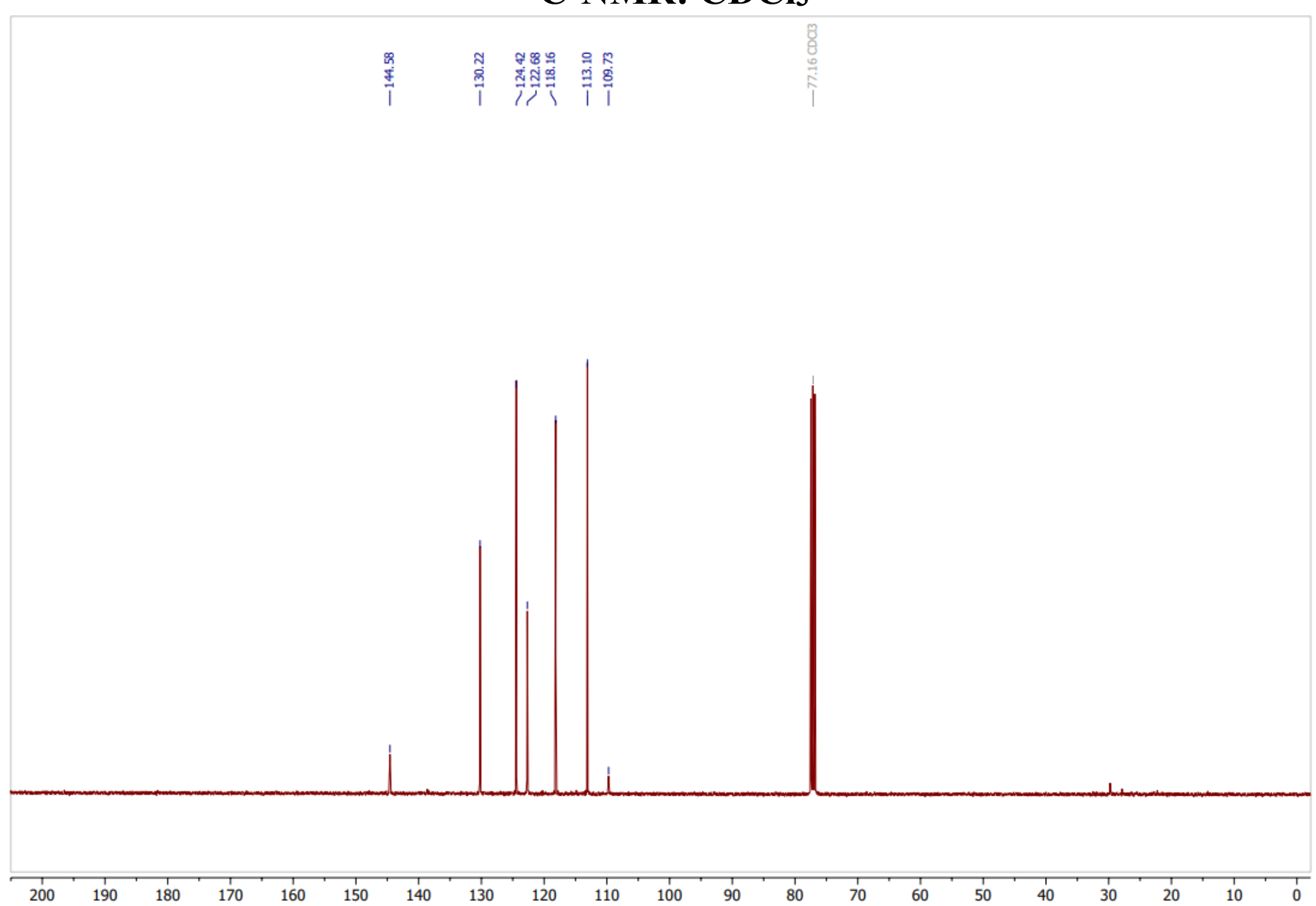

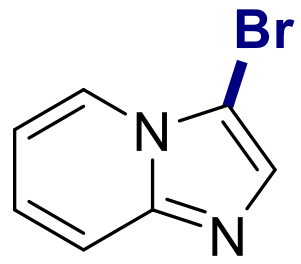

3-bromoimidazo[1,2-a]pyridine (36)

^{1}H-NMR: $CDCl_3$

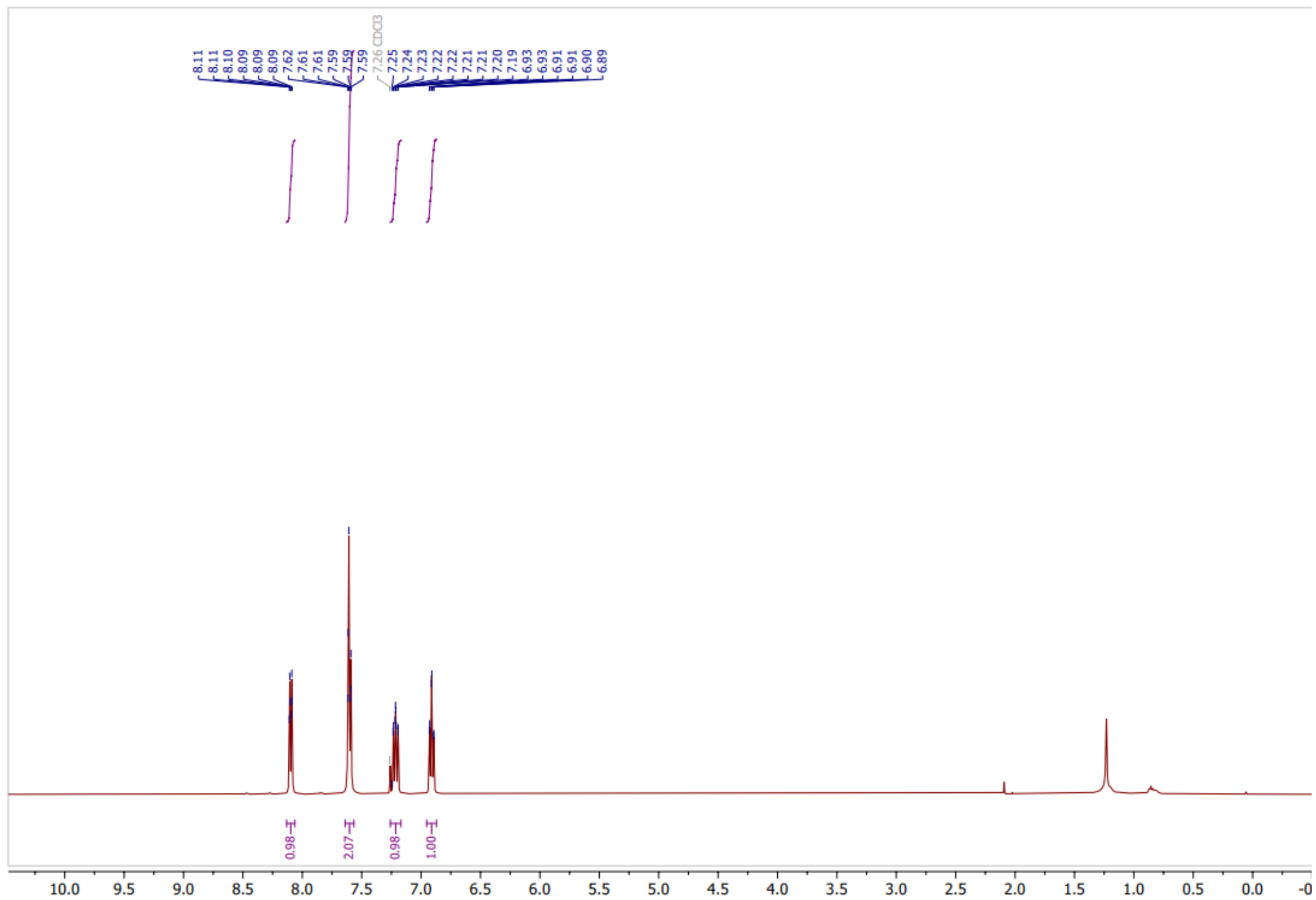

^{13}C-NMR: $CDCl_3$

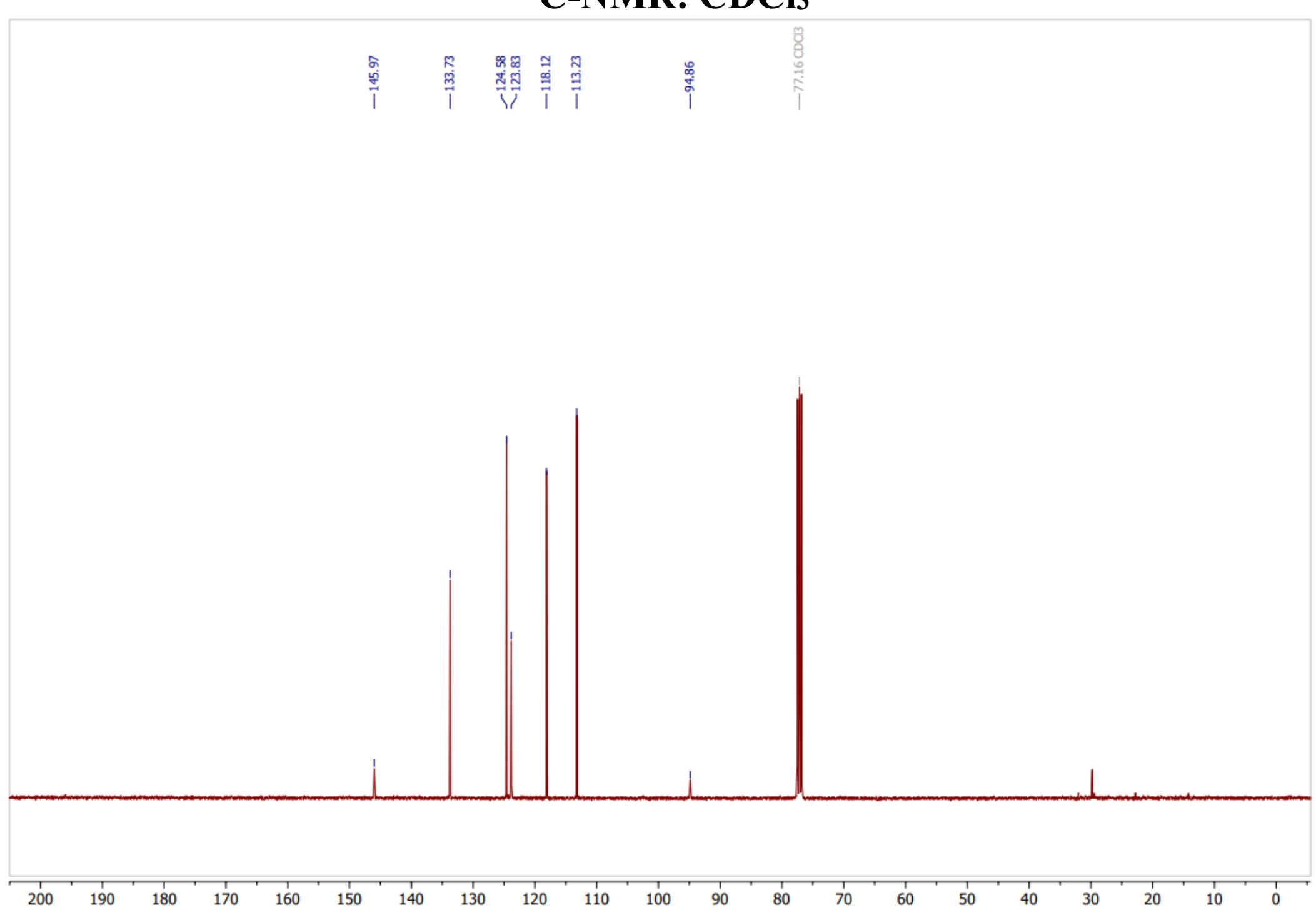

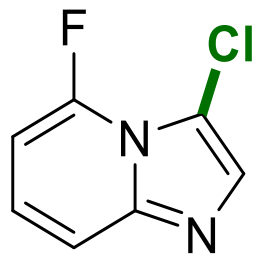

3-chloro-5-fluoroimidazo[1,2-a]pyridine (37)

^{1}H-NMR: $CDCl_3$

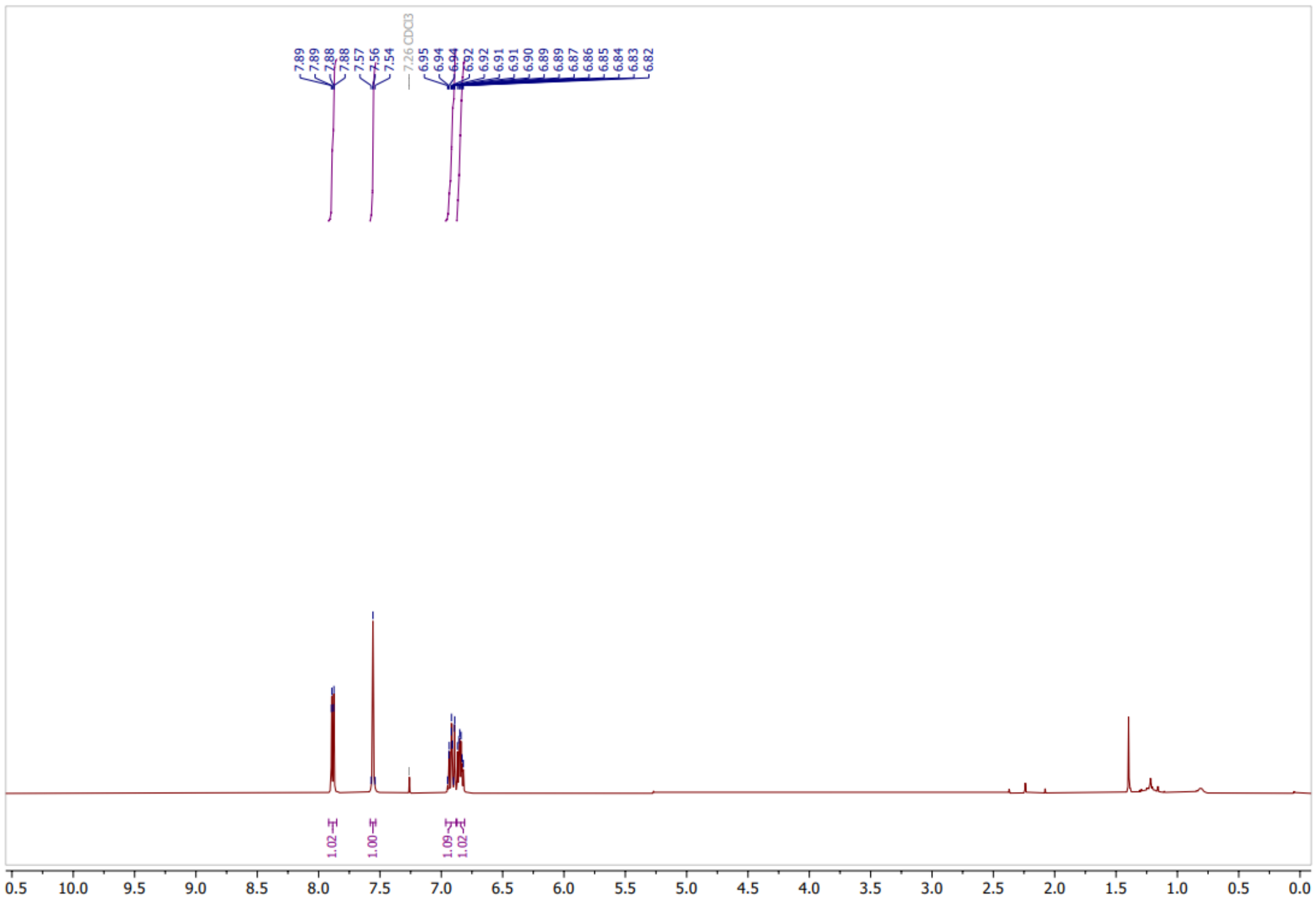

^{13}C-NMR: $CDCl_3$

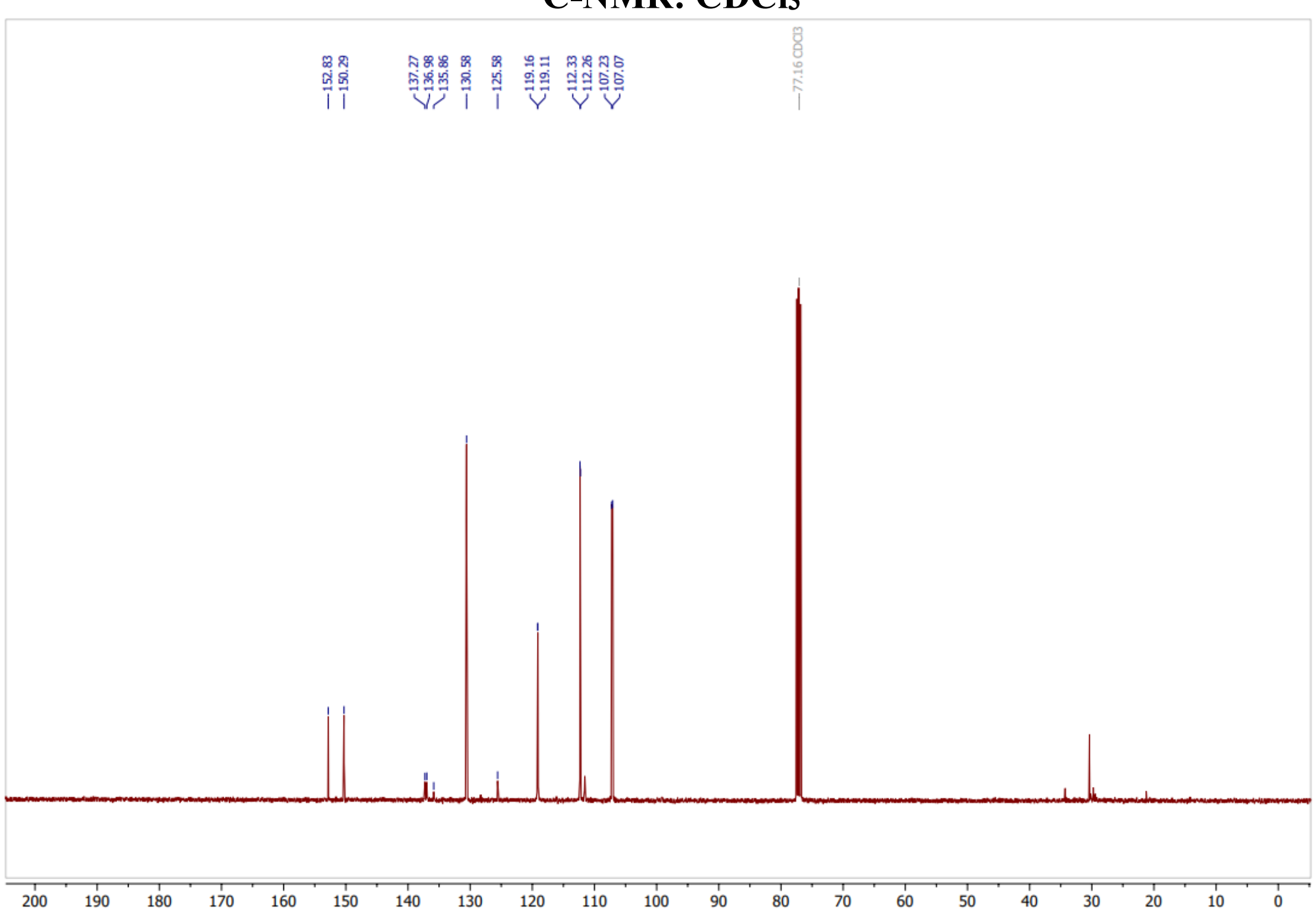

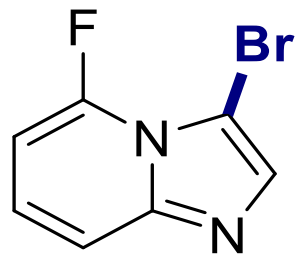

3-bromo-5-fluoroimidazo[1,2-a]pyridine (38)

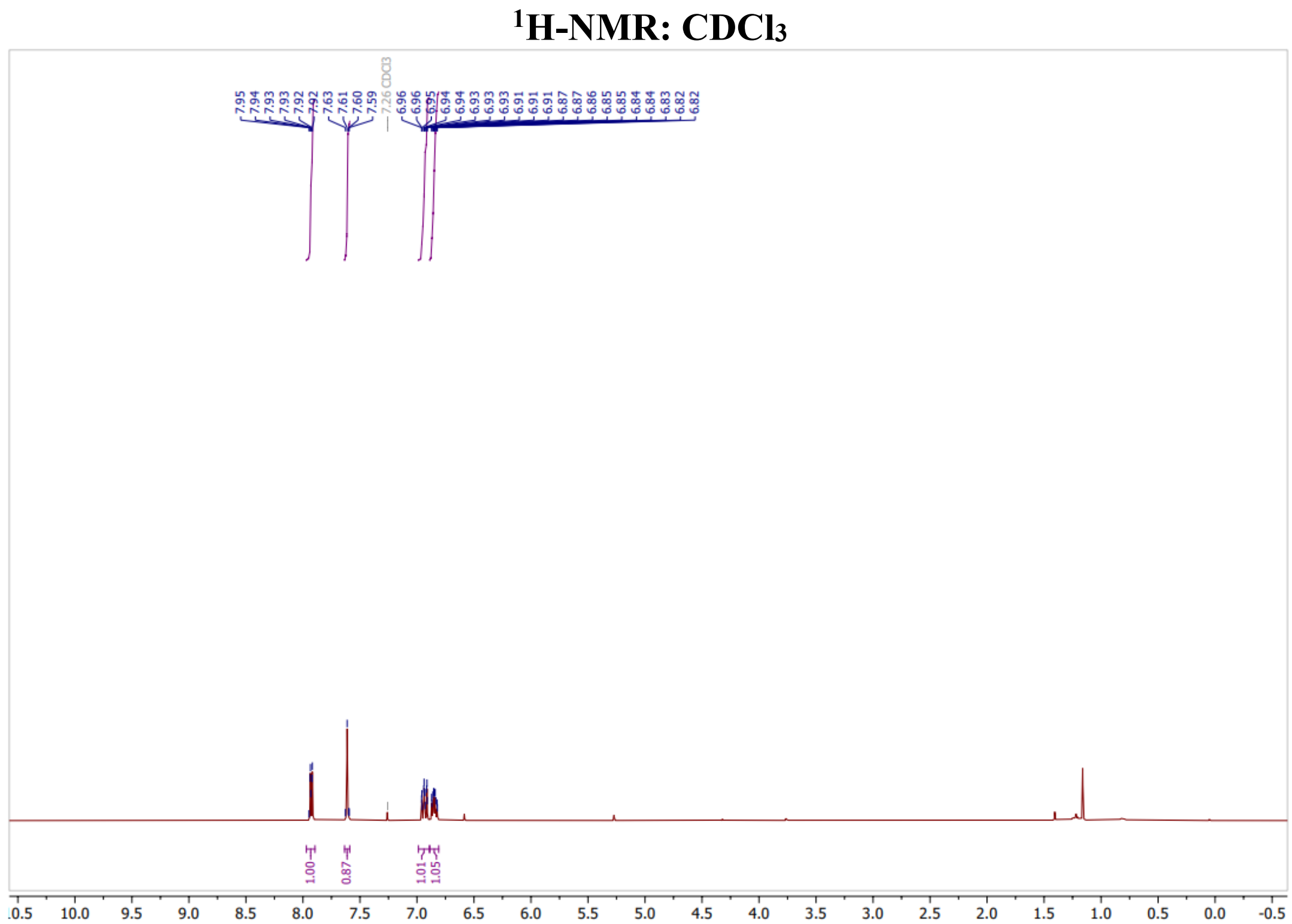

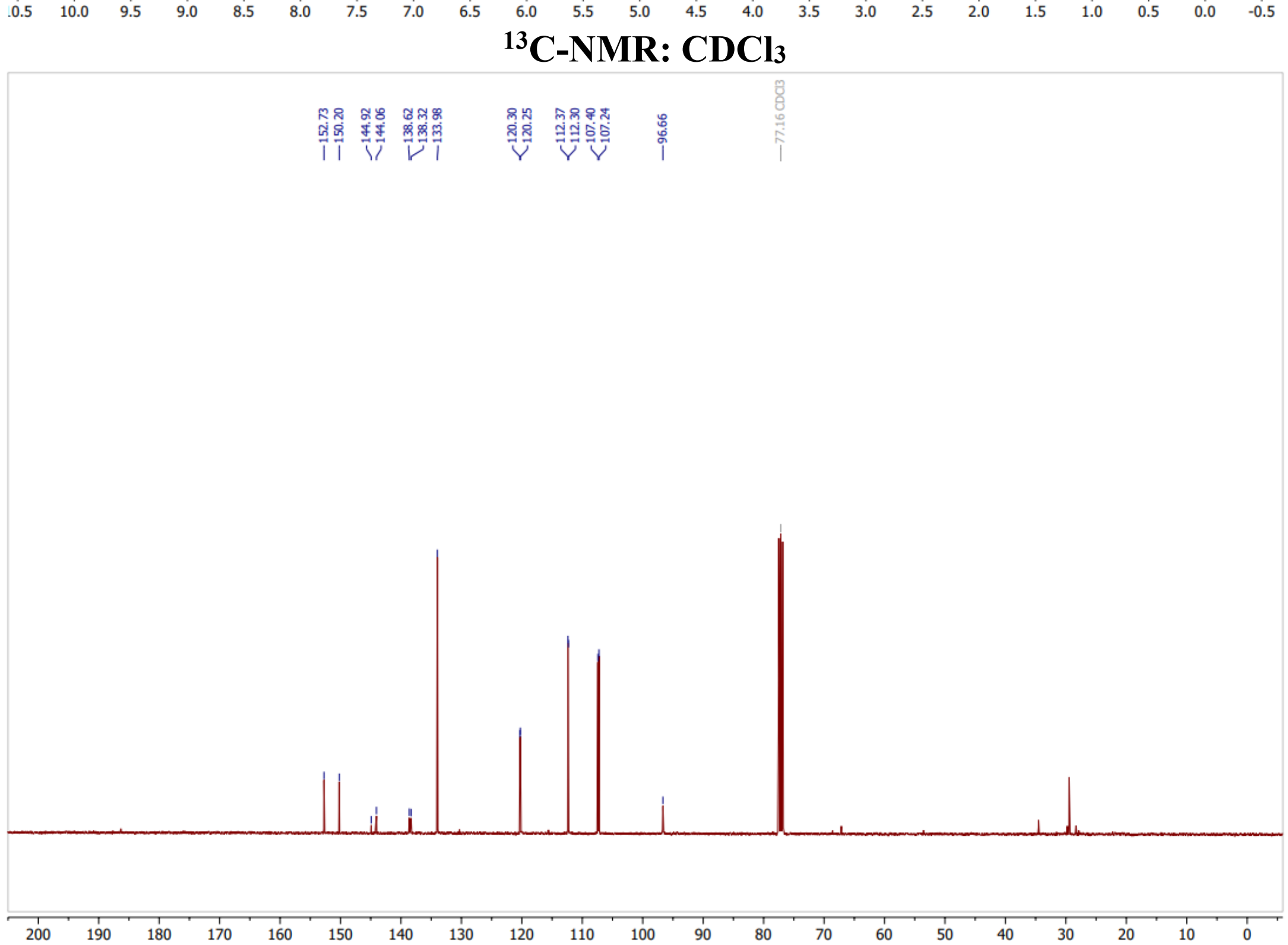

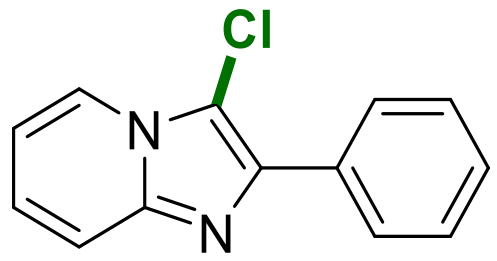

3-chloro-2-phenylimidazo[1,2-a]pyridine (39)

^{1}H-NMR: CDCl$_3$

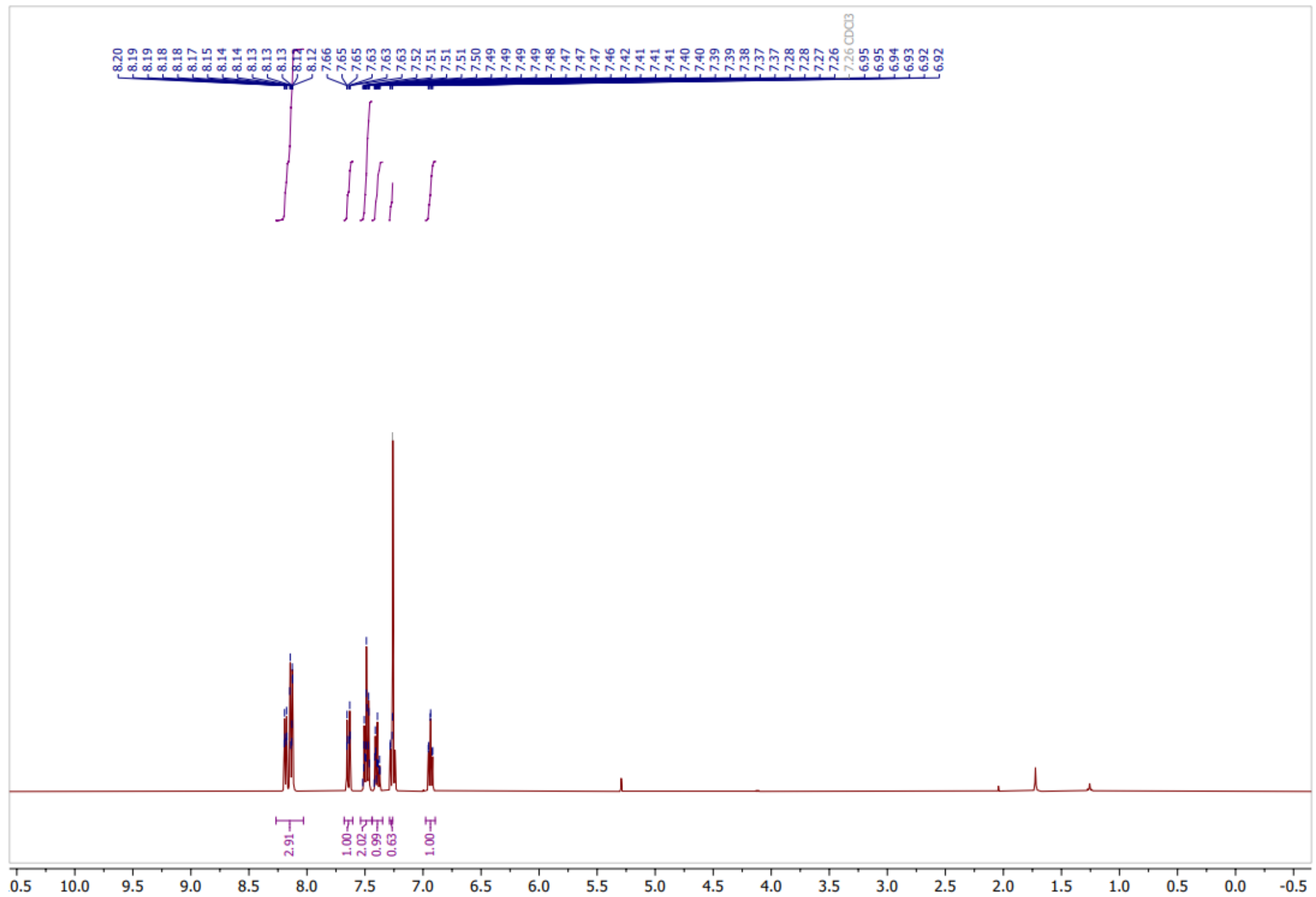

^{13}C-NMR: CDCl$_3$

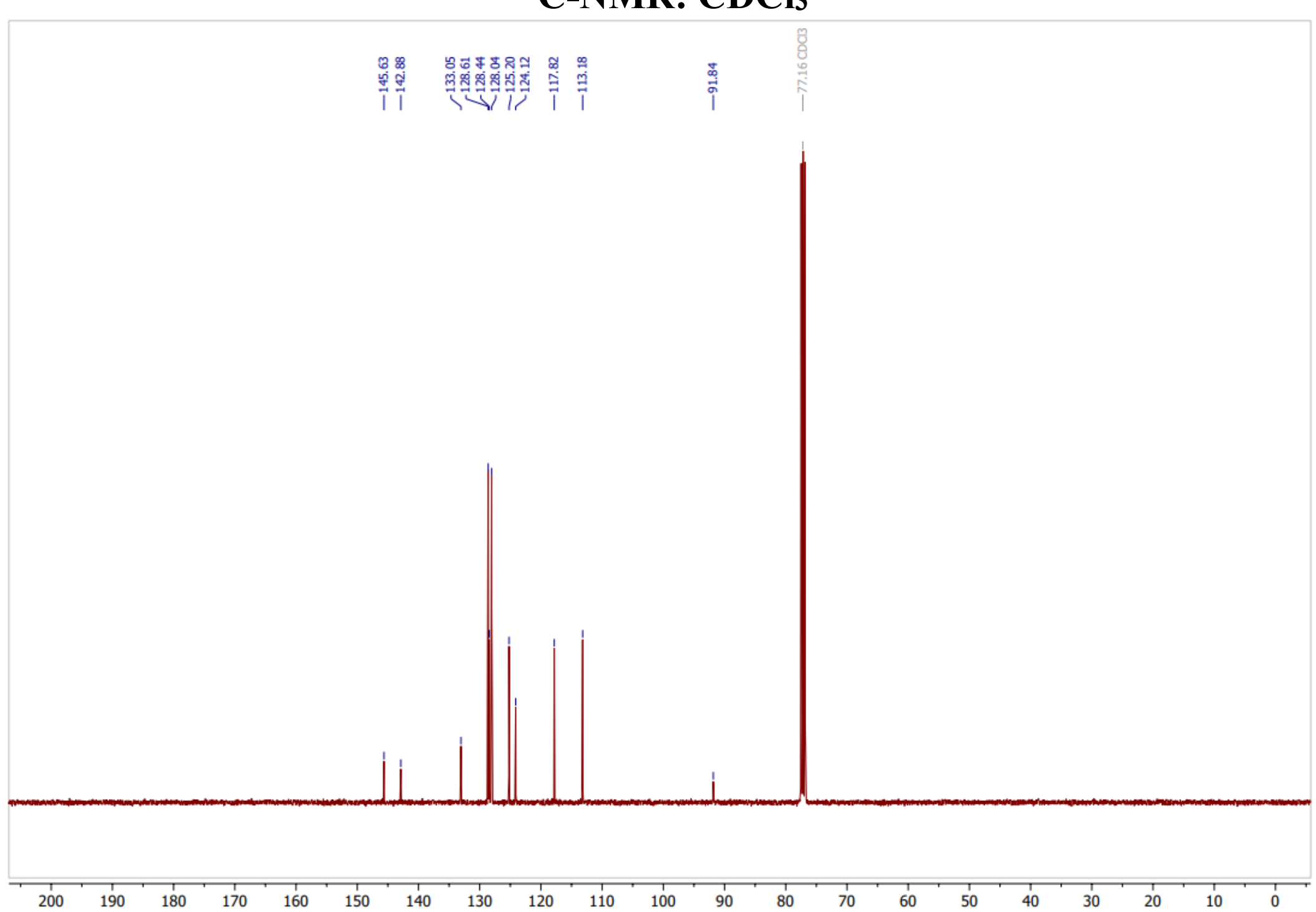

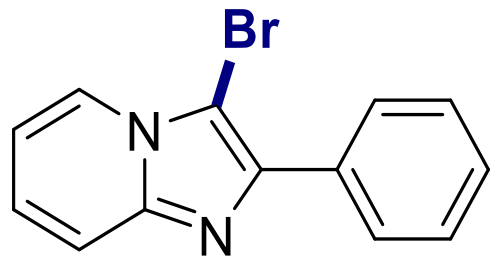

3-bromo-2-phenylimidazo[1,2-a]pyridine (40)

^{1}H-NMR: $CDCl_3$

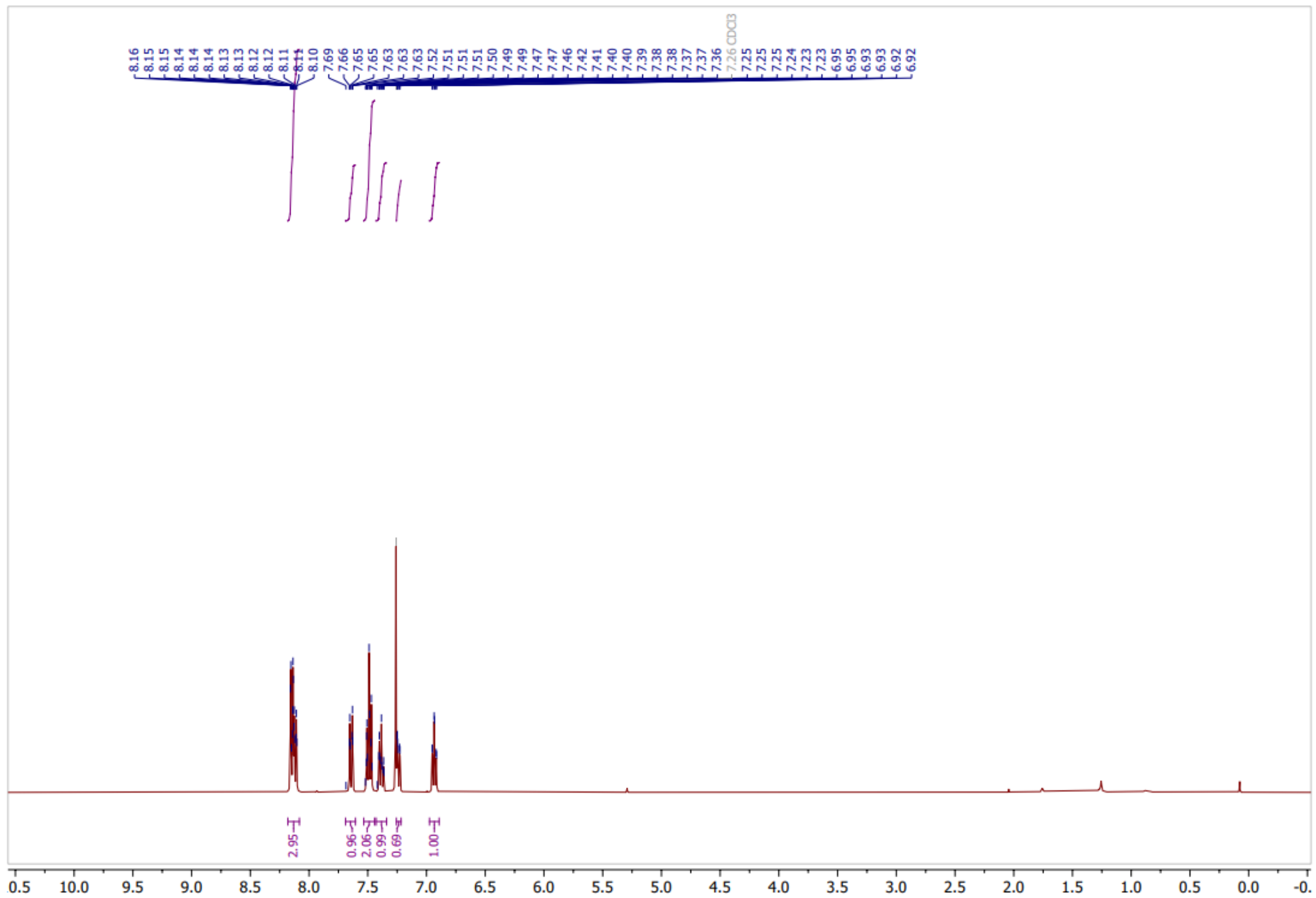

^{13}C-NMR: $CDCl_3$

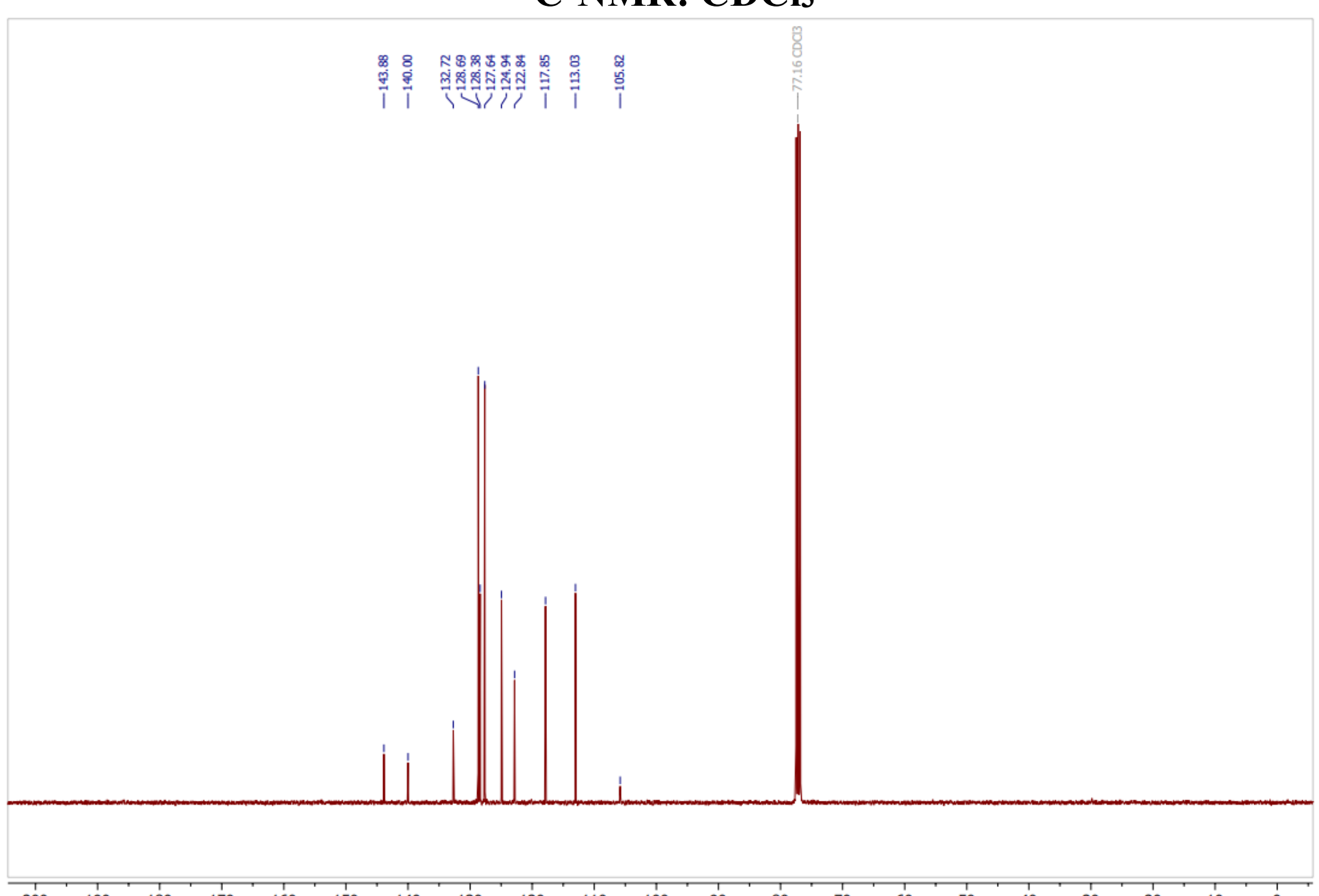

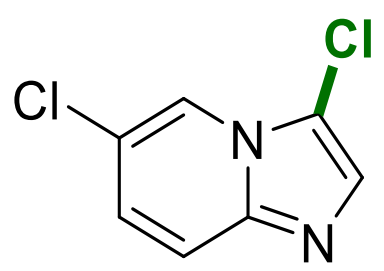

3,6-dichloroimidazo[1,2-a]pyridine (42)

^{1}H-NMR: $CDCl_3$

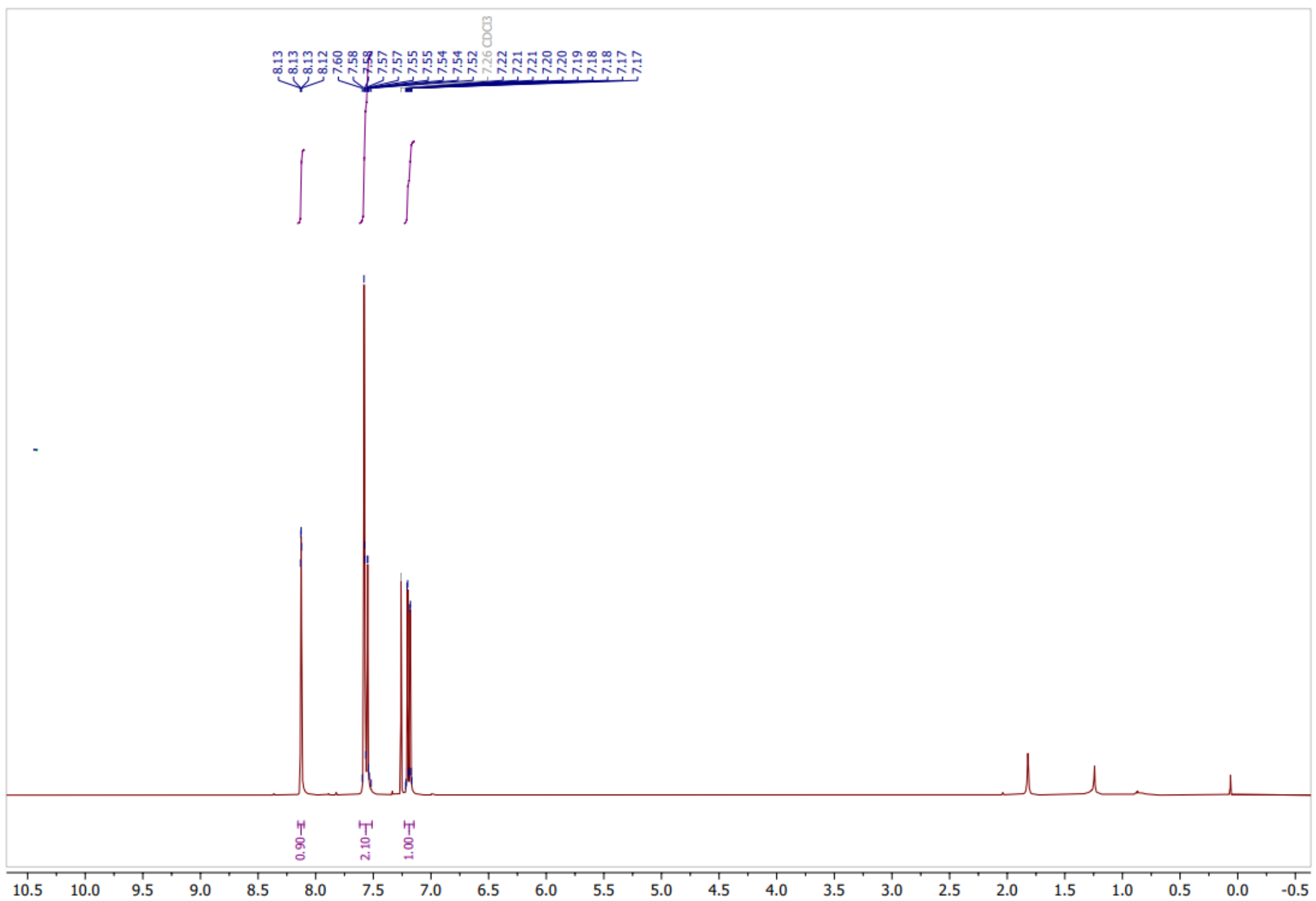

^{13}C-NMR: $CDCl_3$

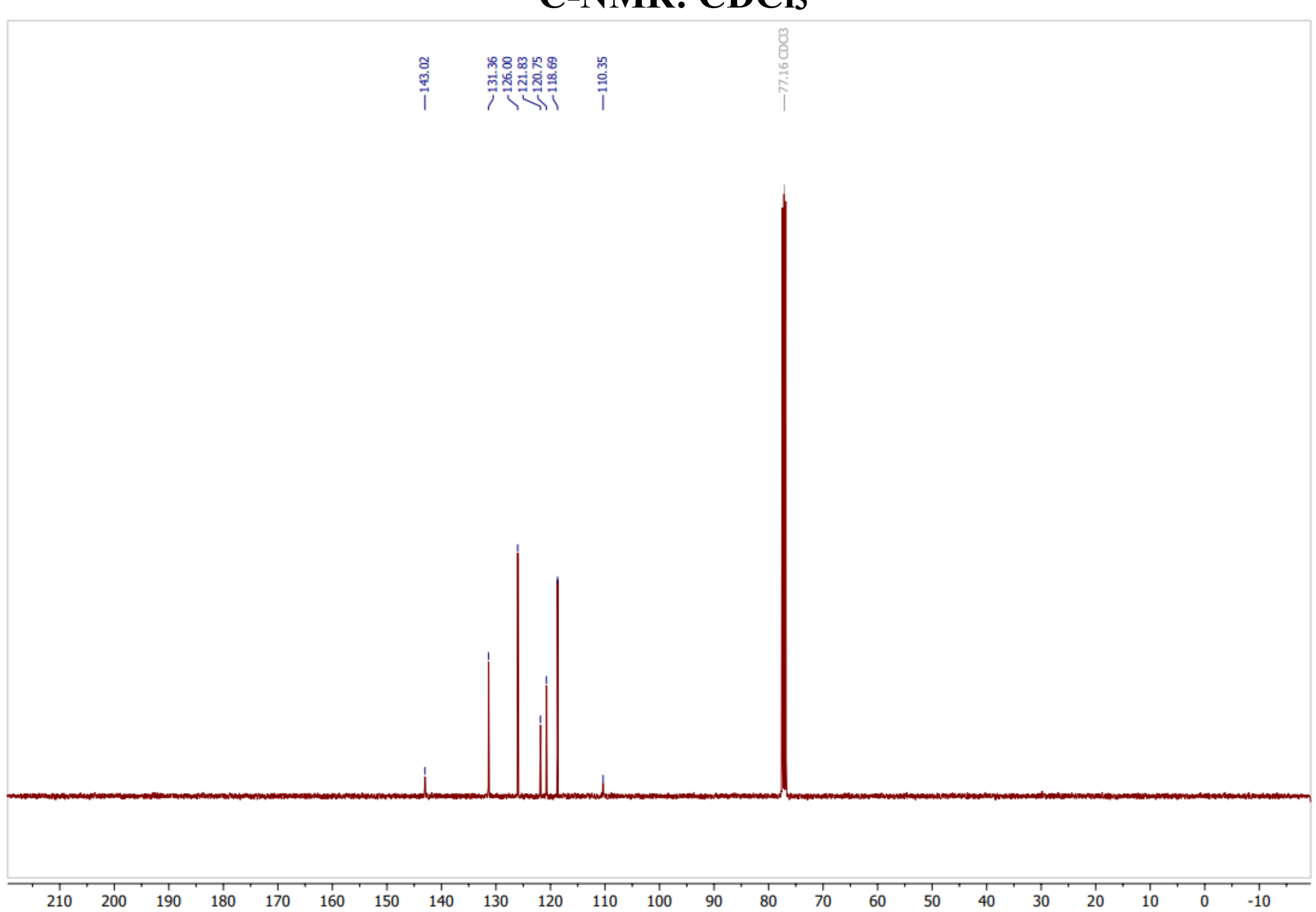

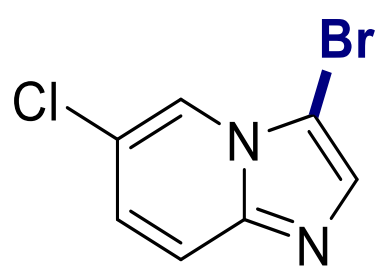

3-bromo-6-chloroimidazo[1,2-a]pyridine (43)

^{1}H-NMR: $CDCl_3$

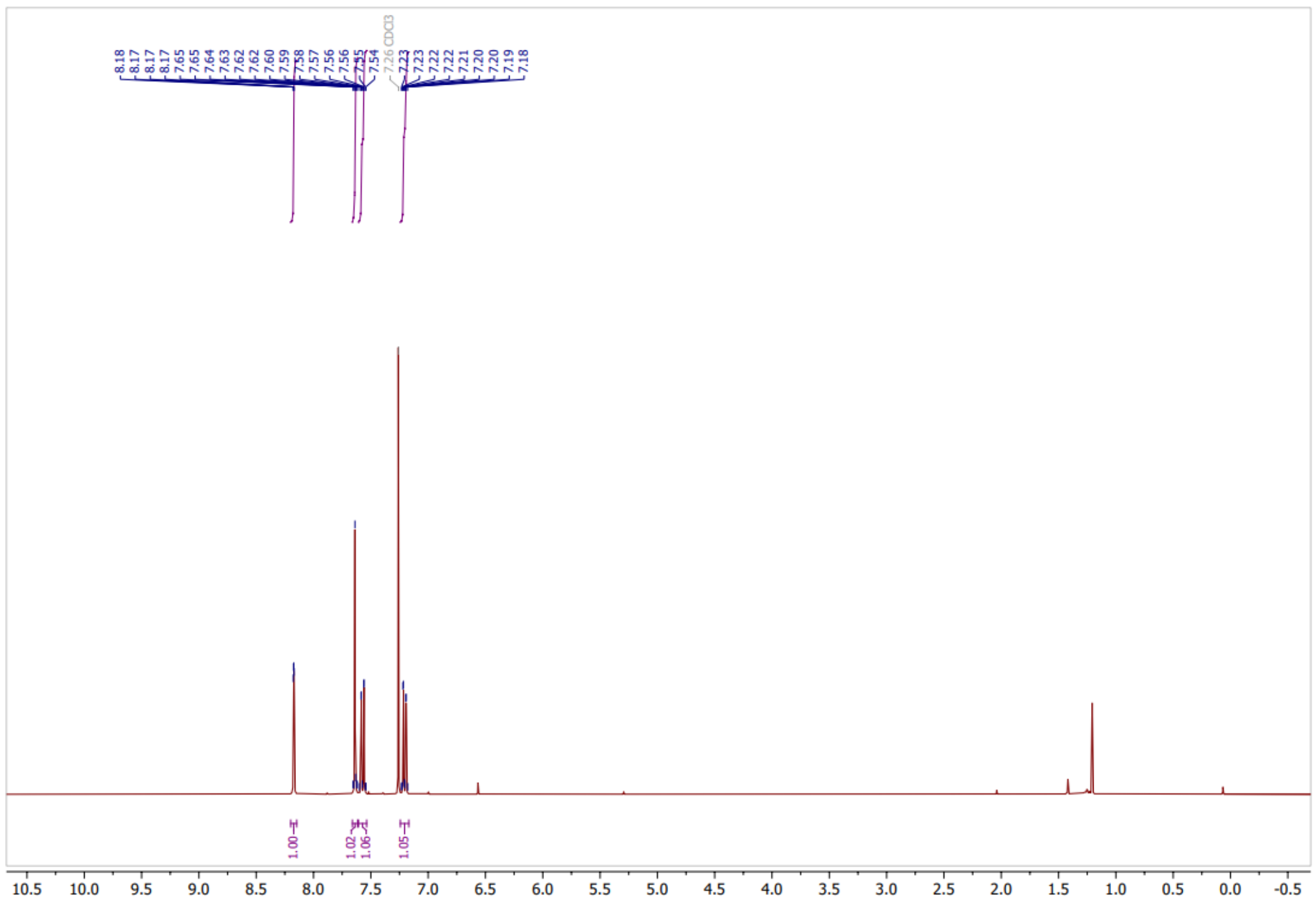

^{13}C-NMR: $CDCl_3$

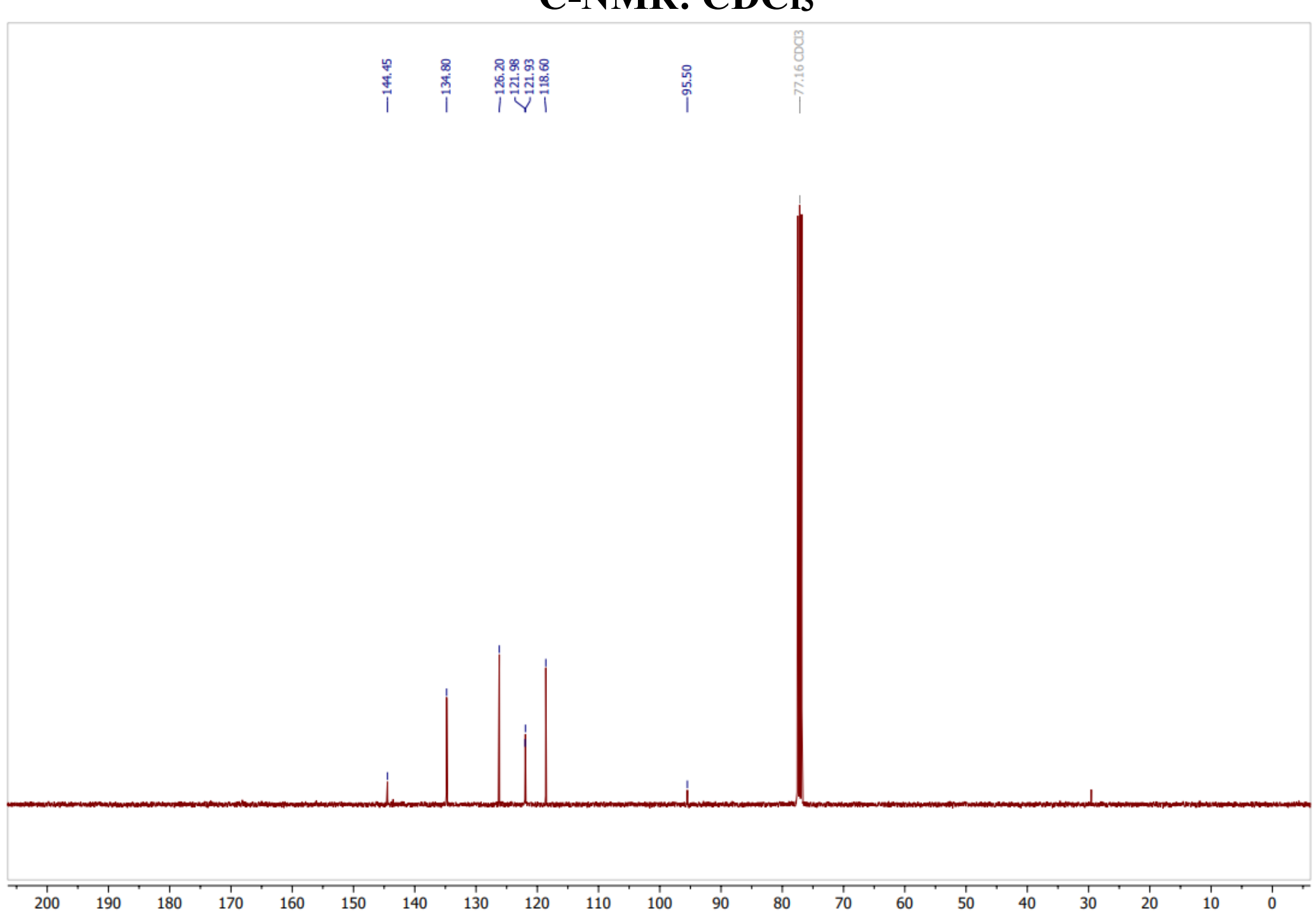

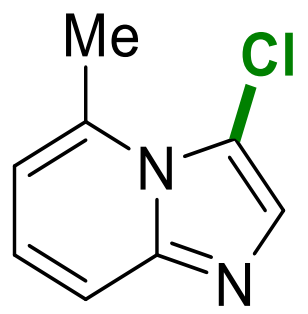

3-chloro-5-methylimidazo[1,2-a]pyridine (44)

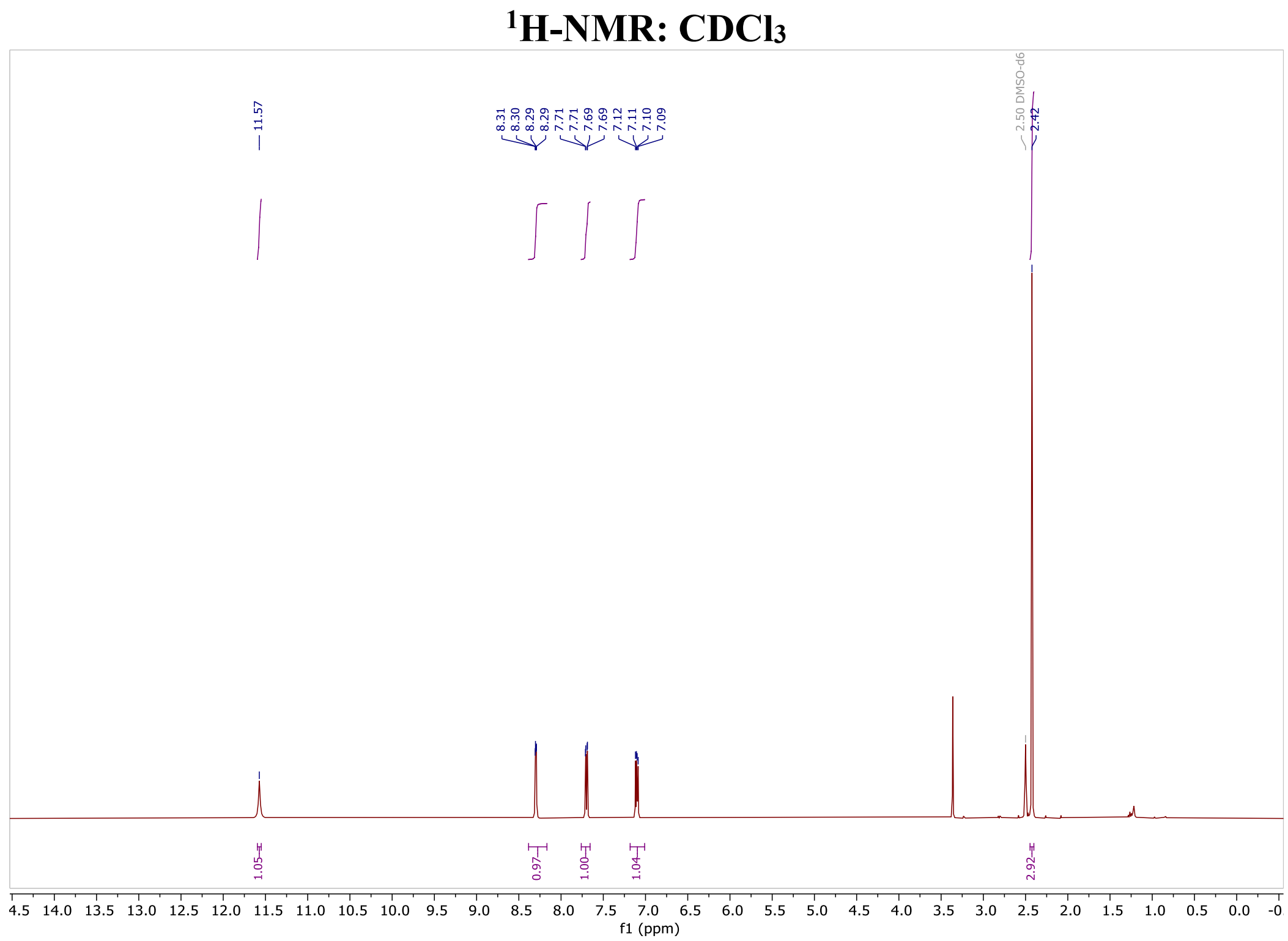

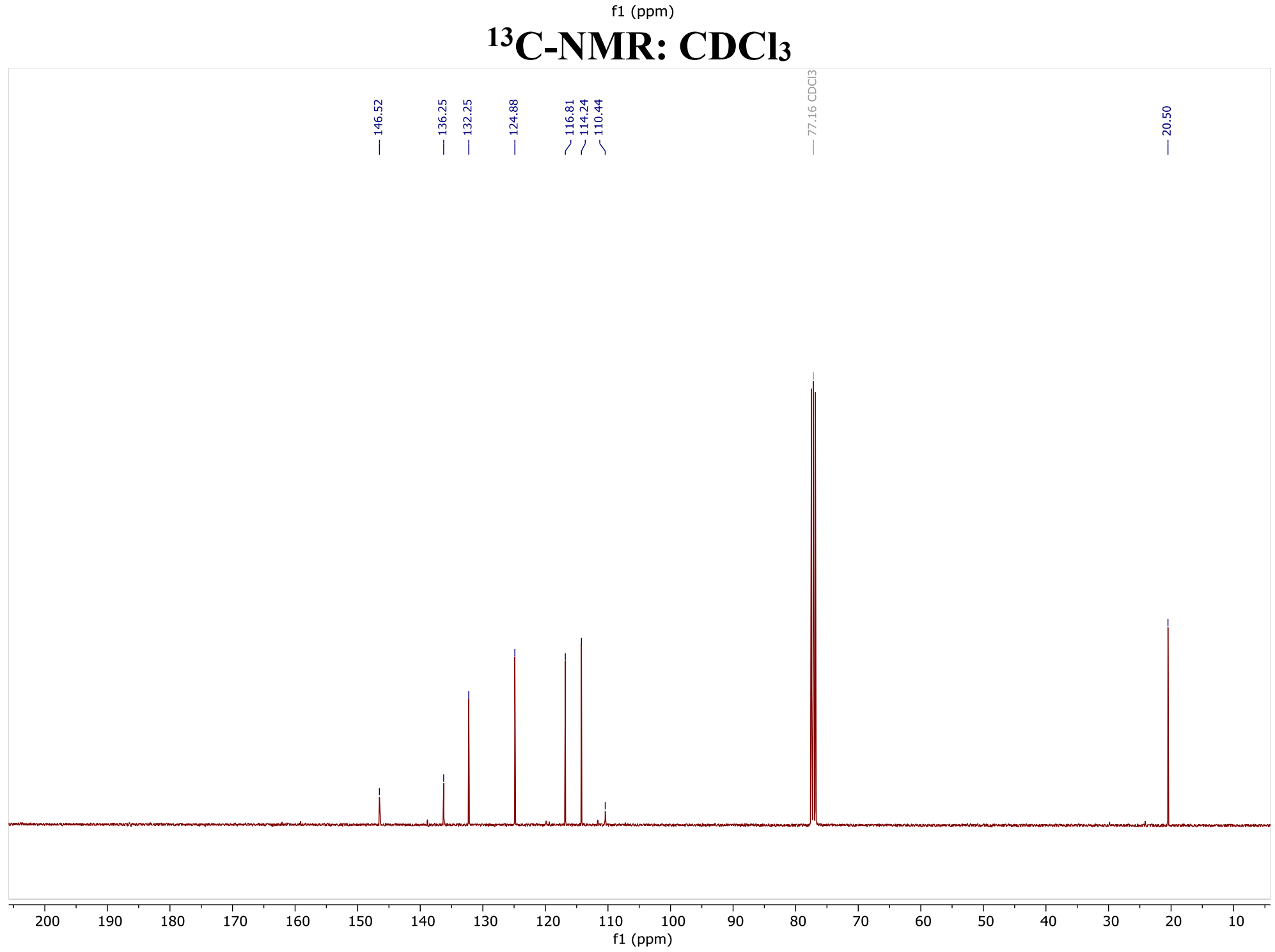

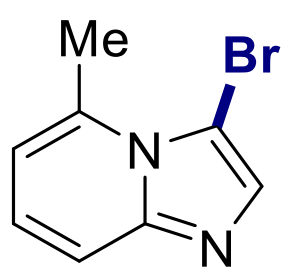

3-bromo-5-methylimidazo[1,2-a]pyridine (45)

^{1}H-NMR: CDCl$_3$

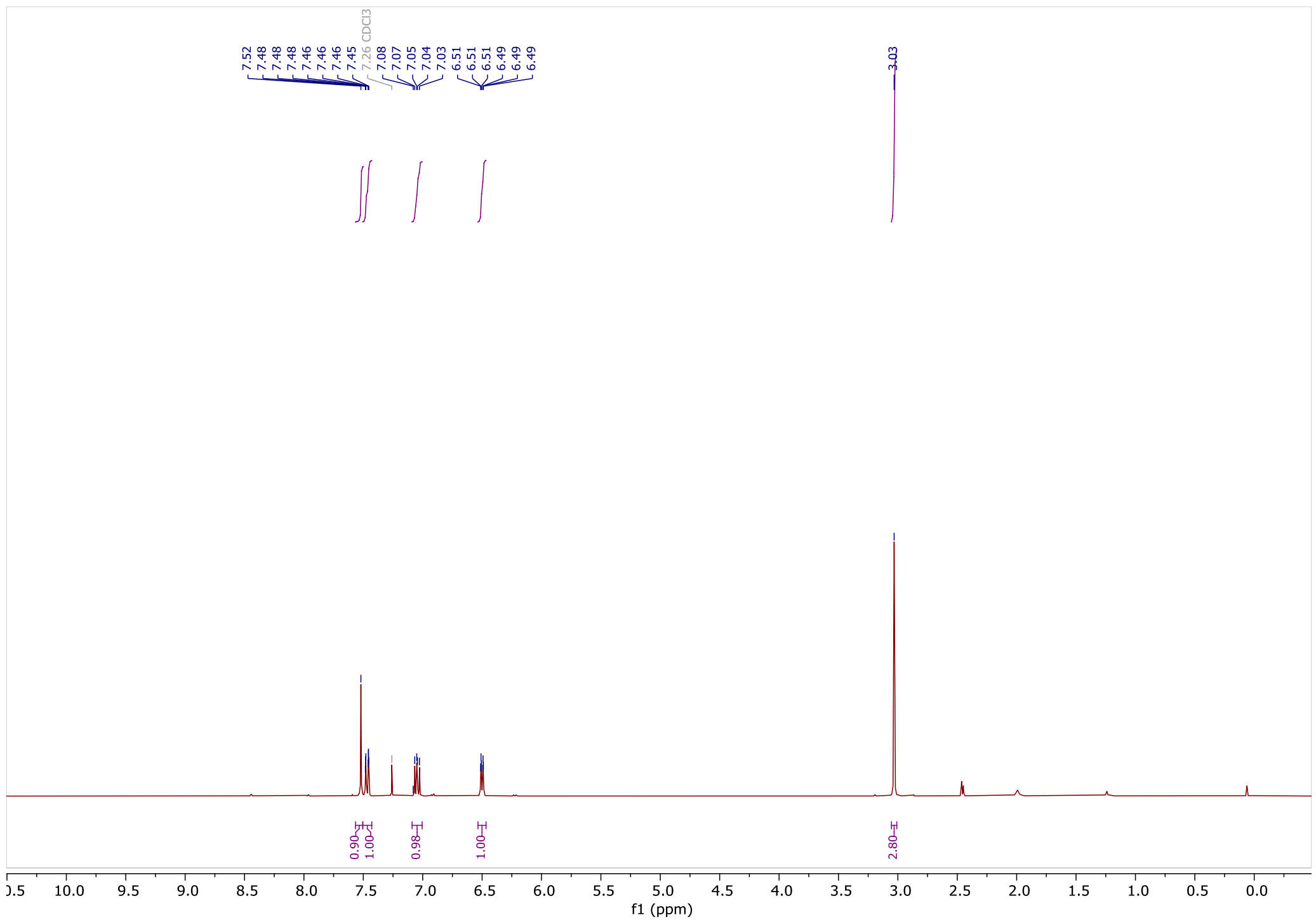

^{13}C-NMR: CDCl$_3$

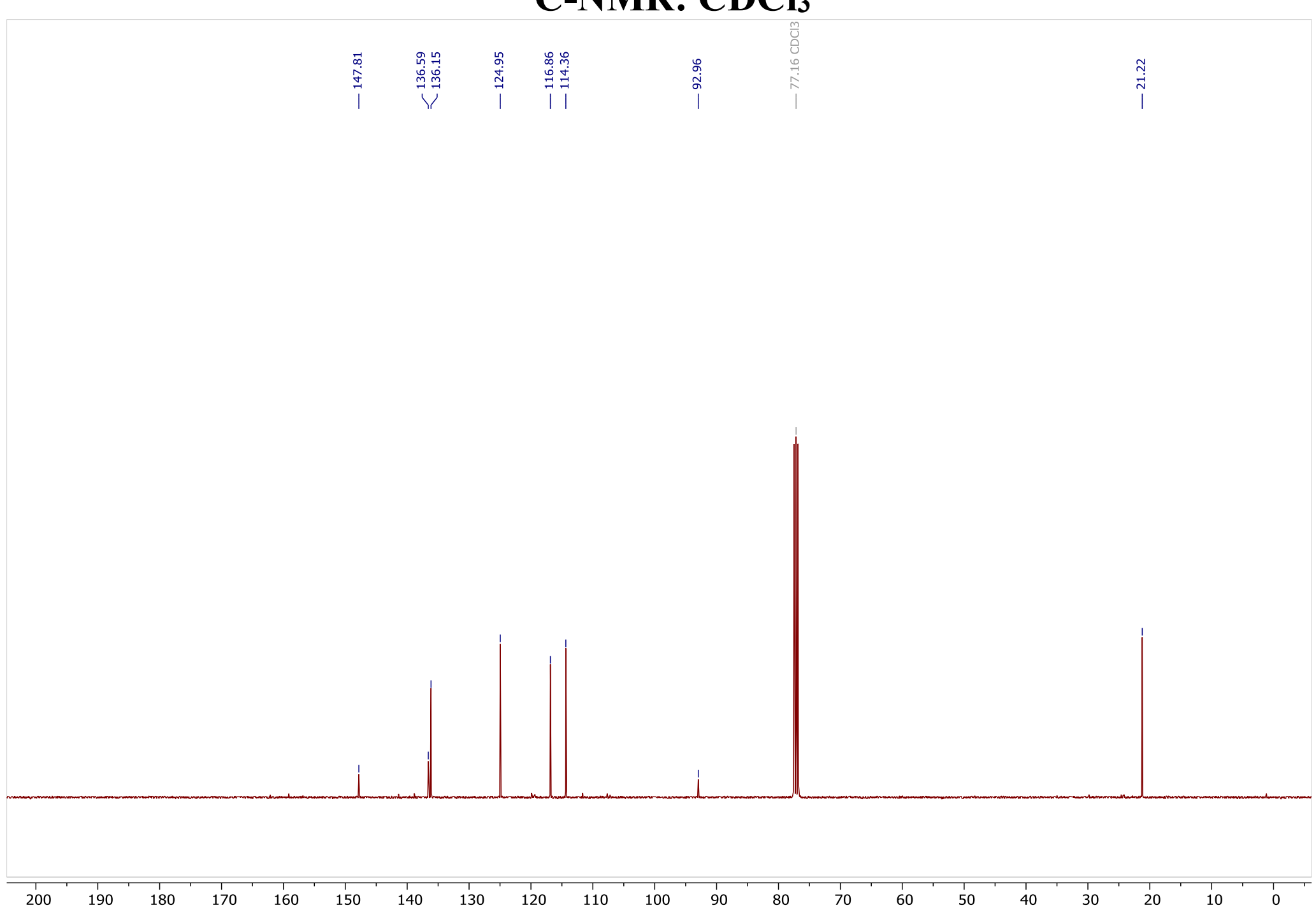

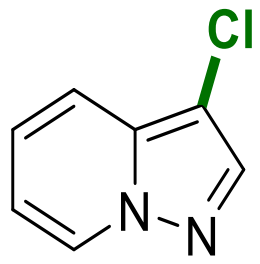

3-chloropyrazolo[1,5-a]pyridine (46)

^{1}H-NMR: $CDCl_3$

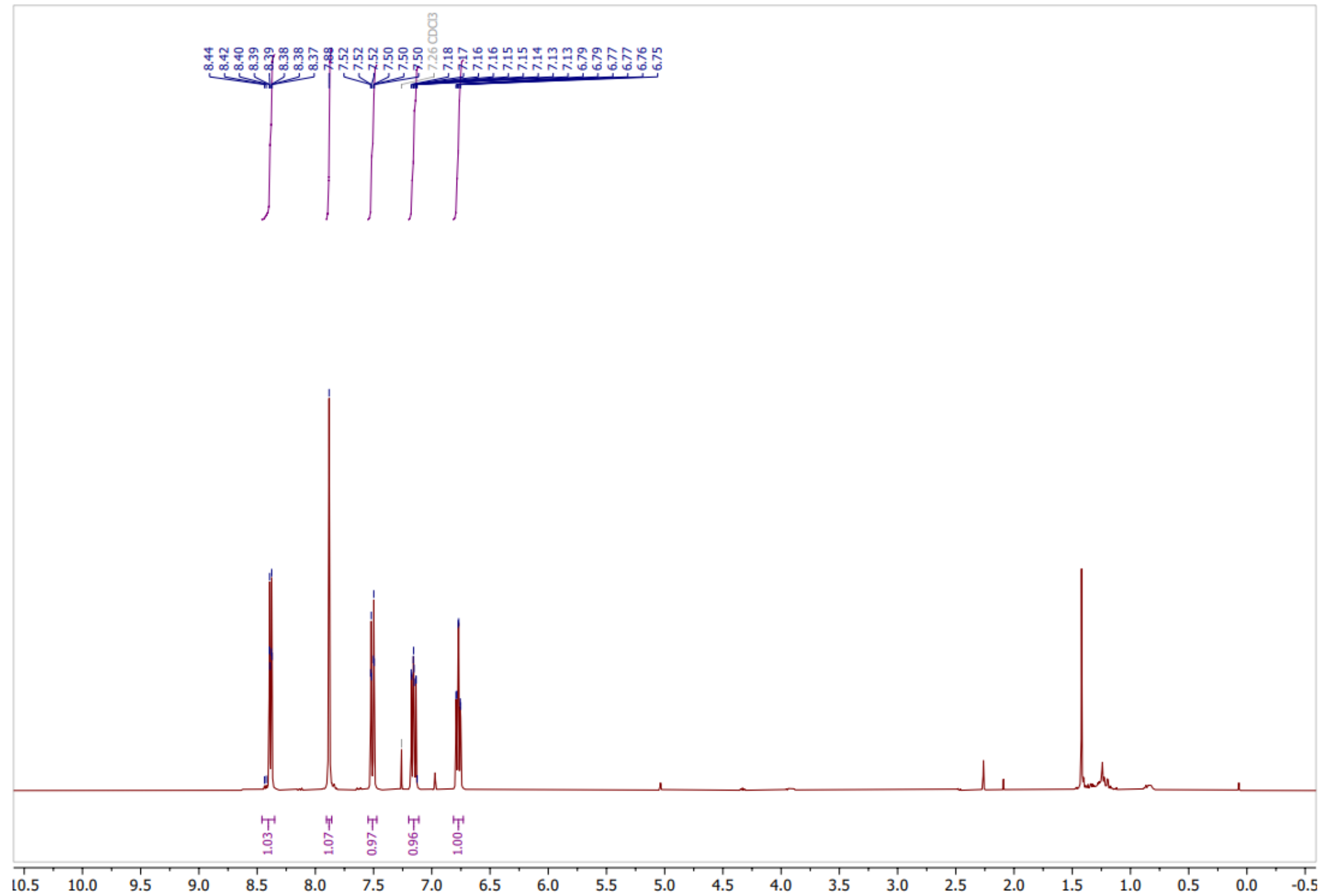

^{13}C-NMR: $CDCl_3$

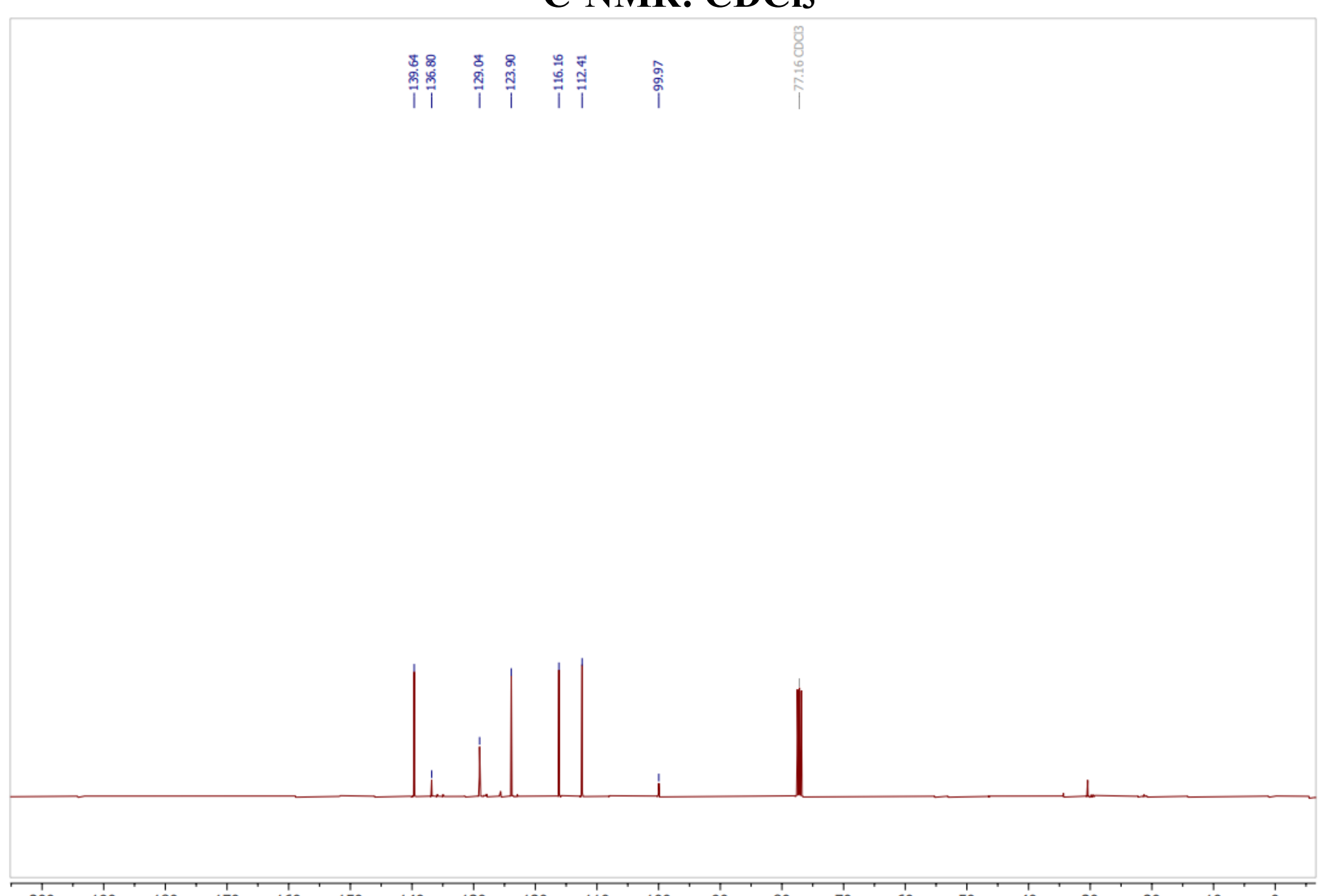

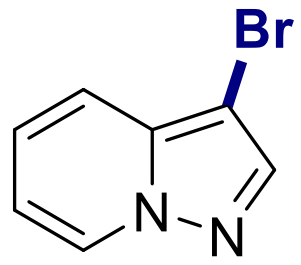

3-bromopyrazolo[1,5-a]pyridine (47)

^{1}H-NMR: $CDCl_3$

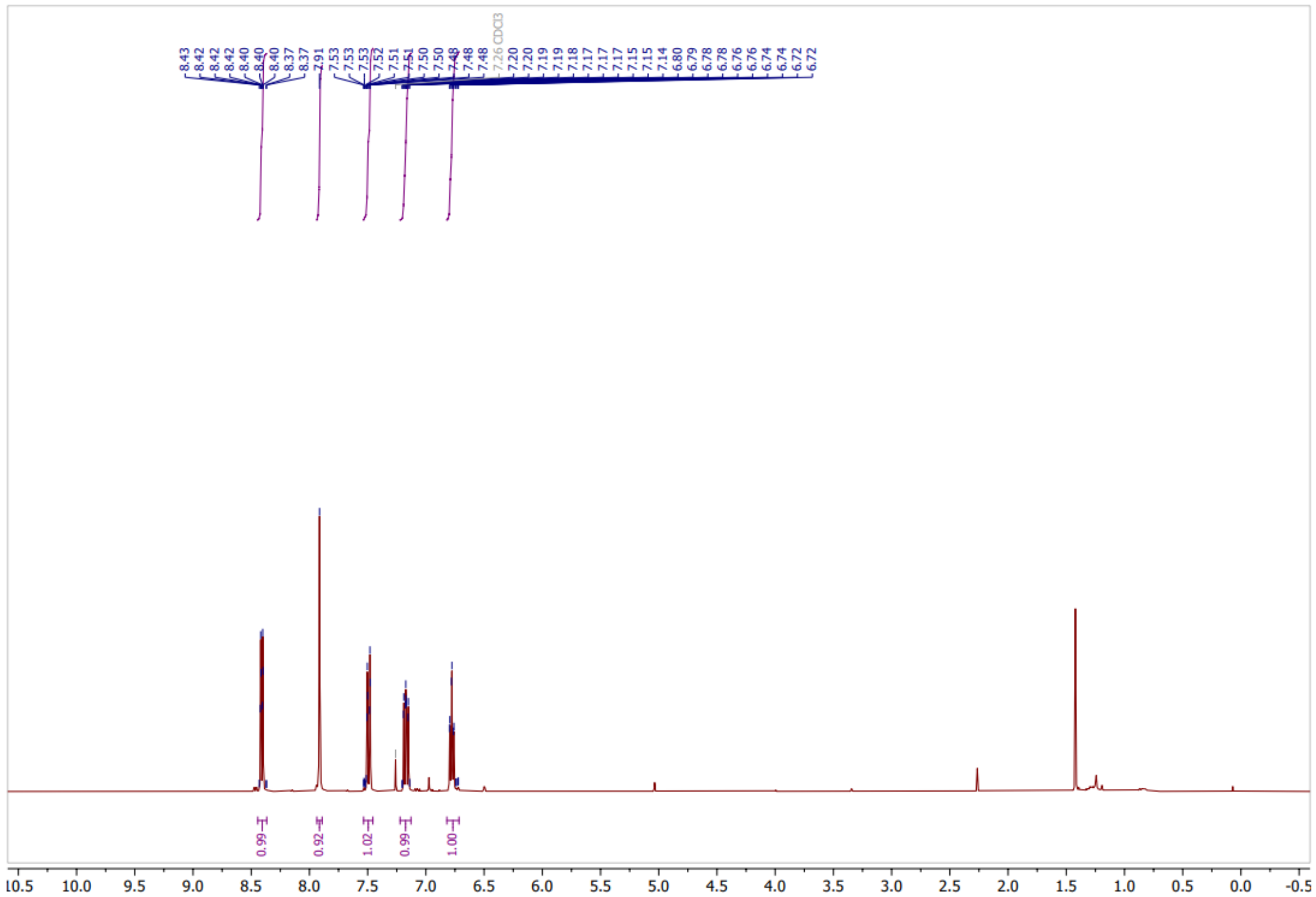

^{13}C-NMR: $CDCl_3$

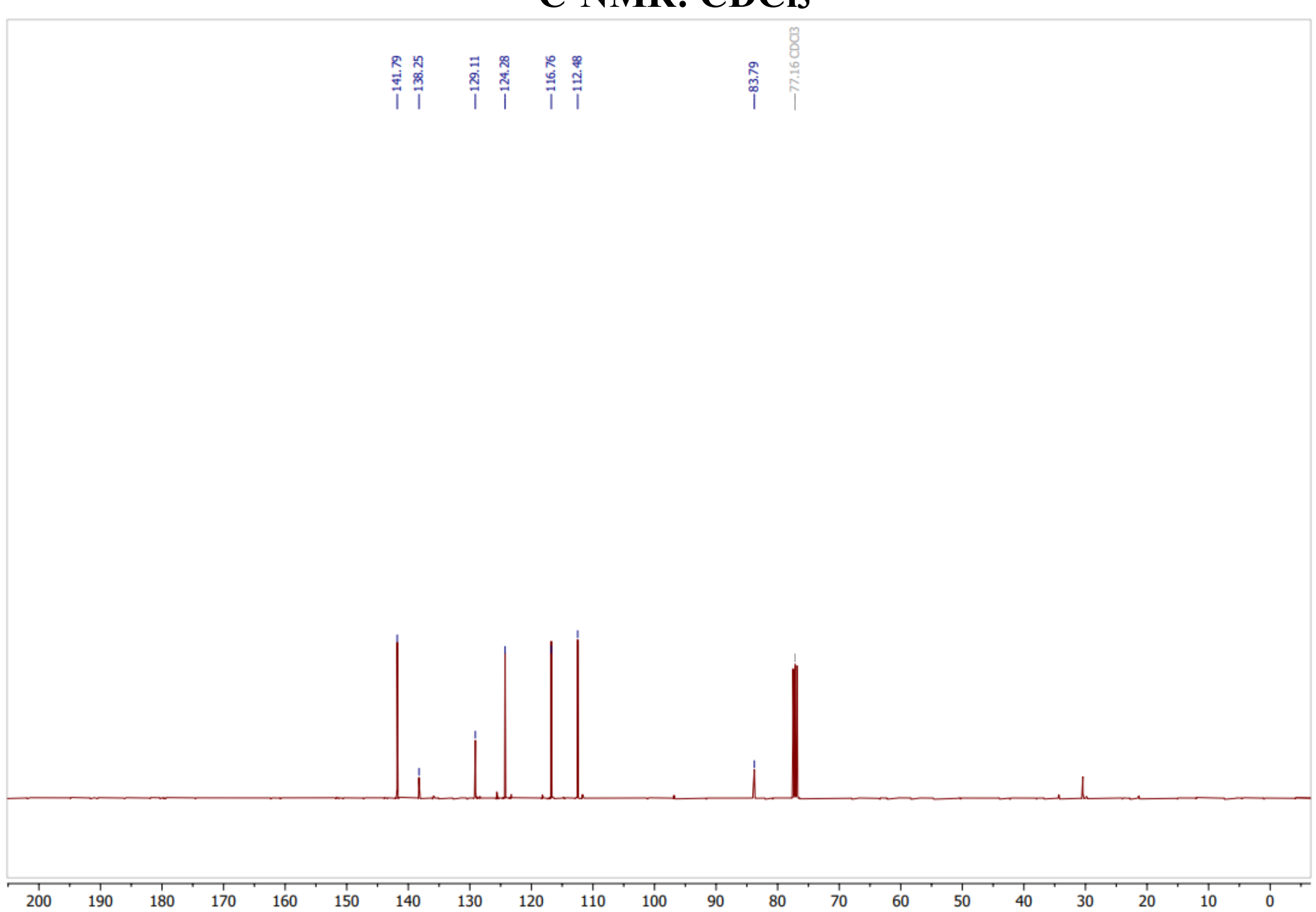

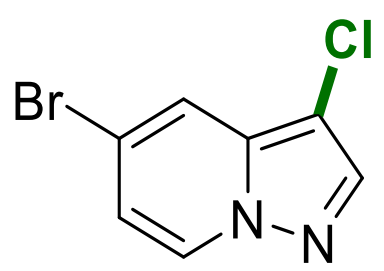

5-bromo-3-chloropyrazolo[1,5-a]pyridine (48)

^{1}H-NMR: $CDCl_3$

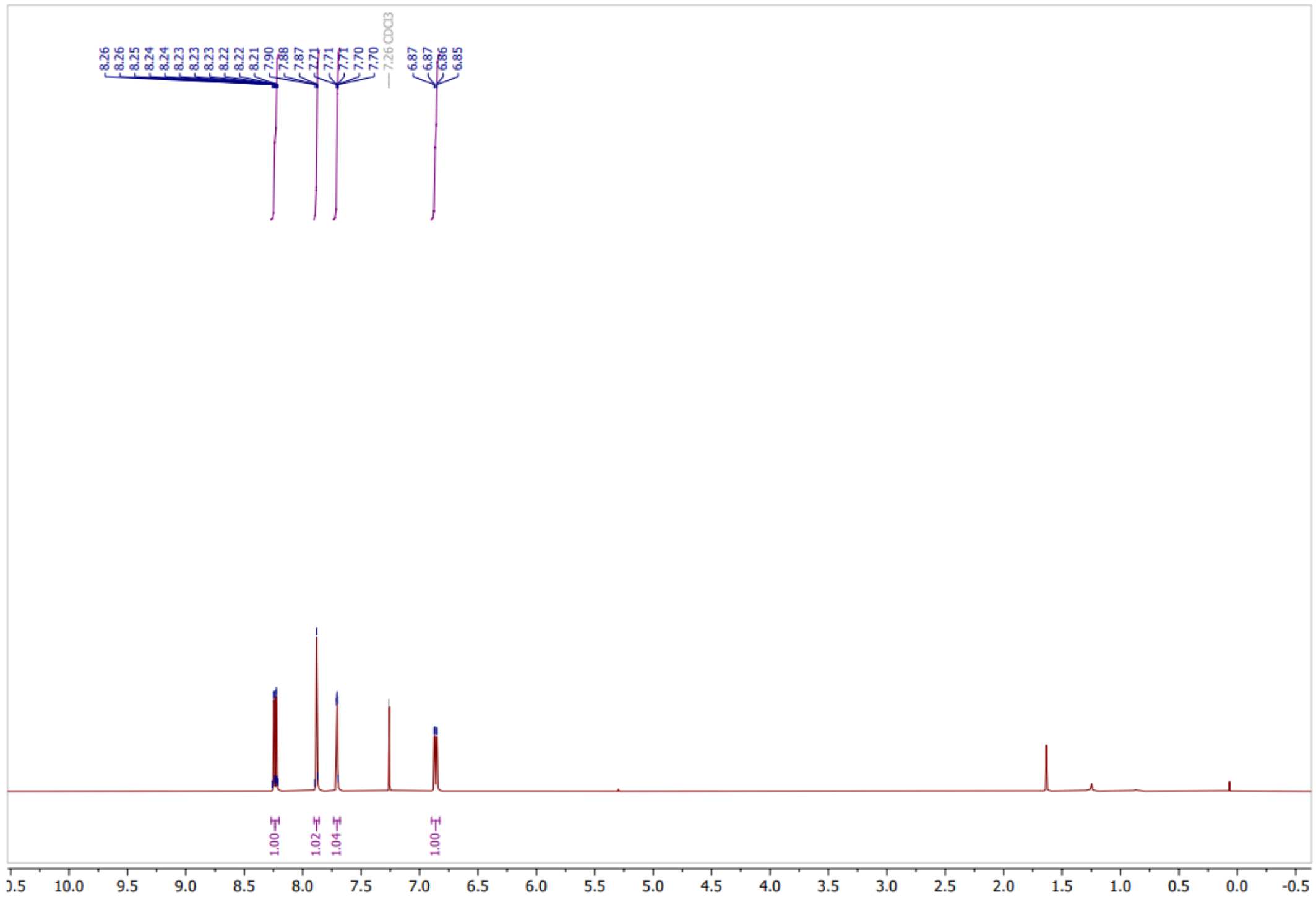

^{13}C-NMR: $CDCl_3$

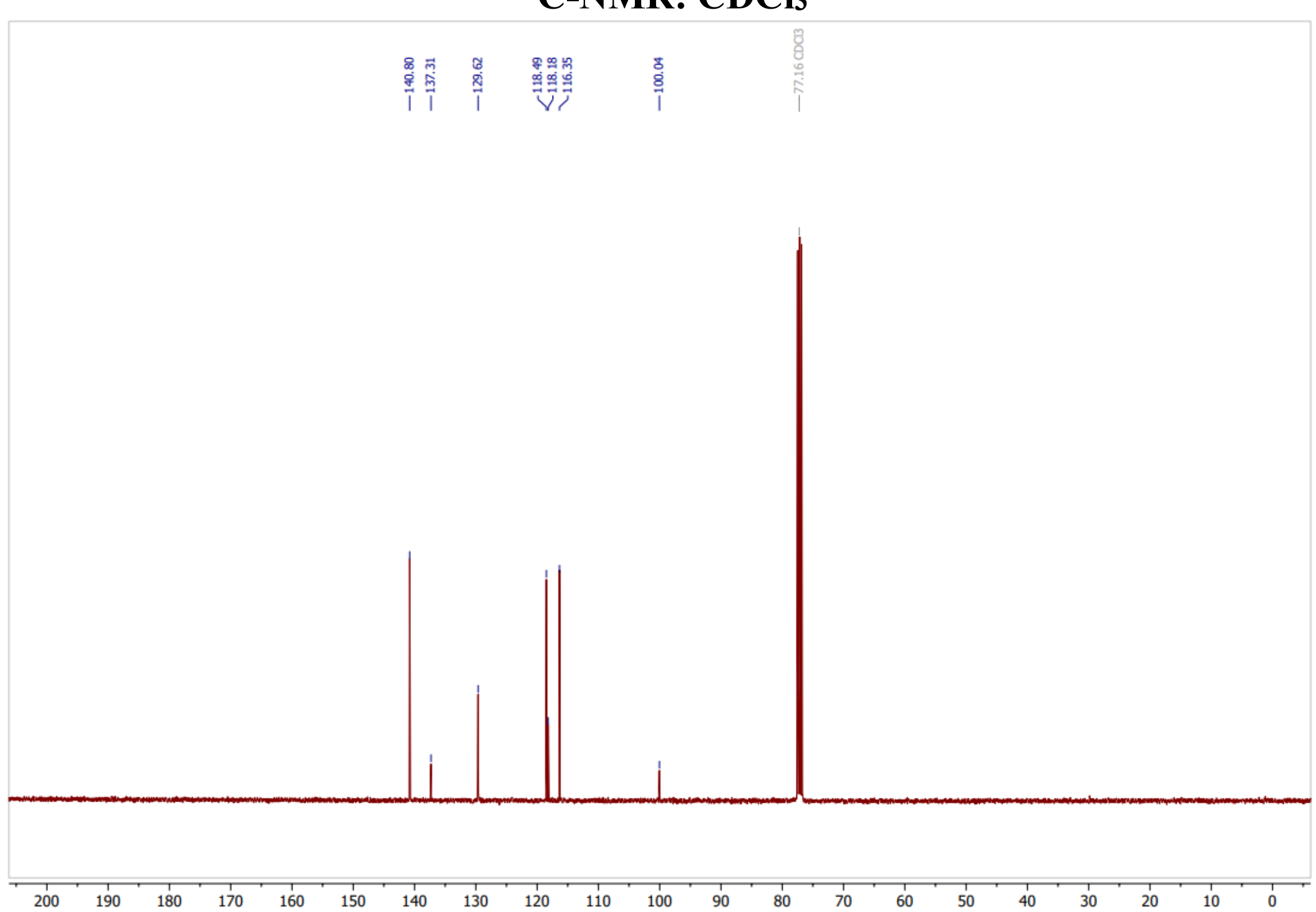

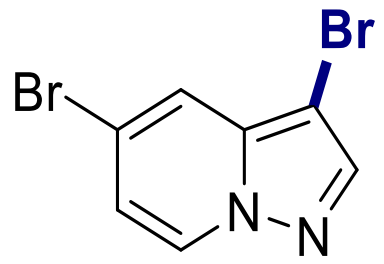

3,5-dibromopyrazolo[1,5-a]pyridine (49)

^{1}H-NMR: $CDCl_3$

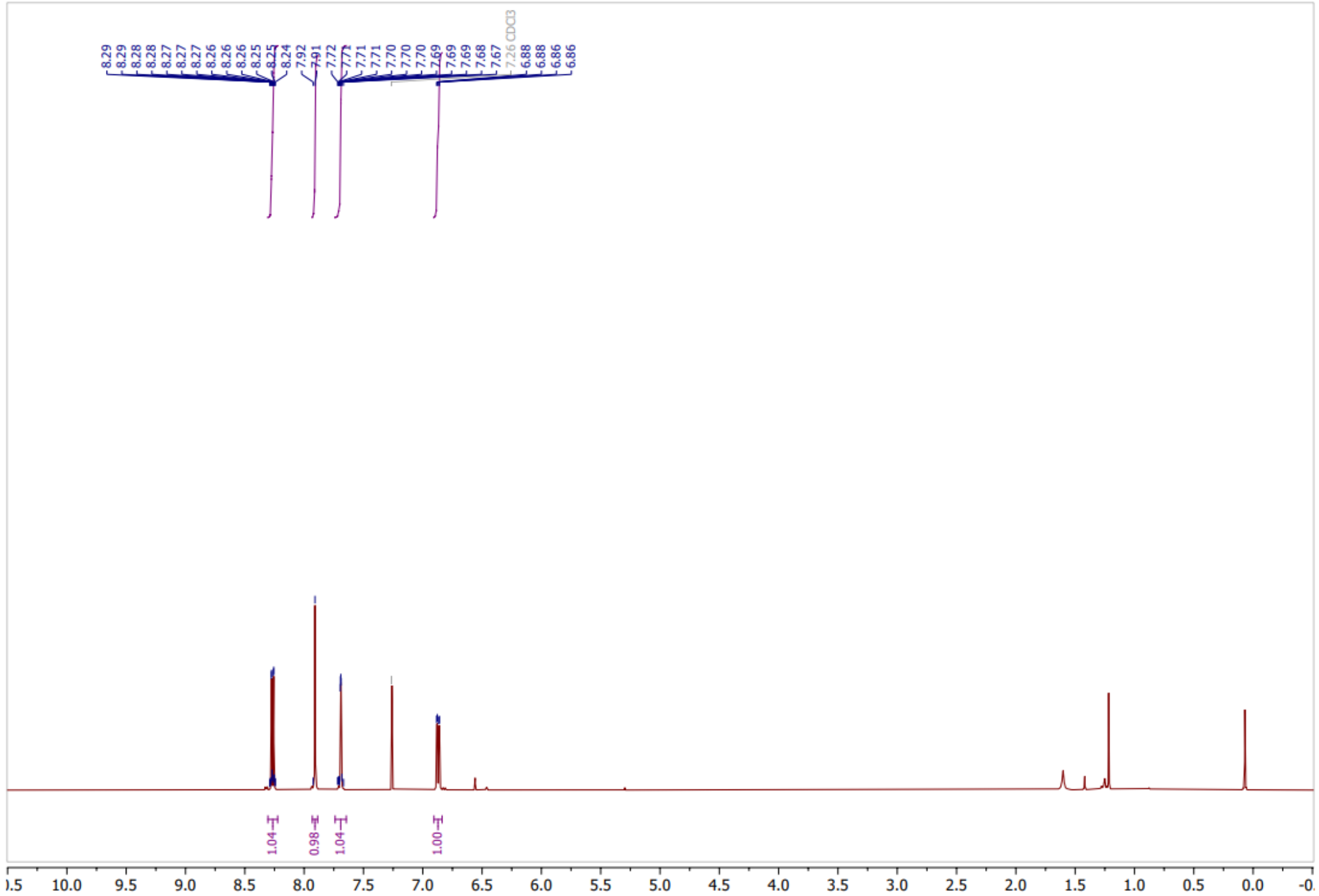

^{13}C-NMR: $CDCl_3$

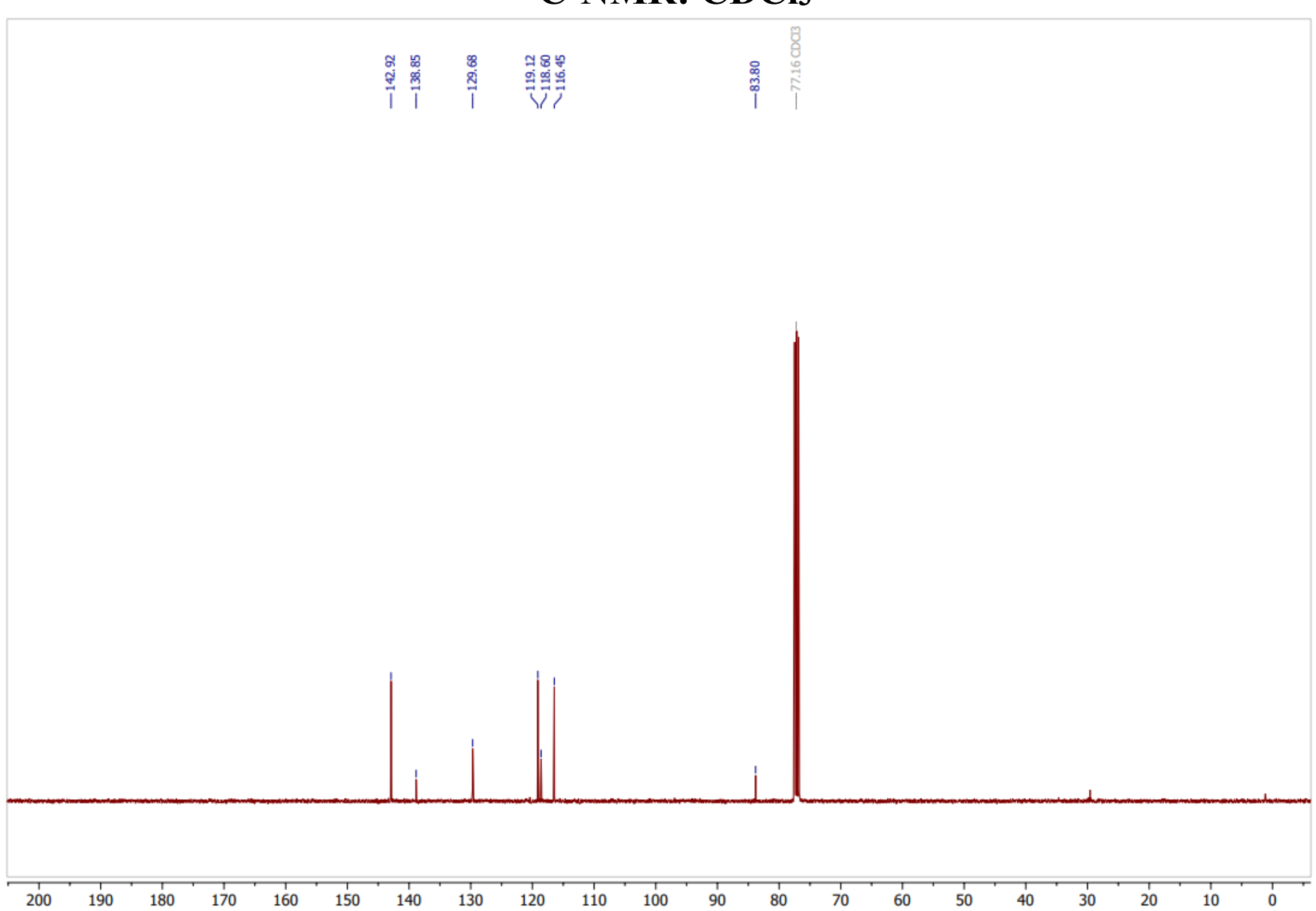

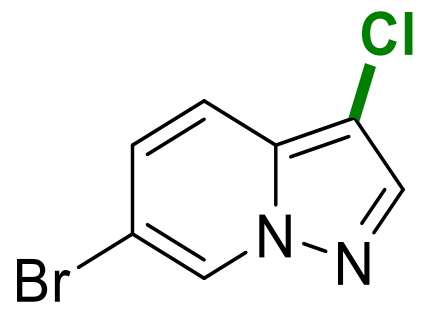

6-bromo-3-chloropyrazolo[1,5-a]pyridine (50)

^{1}H-NMR: $CDCl_3$

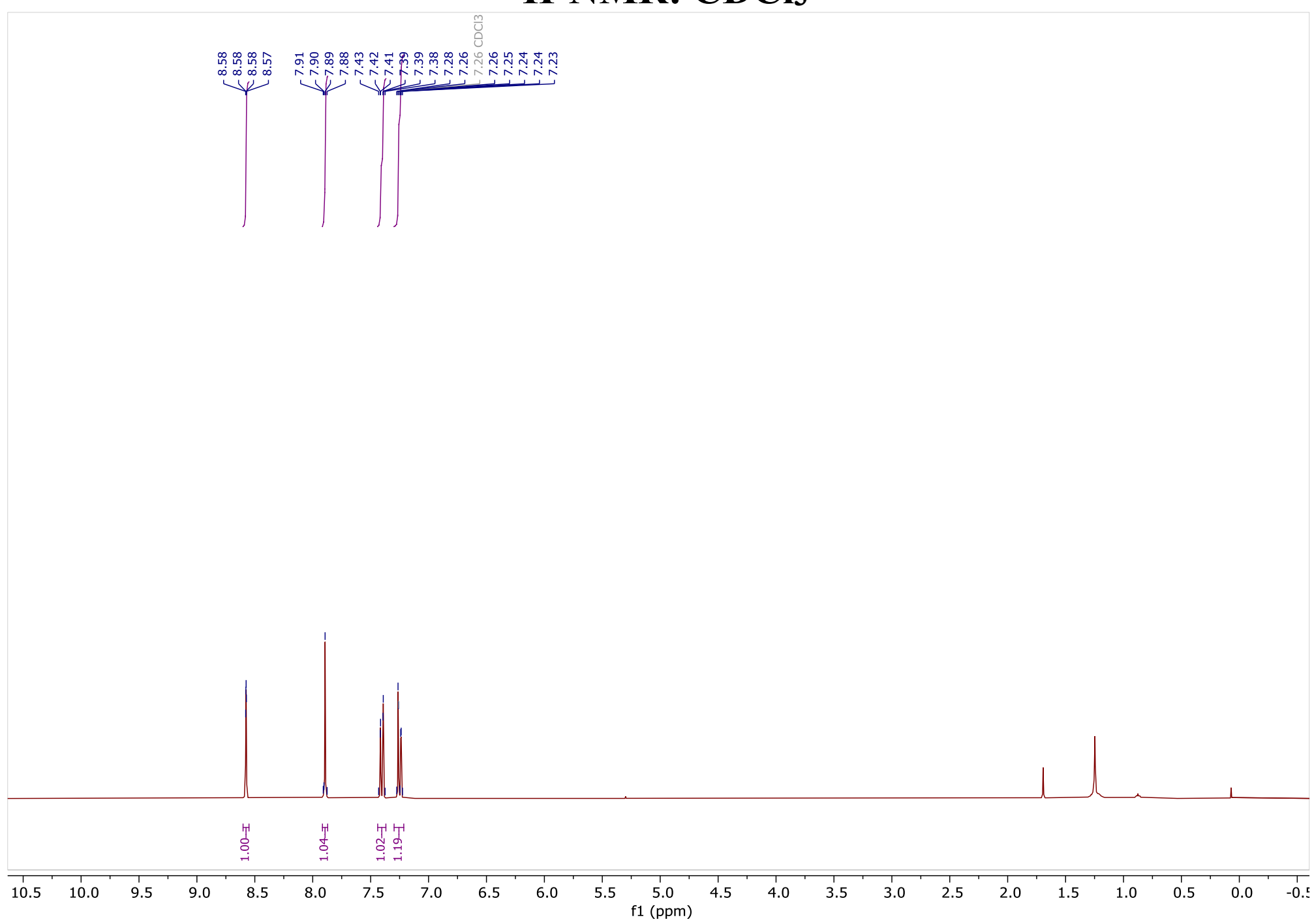

^{13}C-NMR: $CDCl_3$

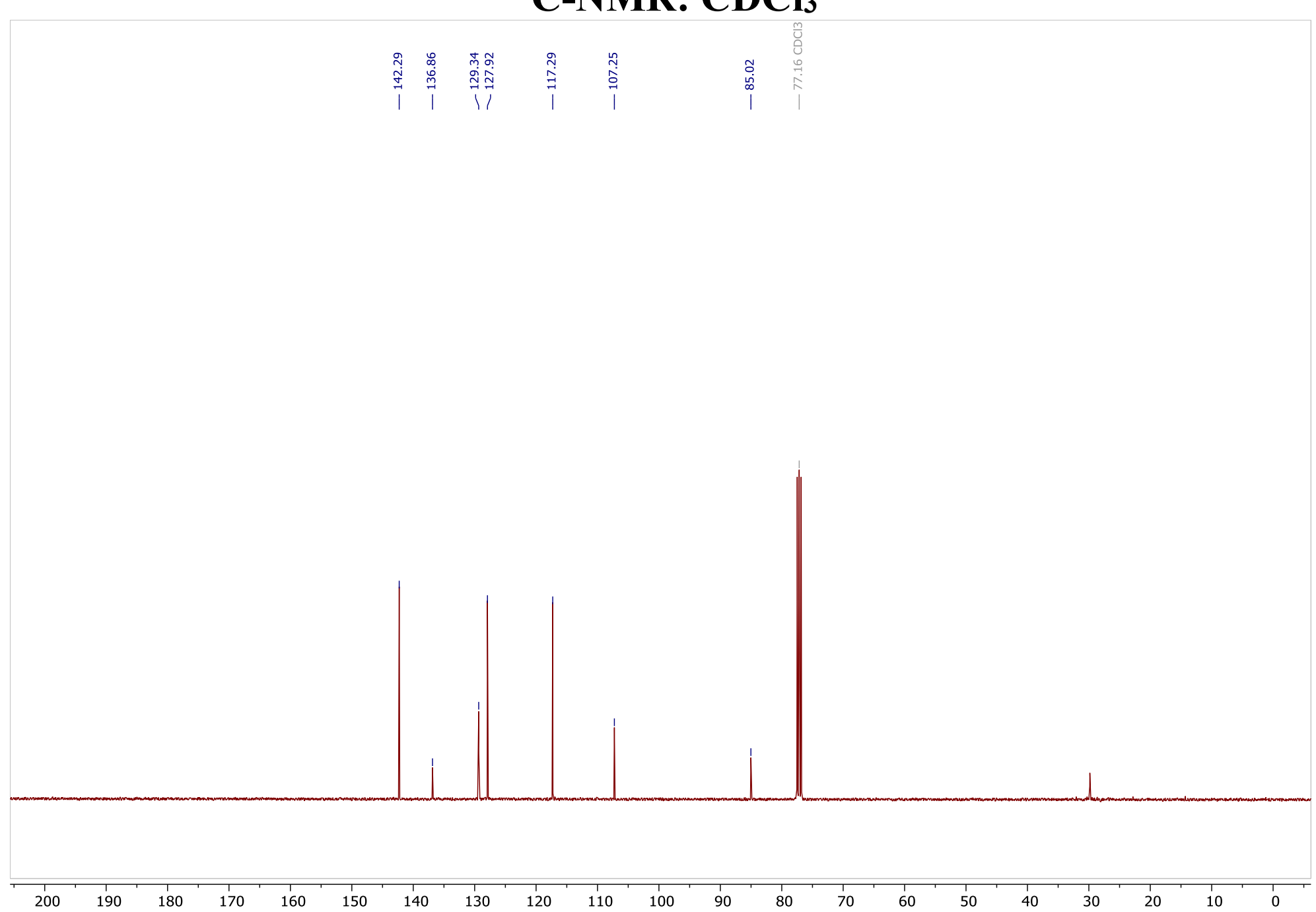

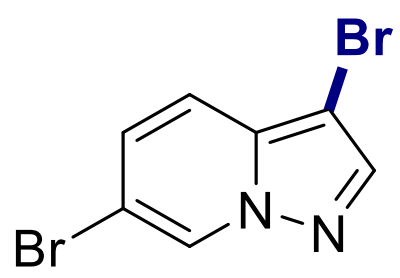

3,6-dibromopyrazolo[1,5-a]pyridine (51)

^{1}H-NMR: CDCl$_3$

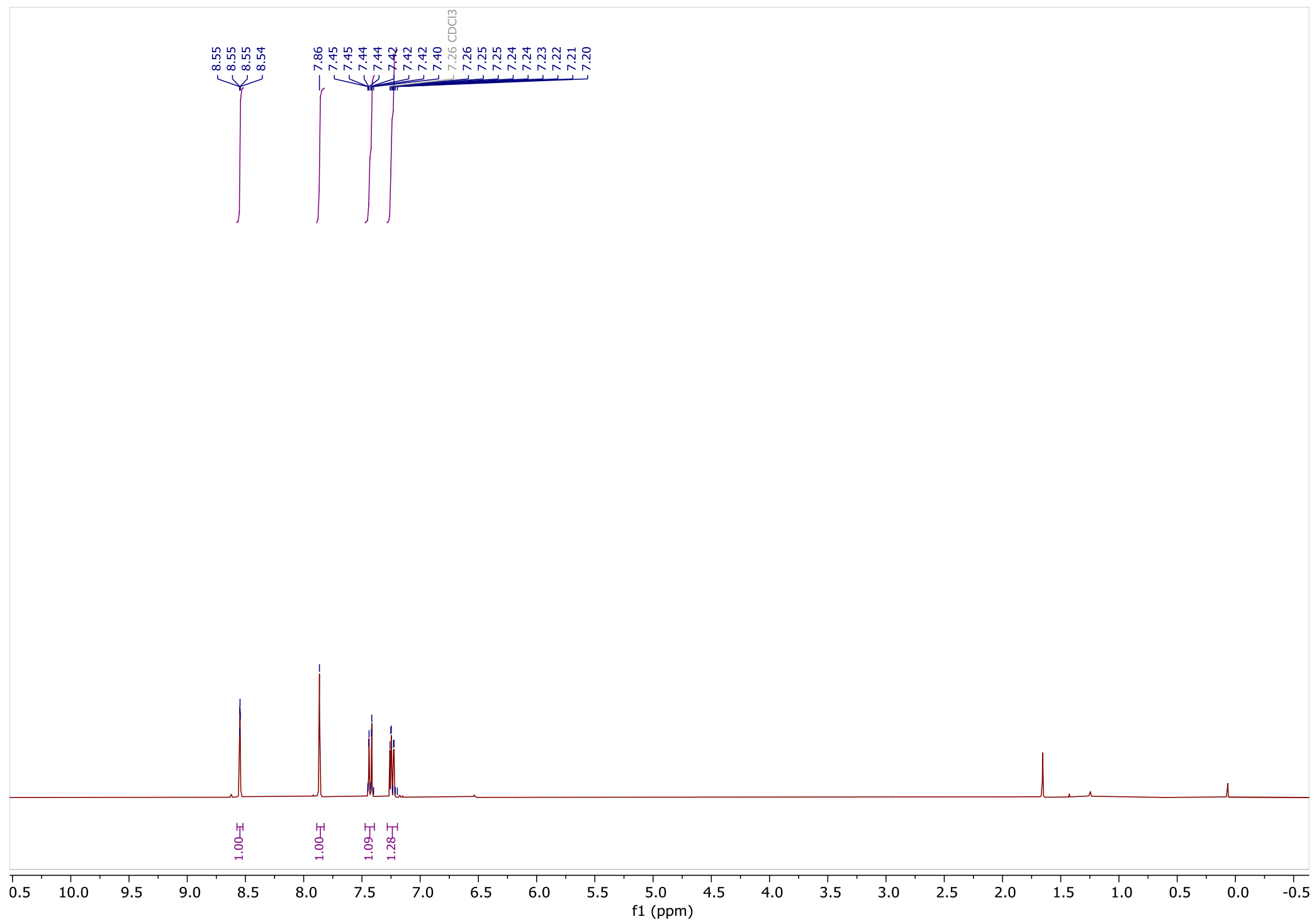

^{13}C-NMR: CDCl$_3$

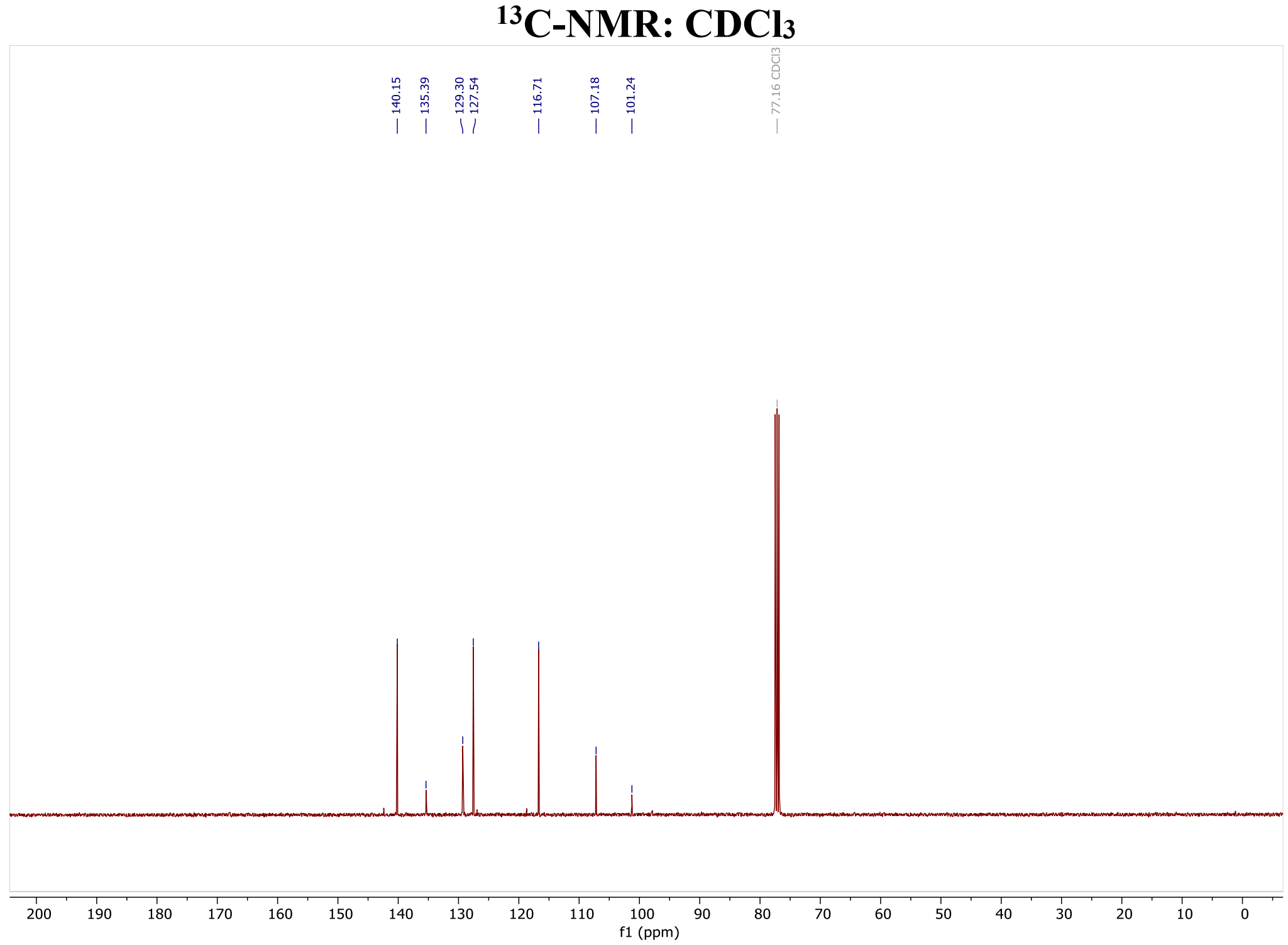

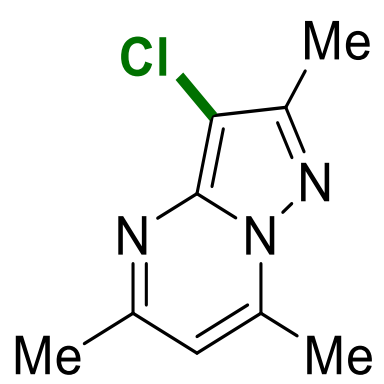

3-chloro-2,5,7-trimethylpyrazolo[1,5-a]pyrimidine (52)

^{1}H-NMR: $CDCl_3$

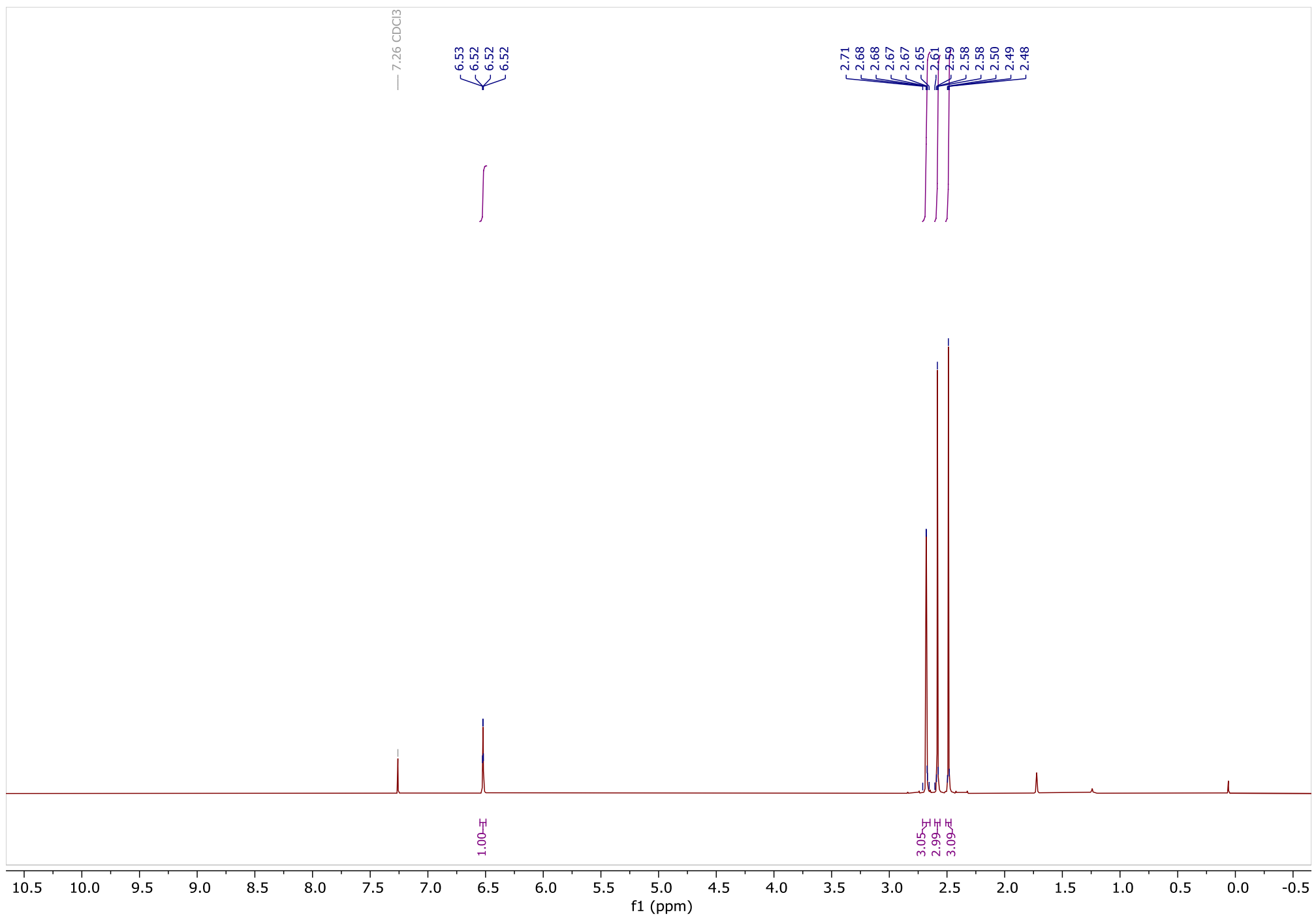

^{13}C-NMR: $CDCl_3$

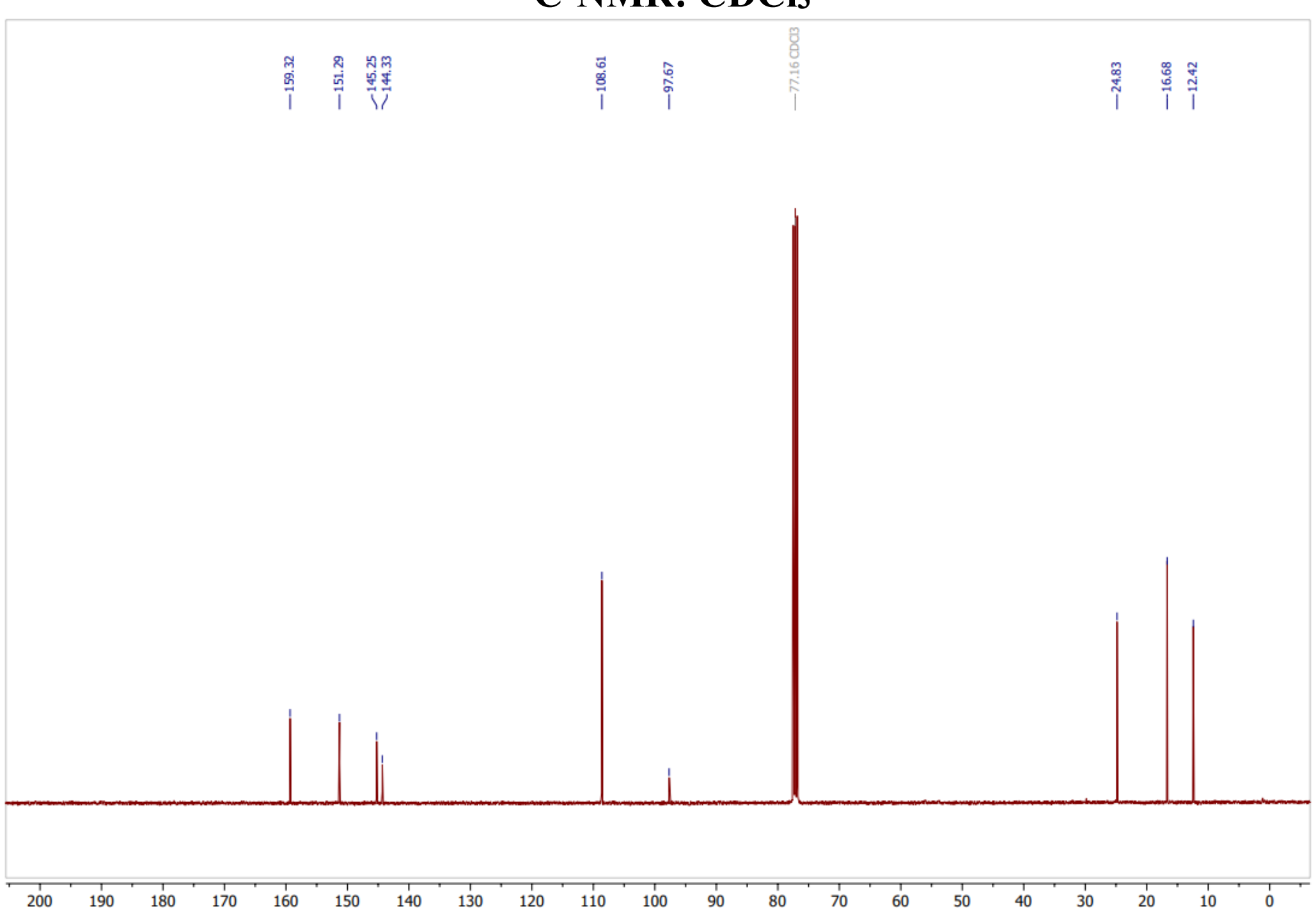

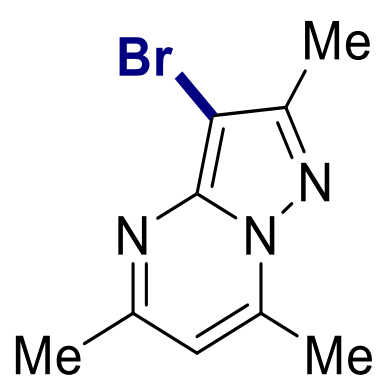

3-bromo-2,5,7-trimethylpyrazolo[1,5-a]pyrimidine (53)

^{1}H-NMR: CDCl$_3$

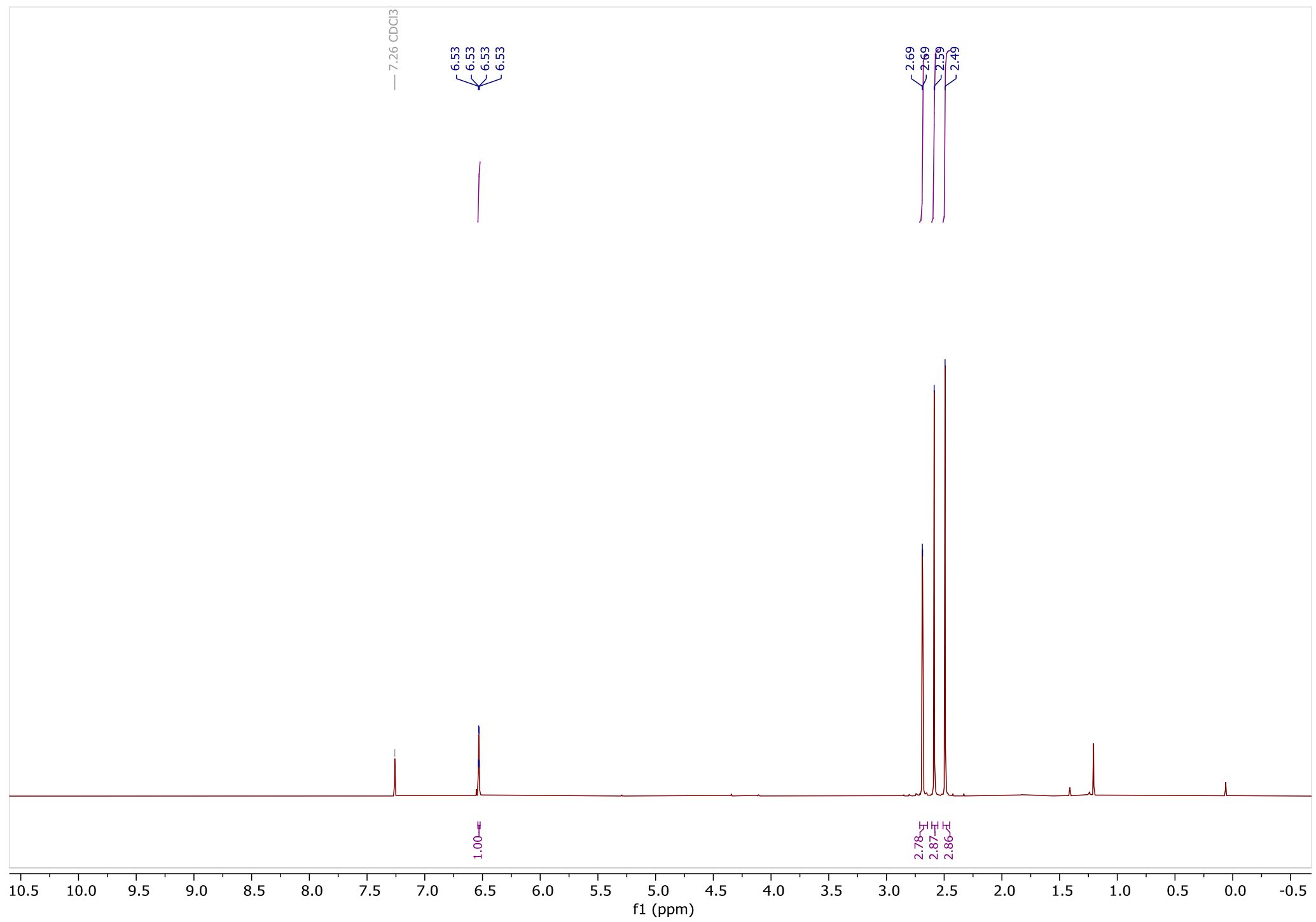

^{13}C-NMR: CDCl$_3$

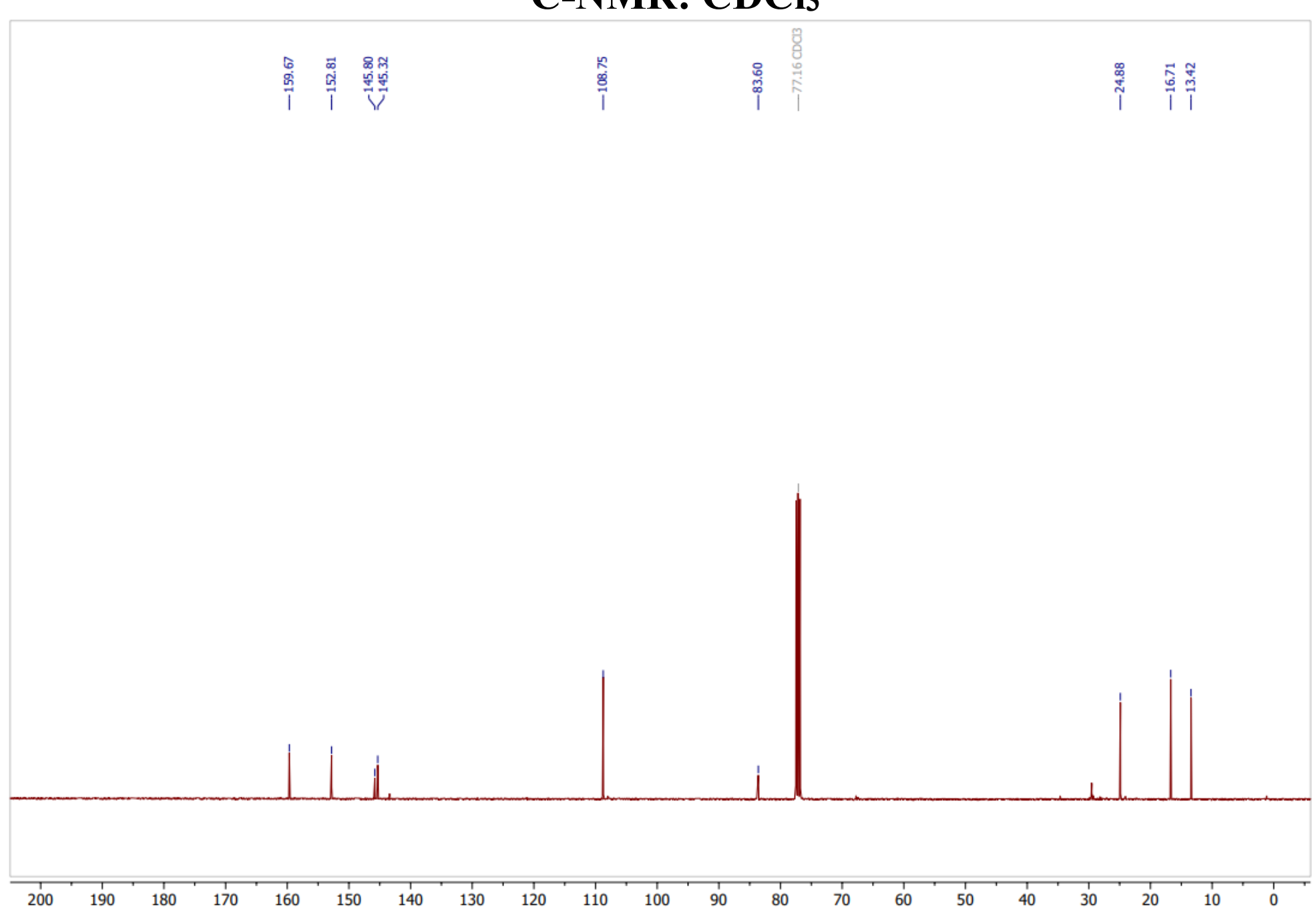

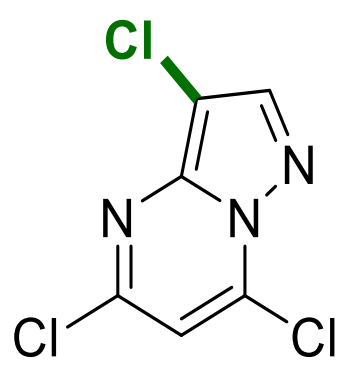

3,5,7-trichloropyrazolo[1,5-a]pyrimidine (54)

^{1}H-NMR: CDCl$_3$

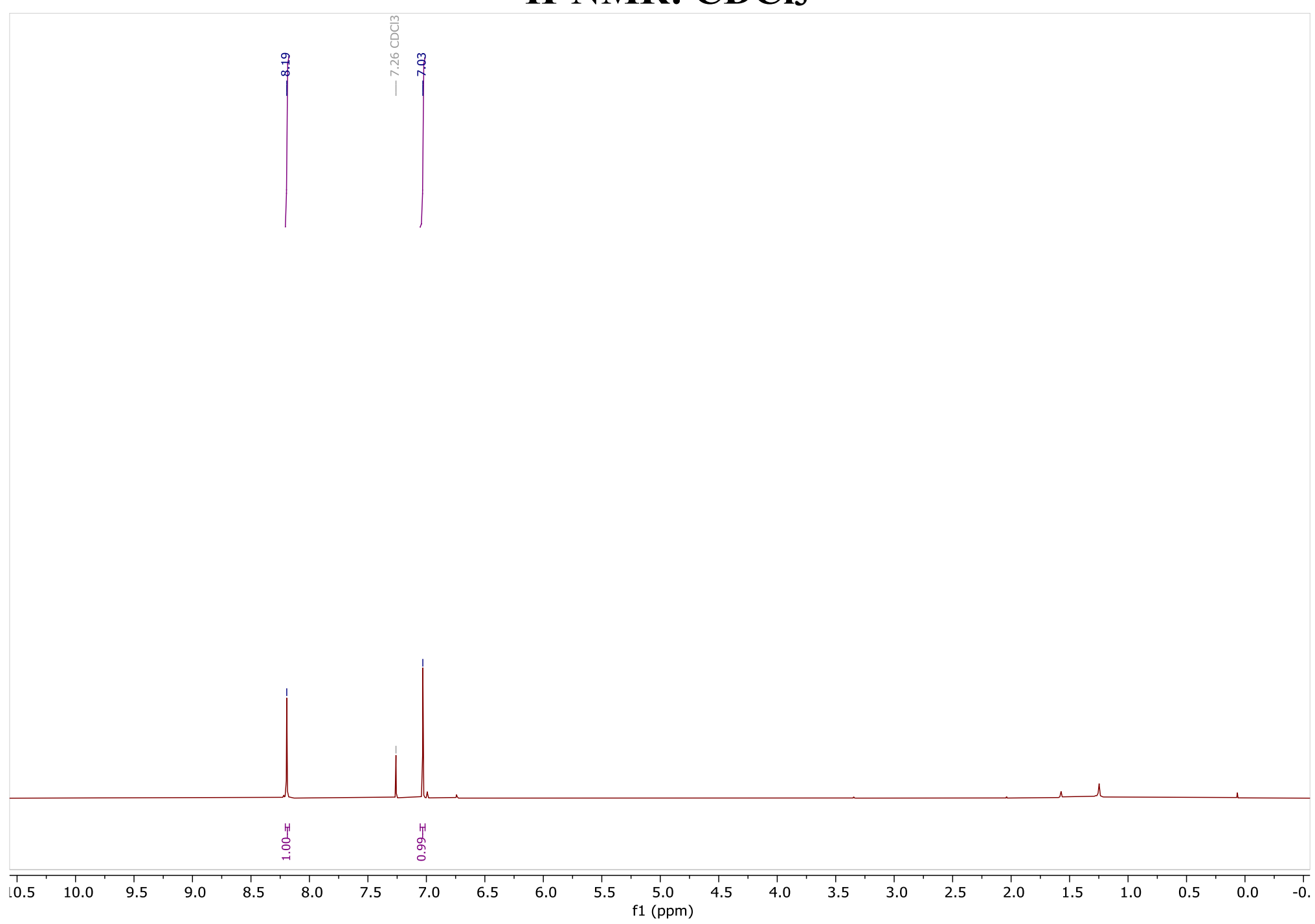

^{13}C-NMR: CDCl$_3$

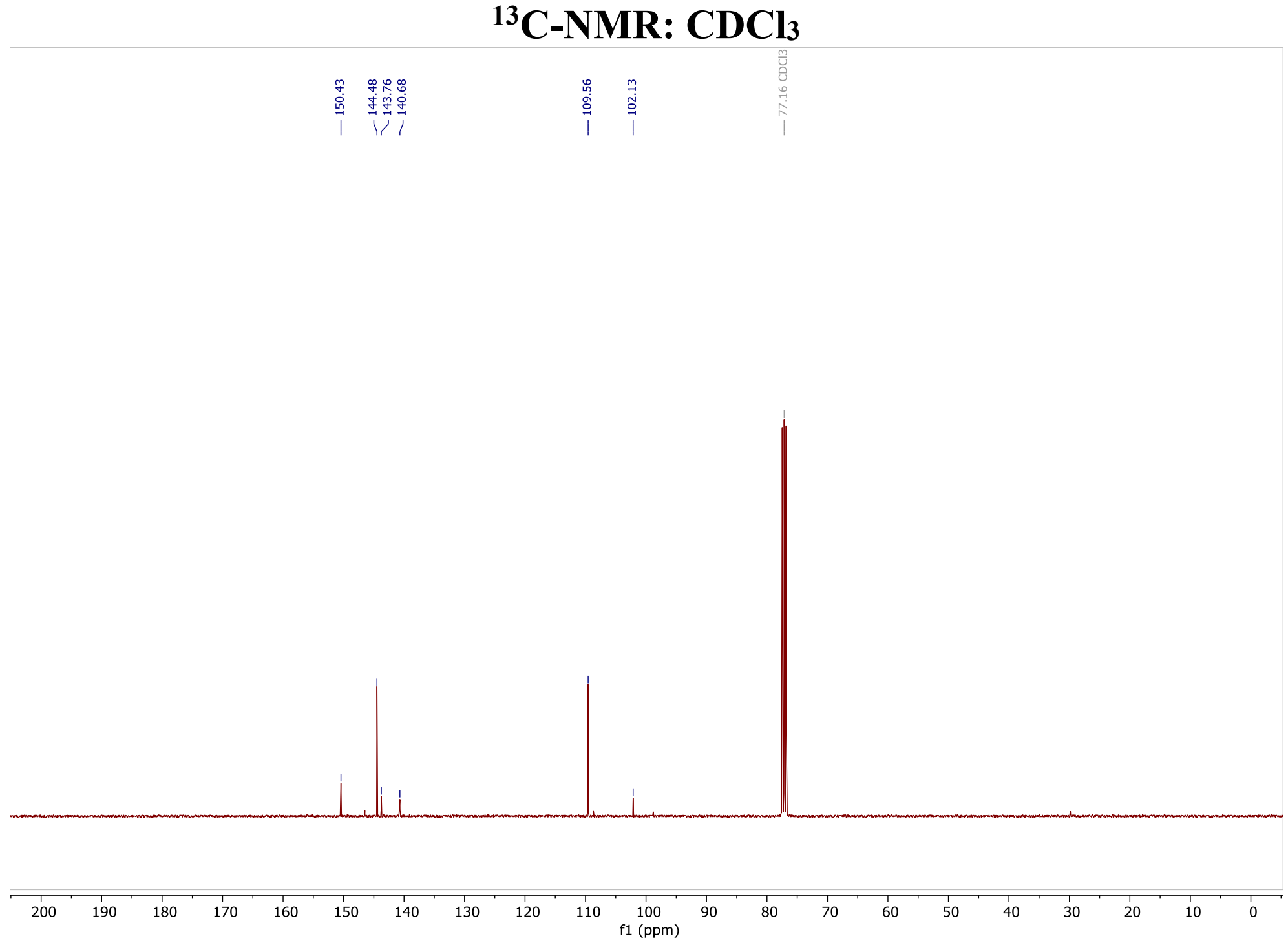

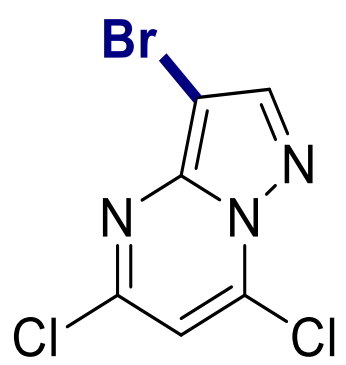

3-bromo-5,7-dichloropyrazolo[1,5-a]pyrimidine (55)

^{1}H-NMR: $CDCl_3$

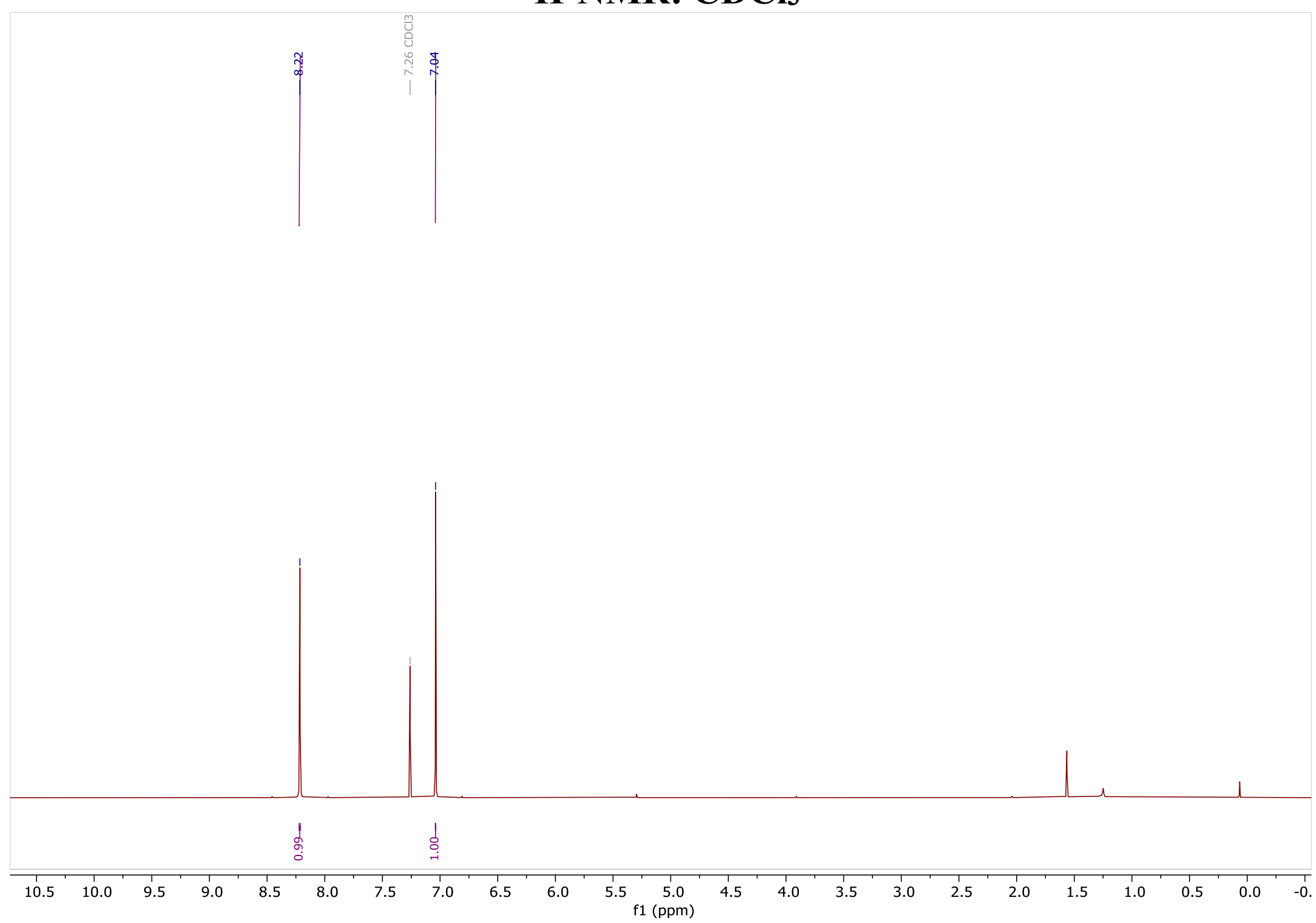

^{13}C-NMR: $CDCl_3$

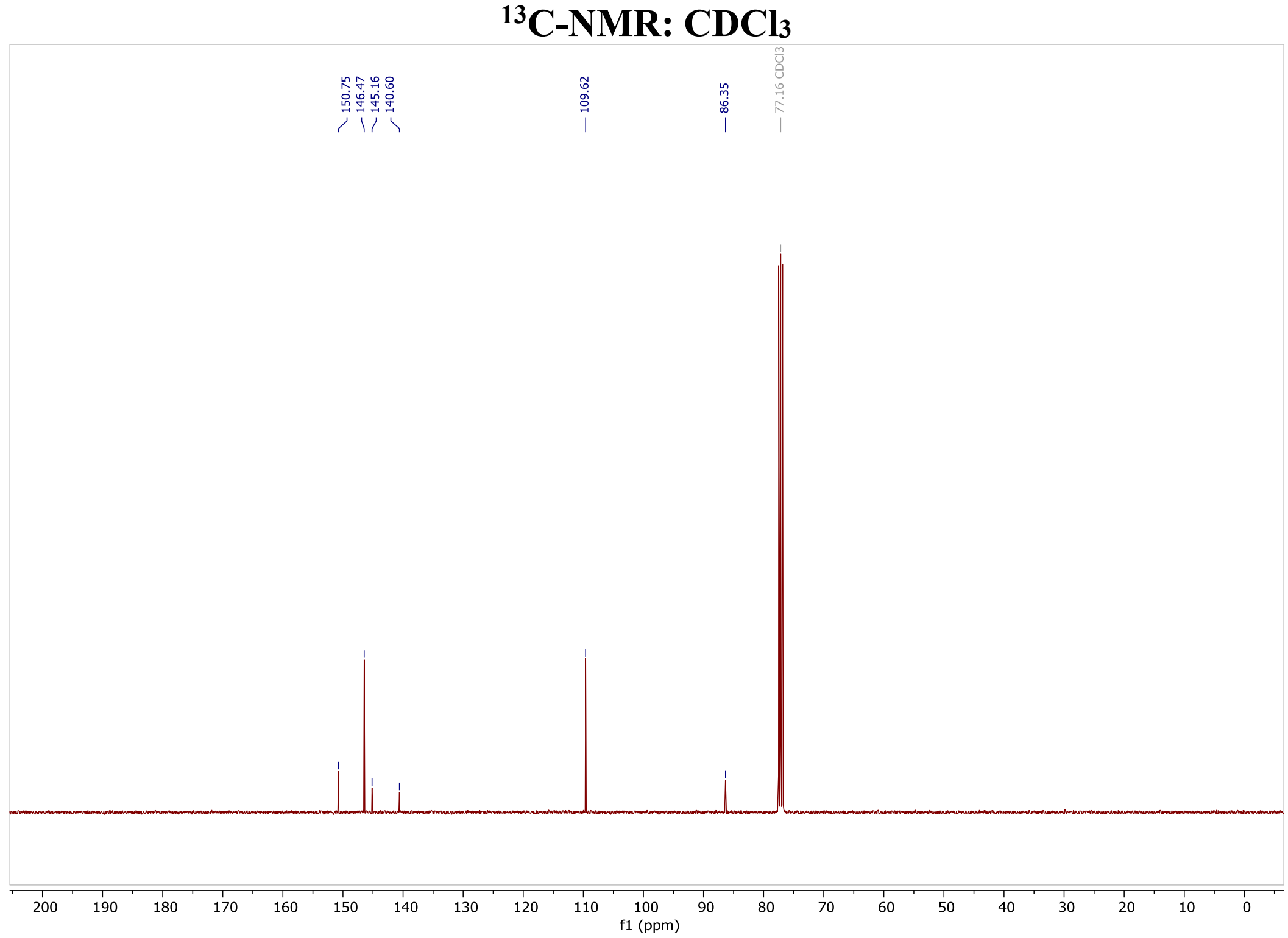

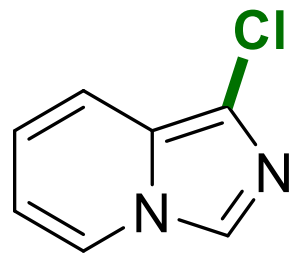

1-chloroimidazo[1,5-a]pyridine (56)

^{1}H-NMR: CDCl$_3$

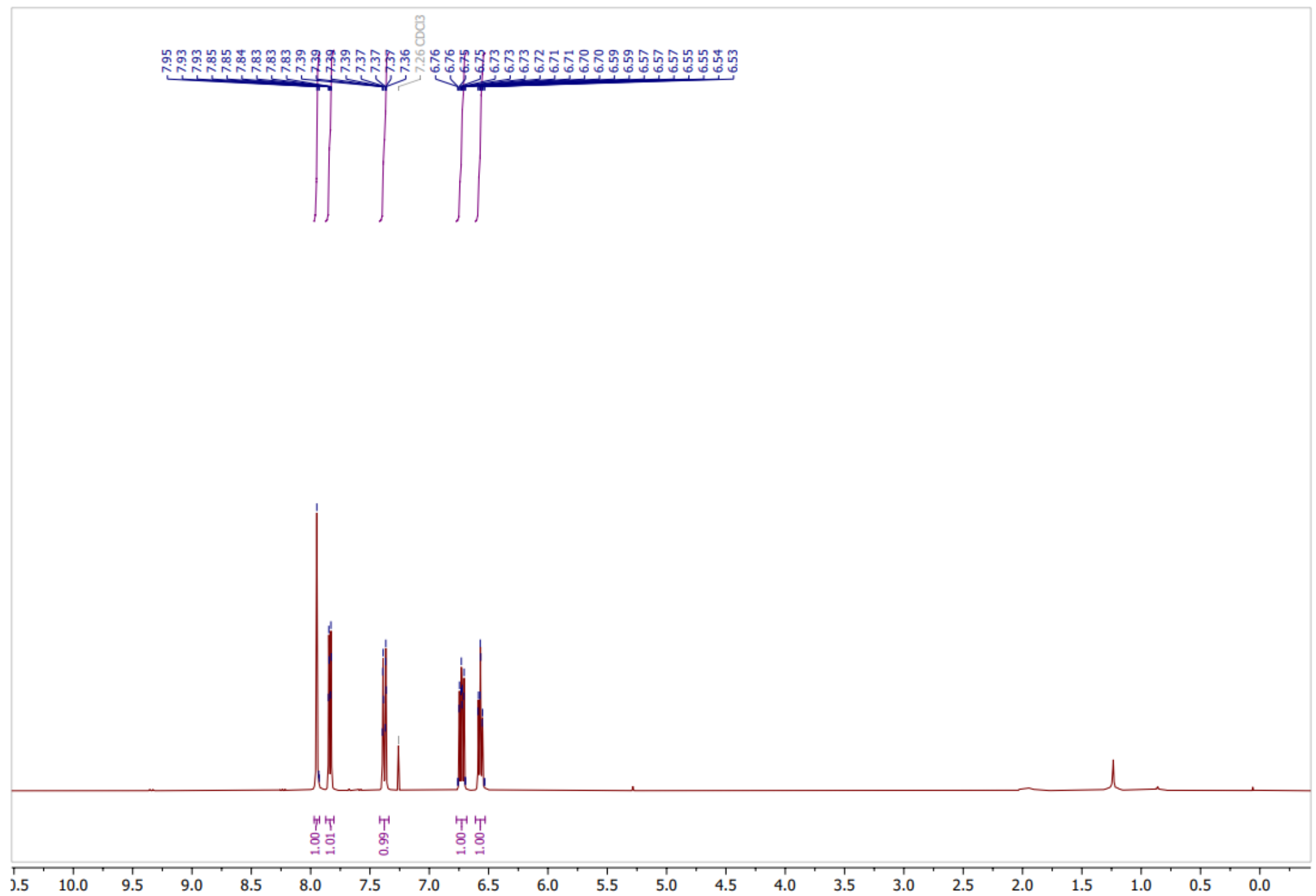

^{13}C-NMR: CDCl$_3$

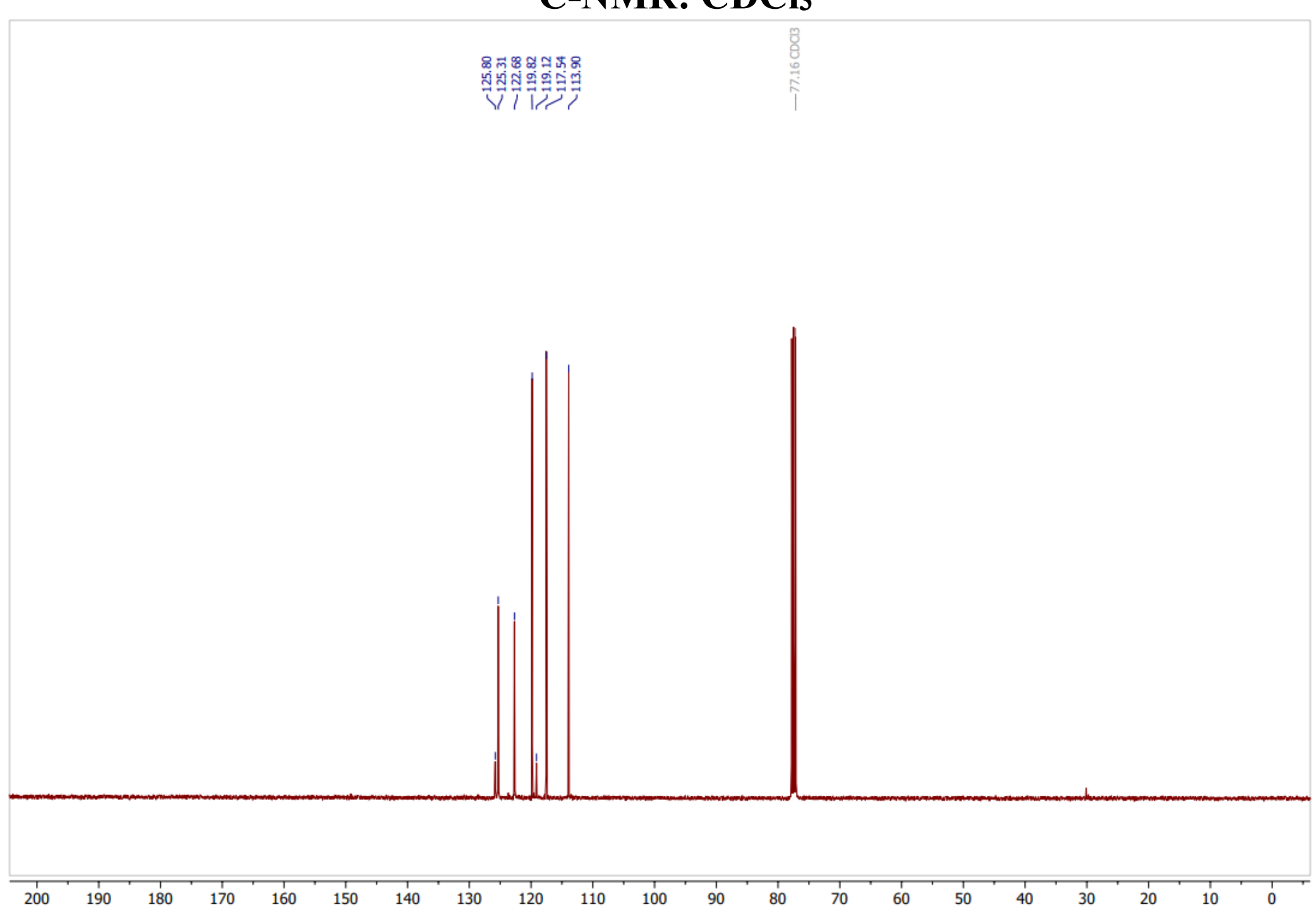

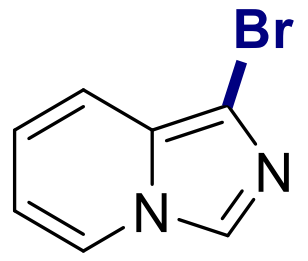

1-bromoimidazo[1,5-a]pyridine (57)

^{1}H-NMR: CDCl$_3$

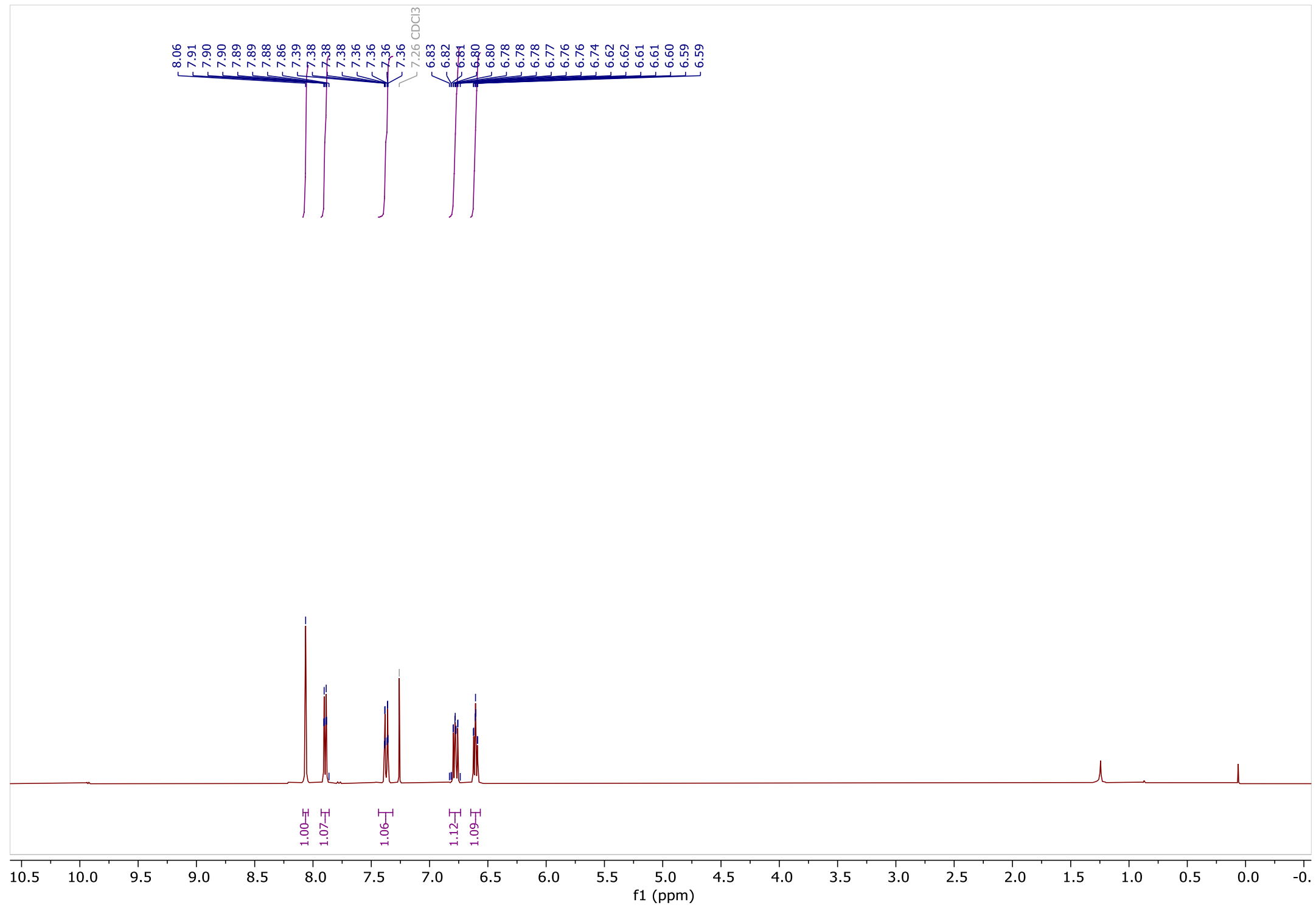

^{13}C-NMR: CDCl$_3$

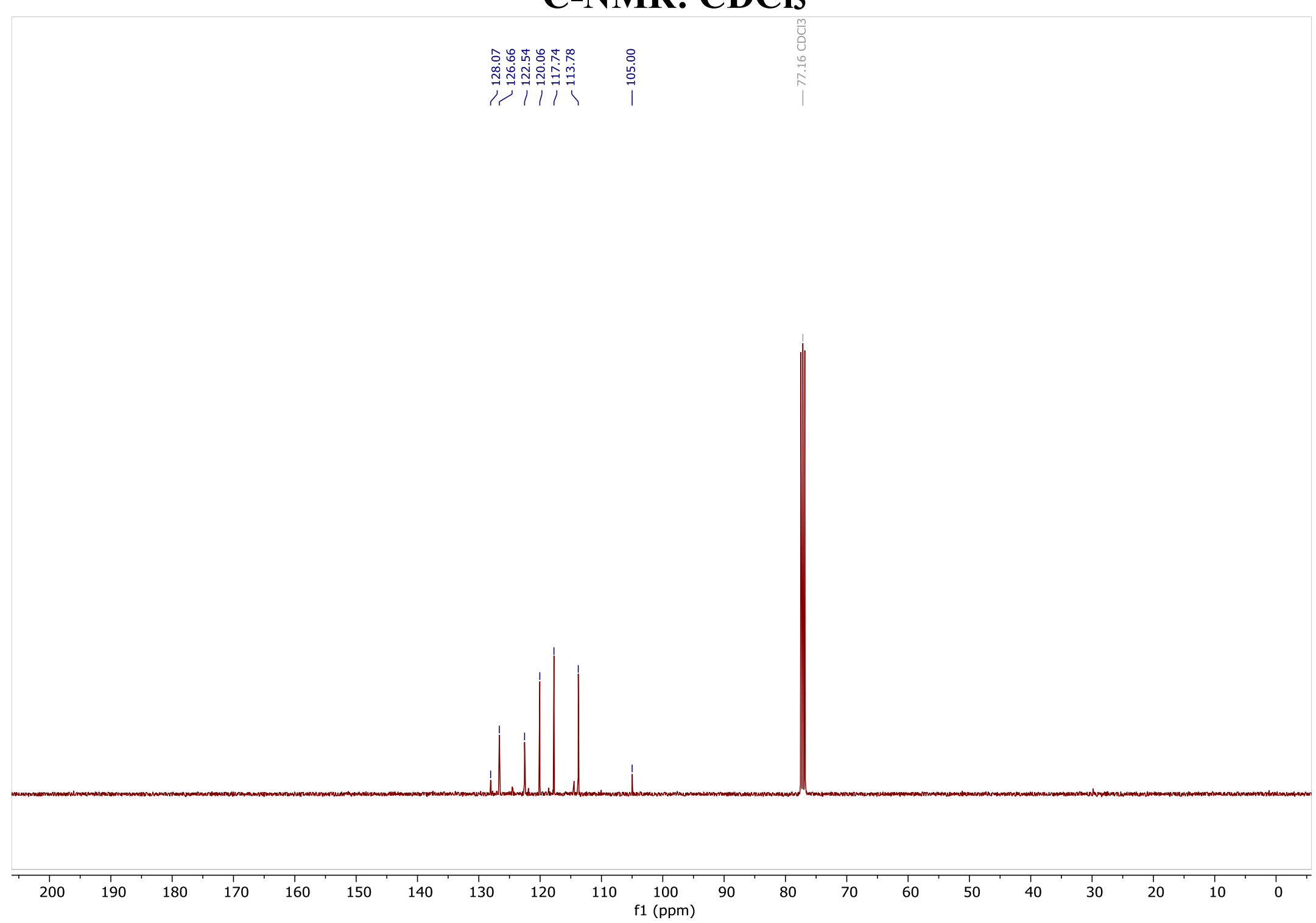

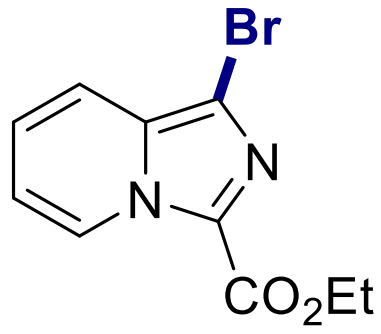

ethyl 1-bromoimidazo[1,5-a]pyridine-3-carboxylate (59)

^{1}H-NMR: CDCl$_3$

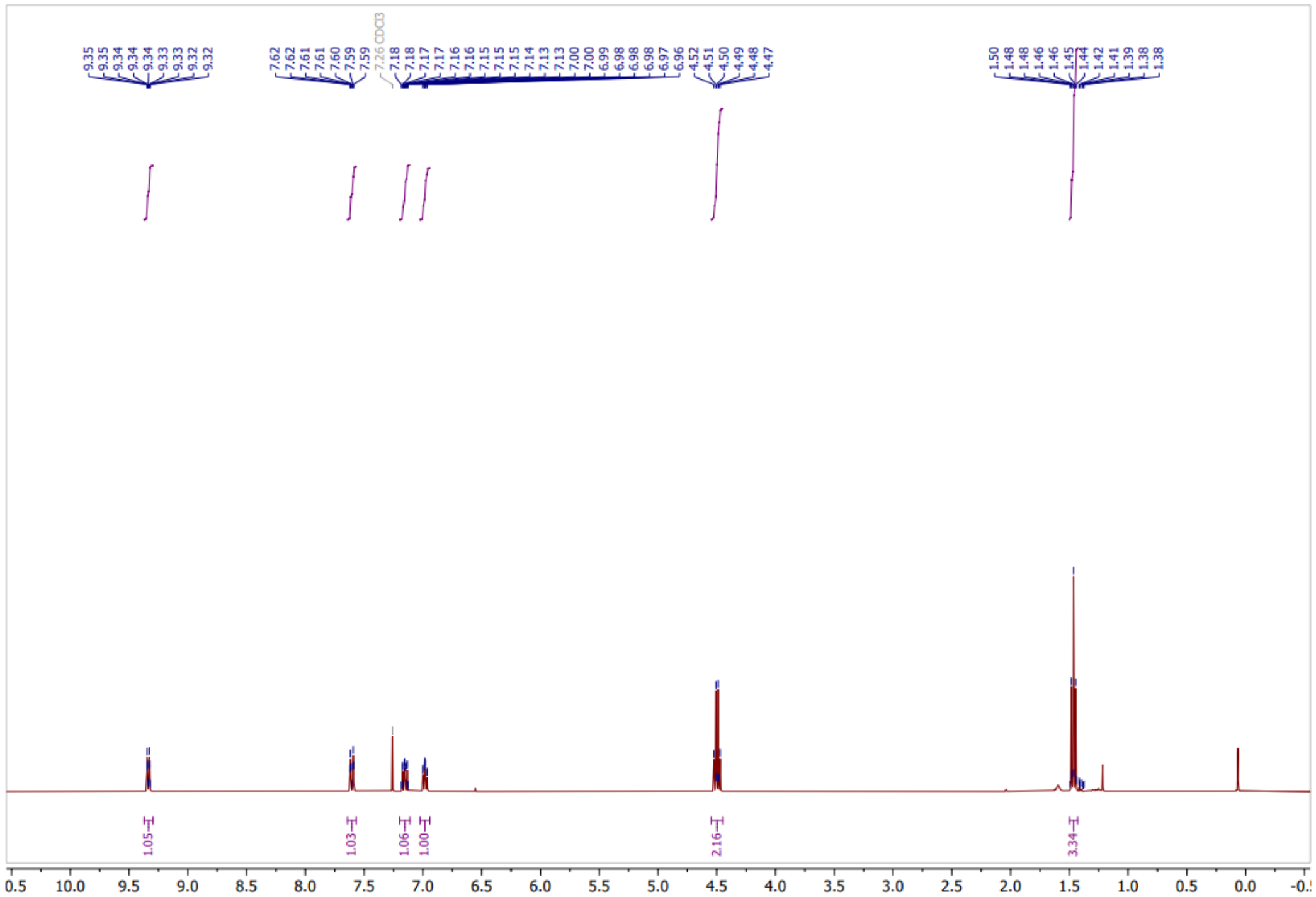

^{13}C-NMR: CDCl$_3$

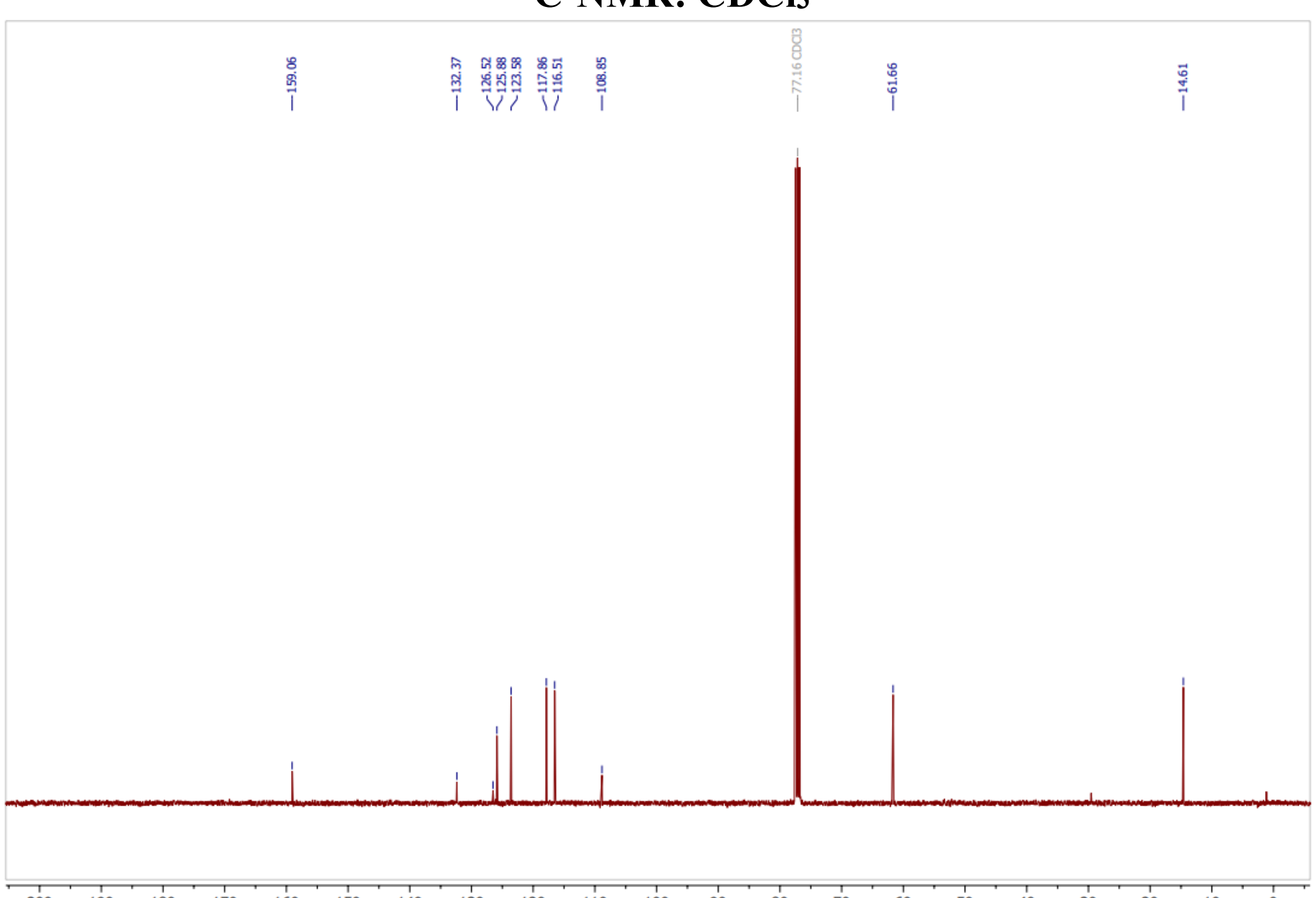

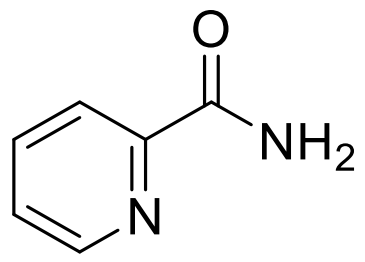

Picolinamide (60)

^{1}H-NMR: $CDCl_3$

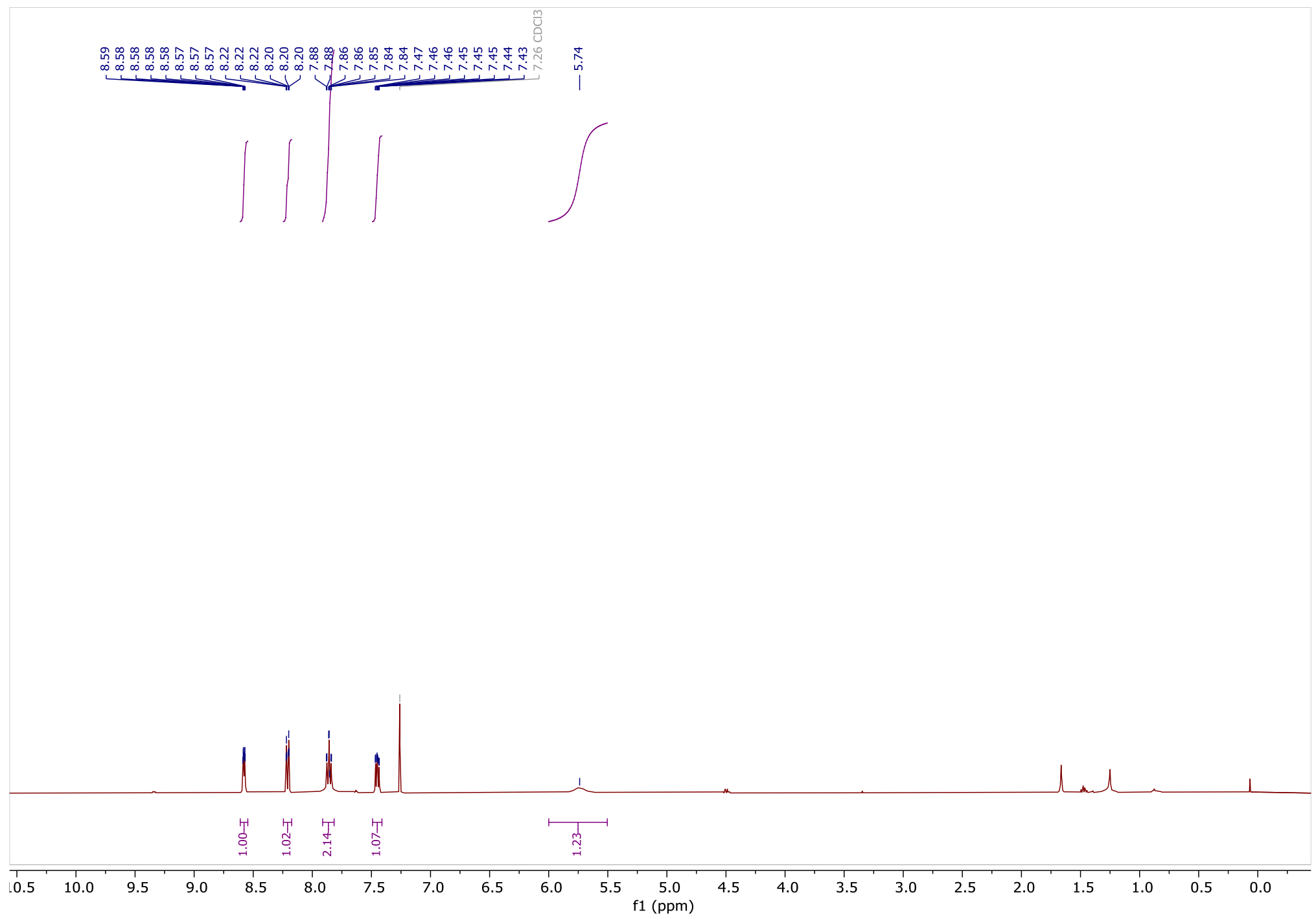

^{13}C-NMR: $CDCl_3$

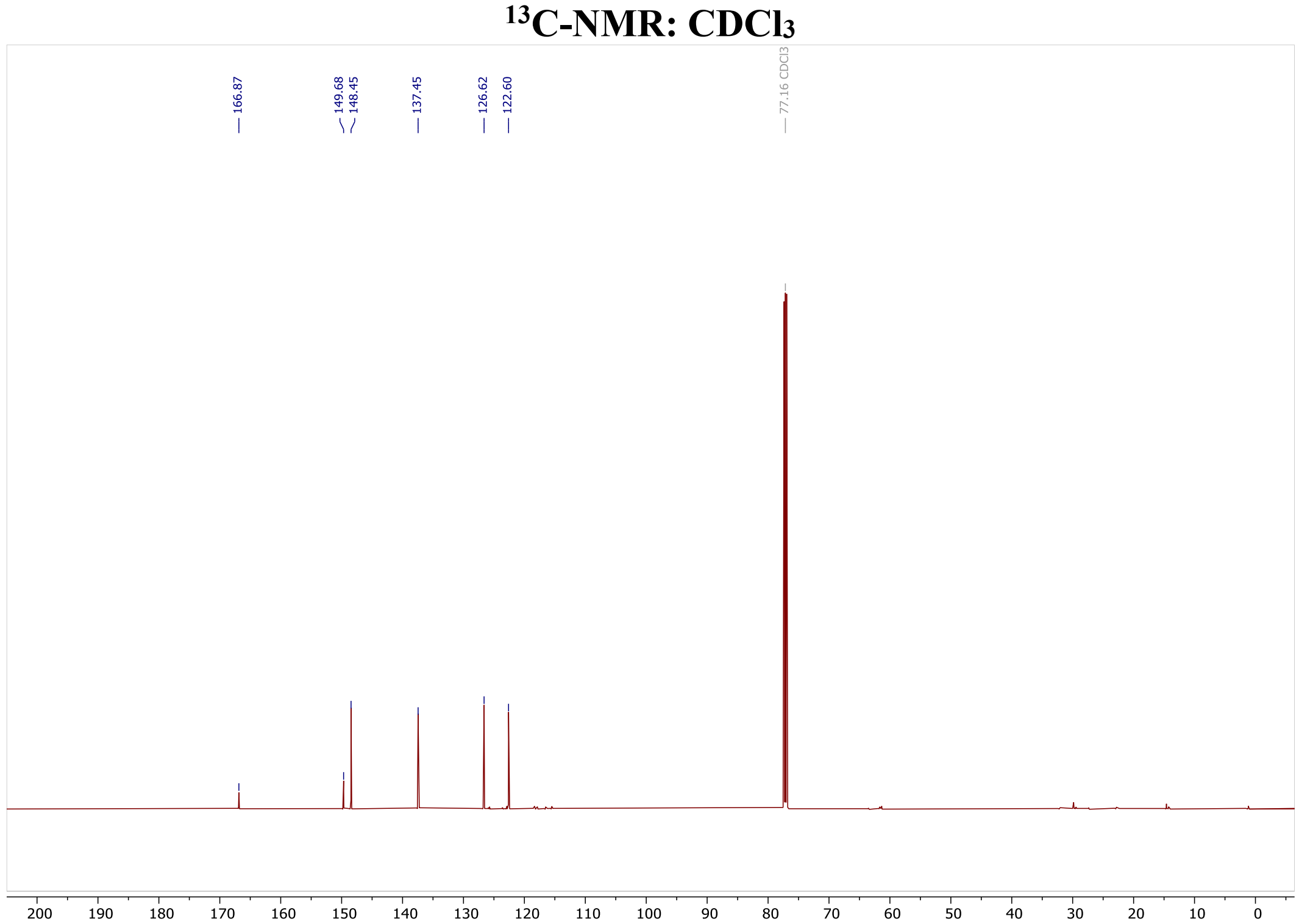

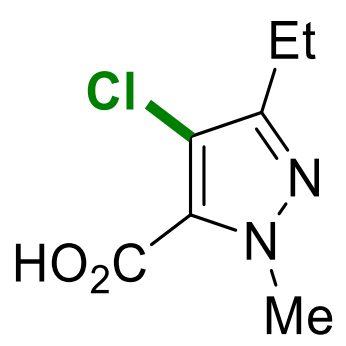

4-chloro-3-ethyl-1-methyl-1H-pyrazole-5-carboxylic acid (62)

^{1}H-NMR: DMSO-d6

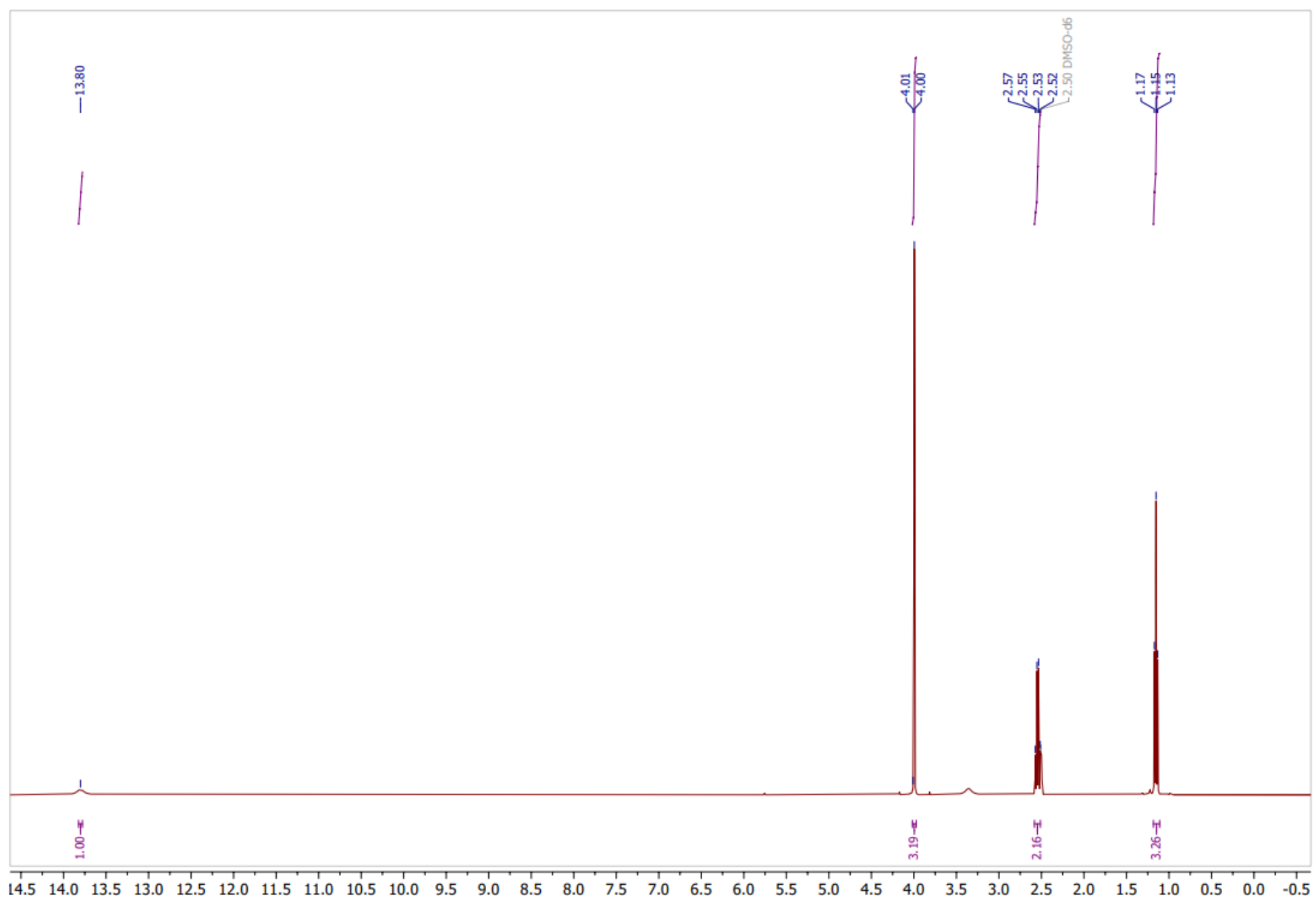

^{13}C-NMR: DMSO-d6

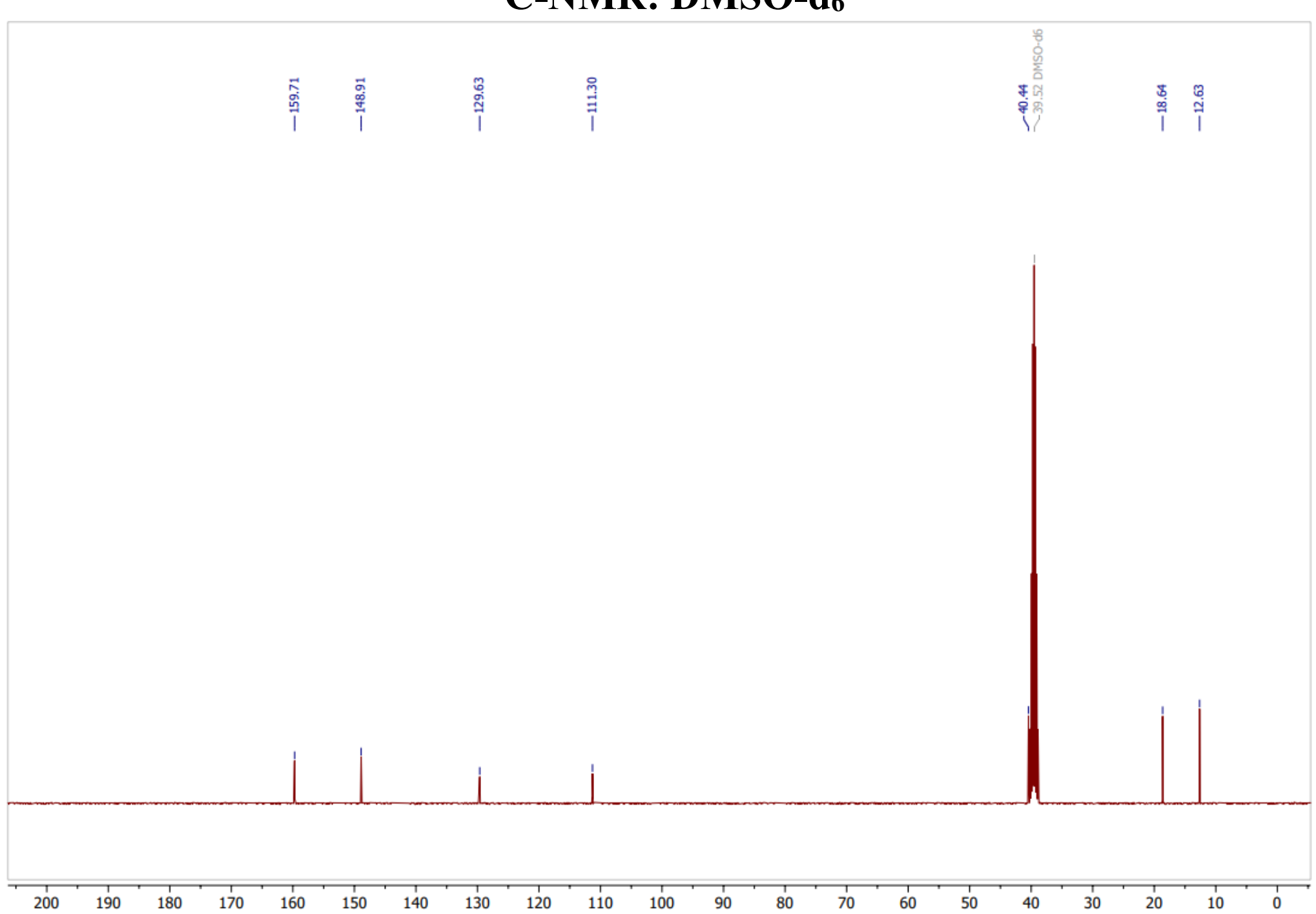

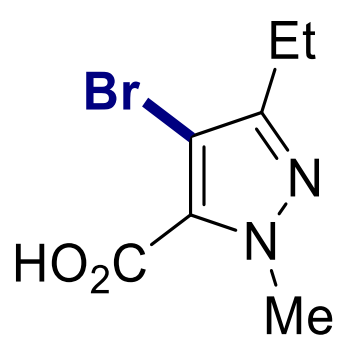

4-bromo-3-ethyl-1-methyl-1H-pyrazole-5-carboxylic acid (63)

^{1}H-NMR: DMSO-d$_6$

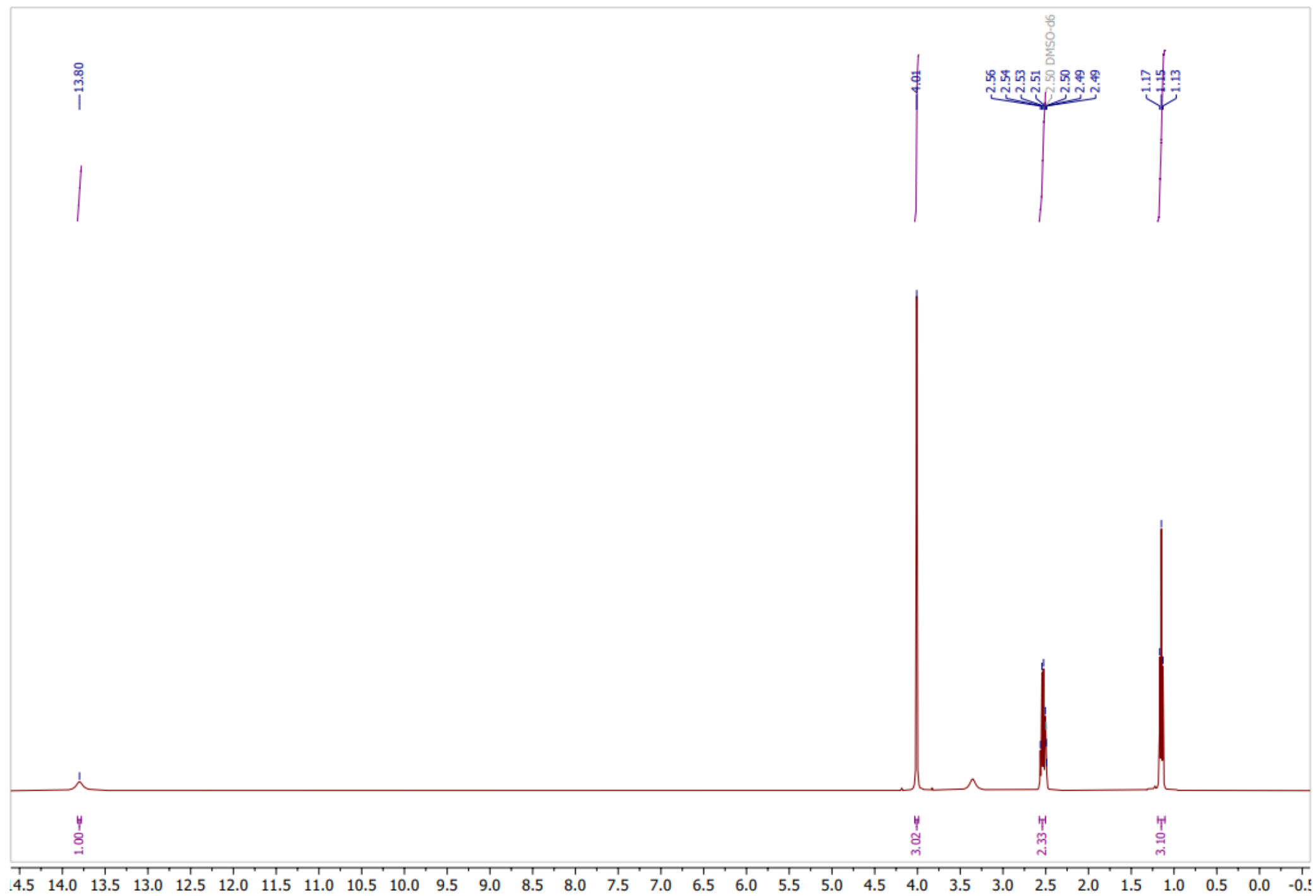

^{13}C-NMR: DMSO-d$_6$

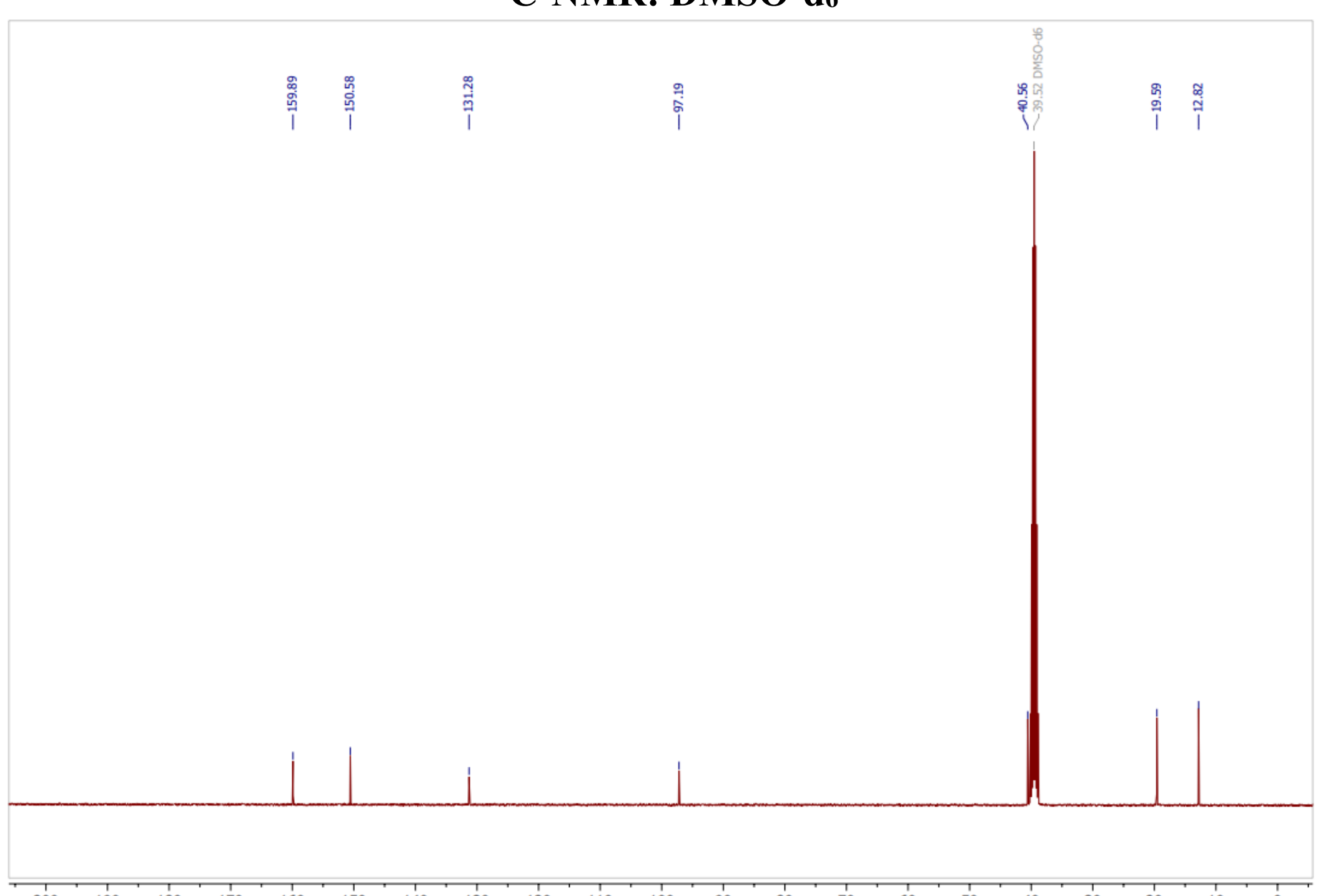

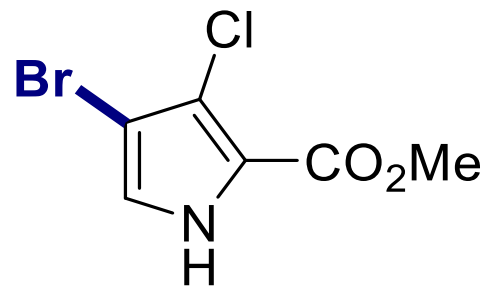

methyl 4-bromo-3-chloro-1H-pyrrole-2-carboxylate (64)

^{1}H-NMR: $CDCl_3$

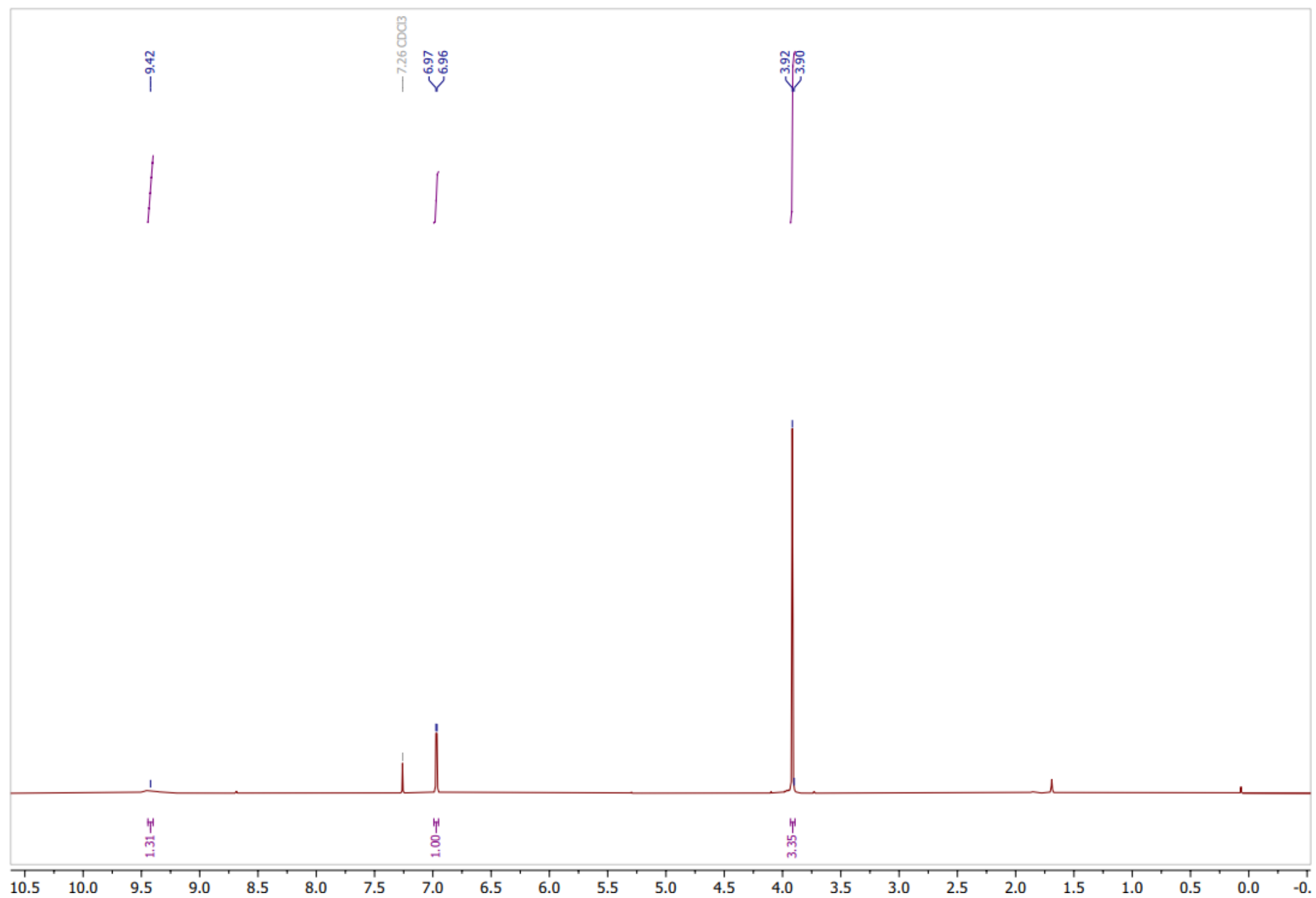

^{13}C-NMR: $CDCl_3$

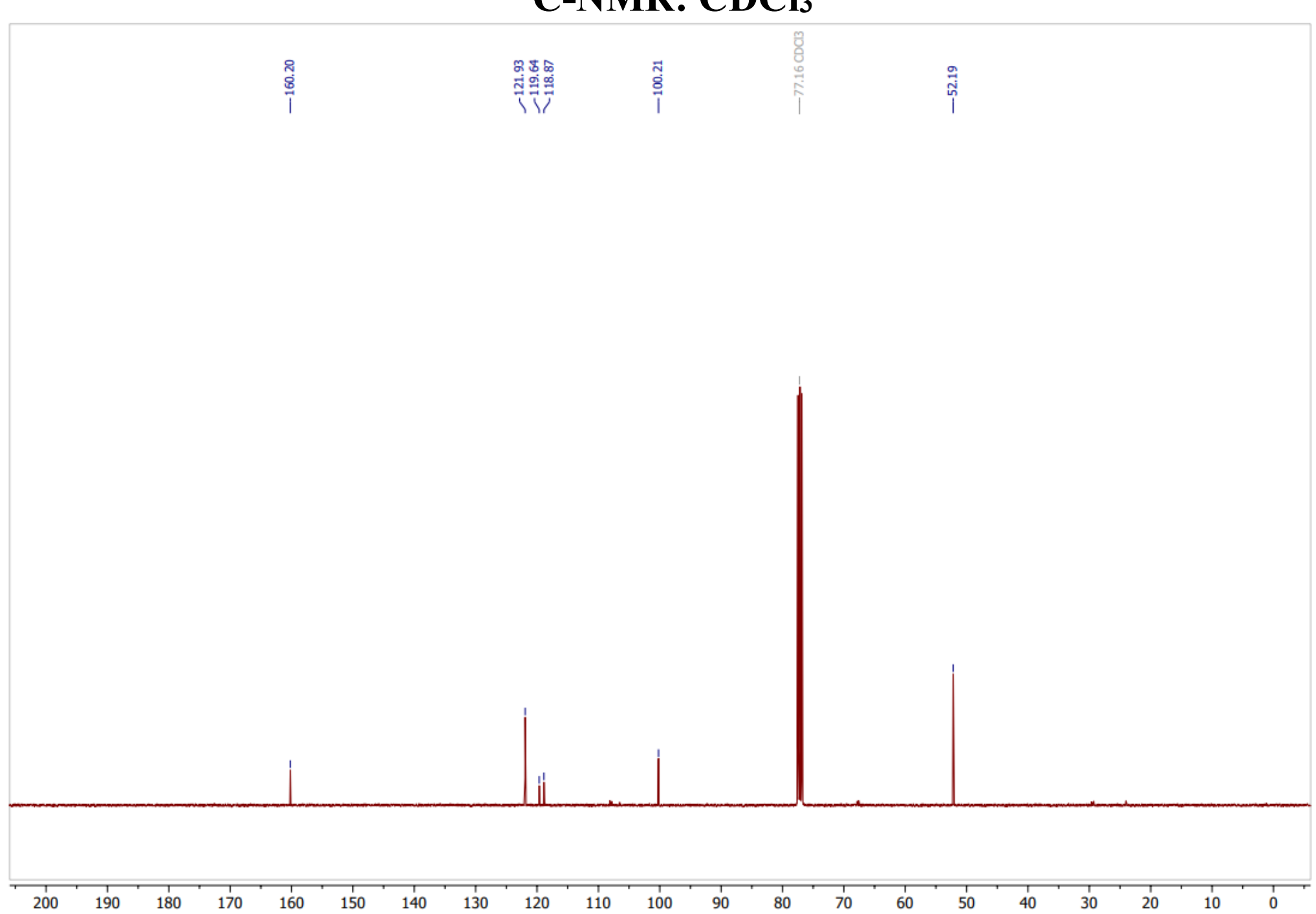

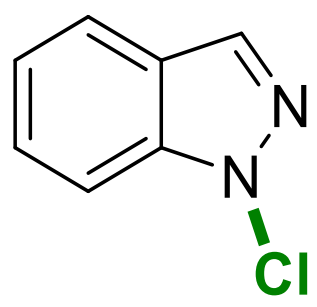

1-chloro-1H-indazole (67)

^{1}H-NMR: DMSO-d$_6$

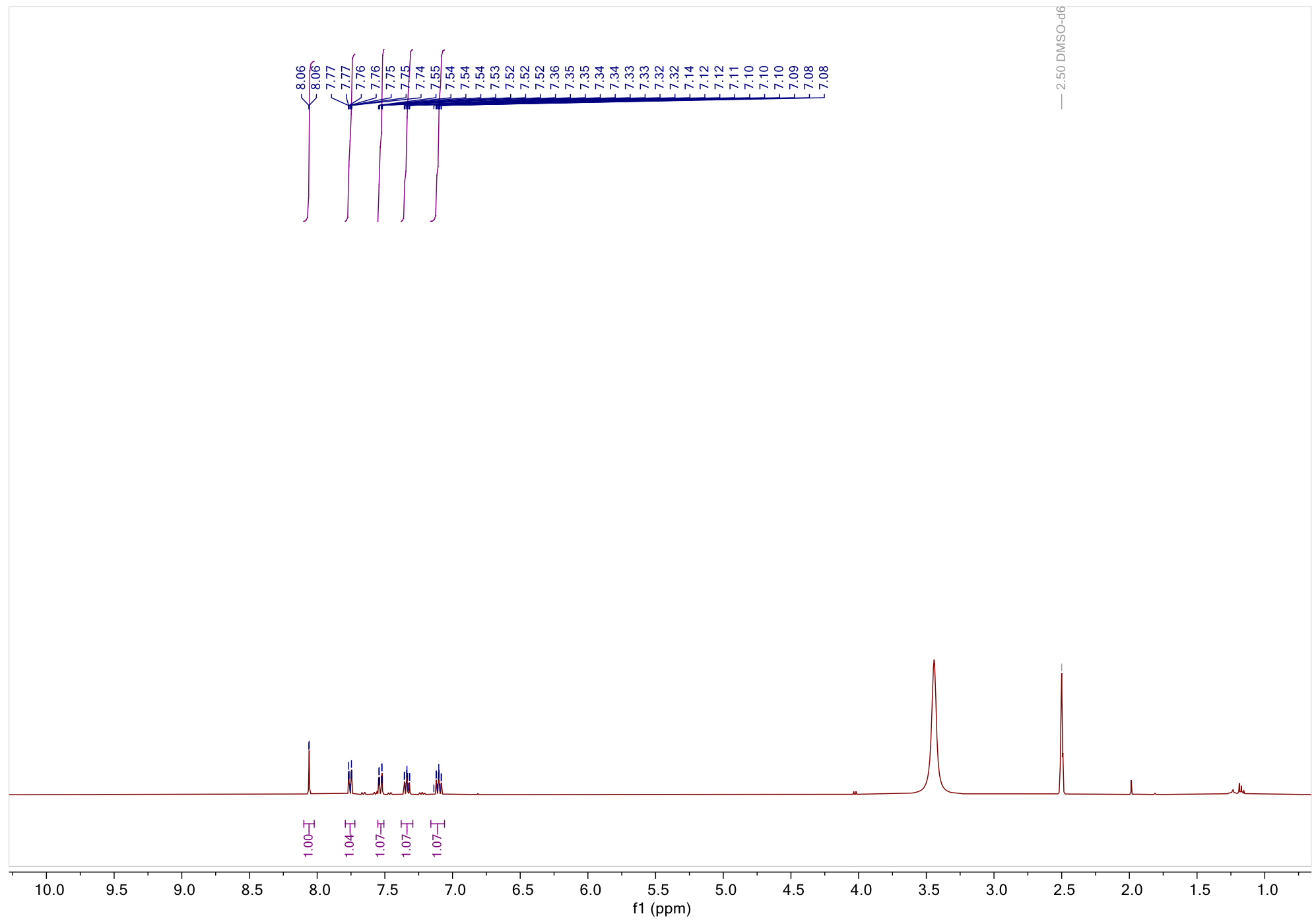

^{13}C-NMR: DMSO-d$_6$

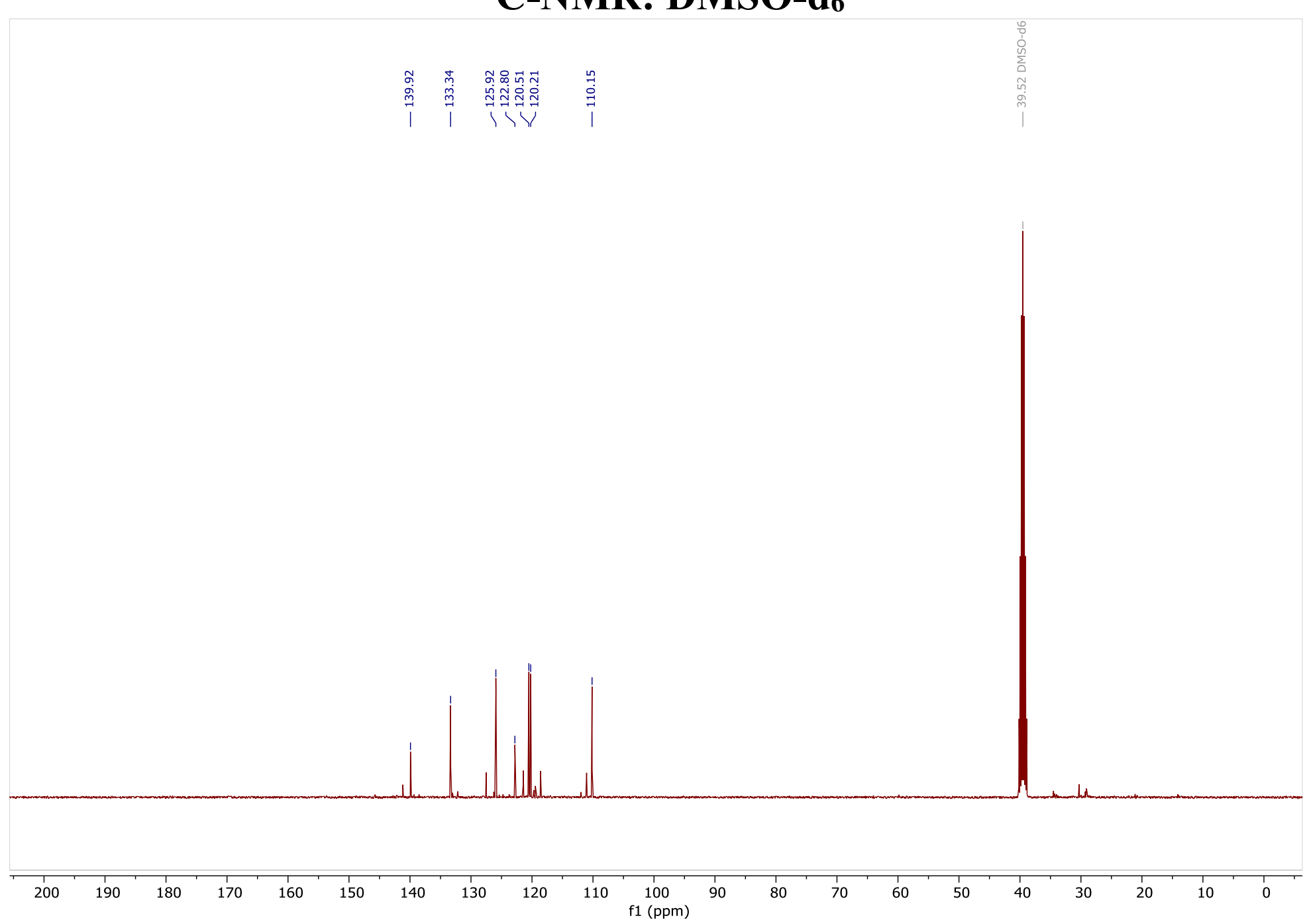

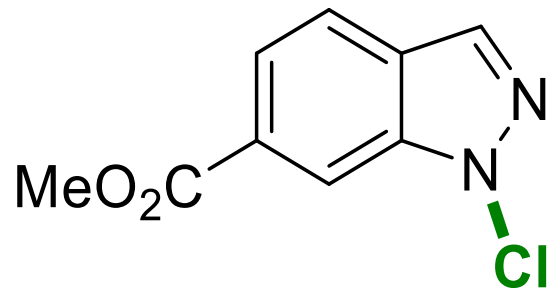

Methyl 1-chloro-1H-indazole-6-carboxylate (68)

^{1}H-NMR: $CDCl_3$

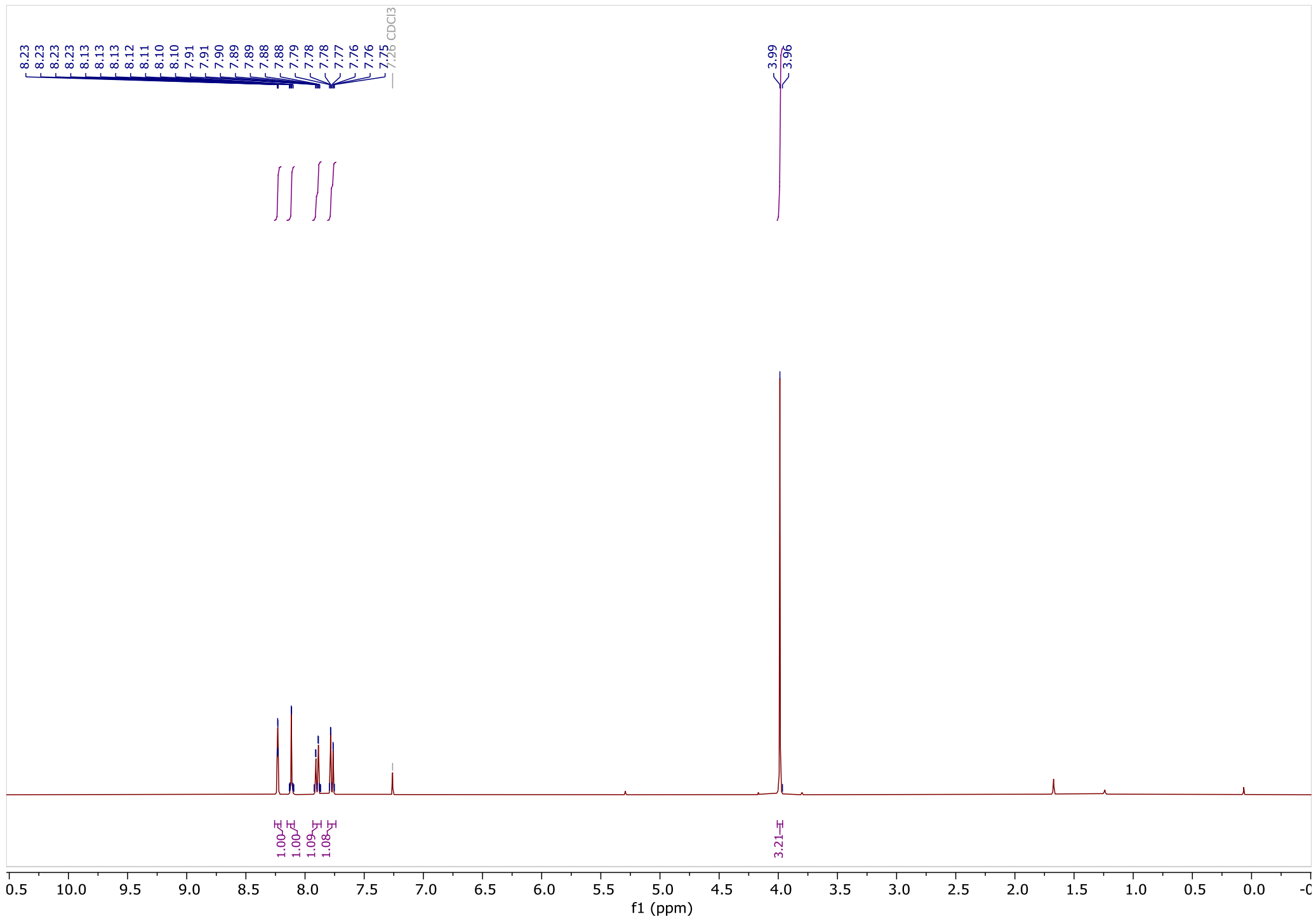

^{13}C-NMR: $CDCl_3$

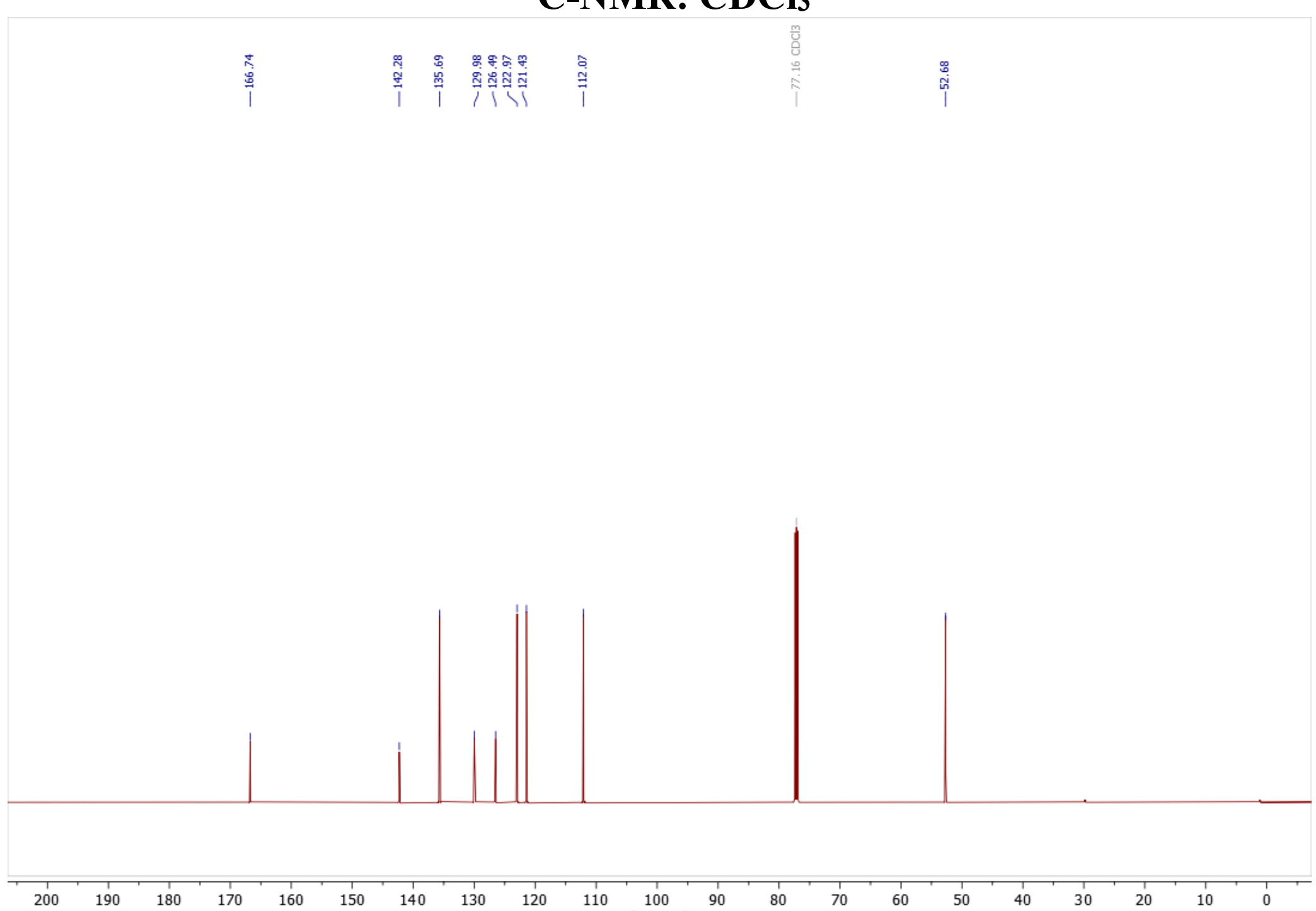

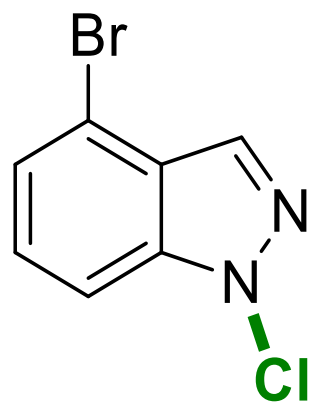

4-bromo-1-chloro-1H-indazole (69)

^{1}H-NMR: $CDCl_3$

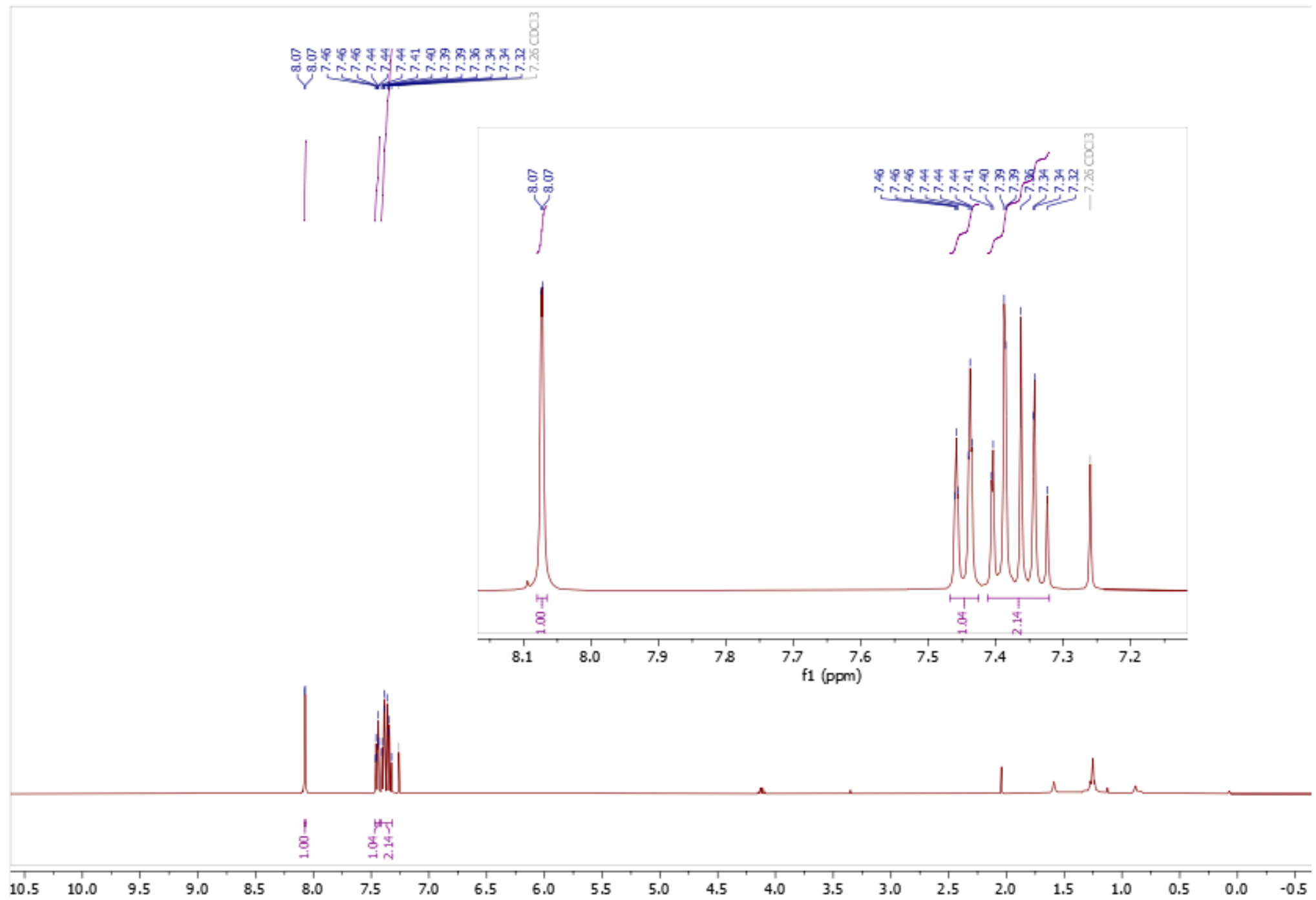

^{13}C-NMR: $CDCl_3$

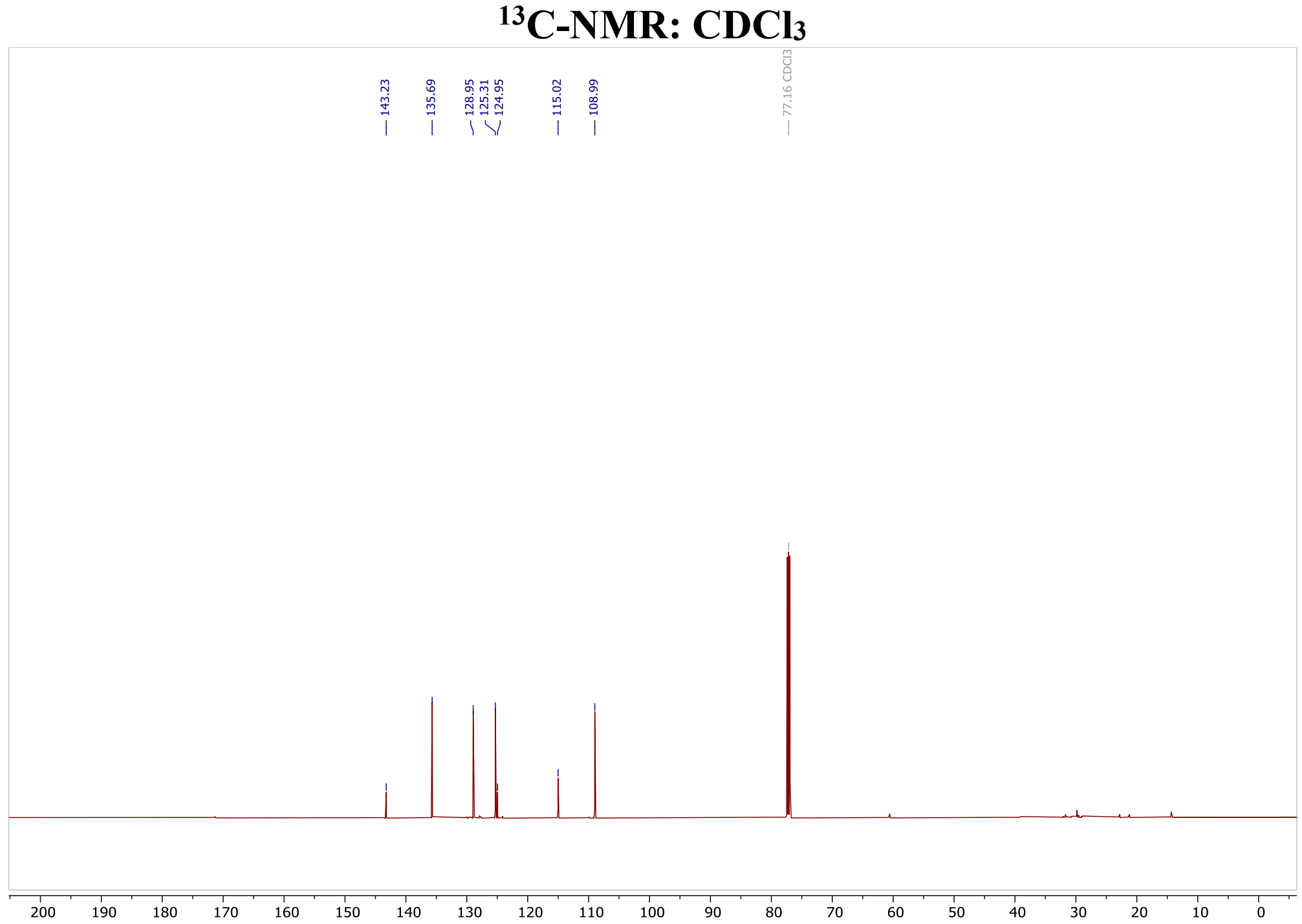

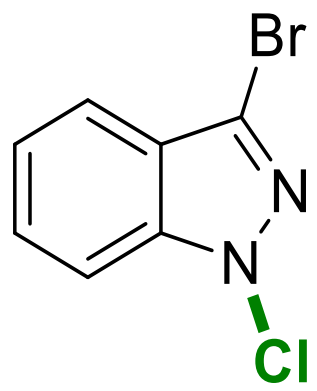

3-bromo-1-chloro-1H-indazole (70)

^{1}H-NMR: CDCl$_3$

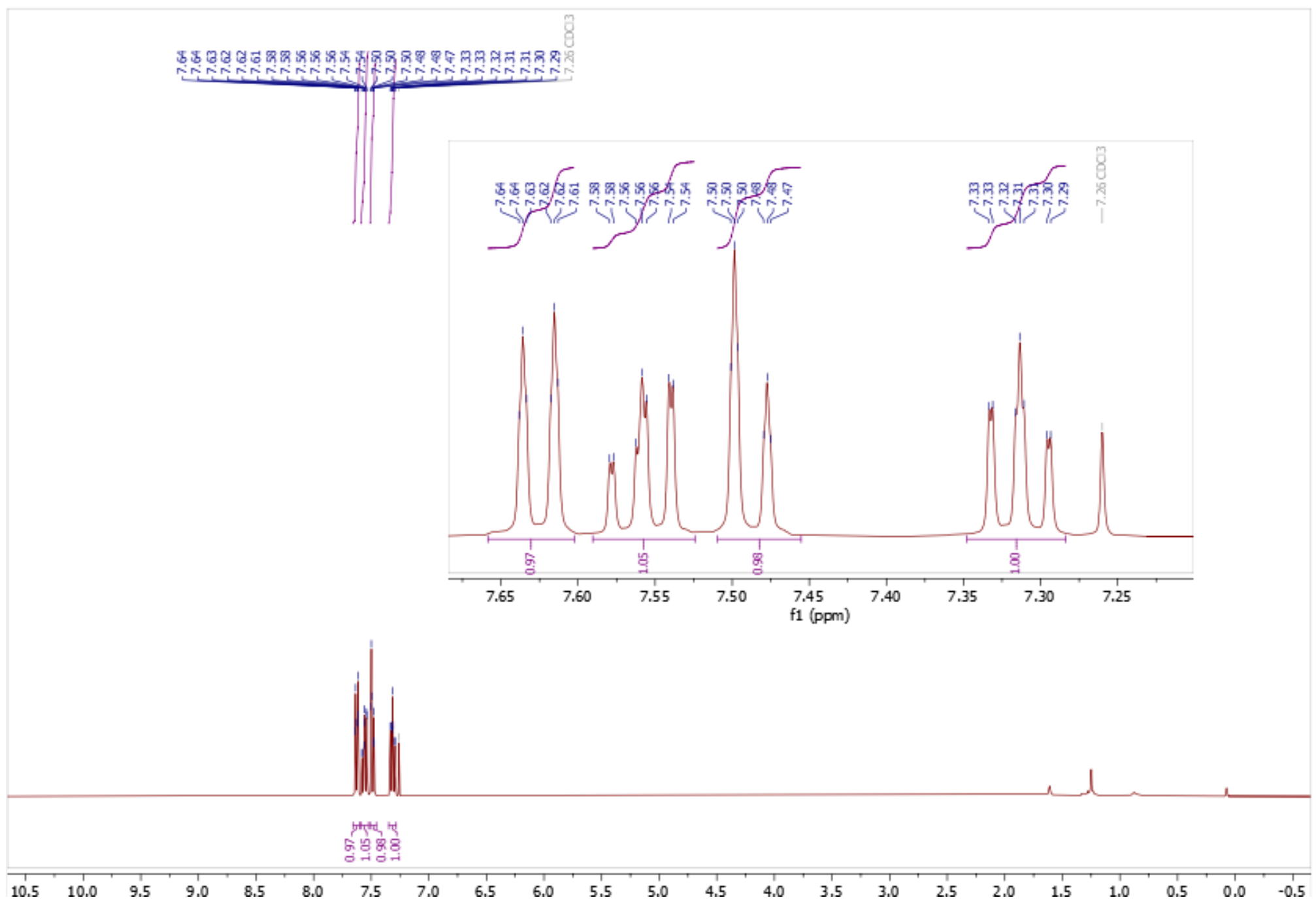

^{13}C-NMR: CDCl$_3$

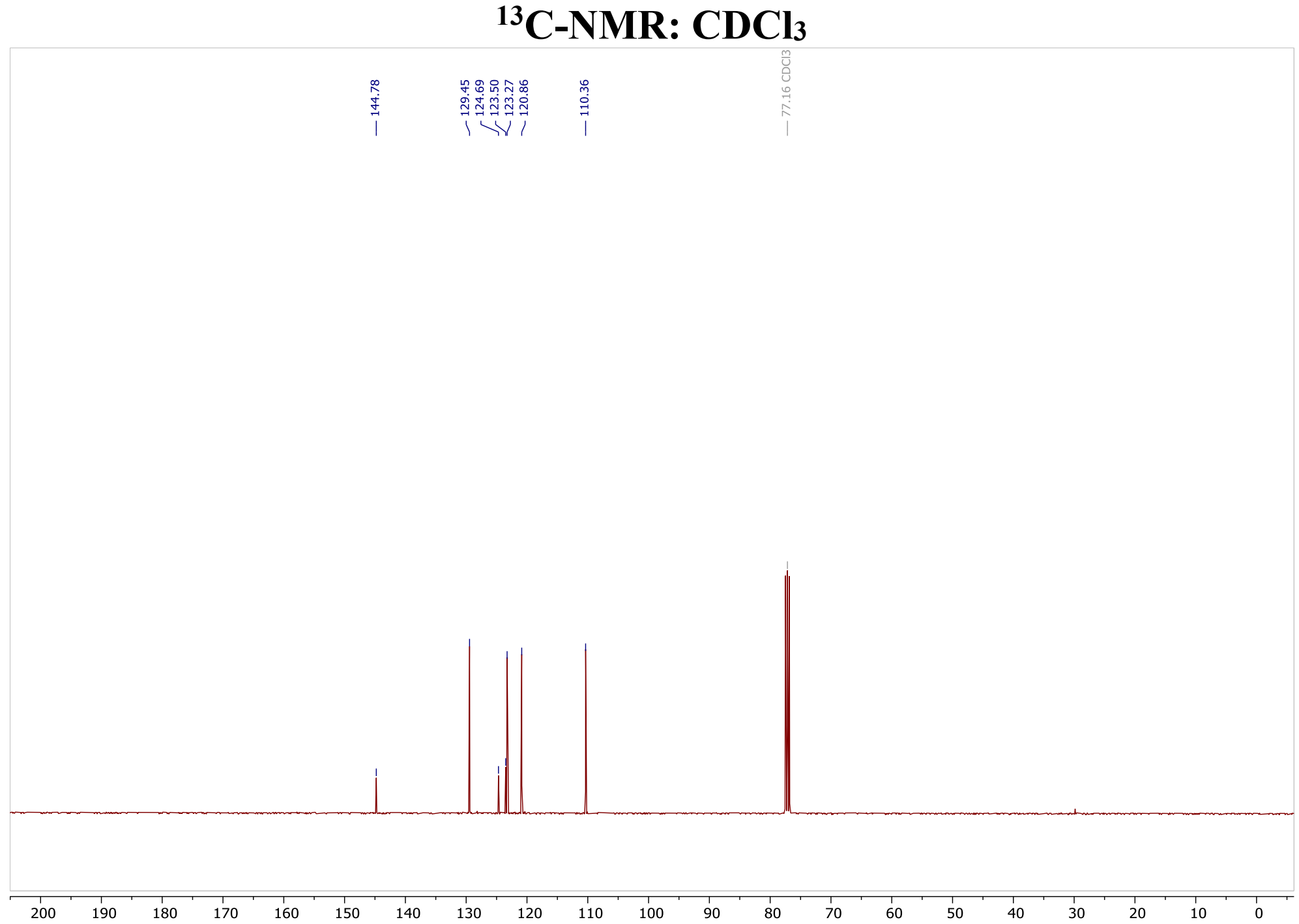

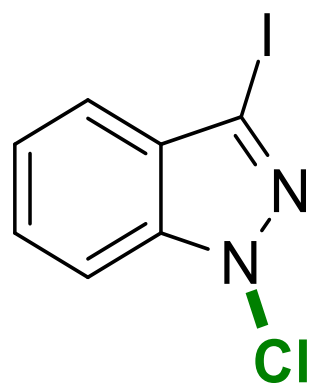

1-chloro-3-iodo-1H-indazole (71)

^{1}H-NMR: $CDCl_3$

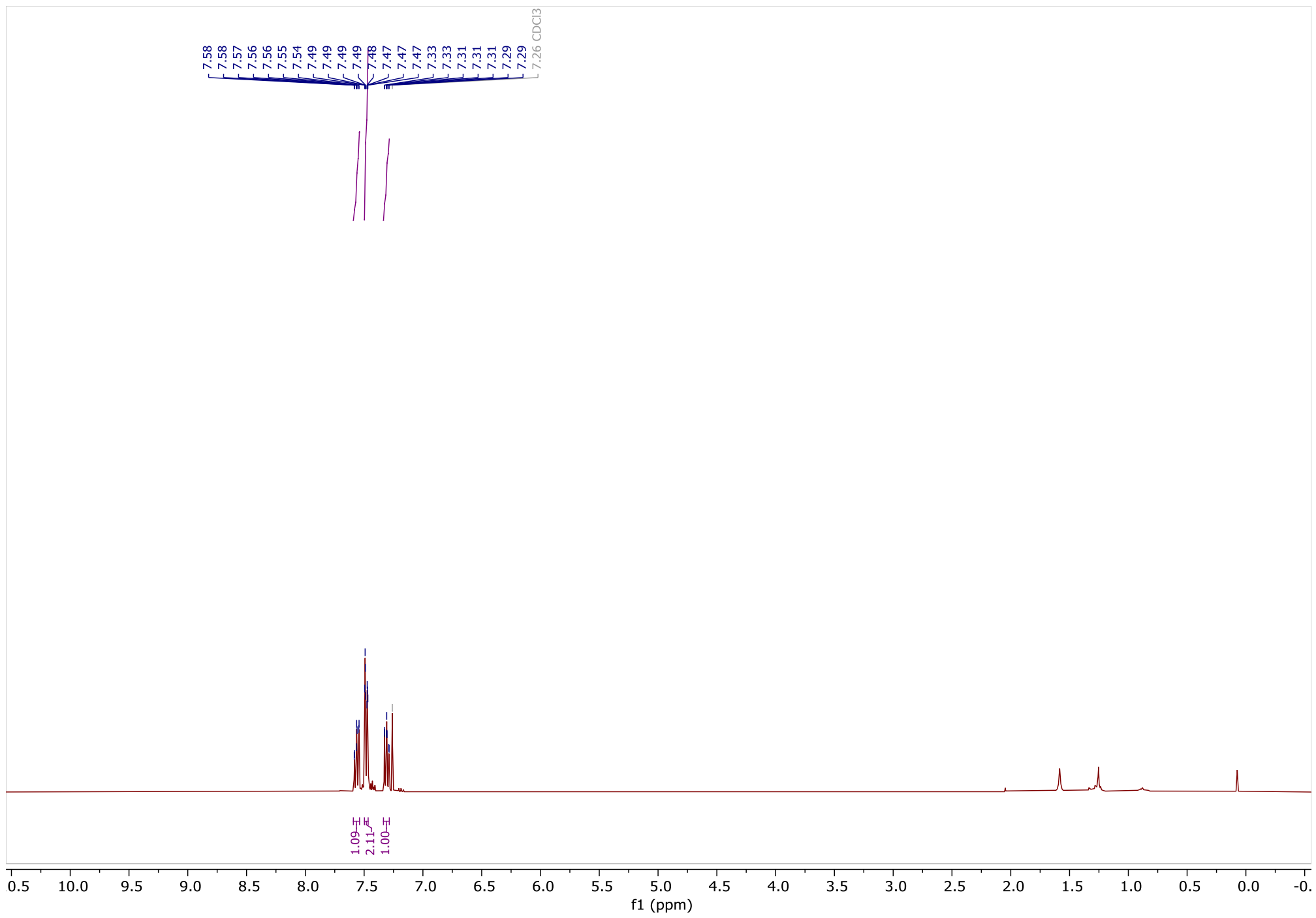

^{13}C-NMR: $CDCl_3$

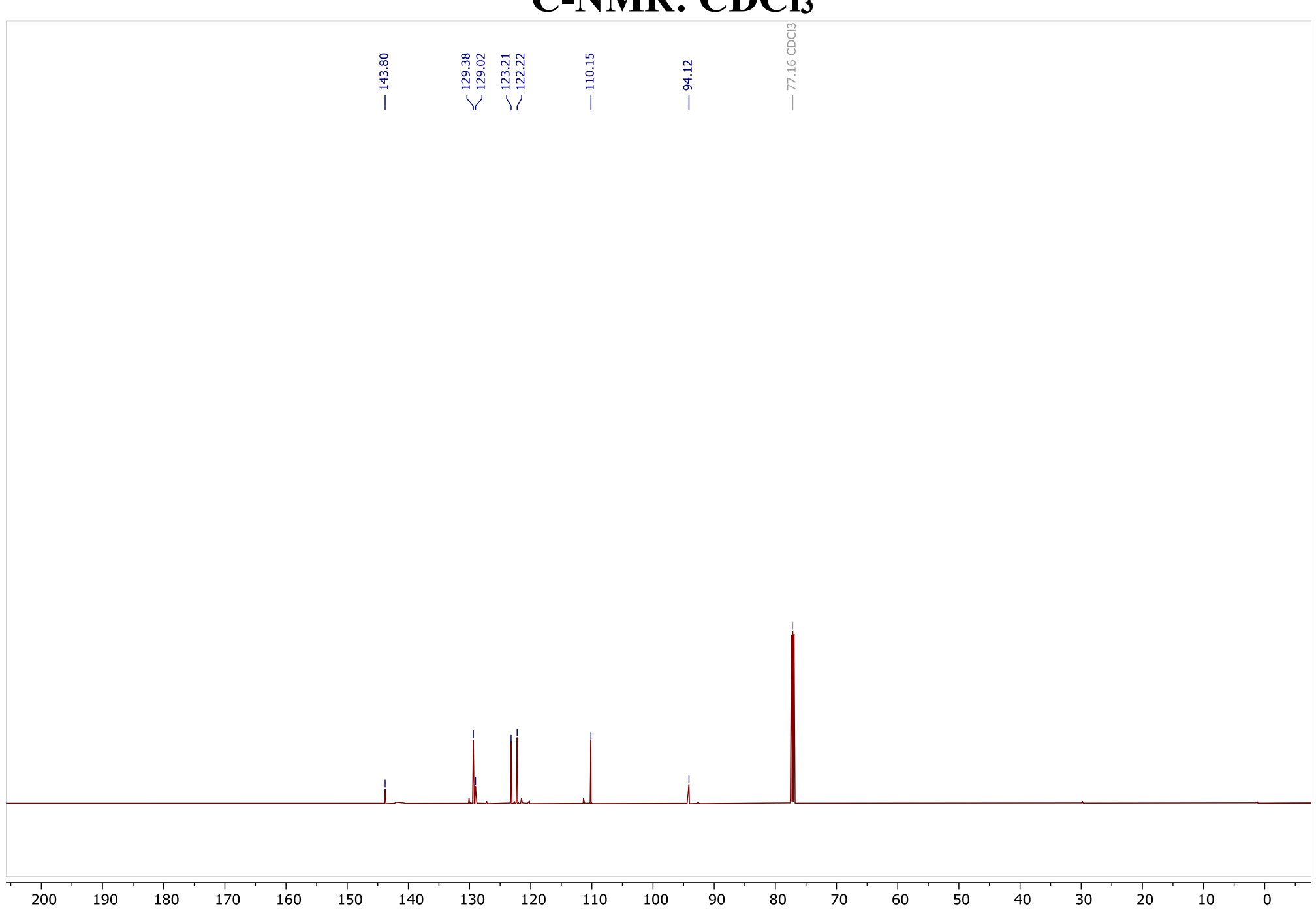

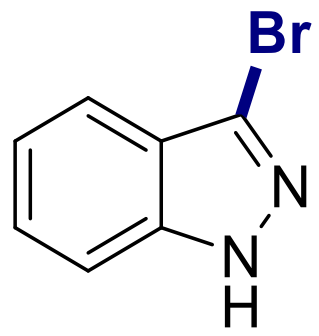

3-bromo-1H-indazole (72)

^{1}H-NMR: DMSO-d$_{6}$

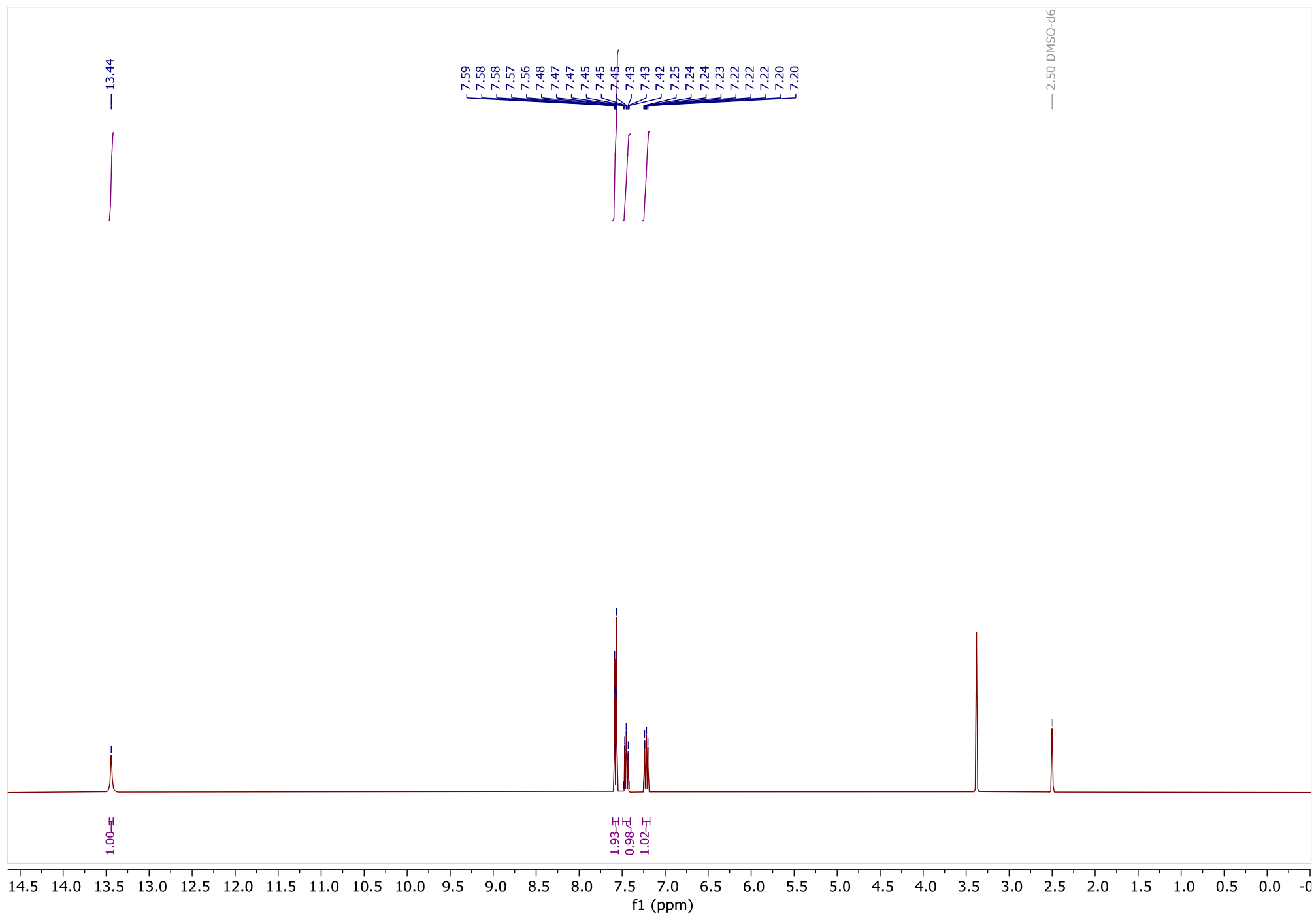

^{1}H-NMR: DMSO-d$_{6}$

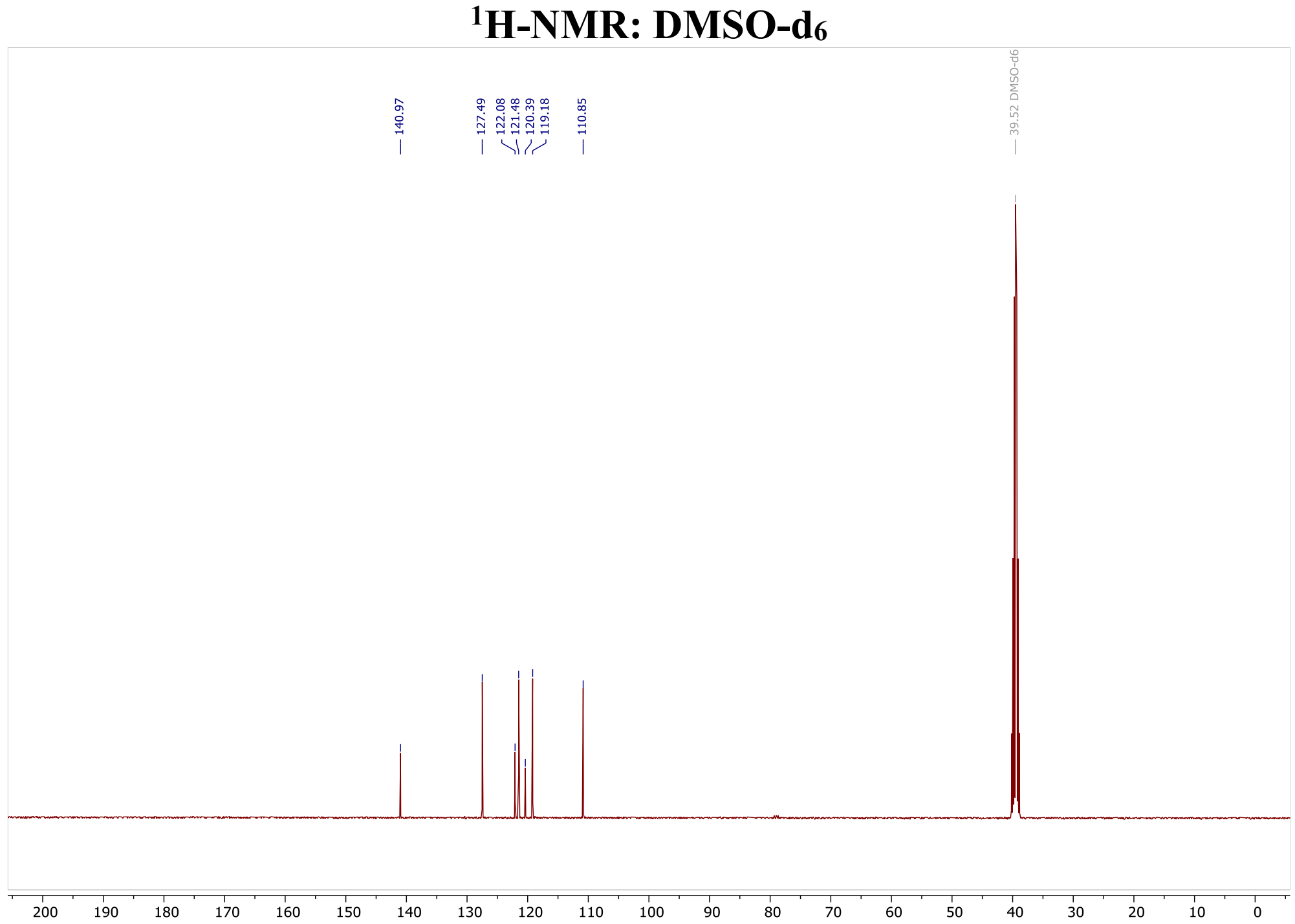

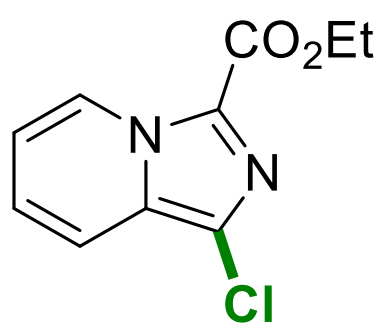

ethyl 1-chloroimidazo[1,5-a]pyridine-3-carboxylate (73)

^{1}H-NMR: $CDCl_3$

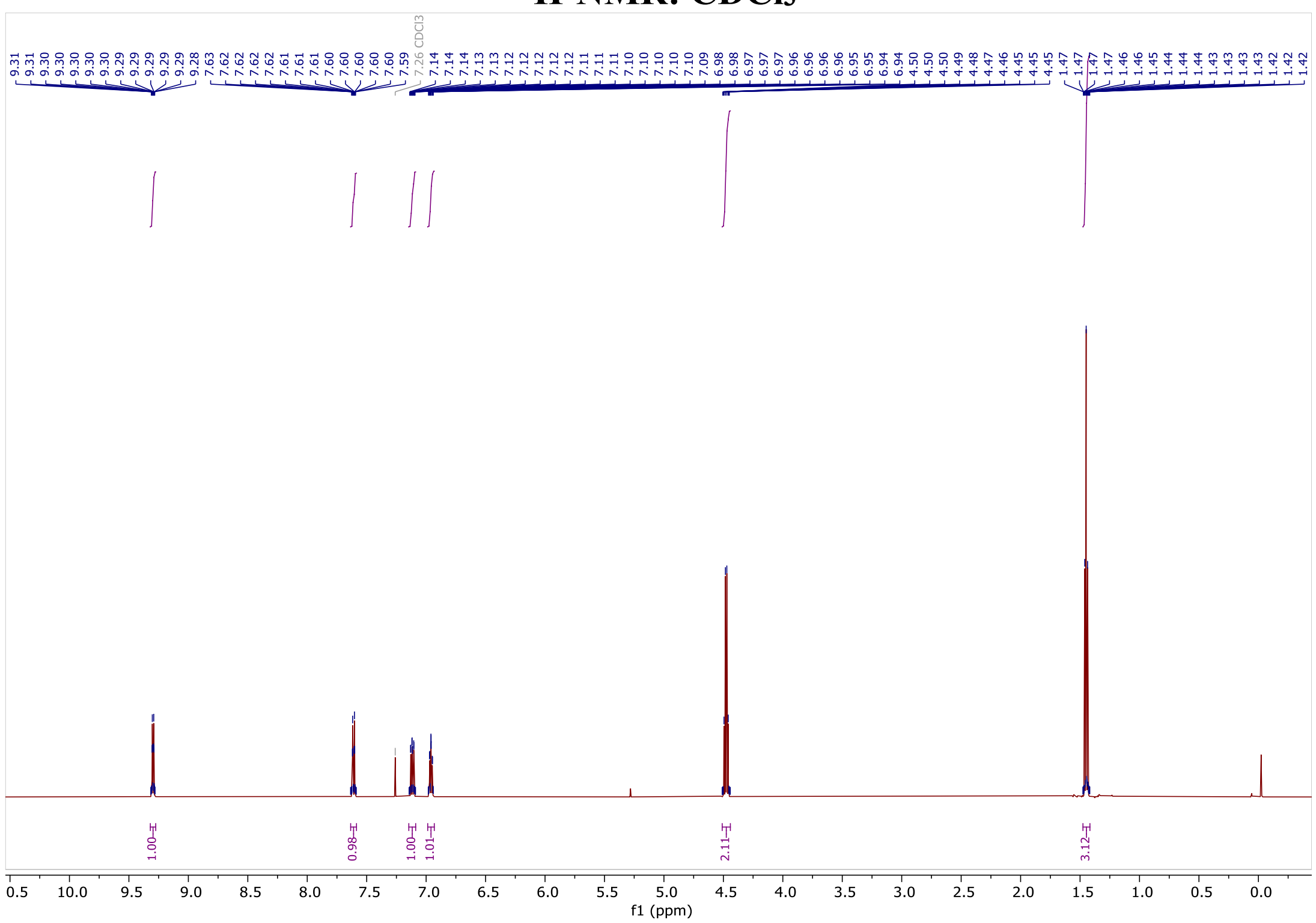

^{13}C-NMR: $CDCl_3$

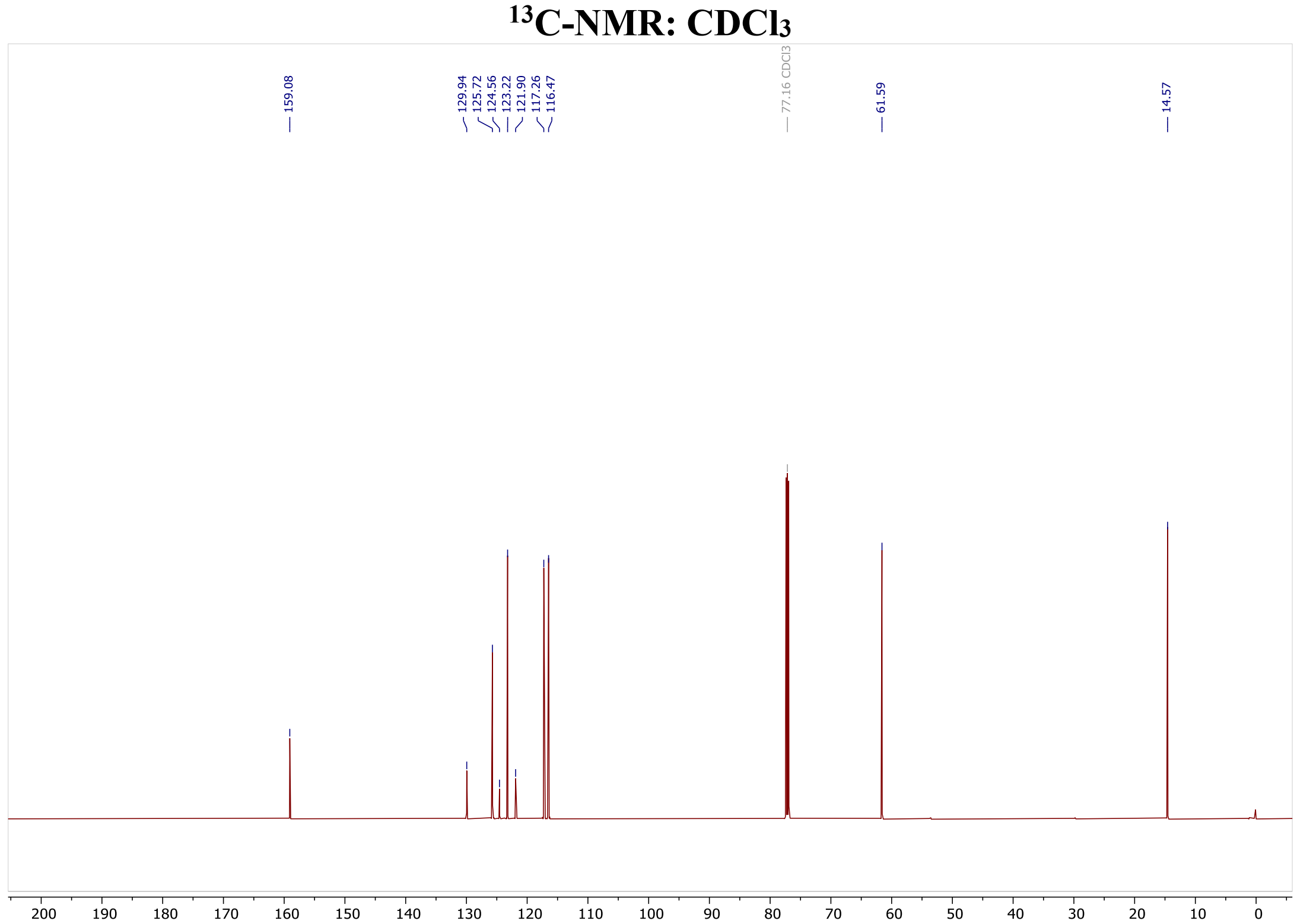

R_1=2.37%

Crystal Data and Experimental

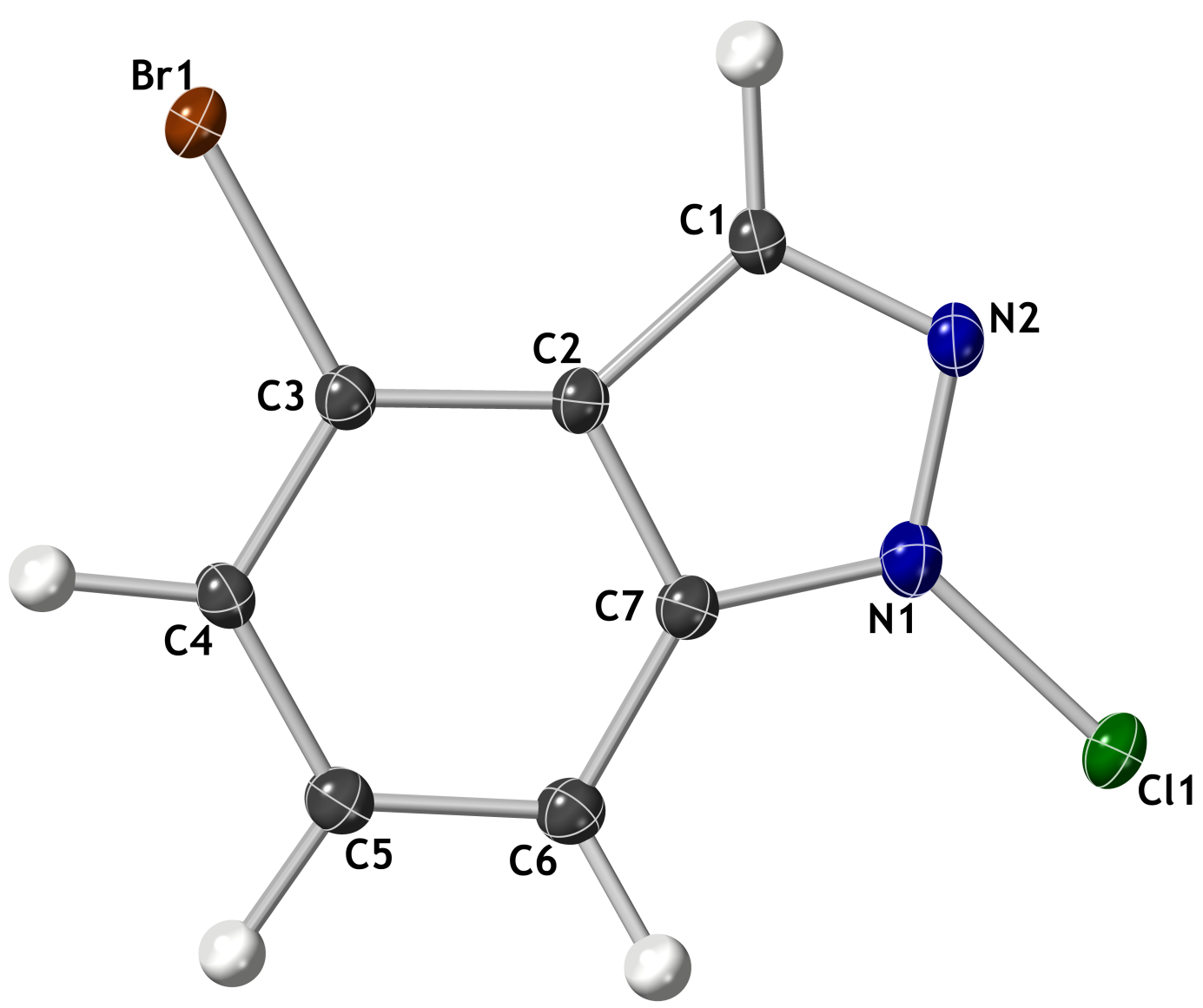

Experimental. Single colorless needle-shaped crystals of **SAS-05-051** were used as supplied. A suitable crystal with dimensions 0.17 × 0.03 × 0.03 mm was selected and mounted on a nylon loop with NVH oil on a XtaLAB Synergy, Dualflex, HyPix diffractometer. The crystal was kept at a constant T = 100.0(1) K during data collection. The structure was solved with the ShelXT (Sheldrick, 2015) and Olex2 1.5-dev (Dolomanov et al., 2009). The model was refined with olex2.refine 1.5-dev (Bourhis et al., 2015) using full matrix least squares minimisation on $|F|^2$.

Crystal Data. $C_7H_4BrClN_2$, M_r = 231.479, orthorhombic, $Pca2_1$ (No. 29), a = 14.2087(2) Å, b = 3.84538(6) Å, c = 27.8444(3) Å, α = 90.0°, β = 90.0°, γ = 90.0°, V = 1521.37(4) Å^3, T = 100.0(1) K, Z = 8, Z' = 2, μ(Cu K$_\alpha$) = 10.005, 15040 reflections measured, 3087 unique (R_{int} = 0.0409) which were used in all calculations. The final wR_2 was 0.0567 (all data) and R_1 was 0.0237 (I≥2σ(I)).

**Compound**	**SAS-05-051**
Formula	$C_7H_4BrClN_2$
$D_{calc.}$/ g cm^{-3}	2.021
μ/mm^{-1}	10.005
Formula Weight	231.479
Color	colourless
Shape	needle-shaped
Size/mm	0.17×0.03×0.03
T/K	100.01(10)
Crystal System	orthorhombic
Flack Parameter	0.47(2)
Hooft Parameter	-0.006(15)
Space Group	$Pca2_1$
a/Å	14.2087(2)
b/Å	3.84538(6)
c/Å	27.8444(3)
α/°	90.0
β/°	90.0
γ/°	90.0
V/Å^3	1521.37(4)
Z	8
Z'	2
Wavelength/Å	1.54184
Radiation type	Cu K_α
Θ_{min}/°	3.17
Θ_{max}/°	77.10
Index range h	-17 ≤ h ≤ 16
Index range k	-4 ≤ k ≤ 4
Index range l	-34 ≤ l ≤ 34
Measured Refl's.	15040
Indep't Refl's	3087
Refl's I≥2σ(I)	3002
R_{int}	0.0409
Parameters	312
Restraints	247
Largest Peak/eÅ^3	0.8284
Deepest Hole/eÅ^3	-0.3806
GooF	1.0732
R_1 (I≥2σ(I) / all)	0.0237 / 0.0252
wR_2 (I≥2σ(I) / all)	0.0562 / 0.0567

Structure Quality Indicators

**Reflections:**	d min (CuKα) 2Θ=154.2° 0.79	I/σ(I) 34.2	Rint m=4.86 4.09%	CAP 139.9° 98% to 154.2° 99.5
**Refinement:**	Shift -0.001	Max Peak 0.8	Min Peak -0.4	GooF 1.073

A colourless needle-shaped crystal with dimensions 0.17 × 0.03 × 0.03 mm was mounted on a nylon loop with NVH oil. Data were collected using a XtaLAB Synergy, Dualflex, HyPix diffractometer equipped with an Oxford Cryosystems low-temperature device operating at T = 100.01(10) K.

Data were measured using ω scans with Cu K_α radiation. The diffraction pattern was indexed and the total

number of runs and images was based on the strategy calculation from the program CrysAlis^{Pro} system (CCD 44.57a 64-bit (release 20-06-2024)). The maximum resolution achieved was Θ = 77.10° (0.79 Å).

The unit cell was refined using CrysAlis^{Pro} on 10769 reflections, 72% of the observed reflections.

Data reduction, scaling and absorption corrections were performed using CrysAlis^{Pro}. The final completeness is 100.00 % out to 77.10° in Θ. An analytical absorption correction using a multifaceted crystal model based on expressions derived by R.C. Clark & J.S. Reid. (Clark, R. C. & Reid, J. S. (1995). Acta Cryst. A51, 887-897) was performed using CrysAlis^{Pro} 1.171.44.57a (Rigaku Oxford Diffraction, 2024). An empirical absorption correction using spherical harmonics, implemented in SCALE3 ABSPACK scaling algorithm was also applied. The absorption coefficient μ of this material is 10.005 mm^{-1} at this wavelength (λ = 1.54184Å) and the minimum and maximum transmissions are 0.425 and 0.800.

The structure was solved in the space group $Pca2_1$ (# 29) by ShelXT (Sheldrick, 2015) using dual methods. It was refined by full matrix least squares minimisation on $|\boldsymbol{F}|^2$ using version of olex2.refine 1.5-dev (Bourhis et al., 2015). All atoms were refined anisotropically.

All hydrogen atoms were freely refined. All hydrogen atoms were located in difference Fourier maps and freely refined, including anisotropic displacement parameters, using Hirshfeld atom refinement (HAR). Refinement employed NoSpherA2, an implementation of non-spherical atomic form factors (F. Kleemiss, O. V. Dolomanov, M. Bodensteiner, H. Puschmann, S. Grabowsky et al., Chem. Sci., 2021, 12, 1675, https://doi.org/10.1039/D0SC05526C). The NoSpherA2 implementation of HAR makes use of tailor-made aspherical atomic form factors calculated from a Hirshfeld-partitioned electron density (ED) rather than from spherical-atom form factors. The ED was calculated from a Gaussian basis set single-determinant SCF wavefunction obtained by DFT using selected functionals for a fragment of the crystal. This fragment was embedded in an electrostatic crystal field by employing cluster charges. SOFTWARE: X:-Kyle_xt2-051-an.tscb PARTITIONING: NoSpherA2 INT ACCURACY: Normal METHOD: B3LYP BASIS SET: def2-SVP CHARGE: 0 MULTIPLICITY: 1 DATE: 2026-04-28_13-47-14

The value of Z' is 2. This means that there are two independent molecules in the asymmetric unit. The moiety formula is C7 H4 Br Cl N2.

The N-Cl bond lengths are 1.678(4) Å (N1-Cl1) and 1.683(4) Å (N1b-Cl1b) for the two independent molecules, consistent with a covalent N-Cl single bond. The chlorine lies essentially in the plane of the indazole ring system (Cl1-N1-N2-C1 torsion -179.2(3)°), as expected for substitution at the sp2 pyrazole nitrogen. Chlorine is bonded to the pyrazole-type N1 and bromine to the carbocyclic ring (indazole position 4), confirming the 4-bromo-1-chloro-1H-indazole connectivity.

The structure is polar but the molecule is achiral, so absolute structure is not a meaningful quantity here. The refined Flack parameter of 0.47(2) shows the crystal is an inversion twin, with both polar orientations present in essentially equal proportion. This is a twinning phenomenon along the polar c axis, not a chirality assignment.

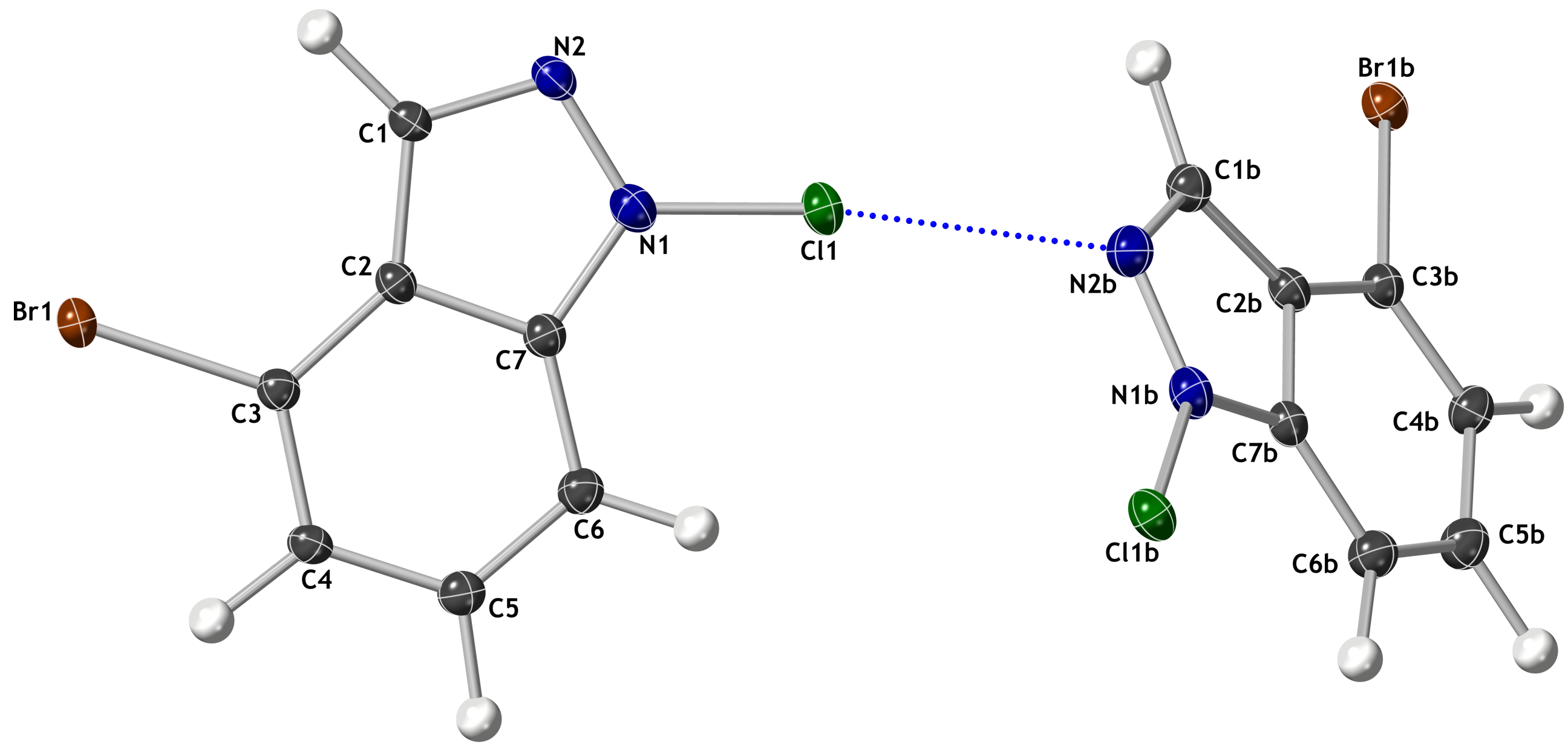

Figure 1 Thermal ellipsoid representation of the asymmetric unit at the 50% probability level, excluding the H atoms, which are shown as spheres. There are two independent molecules in the asymmetric unit.

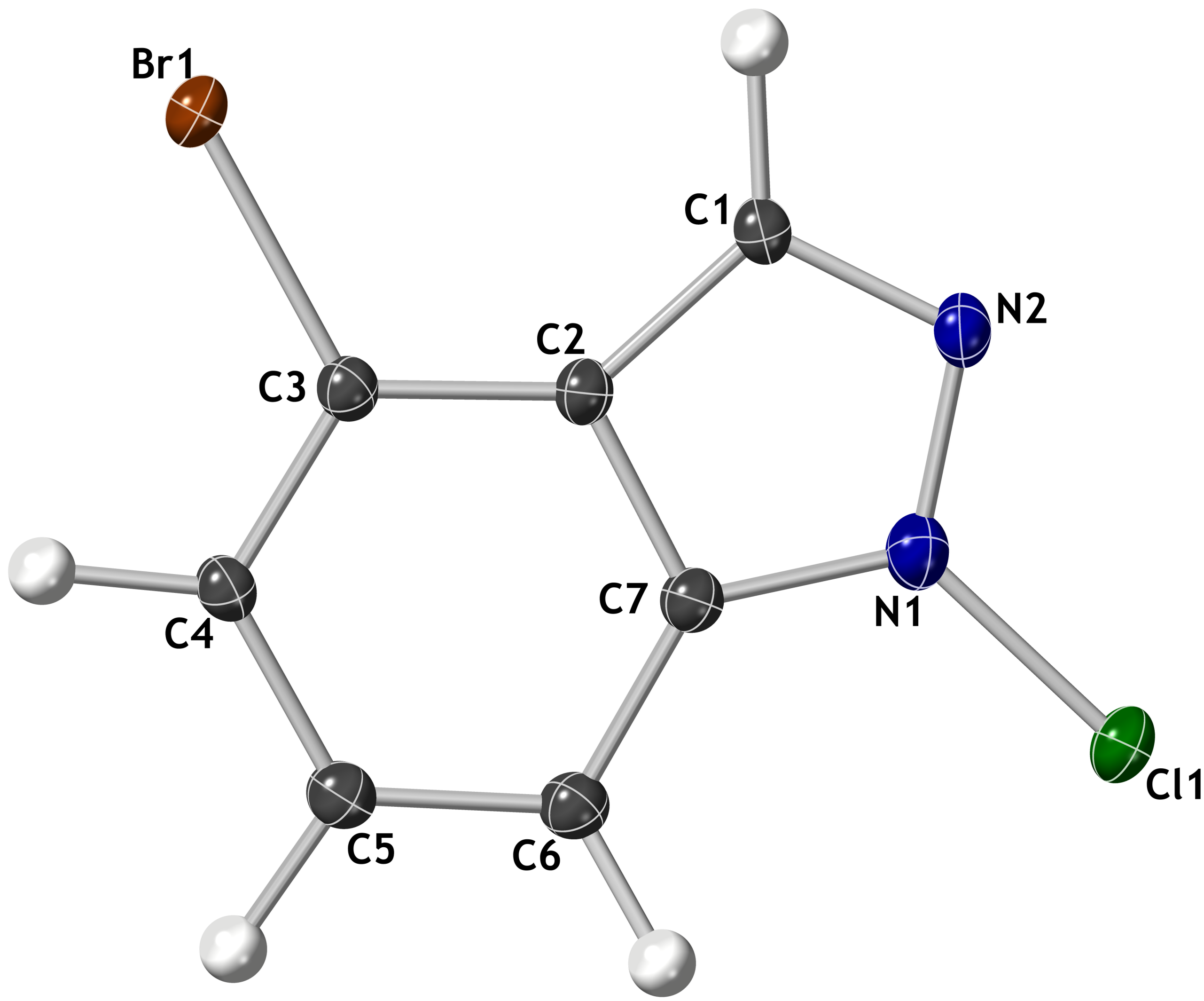

Figure 2 Thermal ellipsoid representation of the molecular structure at the 50% probability level

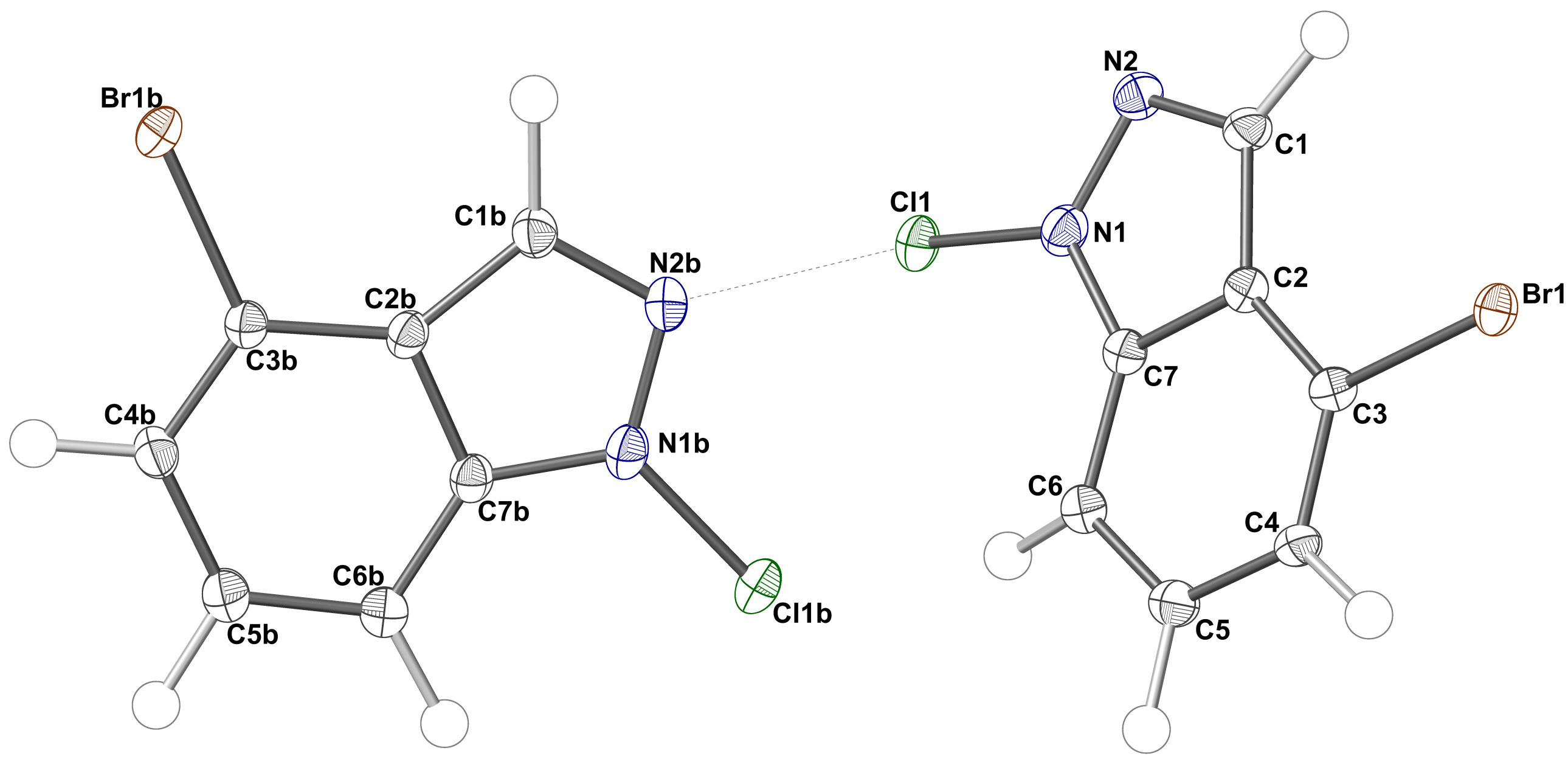

Figure 3 Thermal ellipsoid (2D) representation of the asymmetric unit at the 50% probability level, excluding the H atoms, which are shown as spheres. There are two independent molecules in the asymmetric unit.

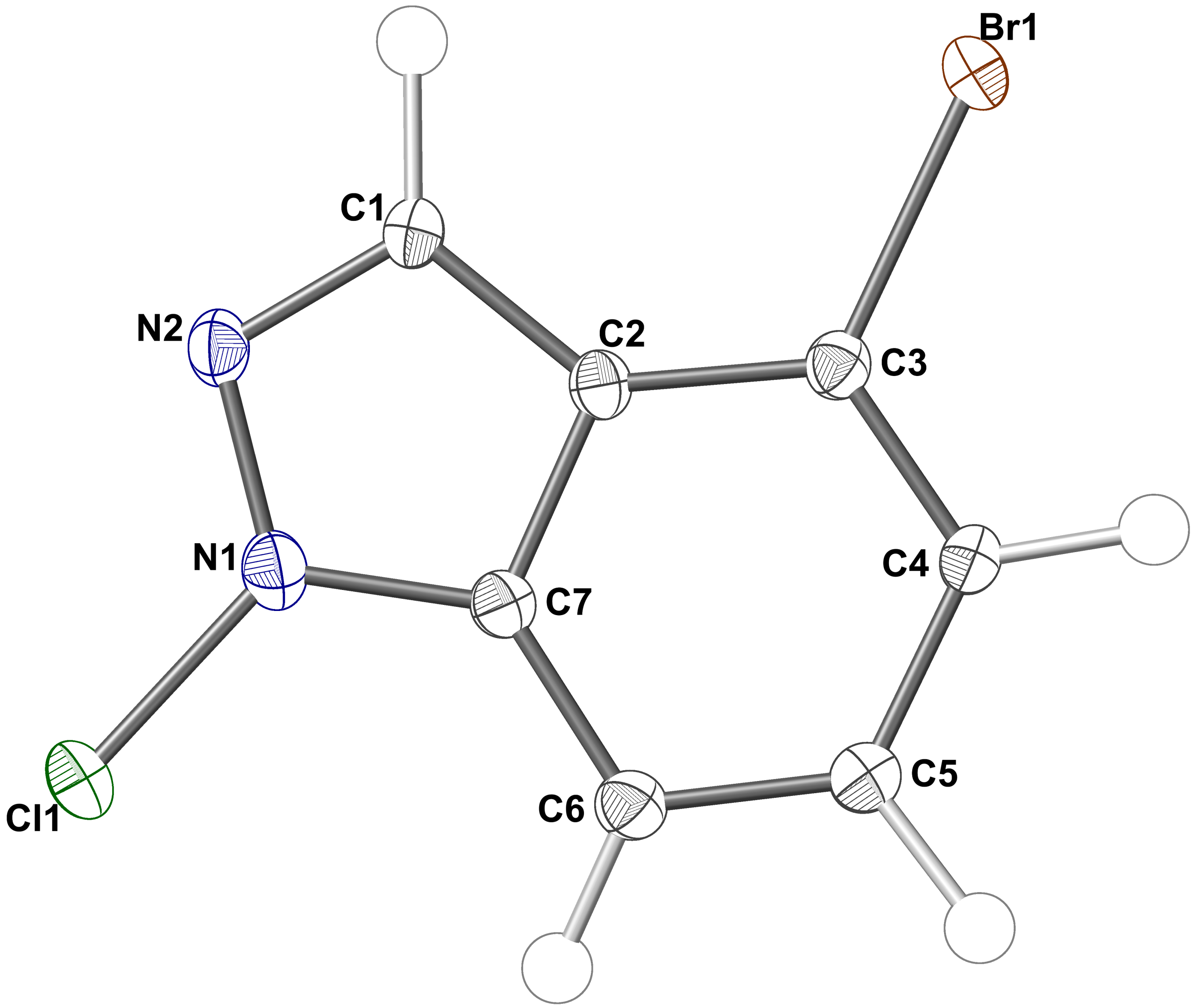

Figure 4 Thermal ellipsoid (2D) representation of the molecular structure at the 50% probability level, excluding the H atoms, which are shown as spheres.

Data Plots: Diffraction Data

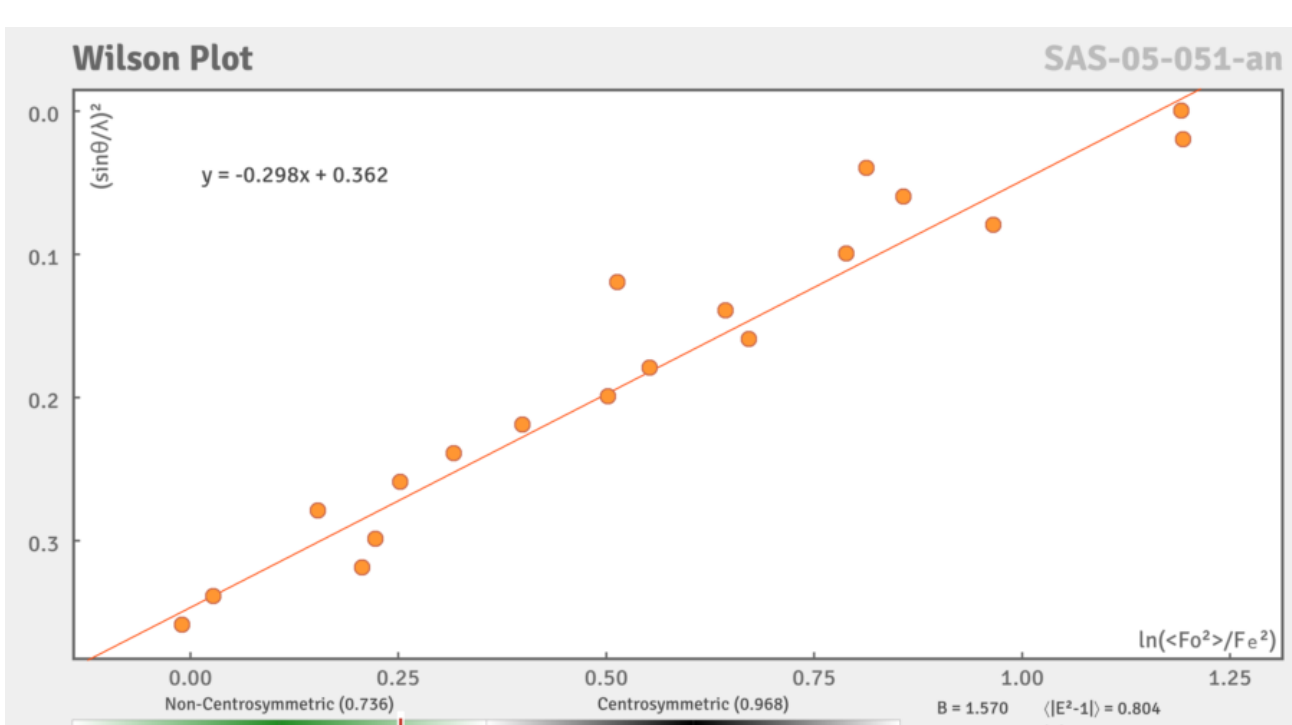

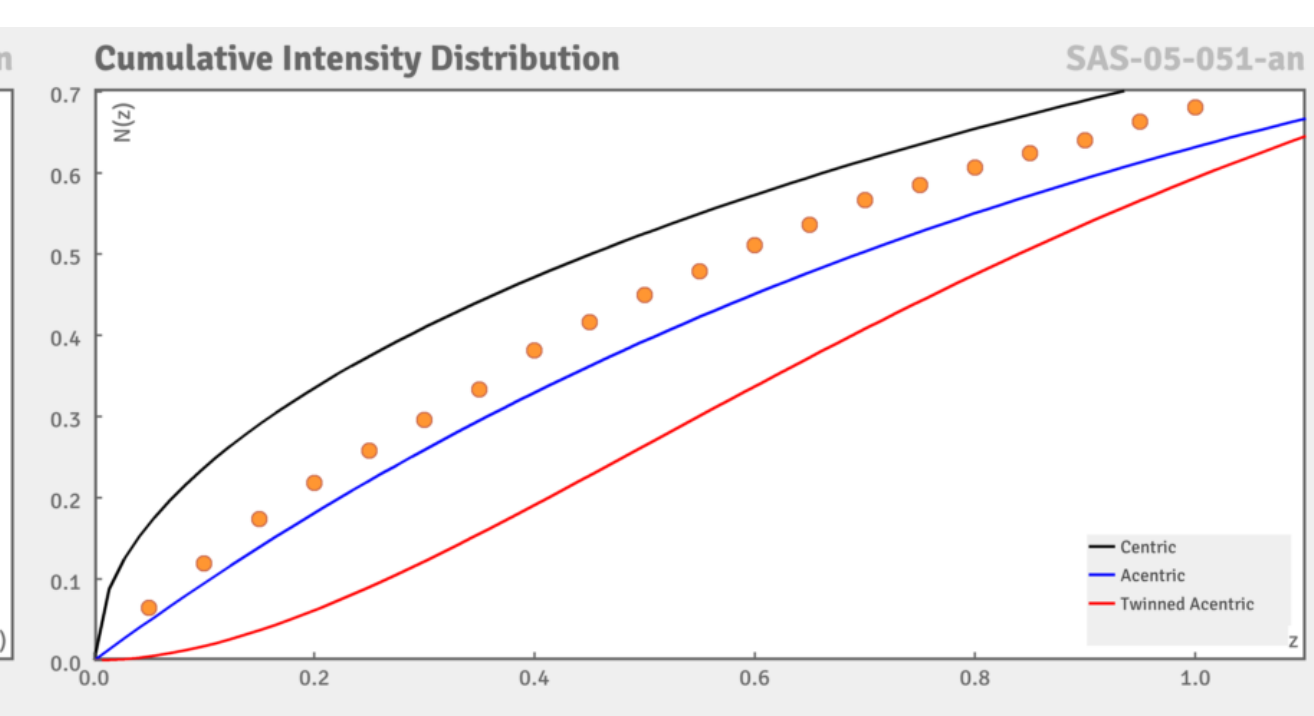

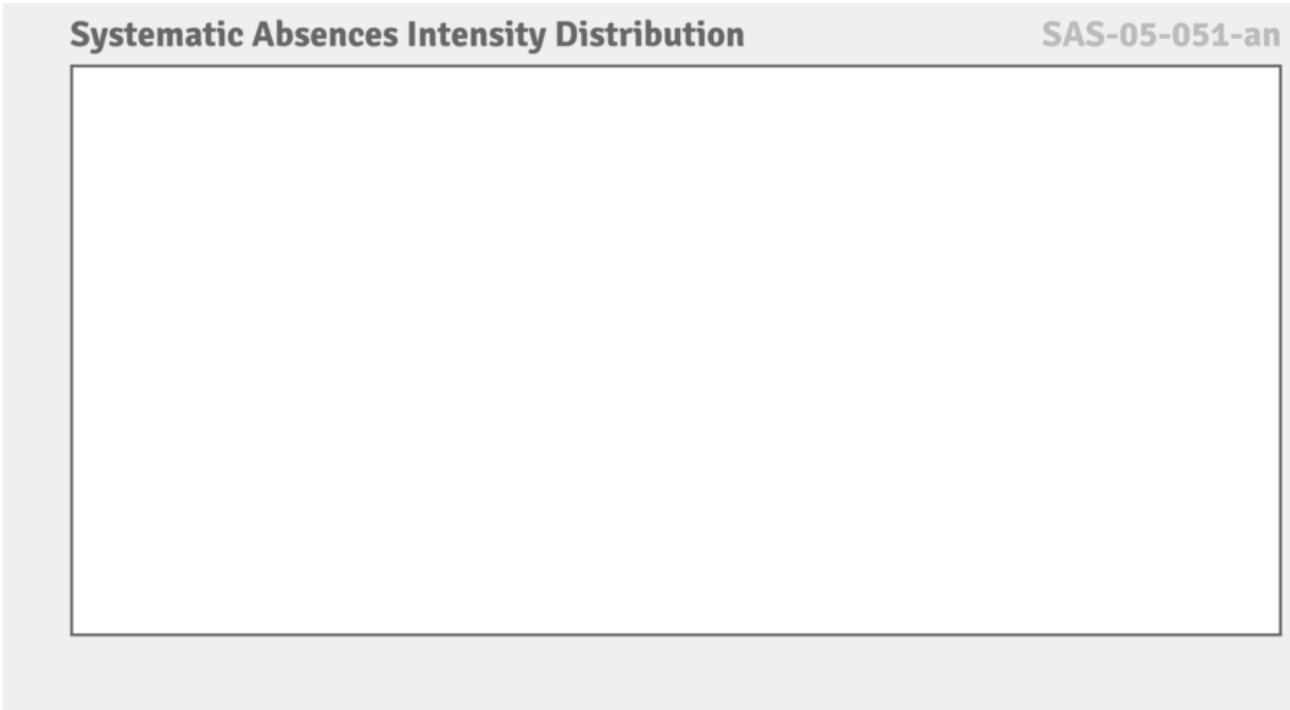

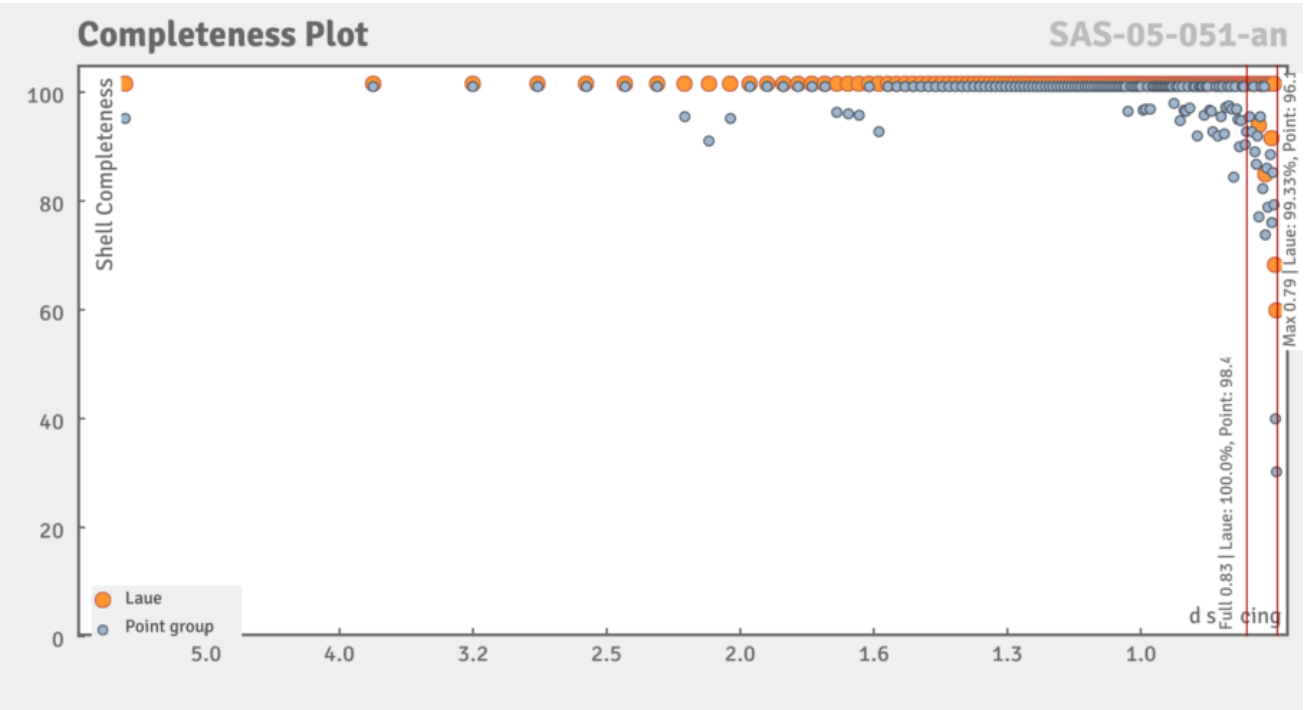

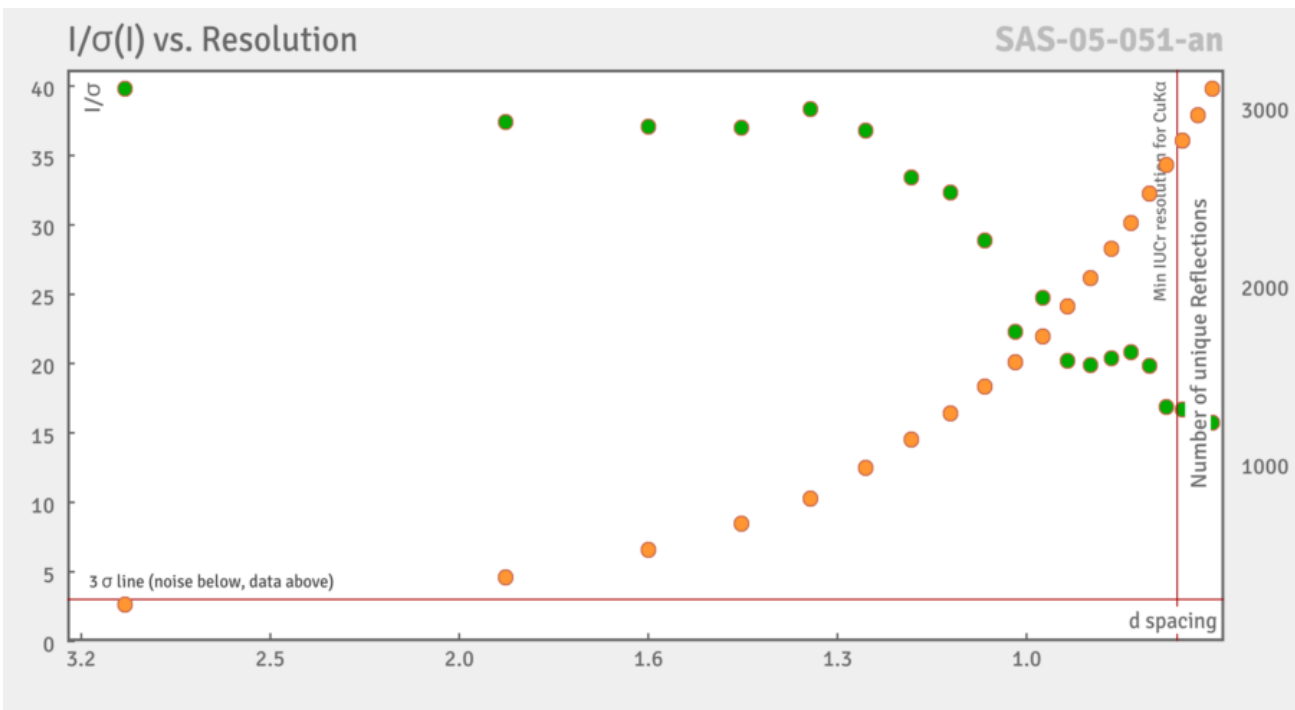

Data Plots: Refinement and Data

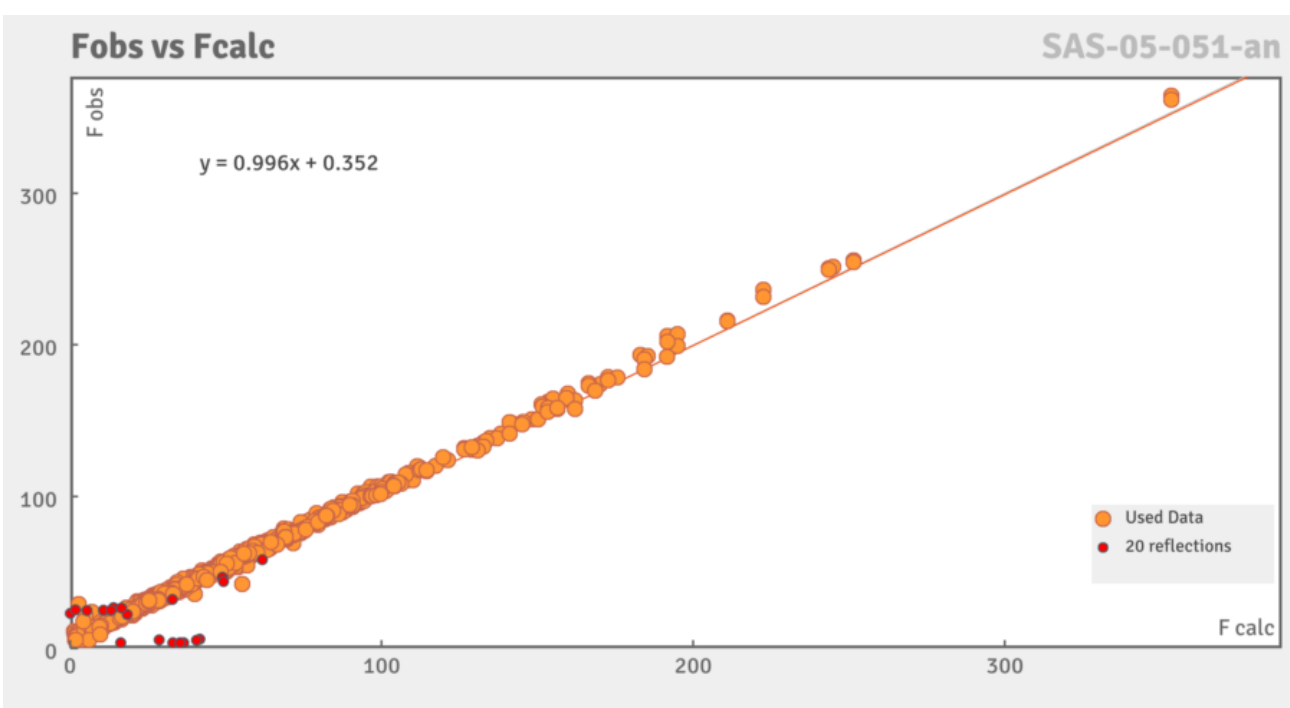

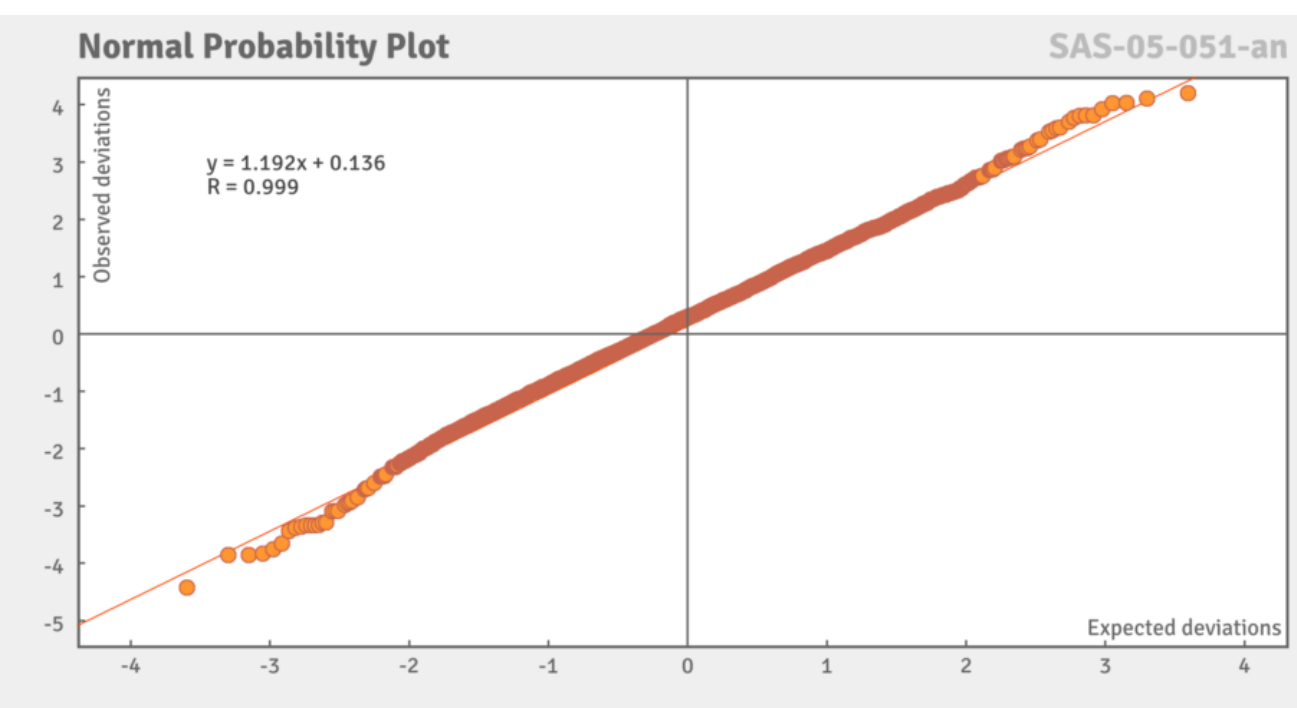

Reflection Statistics

Total reflections (after filtering)	15011	Unique reflections	3087
Completeness	0.961	Mean I/σ	26.45
hkl_{max} collected	(16, 4, 34)	hkl_{min} collected	(-17, -4, -34)
hkl_{max} used	(17, 4, 34)	hkl_{min} used	(0, 0, -34)
Lim d_{max} collected	999.0	Lim d_{min} collected	0.77
d_{max} used	13.92	d_{min} used	0.79
Friedel pairs	2141	Friedel pairs merged	0
Inconsistent equivalents	12	R_{int}	0.0409
R_{sigma}	0.0292	Intensity transformed	0
Omitted reflections	0	Omitted by user (OMIT hkl)	29
Multiplicity	(2987, 1988, 1366, 535, 231, 95, 14, 2)	Maximum multiplicity	17
Removed systematic absences	0	Filtered off (Shel/OMIT)	0

Table 1: Fractional Atomic Coordinates (×10[4]) and Equivalent Isotropic Displacement Parameters ($Å^2$×10[3]) for **SAS-05-051-an**. U_{eq} is defined as 1/3 of the trace of the orthogonalised U_{ij}.

Atom	x	y	z	U_{eq}
Br1	7987.0(6)	-425(2)	2823.1(2)	16.30(9)
Cl1	6598.7(14)	6981(5)	4877.8(6)	18.91(16)
N1	7114(2)	5178(8)	4396.2(15)	18.8(7)
N2	8057(2)	5076(8)	4342.6(13)	17.8(7)
C1	8208(2)	3524(9)	3923.2(13)	17.0(6)
C2	7343(2)	2667(8)	3696.0(12)	14.2(6)
C3	7066(2)	1163(10)	3260.1(13)	15.2(6)
C4	6119(2)	800(10)	3154.7(13)	16.8(6)
C5	5434(2)	1947(10)	3489.1(13)	20.1(7)
C6	5675(2)	3408(10)	3926.5(13)	17.7(7)
C7	6643(2)	3798(9)	4015.8(13)	15.6(6)
Br1b	5340.8(6)	4512(2)	7175.6(2)	17.19(9)
Cl1b	4109.4(14)	11741(5)	5087.0(6)	19.26(17)
N1b	4583(2)	9892(8)	5578.7(15)	19.3(7)
N2b	5521(2)	9475(9)	5633.2(13)	19.7(7)
C1b	5630(2)	8046(10)	6061.3(13)	18.4(7)
C2b	4751(2)	7499(9)	6290.7(12)	15.5(6)
C3b	4436(2)	6180(10)	6733.3(13)	15.4(6)
C4b	3491(2)	6147(10)	6838.9(13)	18.5(7)
C5b	2834(2)	7363(10)	6495.6(13)	20.9(7)
C6b	3104(3)	8649(10)	6052.5(14)	20.0(7)
C7b	4074(2)	8729(9)	5963.6(12)	15.5(6)

Table 2: Anisotropic Displacement Parameters (×10[4]) for **SAS-05-051-an**. The anisotropic displacement factor exponent takes the form: $-2\pi^2[h^2a^{*2} \times U_{11} + ... + 2hka^* \times b^* \times U_{12}]$

Atom	U_{11}	U_{22}	U_{33}	U_{23}	U_{13}	U_{12}
Br1	20.60(15)	14.99(17)	13.30(18)	0.42(12)	1.45(13)	-1.29(19)
Cl1	23.6(4)	20.1(4)	13.0(3)	0.0(3)	2.1(3)	-1.4(3)
N1	21.5(13)	20.5(19)	14.4(15)	-1.8(8)	-0.8(7)	-0.9(9)
N2	19.4(13)	18.8(17)	15.2(15)	-2.6(9)	-1.8(8)	-3.4(10)
C1	15.8(14)	19.9(17)	15.3(13)	-1.6(10)	-1.8(9)	-3.4(10)
H1	16(3)	50(30)	13(12)	1(3)	-3(2)	-8(9)
C2	16.5(13)	12.1(15)	14.1(12)	-0.9(9)	-0.5(8)	-0.9(9)
C3	15.8(14)	13.9(16)	16.0(13)	0.4(9)	-1.4(8)	-0.9(10)
C4	14.9(13)	21.0(17)	14.5(14)	-0.7(10)	-2.3(9)	-1.2(11)
H4	10(9)	40(20)	18(7)	-5(5)	1(3)	-10(6)
C5	16.3(15)	25.5(19)	18.4(14)	-0.3(11)	-0.4(9)	-1.8(11)
H5	16(3)	30(20)	25(12)	0(2)	-1(2)	-7(8)
C6	16.9(14)	19.9(18)	16.4(14)	0.0(10)	1.0(9)	0.8(10)
H6	19(7)	100(30)	49(16)	14(5)	-8(4)	-51(11)
C7	15.9(13)	16.8(17)	14.1(13)	0.1(9)	0.2(8)	0.1(10)
Br1b	22.25(15)	15.50(18)	13.81(18)	0.63(12)	-1.82(13)	1.47(19)
Cl1b	26.1(4)	18.6(4)	13.0(3)	-0.3(3)	-1.8(3)	1.0(3)
N1b	23.3(13)	22(2)	12.5(14)	-0.5(9)	1.9(7)	-0.4(9)
N2b	20.6(13)	24.0(17)	14.6(14)	-1.0(9)	3.2(8)	1.6(10)
C1b	18.5(14)	21.1(18)	15.6(13)	-1.5(10)	1.8(8)	-0.6(10)
H1b	18(4)	20(20)	10(12)	-1(3)	3(3)	-3(7)
C2b	18.6(13)	16.1(16)	11.8(12)	-1.7(9)	-0.3(8)	-0.3(9)
C3b	16.8(14)	15.4(17)	14.0(13)	-0.6(9)	1.0(8)	-0.2(10)
C4b	17.6(14)	20.3(18)	17.6(14)	-0.3(10)	3.6(9)	0.9(11)
H4b	26(10)	70(30)	28(8)	-2(5)	6(3)	23(8)

Atom	U_{11}	U_{22}	U_{33}	U_{23}	U_{13}	U_{12}
C5b	19.9(15)	24.1(18)	18.8(14)	0.1(11)	2.7(9)	1.5(11)
H5b	21(3)	100(50)	45(14)	7(3)	7.3(19)	37(12)
C6b	18.8(14)	23.0(19)	18.1(14)	0.2(10)	0.5(9)	1.8(11)
H6b	20(7)	50(30)	22(8)	4(4)	1(3)	10(7)
C7b	17.9(14)	15.7(17)	12.9(13)	-0.4(9)	0.9(8)	-1.6(10)

Table 3: Bond Lengths in Å for **SAS-05-051-an**.

Atom	Atom	Length/Å
Br1	C3	1.888(4)
Cl1	N1	1.678(4)
N1	N2	1.349(4)
N1	C7	1.361(5)
N2	C1	1.329(5)
C1	C2	1.420(4)
C2	C3	1.401(5)
C2	C7	1.405(5)
C3	C4	1.385(4)
C4	C5	1.417(5)
C5	C6	1.384(5)
C6	C7	1.405(5)

Atom	Atom	Length/Å
Br1b	C3b	1.892(4)
Cl1b	N1b	1.683(4)
N1b	N2b	1.352(4)
N1b	C7b	1.368(5)
N2b	C1b	1.322(5)
C1b	C2b	1.419(4)
C2b	C3b	1.406(5)
C2b	C7b	1.407(4)
C3b	C4b	1.374(5)
C4b	C5b	1.416(5)
C5b	C6b	1.383(5)
C6b	C7b	1.400(5)

Table 4: Bond Angles in ° for **SAS-05-051-an**.

Atom	Atom	Atom	Angle/°
N2	N1	Cl1	122.3(3)
C7	N1	Cl1	124.6(3)
C7	N1	N2	113.1(3)
C1	N2	N1	105.7(3)
C2	C1	N2	110.9(3)
C3	C2	C1	136.4(3)
C7	C2	C1	105.0(3)
C7	C2	C3	118.5(3)
C2	C3	Br1	119.8(2)
C4	C3	Br1	120.3(3)
C4	C3	C2	119.9(3)
C5	C4	C3	119.8(3)
C6	C5	C4	122.4(3)
C7	C6	C5	116.1(3)
C2	C7	N1	105.4(3)
C6	C7	N1	131.4(3)
C6	C7	C2	123.3(3)

Atom	Atom	Atom	Angle/°
N2b	N1b	Cl1b	122.4(3)
C7b	N1b	Cl1b	124.3(2)
C7b	N1b	N2b	113.3(3)
C1b	N2b	N1b	105.4(3)
C2b	C1b	N2b	111.4(3)
C3b	C2b	C1b	136.8(3)
C7b	C2b	C1b	105.1(3)
C7b	C2b	C3b	118.1(3)
C2b	C3b	Br1b	118.4(2)
C4b	C3b	Br1b	121.4(3)
C4b	C3b	C2b	120.2(3)
C5b	C4b	C3b	119.8(3)
C6b	C5b	C4b	122.5(3)
C7b	C6b	C5b	116.0(3)
C2b	C7b	N1b	104.8(3)
C6b	C7b	N1b	131.7(3)
C6b	C7b	C2b	123.5(3)

Table 5: Torsion Angles in ° for **SAS-05-051-an**.

Atom	Atom	Atom	Atom	Angle/°
Br1	C3	C2	C1	2.5(4)
Br1	C3	C2	C7	-179.5(3)
Br1	C3	C4	C5	178.9(3)

Atom	Atom	Atom	Atom	Angle/°
Cl1	N1	N2	C1	-179.2(3)
Cl1	N1	C7	C2	178.8(3)
Cl1	N1	C7	C6	-2.0(4)
N1	N2	C1	C2	1.2(3)
N1	C7	C2	C1	-0.3(3)
N1	C7	C2	C3	-178.9(3)
N1	C7	C6	C5	178.4(4)
N2	N1	C7	C2	1.1(3)
N2	N1	C7	C6	-179.8(3)
N2	C1	C2	C3	177.6(3)
N2	C1	C2	C7	-0.6(3)
C1	N2	N1	C7	-1.4(3)
C1	C2	C3	C4	-178.5(4)
C1	C2	C7	C6	-179.5(3)
C2	C3	C4	C5	-0.1(4)
C2	C7	C6	C5	-2.6(4)
C3	C2	C7	C6	1.9(4)
C3	C4	C5	C6	-0.7(4)
C4	C3	C2	C7	-0.4(4)
C4	C5	C6	C7	1.9(4)
Br1b	C3b	C2b	C1b	-1.4(4)
Br1b	C3b	C2b	C7b	179.9(3)
Br1b	C3b	C4b	C5b	-178.9(3)
Cl1b	N1b	N2b	C1b	178.3(3)
Cl1b	N1b	C7b	C2b	-178.3(3)
Cl1b	N1b	C7b	C6b	1.6(4)
N1b	N2b	C1b	C2b	0.3(3)
N1b	C7b	C2b	C1b	-0.3(3)
N1b	C7b	C2b	C3b	178.8(3)
N1b	C7b	C6b	C5b	-178.0(4)
N2b	N1b	C7b	C2b	0.5(3)
N2b	N1b	C7b	C6b	-179.6(3)
N2b	C1b	C2b	C3b	-178.8(3)
N2b	C1b	C2b	C7b	0.0(3)
C1b	N2b	N1b	C7b	-0.5(3)
C1b	C2b	C3b	C4b	178.0(5)
C1b	C2b	C7b	C6b	179.8(3)
C2b	C3b	C4b	C5b	1.6(4)
C2b	C7b	C6b	C5b	1.9(4)
C3b	C2b	C7b	C6b	-1.2(4)
C3b	C4b	C5b	C6b	-0.8(4)
C4b	C3b	C2b	C7b	-0.7(4)
C4b	C5b	C6b	C7b	-0.9(4)

Table 6: Hydrogen Fractional Atomic Coordinates (×10^4) and Equivalent Isotropic Displacement Parameters (Å^2×10^3) for **SAS-05-051-an**. U_{eq} is defined as 1/3 of the trace of the orthogonalised U_{ij}.

Atom	x	y	z	U_{eq}
H1	8950(30)	3080(120)	3798(14)	25(11)
H4	5960(20)	-480(110)	2813(18)	23(10)
H5	4680(30)	1430(130)	3395(15)	23(10)
H6	5170(30)	4750(160)	4140(20)	57(15)
H1b	6400(30)	7180(110)	6201(14)	16(9)
H4b	3260(30)	5110(130)	7176(19)	42(13)
H5b	2040(30)	7380(170)	6565(17)	56(19)

Atom	x	y	z	U_{eq}
H6b	2610(30)	9520(120)	5764(17)	31(12)

Citations

CrysAlis^{Pro} (Rigaku Oxford Diffraction), Rigaku Oxford Diffraction, Poland (?).

L.J. Bourhis and O.V. Dolomanov and R.J. Gildea and J.A.K. Howard and H. Puschmann, The Anatomy of a Comprehensive Constrained, Restrained, Refinement Program for the Modern Computing Environment - Olex2 Disected, *Acta Cryst. A*, (2015), **A71**, 59-71.

O.V. Dolomanov and L.J. Bourhis and R.J. Gildea and J.A.K. Howard and H. Puschmann, Olex2: A complete structure solution, refinement and analysis program, *J. Appl. Cryst.*, (2009), **42**, 339-341.

Sheldrick, G.M., ShelXT-Integrated space-group and crystal-structure determination, *Acta Cryst.*, (2015), **A71**, 3-8.
